# *In silico* modelling of chelate stabilized tetrylene derivatives†

**DOI:** 10.1039/d4ra01515k

**Published:** 2024-03-27

**Authors:** Alex-Cristian Tomut, Iulia-Andreea Aghion, Raluca Septelean, Ioan-Dan Porumb, Ionut-Tudor Moraru, Gabriela Nemes

**Affiliations:** a Faculty of Chemistry and Chemical Engineering, Department of Chemistry, Babeş-Bolyai University 1 M. Kogalniceanu Street RO-400084 Cluj-Napoca Romania ionut.moraru@ubbcluj.ro gabriela.nemes@ubbcluj.ro

## Abstract

The steric and electronic effects of specific ligands can play crucial roles in stabilizing unsaturated tetrylene species. In this work, hybrid density functional theory (DFT) methods, quantum theory of atoms in molecules (QTAIM) investigations and natural bond orbital (NBO) calculations are employed to evaluate the stabilization of low-valent E(ii) centers (E = Si, Ge, Sn, Pb) through the chelating effect generated by an electron-rich ligand containing the P=C–P=X moiety (X = O or S). Based on several types of analyses, such as the bond dissociation energy (BDE) or the interplay between attractive (*i.e.*, charge-transfer) and repulsive (*i.e.*, Pauli-exchange) effects, we highlight that the stabilization energy induced by chelation is up to *ca.* 70 kcal mol^−1^ for silylenes, yet slightly decreases within the heavier analogues. Moreover, it is emphasized that chelate-stabilized silylenes can form highly stable hybrid metal–metalloid complexes with transition metals (*e.g.*, gold). Due to push–pull effects occurring in the X→Si(ii)→Au fragment, the Si(ii)→Au bonding is significantly stronger than the X→Au, P(sp^2^)→Au or π(C=P)→Au donor–acceptor bonds, which are potentially formed by the electron-rich P=C–P=X unit with the AuCl fragment. These findings are supported by energy decomposition analysis (EDA) calculations.

## Abstract 2

The steric and electronic effects of specific ligands can play crucial roles in stabilizing unsaturated tetrylene species.

## Graphical abstract

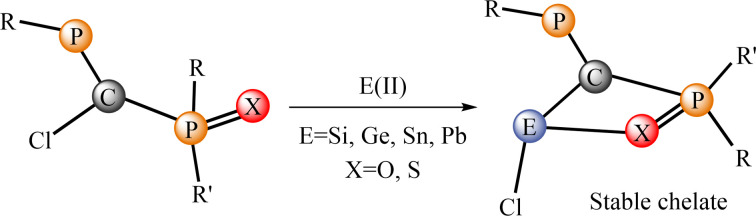

## Introduction

The chemistry of derivatives containing a low-valent p-block element E(ii) (*e.g.*, E = Si, Ge, Sn) is currently on the rise following the multiple applications that these systems can exhibit. The Lewis acid/base behavior of such derivatives can modulate their electronic properties and thus tailor their catalytic efficiency.^1,2^ Isolation of heavier carbene analogues can be achieved by using specific ligands that exhibit steric protection and/or stabilizing electronic effects.^3–6^ Therefore, ligand design plays a crucial role in the development of this chemistry. An appealing yet scarcely employed ligand is the diphosphaalkenyl derivative containing the P=C–P(Cl)=X moiety (X = S or O).^7,8^ Due to its multiple connection sites, this electron-rich system can potentially stabilize the E(ii) species. The presence of halogen substituents on one of the phosphorus atoms, or on the central carbon atom, makes such ligands as suitable building blocks for larger organometallic derivatives.^9,10^ Similarly phosphaalkenyl systems containing the P=C–P moiety have previously been employed as ligands for transition metal complexes.^11^ Monodentate or bidentate coordination compounds of P=C–P ligands with d-metals, *e.g.*, palladium, tungsten^12^ or gold,^13^ involving one or both of the phosphorus atoms have been isolated and fully characterized.

Considering the stabilization of tetrylene species using electron-rich ligands, several experimental studies have been previously conducted in our group using phosphaalkenyl –P=C <svg xmlns="http://www.w3.org/2000/svg" version="1.0" width="10.400000pt" height="16.000000pt" viewBox="0 0 10.400000 16.000000" preserveAspectRatio="xMidYMid meet"><metadata> Created by potrace 1.16, written by Peter Selinger 2001-2019 </metadata><g transform="translate(1.000000,15.000000) scale(0.011667,-0.011667)" fill="currentColor" stroke="none"><path d="M480 1160 l0 -40 -40 0 -40 0 0 -40 0 -40 -40 0 -40 0 0 -40 0 -40 -40 0 -40 0 0 -40 0 -40 -40 0 -40 0 0 -40 0 -40 -40 0 -40 0 0 -80 0 -80 40 0 40 0 0 40 0 40 40 0 40 0 0 40 0 40 40 0 40 0 0 40 0 40 40 0 40 0 0 40 0 40 40 0 40 0 0 40 0 40 40 0 40 0 0 40 0 40 40 0 40 0 0 40 0 40 -80 0 -80 0 0 -40z M80 480 l0 -80 40 0 40 0 0 -40 0 -40 40 0 40 0 0 -40 0 -40 40 0 40 0 0 -40 0 -40 40 0 40 0 0 -40 0 -40 40 0 40 0 0 -40 0 -40 80 0 80 0 0 40 0 40 -40 0 -40 0 0 40 0 40 -40 0 -40 0 0 40 0 40 -40 0 -40 0 0 40 0 40 -40 0 -40 0 0 40 0 40 -40 0 -40 0 0 40 0 40 -40 0 -40 0 0 40 0 40 -40 0 -40 0 0 -80z"/></g></svg> ^14–17^ or OCO-pincer type units.^18–22^ However, to the best of our knowledge, there are no reported studies on diphosphaalkenyl derivatives of P=C–P(Cl)=X type as stabilizing ligands for E(ii) derivatives. Considering thus the multiple connection possibilities of the P=C–P(Cl)=X unit to a metalloid fragment, a systematic study in which the Lewis acid/base properties of the partners are evaluated is mandatory. Using *first principles* calculations, it is possible to explore whether these ligands act as potential stabilizing agents for heavier carbene analogous through multiple connection possibilities.^17^

Herein, we describe an extensive DFT study regarding the ability of the P=C–P=X backbone to act as a stabilizing chelating ligand for low-valent E(ii) centers (E = heavy group 14 element). The steric and electronic effects of such ligands are systematically taken into account, while the Lewis acid/base behavior of the E(ii) species stabilized through chelation is also evaluated. Moreover, in the last section of this work we discuss the possible formation of novel *hybrid* complexes (*i.e.*, *hybrid* whereas they involve a *metal–metalloid* bond), species that are presumably stabilized *via push–pull* electronic effects occurring within the X→E(ii)→M moiety.

## Results and discussions

**Scheme 1:**
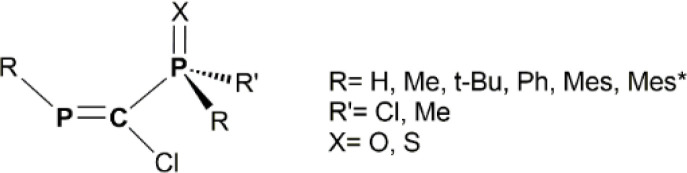
General representation of the RP=C(Cl)–P(X)RR′ model systems considered in the current DFT study.

**Scheme 2:**
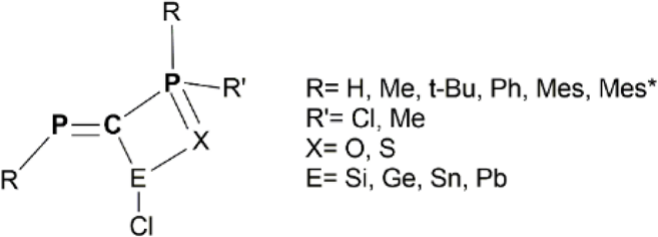
General representation of the investigated RP=C(E(ii)–Cl)–P(X)RR′ model systems.

**Scheme 3:**
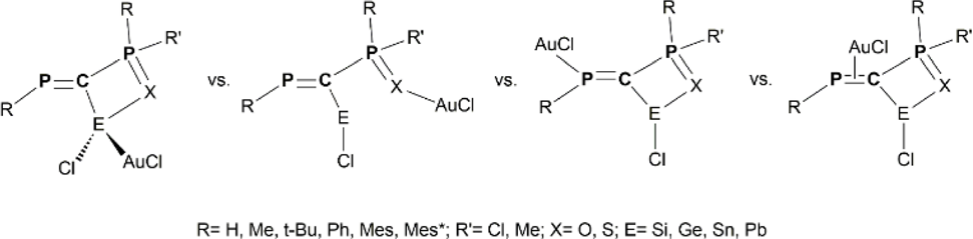
Schematic representation of model {R-P=C(E(ii))(Cl)–P(X)RR′}AuCl coordination isomers obtained *via* E(ii)→Au, X→Au, P(sp^2^)→Au and π(C=P)→Au bonds.

In this work, we combine hybrid DFT methods with NBO, AIM and EDA techniques to assess the structural and electronic properties of various model derivatives that incorporate the P=C–P=X (X = O or S) fragment (Scheme 1), as well as the stabilization of low-valent E(ii) atoms (E = Si, Ge, Sn, Pb) by such electron-rich units (Scheme 2). In fact, the main goal of this study is to theoretically design and evaluate the stability of a new class of chelate stabilized tetrylene derivatives. The X→E(ii) donation occurring within the chelate rings increases the electron density at the E(ii) atom, which can subsequently act as a donor for transition metals (*e.g.*, Au complexes, as illustrated Scheme 3).

### PCPX model derivatives

Even though the main target is to design by theoretical means novel tetrylene derivatives stabilized through the chelating effect of the P=C–P=X unit, it is first necessary to understand the structural and electronic features of the protecting ligands. Thus, we consider in the current DFT investigation several model derivatives with the RP=C(Cl)–P(X)RR′ general formula (Scheme 1), species that can be seen as precursors for ligands containing the P=C–P=X backbone. For simplicity, these precursors are abbreviated throughout the text as PCPX (X = O or S). Given the electron-donor properties and the multiple connection capabilities of the P=C–P=X moieties, it is appealing to assess whether their coordination ability can be tailored by playing with the volume and/or the electronegativity of R and R′ groups attached to the two phosphorus atoms. For this purpose, we investigate various R substituents of different bulkiness ranging from hydrogen to 2,4,6-tri-*tert*-butylphenyl (*i.e.*, Mes*) groups, while for R′ groups we employ either methyl or chlorine substituents.

The molecular structures of model PCPX ligands were optimized at the DFT (PBE0/Def2-TZVP) level of theory, with their most relevant geometrical parameters being presented in Table S1.† It must be recalled that the structural properties of model compounds containing the P=C–P=O fragment have already been described in previous DFT studies by our group^23–25^ and therefore, a detailed structural characterization of the PCPO models employed herein is beyond the purposes of the current study. However, theoretical data reported for derivatives incorporating the P=C–P=S moiety are scarce.^9^ Further insights into their features are required, as well as a systematic comparison between the PCPO and PCPS systems. The impact of R and R′ substituents on the electronic and structural properties of PCPX derivatives is evaluated, allowing thus an improved design of targeted ligands. DFT calculations reveal for the PCPO systems that the length of the bonds within the P=C–P=O unit is impacted only to a small extent by the bulkiness of the R group, or by the electronegativity of R′ substituent. The P=C bond distance is found to be the same within 1/100 Å in all model systems, while C–P and P=O bond lengths slightly increase with the bulkiness of R groups. The C–P=O angle narrows with the increasing volume of R, while the opposite is found for the P=C–P unit (Table S1†). The PCPS models reveal similar trends, as P=C bond lengths are found to be the same within 1/100 Å in all the investigated systems, while the equilibrium C–P and P=S distances are to some extent influenced by the volume of R groups. The C–P=S angle narrows with the increasing bulkiness of R, opposite to the P=C–P angle (Table S1†). It should also be mentioned that for both PCPO and PCPS systems, the sterically hindered (Mes*)P=C(Cl)–P(X)(Mes*)R′ model reveals a *ca.* 20° bending of the benzene ring of the Mes* group linked to the P(sp^3^) atom. Such peculiar structural behavior has nevertheless been reported in the literature.^26–29^

**Fig. 1:**
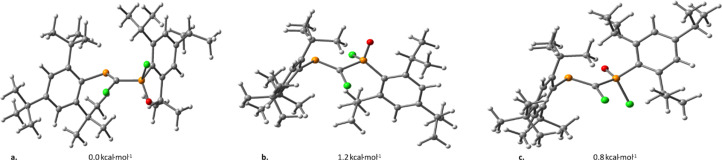
Three different conformers of (Mes*)P=C(Cl)–P(O)(Mes*)(Cl) model compound, obtained *via* rotations around the σ(P–C) bond, along with their calculated relative energies. The equilibrium values of the Cl–C–P=O dihedrals are (a) 56.0°, (b) 91.9° and (c) 173.6°.

The conformational analysis of model PCPX systems has also been carried out, study in which we mainly assess conformers obtained through rotation around the σ(P–C) bond of the P=C–P=X moiety. Based on DFT calculations performed on several conformers, it is shown that the rotational energy is lower than 2 kcal mol^−1^ for all model compounds. As an example, three different rotational conformations of the (Mes*)P=C(Cl)–P(O)(Mes*)(Cl) system, displaying Cl–C–P=O dihedral angles of *ca.* 60° (Fig. 1a), 90° (Fig. 1b) and 180° (Fig. 1c), vary within an energy range of 1.2 kcal mol^−1^.

### PCPX–E(ii) systems

The next type of systems investigated in this study are those with the RP=C(E(ii)–Cl)–P(X)RR′ general formula (Scheme 2). Such tetrylene species are stabilized through a X→E(ii) dative bond that leads to the formation of a chelate ring. These model compounds are abbreviated as PCPX–E(ii) throughout the text. To the best of our knowledge, PCPX–E(ii) chelate species have never been reported so far in the literature. However, several tetrylene derivatives stabilized by phosphaalkenyl –P=C units (*i.e.*, compounds with the RP=C(R)–E(ii)–R (E = Si, Ge, Sn) general formula) were previously synthesized and fully characterized by our research group.^14,16,17^ Thus, given our expertise in the field, we consider that the chelating effect induced by the electron-rich PCPX ligands could play crucial roles in stabilizing tetrylene derivatives. The theoretical analysis of various PCPX–E(ii) model systems also brings a great opportunity of shedding light on the nature of organometallic C–E(ii) bonds (E = Si, Ge, Sn, Pb). Based on computational analyses we evaluate the manner in which different types of R and R′ substituents, as well as different types of E(ii) atoms affect the molecular geometries, the electronic structure and the stability of targeted tetrylenes.

The molecular geometries of PCPX–E(ii) model compounds were optimized at the DFT (PBE0/Def2-TZVP) level of theory. According to the calculations, the PCPX–E(ii) species of different E(ii) atoms share many common structural features, the main difference between them being related to the equilibrium distance of the C–E(ii) bond. Such distances increase, as expected, from Si to Pb (Tables S2–S5†). As in the case of PCPX ligands (see the above section), the bulkiness of R groups or the different electronegativity of R′ substituents influence only to a lesser extent the equilibrium length of P=C, C–P, P=X, C–E(ii) chemical bonds, or the widening of P=C–P and C–P=X bonding angles. Besides these geometrical parameters, special attention is paid to the donor–acceptor X–E(ii) bonding, because an increased X→E(ii) electron-donation would stabilize the low-valent E(ii) atom. According to the DFT data, the X–E(ii) bond length is somewhat impacted by the type of R substituents, whereas it slightly increases with the bulkiness of the R groups. The electronegativity of R′ groups also affects to a lesser extent the X–E(ii) bonds distances, increasing slightly for more electronegative substituents (Tables S2–S5†). Comparisons of the equilibrium X→E(ii) bond distances and a reference length obtained by summing the covalent radii of E and X atoms indicate that they are expected to be considerably strong for a coordinate bond. For instance, O–Si(ii) bond lengths of investigated PCPO–Si(ii) model systems range between 1.867 Å and 1.919 Å, distances that are close to those expected for a sigma Si–O bond length (1.79 Å according to the sum of covalent radii of Si and O). Similar findings are reported for the S–Si(ii) bonds of PCPS–Si(ii) systems, given that calculated S–Si(ii) bond lengths, ranging between 2.396 and 2.451 Å, are slightly larger than the expected 2.19 Å length for a simple covalent Si–S bond (Table S2†). For the other investigated tetrylenes, the equilibrium X–E(ii) distances (X = O, S; E = Ge, Sn, Pb) reveal similar trends (see Tables S3–S5 in the ESI†). DFT calculations also highlight that the chelate rings formed through the X→E(ii) coordination are not planar, deviations from planarity being influenced by the nature of R substituent. For any given series of PCPX–E(ii) models (with X and E(ii) being the same for the entire series), the smallest deviations from planarity are obtained for R = *t*-Bu groups while the largest ones for R = H or Mes* substituents. Comparisons between PCPX–E(ii) derivatives of different E(ii) systems (*i.e.*, with the same X atom and R group) have additionally shown that deviations from planarity of C–P–X–E(ii) chelates are hardly affected by the nature of E atom (Tables S2–S5†).

The strength of the X→E(ii) bonds is further analysed. The increased stability of targeted tetrylenes can be related to the formation of C–P=X–E(ii) chelate rings, and involves *inter alia* thermal stabilization due to the formation of X→E(ii) donor–acceptor bonds, or the stabilization of the lone pair electrons (LP) at E atom, known as the inert-pair effect.

**Fig. 2:**
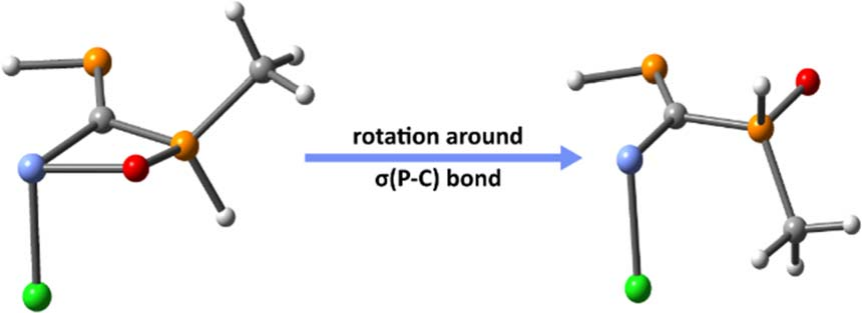
The chelate structure (left) and the geometry obtained by rotating the P–C bond with 180° (right), for the particular case of (H)P=C(Si(ii)–Cl)–P(O)(H)(Me) model compound. The bond dissection energy (BDE) energy corresponds to the difference between the two structures.

A rough estimate of the X→E(ii) bond dissociation energy (BDE) is obtained by rotating the P–C bond with 180°, which results into a new isomer that precludes the formation of the donor–acceptor bond (Fig. 2). The accuracy of such an approach should be reasonably high, bearing in mind that for PCPX ligands (*i.e.*, in the absence of E(ii) atom) the rotation around the P–C bond is barrierless, the energy gap between such rotational conformations being <2 kcal mol^−1^ (see Fig. 1). Hence, in order to estimate the BDE of the X→E(ii) bonds, two different approaches are taken into account: (i) one in which the molecular structure of the non-chelating isomer is optimized (*relaxed* approximation) and (ii) another approach in which the resulting geometry obtained by rotation around the σ(P–C) bond is preserved (*unrelaxed* approximation, which involves only *single-point* calculations). In both cases, BDEs are determined as the difference between the computed energy of the chelating structure and the energy of the geometry obtained through P–C bond rotation.

**Fig. 3:**
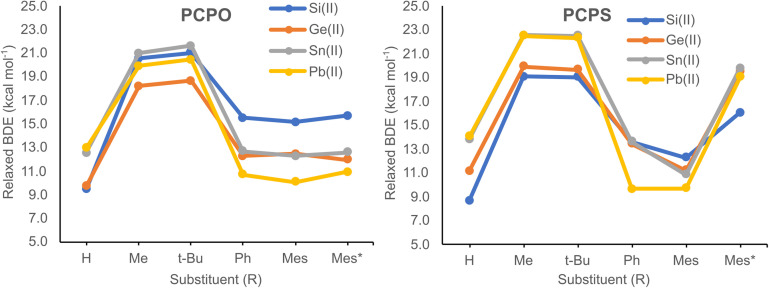
*Relaxed* BDE values for the O–E(ii) bonds of PCPO–E(ii) model compounds (left) and the S–E(ii) bonds PCPS–E(ii) model systems (right), plotted as a function of the R substituent.

Calculated BDEs obtained *via* the *relaxed approximation* are illustrated in Fig. 3, while numerical values are presented in Table S6.† Relaxed BDEs vary within the range of 9.0–22.5 kcal mol^−1^, their values being highly dependent on the type of R group (Fig. 3). For both PCPO and PCPS systems, the highest BDEs are obtained for the (Me)P=C(E(ii)–Cl)–P(X)(Me)(Me) and (*t*-Bu)P=C(E(ii)–Cl)–P(X)(*t*-Bu)(Me) model compounds (*i.e.*, PCPX systems with R = Me and *t*-Bu). Concerning the other investigated model derivatives, the lower BDE values are explained in terms of structural reorganizations that tend to minimize energy losses derived from breaking the C–P–X–E(ii) chelate ring. As an example, for the (H)P=C(E(ii)–Cl)–P(X)(H)(Me) model systems, upon geometry optimization the H atom bound to the P(sp^2^) atom migrates into a bridging coordination mode between the P(sp^2^) and the E atoms (Fig. S1†), which tends to compensate the energy loss due to the breaking of X→E(ii) bond. As for the model systems with R = Ph, Mes or Mes*, the energy loss is minimized through charge transfers that occur from the π electrons of the phenyl rings into vacant orbitals on the E atom (Fig. S2†). Nevertheless, even for the (Me)P=C(E(ii)–Cl)–P(X)(Me)(Me) and (*t*-Bu)P=C(E(ii)–Cl)–P(X)(*t*-Bu)(Me) systems, the energy loss is compensated by the strengthening of E(ii)–C and P=X bonds. This is reflected into the equilibrium distances of E(ii)–C and P=X bonds, which are considerably shorter within the non-chelating isomer than in the chelate one. As an example, the chelate structures of (Me)P=C(Si(ii)–Cl)–P(O)(Me)(Me) and (*t*-Bu)P=C(Si(ii)–Cl)–P(O)(*t*-Bu)(Me) compounds exhibit Si(ii)–C and P=O bond distances of *ca.* 1.99 Å and 1.56 Å, respectively, while the non-chelating geometries of *ca.* 1.86 Å and 1.48 Å, respectively. The same behaviour is noticed for the (Me)P=C(Si(ii)–Cl)–P(S)(Me)(Me) and (*t*-Bu)P=C(Si(ii)–Cl)–P(S)(*t*-Bu)(Me) systems, whereas the equilibrium length of Si(ii)–C and P=S bonds are with *ca.* 0.100 Å and 0.065 Å shorter in the non-chelating isomers. Similar trends are noticed for the other investigated PCPX–E(ii) systems (X = O, S; E = Ge, Sn, Pb). Therefore, a more accurate estimate of the BDEs is probably obtained through the *unrelaxed* approach. According to this analysis, calculated BDEs reveal much higher values compared to those obtained *via* the *relaxed* approximation (Table 1). It is also shown that *unrelaxed* BDE values are significantly higher for the X–E(ii) bonds (X = O or S) of PCPO–E(ii) systems than for the PCPS–E(ii) counterparts, particularly for the PCPX–Si(ii) models for which calculated differences between the *unrelaxed* BDEs of O–Si(ii) and S–Si(ii) bonds are at least 20 kcal mol^−1^.

**Table 1:** Calculated *unrelaxed* BDE values (kcal mol^−1^) for the O–E(ii) and S–E(ii) bonds of several model PCPO–E(ii) and PCPS–E(ii) systems

R	Si(ii)	Ge(ii)	Sn(ii)	Pb(ii)
**PCPO–E(** **ii** **)**				
H	62.1	55.3	51.8	44.7
Me	66.6	57.5	55.0	48.9
*t*-Bu	68.5	58.3	55.2	51.4

**PCPS–E(** **ii** **)**				
H	41.4	42.1	42.0	40.1
Me	43.0	42.3	42.3	40.6
*t*-Bu	45.2	43.2	46.0	48.2

Similar trends are highlighted for the PCPX–Ge(ii) and PCPX–Sn(ii) systems (X = O or S), albeit the gap between the *unrelaxed* BDE values of O–E(ii) and S–E(ii) bonds (E = Ge or Sn) is lower than in the previous case: *e.g.*, differences of *ca.* 13–15 kcal mol^−1^ are computed between the BDEs of O–Ge(ii) and S–Ge(ii) bonds, and of *ca.* 9–13 kcal mol^−1^ between O–Sn(ii) and S–Sn(ii) bonds. Concerning the PCPX–Pb(ii) systems, the energy differences between the *unrelaxed* BDEs of O–Pb(ii) and S–Pb(ii) bonds are much lower, namely 3–8 kcal mol^−1^. As a general trend, the strength of the O–E(ii) bonds of PCPO–E(ii) models (E = Si, Ge, Sn, Pb) decrease with the increasing atomic number of E (*i.e.*, from PCPO–Si(ii) systems to PCPO–Pb(ii) ones), while the strength of the S–E(ii) bonds of PCPS–E(ii) systems is barely affected by the nature of E(ii) atom. These differences can be explained in terms of secondary electronic interactions that occur within E-O bonds, such as LP(O)→σ*(E–R) hyperconjugations and LP(O)→d(E) back-donations, and which according to previous theoretical studies decrease drastically from Si to Sn, or Pb in this case.^30–32^

In order to gain insights into the nature of the X–E(ii) coordinate bonds (X = O or S; E = Si, Ge, Sb, Pb), Quantum Theory of Atoms in Molecules (QTAIM) analyses were carried out on several model systems. Based on this technique, the nature of bonds is described in terms of the topology of the electron density. Computed values of selected indices at the bond critical point (BCP) of σ(E–X) bonds are represented in Table 2. It is noticed that the electron density (*ρ*) has slightly larger values for the PCPO–E(ii) systems than for the PCPS–E(ii) ones, suggesting an increased X–E(ii) bond strength for the former. The calculated *ρ* values, which are in all cases <0.1 a.u. (specific for closed shell interactions) gradually decrease from silylenes to plumbylenes, suggesting the weakening of the X–E(ii) bond from Si to Pb. All these findings are in line with data obtained from the BDE calculations, thus strengthening our understanding of the X–E(ii) bond picture.

**Table 2:** Selected electronic density proprieties at the bond critical point of σ(X–E) bonds for several model PCPX–E(ii) systems

E(ii)	R	*ρ* (a.u.)	∇^2^(*ρ*) (a.u.)	*H* (a.u.)
**PCPO–E(** **ii** **)**				
Si(ii)	Me	0.0847	0.2967	−0.0334
	Mes*	0.0846	0.2929	−0.0335
Ge(ii)	Me	0.0794	0.2192	−0.0251
	Mes*	0.0787	0.2120	−0.0250
Sn(ii)	Me	0.0665	0.2234	−0.0132
	Mes*	0.0642	0.2117	−0.0123
Pb(ii)	Me	0.0591	0.1974	−0.0088
	Mes*	0.0549	0.1812	−0.0075

**PCPS–E(** **ii** **)**				
Si(ii)	Me	0.0635	−0.0257	−0.0328
	Mes*	0.0658	−0.0214	−0.0348
Ge(ii)	Me	0.0606	0.0273	−0.0218
	Mes*	0.0630	0.0281	−0.0233
Sn(ii)	Me	0.0505	0.0690	−0.0110
	Mes*	0.0520	0.0712	−0.0117
Pb(ii)	Me	0.0475	0.0768	−0.0086
	Mes*	0.0484	0.0787	−0.0090

Another evaluated index is the Laplacian of the electron density (∇^2^(*ρ*)). For PCPO–E(ii) systems, the computed ∇^2^(*ρ*) values gradually decrease from Si to Pb, but the opposite is found for the PCPS–E(ii) counterparts. Moreover, the ∇^2^(*ρ*) values computed for the PCPO–E(ii) structures are considerably larger than those for PCPS–E(ii) systems, which suggest a greater tendency of electron localization within the O–E(ii) bonds of the former. This can be explained based on electronegativity differences, which is much higher for the O atom than for the S atom. In the particular case of PCPS–Si(ii) systems, ∇^2^(*ρ*) values are negative, which suggest an increased covalent character of the S–Si(ii) bonds. The total energy (H) at the BCP is also analysed. The computed H-values for all X–E(ii) bonds are negative, their absolute value decreasing gradually from Si to Pb, for both PCPO–E(ii) and PCPS–E(ii) systems. Based on the computed H indices, it can be concluded that all X–E(ii) bonds are closed-shell in nature and exhibit an increased amount of covalency.

The nature of the X–E(ii) coordinate bonds (X = O or S; E = Si, Ge, Sb, Pb) was also evaluated by means of NBO explorations, which were carried out on the evaluated PCPO–E(ii) and PCPS–E(ii) model systems. For each case, two different types of derivatives were systematically considered: (i) one in which the steric repulsions are basically absent, *i.e.*, the (Me)P=C(E(ii)–Cl)–P(X)(Me)(Me) compounds, and (ii) a system with increased steric hindrance, *i.e.*, the (Mes*)P=C(E(ii)–Cl)–P(X)(Mes*)(Me) models. Based on NBO analyses, we show for all (Me)P=C(E(ii)–Cl)–P(X)(Me)(Me) model compounds that the X–E(ii) chemical bonds are highly polar covalent bonds, the contribution of E(ii) to this bonding decreasing in all cases from Si to Pb (Table S7†). As for the bulkier (Mes*)P=C(E(ii)–Cl)–P(X)(Mes*)(Me) model systems, with the exception of the PCPS–E(ii) derivatives containing Si(ii), Ge(ii) and Sn(ii), the X–E(ii) bonding is regarded by NBO calculations as a donor–acceptor bond formed through electron-transfers from X's LPs into the vacant p orbital on the E atom. For the series of (Mes*)P=C(E(ii)–Cl)–P(O)(Mes*)(Me) derivatives, the computed strength of the O→E(ii) bonds is 114.0 kcal mol^−1^ for PCPO–Si(ii), 94.5 kcal mol^−1^ for PCPO–Ge(ii), 76.0 kcal mol^−1^ for PCPO–Sn(ii) and 53.9 kcal mol^−1^ for PCPO–Pb(ii) model system, charge-transfer interactions of significantly increased magnitude. Nevertheless, according to previous computational studies, NBO-based second-order perturbation theory interactions tend to overestimate the strength of donor–acceptor (supramolecular) bonds.^33^ Several other studies indicate that in order to obtain accurate estimates of the strength of charge-transfer bonds it is necessary to take into account, along with electron-donations, the Pauli repulsions that occur between filled orbitals from the donor and acceptor atoms.^34–36^ Such Pauli-exchange repulsions are thus regarded as pertinent corrections to the charge-transfer component of donor–acceptor bonds.^37^ For the O→E(ii) bonding (E = Si, Ge, Sn, Pb) of model (Mes*)P=C(E(ii)–Cl)–P(O)(Mes*)(Me) systems, the most relevant Pauli repulsion are of LP(O)⋯p(E), LP(O)⋯σ(E–C) and LP(O)⋯σ(E–Cl) type. According to the NBO calculations, the total repulsion energy derived from these Pauli-exchange interactions is *ca.* 40.5 kcal mol^−1^ for PCPO–Si(ii), 29.6 kcal mol^−1^ for PCPO–Ge(ii), 23.9 kcal mol^−1^ for PCPO–Sn(ii) and 21.4 kcal mol^−1^ for PCPO–Pb(ii). Thus, the ‘corrected’ charge-transfer energies obtained by offsetting the attractive and repulsive components of the O→E(ii) bonds reveal more realistic estimates of their strength: *i.e.*, 73.5 kcal mol^−1^ for O→Si(ii), 64.9 kcal mol^−1^ for O→Ge(ii), 52.1 kcal mol^−1^ for O→Sn(ii) and 32.5 kcal mol^−1^ for O→Pb(ii).

**Fig. 4:**
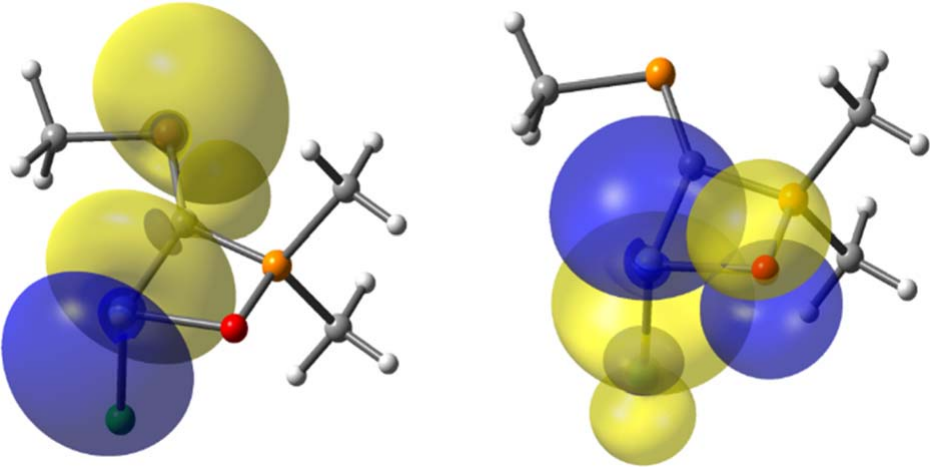
Pre-orthogonal NBOs describing the orbital overlaps of LP(P)→σ*(Si–C) (left) and LP(O)→σ*(Si–Cl) (right) electronic effects occurring in the (Me)P=C(Si(ii)–Cl)–P(O)(Me)(Me) system. The interaction-energies are computed within the framework of the second-order perturbative analysis (E2PERT) of the NBO theory and reveal values of *ca.* 7 kcal mol^−1^ for LP(P)→σ*(Si–C) and 5 kcal mol^−1^ for LP(O)→σ*(Si–Cl).

Besides X–E(ii) bonding, the orbital description of the other bonds formed by the tetrylene species E(ii) with the C and Cl atoms are displayed (Table S7†). NBO calculations indicate that E(ii) participates in all bonds (*i.e.*, O–E(ii), Cl–E(ii) and C–E(ii) bonds) with nearly pure p orbitals, thus highlighting a poor hybridization of the AOs of E(ii). In addition, it is shown that the organometallic C–E(ii) covalent bond is significantly strengthened by secondary electronic effects, such as the LP(P)→σ*(E-R), LP(P)→σ*(E–Cl), LP(X)→p(E) or π(C=P)→σ*(E–Cl) donations (Fig. 4). The computed energies of these interactions are presented in the ESI (see Table S8†).

Based on NBO data, it is also shown that for all investigated model derivatives, the tetrylene atom possess a LP which exhibits a predominant s AO character (*i.e.*, inert-pair effect), in line with the lack of hybridization of E(ii). The s orbital character increases with the atomic number, from Si (*ca.* 75%) to Pb (*ca.* 93%) (Table 3), results that are supported by previous theoretical data on tetrylene systems.^17,38^

**Table 3:** Atomic orbital (AO) composition of LP(E) for (Me)P=C(E(ii)–Cl)–P(X)(Me)(Me) and (Mes*)P=C(E(ii)–Cl)–P(X)(Mes*)(Me) model systems. Charges on E(ii) atom were computed within the framework of the Natural Population Analysis (NPA) of the NBO theory

E(ii)	R	% s of LP(E)	% p of LP(E)	Occup. of LP(E)	Charge of E(ii)
**PCPO–E(** **ii** **)**					
Si(ii)	Me	75.00	25.00	1.934	0.909
	Mes*	73.97	25.55	1.945	0.910
Ge(ii)	Me	84.52	15.48	1.971	1.005
	Mes*	83.88	16.12	1.975	0.993
Sn(ii)	Me	86.91	13.09	1.982	1.100
	Mes*	87.17	12.83	1.985	1.133
Pb(ii)	Me	92.31	7.69	1.988	1.145
	Mes*	93.04	6.96	1.989	1.214

**PCPS–E(** **ii** **)**					
Si(ii)	Me	76.99	23.01	1.938	0.718
	Mes*	76.32	23.68	1.943	0.722
Ge(ii)	Me	84.78	15.22	1.969	0.800
	Mes*	84.42	15.58	1.972	0.788
Sn(ii)	Me	87.18	12.82	1.980	0.924
	Mes*	87.52	12.48	1.983	0.961
Pb(ii)	Me	92.43	7.57	1.987	0.985
	Mes*	92.97	7.03	1.988	1.047

Considering that PCPX–E(ii) chelates are electron-rich species, their ability to form complexes with transition metals is further evaluated. Particularly, we are interested in the coordination through the LP on the E(ii) atom since the possible formation of a strong E(ii)→M (M = transition metal) bonding can lead to new classes of complexes. Such *hybrid* complexes (*i.e.*, *hybrid* whereas they involve a *metal–metalloid* bond) are expected to exhibit particular electronic features, which can be exploited in various applications, *e.g.*, homogenous catalysis. Yet, it is first necessary to assess the electron-donor properties of E(ii) which are expected to be enriched due to the X→E(ii) donation. Within a qualitative approach, we compare the natural charges computed for the E(ii) atoms of PCPX–E(ii) systems (Table 3) with charges of the E atoms of some standard compounds that contain E–X bonds (X = O or S). Hence, we choose for comparisons the (Me_3_E)-X-(EMe_3_) derivatives (X = O or S; E = Si, Ge, Sn, Pb), for which the computed natural charges of the E atoms are presented in Table 4.

**Table 4:** Natural charges computed for the E atoms of model (Me_3_E)–X–(Me_3_E) derivatives (X = O or S; E = Si, Ge, Sn, Pb), used as a standard for the charges of E(ii) atoms of PCPX–E(ii) chelating systems

Compound	NPA charge of the E atom			
	Si	Ge	Sn	Pb
(Me_3_E)–O–(Me_3_E)	1.905	1.883	1.847	1.607
(Me_3_E)–S–(Me_3_E)	1.525	1.511	1.489	1.286

It is noticed that the charges of the E atoms of the standard compounds are in all cases significantly more positive than those of corresponding E(ii) atoms of PCPX–E(ii) systems. This suggests an increased electron density at the E atoms of PCPX–E(ii) tetrylenes, particularly for the silicon derivatives. Precisely, the computed natural charge of the Si atom in (Me_3_Si)_2_O derivative is with *ca.* 1.0 higher than that of the Si atoms of PCPO–Si(ii) systems, and with *ca.* 0.8 for the Si atom of (Me_3_Si)_2_S compound than those of PCPS–Si(ii) model derivatives. This is most probably due to strong X→Si(ii) electron donations occurring within the PCPX–Si(ii) systems, while weaker X→E(ii) (E = Ge, Sn, Pb) bonds can motivate the higher charges computed for the E(ii) atoms of heavier tetrylenes. An increased electron density at the E(ii) centre can also favour the formation with transition metals of strong E(ii)→M coordinate bonds. Yet, this is expected especially for silylenes, not only because of the higher charge density at the Si(ii) atom, but also because of the inert-pair effect which is more pronounced in heavier tetrylene species.

### PCPX–E(ii)–AuCl

Based on the electronic features of PCPX–E(ii) systems, we further evaluate their potential role as ligands for metal complexes. We choose gold complexes as a case study mainly because they usually exhibit monodentate-binding modes, thereby allowing an insightful picture of the metal–metalloid bonding. Moreover, previous studies carried out by our group on germylene derivatives stabilized by P=C units and N-heterocyclic carbenes (*e.g.*, as in the (NHC)Ge(CCl=PMes*)_2_ compounds) revealed their ability to form isolable complexes involving the Ge(ii)→Au bonding,^14^ thus reinforcing our choice for Au as a potential candidate for novel chelate-stabilized *hybrid* complexes. Herein, we evaluate by DFT explorations several model complexes with the {RP=C(E(ii)–Cl)–P(X)RR′}AuCl formula (Scheme 3), special attention being paid to the strength of the E(ii)→Au bonding (E = Si, Ge, Sn, Pb). These model systems are abbreviated throughout the text as PCPX–E(ii)–AuCl (X = O or S; E = Si, Ge, Sn, Pb). Given that the electron-rich PCPX–E(ii) ligands exhibit multiple connection sites, we systematically compare the energies of PCPX–E(ii)–AuCl complexes obtained though E(ii)→Au bonds with those of other complexes potentially formed *via* P(sp^2^)→Au, X→Au (X = O or S) and π(P=C)→Au electron-donations (Scheme 3). The most relevant geometrical features computed for model complexes displaying E(ii)→Au bonds are presented in the ESI (Tables S9–S12†).

DFT calculations reveal that the strength of the coordination bonds formed by the PCPX–E(ii) systems with the AuCl moiety is highly dependent on the nature of the E(ii) metalloid atom (E = Si, Ge, Sn, Pb). Particularly for the PCPX–Si(ii)–AuCl model derivatives, the most stable isomers are in all cases those obtained through Si(ii)→Au bonds (Table 5). The relative energies of the coordination isomers obtained *via* P(sp^2^)→Au bonds are at least 16 kcal mol^−1^ higher within the PCPO–Si(ii)–AuCl systems, respectively 13 kcal mol^−1^ within the PCPS–Si(ii)–AuCl ones, the computed gap slightly increasing with the bulkiness of R substituents (*e.g.*, the largest energy difference is obtained when R = Mes*). Similar trends are noticed for the isomers displaying π(P=C)→Au bonds, with the special mention that steric hindrance has an increased impact on the relative stability of these model complexes. Regarding the isomers achieved through X→Au (X = O or S) donations, their stability is significantly lower than those involving Si(ii)→Au bonds, especially for the PCPO–Si(ii)–AuCl model complexes (Table 5). Differences between the relative energies of PCPO–Si(ii)–AuCl and PCPS–Si(ii)–AuCl systems formed *via* X→Au donations are also observed, yet expected. These discrepancies are explained based on hard/soft Lewis acid/base (HASB) theory in terms of an increased strength of S→Au bonds compared to O→Au ones.^39–41^

**Table 5:** Computed relative enthalpies (kcal mol^−1^) of PCPO–Si(ii)–AuCl and PCPS–Si(ii)–AuCl coordination isomers obtained through Si(ii)→Au, X→Au (X = O or S), P(sp^2^)→Au and π(C=P)→Au donations. In all cases, the relative enthalpies are calculated with respect to the coordination isomer exhibiting Si(ii)→Au bonds

R	R′	PCPO–Si(ii)–AuCl				PCPS–Si(ii)–AuCl			
		Si(ii)→Au	O→Au	P→Au	π(C=P)→Au	Si(ii)→Au	S→Au	P→Au	π(C=P)→Au
H	Me	0.0	48.1	18.9	21.3	0.0	31.2	17.0	19.2
	Cl	0.0	47.6	17.6	19.2	0.0	30.3	15.7	16.7
Me	Me	0.0	48.8	16.8	23.2	0.0	32.4	14.7	21.0
	Cl	0.0	48.4	15.8	21.7	0.0	31.2	13.8	19.1
*t*-Bu	Me	0.0	50.4	17.5	25.2	0.0	35.8	15.8	23.2
	Cl	0.0	50.2	16.5	23.7	0.0	34.8	14.8	21.6
Ph	Me	0.0	49.4	16.1	25.4	0.0	32.7	13.9	22.8
	Cl	0.0	49.1	15.4	24.2	0.0	31.2	13.0	20.8
Mes	Me	0.0	54.1	19.6	28.8	0.0	36.9	15.3	27.1
	Cl	0.0	51.5	18.3	27.7	0.0	37.0	14.4	25.3
Mes*	Me	0.0	54.7	25.3	41.0	0.0	31.3	22.3	41.3
	Cl	0.0	50.4	23.4	40.7	0.0	32.0	20.8	40.9

Concerning the PCPX–E(ii)–AuCl complexes of heavier tetrylenes (E = Ge, Sn, Pb), DFT calculations suggest that the coordination isomers obtained through E(ii)→Au donations are, with few exceptions for the PCPX–Ge(ii)–AuCl models, less stable than isomers displaying P(sp^2^)→Au or even π(C=P)→Au bonds (see ESI, Tables S13–S15†). Precisely, for the PCPX–Ge(ii)–AuCl systems (X = O or S) that involve bulky substituents (*i.e.*, R = Mes*) the isomers obtained *via* Ge(ii)→Au bonds are with *ca.* 4–7 kcal mol^−1^ more stable than those displaying P(sp^2^)→Au connections, but for the other model complexes the molecular energies of such coordination isomers are comparable (Table S13†). In few cases, the latter are even more stable, such as the {(Ph)P=C(Ge(ii)–Cl)–P(O)(Ph)(Cl)}AuCl model complex, for which the P(sp^2^)→Au donation leads to an isomer that is 2.8 kcal mol^−1^ lower in energy than the one obtained through Ge(ii)→Au bond. The same behaviour is noticed for the {(Ph)P=C(Ge(ii)–Cl)–P(S)(Ph)(Cl)}AuCl counterpart, for which the computed energy gap is 2.4 kcal mol^−1^. The other potential coordination isomers formed by the chelate-PCPX–Ge(ii) ligand with the AuCl moiety (*i.e.*, through π(C=P)→Au or X→Au bonds) exhibit in most cases considerably lower stabilities than their reference systems. Regarding the PCPX–Sn(ii)–AuCl complexes (X = O or S), the DFT data highlight that the P(sp^2^)→Au donation is favoured in all cases, while isomers obtained *via* Sn(ii)→Au bonds are with *ca.* 4–13 kcal mol^−1^ higher in energy. In addition, with the exception of bulkier PCPX–E(ii) ligands (*i.e.*, protected by Mes or Mes* groups), the PCPX–Sn(ii)–AuCl complexes (X = O or S) formed *via* the π(C=P)→Au donation are more stable than those involving the Sn(ii)→Au one (Table S14†). As for the PCPX–Pb(ii)–AuCl model complexes (X = O or S), the coordination isomers formed through the Pb(ii)→Au bonding are in most cases considerably less stable than all other investigated isomers (Table S15†). All these trends are explained in terms of a weakening of the E(ii)→Au bond with the increasing atomic number of E (from Si to Pb), which is closely related to the inert-pair effect, *i.e.*, the increasing in *s* character of LP(E) from Si to Pb (see Table 2). Other factors that potentially impact the strength of E(ii)→Au bonds are (i) the electron density at the E(ii) centre or (ii) the magnitude of the d(Au)→σ*(E–Y) (Y = C, Cl or X) back-bonding interactions. The first one is closely related to the X→E(ii) donation, coordinate bond that increases the charge density at E(ii) through chelation.

**Fig. 5:**
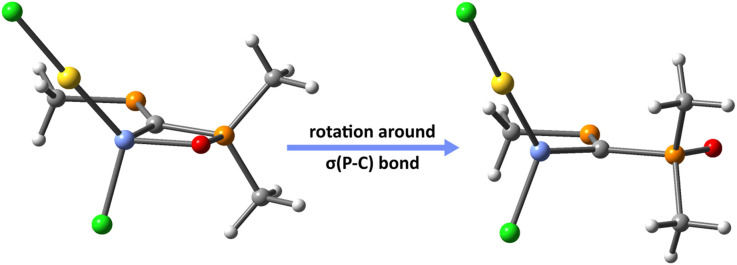
Au complexes involving chelate (left) and non-chelate (right) PCPX–E(ii) ligands, illustrated for {(Me)P=C(Si(ii)–Cl)–P(O)(Me)(Me)}AuCl systems as a particular case.

Thus, in order to understand the influence of X→E(ii) on the strengthening of E(ii)→Au bond, we re-optimize the molecular geometries of the PCPX–E(ii)–AuCl complexes formed *via* E(ii)→Au donation, by rotating the σ(P–C) bond of the PCPX–E(ii) ligand with 180° (Fig. 5). This results into new coordination isomers lacking the X→E(ii) bonds, which are in all cases significantly less stable than the complexes involving the chelate PCPX–E(ii) ligands, especially for silylene systems. In fact, the stabilization effect due to chelation is gradually decreased from silylenes to plumbylenes (Table S16†) and can be motivated in terms of a *push–pull* effect occurring in the X→E(ii)→Au unit. Regarding the d(Au)→σ*(E–Y) (Y = C, Cl or X) back-donations, NBO calculations suggest that their interaction-energies decrease considerably from silylenes to plumbylenes (Table S17†), further explaining the weaking of E(ii)→Au bonds from Si to Pb.

In order to gain further insights into the coordinate bonds formed by the AuCl fragment with the PCPX–E(ii) chelate ligands, EDA calculations were performed. Based on these analyses, the energies of E(ii)→Au, X→Au, P(sp^2^)→Au and π(C=P)→Au donations occurring within model {RP=C(E(ii)–Cl)–P(X)(Me)R}AuCl complexes (E = Si, Ge, Sn, Pb; R = Me, Mes*; X = O, S) have been computed, revealing similar trends as those highlighted above. In short, for the PCPX–Si(ii)–AuCl systems Si(ii)→Au bonds are considerably strong, with calculated interaction energies of *ca.* −80 to −90 kcal mol^−1^. These bond energies are in absolute values with 20–30 kcal mol^−1^ stronger than the P(sp^2^)→Au and π(C=P)→Au ones, the computed gap with respect to the energy X→Au donations being even greater (Tables 6 and S18†). Besides bond energies, EDA calculations shed more light on the nature of these coordinate bonds, whereas the total interaction energy between the donor atom and Au can be decomposed into several contributions, such as electrostatic, exchange-repulsion and orbital relaxation. For example, the Si(ii)→Au coordinate bond of {(Mes*)P=C(E(ii)–Cl)–P(X)(Me)(Mes*)}AuCl model complexes displays a relative large value of the orbital polarization energy (*ca.* 125–130 kcal mol^−1^), meaning that the orbitals involved in this bonding undergo considerable change in their shape. This indicates an increased covalent character of the Si(ii)→Au bond. In addition, electrostatics play a crucial role in the formation of this bonding, being about 30% of the total stabilization energy. The other components of the Si(ii)→Au bonding are described in Table 6, as well as the components of the coordinate bonds possibly formed by the PCPX–E(ii) with Au. Concerning the coordinate bonds formed in the less sterically hindered {(Me)P=C(Si(ii)–Cl)–P(X)(Me)(Me)}AuCl complex, EDA calculations reveal similar descriptions (Table S18†). For PCPX–Ge(ii)–AuCl complexes, Ge(ii)→Au and P(sp^2^)→Au coordinate bonds have comparable energies in case of {(Me)P=C(Ge(ii)–Cl)–P(X)(Me)(Me)}AuCl complexes (*i.e.*, calculated interaction energies are *ca.* −63 kcal mol^−1^), with the former becoming slightly stronger in sterically hindered models, such as the {(Mes*)P=C(Ge(ii)–Cl)–P(X)(Me)(Mes*)}AuCl complexes. A detailed decomposition scheme of the total bond energy is presented in Table S19 (see ESI†). According to the EDA calculations, the computed components of Ge(ii)→Au bonds are overall smaller in absolute values than those of Si(ii)→Au bonds. Yet, the ratio of the electrostatic component in the total stabilization energy is higher in case of the former (*ca.* 40%). For the PCPX–Sn(ii)–AuCl complexes, Sn(ii)→Au donations reveal interaction energies of *ca.* −50 to −55 kcal mol^−1^, which are generally lower (in absolute values) than those of P(sp^2^)→Au and π(C=P)→Au bonds (Table S20†). Regarding PCPX–Pb(ii)–AuCl systems, the Pb(ii)→Au donations (*i.e.*, interaction energies of −33 to −38 kcal mol^−1^) are weaker than the other coordinate bonds formed by the PCPX–Pb(ii) chelate ligand with the AuCl fragment (Table S21†). For the complexes involving Sn(ii)→Au or Pb(ii)→Au donations, the electrostatic component of these bonds is lower than that of their lighter counterparts containing Si(ii)→Au or Ge(ii)→Au dative bonds. Concerning the X→Au, P(sp^2^)→Au or π(C=P)→Au bonds, of all investigated PCPX–E(ii)–AuCl systems, EDA data suggest that there is always an interplay between a larger covalent component (orbital relaxation) and a smaller electrostatic contribution.

**Table 6:** Calculated EDA parameters (kcal mol^−1^) for the {(Mes*)P=C(Si(ii)–Cl)–P(X)(Me)(Mes*)}AuCl model complexes obtained *via* Si(ii)→Au, X→Au, P→Au or π(C=P)→Au coordination

Interaction type (kcal mol^−1^)	PCPO–Si(ii)–AuCl				PCPS–Si(ii)–AuCl			
	Si(ii)→Au	O→Au	P→Au	π(CP)→Au	Si(ii)→Au	S→Au	P→Au	π(CP)→Au
Total interaction energy	−88.4	−32.9	−60.9	−57.9	−84.7	−49.0	−60.8	−54.5
Electrostatic interaction	−93.1	−45.0	−76.8	−102.4	−87.1	−57.5	−76.6	−93.6
Exchange–repulsion	154.4	65.7	144.2	201.4	147.7	101.9	144.2	189.5
Exchange interaction	−91.6	−34.8	−80.8	−109.7	−87.6	−56.6	−81.0	−102.4
Repulsion	245.9	100.5	225.0	311.2	235.4	158.5	225.1	291.8
Orbital relaxation	−129.2	−39.7	−105.0	−119.8	−124.6	−75.8	−104.8	−113.6

## Conclusions

In summary, the current research brings fundamental insights into the chemistry of low-valent E(ii) derivatives (E = Si, Ge, Sn, Pb) and their potential stabilization through charge-transfer electronic effects. Based on a systematic DFT study, we highlight for the first time that electron-rich ligands incorporating the P=C–P=X (X = O or S) moiety can efficiently act as chelate ligands for the E(ii) centre of heavier tetrylenes. According to the computed BDE, AIM and NBO data, the greatest stabilization due to chelation is achieved for the silicon derivatives, *i.e.*, species that are abbreviated throughout the text as PCPX–Si(ii) systems. The lower stability of the chelate-structures formed within the PCPX–Sn(ii) and PCPX–Pb(ii) systems is correlated with the decreasing strength of the X→E(ii) bonds, as the atomic number of E increases. Calculations also suggest that the bulkiness and the electronegativity of the substituents attached on the two phosphorus atoms impact the stabilization of targeted tetrylene systems only to a lesser extent. Comparisons between the computed charges of the E(ii) atoms of the PCPX–E(ii) systems with those of E(iv) atoms of some standard (Me_3_E)–X–(EMe_3_) compounds that contain E–X bonds (X = O or S) revealed that the former exhibit an increased electron density at the 14-group element atom, especially in silicon derivatives. Therefore, the ability of such PCPX–Si(ii) derivatives to form highly stable *hybrid* metal–metalloid complexes with gold has also been emphasized. Due to the *push–pull effects* occurring within the X→Si(ii)→Au fragment, the Si(ii)→Au bonding is significantly stronger than other bonds potentially formed by the P=C–P=X backbone with the AuCl moiety (*i.e.*, the P(sp^2^)→Au, X→Au (X = O or S) and π(P=C)→Au donations). Concerning the heavier PCPX–E(ii)–AuCl counterparts (E = Ge, Sn or Pb), DFT calculations suggest that the coordination isomers obtained through E(ii)→Au donations are in most cases considerably less stable than complexes obtained *via* P(sp^2^)→Au or π(C=P)→Au bonds. These findings are supported by EDA calculations. As a perspective, the current research could serve as a starting point for future experimental studies.

## Computational details

### Geometry optimizations and vibrational analyses

All calculations were performed within the framework of the Density Functional Theory (DFT), using the *Gaussian 09* software package.^42^ The molecular geometries of investigated systems were fully optimized in the gas phase without any symmetry constrains, with the optimization criteria being set to tight. In all DFT investigations, we employed the hybrid functional of Adamo and Barone, *i.e.*, PBE0,^43^ and the valence triple-zeta quality Def2-TZVP basis set.^44,45^ For the tin and lead atoms, the relativistic core electrons were replaced within calculations by effective core potentials (ECPs), such pseudo-potentials being already embedded in the *Gaussian 09* implemented version of Def2-TZVP basis set. Vibrational analyses were carried out in order to characterize the nature of the stationary points. Additionally, frequency calculations were used to compute molecular enthalpies within the framework of the harmonic oscillator approximation for vibrational contribution (*e.g.*, further details regarding the thermodynamic equations are available in ref. 46). The integration grid used was of 99 radial shells and 950 angular points for each shell (99 950), which is defined in *Gaussian 09* as the “ultrafine” grid.

### NBO calculations

Natural Bond Orbital (NBO)^47–49^ analyses were carried out on the optimized structures of the investigated species. Charges were computed within the framework of the Natural Population Analysis (NPA) of the NBO theory.^50^ The energies of donor–acceptor interactions were computed with second-order perturbation theory analysis,^51^ while energetic estimates of Pauli-exchange repulsions were determined by performing Natural Steric analyses.^52–54^ All these calculations were performed using the *NBO7.0* program.^55^

### AIM calculations

The Quantum Theory Atoms in Molecules (QTAIM) developed by Bader^56,57^ has been employed to gain insights into the bonding of investigated systems. This type of analysis focuses on the proprieties of the electron density at Bond Critical Point (BCP), the point of minimum electron density along the bond path (*i.e.*, the line of maximum electron density) between two atoms. Herein, we evaluate three different indices: the electron density (*σ*), the Laplacian of the electron density (∇^2^(*ρ*)) and the total energy (*H*). Regarding the Laplacian of the electron density, this index is known to show the depletion (∇^2^(*ρ*) > 0) or accumulation (∇^2^(*ρ*) < 0) of electron density in the internuclear region of two atoms. As for the total energy, H, literature data suggest that this index can also accurately describe chemical bonding, possibly even better than ∇^2^(*ρ*).^58,59^ In the current study, the *Gaussian 09* calculated wavefunctions were used to analyse the probability density topology. All QTAIM calculations were performed using the *AIMAll* program.^60^

### EDA calculations

Energy Decomposition Analysis (EDA), proposed by Li and Su,^61^ has been employed to compute the interaction energies of the coordinate bonds formed between the PCPX–E(ii) chelate ligands and the AuCl moiety. Within this technique the energy difference is decomposed as follows:Δ*E*_DFT_ = Δ*E*_ele_ + Δ*E*_ex–rep_ + Δ*E*_orb_ + Δ*E*_cor_where Δ*E*_ele_ is the electrostatic energy, Δ*E*_ex–rep_ the difference between exchange and repulsions Δ*E*_orb_ the orbital relaxation (*i.e.*, the polarization energy), and Δ*E*_cor_ the correlation energy. EDA calculations were performed in *Turbomole* software package (version 7.7).^62^ The wavefunctions of the *Gaussian* optimized structures were calculated using RI-DFT.^63–66^ The functionals and basis set employed in *Turbomole* are similar to those used in *Gaussian 09* for geometry optimizations, *i.e.*, PBE0, Def2-TZVP. For computing the integrals, a grid size of 5 was employed in all *Turbomole* calculations.

### BDE estimates

The heterolytic bond dissociation energy (BDE) of X→E(ii) coordinate bonds is obtained as the difference between the energies of chelated and non-chelated structures. The non-chelated system is obtained from the chelate geometry by rotating the σ(P–C) bond with 180°, which precludes the formation of the X→E(ii) donation. BDEs are assessed *via* two different approaches, as follows:

(i) *Relaxed approximation* – method in which the molecular structure of the non-chelating isomer is optimized. The *relaxed* BDE is calculated with the following formula:Relaxed BDE = *H*_chelated structure_ − *H*_non-chelated structure_where *H* comprises the electronic energy (*E*_ee_), the zero-point energy corrections (ZPE) and the thermal correction to enthalpy.

(ii) *Unrelaxed approximation* – involves only single-point calculations of the non-chelated structures obtained through rotation around the σ(P–C). The *unrelaxed* BDE is calculated with the following formula:Unrelaxed BDE = *E*_chelated structure_ − *E*_non-chelated structure_where *E* represents the electronic energy, without ZPE or thermal energy corrections.

## Conflicts of interest

The authors declare no conflicts of interest.

## Supplementary Material

RA-014-D4RA01515K-s001
