# Supplementary material for: In silico modelling of chelate stabilized tetrylene derivatives
Source: RSC Adv. 2024 Mar 27;14(15):10161–71. doi: 10.1039/d4ra01515k (PMC10966434; doi:10.1039/d4ra01515k)
Supplement: RA-014-D4RA01515K-s001 [file RA-014-D4RA01515K-s001.pdf]

Electronic Supplementary Material (ESI) for RSC Advances.
This journal is © The Royal Society of Chemistry 2024

Electronic Supplementary Information

In Silico Modelling of Chelate Stabilized Tetrylene Derivatives

Alex-Cristian Tomut, Iulia-Andreea Aghion, Raluca Septelean, Ioan-Dan Porumb, Ionut-Tudor Moraru* and Gabriela Nemes*

Faculty of Chemistry and Chemical Engineering, Department of Chemistry, Babeş-Bolyai University, 1 M. Kogalniceanu Street, RO-400084 Cluj-Napoca, Romania

E-mail: ionut.moraru@ubbcluj.ro; gabriela.nemes@ubbcluj.ro

Table of Contents

PCPX systems 2
Table S1. 2
PCPX-E(II) systems 3
Table S2. 3
Table S3. 4
Table S4. 5
Table S5. 6
Table S6. 7
Figure S1. 7
Figure S2. 7
Table S7. 8
Table S8. 8
PCPX-E(II)-AuCl systems 9
Table S9. 9
Table S10. 9
Table S11. 10
Table S12. 10
Table S13. 11
Table S14. 11
Table S15. 12
Table S16. 12
Table S17. 13
Table S18. 13
Table S19. 14
Table S20. 14
Table S21. 15

PCPX systems

Table S1. Selection of the most relevant geometrical parameters computed for model RP=C(Cl)-P(X)RR' derivatives incorporating the P=C(Cl)-P=X (X = O or S) moiety.

R	R'	PCPO					PCPS				
		P=C (Å)	C-P (Å)	P=O (Å)	C-P=O (°)	P=C-P (°)	P=C (Å)	C-P (Å)	P=S (Å)	C-P=S (°)	P=C-P (°)
**H**	**Me**	1.671	1.814	1.474	113.2	117.9	1.674	1.822	1.925	115.9	118.6
	**Cl**	1.673	1.802	1.463	114.6	117.1	1.675	1.811	1.903	117.1	116.9
**Me**	**Me**	1.672	1.815	1.477	111.7	117.4	1.673	1.818	1.934	113.0	118.8
	**Cl**	1.674	1.802	1.466	112.9	116.5	1.675	1.809	1.913	114.4	117.9
***t*-Bu**	**Me**	1.674	1.827	1.481	110.5	114.2	1.674	1.834	1.942	112.4	116.2
	**Cl**	1.676	1.817	1.469	111.6	113.0	1.678	1.828	1.921	113.1	114.6
**Ph**	**Me**	1.676	1.819	1.479	111.0	115.1	1.677	1.826	1.937	112.5	116.9
	**Cl**	1.678	1.807	1.468	112.3	114.2	1.677	1.821	1.915	114.3	122.1
**Mes**	**Me**	1.674	1.827	1.480	111.0	120.9	1.675	1.833	1.941	113.0	118.1
	**Cl**	1.677	1.814	1.468	111.1	119.0	1.676	1.820	1.919	114.0	117.2
**Mes***	**Me**	1.673	1.848	1.484	106.9	125.2	1.676	1.852	1.954	108.1	125.8
	**Cl**	1.675	1.836	1.471	107.8	123.7	1.677	1.842	1.928	109.0	124.2

PCPX-E(II) systems

Table S2. Selected geometrical features for the RP=C(Si(II)-Cl)-P(X)RR' (X = O or S) model systems. The sum of covalent radii for relevant bonds: $\Sigma_{Si\text{-}C}$=1.87 Å, $\Sigma_{Si\text{-}O}$=1.77 Å, $\Sigma_{Si\text{-}S}$=2.16 Å, $\Sigma_{P=O}$=1.59 Å. $\Sigma_{P=S}$=1.96 Å.

R	R'	P=C (Å)	C-P (Å)	P=X (Å)	X-Si(II) (Å)	Si(II)-C (Å)	C-P=X (°)	P=C-P (°)	X-Si(II)-C (°)	dev. pln. (°)
					**PCPO-Si(II)**					
H	Me	1.656	1.743	1.544	1.900	2.009	97.0	130.42	78.29	10.87
	Cl	1.657	1.727	1.533	1.919	2.007	98.4	130.6	77.94	13.81
Me	Me	1.654	1.741	1.553	1.875	1.99	96.4	130.3	78.97	6.81
	Cl	1.655	1.732	1.534	1.914	1.993	97.5	129.22	78.0	2.19
t-Bu	Me	1.659	1.754	1.557	1.867	1.997	96.3	125.23	79.39	4.23
	Cl	1.661	1.74	1.542	1.893	1.998	97.6	125.45	78.85	1.9
Ph	Me	1.661	1.744	1.552	1.877	1.985	96.7	126.31	79.33	3.15
	Cl	1.663	1.727	1.540	1.897	1.988	98.0	127.43	78.88	5.58
Mes	Me	1.657	1.757	1.555	1.87	1.983	94.6	134.11	78.47	6.4
	Cl	1.658	1.74	1.542	1.893	1.987	96.0	134.04	77.98	9.59
Mes*	Me	1.661	1.773	1.562	1.877	2.003	93.6	133.69	77.68	18.73
	Cl	1.661	1.751	1.541	1.912	2.003	95.2	133.61	76.91	16.94
					**PCPS-Si(II)**					
H	Me	1.658	1.751	1.996	2.451	1.972	99.2	127.33	79.71	17.73
	Cl	1.658	1.733	1.98	2.44	1.981	100.52	128.81	79.89	20.65
Me	Me	1.659	1.751	2.009	2.405	1.962	99.22	125.88	81.42	10.88
	Cl	1.659	1.732	1.993	2.398	1.97	100.23	127.82	81.29	15.01
t-Bu	Me	1.666	1.768	2.015	2.39	1.967	99.58	120	82.63	0.27
	Cl	1.667	1.75	1.997	2.396	1.976	100.78	121.08	82.16	4.17
Ph	Me	1.665	1.754	2.006	2.412	1.961	99.92	122.45	81.6	9.07
	Cl	1.666	1.734	1.989	2.416	1.967	101.05	124.94	81.4	13.01
Mes	Me	1.663	1.768	2.023	2.400	1.962	94.69	124.32	78.98	14.98
	Cl	1.663	1.748	2.005	2.398	1.968	96.0	125.84	78.93	22.25
Mes*	Me	1.668	1.779	2.028	2.384	1.984	96.9	128.6	81.01	17.65
	Cl	1.669	1.757	1.998	2.405	1.982	98.43	129.17	80.31	15.12

Table S3. Selected geometrical features for the RP=C(Ge(II)-Cl)-P(X)RR' (X = O or S) model systems. The sum of covalent radii for relevant bonds: $\Sigma_{Ge\text{-}C}$=1.96 Å, $\Sigma_{Ge\text{-}O}$=1.84 Å, $\Sigma_{Ge\text{-}S}$=2.24 Å, $\Sigma_{P=O}$=1.59 Å and $\Sigma_{P=S}$=1.96 Å.

R	R'	P=C (Å)	C-P (Å)	P=X (Å)	X-Si(II) (Å)	Si(II)-C (Å)	C-P=X (°)	P=C-P (°)	X-Si(II)-C (°)	dev. pln. (°)
**PCPO-Ge(II)**										
H	Me	1.654	1.748	1.533	2.079	2.107	99.86	129.58	73.87	13.32
	Cl	1.654	1.734	1.519	2.112	2.110	101.40	129.44	73.32	15.47
Me	Me	1.651	1.750	1.542	2.047	2.103	99.29	128.75	74.48	8.78
	Cl	1.652	1.734	1.527	2.074	2.104	100.64	129.19	73.97	11.35
t-Bu	Me	1.653	1.761	1.544	2.045	2.117	97.39	125.30	74.65	3.17
	Cl	1.655	1.748	1.527	2.078	2.115	100.62	125.32	74.02	1.44
Ph	Me	1.656	1.749	1.539	2.057	2.097	99.71	127.10	74.56	5.09
	Cl	1.657	1.733	1.526	2.083	2.098	101.04	127.96	74.05	6.69
Mes	Me	1.654	1.761	1.543	2.048	2.086	97.32	134.02	73.87	9.11
	Cl	1.658	1.733	1.526	2.085	2.097	101.04	127.96	74.05	6.70
Mes*	Me	1.657	1.770	1.549	2.053	2.117	96.85	135.01	73.20	19.30
	Cl	1.659	1.751	1.529	2.095	2.121	98.51	134.09	73.39	18.26
**PCPS-Ge(II)**										
H	Me	1.657	1.752	1.997	2.549	2.080	100.92	126.63	76.70	20.14
	Cl	1.656	1.733	1.980	2.548	2.091	102.58	128.20	76.73	22.38
Me	Me	1.655	1.753	2.010	2.506	2.078	100.92	124.88	78.06	13.52
	Cl	1.656	1.734	1.992	2.506	2.087	102.19	126.85	77.85	16.77
t-Bu	Me	1.656	1.770	2.014	2.500	2.092	101.65	119.93	78.99	1.41
	Cl	1.661	1.753	1.993	2.513	2.096	103.07	120.76	78.60	4.41
Ph	Me	1.660	1.753	2.005	2.526	2.076	101.68	123.39	78.04	11.10
	Cl	1.661	1.733	1.988	2.528	2.083	103.01	125.75	77.81	13.98
Mes	Me	1.659	1.766	2.018	2.504	2.069	96.38	128.72	75.65	18.95
	Cl	1.659	1.744	1.998	2.505	2.079	98.28	130.97	75.78	20.55
Mes*	Me	1.663	1.773	2.028	2.485	2.106	99.24	129.77	77.88	17.18
	Cl	1.664	1.752	1.997	2.511	2.101	100.88	130.02	77.19	15.54

Table S4. Selected geometrical features for the RP=C(Sn(II)-Cl)-P(X)RR' (X = O or S) model systems. The sum of covalent radii for relevant bonds $\Sigma_{Sn\text{-}C}$=2.15 Å and $\Sigma_{Sn\text{-}O}$=2.03 Å, $\Sigma_{Sn\text{-}S}$=2.43 Å, $\Sigma_{P=O}$=1.59 Å and $\Sigma_{P=S}$=1.96 Å.

R	R'	P=C (Å)	C-P (Å)	P=X (Å)	X-Si(II) (Å)	Si(II)-C (Å)	C-P=X (°)	P=C-P (°)	X-Si(II)-C (°)	dev. pln. (°)
**PCPO-Sn(II)**										
H	Me	1.654	1.752	1.529	2.261	2.307	102.67	127.08	68.32	14.54
	Cl	1.655	1.736	1.515	2.294	2.314	104.21	127.09	67.76	16.39
Me	Me	1.650	1.754	1.537	2.229	2.305	102.11	126.14	68.83	10.72
	Cl	1.651	1.738	1.522	2.256	2.309	103.46	126.72	68.32	13.02
t-Bu	Me	1.652	1.763	1.540	2.229	2.316	102.22	124.60	68.98	2.03
	Cl	1.653	1.750	1.523	2.260	2.316	103.45	124.63	68.42	1.02
Ph	Me	1.654	1.751	1.534	2.249	2.295	102.55	126.96	68.76	6.11
	Cl	1.655	1.734	1.520	2.276	2.295	103.83	127.95	68.25	7.19
Mes	Me	1.653	1.761	1.537	2.241	2.279	99.93	133.42	68.03	10.15
	Cl	1.654	1.743	1.522	2.273	2.283	101.59	133.61	67.57	11.42
Mes*	Me	1.655	1.768	1.543	2.248	2.315	99.79	135.45	67.52	19.40
	Cl	1.657	1.749	1.522	2.291	2.316	101.45	134.41	66.79	19.17
**PCPS-Sn(II)**										
H	Me	1.657	1.754	2.009	2.714	2.284	104.03	124.06	72.13	17.78
	Cl	1.656	1.733	1.982	2.722	2.298	105.42	126.23	77.96	24.15
Me	Me	1.654	1.756	2.013	2.677	2.285	103.58	122.77	72.91	16.17
	Cl	1.655	1.735	1.995	2.680	2.297	104.96	124.91	72.58	19.06
t-Bu	Me	1.658	1.771	2.017	2.672	2.298	104.48	118.98	73.81	3.35
	Cl	1.660	1.752	1.996	2.685	2.304	105.95	120.00	73.38	6.06
Ph	Me	1.658	1.753	2.007	2.705	2.277	104.38	123.37	72.77	12.30
	Cl	1.656	1.733	1.990	2.711	2.286	105.81	125.87	72.46	14.67
Mes	Me	1.659	1.764	2.019	2.684	2.268	98.89	128.01	70.52	20.23
	Cl	1.658	1.741	1.999	2.690	2.280	100.93	130.53	70.55	21.61
Mes*	Me	1.661	1.769	2.032	2.663	2.309	102.18	130.22	72.75	17.39
	Cl	1.663	1.751	2.000	2.690	2.308	103.67	129.93	71.98	16.97

Table S5. Selected geometrical features for the RP=C(Pb(II)-Cl)-P(X)RR' (X = O or S) model systems. The sum of covalent radii for relevant bonds Σ_{Pb-C}=2.19 Å, Σ_{Pb-O}=2.07 Å, Σ_{Pb-S}=2.47, Å $\Sigma_{P=O}$=1.59 Å and $\Sigma_{P=S}$=1.96 Å.

R	R'	P=C (Å)	C-P (Å)	P=X (Å)	X-Si(II) (Å)	Si(II)-C (Å)	C-P=X (°)	P=C-P (°)	X-Si(II)-C (°)	dev. pln. (°)
**PCPO-Pb(II)**										
H	Me	1.654	1.754	1.523	2.390	2.404	104.58	126.22	65.61	15.88
	Cl	1.654	1.737	1.510	2.424	2.412	106.19	126.33	65.03	17.59
Me	Me	1.649	1.757	1.532	2.355	2.403	103.97	125.39	66.08	13.03
	Cl	1.650	1.740	1.517	2.382	2.409	105.39	126.08	65.55	15.18
t-Bu	Me	1.650	1.764	1.533	2.361	2.409	104.16	124.85	66.20	0.16
	Cl	1.651	1.750	1.517	2.389	2.409	105.40	124.97	65.69	0.73
Ph	Me	1.652	1.751	1.528	2.385	2.390	104.41	127.65	65.83	7.79
	Cl	1.653	1.734	1.514	2.412	2.390	105.68	128.72	65.32	8.57
Mes	Me	1.651	1.759	1.530	2.379	2.371	101.64	134.30	65.05	11.53
	Cl	1.652	1.741	1.514	2.414	2.374	103.39	134.57	64.58	12.45
Mes*	Me	1.652	1.766	1.535	2.391	2.406	101.87	137.12	64.71	14.23
	Cl	1.653	1.745	1.514	2.442	2.405	103.69	136.16	63.94	18.97
**PCPS-Pb(II)**										
H	Me	1.657	1.753	2.000	2.805	2.386	105.06	123.68	69.60	24.37
	Cl	1.656	1.731	1.982	2.811	2.404	107.05	125.82	69.45	25.88
Me	Me	1.653	1.755	2.012	2.767	2.391	104.99	122.39	70.52	18.49
	Cl	1.653	1.733	1.995	2.770	2.405	106.48	124.88	70.19	21.17
t-Bu	Me	1.656	1.765	2.019	2.753	2.404	105.79	120.09	71.38	5.95
	Cl	1.657	1.744	1.999	2.763	2.413	107.26	121.46	70.94	8.75
Ph	Me	1.656	1.762	2.011	2.771	2.387	105.61	121.88	70.95	2.95
	Cl	1.655	1.740	1.987	2.809	2.404	106.79	124.16	69.66	19.48
Mes	Me	1.655	1.769	2.019	2.791	2.388	105.12	124.77	70.62	11.46
	Cl	1.657	1.752	1.988	2.822	2.385	106.61	125.64	69.84	13.42
Mes*	Me	1.658	1.765	2.031	2.757	2.409	103.86	131.45	70.44	16.26
	Cl	1.660	1.747	1.998	2.786	2.406	105.40	131.19	69.69	16.68

Table S6. Calculated *relaxed* BDE (kcal mol^{-1}) values for the O-E(II) and S-E(II) bonds of model PCPO-E(II) and PCPS-E(II) systems.

R	R'	PCPO-E(II)				PCPS-E(II)			
		Si(II)	Ge(II)	Sn(II)	Pb(II)	Si(II)	Ge(II)	Sn(II)	Pb(II)
**H**	Me	-9.4	-9.7	-12.6	-13.0	-8.7	-11.1	-13.8	-14.1
**Me**	Me	-20.5	-18.2	-21.0	-19.9	-19.1	-19.9	-22.5	-22.5
***t*-Bu**	Me	-21.0	-18.6	-21.6	-20.4	-19.0	-19.6	-22.5	-22.3
**Ph**		-15.5	-12.3	-12.7	-10.7	-13.6	-13.4	-13.6	-9.6
**Mes**	Me	-15.1	-12.4	-12.3	-10.1	-12.3	-11.2	-10.8	-9.7
**Mes***	Me	-15.7	-12.0	-12.6	-10.9	-16.0	-19.4	-19.8	-19.0

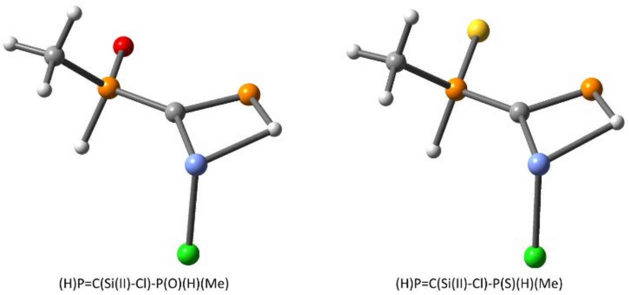

Figure S1. Equilibrium geometry of the (H)P=C(E(II)-Cl)-P(X)(H)(Me) model systems employed in the *relaxed* BDE approximation. The structures were obtained from the initial chelate geometry through rotation with 180° around the σ(P-C) bond. Following the geometry optimization the H atom bound to the P(sp^2) atom migrates into a bridging coordination mode between the P(sp^2) and the E atoms.

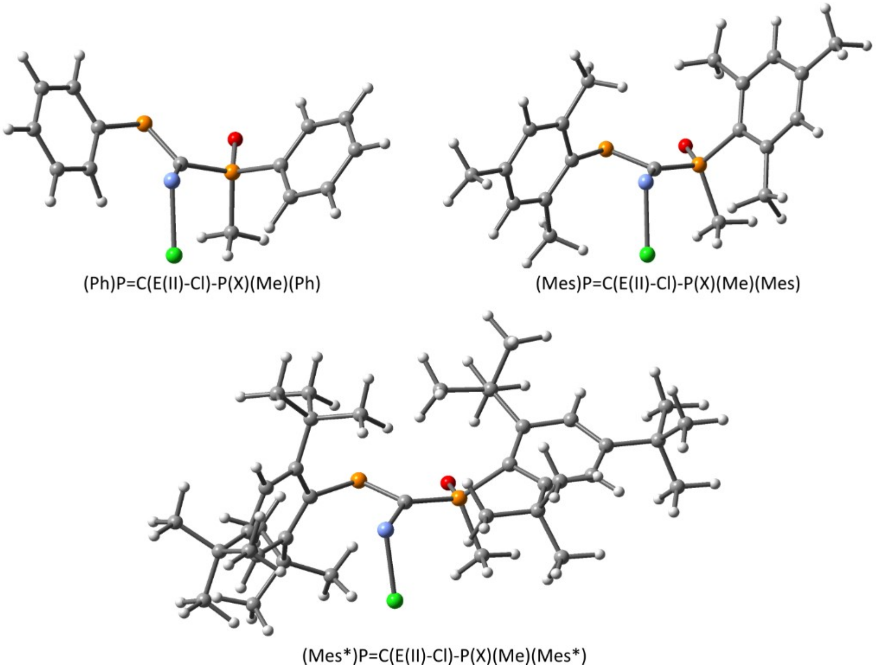

Figure S2. Equilibrium geometry of (Ph)P=C(E(II)-Cl)-P(X)(Ph)(Me), (Mes)P=C(E(II)-Cl)-P(X)(Ph)(Mes) and (Mes*)P=C(E(II)-Cl)-P(X)(Ph)(Mes*) model systems employed in the *relaxed* BDE approximation. The structures were obtained from the initial chelate geometry through rotation with 180° around the σ(P-C) bond.

Table S7. Atomic orbital (AO) composition of the σ(E-X), σ (E-C) and σ (E-Cl) sigma bonds, in the particular cases of (Me)P=C(E(II)-Cl)-P(X)(Me)(Me) and (Mes*)P=C(E(II)-Cl)-P(X)(Mes*)(Me) model systems.

E(II)	R	(%) E(II) in σ(X-E) bond	% p of E(II) in σ(X-E) bond	(%) E(II) in σ(C-E) bond	% p of E(II) in σ(C-E) bond	(%) E(II) in σ(Cl-E) bond	% p of E(II) in σ(Cl-E) bond
				**PCPO-E(II)**			
**Si(II)**	Me	11.12	90.22	20.01	88.13	19.97	89.97
	Mes*	-	-	19.96	85.93	20.01	88.54
**Ge(II)**	Me	8.83	94.06	18.53	92.30	17.67	94.03
	Mes*	-	-	18.84	89.42	-	-
**Sn(II)**	Me	7.56	95.29	16.82	93.49	16.19	94.58
	Mes*	-	-	16.28	91.23	-	-
**Pb(II)**	Me	6.70	96.72	16.43	95.63	15.23	96.55
	Mes*	-	-	14.92	94.19	-	-
				**PCPS-E(II)**			
**Si(II)**	Me	19.84	93.59	20.73	87.49	20.07	90.33
	Mes*	20.02	93.92	20.17	87.17	19.95	89.97
**Ge(II)**	Me	18.07	96.21	19.29	91.68	18.24	94.12
	Mes*	18.46	96.39	19.03	91.42	18.52	93.78
**Sn(II)**	Me	15.88	96.60	17.43	93.18	16.57	94.71
	Mes*	15.41	96.58	16.35	92.14	-	-
**Pb(II)**	Me	14.58	97.92	16.75	95.53	15.55	96.65
	Mes*	-	-	15.10	94.47	-	-

Table S8. Calculated interaction-energies (kcal mol^{-1}) of LP(P)→σ*(E-R), LP(X)→*p*(E) or π(C=P)→σ*(E-Cl) donations, as well as the total electron-donation energy, for several model PCPX-E(II) systems.

E(II)	R	LP(P)→σ*(E-R)	LP2(X)→σ*(E-Cl)	LP2(X)→LV(E)	π(C=P)→σ*(E-Cl)	Total donation
				**PCPO-E(II)**		
**Si(II)**	Me	7.07	5		1.74	13.81
	Mes*	6.64	5.08	11.07	1.54	24.33
**Ge(II)**	Me	6.79	3.43		1.56	11.78
	Mes*	6.9	6.33	9.4	2.94	25.57
**Sn(II)**	Me	6.52	2.34		0.75	9.61
	Mes*	7.32	4.11	8.27	1.56	21.26
**Pb(II)**	Me	6.73	1.79		0.57	9.09
	Mes*	7.39	2.8	7.63	1.22	19.04
				**PCPS-E(II)**		
**Si(II)**	Me	7.21	4.79		2.13	14.13
	Mes*	7.94	4.23		1.74	13.91
**Ge(II)**	Me	6.76	4.52		1.84	13.12
	Mes*	6.92	4.22		1.5	12.64
**Sn(II)**	Me	6.59	3.23		0.83	10.65
	Mes*	7.39	5.01	3.52	1.69	17.61
**Pb(II)**	Me	6.68	2.84		0.59	10.11
	Mes*	7.33	3.98	4.18	1.26	16.75

PCPX-E(II)-AuCl systems

Table S9. The most relevant geometrical data computed for the {RP=C(Si(II)-Cl)-P(X)(Me)R}AuCl model complexes obtained via Si→Au bonds.

X	R	P=X (Å)	X-Si (Å)	Si-C (Å)	Au-Si (Å)	dev. pln. (°)
O	H	1.556	1.814	1.947	2.236	3.29
	Me	1.567	1.794	1.932	2.242	3.66
	t-Bu	1.572	1.787	1.939	2.247	3.48
	Ph	1.568	1.787	1.932	2.246	2.53
	Mes	1.570	1.786	1.925	2.246	0.57
	Mes*	1.581	1.782	1.926	2.256	14.37
S	H	2.012	2.328	1.919	2.240	13.72
	Me	2.026	2.296	1.911	2.248	9.46
	t-Bu	2.032	2.285	1.920	2.253	2.04
	Ph	2.030	2.282	1.911	2.253	4.80
	Mes	2.038	2.277	1.908	2.251	14.88
	Mes*	2.049	2.267	1.918	2.260	14.31

Table S10. The most relevant geometrical data computed for the {RP=C(Ge(II)-Cl)-P(X)(Me)R}AuCl model complexes obtained via Ge→Au bonds.

X	R	P=X (Å)	X-Ge (Å)	Ge-C (Å)	Au-Ge (Å)	dev. pln. (°)
O	H	1.542	1.992	2.030	2.327	6.36
	Me	1.553	1.966	2.021	2.331	5.83
	t-Bu	1.556	1.958	2.030	2.336	3.27
	Ph	1.552	1.967	2.017	2.332	2.79
	Mes	1.555	1.960	2.010	2.335	6.88
	Mes*	1.564	1.958	2.024	2.340	16.76
S	H	2.008	2.439	2.011	2.332	16.20
	Me	2.023	2.401	2.006	2.338	12.27
	t-Bu	2.389	2.388	2.013	2.342	2.87
	Ph	2.021	2.408	2.004	2.339	9.69
	Mes	2.041	2.386	1.996	2.341	15.40
	Mes*	2.027	2.375	2.021	2.346	15.34

Table S11. The most relevant geometrical data computed for the {RP=C(Sn(II)-Cl)-P(X)(Me)R}AuCl model complexes obtained via Sn→Au bonds.

X	R	P=X (Å)	X-Sn (Å)	Sn-C (Å)	Au-Sn (Å)	dev. pln. (°)
O	**H**	1.534	2.191	2.227	2.502	7.93
	**Me**	1.543	2.165	2.216	2.504	7.84
	***t*-Bu**	1.547	2.161	2.221	2.507	2.19
	**Ph**	1.542	2.176	2.207	2.506	4.46
	**Mes**	1.545	2.174	2.194	2.508	8.47
	**Mes***	1.552	2.173	2.209	2.512	17.13
S	**H**	2.008	2.622	2.210	2.506	18.12
	**Me**	2.023	2.584	2.584	2.509	14.98
	***t*-Bu**	2.028	2.574	2.212	2.513	3.85
	**Ph**	2.019	2.602	2.197	2.511	11.34
	**Mes**	2.040	2.581	2.186	2.514	16.31
	**Mes***	2.046	2.565	2.211	2.517	15.92

Table S12. The most relevant geometrical data computed for the {RP=C(Pb(II)-Cl)-P(X)(Me)R}AuCl model complexes obtained via Pb→Au bonds.

X	R	P=X (Å)	X-Pb (Å)	Pb-C (Å)	Au-Pb (Å)	dev. pln. (°)
O	**H**	1.523	2.354	2.306	2.591	10.73
	**Me**	1.531	2.321	2.298	2.591	9.94
	***t*-Bu**	1.534	2.319	2.302	2.594	0.80
	**Ph**	1.528	2.346	2.285	2.593	6.34
	**Mes**	1.529	2.354	2.267	2.593	7.00
	**Mes***	1.535	2.352	2.287	2.598	16.92
S	**H**	2.000	2.743	2.297	2.595	21.04
	**Me**	2.015	2.702	2.294	2.596	16.91
	***t*-Bu**	2.020	2.692	2.302	2.600	4.52
	**Ph**	2.009	2.732	2.282	2.597	13.26
	**Mes**	2.021	2.724	2.269	2.600	18.54
	**Mes***	2.036	2.688	2.296	2.603	15.59

Table S13. Computed relative enthalpies (kcal mol^{-1}) of PCPO-Ge(II)-AuCl and PCPS-Ge(II)-AuCl coordination isomers obtained through Ge(II)→Au, X→Au (X = O or S), P(sp^2)→Au and π(C=P)→Au donations. The relative enthalpies are calculated with respect to the coordination isomer displaying Ge(II)→Au bonds.

R	R'	PCPO-Ge(II)-AuCl				PCPS-Ge(II)-AuCl			
		Ge(II)→Au	O→Au	P→Au	π(C=P)→Au	Ge(II)→Au	S→Au	P→Au	π(C=P)→Au
H	Me	0.0	27.4	0.5	2.6	0.0	14.4	0.6	2.6
	Cl	0.0	27.5	-0.5	0.9	0.0	13.8	-0.4	0.4
Me	Me	0.0	28.1	-1.7	4.1	0.0	15.8	-1.4	4.5
	Cl	0.0	28.2	-2.5	2.9	0.0	15.0	-2.1	2.7
t-Bu	Me	0.0	29.3	-1.1	6.1	0.0	19.0	-0.8	6.8
	Cl	0.0	29.5	-2.1	4.8	0.0	18.3	-1.6	5.4
Ph	Me	0.0	28.5	-2.1	6.4	0.0	16.4	-1.8	6.4
	Cl	0.0	28.5	-2.8	5.4	0.0	16.5	-2.4	4.8
Mes	Me	0.0	30.4	1.0	10.7	0.0	21.4	1.0	12.6
	Cl	0.0	31.0	0.1	9.8	0.0	21.5	0.1	10.6
Mes*	Me	0.0	29.9	6.7	23.7	0.0	14.5	5.9	26.0
	Cl	0.0	29.0	3.9	23.4	0.0	14.5	3.7	25.4

Table S14. Computed relative enthalpies (kcal mol^{-1}) of PCPO-Sn(II)-AuCl and PCPS-Sn(II)-AuCl coordination isomers obtained through Sn(II)→Au, X→Au (X = O or S), P(sp^2)→Au and π(C=P)→Au donations. The relative enthalpies are calculated with respect to the coordination isomer displaying Sn(II)→Au bonds.

R	R'	PCPO-Sn(II)-AuCl				PCPS-Sn(II)-AuCl			
		Sn(II)→Au	O→Au	P→Au	π(C=P)→Au	Sn(II)→Au	S→Au	P→Au	π(C=P)→Au
H	Me	0.0	16.2	-10.4	-8.2	0.0	4.4	-9.7	-7.5
	Cl	0.0	16.7	-11.1	-9.6	0.0	4.2	-10.3	-9.5
Me	Me	0.0	16.4	-12.7	-7.0	0.0	5.6	-11.6	-5.8
	Cl	0.0	17.0	-13.3	-8.0	0.0	5.1	-12.1	-7.4
t-Bu	Me	0.0	17.8	-11.8	-4.9	0.0	9.5	-10.9	-3.4
	Cl	0.0	18.5	-12.5	-6.0	0.0	8.9	-11.6	-4.9
Ph	Me	0.0	16.8	-12.7	-5.1	0.0	6.9	-11.5	-4.2
	Cl	0.0	17.2	-13.3	-5.9	0.0	6.5	-12.0	-5.6
Mes	Me	0.0	18.6	-9.5	-0.7	0.0	10.3	-7.4	1.9
	Cl	0.0	19.5	-10.4	-1.5	0.0	9.4	-9.8	-1.0
Mes*	Me	0.0	18.2	-4.2	12.4	0.0	4.4	-4.2	15.5
	Cl	0.0	18.4	-6.1	13.3	0.0	5.1	-5.9	15.4

Table S15. Computed relative enthalpies (kcal mol^{-1}) of PCPO-Pb(II)-AuCl and PCPS-Pb(II)-AuCl coordination isomers obtained through Pb(II)→Au, X→Au (X = O or S), P(sp^2)→Au and π(C=P)→Au donations. The relative enthalpies are calculated with respect to the coordination isomer displaying Pb(II)→Au bonds.

R	R'	PCPO-Pb(II)-AuCl				PCPS-Pb(II)-AuCl			
		Pb(II)→Au	O→Au	P→Au	π(C=P)→Au	Pb(II)→Au	S→Au	P→Au	π(C=P)→Au
**H**	**Me**	0.0	-1.2	-26.8	-24.9	0.0	-12.1	-25.8	-24.0
	**Cl**	0.0	-0.2	-27.1	-26.0	0.0	-12.0	-26.2	-25.8
**Me**	**Me**	0.0	-1.3	-29.2	-24.0	0.0	-11.4	-27.9	-22.8
	**Cl**	0.0	-0.3	-29.5	-24.8	0.0	-11.6	-28.2	-24.3
***t*-Bu**	**Me**	0.0	-0.6	-28.7	-22.3	0.0	-7.9	-27.8	-20.8
	**Cl**	0.0	0.7	-28.9	-22.9	0.0	-8.8	-28.3	-22.2
**Ph**	**Me**	0.0	-1.0	-28.9	-22.3	0.0	-10.2	-27.7	-21.3
	**Cl**	0.0	-0.2	-29.3	-22.9	0.0	-10.3	-28.1	-22.6
**Mes**	**Me**	0.0	0.6	-26.1	-17.8	0.0	-8.7	-25.7	-16.6
	**Cl**	0.0	1.7	-26.7	-18.5	0.0	-8.2	-26.2	-18.2
**Mes***	**Me**	0.0	-0.2	-21.0	-5.0	0.0	-13.2	-21.2	-2.3
	**Cl**	0.0	0.7	-22.7	-3.5	0.0	-12.1	-22.8	-2.2

Table S16. Relative energies (kcal mol^{-1}) of the PCPX-E(II)-AuCl complexes formed through the E(II)→Au donations, in which the X→E(II) donations are absent (i.e., geometries are obtained from the equilibrium chelate structures by rotating the σ(P-C) bond of the PCPX-E(II) ligand with 180°). The relative energies are calculated with respect to the complexes involving the chelate PCPX-E(II) ligands.

X	R	E			
		**Si**	**Ge**	**Sn**	**Pb**
**O**	**H**	20.3	13.7	15.9	12.2
	**Me**	30.7	23.6	23.7	19.0
	***t*-Bu**	31.5	23.8	24.4	19.7
	**Ph**	25.2	16.7	14.8	10.3
	**Mes**	24.8	17.3	14.4	9.3
	**Mes***	26.1	17.6	15.3	11.6
**S**	**H**	19.3	14.8	17.3	14.5
	**Me**	26.5	24.4	26.7	22.3
	***t*-Bu**	27.1	24.8	26.1	22.2
	**Ph**	20.0	17.3	16.0	12.6
	**Mes**	17.8	17.5	15.2	9.7
	**Mes***	23.1	20.4	19.0	19.0

Table S17. Total back-donation d(Au)→σ*(E-Y) energy (kcal mol^{-1}) arising from d(Au)→σ*(E-C), d(Au)→σ*(E-Cl) and d(Au)→σ*(E-X) interactions, for several {RP=C(E(II)-Cl)-P(X)(Me)R}AuCl model complexes that involve the E(II)→Au dative bond.

E	R'	PCPO-E(II)-AuCl	PCPS-E(II)-AuCl
		d(Au)→σ*(E-Y) (Y = C, Cl or O) (kcal/mol)	d(Au)→σ*(E-Y) (Y = C, Cl or S) (kcal/mol)
Si	Me	21.0	15.5
	Mes*	18.0	12.9
Ge	Me	16.5	12.8
	Mes*	15.0	11.2
Sn	Me	12.8	10.5
	Mes*	10.9	8.9
Pb	Me	9.4	7.8
	Mes*	9.2	7.9

Table S18. Calculated EDA parameters (kcal mol^{-1}) for several model {RP=C(Si(II)-Cl)-P(X)(Me)R}AuCl model complexes (R = Me or Mes*; X = O or S).

R	coord.mode	Total int. energy	Electrostatic Int.	Exchange-Rep.	Exchange Int.	Repulsion	Orbital Relaxation
				PCPO-Si(II)-AuCl			
Me	Si(II)→Au	-81.0	-84.1	143.8	-85.3	229.0	-123.1
	O→Au	-29.7	-41.2	59.8	-31.3	91.1	-37.7
	P→Au	-62.2	-74.9	138.1	-77.4	215.5	-106.8
	π(C=P)→Au	-57.8	-75.7	154.3	-82.4	236.7	-110.3
Mes*	Si(II)→Au	-88.4	-93.1	154.4	-91.6	245.9	-129.2
	O→Au	-32.9	-45.0	65.7	-34.8	100.5	-39.7
	P→Au	-60.9	-76.8	144.2	-80.8	225.0	-105.0
	π(C=P)→Au	-57.9	-102.4	201.4	-109.7	311.2	-119.8
R	coord.mode			PCPS-Si(II)-AuCl			
Me	Si(II)→Au	-77.7	-79.5	139.3	-82.5	221.9	-119.8
	S→Au	-43.2	-51.5	96.8	-53.4	150.3	-71.8
	P→Au	-61.5	-74.2	138.5	-77.5	215.9	-106.8
	π(C=P)→Au	-57.0	-75.2	155.0	-82.6	237.6	-110.0
Mes*	Si(II)→Au	-84.7	-87.1	147.7	-87.6	235.4	-124.6
	S→Au	-49.0	-57.5	101.9	-56.6	158.5	-75.8
	P→Au	-60.8	-76.6	144.2	-81.0	225.1	-104.8
	π(C=P)→Au	-54.5	-93.6	189.5	-102.4	291.8	-113.6

Table S19. Calculated EDA parameters (kcal mol^{-1}) for several model {RP=C(Ge(II)-Cl)-P(X)(Me)R}AuCl model complexes (R = Me or Mes*; X = O or S).

R	coord.mode	Total int. energy	Electrostatic Int.	Exchange-Rep.	Exchange Int.	Repulsion	Orbital Relaxation
		PCPO-Ge(II)-AuCl					
Me	Ge(II)→Au	-62.2	-61.3	106.4	-64.2	170.6	-90.8
	O→Au	-32.1	-44.2	63.0	-33.0	96.0	-40.0
	P→Au	-62.3	-74.8	138.3	-77.4	215.7	-107.0
	π(C=P)→Au	-59.0	-77.1	155.0	-83.0	238.1	-111.1
Mes*	Ge(II)→Au	-67.8	-68.2	113.2	-68.6	181.9	-94.2
	O→Au	-35.5	-48.1	68.9	-36.4	105.3	-42.2
	P→Au	-57.0	-89.0	177.0	-95.6	272.6	-111.0
	π(C=P)→Au	-55.0	-87.8	178.6	-95.8	274.4	-111.6
R	coord.mode	PCPS-Ge(II)-AuCl					
Me	Ge(II)→Au	-61.4	-59.2	104.7	-63.0	167.7	-90.1
	S→Au	-43.6	-52.2	97.6	-53.9	151.5	-72.4
	P→Au	-61.6	-74.2	138.6	-77.6	216.2	-106.9
	π(C=P)→Au	-57.9	-76.3	155.7	-83.0	238.7	-110.9
Mes*	Ge(II)→Au	-66.8	-65.6	111.2	-67.2	178.4	-93.3
	S→Au	-49.7	-58.3	102.7	-57.1	159.8	-76.4
	P→Au	-60.8	-76.2	143.6	-80.7	224.2	-104.7
	π(C=P)→Au	-53.5	-88.0	180.9	-96.8	277.7	-111.3

Table S20. Calculated EDA parameters (kcal mol^{-1}) for several model {RP=C(Sn(II)-Cl)-P(X)(Me)R}AuCl model complexes (R = Me or Mes*; X = O or S).

R	coord.mode	Total int. energy	Electrostatic Int.	Exchange-Rep.	Exchange Int.	Repulsion	Orbital Relaxation
		PCPO-Sn(II)-AuCl					
Me	Sn(II)→Au	-50.6	-43.0	82.1	-52.1	134.2	-74.8
	O→Au	-33.2	-45.8	64.5	-33.9	98.4	-40.7
	P→Au	-62.9	-75.3	138.6	-77.7	216.2	-107.2
	π(C=P)→Au	-59.5	-77.8	154.6	-83.1	237.7	-110.4
Mes*	Sn(II)→Au	-55.9	-48.4	87.6	-55.7	143.3	-78.4
	O→Au	-36.7	-49.9	70.8	-37.5	108.3	-43.3
	P→Au	-60.8	-76.0	142.8	-80.2	222.9	-104.6
	π(C=P)→Au	-55.7	-88.0	177.4	-95.6	273.0	-110.9
R	coord.mode	PCPS-Sn(II)-AuCl					
Me	Sn(II)→Au	-50.8	-42.3	81.3	-51.5	132.8	-74.8
	S→Au	-44.0	-52.6	97.6	-54.0	151.6	-72.3
	P→Au	-61.9	-74.6	139.0	-77.8	216.8	-107.1
	π(C=P)→Au	-58.3	-76.8	155.3	-83.1	238.4	-110.3
Mes*	Sn(II)→Au	-56.0	-47.8	87.0	-55.2	142.2	-78.0
	S→Au	-50.3	-58.8	102.8	-57.3	160.1	-76.3
	P→Au	-60.9	-75.9	143.1	-80.4	223.5	-104.6
	π(C=P)→Au	-54.2	-87.6	178.7	-96.2	275.0	-110.3

Table S21. Calculated EDA parameters (kcal mol^{-1}) for several model {RP=C(Pb(II)-Cl)-P(X)(Me)R}AuCl model complexes (R = Me or Mes*; X = O or S).

R	coord.mode	Total int. energy	Electrostatic Int.	Exchange-Rep.	Exchange Int.	Repulsion	Orbital Relaxation
				PCPO-Pb(II)-AuCl			
Me	Pb(II)→Au	-33.7	-24.1	58.7	-36.6	95.4	-55.8
	O→Au	-34.8	-47.6	66.0	-34.7	100.7	-41.9
	P→Au	-63.3	-75.5	138.3	-77.6	215.9	-107.1
	π(C=P)→Au	-60.2	-78.9	154.8	-83.5	238.2	-110.2
Mes*	Pb(II)→Au	-38.1	-27.7	62.3	-39.0	101.3	-58.7
	O→Au	-38.6	-52.0	72.6	-38.5	111.0	-44.9
	P→Au	-61.0	-75.8	142.2	-79.9	222.1	-104.4
	π(C=P)→Au	-57.0	-89.0	177.0	-95.6	272.6	-111.0
R	coord.mode			PCPS-Pb(II)-AuCl			
Me	Pb(II)→Au	-33.8	-23.6	58.0	-36.1	94.2	-55.7
	S→Au	-45.0	-53.7	98.4	-54.5	152.8	-72.8
	P→Au	-62.2	-74.7	138.7	-77.7	216.3	-106.9
	π(C=P)→Au	-59.1	-78.0	155.6	-83.5	239.1	-110.3
Mes*	Pb(II)→Au	-38.1	-27.5	61.9	-38.7	100.6	-58.0
	S→Au	-51.4	-59.6	103.2	-57.7	160.8	-76.8
	P→Au	-61.1	-75.7	142.3	-80.1	222.4	-104.4
	π(C=P)→Au	-55.5	-88.1	177.5	-95.6	273.1	-110.3

xyz coordinates

PCPX model systems 24

R-P=C(Cl)-P(O)ClR 24

R= H 24

R= Me 24

R = *t*-Bu 24

R = Ph 24

R = Mes 24

R = Mes* 25

R-P=C(Cl)-P(O)MeR 26

R = H 26

R = Me 26

R = *t*-Bu 26

R = Ph 26

R = Mes 26

R = Mes* 27

R-P=C(Cl)-P(S)ClR 28

R= H 28

R= Me 28

R = *t*-Bu 28

R = Ph 28

R = Mes 28

R = Mes* 29

R-P=C(Cl)-P(S)MeR 29

R = H 29

R = Me 30

R = *t*-Bu 30

R = Ph 30

R = Mes 30

R = Mes* 31

PCPX-E(II) model systems 31

E = Si(II) 31

R-P=C(Si(II)Cl)-P(O)ClR 31

R= H 31

R = Me 32

R = *t*-Bu ....

R = Ph ....

R = Mes ....

R = Mes* ....

R-P=C(Si(II)Cl)-P(O)MeR34

R= H ....

R = Me ....

R = *t*-Bu ....

R = Ph ....

R = Mes ....

R = Mes* ....

R-P=C(Si(II)Cl)-P(S)ClR 36

R= H ....

R = Me ....

R = *t*-Bu ....

R = Ph ....

R = Mes* ....

R-P=C(Si(II)Cl)-P(S)MeR 37

R = H ....

R = Me ....

R = *t*-Bu ....

R = Ph ....

R = Mes ....

R = Mes* ....

E = Ge(II) ....

R-P=C(Ge(II)Cl)-P(O)ClR 39

R= H ....

R = Me ....

R = *t*-Bu ....

R = Ph ....

R = Mes ....

R = Mes* ....

R-P=C(Ge(II)Cl)-P(O)MeR 42

R= H ....

R = Me ....

R = *t*-Bu ....

R = Ph ....

R = Mes ....

R = Mes* ....

R-P=C(Ge(II)Cl)-P(S)ClR 44
R= H 44
R = Me 44
R = *t*-Bu 44
R = Ph 44
R = Mes 44
R = Mes* 45
R-P=C(Ge(II)Cl)-P(S)MeR 45
R= H 45
R = Me 46
R = *t*-Bu 46
R = Ph 46
R = Mes 46
R = Mes* 47
E = Sn (II) 48
R-P=C(Sn(II)Cl)-P(O)ClR 48
R= H 48
R = Me 48
R = *t*-Bu 48
R = Ph 48
R = Mes 48
R = Mes* 49
R-P=C(Sn(II)Cl)-P(O)MeR 50
R= H 50
R = Me 50
R = *t*-Bu 50
R = Ph 50
R = Mes 51
R = Mes* 51
R-P=C(Sn(II)Cl)-P(S)ClR 52
R= H 52
R = Me 52
R =*t*-Bu 52
R = Ph 52
R = Mes 53
R = Mes* 53
R-P=C(Sn(II)Cl)-P(S)MeR 54
R= H 54
R =Me 54
R = *t*-Bu 54

R = Ph ....
R = Mes ....
R = Mes* ....
R-P=C(Pb (II)Cl)-P(O)ClR56
R= H ....
R = Me ....
R = *t*-Bu ....
R = Ph ....
R = Mes ....
R = Mes* ....
R-P=C(Pb (II)Cl)-P(O)MeR 58
R= H ....
R = Me ....
R = *t*-Bu ....
R = Ph ....
R = Mes ....
R = Mes* ....
R-P=C(Pb (II)Cl)-P(S)ClR 60
R= H ....
R = Me ....
R = *t*-Bu ....
R = Ph ....
R = Mes ....
R = Mes* ....
R-P=C(Pb (II)Cl)-P(S)MeR 62
R= H ....
R = Me ....
R = *t*-Bu ....
R = Ph ....
R = Mes ....
R = Mes* ....
PCPX-E(II)-AuCl model systems 64
E = Si(II) ....
[R-P=C(Si(II)Cl)-P(O)ClR](AuCl) 64
Coordination through Si(II) atom ....
R= H ....
R = Me ....
R = *t*-Bu ....
R = Ph ....
R = Mes ....

R = Mes *
Coordination through O atom
R= H
R = Me
R = *t*-Bu
R = Ph
R = Mes
R = Mes*
Coordination through P (sp^2) atom
R = H
R = Me
R = *t*-Bu
R= Ph
R = Mes
R = Mes*
Coordination through π(C=P) bond
R = H
R = Me
R = *t*-Bu
R = Ph
R = Mes
R = Mes*
[R-P=C(Si(II)Cl)-P(O)MeR](AuCl) 72
Coordination through Si(II) atom
R= H
R = Me
R = *t*-Bu
R = Ph
R = Mes
R = Mes*
Coordination through O atom 74
R = H
R = Me
R = *t*-Bu
R = Ph
R = Mes
R = Mes*
Coordination through P(sp^2) atom
R = H
R = Me
R = *t*-Bu 65
R = Ph 66
R = Mes 66
R = Mes* 66
Coordination through π(C=P) bond 67
R = H 67
R = Me 67
R = *t*-Bu 67
R = Ph 68
R = Mes 68
R = Mes* 68
[R-P=C(Si(II)Cl)-P(S)ClR](AuCl) 81 68
Coordination through Si(II) atom 69
R= H 69
R = Me 69
R = *t*-Bu 70
R = Ph 70
R = Mes 70
R = Mes* 70
Coordination through S atom 71
R= H 71
R = Me 71
R = *t*-Bu
R = Ph 72
R = Mes 72
R = Mes* 72
Coordination through P(sp^2) atom 72
R= H 73
R = Me 73
R = *t*-Bu 73
R = Ph
R = Mes 74
R = Mes* 74
Coordination through π(C=P) bond 75
R= H 75
R = Me 75
R = *t*-Bu 76
R = Ph 76
R = Mes 76
R = Mes* 77

[R-P=C(Si(II)Cl)-P(S)MeR](AuCl) 89
Coordination through Si(II) atom ... 89
R= H ... 89
R = Me ... 89
R = *t*-Bu ... 89
R = Ph ... 90
R = Mes ... 90
R = Mes* ... 90
Coordination through S atom ... 91
R= H ... 91
R = Me ... 91
R = *t*-Bu ... 91
R = Ph ... 92
R = Mes ... 92
R = Mes* ... 92
Coordination through P(sp^2) atom ... 93
R= H ... 93
R = Me ... 93
R = *t*-Bu ... 93
R = Ph ... 94
R = Mes ... 94
R = Mes* ... 94
Coordination through π(C=P) bond ... 96
R= H ... 96
R = Me ... 96
R = *t*-Bu ... 96
R = Ph ... 96
R = Mes ... 96
R = Mes* ... 97
PCPX-E(II)-AuCl model systems 98
E = Ge(II) ... 98
[R-P=C(Ge(II)Cl)-P(O)ClR](AuCl) 98
Coordination through Ge(II) atom ... 98
R= H ... 98
R = Me ... 98
R = *t*-Bu ... 98
R = Ph ... 98
R = Mes ... 98
R = Mes* ... 99
Coordination through O atom ... 100
R= H ...
R = Me ...
R = *t*-Bu ...
R = Ph ...
R = Mes ...
R = Mes* ...
Coordination through P(sp^2) atom ...
R= H ...
R = Me ...
R = *t*-Bu ...
R = Ph ...
R = Mes ...
R = Mes* ...
Coordination through π(C=P) bond ...
R= H ...
R = Me ...
R = *t*-Bu ...
R = Ph ...
R = Mes ...
R = Mes* ...
[R-P=C(Ge(II)Cl)-P(O)MeR](AuCl) 106
Coordination through Ge(II) atom ...
R= H ...
R = Me ...
R = *t*-Bu ...
R = Ph ...
R = Mes ...
R = Mes* ...
Coordination through O atom ...
R= H ...
R = Me ...
R = *t*-Bu ...
R = Ph ...
R = Mes ...
R = Mes* ...
Coordination through P(sp^2) atom ...
R= H ...
R = Me ...
R = *t*-Bu ...
R = Ph ...

R = Mes 111
R = Mes* 111
Coordination through π(C=P) bond 112
R= H 112
R = Me 112
R = *t*-Bu 112
R = Ph 113
R = Mes 113
R = Mes* 113
[R-P=C(Ge(II)Cl)-P(S)ClR](AuCl) 114
Coordination through Ge(II) atom 114
R= H 114
R = Me 114
R = *t*-Bu 114
R = Ph 115
R = Mes 115
R = Mes* 115
Coordination through S atom 116
R= H 116
R = Me 116
R = *t*-Bu 116
R = Ph 117
R = Mes 117
R = Mes* 117
Coordination through P(sp^2) atom 118
R= H 118
R = Me 118
R = *t*-Bu 118
R = Ph 119
R = Mes 119
R = Mes* 119
Coordination through π(C=P) bond 120
R= H 120
R = Me 120
R = *t*-Bu 120
R = Ph 121
R = Mes 121
R = Mes* 121
[R-P=C(Ge(II)Cl)-P(S)MeR](AuCl) 122
Coordination through Ge(II) atom 122

R= H
R = Me
R = *t*-Bu
R = Ph
R = Mes
R = Mes*
Coordination through S atom
R= H
R = Me
R = *t*-Bu .....
R = Ph
R = Mes
R = Mes*
Coordination through P(sp^2) atom
R= H
R = Me
R = *t*-Bu
R = Ph
R = Mes
R = Mes*
Coordination through π(C=P) bond
R= H
R = Me
R = *t*-Bu
R = Ph
R = Mes
R = Mes*
E = Sn(II)
[R-P=C(Sn(II)Cl)-P(O)ClR](AuCl) 131
Coordination through Sn(II) atom
R= H
R = Me
R = *t*-Bu
R = Ph
R = Mes
R = Mes*
Coordination through O atom
R= H
R = Me .....
R = *t*-Bu

R = Ph 133
R = Mes 133
R = Mes* 134
Coordination through P(sp^2) atom 135
R= H 135
R = Me 135
R = *t*-Bu 135
R = Ph 135
R = Mes 135
R = Mes* 136
Coordination through π(C=P) bond 137
R= H 137
R = Me 137
R = *t*-Bu 137
R = Ph 137
R = Mes 137
R = Mes* 138
[R-P=C(Sn(II)Cl)-P(O)MeR](AuCl) 139
Coordination through Sn(II) atom 139
R= H 139
R = Me 139
R = *t*-Bu 139
R = Ph 139
R = Mes 139
R = Mes* 140
Coordination through O atom 141
R= H 141
R = Me 141
R = *t*-Bu 141
R = Ph 141
R = Mes 142
R = Mes* 142
Coordination through P(sp^2) atom 143
R= H 143
R = Me 143
R = *t*-Bu 143
R = Ph 143
R = Mes 144
R = Mes* 144
Coordination through π(C=P) bond 145

R= H
R = Me
R = *t*-Bu
R = Ph
R = Mes
R = Mes*
[R-P=C(Sn(II)Cl)-P(S)ClR](AuCl) 147
Coordination through Sn(II) atom
R= H
R = Me
R = *t*-Bu
R = Ph
R = Mes
R = Mes*
Coordination through S atom
R= H
R = Me
R = *t*-Bu
R = Ph
R = Mes
R = Mes*
Coordination through P(sp^2) atom
R= H
R = Me
R = *t*-Bu
R = Ph
R = Mes
R = Mes*
Coordination through π(C=P) bond
R= H
R = Me
R = *t*-Bu
R = Ph
R = Mes
R = Mes*
[R-P=C(Sn(II)Cl)-P(S)MeR](AuCl) 155
Coordination through Sn(II) atom
R= H
R = Me
R = *t*-Bu

R = Ph R= H 156

R = Mes R = Me 156

R = Mes* R = *t*-Bu 156

Coordination through S atom R = Ph 157

R= H R = Mes 157

R = Me R = Mes* 157

R = *t*-Bu Coordination through π(C=P) bond 157

R = Ph R= H 158

R = Mes R = Me 158

R = Mes* R = *t*-Bu 158

Coordination through P(sp^2) atom R = Ph 159

R= H R = Mes 159

R = Me R = Mes* 159

R = *t*-Bu [R-P=C(Pb(II)Cl)-P(O)MeR](AuCl) 172 160

R = Ph Coordination through Pb(II) atom 160

R = Mes R= H 160

R = Mes* R = Me 161

Coordination through π(C=P) bond R = *t*-Bu 161

R= H R = Ph 161

R = Me R = Mes 161

R = *t*-Bu R = Mes* 162

R = Ph Coordination through O atom 162

R = Mes R= H 162

R = Mes* R = Me 163

[R-P=C(Pb(II)Cl)-P(O)ClR](AuCl) 164 R = *t*-Bu

Coordination through Pb(II) atom R = Ph 164

R= H R = Mes 164

R = Me R = Mes* 164

R = *t*-Bu Coordination through P(sp^2) atom 164

R = Ph R= H 164

R = Mes R = Me 164

R = Mes* R = *t*-Bu 165

Coordination through O atom R = Ph 166

R= H R = Mes 166

R = Me R = Mes* 166

R = *t*-Bu Coordination through π(C=P) bond 166

R = Ph R= H 166

R = Mes R = Me 166

R = Mes* R = *t*-Bu 167

Coordination through P(sp^2) atom R = Ph 168

R = Mes

R = Mes*

[R-P=C(Pb(II)Cl)-P(S)ClR](AuCl) 180

Coordination through Pb(II) atom

R= H

R = Me

R = *t*-Bu

R = Ph

R = Mes

R = Mes*

Coordination through S atom

R= H

R = Me

R = *t*-Bu

R = Ph

R = Mes

R = Mes*

Coordination through P(sp^2) atom

R= H

R = Me

R = *t*-Bu

R = Ph

R = Mes

R = Mes*

Coordination through π(C=P) bond

R= H

R = Me

R = *t*-Bu

R = Ph

R = Mes

R = Mes* 179

[R-P=C(Pb(II)Cl)-P(S)MeR](AuCl) 188 179

Coordination through Pb(II) atom

R= H 180

R = Me 180

R = *t*-Bu 180

R = Ph 180

R = Mes 181

R = Mes* 181

Coordination through S atom 181

R= H 182

R = Me 182

R = *t*-Bu 182

R = Ph 182

R = Mes 183

R = Mes* 183

Coordination through P(sp^2) atom 183

R= H 184

R = Me 184

R = *t*-Bu 184

R = Ph 184

R = Mes 185

R = Mes* 185

Coordination through π(C=P) bond 185

R= H 186

R = Me 186

R = *t*-Bu 186

R = Ph 186

R = Mes 187

R = Mes* 187

PCPX model systems

R-P=C(Cl)-P(O)ClR

R= H

P	1.79405	0.09029	-2.25367
C	0.31215	-0.02845	-1.48610
P	-1.04264	-0.75902	-2.42456
Cl	-0.09833	0.41754	0.11870
O	-0.65622	-1.31089	-3.72351
Cl	-2.43636	0.70450	-2.48890
H	2.46714	0.68669	-1.14569
H	-1.65784	-1.63561	-1.50952

R= Me

P	1.77770	0.04145	-2.18154
C	0.27617	-0.02102	-1.44367
P	-1.05425	-0.77986	-2.39304
Cl	-0.12714	0.54600	0.13283
O	-0.62108	-1.23927	-3.71641
C	-1.82992	-2.02023	-1.35494
H	-2.11693	-1.60938	-0.38776
H	-2.71261	-2.38967	-1.87876
H	-1.11927	-2.83833	-1.22257
C	2.81082	0.86042	-0.90554
H	2.82660	0.27547	0.01723
H	3.82783	0.95642	-1.28691
H	2.41973	1.85301	-0.66912
Cl	-2.46933	0.68703	-2.45700

R = *t*-Bu

P	1.16488	-1.27309	-0.56412
C	0.59535	-0.01290	-1.51112
P	-0.98752	-0.39204	-2.31823
Cl	1.27291	1.54587	-1.81101
O	-1.60410	-1.60355	-1.75911
C	2.79754	-0.77278	0.21508
C	-0.81468	-0.46707	-4.15116
C	3.19493	-2.00439	1.03587
H	3.31943	-2.88879	0.40514
H	4.14619	-1.81511	1.54263
H	2.44823	-2.24089	1.79862
C	3.87046	-0.51008	-0.83869
H	3.65473	0.37387	-1.43757
H	4.83353	-0.35381	-0.34007
H	3.98609	-1.36313	-1.51283
C	2.62332	0.41701	1.15621
H	2.36716	1.33242	0.62442
H	1.84771	0.22612	1.90252
H	3.56204	0.58953	1.69430
C	-0.27165	0.80300	-4.79466
H	0.75657	1.01001	-4.49737
H	-0.28498	0.67291	-5.88120
H	-0.88243	1.67454	-4.55408
C	-2.21666	-0.77153	-4.68685
H	-2.90149	0.06186	-4.51864
H	-2.14805	-0.93995	-5.76554
H	-2.63603	-1.66432	-4.22120
C	0.11373	-1.65142	-4.43525
H	1.12855	-1.46710	-4.07566
H	-0.25676	-2.56832	-3.97363
H	0.16420	-1.80646	-5.51687
Cl	-2.08643	1.29560	-1.97265

R = Ph

P	1.06821	-1.05973	-0.36084
C	0.80585	-0.30830	-1.83838
P	-0.79193	-0.69339	-2.59101
Cl	1.81880	0.76739	-2.71673
O	-1.60898	-1.57138	-1.74487
C	-0.50386	-1.33437	-4.24480
C	-0.66944	-2.71034	-4.39650
C	-0.12116	-0.55349	-5.33284
C	-0.44557	-3.29986	-5.63062
H	-0.98924	-3.30230	-3.54680
C	0.10256	-1.15043	-6.56237
H	-0.01113	0.51805	-5.22186
C	-0.05710	-2.52159	-6.71104
H	-0.58124	-4.36860	-5.74910
H	0.39621	-0.54213	-7.40995
H	0.11509	-2.98362	-7.67663
C	2.73669	-0.57428	0.15890
C	3.87674	-0.64990	-0.64379
C	2.87740	-0.22199	1.50333
C	5.12195	-0.35347	-0.11580
H	3.79209	-0.95008	-1.68073
C	4.12177	0.09849	2.02221
H	2.00306	-0.19423	2.14650
C	5.24580	0.03155	1.21269
H	6.00202	-0.42130	-0.74521
H	4.21466	0.38806	3.06257
H	6.22225	0.26896	1.61931
Cl	-1.59855	1.14800	-2.90187

R = Mes

P	1.64974	-1.72979	-1.07216
C	0.86245	-0.53173	-1.94196
P	-0.77366	-0.88482	-2.64264
Cl	1.31459	1.11891	-2.06143
O	-1.82859	-0.17986	-1.90401
C	3.05446	-0.86528	-0.30122
C	4.32397	-0.96292	-0.88336
C	2.88031	-0.19781	0.92006
C	5.40517	-0.37731	-0.23579
C	3.98942	0.37033	1.53150
C	5.26004	0.29134	0.97234
H	6.38887	-0.44623	-0.69122
H	3.85592	0.89113	2.47542
C	-0.94608	-2.67638	-2.79894
C	-1.93266	-3.32061	-2.01589
C	-0.13058	-3.43693	-3.66485
C	-2.07935	-4.69736	-2.13987
C	-0.32644	-4.80960	-3.73628
C	-1.29906	-5.46089	-2.99388
H	-2.83365	-5.18876	-1.53340
H	0.31255	-5.38820	-4.39654
C	4.52321	-1.65336	-2.19950
H	4.03723	-2.63440	-2.21970
H	4.09676	-1.06788	-3.01972
H	5.58361	-1.79967	-2.40928
C	1.52881	-0.07976	1.55787
H	0.85442	0.52986	0.94985

H	1.05338	-1.05824	1.67895
H	1.60241	0.38035	2.54393
C	6.44284	0.88843	1.67169
H	6.81097	0.21819	2.45480
H	7.26636	1.06824	0.97884
H	6.18501	1.83527	2.15057
C	-1.51003	-6.93705	-3.12115
H	-1.89035	-7.36671	-2.19288
H	-2.24186	-7.15359	-3.90580
H	-0.58461	-7.45080	-3.38716
C	0.94942	-2.87451	-4.54502
H	0.53495	-2.53299	-5.49642
H	1.47447	-2.03081	-4.10149
H	1.68311	-3.65256	-4.76165
C	-2.83943	-2.63211	-1.03583
H	-2.28245	-2.06416	-0.29014
H	-3.49693	-1.91451	-1.52602
H	-3.44833	-3.37782	-0.52338
Cl	-0.66768	-0.15822	-4.54839

R = Mes*

P	2.29541	-1.92892	-0.69160
C	1.33217	-1.01845	-1.71583
P	-0.25305	-1.61620	-2.42336
Cl	1.51248	0.67005	-2.00997
O	-1.34611	-0.90380	-1.74264
C	3.51219	-0.73546	0.01625
C	4.76688	-0.52942	-0.60954
C	3.19276	-0.01400	1.20091
C	5.57995	0.49855	-0.13356
C	4.06079	0.99263	1.60229
C	5.23782	1.30085	0.93774
H	6.52224	0.67651	-0.62489
H	3.81316	1.56505	2.48338
C	-0.65204	-3.39253	-2.66553
C	-1.78556	-3.83160	-1.91852
C	-0.32440	-4.09761	-3.86406
C	-2.70574	-4.65972	-2.55825
C	-1.28704	-4.93192	-4.41109
C	-2.52806	-5.15729	-3.83409
H	-3.60576	-4.91647	-2.01980
H	-1.05363	-5.43065	-5.34273
C	5.32016	-1.35948	-1.78740
C	1.96817	-0.27992	2.10358
C	6.11043	2.45357	1.42477
C	-3.58224	-5.97800	-4.56608
C	1.04292	-4.18044	-4.57807
C	-2.09136	-3.59526	-0.42158
C	4.64884	-0.93789	-3.09783
H	3.57245	-1.09057	-3.07806
H	4.83188	0.12077	-3.29516
H	5.05788	-1.51586	-3.93180
C	6.82817	-1.15183	-1.98188
H	7.07377	-0.14459	-2.32425
H	7.39370	-1.35895	-1.07014
H	7.17548	-1.84189	-2.75428
C	5.15036	-2.86208	-1.54237
H	5.63999	-3.16117	-0.61227
H	4.10762	-3.18283	-1.48465
C	0.68821	0.29822	1.49011
H	0.79277	1.37485	1.33798
H	0.43235	-0.14638	0.53142
H	-0.15408	0.13263	2.16797
C	2.12029	0.37891	3.48147
H	3.04437	0.08035	3.98265
H	2.08450	1.46925	3.43202
H	1.28323	0.06384	4.10850
C	1.81492	-1.77690	2.39028
H	1.60078	-2.37378	1.50074
H	2.72180	-2.17834	2.84971
H	0.98064	-1.93706	3.07821
C	7.35509	2.64475	0.56220
H	7.93333	3.49161	0.93967
H	8.00582	1.76688	0.58423
H	7.09868	2.85744	-0.47872
C	5.29574	3.75197	1.38479
H	4.96573	3.97463	0.36721
H	4.40845	3.69284	2.01818
H	5.90370	4.58978	1.73773
C	6.56124	2.18276	2.86508
H	7.18309	3.00658	3.22676
H	5.71183	2.08346	3.54382
H	7.14740	1.26232	2.92419
C	-3.34172	-2.73154	-0.23129
H	-4.21182	-3.17626	-0.72055
H	-3.56888	-2.64649	0.83557
H	-3.18306	-1.73428	-0.63874
C	-2.34624	-4.98019	0.21191
H	-2.48922	-4.85203	1.28786
H	-3.23522	-5.47777	-0.17495
H	-1.49244	-5.64591	0.06145
C	-0.93487	-3.00877	0.37833
H	-0.01132	-3.56566	0.20067
H	-0.77213	-1.95773	0.16152
H	-1.16605	-3.08771	1.44372
C	0.95085	-3.80867	-6.06203
H	1.92930	-3.94546	-6.53086
H	0.23895	-4.43412	-6.60317
H	0.65462	-2.76816	-6.19299
C	1.49158	-5.65219	-4.46782
H	2.47809	-5.76428	-4.92635
H	1.56614	-5.95847	-3.42152
H	0.80886	-6.33789	-4.97038
C	2.15580	-3.36819	-3.94150
H	2.26006	-3.60634	-2.88229
H	3.10107	-3.61589	-4.43106
H	2.01239	-2.29620	-4.04995
C	-3.89338	-5.30176	-5.90677
H	-3.00466	-5.22386	-6.53621
H	-4.64311	-5.87972	-6.45424
H	-4.28414	-4.29297	-5.75414
C	-3.04827	-7.39225	-4.82259
H	-2.14468	-7.38080	-5.43542
H	-2.81121	-7.89737	-3.88303
H	-3.79977	-7.98716	-5.34913
C	-4.88123	-6.09071	-3.77276
H	-4.73825	-6.62198	-2.82838
H	-5.30878	-5.10895	-3.55484
H	-5.61643	-6.65110	-4.35525
Cl	-0.15977	-0.93409	-4.35504
H	5.60303	-3.42295	-2.36445

R-P=C(Cl)-P(O)MeR

R = H

P	1.81869	0.09240	-2.16926
C	0.30915	-0.04938	-1.46467
P	-1.04359	-0.70280	-2.48218
Cl	-0.13776	0.33233	0.15530
O	-0.60076	-1.16767	-3.80972
H	2.46307	0.61837	-1.00869
H	-1.57582	-1.69304	-1.61876
C	-2.31618	0.56978	-2.48485
H	-3.19857	0.16483	-2.98378
H	-2.57549	0.87559	-1.47051
H	-1.95047	1.42776	-3.05119

R = Me

P	1.77521	0.03941	-2.15795
C	0.26259	-0.00627	-1.44631
P	-1.09488	-0.72326	-2.41541
Cl	-0.14433	0.56673	0.13637
O	-0.63639	-1.17748	-3.74482
C	-1.77001	-2.03378	-1.37427
H	-2.03890	-1.67148	-0.38137
H	-2.65474	-2.44081	-1.86810
H	-1.02240	-2.82345	-1.28755
C	2.81069	0.82309	-0.85696
H	2.80446	0.22696	0.05870
H	3.83437	0.90225	-1.22455
H	2.44031	1.82142	-0.61202
C	-2.36174	0.56123	-2.46497
H	-1.98359	1.39446	-3.05890
H	-3.24510	0.15087	-2.95850
H	-2.62932	0.91225	-1.46767

R = *t*-Bu

P	1.13042	-1.26021	-0.55769
C	0.57279	-0.00127	-1.50930
P	-1.04478	-0.31754	-2.29831
Cl	1.28064	1.55518	-1.80953
O	-1.65905	-1.54843	-1.74796
C	-2.00651	1.17735	-1.97136
H	-1.51650	2.08521	-2.32096
H	-2.98153	1.07377	-2.44986
H	-2.15570	1.23748	-0.89199
C	2.78438	-0.78688	0.20026
C	-0.80323	-0.45033	-4.12944
C	3.17283	-2.02362	1.01718
H	3.27229	-2.91061	0.38561
H	4.13445	-1.85127	1.51071
H	2.43227	-2.24671	1.78996
C	3.84887	-0.54367	-0.86642
H	3.64168	0.34393	-1.46304
H	4.82189	-0.40522	-0.38151
H	3.93875	-1.39843	-1.54228
C	2.64667	0.40597	1.14327
H	2.39868	1.32538	0.61417
H	1.87853	0.22876	1.90079
H	3.59577	0.56329	1.66809
C	-0.30585	0.82393	-4.80414
H	0.69162	1.10918	-4.46910
H	-0.25768	0.65524	-5.88488
H	-0.97832	1.66856	-4.63726
C	-2.17771	-0.82878	-4.69239
H	-2.90307	-0.01862	-4.58182
H	-2.07823	-1.03538	-5.76244
H	-2.57555	-1.71732	-4.20046
C	0.17365	-1.59958	-4.37638
H	1.18152	-1.35285	-4.03551
H	-0.14954	-2.50810	-3.86416
H	0.22239	-1.80838	-5.44947

R = Ph

P	1.05272	-1.10621	-0.37323
C	0.75340	-0.29653	-1.81014
P	-0.85670	-0.65742	-2.57778
Cl	1.74257	0.85345	-2.63450
O	-1.64248	-1.59577	-1.74729
C	-1.63165	0.95062	-2.82682
H	-0.99828	1.63976	-3.38508
H	-2.56876	0.79367	-3.36364
H	-1.84789	1.37219	-1.84381
C	-0.49131	-1.32374	-4.21953
C	-0.68999	-2.69324	-4.38325
C	-0.04924	-0.55944	-5.29783
C	-0.44197	-3.29134	-5.60891
H	-1.05385	-3.27171	-3.54144
C	0.19675	-1.16111	-6.52198
H	0.10736	0.50709	-5.18878
C	0.00276	-2.52660	-6.67753
H	-0.60129	-4.35663	-5.73102
H	0.53878	-0.56177	-7.35802
H	0.19449	-2.99419	-7.63681
C	2.72079	-0.59776	0.13933
C	3.84958	-0.66404	-0.67944
C	2.87940	-0.24058	1.47981
C	5.10050	-0.35462	-0.17196
H	3.75080	-0.96656	-1.71490
C	4.12903	0.09081	1.97975
H	2.01475	-0.21867	2.13618
C	5.24169	0.03284	1.15394
H	5.97115	-0.41489	-0.81524
H	4.23507	0.38242	3.01841
H	6.22207	0.27921	1.54561

R = Mes

P	1.61470	-1.72697	-1.06597
C	0.84404	-0.53944	-1.96016
P	-0.77665	-0.85553	-2.74245
Cl	1.33187	1.10948	-2.10002
O	-1.86427	-0.18032	-1.99892
C	-0.65692	-0.16442	-4.41140
H	0.32186	-0.26021	-4.87482
H	-1.41492	-0.63484	-5.04030
H	-0.90310	0.89229	-4.29825
C	3.02176	-0.86803	-0.28814
C	4.30050	-0.99690	-0.84366
C	2.83873	-0.17220	0.91629
C	5.37981	-0.41420	-0.18998
C	3.94579	0.39225	1.53527
C	5.22457	0.28244	1.00082
H	6.37018	-0.50838	-0.62621
H	3.80390	0.93467	2.46578
C	-0.92460	-2.66936	-2.83615
C	-1.83675	-3.32234	-1.97740
C	-0.17214	-3.43365	-3.75170
C	-1.99291	-4.70008	-2.09284
C	-0.36814	-4.80673	-3.81733
C	-1.28440	-5.46134	-3.00838
H	-2.69381	-5.19405	-1.42685
H	0.22629	-5.38320	-4.52008
C	4.51431	-1.72000	-2.14010

H	4.01945	-2.69657	-2.14505
H	4.10592	-1.15034	-2.98043
H	5.57671	-1.87997	-2.32929
C	1.47956	-0.02207	1.53091
H	0.81895	0.57449	0.89569
H	0.99304	-0.99206	1.67353
H	1.54443	0.46632	2.50399
C	6.40432	0.87630	1.70836
H	6.74564	0.22004	2.51513
H	7.24407	1.02436	1.02748
H	6.15442	1.83939	2.15833
C	-1.50486	-6.93747	-3.12505
H	-1.82832	-7.36801	-2.17581
H	-2.28262	-7.15391	-3.86431
H	-0.59746	-7.45220	-3.44637
C	0.86717	-2.86069	-4.67455
H	0.41490	-2.41812	-5.56550
H	1.47847	-2.09709	-4.19210
H	1.53563	-3.65365	-5.01227
C	-2.64776	-2.63783	-0.91402
H	-2.02208	-2.05719	-0.23538
H	-3.35740	-1.92830	-1.33842
H	-3.19053	-3.38631	-0.33474

R = Mes*

P	2.28599	-1.92738	-0.66353
C	1.34620	-1.04792	-1.73311
P	-0.28626	-1.58908	-2.41086
Cl	1.60612	0.62133	-2.12121
O	-1.33400	-0.94877	-1.57591
C	-0.38904	-0.87627	-4.07369
H	0.48893	-1.00495	-4.70052
H	-1.25904	-1.32113	-4.55835
H	-0.57276	0.18728	-3.92301
C	3.52016	-0.73516	0.02285
C	4.80780	-0.58965	-0.55439
C	3.17289	0.04292	1.16440
C	5.63059	0.43181	-0.08062
C	4.04930	1.04577	1.55821
C	5.26351	1.29094	0.93705
H	6.60072	0.56205	-0.52984
H	3.77931	1.66448	2.40042
C	-0.65624	-3.38640	-2.67354
C	-1.74717	-3.89236	-1.90859
C	-0.37132	-4.02855	-3.91738
C	-2.67317	-4.71744	-2.54778
C	-1.33347	-4.86222	-4.46911
C	-2.54052	-5.15286	-3.85103
H	-3.54319	-5.02113	-1.98505
H	-1.12753	-5.30902	-5.43349
C	5.38736	-1.48801	-1.67240
C	1.91569	-0.16341	2.04099
C	6.14697	2.43978	1.41420
C	-3.60213	-5.97375	-4.57311
C	0.96394	-4.03007	-4.69497
C	-2.00862	-3.72293	-0.39400
C	4.70943	-1.15653	-3.00497
H	3.64028	-1.35094	-2.98144
H	4.85070	-0.09942	-3.24078
H	5.14509	-1.74119	-3.81897
C	6.88623	-1.24899	-1.88254
H	7.09516	-0.23961	-2.24191
H	7.46709	-1.42385	-0.97324
H	7.25193	-1.93106	-2.65076
C	5.21295	-2.97052	-1.27443
H	5.66723	-3.10644	-0.28677
H	4.14808	-3.18858	-1.13772
C	0.65257	0.38570	1.36787
H	0.77657	1.44607	1.13590
H	0.39003	-0.12761	0.44617
H	-0.19729	0.28770	2.04948
C	2.03298	0.56931	3.38512
H	2.93858	0.29263	3.93072
H	2.00714	1.65571	3.27516
H	1.17460	0.29397	4.00188
C	1.74707	-1.64191	2.40764
H	1.56223	-2.28794	1.54690
H	2.63630	-2.01658	2.92131
H	0.88906	-1.75980	3.07470
C	7.43617	2.55680	0.60543
H	8.02114	3.40361	0.97267
H	8.05844	1.66300	0.69779
H	7.23568	2.72847	-0.45517
C	5.37453	3.75730	1.27759
H	5.10031	3.94259	0.23619
H	4.45648	3.75097	1.86843
H	5.98950	4.59324	1.62296
C	6.52152	2.22315	2.88522
H	7.15065	3.04474	3.23950
H	5.63855	2.17913	3.52586
H	7.07575	1.29005	3.01346
C	-3.25979	-2.87866	-0.13263
H	-4.13960	-3.31077	-0.61655
H	-3.45806	-2.84086	0.94285
H	-3.11640	-1.86418	-0.50147
C	-2.23351	-5.13242	0.19444
H	-2.32896	-5.04642	1.27984
H	-3.13860	-5.61829	-0.17016
H	-1.38612	-5.78926	-0.01866
C	-0.83268	-3.15578	0.39033
H	0.08482	-3.71168	0.18150
H	-0.67522	-2.10195	0.18242
H	-1.04075	-3.25299	1.45924
C	0.79552	-3.55135	-6.14166
H	1.75352	-3.61907	-6.66472
H	0.07702	-4.15965	-6.69351
H	0.45841	-2.51548	-6.19363
C	5.76464	-4.01631	-2.23215
H	5.49068	-5.01510	-1.88418
H	5.35958	-3.90389	-3.24041
H	6.85365	-3.98917	-2.30198
C	1.45201	-5.49283	-4.72466
H	2.42180	-5.54233	-5.22797
H	1.57470	-5.87992	-3.71043
H	0.76631	-6.15372	-5.25523
C	2.09092	-3.25008	-4.04172
H	2.25717	-3.57786	-3.01468
H	3.01304	-3.42196	-4.60243
H	1.92872	-2.17525	-4.02828
C	-3.99725	-5.24990	-5.86579
H	-3.14103	-5.11186	-6.52930
H	-4.75114	-5.82848	-6.40702
H	-4.41551	-4.26401	-5.64878
C	-3.03810	-7.35682	-4.91932
H	-2.16478	-7.28784	-5.57112
H	-2.74137	-7.89507	-4.01586
H	-3.79438	-7.95254	-5.43821
C	-4.85871	-6.16531	-3.72817
H	-4.65473	-6.73231	-2.81644
H	-5.30628	-5.20922	-3.44570
H	-5.60239	-6.72379	-4.30184

R-P=C(Cl)-P(S)ClR

R= H

P	0.78600	-1.26507	-0.12928
C	0.59073	-0.53053	-1.62238
P	-0.99972	-0.77462	-2.45350
Cl	1.72803	0.37958	-2.50890
Cl	-1.87263	1.06120	-2.30019
S	-0.96161	-1.55533	-4.18878
H	2.13914	-0.86803	0.06859
H	-1.70798	-1.45674	-1.44477

R= Me

P	0.89882	-1.24252	-0.11834
C	0.57027	-0.53669	-1.60142
P	-1.03159	-0.79997	-2.41045
Cl	1.65894	0.39830	-2.53883
Cl	-1.84078	1.08664	-2.32588
S	-0.92491	-1.55145	-4.16566
C	-2.07525	-1.68330	-1.23663
H	-1.69574	-2.69881	-1.12077
H	-2.11629	-1.17571	-0.27336
H	-3.07028	-1.72178	-1.68248
C	2.64323	-0.78074	0.19477
H	2.75132	0.30502	0.24548
H	2.95503	-1.21842	1.14364
H	3.28936	-1.15075	-0.60444

R = *t*-Bu

P	1.66990	0.09407	-2.03934
C	0.25258	-0.11062	-1.16484
P	-1.20006	-0.57774	-2.17125
Cl	-0.01184	0.07542	0.53067
S	-0.72702	-1.30178	-3.88617
C	3.04598	0.61564	-0.87014
C	-2.39204	0.85455	-2.25226
C	4.25342	0.77278	-1.80130
H	4.07995	1.53618	-2.56462
H	5.12895	1.07262	-1.21732
H	4.49436	-0.16259	-2.31349
C	3.35059	-0.47460	0.15465
H	4.24904	-0.19708	0.71708
H	2.53827	-0.61496	0.86631
H	3.55061	-1.43377	-0.33019
C	2.75685	1.95845	-0.20457
H	2.53693	2.73210	-0.94532
H	1.92281	1.90463	0.49376
H	3.64329	2.28157	0.35257
C	-1.71450	1.94540	-3.08199
H	-2.41184	2.78105	-3.19168
H	-0.81171	2.32368	-2.59747
H	-1.44872	1.58359	-4.07604
C	-2.77325	1.39921	-0.87965
H	-3.19160	0.62703	-0.23264
H	-1.92774	1.85948	-0.36849
H	-3.53715	2.17009	-1.01940
C	-3.64010	0.35191	-2.97815
H	-4.32198	1.19603	-3.11651
H	-3.39358	-0.05917	-3.95791
H	-4.16226	-0.41313	-2.40084
Cl	-2.15981	-1.92014	-0.94819

R = Ph

P	0.93403	-1.11657	-0.07923
C	0.56811	-0.43887	-1.56960
P	-1.03963	-0.70289	-2.38310
Cl	1.57159	0.52824	-2.57804
S	-0.94574	-1.71286	-4.00755
C	2.68023	-0.72975	0.21975
C	3.00693	-0.25000	1.48954
C	3.70604	-1.00667	-0.68583
C	4.32749	-0.00498	1.83163
H	2.22029	-0.06370	2.21427
C	5.02586	-0.78175	-0.33291
H	3.47209	-1.40653	-1.66500
C	5.33832	-0.27180	0.92042
H	4.56749	0.38322	2.81479
H	5.81613	-1.00486	-1.04063
H	6.37289	-0.09250	1.18994
C	-1.78602	0.93771	-2.50415
C	-2.28049	1.36657	-3.72943
C	-1.86101	1.76729	-1.38730
C	-2.85498	2.62446	-3.83661
H	-2.20829	0.70806	-4.58763
C	-2.43171	3.02260	-1.50164
H	-1.47521	1.43025	-0.43150
C	-2.92979	3.45076	-2.72600
H	-3.24237	2.95892	-4.79193
H	-2.49017	3.66937	-0.63394
H	-3.37730	4.43448	-2.81220
Cl	-2.13927	-1.58699	-0.90122

R = Mes

P	1.76949	-0.04887	-2.16118
C	0.35569	0.29532	-1.32760
P	-1.19456	-0.36989	-2.01081
Cl	0.21438	1.09254	0.19099
S	-0.99357	-1.22131	-3.71869
C	3.07178	0.58089	-1.05245
C	3.72258	1.77687	-1.38073
C	3.51343	-0.19234	0.03226
C	4.79067	2.19844	-0.59794
C	4.58632	0.26660	0.78401
C	5.23455	1.46154	0.49167
H	5.29297	3.12722	-0.85290
H	4.93184	-0.33369	1.62091
C	-2.52143	0.86974	-1.91224
C	-3.85556	0.47573	-1.68753
C	-2.23314	2.22141	-2.19227
C	-4.83323	1.45974	-1.59873
C	-3.25419	3.15713	-2.08868
C	-4.55413	2.80768	-1.75657
H	-5.85835	1.14794	-1.42231
H	-3.02223	4.19617	-2.30221
C	3.27218	2.61168	-2.54238
H	3.10332	2.00330	-3.43712
H	2.33001	3.12244	-2.32250
H	4.01360	3.37283	-2.78936
C	2.85645	-1.49314	0.38417
H	1.83290	-1.33960	0.73737
H	2.79873	-2.16330	-0.47902
H	3.41023	-2.00739	1.17059
C	6.37167	1.94398	1.33943

H	6.00004	2.45341	2.23411
H	6.99752	1.11471	1.67543
H	7.00155	2.65082	0.79689
C	-4.32517	-0.94893	-1.60581
H	-5.36817	-0.99717	-1.92274
H	-3.74881	-1.61415	-2.24912
H	-4.26776	-1.34034	-0.58837
C	-5.62169	3.84548	-1.60453
H	-5.66180	4.20686	-0.57207
H	-5.43304	4.70899	-2.24495
H	-6.60661	3.44343	-1.84817
C	-0.90108	2.71868	-2.67473
H	-0.18810	2.84305	-1.85736
H	-0.45782	2.04558	-3.41011
H	-1.03034	3.69236	-3.14910
Cl	-1.55506	-1.68003	-0.45398

R = Mes*

P	2.26179	-1.89753	-0.67438
C	1.32041	-0.98120	-1.71769
P	-0.24491	-1.57246	-2.48747
Cl	1.56203	0.69359	-2.03773
C	3.49195	-0.71162	0.02426
C	4.75141	-0.52617	-0.59800
C	3.17556	0.02706	1.19932
C	5.57088	0.50222	-0.13403
C	4.04983	1.03264	1.58937
C	5.23059	1.32349	0.92331
H	6.51656	0.66512	-0.62415
H	3.80450	1.61862	2.46214
C	-0.65627	-3.36996	-2.67472
C	-1.77638	-3.83255	-1.92156
C	-0.33893	-4.06923	-3.88147
C	-2.72001	-4.61864	-2.57735
C	-1.33352	-4.84472	-4.45849
C	-2.57889	-5.05044	-3.88282
H	-3.60435	-4.90239	-2.02516
H	-1.11474	-5.32348	-5.40438
C	5.30462	-1.38064	-1.75816
C	1.94968	-0.22310	2.10475
C	6.10916	2.47794	1.39522
C	-3.66884	-5.79699	-4.64021
C	1.05036	-4.24375	-4.53598
C	-1.99787	-3.73601	-0.39481
C	4.65319	-0.96949	-3.08178
H	3.57421	-1.10330	-3.06992
H	4.85538	0.08275	-3.29418
H	5.06035	-1.56767	-3.90230
C	6.81753	-1.19709	-1.93913
H	7.08114	-0.19937	-2.29568
H	7.36978	-1.39628	-1.01752
H	7.16379	-1.90491	-2.69579
C	5.11089	-2.87658	-1.49213
H	5.58692	-3.16826	-0.55264
H	4.06292	-3.18079	-1.44039
C	0.66675	0.33077	1.47621
H	0.76389	1.40354	1.29504
H	0.41229	-0.14024	0.52941
H	-0.17454	0.17800	2.15840
C	2.09341	0.46926	3.46681
H	3.01533	0.18521	3.98023
H	2.05546	1.55802	3.39016
H	1.25384	0.16728	4.09697
C	1.80761	-1.71451	2.42567
H	1.60974	-2.33351	1.54782
H	2.71388	-2.09540	2.90346
H	0.96834	-1.86631	3.10954
C	7.35624	2.64944	0.53201
H	7.93832	3.49889	0.89757
H	8.00231	1.76860	0.56847
H	7.10255	2.84756	-0.51244
C	5.30206	3.78031	1.33509
H	4.97492	3.99012	0.31386
H	4.41341	3.73547	1.96776
H	5.91430	4.61961	1.67690
C	6.55599	2.22546	2.84009
H	7.18206	3.05079	3.19089
H	5.70484	2.14103	3.51868
H	7.13661	1.30254	2.91362
C	-3.33573	-3.09045	-0.02493
H	-4.18161	-3.60530	-0.48487
H	-3.47239	-3.13724	1.05920
H	-3.36177	-2.04605	-0.33310
C	-2.00593	-5.19678	0.10865
H	-2.10168	-5.19659	1.19787
H	-2.83223	-5.77974	-0.29855
H	-1.07354	-5.70498	-0.14997
C	-0.87923	-3.05715	0.37996
H	0.08900	-3.49998	0.13720
H	-0.83765	-1.98501	0.20429
H	-1.05066	-3.20644	1.44928
C	1.06299	-3.90588	-6.02978
H	2.04983	-4.13365	-6.44214
H	0.33284	-4.48960	-6.59294
H	0.85910	-2.85015	-6.20354
C	1.38490	-5.74284	-4.37740
H	2.38418	-5.93299	-4.77912
H	1.38051	-6.03352	-3.32405
H	0.68103	-6.38583	-4.90660
C	2.18447	-3.50313	-3.84943
H	2.20786	-3.72666	-2.78169
H	3.13456	-3.83250	-4.27779
H	2.12881	-2.42670	-3.98236
C	-3.96434	-5.04572	-5.94400
H	-3.07768	-4.97169	-6.57681
H	-4.74003	-5.56770	-6.51119
H	-4.31424	-4.03118	-5.73949
C	-3.19261	-7.21688	-4.96907
H	-2.29623	-7.21092	-5.59247
H	-2.96496	-7.77459	-4.05730
H	-3.97230	-7.75725	-5.51311
C	-4.96399	-5.89643	-3.83883
H	-4.83540	-6.48229	-2.92510
H	-5.34627	-4.91004	-3.56507
H	-5.72797	-6.39411	-4.44090
S	-1.71338	-0.52286	-1.80963
Cl	0.06026	-1.01216	-4.45148
H	5.56332	-3.45657	-2.30098

R-P=C(Cl)-P(S)MeR

R = H

P	0.87571	-1.32772	-0.15207
C	0.59633	-0.54944	-1.60738
P	-1.02898	-0.75259	-2.40692
Cl	1.69880	0.41073	-2.49963
S	-1.01799	-1.61877	-4.12601
H	2.22574	-0.90207	0.01264
H	-1.68637	-1.44085	-1.35989
C	-1.77057	0.89445	-2.34944

H	-2.79361	0.81644	-2.72026
H	-1.76586	1.28886	-1.33165
H	-1.20547	1.55231	-3.01082

R = Me

P	1.79954	-0.04881	-2.25999
C	0.32837	-0.02642	-1.46268
P	-1.16270	-0.55293	-2.35997
Cl	0.07225	0.46239	0.18104
S	-0.80053	-1.08398	-4.18484
C	-1.87315	-1.85777	-1.32423
H	-1.20168	-2.71636	-1.34562
H	-2.01845	-1.52175	-0.29644
H	-2.82998	-2.14905	-1.76059
C	2.95519	0.55348	-0.96253
H	2.94527	-0.10660	-0.09178
H	3.96257	0.57944	-1.37957
H	2.68042	1.55680	-0.62806
C	-2.30277	0.84470	-2.19580
H	-1.90130	1.68350	-2.76479
H	-3.25924	0.55059	-2.63103
H	-2.44004	1.13184	-1.15220

R = *t*-Bu

P	1.64394	0.11191	-2.04027
C	0.23583	-0.16025	-1.17522
P	-1.23578	-0.64574	-2.15690
Cl	-0.01888	-0.07247	0.54108
S	-0.75930	-1.35704	-3.90088
C	-2.08683	-1.86172	-1.11412
H	-1.38658	-2.67392	-0.91568
H	-2.44111	-1.44666	-0.17112
H	-2.92133	-2.25918	-1.69182
C	3.01177	0.62982	-0.85701
C	-2.35359	0.84996	-2.26482
C	4.20737	0.87028	-1.78528
H	4.00209	1.65975	-2.51340
H	5.07619	1.17477	-1.19342
H	4.47661	-0.03247	-2.34014
C	3.36225	-0.49220	0.11749
H	4.25465	-0.20924	0.68710
H	2.55997	-0.69138	0.82676
H	3.59132	-1.42170	-0.41051
C	2.68242	1.93060	-0.12977
H	2.42823	2.72759	-0.83385
H	1.85656	1.81649	0.57119
H	3.56133	2.26089	0.43541
C	-1.68491	1.87267	-3.17876
H	-2.35343	2.73022	-3.30102
H	-0.74446	2.23625	-2.75850
H	-1.47793	1.44720	-4.16162
C	-2.64081	1.48098	-0.90536
H	-3.08393	0.77597	-0.19892
H	-1.74470	1.90400	-0.45040
H	-3.35782	2.29614	-1.04542
C	-3.66148	0.37537	-2.90145
H	-4.29622	1.24574	-3.09313
H	-3.47906	-0.13348	-3.84969
H	-4.21701	-0.29744	-2.24390

R = Ph

P	1.70938	-0.11496	-1.98689
C	0.20792	-0.05643	-1.24135
P	-1.23229	-0.56660	-2.24145
Cl	-0.15977	0.39921	0.38394
S	-0.72973	-1.15194	-4.01797
C	-2.03658	-1.84616	-1.24591
H	-1.38587	-2.72106	-1.25924
H	-2.20478	-1.53100	-0.21614
H	-2.98417	-2.09853	-1.72339
C	2.87885	0.50385	-0.73860
C	2.75187	1.73080	-0.08499
C	4.03655	-0.25048	-0.53890
C	3.74925	2.17616	0.76644
H	1.87316	2.34330	-0.24822
C	5.02257	0.18571	0.33235
H	4.16451	-1.18976	-1.06837
C	4.88050	1.40110	0.98494
H	3.64289	3.13343	1.26426
H	5.90835	-0.41861	0.49133
H	5.65632	1.75092	1.65647
C	-2.34599	0.87022	-2.21683
C	-3.18405	1.15006	-1.13896
C	-2.34930	1.71415	-3.32429
C	-4.01225	2.26146	-1.17118
H	-3.19813	0.50545	-0.26904
C	-3.17268	2.82816	-3.34944
H	-1.71333	1.47269	-4.16900
C	-4.00508	3.10287	-2.27390
H	-4.66595	2.46905	-0.33174
H	-3.17032	3.47884	-4.21640
H	-4.65431	3.97087	-2.29718

R = Mes

P	1.78406	-0.02386	-2.22482
C	0.33483	0.24235	-1.42852
P	-1.19456	-0.45367	-2.16205
Cl	0.12291	0.96375	0.12661
S	-0.93779	-1.10473	-3.97276
C	-1.43977	-1.80027	-0.96178
H	-0.48422	-2.32641	-0.93591
H	-1.67741	-1.42593	0.03457
H	-2.20401	-2.48868	-1.31017
C	3.03932	0.60348	-1.05527
C	3.65470	1.83458	-1.31651
C	3.49111	-0.19824	0.00426
C	4.69283	2.25839	-0.49482
C	4.53269	0.26318	0.79738
C	5.14337	1.49150	0.57057
H	5.16661	3.21408	-0.70044
H	4.88383	-0.36127	1.61414
C	-2.49724	0.81797	-1.98384
C	-3.80847	0.49370	-1.58290
C	-2.20729	2.14479	-2.36207
C	-4.74697	1.51160	-1.45646
C	-3.18516	3.12073	-2.21607
C	-4.45371	2.83675	-1.73654
H	-5.75373	1.24718	-1.14709
H	-2.94397	4.13815	-2.50932
C	3.20077	2.70636	-2.44920
H	3.03826	2.12997	-3.36566
H	2.25395	3.20100	-2.21339
H	3.93617	3.48274	-2.66514
C	2.87560	-1.53503	0.29147
H	1.85011	-1.42606	0.65606
H	2.83536	-2.16214	-0.60436
H	3.44652	-2.07076	1.05099
C	6.24736	1.97552	1.46036
H	5.84269	2.42720	2.37160
H	6.89976	1.15550	1.76759
H	6.85794	2.73078	0.96284

C	-4.30423	-0.90509	-1.34666
H	-5.39438	-0.90481	-1.32808
H	-3.99088	-1.58259	-2.14328
H	-3.96474	-1.31772	-0.39470
C	-5.47383	3.91578	-1.54767
H	-5.39838	4.34654	-0.54417
H	-5.33140	4.72834	-2.26245
H	-6.48850	3.53022	-1.66205
C	-0.90223	2.57326	-2.96549
H	-0.14139	2.74190	-2.19997
H	-0.51604	1.83207	-3.66698
H	-1.04048	3.51166	-3.50451

R = Mes*

P	2.23839	-1.88922	-0.63350
C	1.30880	-0.99707	-1.70518
P	-0.27719	-1.55167	-2.48423
Cl	1.59565	0.66834	-2.08110
C	-0.16301	-0.96072	-4.20032
H	0.74349	-1.24108	-4.72854
H	-1.03945	-1.34780	-4.72047
H	-0.23690	0.12372	-4.14411
C	3.48182	-0.70364	0.04580
C	4.75450	-0.55371	-0.55853
C	3.15951	0.07019	1.19699
C	5.58212	0.47546	-0.11071
C	4.04123	1.07557	1.57128
C	5.23600	1.33199	0.91621
H	6.53818	0.61069	-0.58903
H	3.79087	1.68923	2.42335
C	-0.67029	-3.36666	-2.66922
C	-1.75956	-3.87979	-1.90824
C	-0.37769	-4.02125	-3.90684
C	-2.70179	-4.67193	-2.56267
C	-1.36816	-4.79990	-4.48829
C	-2.58986	-5.06004	-3.88383
H	-3.56450	-4.99106	-1.99577
H	-1.16432	-5.24179	-5.45571
C	5.31379	-1.44690	-1.68640
C	1.92209	-0.14380	2.09728
C	6.12256	2.48792	1.36940
C	-3.68147	-5.81410	-4.63226
C	0.99878	-4.13684	-4.60152
C	-1.95629	-3.81997	-0.37687
C	4.69202	-1.05457	-3.02943
H	3.61016	-1.16342	-3.02446
H	4.92104	-0.01257	-3.26431
H	5.09592	-1.68230	-3.82946
C	6.83274	-1.29462	-1.84469
H	7.12119	-0.31085	-2.22031
H	7.36444	-1.48050	-0.90840
H	7.17985	-2.02719	-2.57715
C	5.08773	-2.93298	-1.39169
H	5.54055	-3.21186	-0.43698
H	4.03346	-3.21605	-1.35241
C	0.64672	0.39847	1.44398
H	0.75616	1.46175	1.21853
H	0.38171	-0.10902	0.51918
H	-0.19605	0.28495	2.13199
C	2.05986	0.58762	3.43961
H	2.97540	0.31294	3.96947
H	2.02955	1.67396	3.33065
H	1.21289	0.30970	4.07086
C	1.76646	-1.62424	2.46149
H	1.57015	-2.26680	1.60067
H	2.66582	-1.99747	2.95824
H	0.92056	-1.74836	3.14281
C	7.38430	2.61989	0.52065
H	7.97163	3.47224	0.87087
H	8.01859	1.73261	0.59153
H	7.14834	2.79121	-0.53269
C	5.33362	3.79832	1.26173
H	5.02469	3.98430	0.23023
H	4.43491	3.78165	1.88137
H	5.95138	4.63895	1.59025
C	6.54496	2.27000	2.82720
H	7.17657	3.09669	3.16478
H	5.68279	2.21490	3.49477
H	7.11221	1.34211	2.93468
C	-3.29958	-3.20490	0.02568
H	-4.14339	-3.73078	-0.42613
H	-3.41559	-3.26920	1.11143
H	-3.34789	-2.15723	-0.26818
C	-1.93003	-5.28892	0.09981
H	-2.00344	-5.30891	1.19063
H	-2.75491	-5.87953	-0.29985
H	-0.99461	-5.77686	-0.18596
C	-0.83647	-3.13320	0.38840
H	0.13292	-3.56777	0.13605
H	-0.80963	-2.06043	0.20975
H	-0.99689	-3.28445	1.45934
C	0.97105	-3.71230	-6.07368
H	1.95404	-3.88320	-6.52112
H	0.24592	-4.28879	-6.65066
H	0.72638	-2.65748	-6.19909
C	1.36520	-5.63575	-4.54903
H	2.36054	-5.78087	-4.97863
H	1.38389	-5.99376	-3.51709
H	0.66415	-6.25639	-5.10740
C	2.13809	-3.43094	-3.88539
H	2.19098	-3.73691	-2.83944
H	3.08300	-3.70319	-4.36214
H	2.07359	-2.34730	-3.91186
C	-4.04629	-5.02734	-5.89687
H	-3.18410	-4.89949	-6.55474
H	-4.82235	-5.55478	-6.45861
H	-4.42432	-4.03415	-5.64288
C	-3.17367	-7.20516	-5.02957
H	-2.30092	-7.14809	-5.68327
H	-2.89561	-7.78671	-4.14720
H	-3.95493	-7.75152	-5.56541
C	-4.94443	-5.98490	-3.79237
H	-4.76468	-6.59633	-2.90449
H	-5.34937	-5.02220	-3.47103
H	-5.71196	-6.48771	-4.38573
S	-1.73993	-0.54169	-1.67052
H	5.54610	-3.53857	-2.17830

PCPX-E(II) model systems

E = Si(II)

R-P=C(Si(II)Cl)-P(O)ClR

R= H

P	0.78783	0.09855	1.08065
C	0.44291	-0.29030	-0.49355
P	-1.05815	-0.29005	-1.34871
Cl	1.12156	-2.66830	-2.35917
Si	1.30782	-0.53452	-2.28854

O	-0.50531	-0.12690	-2.76929
H	2.19863	-0.13316	1.01997
H	-1.77588	-1.48710	-1.19906
Cl	-2.46836	1.07178	-0.92807

R = Me

P	0.84161	0.22802	1.00849
C	0.43296	-0.21088	-0.53535
P	-1.10101	-0.29269	-1.32158
Cl	1.28893	-2.60619	-2.35371
Si	1.23951	-0.45169	-2.34237
O	-0.58989	-0.23174	-2.77471
C	2.65918	-0.02706	1.02877
H	3.10761	0.21259	0.06173
H	3.11018	0.58194	1.81364
H	2.86536	-1.07820	1.25175
C	-2.03599	-1.77900	-1.00372
H	-2.37954	-1.78431	0.03145
H	-2.88908	-1.81816	-1.68294
H	-1.36665	-2.62175	-1.18972
Cl	-2.41578	1.20370	-0.98560

R = *t*-Bu

P	0.65866	0.19207	0.61811
C	0.47677	-0.29956	-0.95859
P	-1.04320	-0.56188	-1.76551
Cl	1.94838	-2.58920	-2.57951
Si	1.34042	-0.53514	-2.74561
O	-0.48171	-0.80713	-3.18094
C	-2.13384	-1.92414	-1.20940
C	-2.71573	-1.58655	0.16051
H	-3.37569	-0.71882	0.12239
H	-3.30028	-2.44260	0.50848
H	-1.93371	-1.39522	0.89975
C	-3.23673	-2.10543	-2.25280
H	-2.82269	-2.30575	-3.24274
H	-3.84855	-2.96361	-1.96121
H	-3.88810	-1.23241	-2.31564
C	-1.27337	-3.18685	-1.12834
H	-0.82330	-3.43800	-2.08918
H	-0.47053	-3.08852	-0.39618
H	-1.91713	-4.01588	-0.82144
C	2.49338	0.16202	0.98166
C	2.92710	-1.30084	1.08784
H	2.33903	-1.84540	1.83278
H	2.83178	-1.81856	0.13194
H	3.97583	-1.34571	1.40218
C	3.30178	0.89170	-0.08226
H	4.36046	0.89067	0.19934
H	3.22383	0.41638	-1.06286
H	2.98640	1.93266	-0.18917
C	2.66378	0.85480	2.33431
H	3.71565	0.82740	2.63447
H	2.35439	1.90277	2.29424
H	2.07923	0.36310	3.11686
Cl	-2.24052	1.07366	-1.80758

R = Ph

P	0.97589	-0.65819	1.28578
C	0.32104	-0.36603	-0.21523
P	-1.35280	-0.29961	-0.63551
Cl	0.96406	-1.14946	-3.23488
Si	0.67927	0.55101	-1.94245
O	-1.21782	0.53227	-1.92467
Cl	-2.55539	0.64120	0.69061
C	-2.16668	-1.85843	-0.94652
C	-2.38816	-2.24611	-2.26454
C	-2.53407	-2.69005	0.10990
C	-2.98819	-3.46969	-2.52092
H	-2.08582	-1.59635	-3.07603
C	-3.12743	-3.91023	-0.15735
H	-2.36036	-2.38421	1.13582
C	-3.35560	-4.29823	-1.47218
H	-3.16452	-3.77546	-3.54533
H	-3.41524	-4.56027	0.66039
H	-3.82271	-5.25464	-1.67776
C	2.75904	-0.63489	1.03086
C	3.39458	-0.80391	-0.20342
C	3.53857	-0.43639	2.17581
C	4.77472	-0.74652	-0.28809
H	2.80958	-1.01498	-1.09131
C	4.91864	-0.37330	2.08619
H	3.05191	-0.32059	3.13959
C	5.53656	-0.52557	0.85262
H	5.25977	-0.88040	-1.24811
H	5.51344	-0.20909	2.97730
H	6.61747	-0.48222	0.78023

R = Mes

P	0.84879	0.09481	1.01712
C	0.42254	-0.50327	-0.47320
P	-1.10997	-0.48680	-1.30743
C	2.68895	0.07342	0.92447
C	3.42579	-1.11295	1.16878
C	3.35896	1.24737	0.50338
C	4.75567	-1.14200	0.77071
C	4.69578	1.13166	0.12537
C	5.40022	-0.05731	0.19328
H	5.32010	-2.05121	0.91950
H	5.20507	2.00839	-0.24017
C	2.72915	2.65928	0.50145
C	2.87109	-2.34448	1.91518
C	6.84125	-0.19527	-0.28830
C	2.05294	-3.25734	0.99648
H	1.16740	-2.76707	0.59696
H	2.64976	-3.58675	0.14402
H	1.73279	-4.14473	1.55140
C	2.02815	-1.90801	3.11647
H	2.60541	-1.27244	3.79264
H	1.12599	-1.35536	2.83467
H	1.69021	-2.78724	3.67183
C	4.00499	-3.20074	2.49456
H	4.57814	-3.71386	1.71977
H	4.69330	-2.61146	3.10533
H	3.56875	-3.97515	3.12990
C	1.69321	2.85172	-0.61296
H	0.77836	2.28522	-0.44081
H	1.41218	3.90726	-0.67062
H	2.09687	2.55076	-1.58205
C	2.09029	2.94563	1.86660
H	1.26216	2.27164	2.09925
H	2.82880	2.85840	2.66729
H	1.68803	3.96245	1.88036
C	3.78830	3.74834	0.29473
H	4.59548	3.68394	1.02806
H	4.22284	3.72107	-0.70712
H	3.30998	4.72346	0.41115
C	7.37345	1.09543	-0.90543
H	7.39953	1.91425	-0.18176
H	8.39564	0.93596	-1.25724
H	6.77386	1.41170	-1.76259

C	6.90711	-1.29743	-1.35249
H	6.57914	-2.26100	-0.95755
H	6.27085	-1.05392	-2.20655
H	7.93364	-1.41405	-1.71152
C	7.74612	-0.57294	0.89033
H	7.44458	-1.51784	1.34683
H	8.78047	-0.68139	0.55178
H	7.72056	0.19748	1.66511
C	-2.24049	-1.88538	-1.15475
C	-2.62836	-2.68155	-2.26574
C	-2.73444	-2.17455	0.13289
C	-3.46314	-3.76187	-2.01947
C	-3.57270	-3.26950	0.29963
C	-3.94294	-4.08174	-0.75598
H	-3.75513	-4.38211	-2.85686
H	-3.95137	-3.49448	1.29109
C	-2.25392	-2.38892	-3.70724
C	-3.16826	-1.29896	-4.27062
C	-2.26982	-3.61180	-4.61648
H	-1.23656	-2.00939	-3.72238
H	-3.11458	-0.37790	-3.68745
H	-2.87976	-1.05890	-5.29691
H	-4.20976	-1.63403	-4.27869
H	-1.67890	-4.43115	-4.20160
H	-3.28071	-3.97736	-4.81790
H	-1.83261	-3.34158	-5.58018
C	-2.43828	-1.34332	1.36412
C	-1.80843	-2.17775	2.47622
C	-3.69683	-0.63333	1.86090
H	-1.71621	-0.56248	1.11240
H	-0.91907	-2.70410	2.12588
H	-1.51953	-1.53367	3.31013
H	-2.50902	-2.92236	2.86143
H	-4.13724	-0.00937	1.08159
H	-4.45224	-1.35435	2.18319
H	-3.45607	0.00469	2.71482
C	-4.84369	-5.27294	-0.53835
C	-4.13077	-6.58015	-0.87764
C	-6.15115	-5.14276	-1.31599
H	-5.09076	-5.29404	0.52904
H	-3.20876	-6.69132	-0.30324
H	-4.77586	-7.43479	-0.65866
H	-3.87010	-6.62307	-1.93835
H	-6.67729	-4.22085	-1.05925
H	-5.97089	-5.13613	-2.39409
H	-6.81114	-5.98559	-1.09588
Cl	1.30069	-2.72813	-2.55678
Si	1.30520	-0.58633	-2.25681
O	-0.50559	-0.28834	-2.71353
Cl	-2.26164	1.16582	-0.96374

R = Mes*

P	1.20466	0.95553	1.22664
C	0.44039	0.39573	-0.13846
P	-1.23677	0.31302	-0.63610
Cl	1.53608	-2.36702	-1.31070
O	-0.98909	-0.66880	-1.79873
C	-2.85166	-0.03796	0.13267
C	-3.42209	-1.31793	-0.13185
C	-3.70296	1.01698	0.54920
C	-4.78913	-1.36531	-0.35992
C	-5.06748	0.87594	0.32077
C	-5.62657	-0.26458	-0.23175
H	-5.22241	-2.31837	-0.63323
H	-5.71392	1.69795	0.59444
C	2.96664	0.63423	0.80916
C	3.60928	-0.48247	1.40161
C	3.63562	1.37999	-0.18734
C	4.77666	-0.95184	0.81009
C	4.79475	0.83710	-0.73365
C	5.35392	-0.35269	-0.30040
H	5.24784	-1.82970	1.22160
H	5.28413	1.37846	-1.53395
C	3.11624	-1.16431	2.69441
C	3.30084	2.81301	-0.65373
C	6.57604	-0.92757	-1.00975
C	-3.29747	2.23329	1.40380
C	-2.69363	-2.67224	-0.02668
C	-7.10052	-0.37333	-0.60118
C	4.51937	3.69103	-0.31305
H	4.33273	4.71958	-0.63435
H	4.70494	3.69848	0.76425
H	5.42967	3.34817	-0.80545
C	2.10962	3.47350	0.03633
H	1.15276	3.07575	-0.30097
H	2.16850	3.39734	1.12745
H	2.11060	4.54085	-0.20385
C	3.05779	2.85994	-2.16561
H	3.92849	2.52015	-2.72942
H	2.21386	2.22759	-2.45030
H	2.84300	3.88699	-2.47507
C	7.73692	0.07101	-0.93712
H	8.61420	-0.33311	-1.45028
H	7.48447	1.02204	-1.41047
H	8.01334	0.27509	0.10042
C	7.03903	-2.24474	-0.39259
H	7.89727	-2.62617	-0.95123
H	7.35273	-2.12114	0.64727
H	6.25545	-3.00548	-0.42891
C	6.21783	-1.18589	-2.47836
H	5.38864	-1.89248	-2.55914
H	5.92144	-0.26740	-2.98891
H	7.07901	-1.60227	-3.00889
C	4.12082	-2.19805	3.21215
H	5.10947	-1.76449	3.37965
H	3.76191	-2.58358	4.16926
H	4.22266	-3.05036	2.53641
C	2.96961	-0.11039	3.80082
H	3.92178	0.39411	3.98245
H	2.22546	0.65117	3.55918
H	2.65455	-0.59058	4.73175
C	1.79162	-1.90925	2.50180
H	0.95285	-1.23358	2.32033
H	1.84389	-2.59801	1.65679
H	1.55518	-2.47733	3.40663
C	-1.81789	2.29390	1.75525
H	-1.46152	1.34638	2.16614
H	-1.18785	2.57632	0.91342
H	-1.66129	3.05314	2.52538
C	-4.03812	2.05767	2.74585
H	-3.76432	2.87532	3.41810
H	-5.12196	2.06874	2.62670
H	-3.76041	1.11572	3.22497
C	-3.72718	3.56609	0.78485
H	-4.80242	3.60461	0.60108
H	-3.48231	4.38119	1.47118
H	-3.21525	3.75261	-0.15885
C	-1.38052	-2.55833	0.74326
H	-0.96505	-3.55712	0.89708
H	-0.60682	-2.00011	0.22304
H	-1.54544	-2.10929	1.72653
C	-2.48472	-3.33989	-1.38882

H	-1.81709	-2.76380	-2.02402
H	-2.05298	-4.33408	-1.24537
H	-3.43912	-3.46156	-1.90821
C	-3.57490	-3.61088	0.82260
H	-3.84238	-3.15402	1.77848
H	-4.49356	-3.90973	0.31660
H	-3.01103	-4.52389	1.02881
C	-7.84565	0.94382	-0.40231
H	-7.86228	1.25023	0.64676
H	-7.40451	1.75173	-0.99135
H	-8.88311	0.82709	-0.72414
C	-7.77342	-1.44517	0.26468
H	-7.31818	-2.42730	0.12369
H	-7.70493	-1.19194	1.32550
H	-8.83167	-1.52778	0.00225
C	-7.20880	-0.77113	-2.07826
H	-6.74635	-0.01853	-2.72124
H	-6.71917	-1.72646	-2.27677
H	-8.25964	-0.86625	-2.36497
Si	0.90058	-0.40351	-1.91658
Cl	-1.61664	2.08730	-1.59641

R-P=C(Si(II)Cl)-P(O)MeR

R= H

P	0.84178	0.04401	1.07484
C	0.47022	-0.29468	-0.50374
P	-1.05794	-0.24430	-1.34110
Cl	1.12088	-2.69170	-2.30979
Si	1.31530	-0.54912	-2.30172
O	-0.48505	-0.16242	-2.77297
C	-2.23308	1.05998	-0.99714
H	-3.07111	0.99102	-1.69396
H	-2.60021	0.95215	0.02632
H	-1.73997	2.02612	-1.10992
H	2.25720	-0.15447	0.99148
H	-1.77725	-1.44043	-1.14831

R = Me

P	0.76849	0.09811	1.00485
C	0.39150	-0.23545	-0.57125
P	-1.15284	-0.27826	-1.37554
Cl	1.34418	-2.55140	-2.41711
Si	1.21847	-0.39302	-2.37490
O	-0.59466	-0.23135	-2.82467
C	-2.28772	1.08140	-1.08779
H	-3.14616	0.99617	-1.75767
H	-2.63054	1.06352	-0.05111
H	-1.76814	2.02032	-1.28083
C	2.59601	-0.08972	1.04706
H	3.04935	0.18944	0.09354
H	3.01382	0.51462	1.85402
H	2.83604	-1.13780	1.24943
C	-2.05120	-1.79944	-1.07988
H	-2.39670	-1.83264	-0.04460
H	-2.90503	-1.86169	-1.75799
H	-1.36953	-2.62980	-1.26961

R = *t*-Bu

P	0.66552	0.11636	0.72656
C	0.44086	-0.27808	-0.86995
P	-1.11618	-0.46662	-1.65676
Cl	1.90574	-2.45851	-2.65051
Si	1.24712	-0.40849	-2.69270
O	-0.55592	-0.68175	-3.09423
C	-2.13327	1.01691	-1.61701
H	-3.02279	0.89764	-2.23746
H	-2.42276	1.25204	-0.59189
H	-1.52814	1.83423	-2.01183
C	-2.14658	-1.89011	-1.13243
C	-2.74377	-1.59617	0.24291
H	-3.45512	-0.76777	0.21746
H	-3.28251	-2.48231	0.59001
H	-1.97100	-1.36837	0.98233
C	-3.24718	-2.08448	-2.17781
H	-2.82879	-2.23536	-3.17482
H	-3.82647	-2.97453	-1.91611
H	-3.94115	-1.24170	-2.21367
C	-1.26624	-3.13710	-1.07097
H	-0.79702	-3.35115	-2.03185
H	-0.47327	-3.03649	-0.32846
H	-1.89115	-3.99080	-0.79292
C	2.51185	0.10497	1.04121
C	2.98180	-1.34995	1.03727
H	2.43042	-1.95528	1.76315
H	2.86446	-1.80676	0.05316
H	4.04157	-1.39097	1.31341
C	3.27194	0.92149	0.00510
H	4.34029	0.91915	0.24839
H	3.16338	0.51613	-1.00354
H	2.93731	1.96194	-0.01516
C	2.70796	0.71117	2.43092
H	3.76882	0.69053	2.69893
H	2.37333	1.75158	2.46941
H	2.15844	0.15485	3.19548

R = Ph

P	0.91565	-0.53044	1.06071
C	0.35667	-0.33559	-0.49162
P	-1.31374	-0.23503	-0.98296
Cl	1.13886	-1.46917	-3.34916
Si	0.81908	0.38235	-2.28434
O	-1.05582	0.40260	-2.37485
C	-2.13426	-1.82013	-1.16117
C	-2.37344	-2.32460	-2.43548
C	-2.51700	-2.54802	-0.03552
C	-3.00719	-3.54979	-2.57962
H	-2.05225	-1.76093	-3.30276
C	-3.14678	-3.77101	-0.18735
H	-2.31034	-2.17237	0.96164
C	-3.39432	-4.26986	-1.45997
H	-3.19199	-3.94456	-3.57176
H	-3.44145	-4.33887	0.68739
H	-3.88624	-5.22879	-1.57680
C	2.71542	-0.56181	0.92895
C	3.42133	-0.83436	-0.24636
C	3.42705	-0.29830	2.10363
C	4.80544	-0.81507	-0.24633
H	2.88626	-1.09333	-1.15292
C	4.81164	-0.27433	2.09903
H	2.88466	-0.10184	3.02371
C	5.50121	-0.53005	0.92195
H	5.34501	-1.02889	-1.16183
H	5.35388	-0.06003	3.01289
H	6.58536	-0.51724	0.91614
C	-2.39017	0.80313	0.00936
H	-3.36018	0.89865	-0.48231
H	-2.52673	0.36241	0.99877
H	-1.92600	1.78468	0.11307

R = Mes

P	1.08691	-1.10708	1.43205
C	0.33606	-0.58878	0.04827
P	-1.34330	-0.40256	-0.43626
Cl	1.12925	-0.91280	-2.95583
Si	0.82555	0.61729	-1.44799
O	-1.04275	0.67026	-1.52110
C	-2.16792	-1.86652	-1.10140
C	-2.73910	-1.86779	-2.39332
C	-2.27195	-3.00909	-0.28687
C	-3.38007	-3.02228	-2.82474
C	-2.93009	-4.13138	-0.77385
C	-3.48318	-4.16477	-2.04332
H	-3.82232	-3.02094	-3.81619
H	-3.00887	-5.00505	-0.13379
C	2.84985	-0.74714	1.10634
C	3.64293	-1.67764	0.42020
C	3.42380	0.41345	1.64202
C	4.99929	-1.41698	0.27127
C	4.78373	0.63406	1.46702
C	5.58838	-0.26654	0.78096
H	5.61447	-2.13839	-0.25902
H	5.22594	1.53676	1.87903
C	-2.46588	0.34798	0.75644
H	-3.35890	0.67379	0.21794
H	-2.75868	-0.35057	1.53936
H	-1.97191	1.21449	1.19829
C	2.58715	1.42732	2.36283
H	1.94415	1.96771	1.66090
H	1.93317	0.95914	3.10637
H	3.21217	2.15846	2.87750
C	3.05574	-2.92973	-0.15927
H	2.43951	-3.46239	0.57276
H	2.41618	-2.70173	-1.01686
H	3.84145	-3.60924	-0.49247
C	7.04699	0.00647	0.57430
H	7.62308	-0.91956	0.52603
H	7.21077	0.54223	-0.36608
H	7.45504	0.62338	1.37708
C	-1.67651	-3.11136	1.08623
H	-0.64189	-3.46091	1.03298
H	-1.65396	-2.17134	1.63744
H	-2.23711	-3.82950	1.68670
C	-2.70791	-0.70802	-3.34644
H	-3.07576	0.21538	-2.89870
H	-1.69106	-0.50621	-3.68655
H	-3.32525	-0.93744	-4.21551
C	-4.15686	-5.39510	-2.56548
H	-3.47072	-5.96984	-3.19523
H	-4.48353	-6.04694	-1.75387
H	-5.02492	-5.14375	-3.17800

R = Mes*

P	1.18182	1.07424	1.07833
C	0.40715	0.42425	-0.24018
P	-1.28667	0.36830	-0.76095
Cl	1.46512	-2.45970	-1.23156
O	-1.02849	-0.75377	-1.81663
C	-2.88467	0.04234	0.07908
C	-3.47323	-1.24367	-0.08685
C	-3.73171	1.13137	0.41070
C	-4.84268	-1.29742	-0.30875
C	-5.09930	0.98998	0.19592
C	-5.67175	-0.18483	-0.26397
H	-5.28331	-2.26515	-0.50996
H	-5.73794	1.83581	0.41017
C	2.94959	0.68681	0.72173
C	3.54833	-0.43122	1.34711
C	3.67915	1.42477	-0.24401
C	4.72723	-0.93144	0.79916
C	4.84770	0.85975	-0.73896
C	5.35747	-0.34935	-0.28748
H	5.16350	-1.81665	1.23301
H	5.39158	1.38721	-1.50982
C	3.01943	-1.06802	2.64991
C	3.32388	2.85576	-0.70852
C	6.59125	-0.95246	-0.95148
C	-3.31217	2.40257	1.17590
C	-2.75734	-2.59332	0.11069
C	-7.14935	-0.31066	-0.61477
C	4.58026	3.59786	-1.18738
H	4.32732	4.64825	-1.35089
H	5.38587	3.55346	-0.45073
H	4.95737	3.21267	-2.13669
C	2.77079	3.68549	0.45336
H	1.80509	3.32084	0.82022
H	3.46275	3.69520	1.29894
H	2.61022	4.71678	0.12683
C	2.32763	2.87227	-1.87132
H	2.67862	2.25495	-2.70039
H	1.35278	2.49367	-1.57005
H	2.20148	3.89775	-2.23297
C	7.77429	0.01488	-0.82962
H	8.65948	-0.40914	-1.31246
H	7.56421	0.97521	-1.30488
H	8.01688	0.20520	0.21899
C	6.99545	-2.28368	-0.32341
H	7.86243	-2.68734	-0.85229
H	7.27478	-2.17158	0.72749
H	6.19263	-3.02194	-0.39024
C	6.28418	-1.19650	-2.43411
H	5.44137	-1.88191	-2.54963
H	6.03084	-0.26883	-2.95128
H	7.15445	-1.63267	-2.93313
C	3.99282	-2.10974	3.20997
H	4.98878	-1.69480	3.38148
H	3.61078	-2.46150	4.17119
H	4.08472	-2.98180	2.55830
C	2.88783	0.02351	3.72156
H	3.84919	0.51290	3.89657
H	2.16326	0.79289	3.44658
H	2.55328	-0.41979	4.66405
C	1.67793	-1.78347	2.47149
H	0.86026	-1.09270	2.25983
H	1.71793	-2.50263	1.65191
H	1.42155	-2.31345	3.39397
C	-1.83203	2.46701	1.52731
H	-1.49672	1.55164	2.01916
H	-1.17746	2.65610	0.67773
H	-1.66249	3.28826	2.22785
C	-4.05005	2.33555	2.52908
H	-3.76729	3.19715	3.14017
H	-5.13387	2.34736	2.41038
H	-3.77975	1.42818	3.07403
C	-3.73641	3.69380	0.46941
H	-4.81598	3.73669	0.31496
H	-3.45850	4.55551	1.08207
H	-3.26162	3.81101	-0.50559
C	-1.43431	-2.43396	0.85290
H	-1.02144	-3.42158	1.07174
H	-0.66952	-1.91487	0.28288
H	-1.58449	-1.91552	1.80386
C	-2.57090	-3.36044	-1.20138

H	-1.90927	-2.83404	-1.88429
H	-2.14070	-4.34355	-0.99180
H	-3.53308	-3.51566	-1.69741
C	-3.63704	-3.45712	1.03729
H	-3.88715	-2.92799	1.96014
H	-4.56559	-3.78254	0.56662
H	-3.07953	-4.35839	1.30383
C	-7.88140	1.02561	-0.52399
H	-7.88973	1.41923	0.49566
H	-7.43578	1.77718	-1.18060
H	-8.92165	0.89272	-0.83030
C	-7.82568	-1.29823	0.34364
H	-7.37869	-2.29239	0.28458
H	-7.74725	-0.95572	1.37836
H	-8.88650	-1.39418	0.09622
C	-7.27374	-0.83210	-2.05138
H	-6.81351	-0.13977	-2.76053
H	-6.79104	-1.80379	-2.17213
H	-8.32752	-0.94495	-2.32045
Si	0.83141	-0.53326	-1.94811
C	-1.62980	1.81924	-1.78742
H	-2.55746	1.63272	-2.33172
H	-1.70360	2.75563	-1.24415
H	-0.80363	1.87618	-2.50044

R-P=C(Si(II)Cl)-P(S)ClR

R= H

P	0.99152	0.22682	0.98007
C	0.49617	-0.11461	-0.56518
P	-1.10124	-0.04251	-1.23519
S	-0.69541	0.57755	-3.07184
Cl	0.97149	-2.44742	-2.53073
Si	1.42024	-0.36218	-2.29969
Cl	-2.50871	1.01145	-0.22884
H	-1.67936	-1.32215	-1.20443
H	2.39015	0.01140	0.75994

R = Me

P	0.99559	0.05330	0.92101
C	0.48717	-0.17723	-0.64198
P	-1.13172	-0.10293	-1.25429
S	-0.77139	0.41055	-3.14649
Cl	1.23084	-2.43084	-2.66621
Si	1.38975	-0.30114	-2.38892
C	2.79794	-0.27392	0.82146
H	3.20800	-0.01883	-0.15937
H	3.31445	0.28810	1.60133
H	2.97181	-1.33904	1.00196
C	-1.97855	-1.67824	-1.09593
H	-1.34208	-2.43401	-1.56095
H	-2.12973	-1.90201	-0.03874
H	-2.93755	-1.62008	-1.61225
Cl	-2.38761	1.18338	-0.28961

R = *t*-Bu

P	0.92547	-0.11459	1.01122
C	0.51219	-0.04298	-0.60267
P	-1.15865	-0.14499	-1.11294
S	-1.01959	0.53455	-2.98568
Cl	1.89748	-1.38633	-3.13567
Si	1.28702	0.51038	-2.33461
C	-1.94543	-1.82072	-0.99031
C	-1.05042	-2.80386	-1.74198
H	-1.52102	-3.79036	-1.70146
H	-0.92759	-2.52596	-2.78938

H	-0.05785	-2.87951	-1.29605
C	-3.31870	-1.74496	-1.65396
H	-3.76317	-2.74394	-1.63886
H	-3.99219	-1.06767	-1.12647
H	-3.24453	-1.42662	-2.69590
C	-2.07002	-2.22856	0.47411
H	-2.73842	-1.56962	1.02923
H	-2.48161	-3.24114	0.51297
H	-1.10136	-2.24408	0.97852
C	2.79153	-0.04764	1.13437
C	3.42265	-1.23492	0.41285
H	3.04389	-2.18702	0.79535
H	3.23903	-1.20995	-0.66175
H	4.50626	-1.22283	0.57483
C	3.09959	-0.12888	2.63084
H	4.18191	-0.08869	2.78660
H	2.65010	0.70076	3.18329
H	2.73256	-1.06086	3.06960
C	3.30820	1.27600	0.57640
H	3.11844	1.37629	-0.49523
H	2.85377	2.13283	1.08105
H	4.39114	1.33641	0.73093
Cl	-2.39603	1.03535	0.00834

R = Ph

P	0.96493	0.36943	0.26073
C	0.45431	-0.22840	-1.20826
P	-1.18729	-0.22059	-1.76732
C	2.73102	0.01378	0.31850
C	3.35657	-1.03639	-0.36107
C	3.50365	0.86068	1.12061
C	4.72715	-1.20960	-0.26343
C	4.87432	0.68907	1.20895
C	5.48661	-0.34497	0.51337
H	5.20362	-2.02694	-0.79228
H	5.46596	1.35760	1.82364
C	-2.09635	-1.68543	-1.24699
C	-2.49161	-2.63275	-2.18271
C	-2.36929	-1.87186	0.10712
C	-3.16997	-3.76706	-1.76187
C	-3.04467	-3.00654	0.51792
C	-3.44609	-3.95332	-0.41685
H	-3.47777	-4.50810	-2.49001
H	-3.25855	-3.15428	1.56993
S	-0.91963	-0.09283	-3.73493
Cl	1.15581	-2.78716	-2.94222
Si	1.29589	-0.63822	-2.93866
H	-2.05642	-1.13314	0.83710
H	-2.25979	-2.48346	-3.23027
H	-3.97467	-4.84196	-0.09118
H	3.02212	1.66554	1.66776
H	6.55919	-0.48503	0.58653
H	2.76642	-1.74429	-0.93233
Cl	-2.31068	1.31051	-1.01440

R = Mes*

P	1.12946	0.69069	1.22483
C	0.43737	0.29826	-0.24228
P	-1.25661	0.31043	-0.70903
Cl	1.88974	-2.23060	-1.48792
C	-2.83915	-0.00696	0.17148
C	-3.42549	-1.29020	-0.03979
C	-3.68103	1.06038	0.58082
C	-4.79362	-1.33479	-0.26206
C	-5.04582	0.92972	0.34232

C	-5.61667	-0.21845	-0.18278
H	-5.23808	-2.29654	-0.48633
H	-5.68396	1.76558	0.59339
C	2.92278	0.51055	0.86181
C	3.61485	-0.61346	1.38233
C	3.58650	1.38292	-0.03320
C	4.82474	-0.96475	0.79551
C	4.79057	0.95319	-0.58314
C	5.39892	-0.24198	-0.24067
H	5.33295	-1.84727	1.14887
H	5.27895	1.59482	-1.30611
C	3.12707	-1.42545	2.59859
C	3.21860	2.85168	-0.33839
C	6.66647	-0.69073	-0.96041
C	-3.27455	2.25995	1.46189
C	-2.73730	-2.65358	0.18308
C	-7.08800	-0.31724	-0.56424
C	4.36501	3.71100	0.22766
H	4.16512	4.76806	0.03046
H	4.45523	3.57634	1.30879
H	5.32731	3.46173	-0.22064
C	1.94326	3.37093	0.31943
H	1.04042	2.98350	-0.15039
H	1.91249	3.15499	1.39327
H	1.91442	4.45999	0.21926
C	3.10459	3.10499	-1.84503
H	4.03676	2.89035	-2.37042
H	2.32375	2.48549	-2.29107
H	2.86058	4.15602	-2.02437
C	7.77078	0.35627	-0.77563
H	8.67934	0.04067	-1.29633
H	7.47993	1.32914	-1.17700
H	8.01285	0.48764	0.28207
C	7.18502	-2.02935	-0.44171
H	8.07491	-2.31807	-1.00640
H	7.46788	-1.97738	0.61297
H	6.44416	-2.82348	-0.56201
C	6.35294	-0.84339	-2.45393
H	5.56370	-1.58186	-2.61246
H	6.01835	0.09832	-2.89377
H	7.24572	-1.16955	-2.99526
C	4.17436	-2.44019	3.06617
H	5.12976	-1.96694	3.30441
H	3.80892	-2.92400	3.97500
H	4.34797	-3.22596	2.32736
C	2.88601	-0.47366	3.77884
H	3.79979	0.07109	4.02870
H	2.10229	0.25800	3.57338
H	2.57771	-1.04469	4.65936
C	1.85468	-2.22112	2.30062
H	0.99402	-1.57362	2.11916
H	1.97823	-2.85234	1.41911
H	1.60481	-2.85354	3.15801
C	-1.80300	2.29103	1.85185
H	-1.47792	1.33257	2.26183
H	-1.14374	2.56411	1.03092
H	-1.65591	3.04081	2.63340
C	-4.04431	2.05944	2.78564
H	-3.76624	2.85198	3.48566
H	-5.12531	2.09599	2.64763
H	-3.79364	1.09890	3.24218
C	-3.68381	3.61266	0.87408
H	-4.75407	3.66003	0.66436
H	-3.45599	4.40387	1.59343
H	-3.14808	3.83035	-0.04886
C	-1.28885	-2.56023	0.64708
H	-0.96319	-3.54610	0.98852
H	-0.59508	-2.26223	-0.13592
H	-1.19205	-1.87467	1.49266
C	-2.83681	-3.58653	-1.02519
H	-2.26780	-3.20283	-1.87083
H	-2.43314	-4.56738	-0.76023
H	-3.87150	-3.73099	-1.34382
C	-3.50285	-3.30144	1.35766
H	-3.47532	-2.66270	2.24400
H	-4.54766	-3.50188	1.11821
H	-3.02775	-4.25316	1.60975
C	-7.81325	1.01815	-0.42148
H	-7.83970	1.36068	0.61615
H	-7.34935	1.79768	-1.03087
H	-8.84785	0.90792	-0.75467
C	-7.78857	-1.34635	0.33095
H	-7.35048	-2.34096	0.22766
H	-7.72574	-1.05819	1.38317
H	-8.84556	-1.41922	0.06064
C	-7.18575	-0.76633	-2.02732
H	-6.70385	-0.04475	-2.69119
H	-6.70833	-1.73540	-2.18495
H	-8.23453	-0.85579	-2.32332
Si	1.19699	-0.25169	-1.98890
S	-1.11410	-0.80737	-2.35954
Cl	-1.56241	2.21532	-1.44969

R-P=C(Si(II)Cl)-P(S)MeR

R = H

P	1.05530	-0.00794	1.07853
C	0.59421	-0.32648	-0.48245
P	-1.00307	-0.14272	-1.17651
S	-0.48629	0.34663	-3.04205
Cl	1.02407	-2.71260	-2.33466
Si	1.57823	-0.64039	-2.16206
H	-1.64786	-1.39437	-1.11868
H	2.45618	-0.25909	0.91202
C	-2.15501	0.97779	-0.36609
H	-3.09072	0.99945	-0.92839
H	-2.34578	0.62869	0.65156
H	-1.72137	1.97775	-0.34003

R = Me

P	1.00029	-0.01430	0.87539
C	0.50963	-0.19084	-0.69937
P	-1.14277	-0.11970	-1.27356
S	-0.79353	0.29313	-3.20902
Cl	1.37316	-2.40412	-2.67357
Si	1.41769	-0.25766	-2.43803
C	-2.20641	1.09376	-0.46692
H	-1.75607	2.08105	-0.56779
H	-3.18765	1.09155	-0.94619
H	-2.31349	0.84473	0.59131
C	2.81854	-0.25457	0.78875
H	3.22290	0.03635	-0.18411
H	3.30322	0.31738	1.58194
H	3.04000	-1.31329	0.95322
C	-1.94721	-1.71726	-1.05788
H	-1.30453	-2.47679	-1.50491
H	-2.08067	-1.92235	0.00668
H	-2.91459	-1.70918	-1.56383

R = *t*-Bu

P	0.63919	0.14610	0.54238
C	0.47687	-0.28087	-1.05998

P	-1.13328	-0.43848	-1.77469
Cl	2.12696	-2.50805	-2.56288
Si	1.53822	-0.44095	-2.70840
C	-2.14198	1.04234	-1.53006
H	-3.09766	0.94202	-2.04655
H	-2.30564	1.21539	-0.46526
H	-1.59409	1.88428	-1.95345
C	-2.11307	-1.88483	-1.14723
C	-2.57674	-1.61356	0.28193
H	-3.29162	-0.78983	0.33594
H	-3.07712	-2.50893	0.66214
H	-1.74086	-1.39191	0.94987
C	-3.31597	-2.07367	-2.07121
H	-3.00567	-2.24080	-3.10455
H	-3.87500	-2.95355	-1.74023
H	-4.00034	-1.22244	-2.04449
C	-1.22587	-3.12547	-1.19126
H	-0.84798	-3.31664	-2.19676
H	-0.36756	-3.03471	-0.52413
H	-1.81749	-3.98956	-0.87512
C	2.45406	0.10105	1.00007
C	2.88397	-1.36295	1.09871
H	2.25370	-1.92314	1.79599
H	2.84634	-1.86066	0.12856
H	3.91262	-1.41402	1.47328
C	3.32842	0.86067	0.01202
H	4.36531	0.85936	0.36649
H	3.32298	0.41068	-0.98395
H	3.01299	1.90193	-0.09199
C	2.54172	0.75808	2.37882
H	3.57406	0.72125	2.74006
H	2.23483	1.80723	2.34793
H	1.91142	0.24658	3.11157
S	-0.62023	-0.66947	-3.71038

R = Ph

P	0.96901	0.21906	0.32572
C	0.46498	-0.27089	-1.18373
P	-1.21720	-0.21652	-1.67790
C	2.75204	-0.05620	0.35979
C	3.42096	-1.00987	-0.41308
C	3.48834	0.74641	1.23700
C	4.79831	-1.13034	-0.32982
C	4.86563	0.62757	1.31240
C	5.52137	-0.30950	0.52528
H	5.30879	-1.87272	-0.93243
H	5.42859	1.26162	1.98774
C	-2.09778	-1.71032	-1.16341
C	-2.57878	-2.61160	-2.10444
C	-2.28796	-1.95216	0.19639
C	-3.26159	-3.74474	-1.68680
C	-2.96978	-3.08436	0.60715
C	-3.45923	-3.97976	-0.33537
H	-3.63261	-4.44883	-2.42233
H	-3.11395	-3.27280	1.66466
S	-0.98924	-0.09298	-3.66730
Cl	1.22819	-2.70092	-3.02659
Si	1.27633	-0.54442	-2.94771
C	-2.16737	1.16089	-0.99996
H	-1.66967	2.09290	-1.26853
H	-3.17216	1.14043	-1.42605
H	-2.22992	1.07666	0.08670
H	-1.88786	-1.26895	0.93903
H	-2.40481	-2.42634	-3.15801
H	-3.98953	-4.86810	-0.01162
H	2.97311	1.47551	1.85519
H	6.59929	-0.40840	0.58681
H	2.86084	-1.68515	-1.05029

R = Mes

P	1.12944	0.09831	0.71639
C	0.62537	-0.57564	-0.71821
P	-1.02936	-0.48446	-1.33583
C	2.95374	0.05521	0.61006
C	3.66889	-1.10343	0.94232
C	3.63570	1.24294	0.30658
C	5.05746	-1.05333	0.95060
C	5.02342	1.24589	0.32381
C	5.75409	0.10702	0.63949
H	5.61074	-1.95107	1.21124
H	5.54867	2.16569	0.08267
C	-2.13802	-1.92307	-1.17875
C	-3.27727	-1.95767	-2.01080
C	-1.92894	-2.95378	-0.24113
C	-4.16907	-3.01612	-1.88379
C	-2.85389	-3.98782	-0.16774
C	-3.97705	-4.04638	-0.97809
H	-5.04422	-3.03060	-2.52630
H	-2.68332	-4.77699	0.55788
S	-0.47303	-0.10286	-3.24380
Cl	1.32253	-3.00580	-2.49312
Si	1.63338	-0.85908	-2.37714
C	-1.93112	0.87524	-0.54780
H	-1.39053	1.80516	-0.72517
H	-2.94815	0.95217	-0.92965
H	-1.97026	0.67518	0.52612
C	2.89019	2.48701	-0.07209
H	2.44042	2.38309	-1.06461
H	2.07711	2.70274	0.62952
H	3.55453	3.35198	-0.09299
C	2.97259	-2.38981	1.27079
H	2.12437	-2.23312	1.94438
H	2.58316	-2.86580	0.36569
H	3.65736	-3.09119	1.74967
C	7.25190	0.12492	0.62113
H	7.66808	-0.61824	1.30360
H	7.62853	-0.10327	-0.38096
H	7.64261	1.10527	0.90055
C	-0.75917	-3.01841	0.69202
H	0.18090	-3.05711	0.14037
H	-0.71216	-2.15506	1.35896
H	-0.83001	-3.91413	1.30977
C	-3.59508	-0.94405	-3.07328
H	-3.38248	0.08585	-2.78805
H	-3.01182	-1.13798	-3.97659
H	-4.65317	-1.00367	-3.33197
C	-4.93366	-5.19426	-0.89285
H	-4.59401	-6.02387	-1.52070
H	-5.01379	-5.57103	0.12848
H	-5.92983	-4.91002	-1.23567

R = Mes*

P	1.10170	0.73783	1.12028
C	0.41127	0.28526	-0.32971
P	-1.30168	0.33782	-0.80787
Cl	1.85265	-2.33518	-1.45518
C	-2.87571	0.07743	0.12307
C	-3.48791	-1.20584	0.03765
C	-3.70357	1.19162	0.42289
C	-4.85971	-1.25110	-0.16936
C	-5.07258	1.06502	0.20351

C	-5.66659	-0.12144	-0.19636
H	-5.31878	-2.22342	-0.29894
H	-5.69574	1.93267	0.37184
C	2.90718	0.53563	0.80039
C	3.58645	-0.59353	1.31491
C	3.60348	1.44537	-0.03679
C	4.81380	-0.93128	0.75092
C	4.82216	1.03389	-0.56156
C	5.41454	-0.18053	-0.24554
H	5.31232	-1.82290	1.09550
H	5.34104	1.69362	-1.24272
C	3.08740	-1.40973	2.52469
C	3.16928	2.90591	-0.30422
C	6.70050	-0.60815	-0.94514
C	-3.26938	2.46995	1.17169
C	-2.81741	-2.55465	0.36816
C	-7.14380	-0.23629	-0.55122
C	4.39594	3.78597	-0.58962
H	4.08214	4.83262	-0.60345
H	5.16621	3.67205	0.17665
H	4.84317	3.57814	-1.56344
C	2.50613	3.51425	0.93461
H	1.55124	3.04078	1.18714
H	3.15477	3.43653	1.81045
H	2.29271	4.57193	0.75648
C	2.23409	3.04472	-1.50779
H	2.66752	2.58652	-2.39877
H	1.27367	2.56674	-1.32611
H	2.05666	4.10475	-1.71574
C	7.80564	0.42140	-0.68295
H	8.72723	0.12250	-1.19067
H	7.53180	1.41333	-1.04806
H	8.01746	0.50374	0.38617
C	7.19348	-1.97168	-0.46818
H	8.09560	-2.24632	-1.02050
H	7.44855	-1.96516	0.59479
H	6.44849	-2.75244	-0.63956
C	6.43355	-0.69579	-2.45288
H	5.64558	-1.42148	-2.66710
H	6.11909	0.26610	-2.86293
H	7.34093	-1.00555	-2.97948
C	4.12965	-2.42708	2.99755
H	5.08361	-1.95640	3.24666
H	3.75527	-2.91456	3.90085
H	4.30879	-3.20973	2.25667
C	2.84535	-0.45341	3.70152
H	3.75810	0.09365	3.95053
H	2.06154	0.27663	3.48960
H	2.53582	-1.02106	4.58391
C	1.81186	-2.19879	2.23030
H	0.96102	-1.54722	2.02638
H	1.93726	-2.85000	1.36421
H	1.54924	-2.81021	3.09918
C	-1.80665	2.48180	1.59949
H	-1.54909	1.57752	2.15389
H	-1.09471	2.58435	0.78343
H	-1.63435	3.33166	2.26471
C	-4.06720	2.46362	2.49353
H	-3.76161	3.31822	3.10338
H	-5.14274	2.53498	2.33059
H	-3.87061	1.55197	3.06224
C	-3.62115	3.75939	0.42347
H	-4.69004	3.82090	0.21020
H	-3.35980	4.62375	1.03979
H	-3.09231	3.85403	-0.52483
C	-1.36020	-2.44124	0.79559
H	-1.03828	-3.39800	1.21434
H	-0.68224	-2.21764	-0.02543
H	-1.24022	-1.68589	1.57605
C	-2.95156	-3.58657	-0.75361
H	-2.39846	-3.28051	-1.64044
H	-2.54907	-4.54479	-0.41424
H	-3.99439	-3.74886	-1.03542
C	-3.57070	-3.08615	1.60686
H	-3.51439	-2.37544	2.43528
H	-4.62355	-3.28663	1.40465
H	-3.10715	-4.02189	1.93024
C	-7.84861	1.11749	-0.53832
H	-7.85707	1.56511	0.45885
H	-7.38199	1.82357	-1.22984
H	-8.88887	0.98974	-0.84675
C	-7.84817	-1.15721	0.45223
H	-7.42453	-2.16331	0.44744
H	-7.76854	-0.76339	1.46845
H	-8.90927	-1.24211	0.20210
C	-7.26719	-0.83143	-1.95908
H	-6.78574	-0.18831	-2.69967
H	-6.80383	-1.81787	-2.02266
H	-8.32083	-0.93648	-2.23190
Si	1.12649	-0.39801	-2.04988
S	-1.16725	-0.98171	-2.34218
C	-1.53890	1.90868	-1.69529
H	-1.45654	2.79575	-1.07529
H	-0.75904	1.92633	-2.45878
H	-2.51194	1.87714	-2.18736

E = Ge(II)

R-P=C(Ge(II)Cl)-P(O)ClR

R= H

P	0.29435	0.56617	0.60842
C	0.22926	0.36855	-1.03311
P	-1.12731	-0.01182	-2.04410
Ge	1.31552	0.82146	-2.78519
Cl	1.83806	-1.31483	-3.21569
O	-0.70661	0.61857	-3.36114
H	1.67818	0.92075	0.70701
H	-1.34825	-1.39419	-2.14827
Cl	-2.93656	0.61830	-1.44062

R = Me

P	0.55866	0.25480	0.66466
C	0.21676	0.15235	-0.94849
P	-1.31956	-0.01921	-1.73437
Ge	1.06971	0.52876	-2.83525
Cl	1.40028	-1.63920	-3.35883
O	-0.98299	0.50475	-3.12946
C	-1.95133	-1.68883	-1.76625
H	-2.20074	-2.01365	-0.75559
H	-2.83784	-1.72212	-2.40166
H	-1.16423	-2.31821	-2.18758
C	2.38914	0.39865	0.71760
H	2.80502	-0.59767	0.89858
H	2.80907	0.79010	-0.20974
H	2.68031	1.03306	1.55732
Cl	-2.83087	1.05683	-0.92521

R = *t*-Bu

P	0.76669	-0.23326	0.59292
C	0.15162	0.09962	-0.90733

P	-1.51485	-0.12009	-1.38818
Ge	0.53203	0.98241	-2.79196
Cl	1.31611	-0.87798	-3.79327
O	-1.47478	0.44185	-2.80824
C	-2.24873	-1.79783	-1.33161
C	2.60300	0.11753	0.56529
Cl	-2.76130	1.02082	-0.25510
C	3.10727	0.82901	-0.67617
H	2.61391	1.79383	-0.81922
H	4.18159	1.02209	-0.58192
H	2.96546	0.22179	-1.57356
C	2.87968	0.95689	1.81564
H	2.50027	0.47470	2.72084
H	3.95943	1.09152	1.93594
H	2.42417	1.94804	1.74489
C	3.27642	-1.25137	0.70850
H	4.35578	-1.11488	0.83440
H	2.90849	-1.79811	1.58164
H	3.11583	-1.87040	-0.17731
C	-2.36263	-2.27996	0.11121
H	-2.74374	-3.30503	0.10553
H	-1.39402	-2.28811	0.61701
H	-3.05100	-1.66626	0.69397
C	-1.32003	-2.71533	-2.13080
H	-1.20225	-2.37978	-3.16164
H	-0.32655	-2.78163	-1.68469
H	-1.75791	-3.71746	-2.14124
C	-3.62203	-1.73182	-2.00121
H	-4.03486	-2.74369	-2.04308
H	-4.31891	-1.10559	-1.44180
H	-3.55141	-1.35089	-3.02157

R = Ph

P	0.96480	-0.20178	1.05995
C	0.24416	-0.02245	-0.42218
P	-1.44902	-0.03100	-0.79238
C	2.73767	-0.10470	0.72188
C	3.55351	0.29590	1.78384
C	3.32673	-0.42806	-0.50417
C	4.92316	0.41430	1.61347
C	4.69694	-0.31946	-0.66776
C	5.49454	0.10894	0.38636
H	5.54615	0.73907	2.43888
H	5.14644	-0.57701	-1.61992
Ge	0.60894	0.85576	-2.29204
Cl	0.82720	-1.03784	-3.49713
O	-1.46513	0.74764	-2.10474
H	2.71454	-0.80090	-1.31867
H	6.56718	0.19293	0.25304
H	3.10537	0.52576	2.74583
Cl	-2.59073	0.89447	0.60369
C	-2.19443	-1.64606	-0.96669
C	-2.47437	-2.11028	-2.24838
C	-2.44976	-2.44403	0.14705
C	-3.01966	-3.37504	-2.41054
H	-2.25854	-1.48528	-3.10575
C	-2.98955	-3.70550	-0.02552
H	-2.23143	-2.07911	1.14472
C	-3.27558	-4.16948	-1.30401
H	-3.24033	-3.73970	-3.40675
H	-3.19037	-4.32887	0.83779
H	-3.70000	-5.15835	-1.43545

R = Mes

P	0.84382	0.10389	1.05317
C	0.41343	-0.51604	-0.42272
P	-1.10285	-0.47514	-1.29025
C	2.68673	0.08341	0.93951
C	3.42585	-1.10253	1.17981
C	3.35259	1.25258	0.49715
C	4.74922	-1.13760	0.75935
C	4.68382	1.13176	0.09981
C	5.38772	-0.05797	0.16604
H	5.31332	-2.04762	0.90484
H	5.18959	2.00533	-0.27813
C	2.72498	2.66591	0.48672
C	2.88140	-2.33045	1.93939
C	6.82260	-0.20096	-0.33244
C	2.07444	-3.25972	1.02693
H	1.18616	-2.78090	0.61941
H	2.67677	-3.59380	0.18004
H	1.75982	-4.14417	1.58952
C	2.03187	-1.89195	3.13489
H	2.59909	-1.24064	3.80458
H	1.12088	-1.35686	2.84751
H	1.70622	-2.76976	3.69955
C	4.02411	-3.16821	2.52921
H	4.60030	-3.68842	1.76151
H	4.70842	-2.56377	3.12966
H	3.59599	-3.93689	3.17680
C	1.70095	2.85526	-0.63937
H	0.79515	2.26811	-0.49245
H	1.40090	3.90602	-0.68635
H	2.12827	2.58243	-1.60730
C	2.07336	2.96183	1.84356
H	1.23567	2.29787	2.06994
H	2.80218	2.87007	2.65256
H	1.68130	3.98258	1.84956
C	3.78851	3.75225	0.28614
H	4.59118	3.68452	1.02410
H	4.22925	3.72554	-0.71298
H	3.31247	4.72872	0.40025
C	7.34974	1.08561	-0.96236
H	7.38538	1.90819	-0.24341
H	8.36757	0.92267	-1.32492
H	6.74130	1.39864	-1.81454
C	6.87480	-1.30906	-1.39114
H	6.54684	-2.26940	-0.98848
H	6.23259	-1.06768	-2.24148
H	7.89751	-1.43147	-1.75883
C	7.74047	-0.57382	0.83772
H	7.44344	-1.51622	1.30220
H	8.77075	-0.68480	0.48791
H	7.72441	0.20034	1.60897
C	-2.23563	-1.87453	-1.13708
C	-2.62662	-2.65782	-2.25634
C	-2.72725	-2.18330	0.14767
C	-3.46443	-3.73935	-2.02429
C	-3.56750	-3.27887	0.30028
C	-3.94347	-4.07520	-0.76531
H	-3.75852	-4.34717	-2.87009
H	-3.94401	-3.51735	1.28944
C	-2.25256	-2.35347	-3.69570
C	-3.18654	-1.28013	-4.25840
C	-2.23947	-3.57455	-4.60766
H	-1.24395	-1.95174	-3.70545
H	-3.14879	-0.36029	-3.67211
H	-2.90124	-1.03244	-5.28391
H	-4.22180	-1.63426	-4.26888
H	-1.63508	-4.38258	-4.19012
H	-3.24155	-3.95956	-4.81691

H	-1.80132	-3.29398	-5.56810
C	-2.42733	-1.37685	1.39494
C	-1.79191	-2.23404	2.48660
C	-3.68491	-0.68008	1.91253
H	-1.70905	-0.59005	1.15615
H	-0.90185	-2.74956	2.12202
H	-1.50306	-1.60797	3.33433
H	-2.48847	-2.98981	2.85712
H	-4.12955	-0.04099	1.14808
H	-4.43785	-1.40861	2.22358
H	-3.44136	-0.05908	2.77825
C	-4.84905	-5.26538	-0.56215
C	-4.14666	-6.57082	-0.92864
C	-6.16085	-5.11399	-1.32867
H	-5.08932	-5.30347	0.50637
H	-3.22123	-6.69702	-0.36291
H	-4.79528	-7.42536	-0.71964
H	-3.89363	-6.59721	-1.99172
H	-6.68002	-4.19400	-1.05160
H	-5.98701	-5.08799	-2.40755
H	-6.82431	-5.95712	-1.12014
Ge	1.44748	-0.62105	-2.23705
Cl	1.29913	-2.85315	-2.56725
O	-0.54350	-0.23626	-2.69338
Cl	-2.24388	1.16925	-0.85645

R = Mes*

P	1.11140	0.90602	1.34322
C	0.39006	0.41172	-0.06686
P	-1.27207	0.27398	-0.60259
Cl	1.60635	-2.51048	-1.13673
O	-1.06922	-0.76619	-1.70456
C	-2.88804	-0.00358	0.19870
C	-3.46312	-1.29578	0.01482
C	-3.73935	1.07787	0.54177
C	-4.82968	-1.35621	-0.21287
C	-5.10379	0.92471	0.31734
C	-5.66475	-0.24798	-0.15996
H	-5.26362	-2.32396	-0.42766
H	-5.74889	1.76515	0.53163
C	2.89031	0.64466	0.94596
C	3.55413	-0.47636	1.49778
C	3.55106	1.45145	-0.01140
C	4.72962	-0.90260	0.88600
C	4.71415	0.95007	-0.58429
C	5.28930	-0.25345	-0.20316
H	5.21730	-1.78660	1.26400
H	5.20077	1.53061	-1.35659
C	3.08276	-1.19940	2.77586
C	3.14277	2.89173	-0.39330
C	6.51525	-0.78169	-0.94147
C	-3.34103	2.35156	1.31371
C	-2.74104	-2.64525	0.20011
C	-7.13774	-0.37707	-0.52710
C	4.40636	3.74745	-0.58883
H	4.11353	4.79391	-0.70437
H	5.07833	3.67353	0.26939
H	4.96526	3.47588	-1.48526
C	2.34837	3.58521	0.71381
H	1.34425	3.16978	0.84817
H	2.86789	3.53661	1.67387
H	2.20514	4.63812	0.45599
C	2.34346	2.93900	-1.69831
H	2.87731	2.43163	-2.50503
H	1.36609	2.46885	-1.59093
H	2.18346	3.97888	-1.99945
C	7.66466	0.22660	-0.83025
H	8.54386	-0.14388	-1.36505
H	7.39872	1.19513	-1.25859
H	7.94423	0.38694	0.21410
C	6.99574	-2.11910	-0.38432
H	7.85425	-2.46722	-0.96390
H	7.31510	-2.03660	0.65784
H	6.21948	-2.88546	-0.44816
C	6.15337	-0.98079	-2.41844
H	5.33399	-1.69548	-2.52558
H	5.84334	-0.04473	-2.88760
H	7.01718	-1.36229	-2.97039
C	4.10690	-2.23172	3.25573
H	5.09068	-1.78844	3.42690
H	3.76257	-2.64929	4.20470
H	4.21468	-3.06290	2.55502
C	2.93389	-0.17180	3.90671
H	3.87956	0.34525	4.08742
H	2.17388	0.58117	3.68871
H	2.63894	-0.67698	4.83094
C	1.76490	-1.95441	2.58248
H	0.91807	-1.28367	2.41950
H	1.81584	-2.62985	1.72732
H	1.54130	-2.53668	3.48155
C	-1.86575	2.43750	1.67728
H	-1.51805	1.52380	2.16435
H	-1.22657	2.64990	0.82227
H	-1.71826	3.25413	2.38851
C	-4.09549	2.27280	2.65725
H	-3.82648	3.13557	3.27273
H	-5.17811	2.27695	2.52746
H	-3.82434	1.36675	3.20451
C	-3.76292	3.63748	0.59737
H	-4.83613	3.66305	0.39999
H	-3.52448	4.49920	1.22680
H	-3.24108	3.75639	-0.35176
C	-1.40201	-2.49996	0.91763
H	-1.01941	-3.49239	1.16785
H	-0.62772	-2.03308	0.31515
H	-1.51801	-1.94308	1.85147
C	-2.57558	-3.40811	-1.11700
H	-1.92694	-2.87476	-1.80774
H	-2.13832	-4.39041	-0.91848
H	-3.54422	-3.56451	-1.59932
C	-3.60883	-3.50599	1.14112
H	-3.82560	-2.98178	2.07516
H	-4.55524	-3.81026	0.69312
H	-3.05932	-4.41879	1.38394
C	-7.87875	0.95328	-0.42369
H	-7.89884	1.33186	0.60149
H	-7.43151	1.71674	-1.06511
H	-8.91523	0.81831	-0.74168
C	-7.81786	-1.38353	0.40870
H	-7.36478	-2.37426	0.33875
H	-7.75261	-1.05683	1.44946
H	-8.87538	-1.48150	0.14834
C	-7.24247	-0.87667	-1.97315
H	-6.77494	-0.17230	-2.66525
H	-6.75539	-1.84506	-2.10271
H	-8.29262	-0.98826	-2.25660
Ge	0.99697	-0.47808	-1.89424
Cl	-1.61783	2.01492	-1.64698

R-P=C(Ge(II)Cl)-P(O)MeR

R= H

P	0.42553	0.80794	0.52687
C	0.27561	0.49788	-1.09096
P	-1.16129	0.18374	-2.03701
Ge	1.35703	0.63861	-2.89398
Cl	1.65054	-1.58609	-3.04703
O	-0.65133	0.57819	-3.42832
C	-2.67034	1.03509	-1.58428
H	-2.49104	2.11083	-1.58887
H	-3.45664	0.79212	-2.30183
H	-2.97833	0.71902	-0.58472
H	1.83500	1.06083	0.56413
H	-1.49154	-1.18469	-1.96574

R = Me

P	0.50824	0.33422	0.55788
C	0.21045	0.19946	-1.06025
P	-1.32547	0.03277	-1.88083
Ge	1.12626	0.44209	-2.93744
Cl	1.46983	-1.76914	-3.27026
O	-0.88610	0.40847	-3.31029
C	-2.64057	1.11271	-1.30574
H	-2.29502	2.14581	-1.35222
H	-3.51944	0.99613	-1.94347
H	-2.90167	0.86488	-0.27476
C	-1.95287	-1.64512	-1.80514
H	-2.23670	-1.89575	-0.78116
H	-2.81836	-1.74211	-2.46426
H	-1.15879	-2.31251	-2.14363
C	2.33665	0.49092	0.67556
H	2.74774	-0.48402	0.95542
H	2.79091	0.80790	-0.26379
H	2.59287	1.19424	1.47104

R = *t*-Bu

P	0.69725	-0.19789	0.50722
C	0.16785	0.10469	-1.02985
P	-1.49740	-0.04862	-1.58118
Ge	0.66012	0.91305	-2.92316
Cl	1.47173	-0.99240	-3.82575
O	-1.32622	0.44019	-3.03630
C	-2.22935	-1.73207	-1.53304
C	2.54264	0.11116	0.58081
C	3.13205	0.76262	-0.65614
H	2.67234	1.73261	-0.86180
H	4.20487	0.93191	-0.51065
H	3.01989	0.12700	-1.53774
C	2.76830	0.99228	1.81174
H	2.32497	0.55487	2.71080
H	3.84221	1.10798	1.99104
H	2.34207	1.98985	1.67631
C	3.17680	-1.26361	0.81313
H	4.25058	-1.14578	0.99461
H	2.74737	-1.76873	1.68329
H	3.05104	-1.91272	-0.05665
C	-2.49683	-2.14143	-0.08618
H	-2.85679	-3.17424	-0.07183
H	-1.59096	-2.09862	0.52445
H	-3.26188	-1.52056	0.38524
C	-1.25128	-2.70737	-2.18586
H	-1.01525	-2.42036	-3.21117
H	-0.31182	-2.76909	-1.63458
H	-1.70665	-3.70204	-2.20052
C	-3.53129	-1.69476	-2.33704
H	-3.94361	-2.70675	-2.38667
H	-4.28738	-1.05586	-1.87476
H	-3.35915	-1.34839	-3.35779
C	-2.59737	1.06130	-0.68364
H	-2.18933	2.06902	-0.77358
H	-3.59915	1.04436	-1.11566
H	-2.64107	0.78952	0.37175

R = Ph

P	0.85677	-0.02478	0.77304
C	0.24094	0.01495	-0.76362
P	-1.44603	0.00864	-1.22452
C	2.65477	0.02984	0.56724
C	3.39738	0.53772	1.63608
C	3.32567	-0.42930	-0.56938
C	4.77777	0.62633	1.55582
C	4.70596	-0.34937	-0.64248
C	5.43223	0.18485	0.41468
H	5.34414	1.03431	2.38524
H	5.21873	-0.71198	-1.52607
Ge	0.72951	0.61912	-2.71241
Cl	0.99670	-1.44284	-3.59928
O	-1.32683	0.56913	-2.65393
H	2.76733	-0.88151	-1.38243
H	6.51292	0.24569	0.35250
H	2.88444	0.87417	2.53218
C	-2.17241	-1.63412	-1.23901
C	-2.48592	-2.22283	-2.45973
C	-2.41425	-2.31920	-0.04950
C	-3.05269	-3.48849	-2.48651
H	-2.27287	-1.69002	-3.37840
C	-2.97842	-3.58260	-0.08321
H	-2.14772	-1.87704	0.90538
C	-3.30025	-4.16559	-1.30242
H	-3.29404	-3.94854	-3.43759
H	-3.16316	-4.11662	0.84161
H	-3.73987	-5.15626	-1.32705
C	-2.51795	1.03392	-0.21026
H	-2.11230	2.04614	-0.18629
H	-3.52087	1.04662	-0.64109
H	-2.56470	0.64005	0.80680

R = Mes

P	1.06709	-0.48152	1.16641
C	0.27967	-0.21897	-0.26477
P	-1.41603	-0.11249	-0.72644
C	2.81144	-0.11343	0.75178
C	3.36803	1.10499	1.16451
C	3.61058	-1.07771	0.11912
C	4.71173	1.35225	0.91397
C	4.95031	-0.78786	-0.10831
C	5.51895	0.42117	0.27327
H	5.13927	2.29926	1.23113
H	5.56999	-1.53522	-0.59561
Ge	0.81069	0.81601	-1.99622
Cl	0.96005	-1.01004	-3.33603
O	-1.23573	0.85355	-1.91588
C	-2.20493	-1.67504	-1.18876
C	-2.83492	-1.83126	-2.44367
C	-2.22667	-2.73286	-0.26034
C	-3.45134	-3.04474	-2.72356
C	-2.86296	-3.92093	-0.59796
C	-3.47446	-4.10348	-1.82714
H	-3.93817	-3.16005	-3.68707

H	-2.87687	-4.72675	0.12985
C	-2.50341	0.69251	0.46599
H	-2.03160	1.62096	0.79095
H	-3.44339	0.92250	-0.04079
H	-2.71082	0.06250	1.33009
C	2.53072	2.14954	1.84120
H	1.84856	2.62799	1.13154
H	1.91417	1.72109	2.63836
H	3.15543	2.92846	2.28068
C	3.05370	-2.40225	-0.31070
H	2.48459	-2.87872	0.49417
H	2.37706	-2.29100	-1.16271
H	3.85558	-3.08216	-0.60170
C	6.95837	0.71873	-0.01679
H	7.56689	-0.18707	0.01526
H	7.07051	1.15134	-1.01600
H	7.36963	1.43429	0.69750
C	-2.89038	-0.77982	-3.51441
H	-3.27106	0.17408	-3.14975
H	-1.89723	-0.58395	-3.92137
H	-3.53571	-1.12252	-4.32402
C	-1.56352	-2.67999	1.08444
H	-0.51256	-2.97159	1.00763
H	-1.57610	-1.69625	1.55260
H	-2.04964	-3.37565	1.77013
C	-4.12440	-5.40293	-2.18709
H	-3.44552	-6.01892	-2.78494
H	-4.39203	-5.97574	-1.29784
H	-5.02666	-5.24529	-2.78126

R = Mes*

P	1.09169	1.08510	1.19825
C	0.34967	0.46462	-0.14751
P	-1.32367	0.34806	-0.71278
Cl	1.53612	-2.56875	-1.00209
O	-1.09906	-0.80982	-1.71793
C	-2.92453	0.07361	0.14749
C	-3.50850	-1.22123	0.04219
C	-3.77999	1.17208	0.42238
C	-4.87666	-1.29410	-0.18188
C	-5.14608	1.01335	0.20663
C	-5.71131	-0.18510	-0.19672
H	-5.31089	-2.27299	-0.33860
H	-5.78958	1.86607	0.37375
C	2.87082	0.74349	0.84486
C	3.49758	-0.34750	1.49036
C	3.57616	1.46991	-0.14704
C	4.67828	-0.84060	0.93995
C	4.74700	0.91169	-0.64577
C	5.28345	-0.27685	-0.17061
H	5.13428	-1.70801	1.38912
H	5.27261	1.43004	-1.43568
C	2.99032	-0.96424	2.81079
C	3.19786	2.88702	-0.63609
C	6.51741	-0.87732	-0.83666
C	-3.37694	2.48399	1.12690
C	-2.78862	-2.55828	0.30336
C	-7.18638	-0.33498	-0.54906
C	4.45430	3.65564	-1.07382
H	4.18549	4.69981	-1.25141
H	5.23285	3.63036	-0.30793
H	4.87528	3.27564	-2.00644
C	2.57865	3.71644	0.49151
H	1.60581	3.33686	0.82155
H	3.23415	3.75123	1.36491
H	2.41057	4.74029	0.14595
C	2.24621	2.86461	-1.83590
H	2.64870	2.25210	-2.64578
H	1.27142	2.46414	-1.56263
H	2.10445	3.88147	-2.21529
C	7.68605	0.11173	-0.75782
H	8.57121	-0.31111	-1.24167
H	7.45402	1.05547	-1.25568
H	7.94007	0.33415	0.28169
C	6.95186	-2.18506	-0.18017
H	7.81765	-2.58870	-0.71097
H	7.24428	-2.04091	0.86319
H	6.16005	-2.93718	-0.21606
C	6.19296	-1.16536	-2.30770
H	5.35999	-1.86722	-2.39238
H	5.91833	-0.25541	-2.84537
H	7.06271	-1.60105	-2.80794
C	3.98461	-1.97896	3.38313
H	4.97552	-1.54525	3.53702
H	3.61672	-2.31643	4.35489
H	4.08385	-2.86278	2.74864
C	2.85055	0.14831	3.85963
H	3.80550	0.65648	4.01552
H	2.11120	0.89936	3.57415
H	2.53171	-0.27989	4.81440
C	1.65874	-1.70396	2.65745
H	0.82820	-1.03027	2.43831
H	1.70356	-2.43921	1.85275
H	1.41667	-2.21727	3.59309
C	-1.90401	2.57188	1.50267
H	-1.57893	1.68605	2.05179
H	-1.23364	2.71033	0.65631
H	-1.74981	3.43230	2.15852
C	-4.13802	2.48863	2.46896
H	-3.86399	3.38081	3.03884
H	-5.21970	2.49538	2.33242
H	-3.87836	1.61079	3.06506
C	-3.79143	3.73443	0.34503
H	-4.86780	3.76303	0.16645
H	-3.53003	4.62869	0.91717
H	-3.29720	3.80258	-0.62490
C	-1.45345	-2.36656	1.01513
H	-1.05381	-3.34241	1.30116
H	-0.69027	-1.90513	0.39569
H	-1.58096	-1.77709	1.92723
C	-2.61906	-3.39248	-0.96929
H	-1.97068	-2.89527	-1.68647
H	-2.17939	-4.36115	-0.71630
H	-3.58727	-3.57823	-1.44261
C	-3.65738	-3.36799	1.28745
H	-3.88377	-2.79183	2.18810
H	-4.59880	-3.70520	0.85213
H	-3.10331	-4.26109	1.58682
C	-7.92576	1.00009	-0.52450
H	-7.94027	1.44135	0.47541
H	-7.48104	1.72225	-1.21392
H	-8.96407	0.84753	-0.82839
C	-7.86354	-1.28030	0.45041
H	-7.41129	-2.27376	0.44013
H	-7.79239	-0.88965	1.46849
H	-8.92252	-1.39321	0.20207
C	-7.30052	-0.92323	-1.96048
H	-6.83938	-0.26228	-2.69841
H	-6.81232	-1.89695	-2.03330
H	-8.35225	-1.05373	-2.22986
Ge	0.93510	-0.58619	-1.89034
C	-1.64986	1.77733	-1.77831

H	-2.58594	1.59086	-2.30778
H	-1.70044	2.73244	-1.26583
H	-0.83454	1.80223	-2.50506

R-P=C(Ge(II)Cl)-P(S)ClR

R= H

P	0.18249	0.84541	0.54154
C	0.17159	0.39415	-1.05207
P	-1.17688	-0.12328	-2.01041
Ge	1.47816	0.64897	-2.66504
S	-0.82260	0.74149	-3.75590
Cl	1.85163	-1.55080	-2.95280
H	1.56470	1.21442	0.61550
Cl	-3.02618	0.19711	-1.24832
H	-1.17824	-1.52544	-2.08391

R = Me

P	0.51829	0.36678	0.61726
C	0.18402	0.16405	-0.99175
P	-1.38282	-0.00282	-1.71547
Ge	1.25233	0.41866	-2.76702
S	-1.10738	0.81841	-3.50957
Cl	1.43021	-1.77866	-3.26651
C	-1.89567	-1.72109	-1.79709
H	-1.10135	-2.26898	-2.30896
H	-2.03689	-2.10544	-0.78582
H	-2.82642	-1.78647	-2.36201
C	2.35229	0.43538	0.68237
H	2.72165	-0.55644	0.96147
H	2.80523	0.72313	-0.26788
H	2.65892	1.13210	1.46528
Cl	-2.90605	0.86941	-0.67534

R = *t*-Bu

P	0.66956	-0.10529	0.57230
C	0.11335	0.18396	-0.96630
P	-1.58255	-0.02290	-1.36046
Ge	0.72381	1.10415	-2.74855
S	-1.75448	0.90799	-3.11457
Cl	1.43754	-0.75248	-3.82169
C	-2.19181	-1.77497	-1.40708
C	2.52747	0.09277	0.61783
Cl	-2.79295	0.85018	0.04185
C	2.81742	0.92611	1.86935
H	3.89782	0.96475	2.04140
H	2.45809	1.95281	1.76218
H	2.35194	0.49670	2.76077
C	3.06520	-1.32900	0.81614
H	4.14411	-1.28556	0.99967
H	2.60419	-1.82530	1.67519
H	2.89802	-1.94681	-0.06922
C	3.16270	0.73085	-0.60330
H	2.77980	1.73864	-0.78157
H	4.24489	0.81607	-0.45339
H	3.00977	0.12756	-1.50158
C	-2.11892	-2.39564	-0.01601
H	-2.44229	-3.43793	-0.08968
H	-1.10203	-2.39078	0.38191
H	-2.77095	-1.88830	0.69584
C	-3.63121	-1.75900	-1.91768
H	-3.97511	-2.79320	-2.00735
H	-4.30275	-1.23767	-1.23361
H	-3.70315	-1.29434	-2.90302
C	-1.29760	-2.54282	-2.37908
H	-1.31963	-2.11282	-3.38104
H	-0.25797	-2.56793	-2.05008
H	-1.66457	-3.57156	-2.43800

R = Ph

P	0.85068	0.02553	1.03352
C	0.18133	0.06215	-0.48649
P	-1.51466	0.06577	-0.84678
C	2.63404	-0.02257	0.73944
C	3.44737	0.54573	1.72394
C	3.23076	-0.61573	-0.37742
C	4.82430	0.56278	1.57506
C	4.60858	-0.60641	-0.51735
C	5.40479	-0.01105	0.45245
H	5.44604	1.01818	2.33725
H	5.06299	-1.07149	-1.38458
C	-2.16575	-1.60266	-1.03593
C	-2.57424	-2.05427	-2.28435
C	-2.21966	-2.44565	0.07300
C	-3.04790	-3.35078	-2.42086
C	-2.69156	-3.73726	-0.07254
C	-3.10705	-4.18920	-1.31939
H	-3.36540	-3.70510	-3.39435
H	-2.73544	-4.39445	0.78798
Ge	0.86082	0.68615	-2.35472
S	-1.61839	1.16217	-2.50212
Cl	0.88704	-1.32564	-3.38360
H	-3.47561	-5.20266	-1.43007
H	-2.51076	-1.39529	-3.14154
H	-1.89659	-2.09334	1.04652
H	2.99285	0.98613	2.60624
H	2.61879	-1.11937	-1.11870
H	6.48288	-0.00568	0.33831
Cl	-2.65577	0.83149	0.66556

R = Mes

P	0.93354	-0.14691	1.19103
C	0.19540	-0.11595	-0.29451
P	-1.49080	-0.02520	-0.73209
C	2.70094	0.03572	0.75876
C	3.33117	1.26029	1.02497
C	3.44273	-1.05108	0.26937
C	4.69053	1.38748	0.77209
C	4.80100	-0.87722	0.03465
C	5.44252	0.33259	0.27104
H	5.17505	2.33842	0.97420
H	5.37708	-1.71833	-0.34006
C	-2.20474	-1.62129	-1.21769
C	-2.86075	-1.82256	-2.45359
C	-2.08495	-2.69451	-0.30625
C	-3.32815	-3.09702	-2.74935
C	-2.57718	-3.94167	-0.66748
C	-3.18751	-4.17367	-1.88820
H	-3.83220	-3.24536	-3.69896
H	-2.48239	-4.75636	0.04378
Ge	1.01754	0.64863	-2.04513
S	-1.35878	1.43984	-2.08469
Cl	0.80354	-1.22313	-3.30916
C	2.81178	-2.38643	0.00517
H	2.22180	-2.72718	0.86197
H	2.14007	-2.35096	-0.85720
H	3.57517	-3.13942	-0.19459
C	2.55311	2.43339	1.54202
H	1.90885	2.85390	0.76353
H	1.90181	2.15301	2.37653
H	3.21974	3.22487	1.88697

C	6.90136	0.49904	-0.02523
H	7.05152	0.80729	-1.06472
H	7.35294	1.26255	0.61064
H	7.44665	-0.43550	0.11898
C	-1.45280	-2.60055	1.05041
H	-0.36790	-2.71291	0.98294
H	-1.65210	-1.65995	1.56114
H	-1.82840	-3.40488	1.68411
C	-3.11422	-0.77199	-3.49362
H	-3.60105	0.11406	-3.08653
H	-2.18496	-0.44387	-3.96171
H	-3.75919	-1.18614	-4.26927
C	-3.67315	-5.53647	-2.26873
H	-3.89611	-6.14147	-1.38850
H	-4.57021	-5.48125	-2.88804
H	-2.90952	-6.06550	-2.84746
Cl	-2.73151	0.61792	0.78131

R = Mes*

P	1.04478	0.72566	1.35970
C	0.38535	0.30895	-0.11085
P	-1.28886	0.28486	-0.62922
Cl	1.89127	-2.42350	-1.22625
C	-2.88835	0.01936	0.24012
C	-3.46869	-1.27577	0.09276
C	-3.74115	1.10451	0.57303
C	-4.83235	-1.33870	-0.15031
C	-5.10064	0.95631	0.31367
C	-5.65899	-0.22212	-0.15406
H	-5.27038	-2.31364	-0.32476
H	-5.74513	1.80366	0.50240
C	2.84283	0.57559	1.00175
C	3.55718	-0.52800	1.53159
C	3.47681	1.43522	0.07397
C	4.75496	-0.88267	0.92134
C	4.66421	0.99879	-0.50626
C	5.29071	-0.18470	-0.15180
H	5.27967	-1.75262	1.28188
H	5.12536	1.62444	-1.26092
C	3.10304	-1.31171	2.77834
C	3.08156	2.89483	-0.24229
C	6.53610	-0.64696	-0.90109
C	-3.35824	2.35179	1.39702
C	-2.78055	-2.62175	0.40516
C	-7.12224	-0.34753	-0.55804
C	4.27941	3.77405	0.16541
H	4.04353	4.82516	-0.02250
H	4.50154	3.65893	1.22936
H	5.18414	3.53118	-0.39220
C	1.88817	3.43527	0.54173
H	0.93756	3.03992	0.18532
H	1.97902	3.24216	1.61538
H	1.84466	4.52109	0.41622
C	2.80518	3.09904	-1.73422
H	3.64989	2.79044	-2.35280
H	1.93165	2.52985	-2.05809
H	2.61339	4.15716	-1.93468
C	7.64028	0.40965	-0.78277
H	8.53270	0.08345	-1.32438
H	7.33131	1.36968	-1.20133
H	7.91669	0.57186	0.26214
C	7.07843	-1.96874	-0.36392
H	7.95022	-2.26914	-0.95030
H	7.39650	-1.88659	0.67873
H	6.33760	-2.76849	-0.43757
C	6.17287	-0.84271	-2.37830
H	5.38340	-1.58970	-2.48888
H	5.81912	0.08523	-2.83252
H	7.04859	-1.17948	-2.94056
C	4.17491	-2.29638	3.25367
H	5.12834	-1.80229	3.45505
H	3.83709	-2.75666	4.18510
H	4.34324	-3.10227	2.53565
C	2.86772	-0.32846	3.93358
H	3.77647	0.23820	4.15124
H	2.06871	0.38391	3.71890
H	2.58344	-0.87714	4.83607
C	1.83647	-2.13328	2.52745
H	0.96138	-1.50280	2.35314
H	1.94912	-2.78221	1.65771
H	1.61663	-2.74862	3.40525
C	-1.89882	2.40216	1.82860
H	-1.59081	1.46870	2.30419
H	-1.21591	2.62205	1.01169
H	-1.77136	3.19776	2.56713
C	-4.16648	2.22847	2.70720
H	-3.90352	3.05750	3.36986
H	-5.24294	2.26219	2.53711
H	-3.93437	1.29375	3.22321
C	-3.74676	3.66952	0.72201
H	-4.80989	3.70357	0.47620
H	-3.54126	4.50000	1.40288
H	-3.18230	3.83587	-0.19442
C	-1.33573	-2.50086	0.87405
H	-1.01577	-3.46301	1.28201
H	-0.63454	-2.25845	0.07826
H	-1.24284	-1.76134	1.67344
C	-2.86755	-3.62729	-0.74447
H	-2.29180	-3.29360	-1.60669
H	-2.46292	-4.58850	-0.41656
H	-3.89881	-3.79493	-1.06274
C	-3.55699	-3.19618	1.61034
H	-3.53382	-2.50554	2.45706
H	-4.60079	-3.40683	1.37501
H	-3.08721	-4.13260	1.92257
C	-7.85133	0.99260	-0.51274
H	-7.89971	1.39827	0.50109
H	-7.37523	1.73414	-1.15903
H	-8.87873	0.86020	-0.85991
C	-7.83969	-1.32077	0.38494
H	-7.39814	-2.31867	0.35261
H	-7.79856	-0.96758	1.41825
H	-8.89089	-1.41308	0.09837
C	-7.19060	-0.88639	-1.99218
H	-6.69549	-0.20656	-2.68956
H	-6.70998	-1.86269	-2.08019
H	-8.23325	-0.99541	-2.30304
Ge	1.25622	-0.35766	-1.90388
S	-1.17236	-0.91016	-2.22535
Cl	-1.54897	2.16403	-1.45732

R-P=C(Ge(II)Cl)-P(S)MeR

R= H

P	0.32897	1.07023	0.55315
C	0.14659	0.46782	-0.97974
P	-1.36391	0.07867	-1.77700
Ge	1.37533	0.33335	-2.65217
S	-0.95068	0.61849	-3.65510
Cl	1.38815	-1.92294	-2.63541
H	1.74723	1.27230	0.51981
C	-2.87284	0.78533	-1.09496

H	-3.72466	0.46137	-1.69609
H	-3.00034	0.44587	-0.06432
H	-2.80106	1.87292	-1.12051
H	-1.55507	-1.31216	-1.66179

R = Me

P	0.47572	0.49529	0.43608
C	0.21324	0.21321	-1.17370
P	-1.36531	0.06059	-1.92052
C	-2.66800	1.06751	-1.18162
H	-3.59217	0.93496	-1.74789
H	-2.82595	0.76507	-0.14394
H	-2.36901	2.11492	-1.21670
Ge	1.36548	0.26551	-2.90326
S	-0.94673	0.60169	-3.81029
Cl	1.50410	-1.98737	-3.11929
C	-1.93512	-1.64543	-1.82033
H	-1.14775	-2.28383	-2.22379
H	-2.13151	-1.90876	-0.77867
H	-2.84348	-1.75903	-2.41518
C	2.30484	0.55840	0.60090
H	2.64535	-0.39485	1.01661
H	2.81162	0.72908	-0.35020
H	2.57759	1.34157	1.31174

R = *t*-Bu

P	0.64514	-0.06737	0.39256
C	0.15594	0.17902	-1.17433
P	-1.55582	0.02519	-1.60006
Ge	0.86855	0.94778	-2.98499
S	-1.57458	0.75029	-3.47899
Cl	1.60468	-1.01630	-3.84405
C	-2.21016	-1.71150	-1.53207
C	2.50253	0.11287	0.52546
C	2.74351	0.97348	1.76821
H	3.81522	1.00794	1.98921
H	2.39899	2.00031	1.62001
H	2.23375	0.56960	2.64742
C	3.02292	-1.30602	0.77838
H	4.09393	-1.26577	1.00462
H	2.52305	-1.77936	1.62860
H	2.88666	-1.94250	-0.09899
C	3.19393	0.71911	-0.68152
H	2.81654	1.71906	-0.90824
H	4.26783	0.81164	-0.48306
H	3.08440	0.08971	-1.56801
C	-2.30343	-2.17665	-0.08083
H	-2.61827	-3.22428	-0.07038
H	-1.34178	-2.11476	0.43381
H	-3.03922	-1.60723	0.49092
C	-3.59407	-1.71887	-2.18123
H	-3.96625	-2.74729	-2.19600
H	-4.31808	-1.11687	-1.62711
H	-3.55588	-1.35950	-3.21135
C	-1.26619	-2.61649	-2.31816
H	-1.15752	-2.28699	-3.35240
H	-0.26977	-2.64892	-1.87551
H	-1.67561	-3.63100	-2.32001
C	-2.60057	1.02715	-0.51375
H	-3.64096	0.97675	-0.83783
H	-2.51007	0.68813	0.51930
H	-2.25509	2.05837	-0.58749

R = Ph

P	0.75275	0.15675	0.68606
C	0.20134	0.08260	-0.87836
P	-1.50015	0.07398	-1.30251
C	2.55745	0.11649	0.55345
C	3.28287	0.76657	1.55499
C	3.24728	-0.55070	-0.46272
C	4.66751	0.78874	1.51919
C	4.63208	-0.53471	-0.48980
C	5.34241	0.14016	0.49438
H	5.22140	1.30698	2.29363
H	5.15898	-1.05685	-1.28021
C	-2.56353	0.97801	-0.15609
H	-3.58686	0.95978	-0.53532
H	-2.53234	0.51602	0.83252
H	-2.21128	2.00771	-0.09191
C	-2.14195	-1.61730	-1.31898
C	-2.57403	-2.20133	-2.50266
C	-2.19587	-2.33587	-0.12556
C	-3.07390	-3.49534	-2.48996
C	-2.69544	-3.62653	-0.11876
C	-3.13728	-4.20553	-1.30161
H	-3.40610	-3.95088	-3.41544
H	-2.73357	-4.18508	0.80927
Ge	0.99648	0.50325	-2.74996
S	-1.46877	0.91006	-3.12525
Cl	1.09799	-1.63142	-3.50057
H	-3.52404	-5.21825	-1.29537
H	-2.50337	-1.64337	-3.42898
H	-1.83047	-1.89838	0.79822
H	2.75445	1.26612	2.36145
H	2.70082	-1.11378	-1.21247
H	6.42622	0.15012	0.46877

R = Mes

P	0.96591	-0.20870	1.15034
C	0.24053	-0.15585	-0.34171
P	-1.47882	-0.05452	-0.73114
C	2.73679	0.03070	0.75499
C	3.33259	1.26923	1.03409
C	3.51429	-1.03510	0.27582
C	4.69295	1.43029	0.80738
C	4.87223	-0.82804	0.06588
C	5.47987	0.39567	0.31766
H	5.15021	2.39250	1.01988
H	5.47471	-1.65329	-0.30271
C	-2.56893	0.56460	0.57936
H	-3.51556	0.83802	0.10778
H	-2.75980	-0.17812	1.35274
H	-2.11432	1.45551	1.01374
C	-2.20013	-1.65504	-1.24272
C	-2.88631	-1.84426	-2.46385
C	-2.09676	-2.73217	-0.33689
C	-3.42645	-3.09573	-2.73441
C	-2.66302	-3.95812	-0.66744
C	-3.32463	-4.16925	-1.86359
H	-3.95368	-3.22927	-3.67379
H	-2.57692	-4.77320	0.04483
Ge	1.06620	0.61972	-2.07389
S	-1.31042	1.40708	-2.11259
Cl	0.82381	-1.26820	-3.32351
C	2.91956	-2.38269	-0.00948
H	2.31759	-2.74055	0.83197
H	2.26594	-2.35611	-0.88612
H	3.70368	-3.11759	-0.19636
C	2.51780	2.42294	1.53812
H	1.86684	2.81786	0.75178
H	1.87066	2.12881	2.37100

H	3.15931	3.23600	1.88074
C	6.93930	0.59928	0.04795
H	7.09955	0.92653	-0.98422
H	7.36313	1.36318	0.70238
H	7.50334	-0.32477	0.18774
C	-1.37402	-2.68366	0.97794
H	-0.31105	-2.89554	0.83754
H	-1.43223	-1.72807	1.49510
H	-1.77727	-3.44378	1.64863
C	-3.08650	-0.80094	-3.52375
H	-3.54978	0.10882	-3.14188
H	-2.13683	-0.50968	-3.97439
H	-3.72909	-1.20449	-4.30715
C	-3.89526	-5.50717	-2.21552
H	-4.05427	-6.12001	-1.32696
H	-4.84676	-5.40789	-2.74171
H	-3.21434	-6.05262	-2.87617

R = Mes*

P	1.02219	0.81062	1.23389
C	0.35356	0.33431	-0.21281
P	-1.34079	0.33882	-0.73559
Cl	1.83358	-2.50005	-1.17776
C	-2.92519	0.10433	0.19004
C	-3.52651	-1.18648	0.15060
C	-3.76672	1.22080	0.43954
C	-4.89572	-1.25299	-0.06737
C	-5.13239	1.07340	0.21133
C	-5.71221	-0.13287	-0.14751
H	-5.34452	-2.23405	-0.16259
H	-5.76466	1.94132	0.33946
C	2.83084	0.62285	0.92105
C	3.51951	-0.47562	1.48728
C	3.51393	1.48761	0.02815
C	4.74142	-0.83919	0.92771
C	4.72757	1.05196	-0.48989
C	5.32779	-0.14187	-0.11532
H	5.24545	-1.71139	1.31151
H	5.23588	1.67594	-1.21195
C	3.03239	-1.23104	2.74040
C	3.07339	2.93046	-0.31324
C	6.60442	-0.60611	-0.80864
C	-3.35425	2.53101	1.14508
C	-2.84797	-2.51491	0.54180
C	-7.18453	-0.27450	-0.51295
C	4.30451	3.80990	-0.58501
H	3.98576	4.85311	-0.65078
H	5.04442	3.72955	0.21457
H	4.79218	3.56936	-1.53126
C	2.35353	3.58666	0.86732
H	1.38970	3.12085	1.09746
H	2.96315	3.55150	1.77331
H	2.14414	4.63436	0.63400
C	2.18661	3.00399	-1.55876
H	2.66521	2.51776	-2.41162
H	1.22596	2.52012	-1.39164
H	2.00180	4.05046	-1.82127
C	7.70983	0.44014	-0.62628
H	8.62409	0.11384	-1.13029
H	7.42711	1.40806	-1.04516
H	7.93868	0.58636	0.43244
C	7.10994	-1.93770	-0.25988
H	8.00533	-2.24090	-0.80816
H	7.37999	-1.86784	0.79708
H	6.36604	-2.72972	-0.37470
C	6.31248	-0.78430	-2.30377
H	5.52348	-1.52362	-2.46018
H	5.98866	0.15107	-2.76504
H	7.21170	-1.12316	-2.82638
C	4.08037	-2.22338	3.25188
H	5.03695	-1.74092	3.46613
H	3.71657	-2.66454	4.18283
H	4.25081	-3.04227	2.54911
C	2.80025	-0.21738	3.87028
H	3.71537	0.34002	4.08561
H	2.01567	0.50209	3.62762
H	2.49664	-0.74043	4.78178
C	1.75476	-2.03499	2.49654
H	0.89563	-1.39363	2.29207
H	1.86534	-2.71168	1.64832
H	1.51276	-2.61918	3.38977
C	-1.89910	2.57043	1.59655
H	-1.64641	1.69172	2.19275
H	-1.17474	2.64120	0.78841
H	-1.74298	3.44891	2.22780
C	-4.17317	2.57089	2.45346
H	-3.88077	3.44931	3.03536
H	-5.24627	2.63122	2.27138
H	-3.98204	1.68219	3.05915
C	-3.70258	3.78784	0.34183
H	-4.76756	3.83023	0.10544
H	-3.46170	4.67751	0.92992
H	-3.15570	3.85180	-0.59876
C	-1.39365	-2.37358	0.97003
H	-1.06793	-3.30857	1.43270
H	-0.71274	-2.18519	0.14265
H	-1.28145	-1.58294	1.71587
C	-2.96834	-3.59541	-0.53484
H	-2.41216	-3.32314	-1.43067
H	-2.56012	-4.53465	-0.15210
H	-4.00822	-3.77819	-0.81509
C	-3.60440	-2.99731	1.79857
H	-3.55586	-2.25146	2.59609
H	-4.65531	-3.21169	1.60013
H	-3.13746	-3.91581	2.16384
C	-7.90213	1.07200	-0.55422
H	-7.92362	1.55454	0.42634
H	-7.43582	1.75762	-1.26625
H	-8.93836	0.92347	-0.86698
C	-7.89112	-1.16681	0.51444
H	-7.45826	-2.16838	0.54893
H	-7.82584	-0.73736	1.51716
H	-8.94871	-1.27047	0.25652
C	-7.28756	-0.91913	-1.90048
H	-6.80399	-0.29752	-2.65788
H	-6.81424	-1.90257	-1.92468
H	-8.33724	-1.04363	-2.18046
Ge	1.18461	-0.47975	-1.96823
S	-1.22515	-1.03131	-2.22715
C	-1.57955	1.88299	-1.67086
H	-1.49170	2.78866	-1.07898
H	-0.80890	1.87921	-2.44286
H	-2.55607	1.83759	-2.15460

E = Sn (II)

R-P=C(Sn(II)Cl)-P(O)ClR

R= H

P	0.69394	0.17761	0.95797
C	0.43367	-0.24523	-0.62073
P	-1.08519	-0.26900	-1.46159
Cl	1.12794	-2.83210	-2.73632
Sn	1.51799	-0.45893	-2.65407
O	-0.71546	0.00340	-2.90543
Cl	-2.49120	1.01015	-0.79574
H	-1.74987	-1.49475	-1.29647
H	2.12060	0.04682	0.97532

R = Me

P	0.72181	0.13247	0.93344
C	0.43372	-0.24877	-0.64741
P	-1.11358	-0.30536	-1.43756
Cl	1.26300	-2.82658	-2.78254
Sn	1.45565	-0.41400	-2.71131
O	-0.76960	-0.09686	-2.90620
Cl	-2.41790	1.12315	-0.82561
C	2.54508	-0.00237	1.11754
H	2.76234	-0.89088	1.71900
H	3.06826	-0.08207	0.16443
H	2.91551	0.86062	1.67671
C	-1.99834	-1.83290	-1.16096
H	-2.26663	-1.92689	-0.10835
H	-2.89607	-1.84112	-1.78113
H	-1.33187	-2.64585	-1.45894

R = *t*-Bu

P	0.77775	0.26149	0.18967
C	0.68143	-0.67807	-1.16759
P	-0.79635	-1.01124	-2.04391
Cl	1.86171	-3.93198	-1.50801
Sn	1.88495	-1.85709	-2.75632
O	-0.32695	-1.85354	-3.22266
C	2.55218	0.25173	0.78867
C	2.94326	1.72393	0.94238
H	3.00026	2.22938	-0.02520
H	2.22961	2.26936	1.56612
H	3.92653	1.79670	1.41852
C	3.53819	-0.47031	-0.10865
H	3.29150	-1.53137	-0.20491
H	3.58256	-0.01799	-1.10370
H	4.54542	-0.40958	0.31792
C	2.50455	-0.41153	2.16884
H	2.24867	-1.47128	2.09522
H	3.48671	-0.33360	2.64703
H	1.77652	0.07158	2.82683
C	-2.17744	-1.82307	-1.15138
C	-2.73177	-0.91109	-0.06231
H	-3.17359	-0.00345	-0.47615
H	-3.51286	-1.45188	0.47970
H	-1.96428	-0.62278	0.65987
C	-3.25746	-2.16925	-2.17727
H	-2.86181	-2.79732	-2.97704
H	-4.05289	-2.72213	-1.66942
H	-3.69828	-1.27515	-2.62152
C	-1.60949	-3.10570	-0.53748
H	-1.18108	-3.76606	-1.29240
H	-0.83561	-2.89738	0.20287
H	-2.42541	-3.63638	-0.03852
Cl	-1.59733	0.72375	-2.75716

R = Ph

P	0.76869	0.40716	0.36710
C	0.32912	-0.27270	-1.07706
P	-1.25917	-0.38268	-1.76459
C	2.56010	0.14271	0.44785
C	3.21703	-0.93231	-0.15842
C	3.30465	1.05937	1.19390
C	4.59118	-1.06259	-0.04746
C	4.68133	0.93433	1.29198
C	5.32515	-0.12531	0.66908
H	5.09098	-1.90392	-0.51389
H	5.25211	1.66014	1.85982
C	-2.22898	-1.75818	-1.15748
C	-2.39833	-2.86300	-1.98638
C	-2.76453	-1.75195	0.12912
C	-3.11306	-3.95871	-1.52577
C	-3.47382	-2.84996	0.58061
C	-3.64868	-3.95237	-0.24755
H	-3.24646	-4.82042	-2.16905
H	-3.89339	-2.84738	1.57976
Cl	1.22683	-3.09893	-2.96803
Sn	1.26292	-0.68361	-3.13364
O	-1.00178	-0.48986	-3.25919
Cl	-2.40697	1.24632	-1.36611
H	-2.63164	-0.88961	0.77323
H	-4.20557	-4.81131	0.10941
H	-1.96630	-2.86150	-2.97919
H	2.79902	1.88222	1.69024
H	6.40090	-0.23014	0.75313
H	2.64327	-1.69023	-0.68310

R = Mes

P	0.81483	0.14849	1.06723
C	0.40895	-0.51623	-0.39429
P	-1.08090	-0.46356	-1.30350
C	2.66191	0.10628	0.97024
C	3.38637	-1.09611	1.16780
C	3.34124	1.27726	0.55304
C	4.70096	-1.14259	0.71921
C	4.66443	1.14742	0.13074
C	5.34798	-0.05662	0.14628
H	5.25123	-2.06624	0.82958
H	5.17952	2.02416	-0.22711
C	2.73339	2.69829	0.59763
C	2.83977	-2.32862	1.91827
C	6.77099	-0.20911	-0.38269
C	2.04408	-3.26144	0.99906
H	1.15112	-2.78828	0.59490
H	2.64998	-3.58720	0.15124
H	1.73793	-4.15184	1.55678
C	1.97837	-1.89747	3.10732
H	2.53641	-1.24629	3.78480
H	1.06729	-1.36578	2.81347
H	1.65091	-2.77859	3.66562
C	3.98535	-3.15837	2.51514
H	4.56520	-3.68102	1.75196
H	4.66613	-2.54686	3.11247
H	3.55988	-3.92521	3.16662
C	1.67875	2.93158	-0.49152
H	0.77237	2.34527	-0.34172
H	1.38444	3.98493	-0.49466
H	2.07818	2.69269	-1.48075
C	2.12519	2.96216	1.98142

H	1.28965	2.29829	2.21286
H	2.87805	2.84160	2.76419
H	1.74459	3.98623	2.02784
C	3.80470	3.77596	0.39456
H	4.62441	3.68199	1.11055
H	4.22052	3.76678	-0.61563
H	3.34414	4.75521	0.54303
C	7.30819	1.08591	-0.98626
H	7.37186	1.88696	-0.24533
H	8.31596	0.91638	-1.37299
H	6.68934	1.43341	-1.81737
C	6.78592	-1.28797	-1.47229
H	6.44677	-2.25337	-1.09168
H	6.13572	-1.01120	-2.30588
H	7.80008	-1.41850	-1.86013
C	7.70325	-0.62941	0.75975
H	7.39941	-1.57993	1.20274
H	8.72523	-0.74634	0.38835
H	7.71319	0.12265	1.55263
C	-2.21224	-1.86802	-1.14971
C	-2.58374	-2.66334	-2.26760
C	-2.72329	-2.17165	0.12959
C	-3.42111	-3.74675	-2.04099
C	-3.56210	-3.26923	0.27660
C	-3.91915	-4.07519	-0.78806
H	-3.69900	-4.36170	-2.88713
H	-3.95336	-3.50152	1.26152
C	-2.19175	-2.37337	-3.70543
C	-3.13846	-1.32825	-4.29894
C	-2.13762	-3.60792	-4.59783
H	-1.19222	-1.95037	-3.70531
H	-3.12710	-0.39979	-3.72550
H	-2.84274	-1.09046	-5.32398
H	-4.16601	-1.70387	-4.31988
H	-1.52425	-4.39627	-4.15670
H	-3.12763	-4.01639	-4.81968
H	-1.68732	-3.33320	-5.55442
C	-2.44864	-1.36028	1.37999
C	-1.82953	-2.21308	2.48464
C	-3.71814	-0.66843	1.87460
H	-1.73054	-0.57141	1.15156
H	-0.93468	-2.73071	2.13503
H	-1.55281	-1.58365	3.33408
H	-2.53122	-2.96733	2.84857
H	-4.15146	-0.03220	1.10139
H	-4.47357	-1.39948	2.17360
H	-3.49241	-0.04498	2.74349
C	-4.82271	-5.26775	-0.58968
C	-4.10485	-6.57324	-0.92500
C	-6.11866	-5.13422	-1.38571
H	-5.08550	-5.29428	0.47392
H	-3.19159	-6.68714	-0.33728
H	-4.75291	-7.42943	-0.72080
H	-3.82752	-6.60988	-1.98172
H	-6.64874	-4.21377	-1.13178
H	-5.92206	-5.12114	-2.46090
H	-6.78168	-5.97853	-1.18040
Cl	1.40889	-3.00852	-2.70085
Sn	1.65319	-0.62462	-2.30773
O	-0.54882	-0.21078	-2.70771
Cl	-2.22926	1.17824	-0.85149

R = Mes*

P	0.99684	0.98162	1.43602
C	0.32100	0.45928	0.01622
P	-1.31866	0.25225	-0.55526
Cl	1.68431	-2.74232	-0.88072
O	-1.14275	-0.85681	-1.58357
C	-2.94413	0.04028	0.25567
C	-3.51410	-1.26415	0.16726
C	-3.80300	1.14019	0.50990
C	-4.87931	-1.34986	-0.06015
C	-5.16591	0.96280	0.28967
C	-5.72005	-0.24545	-0.09662
H	-5.30696	-2.33357	-0.20369
H	-5.81547	1.81479	0.43264
C	2.78936	0.74484	1.06840
C	3.46806	-0.34745	1.65773
C	3.44051	1.52448	0.08190
C	4.64539	-0.78430	1.05600
C	4.60410	1.01206	-0.48226
C	5.19284	-0.17189	-0.06039
H	5.14218	-1.65018	1.46291
H	5.08249	1.57052	-1.27615
C	3.00677	-1.02918	2.96145
C	3.02776	2.95383	-0.33604
C	6.41955	-0.71681	-0.78542
C	-3.42260	2.47555	1.18159
C	-2.78918	-2.59576	0.45011
C	-7.19088	-0.40937	-0.45884
C	4.29147	3.81453	-0.50990
H	3.99635	4.85597	-0.66007
H	4.93321	3.76534	0.37283
H	4.88390	3.52539	-1.37876
C	2.19624	3.66199	0.73375
H	1.19100	3.24429	0.84386
H	2.68701	3.63320	1.70958
H	2.05850	4.70981	0.45317
C	2.26477	2.97068	-1.66330
H	2.82924	2.46131	-2.44828
H	1.28984	2.49070	-1.57223
H	2.09839	4.00296	-1.98604
C	7.56016	0.30533	-0.71931
H	8.44048	-0.07828	-1.24282
H	7.28402	1.25385	-1.18452
H	7.84249	0.50930	0.31660
C	6.91469	-2.02801	-0.18076
H	7.77273	-2.39073	-0.75199
H	7.23956	-1.90299	0.85540
H	6.14447	-2.80245	-0.21056
C	6.05218	-0.97544	-2.25176
H	5.24357	-1.70651	-2.32705
H	5.72702	-0.06159	-2.75368
H	6.91790	-1.36528	-2.79470
C	4.04691	-2.02638	3.47929
H	5.02451	-1.56226	3.62994
H	3.71075	-2.41050	4.44513
H	4.16481	-2.88340	2.81216
C	2.83959	0.03818	4.05213
H	3.77646	0.57691	4.21491
H	2.06794	0.76955	3.80386
H	2.55083	-0.43643	4.99430
C	1.70073	-1.81068	2.79416
H	0.84353	-1.15746	2.61512
H	1.76034	-2.51055	1.95934
H	1.48867	-2.36923	3.71090
C	-1.95395	2.60194	1.55913
H	-1.61064	1.73524	2.12804
H	-1.30283	2.74224	0.69871
H	-1.82291	3.47785	2.19968
C	-4.19569	2.50355	2.51658
H	-3.93747	3.41458	3.06337

H	-5.27646	2.49317	2.37300
H	-3.92947	1.64609	3.13935
C	-3.84068	3.69554	0.35579
H	-4.91094	3.69779	0.14143
H	-3.61613	4.60774	0.91544
H	-3.30555	3.73905	-0.59239
C	-1.43489	-2.40693	1.12774
H	-1.06914	-3.37685	1.47307
H	-0.66290	-2.02189	0.46729
H	-1.52189	-1.75552	2.00163
C	-2.64970	-3.46042	-0.80549
H	-2.02271	-2.97595	-1.55061
H	-2.19788	-4.42059	-0.54189
H	-3.62746	-3.66187	-1.25117
C	-3.64340	-3.37186	1.47376
H	-3.82648	-2.77641	2.37169
H	-4.60672	-3.69077	1.07502
H	-3.10133	-4.27355	1.76929
C	-7.93909	0.92099	-0.45702
H	-7.96354	1.37521	0.53688
H	-7.49410	1.63669	-1.15278
H	-8.97413	0.75722	-0.76618
C	-7.87028	-1.34708	0.54626
H	-7.41241	-2.33805	0.55148
H	-7.81040	-0.94397	1.56021
H	-8.92635	-1.46927	0.29017
C	-7.28770	-1.01509	-1.86426
H	-6.82039	-0.36161	-2.60474
H	-6.79568	-1.98806	-1.91997
H	-8.33620	-1.15236	-2.14272
Sn	1.11531	-0.60660	-1.88096
Cl	-1.63779	1.92929	-1.71858

R-P=C(Sn(II)Cl)-P(O)MeR

R= H

P	0.72355	0.08207	1.06498
C	0.50601	-0.27368	-0.53604
P	-0.99701	-0.21989	-1.43563
Cl	1.17390	-2.93133	-2.53486
Sn	1.64945	-0.56471	-2.51938
O	-0.51930	-0.03131	-2.87599
H	2.14801	-0.05751	1.13695
H	-1.67650	-1.44386	-1.26598
C	-2.19104	1.02356	-0.94288
H	-3.06460	0.97197	-1.59553
H	-2.49551	0.84593	0.09129
H	-1.73392	2.01058	-1.02340

R = Me

P	0.71825	0.06447	0.93512
C	0.44651	-0.23992	-0.66390
P	-1.11538	-0.25737	-1.46330
Cl	1.30734	-2.83990	-2.74302
Sn	1.47410	-0.41763	-2.71947
O	-0.72259	-0.10740	-2.94180
C	2.54378	-0.03232	1.13969
H	2.77930	-0.92775	1.72309
H	3.07257	-0.07795	0.18728
H	2.89039	0.82814	1.71789
C	-1.97362	-1.80618	-1.17140
H	-2.24602	-1.89647	-0.11817
H	-2.87096	-1.84853	-1.79255
H	-1.30010	-2.61870	-1.44976
C	-2.23315	1.05979	-0.96442
H	-3.15316	1.00457	-1.55001
H	-2.46840	0.96763	0.09793
H	-1.74953	2.02056	-1.14348

R = *t*-Bu

P	0.72196	0.23363	0.43195
C	0.55535	-0.54342	-1.01629
P	-0.97651	-0.77277	-1.85938
Cl	1.80772	-3.70459	-1.86256
Sn	1.67091	-1.47832	-2.81757
O	-0.53233	-1.46702	-3.15976
C	-1.74174	0.81497	-2.24376
H	-2.63930	0.67308	-2.84771
H	-1.98750	1.35624	-1.32931
H	-1.01961	1.39881	-2.81636
C	2.53229	0.21797	0.92297
C	2.90065	1.67472	1.21247
H	2.88601	2.28202	0.30354
H	2.21500	2.12962	1.93297
H	3.90986	1.72548	1.63446
C	3.47300	-0.37482	-0.10776
H	3.23759	-1.42306	-0.31120
H	3.44664	0.18616	-1.04637
H	4.50380	-0.33854	0.26195
C	2.59084	-0.59124	2.22198
H	2.35277	-1.64349	2.04843
H	3.60027	-0.53846	2.64383
H	1.89653	-0.20347	2.97298
C	-2.22315	-1.79834	-0.97848
C	-2.77935	-1.04139	0.22496
H	-3.34695	-0.15577	-0.06945
H	-3.45891	-1.69967	0.77419
H	-1.98934	-0.73230	0.91441
C	-3.33933	-2.12472	-1.97356
H	-2.94695	-2.62306	-2.86164
H	-4.05533	-2.79761	-1.49250
H	-3.88821	-1.23400	-2.28851
C	-1.55000	-3.09220	-0.52277
H	-1.10985	-3.63838	-1.35804
H	-0.75780	-2.90324	0.20320
H	-2.30185	-3.73170	-0.05064

R = Ph

P	0.77065	0.34447	0.42772
C	0.33531	-0.27034	-1.04485
P	-1.27787	-0.33920	-1.72168
C	2.56982	0.11580	0.50511
C	3.24088	-0.93150	-0.13175
C	3.30171	1.02029	1.27690
C	4.61705	-1.04596	-0.02490
C	4.68020	0.91149	1.37183
C	5.33876	-0.12048	0.71860
H	5.12794	-1.86615	-0.51643
H	5.24102	1.62840	1.96073
C	-2.19987	-1.75821	-1.11236
C	-2.51386	-2.78697	-1.99410
C	-2.59245	-1.83913	0.22239
C	-3.22981	-3.88601	-1.54263
C	-3.30709	-2.93778	0.66725
C	-3.62771	-3.96038	-0.21687
H	-3.46990	-4.68948	-2.22916
H	-3.60951	-3.00122	1.70611
Cl	1.19731	-3.10657	-2.91185
Sn	1.23295	-0.69016	-3.11513
O	-1.00314	-0.47317	-3.22542
H	-2.32649	-1.05338	0.92269

H	-4.18411	-4.82216	0.13402
H	-2.18503	-2.72361	-3.02424
H	2.78507	1.82131	1.79723
H	6.41593	-0.21285	0.79959
H	2.67686	-1.67984	-0.68023
C	-2.28913	1.10885	-1.37979
H	-3.24521	1.01314	-1.89756
H	-2.46419	1.20367	-0.30647
H	-1.76358	1.99519	-1.73781

R = Mes

P	0.87119	0.14466	0.97501
C	0.30879	-0.28708	-0.51883
P	-1.28340	-0.24806	-1.27078
C	2.68939	0.07091	0.75779
C	3.36260	-1.16138	0.74362
C	3.41370	1.27000	0.68944
C	4.74790	-1.16294	0.62919
C	4.79708	1.21922	0.57297
C	5.48317	0.01220	0.53361
H	5.26887	-2.11612	0.62277
H	5.35377	2.15040	0.51565
C	-2.24537	-1.77291	-1.07095
C	-2.75354	-2.46716	-2.19246
C	-2.52655	-2.24764	0.22486
C	-3.51038	-3.61192	-1.97286
C	-3.29299	-3.39652	0.37895
C	-3.78844	-4.10116	-0.70508
H	-3.90165	-4.13884	-2.83769
H	-3.50581	-3.74636	1.38476
Cl	1.22043	-2.69121	-2.86199
Sn	1.33293	-0.27873	-2.55540
O	-0.88113	0.02316	-2.72926
C	-2.34615	1.11552	-0.74971
H	-3.19635	1.15782	-1.43394
H	-2.71383	0.99662	0.26857
H	-1.77571	2.04240	-0.82753
C	2.71767	2.59930	0.71303
H	2.19519	2.79139	-0.22937
H	1.96723	2.64941	1.50846
H	3.42987	3.41069	0.86964
C	2.63043	-2.46597	0.86012
H	1.90135	-2.44479	1.67598
H	2.08044	-2.70308	-0.05487
H	3.32850	-3.28151	1.05399
C	6.97270	-0.02371	0.37695
H	7.24951	-0.10658	-0.67884
H	7.43675	0.88418	0.76643
H	7.40715	-0.88097	0.89471
C	-2.01667	-1.60990	1.48379
H	-1.02090	-1.98785	1.73119
H	-1.93041	-0.52547	1.43505
H	-2.67685	-1.85005	2.31867
C	-2.53815	-2.06838	-3.62447
H	-2.79877	-1.02833	-3.81696
H	-1.49138	-2.18734	-3.90860
H	-3.14453	-2.70439	-4.27051
C	-4.58250	-5.35660	-0.52048
H	-3.94366	-6.23664	-0.64371
H	-5.02429	-5.40709	0.47593
H	-5.38394	-5.43248	-1.25804

R = Mes*

P	0.98449	1.17810	1.26456
C	0.28364	0.51089	-0.07810
P	-1.36975	0.32425	-0.67518
Cl	1.58008	-2.82633	-0.67886
O	-1.18000	-0.90053	-1.59494
C	-2.97933	0.12442	0.20040
C	-3.55979	-1.17623	0.19970
C	-3.84166	1.23703	0.38145
C	-4.92657	-1.27459	-0.02228
C	-5.20634	1.05485	0.17117
C	-5.76571	-0.17455	-0.13340
H	-5.35548	-2.26529	-0.10070
H	-5.85348	1.91631	0.26161
C	2.77732	0.86049	0.94036
C	3.42123	-0.18983	1.63459
C	3.47261	1.54973	-0.08516
C	4.60699	-0.69345	1.10399
C	4.64972	0.98366	-0.56204
C	5.20321	-0.17445	-0.03317
H	5.07362	-1.53402	1.59157
H	5.16842	1.47355	-1.37469
C	2.92475	-0.74872	2.98428
C	3.08032	2.94135	-0.63382
C	6.44616	-0.78763	-0.67071
C	-3.45395	2.60719	0.97673
C	-2.83758	-2.48670	0.56854
C	-7.23836	-0.35890	-0.47967
C	4.33611	3.71731	-1.06182
H	4.05552	4.74976	-1.28408
H	5.09086	3.73222	-0.27228
H	4.79275	3.31320	-1.96700
C	2.41215	3.80010	0.44243
H	1.43432	3.41845	0.75338
H	3.03802	3.87622	1.33466
H	2.24223	4.80840	0.05466
C	2.16466	2.85612	-1.85861
H	2.61178	2.23663	-2.64013
H	1.19508	2.43352	-1.59875
H	2.00241	3.85622	-2.27262
C	7.60085	0.22016	-0.63409
H	8.49340	-0.21212	-1.09541
H	7.35776	1.13694	-1.17525
H	7.84807	0.49339	0.39484
C	6.89651	-2.05891	0.04406
H	7.76775	-2.47503	-0.46783
H	7.18651	-1.86405	1.07978
H	6.11423	-2.82174	0.04214
C	6.12993	-1.14562	-2.12805
H	5.31111	-1.86707	-2.18263
H	5.84067	-0.26531	-2.70606
H	7.00820	-1.58723	-2.60763
C	3.93471	-1.71999	3.60248
H	4.91990	-1.26547	3.73165
H	3.57414	-2.01410	4.59089
H	4.04420	-2.63263	3.01199
C	2.77012	0.41268	3.97651
H	3.71739	0.94261	4.10489
H	2.01867	1.13654	3.65494
H	2.45957	0.02772	4.95215
C	1.60358	-1.51363	2.86963
H	0.76319	-0.86228	2.62201
H	1.65745	-2.28626	2.10124
H	1.37081	-1.98476	3.82949
C	-1.98917	2.73190	1.37280
H	-1.67719	1.90066	2.00792
H	-1.30170	2.78882	0.53138
H	-1.84834	3.65001	1.94874
C	-4.23921	2.72900	2.29939

H	-3.97227	3.66660	2.79494
H	-5.31836	2.72635	2.14444
H	-3.99295	1.90547	2.97355
C	-3.85568	3.78311	0.08071
H	-4.92833	3.79031	-0.12121
H	-3.60899	4.72521	0.57786
H	-3.34199	3.76871	-0.88126
C	-1.49100	-2.24400	1.24162
H	-1.10131	-3.19148	1.62111
H	-0.73046	-1.85991	0.56859
H	-1.59736	-1.56226	2.08979
C	-2.68750	-3.42803	-0.62940
H	-2.05828	-2.98773	-1.39928
H	-2.23363	-4.36791	-0.30353
H	-3.66289	-3.66073	-1.06564
C	-3.69532	-3.20201	1.63236
H	-3.89533	-2.55040	2.48670
H	-4.65036	-3.55887	1.24527
H	-3.14666	-4.07550	1.99337
C	-7.98349	0.97053	-0.56496
H	-8.00348	1.49046	0.39630
H	-7.53885	1.63701	-1.30840
H	-9.01999	0.78953	-0.85940
C	-7.91699	-1.22447	0.58861
H	-7.46110	-2.21381	0.65925
H	-7.85173	-0.75393	1.57273
H	-8.97448	-1.36101	0.34560
C	-7.34323	-1.05811	-1.84023
H	-6.88096	-0.45589	-2.62619
H	-6.85025	-2.03210	-1.83262
H	-8.39305	-1.21457	-2.10341
Sn	1.04999	-0.76816	-1.84906
C	-1.67888	1.68714	-1.83223
H	-2.62307	1.48478	-2.34079
H	-1.70424	2.67625	-1.38659
H	-0.87234	1.64947	-2.56812

R-P=C(Sn(II)Cl)-P(S)ClR

R= H

P	0.04347	0.93418	0.51148
C	0.15358	0.42272	-1.06044
P	-1.15150	-0.15594	-2.04328
Cl	1.98926	-1.66301	-3.04141
Sn	1.63672	0.71514	-2.79121
S	-0.88388	0.68025	-3.82066
H	1.40813	1.33760	0.68077
Cl	-3.02228	0.08518	-1.29632
H	-1.08601	-1.55781	-2.09220

R = Me

P	0.47280	0.42786	0.60047
C	0.19222	0.15376	-1.00729
P	-1.37691	-0.03348	-1.72371
Cl	1.42712	-1.93366	-3.49360
Sn	1.36302	0.43680	-2.96333
S	-1.23062	0.82849	-3.51695
Cl	-2.90685	0.76724	-0.63210
C	2.30197	0.52513	0.74319
H	2.64605	-0.35339	1.29800
H	2.81629	0.56142	-0.21785
H	2.56725	1.40293	1.33814
C	-1.83641	-1.76633	-1.82376
H	-1.93861	-2.17242	-0.81634
H	-2.77882	-1.85137	-2.36610
H	-1.03982	-2.28151	-2.36589

R =*t*-Bu

P	0.31834	0.07007	0.55364
C	0.06825	0.23399	-1.07896
P	-1.54089	0.03444	-1.74327
Cl	1.91897	-0.97641	-3.83029
Sn	1.10169	1.12776	-2.93425
S	-1.50441	0.90379	-3.54000
C	2.14809	0.21017	0.92293
C	2.26744	1.25131	2.03890
H	3.29788	1.27920	2.40792
H	2.01022	2.25301	1.68511
H	1.61637	1.01484	2.88502
C	2.53218	-1.16908	1.47082
H	3.55828	-1.13613	1.85210
H	1.88222	-1.47615	2.29528
H	2.48595	-1.93636	0.69418
C	3.04273	0.57591	-0.24522
H	4.08928	0.59322	0.07877
H	2.96825	-0.15085	-1.05880
H	2.81398	1.57359	-0.63078
C	-2.13284	-1.72407	-1.82389
C	-1.10395	-2.51474	-2.63050
C	-2.26118	-2.30242	-0.41877
C	-3.48336	-1.73962	-2.53685
H	-0.98343	-2.11904	-3.63955
H	-0.12202	-2.51925	-2.15569
H	-1.45303	-3.54863	-2.70701
H	-3.00651	-1.77476	0.17742
H	-2.57411	-3.34681	-0.50676
H	-1.31160	-2.28523	0.11989
H	-3.80573	-2.77971	-2.63808
H	-4.24890	-1.20150	-1.97548
H	-3.41446	-1.30930	-3.53760
Cl	-2.95664	0.94086	-0.56767

R = Ph

P	0.79669	0.50120	0.49437
C	0.27373	-0.12477	-0.95053
P	-1.36146	-0.12752	-1.52336
C	2.56713	0.11028	0.51518
C	3.11400	-1.03855	-0.06525
C	3.41009	0.99748	1.18852
C	4.47832	-1.27093	-0.00119
C	4.77577	0.76867	1.23979
C	5.31050	-0.36429	0.64261
H	4.89189	-2.16742	-0.44876
H	5.42350	1.47135	1.75135
C	-2.20735	-1.64461	-1.04291
C	-2.55129	-2.58616	-2.00447
C	-2.47388	-1.88259	0.30464
C	-3.17294	-3.76415	-1.61707
C	-3.09360	-3.05958	0.68218
C	-3.44424	-4.00009	-0.27917
H	-3.43918	-4.50019	-2.36631
H	-3.30365	-3.24553	1.72894
Cl	1.06515	-2.92125	-2.93321
Sn	1.33096	-0.50811	-2.94067
S	-1.24599	0.12561	-3.49360
Cl	-2.51026	1.32678	-0.65510
H	-2.20107	-1.14908	1.05538
H	-2.32368	-2.39829	-3.04647
H	-3.92853	-4.92255	0.02038
H	2.46423	-1.77266	-0.53242

H	2.99089	1.87838	1.66524
H	6.37768	-0.54890	0.69026

R = Mes

P	-0.93732	-0.20597	1.88111
C	-0.02011	-0.45939	0.51954
P	1.44833	-1.39282	0.37799
C	-2.55934	0.30818	1.15171
C	-2.76982	1.60905	0.62756
C	-3.57029	-0.67635	1.01266
C	-3.86415	1.80556	-0.20666
C	-4.64432	-0.39358	0.16903
C	-4.78698	0.81135	-0.49751
H	-4.01137	2.78343	-0.64253
H	-5.39857	-1.14987	0.02484
C	-3.59316	-2.01946	1.77874
C	-1.91020	2.84056	0.97870
C	-5.92633	1.08979	-1.47324
C	-0.67090	2.94718	0.08409
H	0.00591	2.10301	0.20280
H	-0.95329	2.99324	-0.97000
H	-0.12055	3.86245	0.32264
C	-1.50643	2.81139	2.45428
H	-2.38583	2.76196	3.10125
H	-0.85864	1.96739	2.71116
H	-0.94452	3.71557	2.70285
C	-2.70707	4.13992	0.79438
H	-2.89483	4.37119	-0.25591
H	-3.66363	4.11199	1.32192
H	-2.12221	4.96918	1.19911
C	-2.54750	-3.01484	1.26118
H	-1.52186	-2.70137	1.45400
H	-2.68828	-3.98121	1.75361
H	-2.65423	-3.16943	0.18438
C	-3.39009	-1.77090	3.27938
H	-2.41322	-1.34312	3.51271
H	-4.15587	-1.09393	3.66607
H	-3.46475	-2.71702	3.82244
C	-4.95062	-2.72055	1.64944
H	-5.77698	-2.08276	1.97180
H	-5.14942	-3.06244	0.63108
H	-4.94509	-3.60563	2.28942
C	-6.83223	-0.12293	-1.66972
H	-7.32638	-0.41891	-0.74079
H	-7.61360	0.12024	-2.39373
H	-6.28010	-0.98343	-2.05586
C	-5.33490	1.47230	-2.83529
H	-4.70409	2.36086	-2.76789
H	-4.72523	0.66008	-3.23856
H	-6.13645	1.68405	-3.54849
C	-6.78227	2.24871	-0.94859
H	-6.19915	3.16457	-0.83359
H	-7.59896	2.45897	-1.64489
H	-7.21815	2.00388	0.02322
C	2.98458	-0.40887	0.21642
C	3.83798	-0.44987	-0.91934
C	3.31871	0.43026	1.30631
C	4.97018	0.35327	-0.91711
C	4.47156	1.20242	1.23077
C	5.31255	1.18646	0.13544
H	5.61438	0.32185	-1.78625
H	4.72392	1.84125	2.07032
C	3.65406	-1.34946	-2.12545
C	4.30732	-2.70737	-1.86787
C	4.16339	-0.75268	-3.43341
H	2.58969	-1.50847	-2.26809
H	3.90858	-3.18789	-0.97215
H	4.13273	-3.37866	-2.71276
H	5.38808	-2.59646	-1.73817
H	3.76918	0.25249	-3.59326
H	5.25489	-0.71708	-3.48973
H	3.82488	-1.37873	-4.26237
C	2.53825	0.55795	2.60170
C	2.11608	2.00168	2.86606
C	3.33912	0.01203	3.78369
H	1.62281	-0.02913	2.54124
H	1.56251	2.41791	2.02354
H	1.47887	2.04720	3.75269
H	2.97931	2.64525	3.05026
H	3.64231	-1.02243	3.61885
H	4.24068	0.60556	3.95489
H	2.73581	0.05049	4.69432
C	6.55354	2.04367	0.09345
C	6.48822	3.06694	-1.03806
C	7.82014	1.19668	-0.00507
H	6.59244	2.59383	1.04048
H	5.59735	3.69315	-0.95700
H	7.36709	3.71643	-1.01732
H	6.46115	2.57446	-2.01356
H	7.88493	0.48035	0.81673
H	7.84633	0.63404	-0.94194
H	8.70850	1.83271	0.02467
Cl	0.67675	0.75464	-2.72052
Sn	-0.87825	-0.74190	-1.57971
S	0.89542	-2.68608	-1.05223
Cl	1.84440	-2.59883	2.01188

R = Mes*

P	0.92866	0.74682	1.43933
C	0.31814	0.34860	-0.05561
P	-1.34254	0.24874	-0.60098
Cl	1.93487	-2.68408	-1.03843
C	-2.94999	0.06365	0.28455
C	-3.53686	-1.23679	0.25199
C	-3.79874	1.17699	0.52189
C	-4.90173	-1.31587	0.02096
C	-5.16034	1.01055	0.28287
C	-5.72544	-0.20168	-0.07595
H	-5.34293	-2.30085	-0.06777
H	-5.80095	1.87376	0.39774
C	2.74326	0.63526	1.12938
C	3.46606	-0.45899	1.66182
C	3.38659	1.52546	0.23400
C	4.68114	-0.78817	1.06768
C	4.59047	1.11674	-0.33007
C	5.22518	-0.06800	0.01562
H	5.21159	-1.65528	1.42660
H	5.06277	1.76065	-1.06042
C	3.01273	-1.24993	2.90437
C	2.92841	2.97112	-0.06413
C	6.49226	-0.49958	-0.71544
C	-3.41618	2.49777	1.22428
C	-2.85592	-2.55743	0.67197
C	-7.19115	-0.35651	-0.46050
C	4.16156	3.89182	-0.09125
H	3.82955	4.93129	-0.15173
H	4.76502	3.77809	0.81227
H	4.80363	3.71361	-0.95461
C	2.02424	3.53634	1.03092
H	1.03870	3.06299	1.06194
H	2.48104	3.43920	2.01879
H	1.84397	4.59824	0.84244

C	2.22470	3.09269	-1.41770
H	2.84362	2.68685	-2.22155
H	1.26845	2.56896	-1.42216
H	2.02860	4.14536	-1.64333
C	7.58034	0.56742	-0.54956
H	8.48870	0.26358	-1.07749
H	7.26902	1.53300	-0.95334
H	7.83106	0.70945	0.50469
C	7.03777	-1.82646	-0.19427
H	7.92371	-2.10639	-0.76946
H	7.33524	-1.76170	0.85560
H	6.30645	-2.63165	-0.29762
C	6.16632	-0.66840	-2.20455
H	5.39613	-1.42972	-2.34971
H	5.80550	0.26277	-2.64674
H	7.06063	-0.97589	-2.75403
C	4.09252	-2.22339	3.38426
H	5.03922	-1.71965	3.59361
H	3.75354	-2.68948	4.31234
H	4.27370	-3.02553	2.66514
C	2.76244	-0.26530	4.05558
H	3.66337	0.31323	4.27491
H	1.95584	0.43621	3.83356
H	2.48135	-0.81433	4.95883
C	1.75454	-2.08238	2.65223
H	0.87514	-1.45838	2.47816
H	1.87331	-2.73232	1.78425
H	1.53858	-2.69704	3.53148
C	-1.95567	2.59864	1.64310
H	-1.64092	1.71865	2.20773
H	-1.27861	2.73971	0.80410
H	-1.83252	3.46423	2.29920
C	-4.21795	2.49636	2.54408
H	-3.95674	3.38613	3.12348
H	-5.29530	2.50806	2.37704
H	-3.97835	1.61622	3.14555
C	-3.81259	3.74425	0.42869
H	-4.87745	3.75407	0.18864
H	-3.60325	4.63657	1.02498
H	-3.25521	3.82203	-0.50378
C	-1.40559	-2.41302	1.11518
H	-1.09333	-3.33779	1.60668
H	-0.70990	-2.25222	0.29406
H	-1.29762	-1.60498	1.84300
C	-2.96245	-3.65680	-0.38659
H	-2.39415	-3.40158	-1.28000
H	-2.55980	-4.58922	0.01796
H	-3.99796	-3.84419	-0.67876
C	-3.62442	-3.01949	1.92957
H	-3.58350	-2.26021	2.71466
H	-4.67331	-3.23596	1.72412
H	-3.16180	-3.93204	2.31474
C	-7.91821	0.98427	-0.51850
H	-7.95734	1.47120	0.45932
H	-7.44660	1.66981	-1.22694
H	-8.94866	0.82609	-0.84531
C	-7.90522	-1.24975	0.56091
H	-7.46615	-2.24827	0.60559
H	-7.85722	-0.81565	1.56262
H	-8.95831	-1.36191	0.28858
C	-7.26830	-1.00813	-1.84655
H	-6.77598	-0.38714	-2.59864
H	-6.78917	-1.98904	-1.85844
H	-8.31280	-1.13989	-2.14217
Sn	1.34991	-0.49570	-1.93953
S	-1.27089	-1.08018	-2.09336
Cl	-1.60583	2.04713	-1.59608

R-P=C(Sn(II)Cl)-P(S)MeR

R= H

P	0.14006	1.22038	0.52450
C	0.09232	0.53244	-0.98295
P	-1.38030	0.05366	-1.80612
Cl	1.46433	-2.05666	-2.65996
Sn	1.49981	0.36486	-2.77288
S	-1.04351	0.56808	-3.70953
H	1.54755	1.48074	0.59750
C	-2.93538	0.68045	-1.14607
H	-3.76232	0.28726	-1.74018
H	-3.04507	0.36288	-0.10656
H	-2.93290	1.76939	-1.20107
H	-1.48855	-1.34373	-1.66450

R =Me

P	0.41911	0.55090	0.42353
C	0.22966	0.21528	-1.18559
P	-1.34575	0.06851	-1.94670
Cl	1.49838	-2.17341	-3.31381
Sn	1.51654	0.25158	-3.07401
S	-1.01458	0.67066	-3.83869
C	2.23661	0.62344	0.68954
H	2.52588	-0.21980	1.32396
H	2.80823	0.58340	-0.23856
H	2.48441	1.53699	1.23630
C	-1.88183	-1.65089	-1.88715
H	-2.06262	-1.94473	-0.85088
H	-2.79358	-1.76548	-2.47638
H	-1.08763	-2.26543	-2.31463
C	-2.66387	1.02666	-1.16818
H	-3.59105	0.88473	-1.72684
H	-2.80148	0.69779	-0.13578
H	-2.39119	2.08176	-1.18230

R = *t*-Bu

P	0.56346	-0.00369	0.24580
C	0.20244	0.36965	-1.32928
P	-1.46089	0.15893	-1.90186
Cl	1.99211	-0.47670	-4.22735
Sn	1.07723	1.49596	-3.13170
S	-1.51761	1.14443	-3.66103
C	2.38943	0.26495	0.56910
C	2.46646	1.17307	1.79859
H	3.50658	1.25859	2.13008
H	2.10027	2.17947	1.57952
H	1.88246	0.77518	2.63327
C	2.93184	-1.12273	0.92676
H	3.96966	-1.03368	1.26517
H	2.35838	-1.58846	1.73338
H	2.91540	-1.79341	0.06427
C	3.18279	0.85521	-0.58020
H	4.23822	0.94156	-0.29837
H	3.13624	0.22421	-1.47196
H	2.83719	1.86118	-0.83414
C	-1.94578	-1.62226	-2.12406
C	-0.88206	-2.31237	-2.97279
C	-2.05997	-2.30479	-0.76329
C	-3.29122	-1.66273	-2.84819
H	-0.75924	-1.82804	-3.94249
H	0.09202	-2.31834	-2.48197
H	-1.18821	-3.34897	-3.14235

H	-2.87282	-1.89496	-0.16007
H	-2.27187	-3.36611	-0.92299
H	-1.13386	-2.23524	-0.18824
H	-3.55922	-2.70825	-3.02581
H	-4.09527	-1.21418	-2.25994
H	-3.24184	-1.15750	-3.81433
C	-2.65385	0.88669	-0.74984
H	-3.66676	0.78830	-1.14226
H	-2.57866	0.40864	0.22795
H	-2.41236	1.94529	-0.65323

R = Ph

P	0.80164	0.37975	0.54177
C	0.30266	-0.16374	-0.94387
P	-1.36835	-0.12300	-1.47453
C	2.58705	0.05599	0.56211
C	3.17256	-1.04326	-0.07255
C	3.39744	0.93346	1.28518
C	4.54351	-1.23510	-0.01182
C	4.76955	0.74550	1.33430
C	5.34344	-0.33756	0.68329
H	4.98754	-2.09344	-0.50298
H	5.39193	1.44109	1.88568
C	-2.19034	-1.66598	-1.00418
C	-2.66930	-2.54059	-1.97080
C	-2.33809	-1.97222	0.34781
C	-3.30818	-3.71043	-1.58628
C	-2.97760	-3.13982	0.72602
C	-3.46510	-4.00856	-0.24210
H	-3.67576	-4.39393	-2.34265
H	-3.08895	-3.37731	1.77765
Cl	1.09664	-2.91723	-2.92846
Sn	1.33242	-0.49623	-2.94776
S	-1.25377	0.10492	-3.46572
H	-1.93753	-1.31029	1.10918
H	-2.52734	-2.30583	-3.01913
H	-3.96074	-4.92560	0.05559
H	2.54834	-1.77109	-0.58255
H	2.94805	1.77521	1.80346
H	6.41577	-0.49045	0.72830
C	-2.35678	1.19026	-0.72205
H	-3.36725	1.15215	-1.13234
H	-2.39647	1.05941	0.36103
H	-1.89872	2.15084	-0.95889

R = Mes

P	0.93983	0.19335	1.07394
C	0.30451	-0.24543	-0.39465
P	-1.36033	-0.07448	-0.95311
C	2.74335	0.02288	0.79747
C	3.35423	-1.24122	0.75021
C	3.52497	1.18536	0.71346
C	4.73332	-1.31034	0.58964
C	4.89917	1.06674	0.55170
C	5.52271	-0.17240	0.47986
H	5.20570	-2.28791	0.55681
H	5.49929	1.96956	0.48175
C	-2.25582	-1.67117	-0.94718
C	-2.89003	-2.21766	-2.08679
C	-2.34293	-2.35906	0.28282
C	-3.56950	-3.42287	-1.95515
C	-3.04186	-3.55940	0.34630
C	-3.65613	-4.11802	-0.75951
H	-4.05419	-3.83116	-2.83636
H	-3.10104	-4.06975	1.30296
Cl	0.86444	-2.55201	-2.92200
Sn	1.43610	-0.24384	-2.36070
S	-1.00358	0.81374	-2.73139
C	-2.41709	1.07050	-0.02102
H	-3.29932	1.26601	-0.63421
H	-2.73451	0.66699	0.93920
H	-1.86888	2.00209	0.12359
C	2.89703	2.54709	0.76570
H	2.33017	2.75855	-0.14644
H	2.19777	2.63851	1.60281
H	3.65593	3.32325	0.87348
C	2.56573	-2.51178	0.88119
H	1.88915	-2.47383	1.74045
H	1.95082	-2.70352	-0.00267
H	3.23301	-3.36396	1.01652
C	7.00241	-0.27981	0.27250
H	7.24050	-0.33514	-0.79446
H	7.52612	0.58708	0.67947
H	7.40666	-1.17794	0.74304
C	-1.69734	-1.92183	1.56627
H	-0.67029	-2.29149	1.62146
H	-1.64475	-0.84501	1.70873
H	-2.24440	-2.33843	2.41335
C	-2.89606	-1.61302	-3.46029
H	-3.26815	-0.58886	-3.47103
H	-1.89223	-1.59373	-3.88632
H	-3.53271	-2.21250	-4.11206
C	-4.37222	-5.42949	-0.67724
H	-4.65532	-5.66647	0.34948
H	-5.27379	-5.42972	-1.29313
H	-3.73157	-6.23946	-1.03939

R = Mes*

P	0.91279	0.90362	1.31404
C	0.28475	0.37652	-0.13009
P	-1.39171	0.31040	-0.69050
Cl	1.84504	-2.78716	-0.86494
C	-2.98938	0.15191	0.23748
C	-3.58904	-1.13855	0.30658
C	-3.83558	1.28303	0.38556
C	-4.95698	-1.22724	0.08951
C	-5.19969	1.11296	0.16113
C	-5.77531	-0.12089	-0.09399
H	-5.40336	-2.21390	0.07776
H	-5.83418	1.98745	0.20789
C	2.73156	0.74027	1.02711
C	3.43411	-0.32083	1.64563
C	3.40639	1.57091	0.09595
C	4.66133	-0.69537	1.10404
C	4.62730	1.12761	-0.40004
C	5.24236	-0.03953	0.03137
H	5.17401	-1.54385	1.52730
H	5.12975	1.72514	-1.14836
C	2.95624	-1.01924	2.93483
C	2.94985	2.99067	-0.31410
C	6.52883	-0.51763	-0.63413
C	-3.43507	2.65218	0.97828
C	-2.91184	-2.42949	0.81039
C	-7.24498	-0.29816	-0.45440
C	4.17469	3.88134	-0.57979
H	3.84155	4.91543	-0.69771
H	4.88730	3.84524	0.24730
H	4.69979	3.61308	-1.49801
C	2.17883	3.68110	0.81295
H	1.21422	3.21071	1.02747
H	2.75669	3.69216	1.74007

H	1.96364	4.71537	0.53025
C	2.10405	2.99443	-1.59000
H	2.62977	2.49968	-2.41034
H	1.15455	2.48386	-1.43622
H	1.89399	4.02430	-1.89535
C	7.61818	0.55184	-0.49465
H	8.54058	0.21481	-0.97620
H	7.32535	1.49463	-0.96132
H	7.83701	0.75163	0.55736
C	7.04941	-1.81542	-0.02204
H	7.95129	-2.13146	-0.55208
H	7.31376	-1.69275	1.03150
H	6.31667	-2.62187	-0.10282
C	6.24893	-0.76871	-2.12101
H	5.47698	-1.53154	-2.24765
H	5.90964	0.13829	-2.62596
H	7.15746	-1.11279	-2.62360
C	4.01406	-1.97758	3.48899
H	4.96767	-1.47820	3.67642
H	3.65708	-2.37615	4.44147
H	4.18764	-2.82791	2.82544
C	2.71945	0.04780	4.01338
H	3.63054	0.62252	4.19853
H	1.92849	0.74749	3.73608
H	2.42207	-0.43163	4.95052
C	1.68369	-1.84378	2.73611
H	0.81534	-1.21777	2.52276
H	1.78992	-2.55013	1.91181
H	1.45927	-2.39702	3.65328
C	-1.98715	2.73606	1.44667
H	-1.74384	1.91615	2.12490
H	-1.25082	2.73209	0.64668
H	-1.84197	3.66941	1.99665
C	-4.27243	2.80764	2.26633
H	-3.98679	3.73384	2.77266
H	-5.34284	2.85235	2.06518
H	-4.09119	1.97568	2.95056
C	-3.77335	3.83189	0.06159
H	-4.83471	3.84906	-0.19357
H	-3.54366	4.77114	0.57205
H	-3.21223	3.81409	-0.87243
C	-1.45564	-2.25766	1.21952
H	-1.13202	-3.14940	1.76197
H	-0.77790	-2.14729	0.37550
H	-1.33692	-1.40421	1.89132
C	-3.03772	-3.59950	-0.16772
H	-2.48840	-3.40448	-1.08763
H	-2.62438	-4.50123	0.29196
H	-4.07896	-3.80808	-0.42374
C	-3.66535	-2.79832	2.10668
H	-3.60960	-1.98701	2.83701
H	-4.71826	-3.02247	1.93084
H	-3.20207	-3.68487	2.54781
C	-7.96383	1.03748	-0.62460
H	-7.99306	1.60798	0.30731
H	-7.49265	1.65559	-1.39302
H	-8.99752	0.85898	-0.93007
C	-7.95688	-1.09289	0.64682
H	-7.52190	-2.08567	0.77705
H	-7.89891	-0.57188	1.60566
H	-9.01260	-1.22290	0.39304
C	-7.33857	-1.06865	-1.77690
H	-6.85082	-0.51891	-2.58546
H	-6.86366	-2.04914	-1.70707
H	-8.38630	-1.22061	-2.05073
Sn	1.28719	-0.66682	-1.93018
S	-1.32241	-1.17446	-2.07603
C	-1.62008	1.77880	-1.74502
H	-1.52233	2.72776	-1.22712
H	-0.85373	1.70941	-2.51788
H	-2.59821	1.70351	-2.22135

E = Pb(II)

R-P=C(Pb (II)Cl)-P(O)ClR

R= H

P	0.74445	0.05730	1.03903
C	0.55043	-0.27560	-0.56974
P	-0.95773	-0.37109	-1.42649
O	-0.66733	0.07034	-2.84053
Pb	1.73236	-0.22944	-2.67214
Cl	1.56902	-2.69827	-2.96595
H	2.17718	0.04900	1.08979
H	-1.48508	-1.67128	-1.35966
Cl	-2.47867	0.68126	-0.61557

R = Me

P	0.90353	0.29640	0.88416
C	0.59512	-0.21487	-0.65421
P	-0.97078	-0.31466	-1.40622
O	-0.71293	-0.16893	-2.89395
Pb	1.63496	-0.56190	-2.79913
Cl	1.38963	-3.05660	-2.69189
C	2.72968	0.17283	1.06806
H	3.22820	-0.19998	0.17321
H	3.12791	1.15607	1.33520
H	2.94833	-0.49536	1.90646
C	-1.81928	-1.83808	-1.01086
H	-2.03960	-1.88274	0.05590
H	-2.74275	-1.88996	-1.58971
H	-1.15491	-2.65752	-1.29672
Cl	-2.26732	1.12589	-0.78962

R = *t*-Bu

P	0.71803	0.32595	0.23937
C	0.61858	-0.62568	-1.10658
P	-0.85234	-0.95388	-1.99684
O	-0.42158	-1.77293	-3.19872
C	2.49262	0.30287	0.84799
C	2.89865	1.77139	0.99339
H	2.96476	2.26999	0.02270
H	2.18634	2.32775	1.60888
H	3.87993	1.83917	1.47460
C	3.47680	-0.43556	-0.03781
H	3.22038	-1.49544	-0.12470
H	3.53080	0.01341	-1.03486
H	4.48294	-0.38091	0.39208
C	2.43285	-0.35113	2.23179
H	2.16696	-1.40878	2.16355
H	3.41257	-0.27907	2.71603
H	1.70503	0.14293	2.88173
C	-2.22046	-1.77469	-1.08997
C	-2.75790	-0.88298	0.02336
H	-3.20346	0.03305	-0.36749
H	-3.53245	-1.43205	0.56665
H	-1.97992	-0.60893	0.73973
C	-3.31872	-2.10472	-2.10157
H	-2.93690	-2.71972	-2.91792
H	-4.10633	-2.66470	-1.58912
H	-3.76567	-1.20318	-2.52442
C	-1.64221	-3.06758	-0.50717

H	-1.22879	-3.71639	-1.28043
H	-0.85383	-2.87216	0.22137
H	-2.44828	-3.60689	-0.00128
Cl	-1.66157	0.80348	-2.65880
Pb	1.91623	-1.86800	-2.71183
Cl	1.88286	-4.00331	-1.38999

R = Ph

P	0.77895	0.43510	0.40549
C	0.32367	-0.22947	-1.03787
P	-1.26032	-0.32183	-1.73818
C	2.57399	0.15329	0.45781
C	3.20263	-0.94414	-0.13848
C	3.34777	1.07367	1.16726
C	4.57748	-1.09218	-0.05541
C	4.72505	0.93113	1.23710
C	5.34081	-0.15020	0.62350
H	5.05405	-1.95239	-0.51194
H	5.31814	1.66086	1.77647
C	-2.20162	-1.73058	-1.15809
C	-2.35347	-2.81633	-2.01514
C	-2.72839	-1.77196	0.13137
C	-3.04103	-3.93979	-1.58059
C	-3.41189	-2.89647	0.55687
C	-3.56870	-3.97973	-0.29973
H	-3.15925	-4.78664	-2.24622
H	-3.82524	-2.92979	1.55812
O	-1.04373	-0.36364	-3.23593
Pb	1.35411	-0.61546	-3.16056
Cl	1.32205	-3.12247	-3.04755
Cl	-2.41591	1.28092	-1.24343
H	-1.92894	-2.77856	-3.01051
H	-4.10477	-4.85984	0.03705
H	-2.60905	-0.92489	0.79792
H	2.60623	-1.70529	-0.63328
H	2.86526	1.91361	1.65804
H	6.41657	-0.26873	0.68660

R = Mes

P	0.79515	0.20083	1.11754
C	0.38237	-0.45803	-0.34086
P	-1.08012	-0.40723	-1.28726
C	2.64565	0.13049	0.99246
C	3.35019	-1.08751	1.16665
C	3.33831	1.28674	0.55543
C	4.64852	-1.16318	0.67451
C	4.64601	1.12932	0.09281
C	5.30324	-0.08999	0.08545
H	5.18016	-2.09981	0.76543
H	5.17061	1.99646	-0.27515
C	2.76254	2.72066	0.61839
C	2.80102	-2.30812	1.93484
C	6.70913	-0.27136	-0.47968
C	1.96687	-3.23106	1.03967
H	1.07002	-2.74476	0.66029
H	2.54227	-3.56772	0.17503
H	1.66344	-4.11506	1.60884
C	1.97692	-1.85882	3.14377
H	2.56163	-1.21322	3.80397
H	1.06607	-1.31622	2.87105
H	1.65291	-2.73333	3.71420
C	3.94878	-3.15387	2.50492
H	4.49846	-3.69074	1.72938
H	4.65577	-2.55081	3.08012
H	3.52847	-3.90982	3.17216
C	1.70961	2.98764	-0.46482
H	0.80231	2.39828	-0.33506
H	1.41844	4.04153	-0.43942
H	2.11248	2.78012	-1.46031
C	2.16514	2.98575	2.00647
H	1.31076	2.34471	2.23260
H	2.91587	2.83426	2.78589
H	1.81537	4.02004	2.06526
C	3.85803	3.77536	0.42255
H	4.68059	3.65014	1.13049
H	4.26644	3.77284	-0.59071
H	3.42269	4.76314	0.58922
C	7.25868	1.01353	-1.09369
H	7.35822	1.81100	-0.35291
H	8.25247	0.82361	-1.50611
H	6.62725	1.37710	-1.90844
C	6.67682	-1.34861	-1.57028
H	6.31841	-2.30554	-1.18624
H	6.02154	-1.05358	-2.39380
H	7.68020	-1.50520	-1.97584
C	7.65952	-0.71280	0.63980
H	7.34810	-1.65881	1.08705
H	8.67018	-0.84772	0.24444
H	7.70194	0.03646	1.43420
C	-2.18635	-1.83366	-1.13051
C	-2.51217	-2.65507	-2.24327
C	-2.72462	-2.12904	0.14037
C	-3.34489	-3.74416	-2.02488
C	-3.55495	-3.23402	0.27978
C	-3.87678	-4.05783	-0.78266
H	-3.58896	-4.37616	-2.86907
H	-3.96824	-3.45843	1.25758
C	-2.07022	-2.39512	-3.67249
C	-3.03040	-1.40946	-4.34074
C	-1.92721	-3.65629	-4.51706
H	-1.08946	-1.93099	-3.64487
H	-3.07750	-0.46336	-3.79901
H	-2.70095	-1.19436	-5.36061
H	-4.04094	-1.82638	-4.39109
H	-1.30344	-4.40243	-4.02083
H	-2.88907	-4.11239	-4.76802
H	-1.44427	-3.39638	-5.46185
C	-2.48512	-1.30730	1.39207
C	-1.87346	-2.14745	2.51094
C	-3.77284	-0.63287	1.86274
H	-1.77456	-0.50993	1.17272
H	-0.96544	-2.65377	2.17920
H	-1.62020	-1.51082	3.36248
H	-2.57041	-2.91046	2.86574
H	-4.20195	-0.00569	1.07993
H	-4.52261	-1.37370	2.15189
H	-3.57116	-0.00310	2.73306
C	-4.77558	-5.25529	-0.59174
C	-4.03842	-6.55955	-0.88759
C	-6.04974	-5.14431	-1.42549
H	-5.06784	-5.26857	0.46442
H	-3.14126	-6.65733	-0.27277
H	-4.68516	-7.41818	-0.68935
H	-3.73082	-6.60895	-1.93538
H	-6.59412	-4.22488	-1.19973
H	-5.82282	-5.14442	-2.49478
H	-6.71146	-5.99132	-1.22696
O	-0.56297	-0.13075	-2.68445
Pb	1.78436	-0.55147	-2.25974
Cl	1.60875	-3.02126	-2.72483
Cl	-2.25786	1.21548	-0.81818

R = Mes*

P	0.82808	1.22193	1.54125
C	0.19234	0.55664	0.16708
P	-1.40756	0.24983	-0.45924
Cl	1.79779	-2.78639	-0.37800
O	-1.21159	-0.93874	-1.37678
C	-3.05222	0.09550	0.33062
C	-3.59236	-1.22399	0.36051
C	-3.94102	1.19363	0.45452
C	-4.94962	-1.36544	0.11439
C	-5.29398	0.96284	0.21927
C	-5.81252	-0.29079	-0.05427
H	-5.35232	-2.36858	0.06071
H	-5.96455	1.80972	0.25777
C	2.62788	0.96562	1.20110
C	3.31933	-0.05202	1.89776
C	3.25289	1.61114	0.10740
C	4.47634	-0.56870	1.31969
C	4.38813	1.01419	-0.43540
C	4.98639	-0.11176	0.11429
H	4.97947	-1.38339	1.81484
H	4.83946	1.46483	-1.31103
C	2.87836	-0.58378	3.27508
C	2.86757	2.99745	-0.45637
C	6.17979	-0.76153	-0.57923
C	-3.60931	2.59796	0.99985
C	-2.84616	-2.50560	0.78603
C	-7.26987	-0.52605	-0.43178
C	4.14773	3.84952	-0.53867
H	3.88750	4.86386	-0.85198
H	4.64213	3.90927	0.43378
H	4.86880	3.46352	-1.25946
C	1.91091	3.78604	0.43717
H	0.89310	3.39176	0.42605
H	2.26106	3.82185	1.47198
H	1.84624	4.81510	0.07278
C	2.26428	2.89523	-1.85961
H	2.91869	2.33990	-2.53646
H	1.28745	2.40756	-1.83571
H	2.12094	3.89410	-2.28219
C	7.32986	0.24581	-0.69071
H	8.18619	-0.21424	-1.19188
H	7.04371	1.12887	-1.26593
H	7.65372	0.58000	0.29812
C	6.68735	-1.98936	0.17234
H	7.51999	-2.43402	-0.37819
H	7.05298	-1.73448	1.17040
H	5.90998	-2.75059	0.27194
C	5.75293	-1.20560	-1.98379
H	4.93886	-1.93282	-1.92946
H	5.41558	-0.36088	-2.58900
H	6.59390	-1.67268	-2.50413
C	3.94447	-1.48840	3.89958
H	4.91424	-0.99054	3.97608
H	3.62742	-1.75393	4.91069
H	4.07179	-2.42065	3.34467
C	2.68512	0.59695	4.23628
H	3.60838	1.17374	4.33385
H	1.89501	1.27560	3.90923
H	2.40869	0.22611	5.22735
C	1.59181	-1.41184	3.19845
H	0.71960	-0.80366	2.94815
H	1.67160	-2.19847	2.44623
H	1.39346	-1.87037	4.17198
C	-2.15796	2.79155	1.41453
H	-1.82312	1.99387	2.08107
H	-1.47824	2.85349	0.56767
H	-2.06471	3.73127	1.96499
C	-4.43064	2.74697	2.29750
H	-4.20569	3.71332	2.75716
H	-5.50537	2.70260	2.11933
H	-4.17444	1.96208	3.01322
C	-4.01807	3.71860	0.03949
H	-5.07979	3.67521	-0.21051
H	-3.83064	4.68792	0.50981
H	-3.44984	3.67638	-0.88923
C	-1.49612	-2.23312	1.44435
H	-1.13525	-3.15187	1.91276
H	-0.71982	-1.93625	0.74453
H	-1.58635	-1.47631	2.22834
C	-2.68550	-3.49245	-0.37307
H	-2.06827	-3.06971	-1.16309
H	-2.21110	-4.40928	-0.01256
H	-3.65680	-3.76205	-0.79619
C	-3.69593	-3.18129	1.88219
H	-3.87891	-2.50073	2.71759
H	-4.65962	-3.53909	1.51908
H	-3.15085	-4.04813	2.26397
C	-8.04429	0.77938	-0.59247
H	-8.10538	1.33678	0.34576
H	-7.59404	1.42636	-1.34946
H	-9.06684	0.55978	-0.90865
C	-7.95622	-1.36463	0.65301
H	-7.47813	-2.33822	0.77689
H	-7.93049	-0.85270	1.61812
H	-9.00272	-1.53833	0.38726
C	-7.31855	-1.28229	-1.76488
H	-6.84561	-0.70207	-2.56077
H	-6.80539	-2.24391	-1.70310
H	-8.35652	-1.47311	-2.05129
Pb	1.20371	-0.68090	-1.63090
Cl	-1.69114	1.82846	-1.77198

R-P=C(Pb (II)Cl)-P(O)MeR

R= H

P	0.57719	0.06487	1.08137
C	0.43195	-0.24338	-0.53713
P	-1.05550	-0.22315	-1.46700
O	-0.61193	0.07413	-2.89411
Pb	1.70355	-0.42060	-2.56975
Cl	1.33322	-2.88970	-2.69871
H	2.00296	-0.01551	1.21088
H	-1.65547	-1.49544	-1.35495
C	-2.32447	0.91035	-0.89512
H	-3.19926	0.83896	-1.54409
H	-2.60628	0.65713	0.12965
H	-1.93352	1.92828	-0.92316

R = Me

P	0.81455	0.15763	0.87749
C	0.51441	-0.22188	-0.69918
P	-1.07241	-0.25822	-1.45304
O	-0.77434	-0.12391	-2.94909
Pb	1.54519	-0.52136	-2.84917
Cl	1.32359	-3.02703	-2.76717
C	-2.18107	1.04924	-0.90422
H	-3.13210	0.97269	-1.43523
H	-2.35320	0.96908	0.17108
H	-1.72429	2.01489	-1.12341

C	-1.89022	-1.81496	-1.08704
H	-2.10950	-1.89008	-0.02041
H	-2.81544	-1.88357	-1.66339
H	-1.21847	-2.62331	-1.38288
C	2.64358	0.06240	1.06766
H	3.15189	-0.17844	0.13359
H	3.01592	1.01319	1.45940
H	2.87809	-0.70388	1.81243

R = *t*-Bu

P	0.71160	0.32899	0.23787
C	0.59668	-0.62937	-1.10041
P	-0.88212	-0.89788	-2.02446
O	-0.42580	-1.72801	-3.23005
C	2.48595	0.29745	0.85597
C	2.91407	1.76100	0.97888
H	2.99021	2.24215	0.00002
H	2.20794	2.33792	1.58270
H	3.89499	1.82355	1.46190
C	3.45871	-0.46979	-0.01782
H	3.17990	-1.52436	-0.09785
H	3.52490	-0.03327	-1.01948
H	4.46415	-0.43157	0.41587
C	2.41789	-0.33367	2.24943
H	2.13575	-1.38803	2.19722
H	3.39843	-0.26936	2.73342
H	1.69667	0.18065	2.89103
C	-2.20508	-1.78521	-1.10162
C	-2.78357	-0.89967	-0.00160
H	-3.30319	-0.02700	-0.40383
H	-3.51143	-1.47876	0.57455
H	-2.01321	-0.55307	0.69211
C	-3.29153	-2.16933	-2.10873
H	-2.88146	-2.76311	-2.92731
H	-4.05167	-2.76831	-1.59832
H	-3.79302	-1.29511	-2.53107
C	-1.60072	-3.05134	-0.49564
H	-1.14888	-3.69131	-1.25479
H	-0.83103	-2.82122	0.24257
H	-2.39404	-3.61722	0.00221
Pb	1.87280	-1.88272	-2.71449
Cl	1.84279	-4.04461	-1.42485
C	-1.56846	0.67540	-2.58495
H	-2.44364	0.50911	-3.21493
H	-1.83226	1.30715	-1.73588
H	-0.80101	1.17936	-3.17423

R = Ph

P	0.79562	0.36193	0.48366
C	0.34127	-0.22534	-0.99194
P	-1.27111	-0.27412	-1.67372
C	2.59851	0.11740	0.52708
C	3.24014	-0.95036	-0.10638
C	3.36121	1.02405	1.26457
C	4.61737	-1.08212	-0.03151
C	4.74063	0.89838	1.32699
C	5.36991	-0.15333	0.67661
H	5.10423	-1.91980	-0.51821
H	5.32502	1.61809	1.88907
C	-2.16006	-1.73103	-1.09925
C	-2.50150	-2.71500	-2.02082
C	-2.50119	-1.88585	0.24293
C	-3.19252	-3.84257	-1.60171
C	-3.19243	-3.01163	0.65593
C	-3.53984	-3.98948	-0.26788
H	-3.45260	-4.61134	-2.32001
H	-3.45487	-3.13186	1.70069
O	-1.04402	-0.33395	-3.18361
Pb	1.32285	-0.62291	-3.13483
Cl	1.29171	-3.13184	-2.97939
H	-2.21357	-2.59367	-3.05808
H	-4.07680	-4.87319	0.05792
H	-2.21228	-1.13608	0.97299
H	2.65239	-1.70107	-0.62691
H	2.86864	1.84054	1.78427
H	6.44740	-0.25900	0.73334
C	-2.28952	1.14378	-1.23148
H	-3.25653	1.06401	-1.73122
H	-2.43967	1.18226	-0.15093
H	-1.78308	2.05385	-1.55612

R = Mes

P	0.89816	0.15221	1.03172
C	0.32391	-0.26795	-0.45866
P	-1.25573	-0.19972	-1.23136
C	2.71612	0.05992	0.78737
C	3.37570	-1.17917	0.72813
C	3.45358	1.25208	0.72733
C	4.75751	-1.19308	0.57274
C	4.83280	1.18878	0.56916
C	5.50363	-0.02442	0.48175
H	5.26702	-2.15164	0.53222
H	5.39884	2.11484	0.52023
C	-2.19996	-1.74173	-1.06016
C	-2.68591	-2.42826	-2.19641
C	-2.48944	-2.24003	0.22533
C	-3.43506	-3.58271	-2.00198
C	-3.24740	-3.39791	0.35433
C	-3.72523	-4.09107	-0.74467
H	-3.80901	-4.10235	-2.87885
H	-3.46710	-3.76477	1.35264
O	-0.87795	0.12346	-2.67849
Pb	1.46811	-0.24999	-2.53495
Cl	1.33453	-2.74237	-2.90906
C	-2.32667	1.13719	-0.65384
H	-3.17842	1.19874	-1.33454
H	-2.69120	0.98467	0.36083
H	-1.76180	2.06955	-0.70314
C	2.77724	2.58978	0.81267
H	2.20719	2.81065	-0.09517
H	2.07016	2.63228	1.64685
H	3.50821	3.38813	0.94852
C	2.63498	-2.47927	0.84307
H	1.91311	-2.45685	1.66482
H	2.07557	-2.70891	-0.06825
H	3.32960	-3.30038	1.02566
C	6.98799	-0.07368	0.28390
H	7.23581	-0.16442	-0.77843
H	7.47020	0.83175	0.65649
H	7.42913	-0.93230	0.79378
C	-1.99522	-1.62332	1.50133
H	-1.00207	-2.00570	1.75287
H	-1.90824	-0.53874	1.47313
H	-2.66467	-1.87954	2.32406
C	-2.44993	-2.01438	-3.62102
H	-2.71160	-0.97390	-3.80766
H	-1.39769	-2.12733	-3.88799
H	-3.04095	-2.64883	-4.28281
C	-4.51137	-5.35525	-0.58684
H	-3.86658	-6.22872	-0.72491
H	-4.95624	-5.42764	0.40688

H	-5.31004	-5.42214	-1.32837

R = Mes*

P	0.82879	1.48234	1.26754
C	0.15935	0.63147	0.01926
P	-1.45955	0.32219	-0.61474
Cl	1.67199	-2.84891	-0.07728
O	-1.25105	-0.98729	-1.38820
C	-3.08183	0.19408	0.25944
C	-3.63270	-1.11172	0.40286
C	-3.97380	1.29703	0.30215
C	-4.99368	-1.26983	0.18050
C	-5.33101	1.05765	0.09719
C	-5.85718	-0.21192	-0.06789
H	-5.39764	-2.27353	0.21254
H	-5.99904	1.90761	0.07744
C	2.62795	1.12244	1.00267
C	3.26592	0.17884	1.83984
C	3.32212	1.63929	-0.12069
C	4.43929	-0.41617	1.38043
C	4.48278	0.98548	-0.52307
C	5.02677	-0.08803	0.17016
H	4.89841	-1.18254	1.98356
H	4.99852	1.34015	-1.40540
C	2.76682	-0.17687	3.25533
C	2.95125	2.94504	-0.86230
C	6.24919	-0.81535	-0.38155
C	-3.63076	2.73747	0.73828
C	-2.88525	-2.35548	0.92280
C	-7.31982	-0.47282	-0.40753
C	4.22596	3.65241	-1.35072
H	3.96097	4.64546	-1.72192
H	4.95295	3.77304	-0.54449
H	4.71176	3.12726	-2.17477
C	2.25397	3.94538	0.06207
H	1.26039	3.61718	0.38156
H	2.84694	4.13623	0.95950
H	2.11380	4.89371	-0.46414
C	2.07235	2.69297	-2.09112
H	2.54913	1.98494	-2.77486
H	1.09740	2.29986	-1.80384
H	1.91471	3.62785	-2.63776
C	7.41974	0.16446	-0.52156
H	8.29744	-0.35138	-0.92134
H	7.18233	0.98876	-1.19737
H	7.68865	0.59212	0.44756
C	6.69149	-1.96709	0.51736
H	7.54797	-2.47318	0.06510
H	7.00110	-1.61758	1.50566
H	5.89733	-2.70654	0.64503
C	5.90128	-1.39316	-1.75898
H	5.07536	-2.10489	-1.68350
H	5.61111	-0.61009	-2.46320
H	6.76531	-1.91617	-2.17879
C	3.78167	-1.03746	4.01368
H	4.76534	-0.56471	4.06871
H	3.42372	-1.17689	5.03635
H	3.89345	-2.03001	3.57128
C	2.59566	1.11514	4.06641
H	3.53812	1.66591	4.12339
H	1.84143	1.77820	3.63824
H	2.28003	0.87316	5.08533
C	1.45283	-0.96379	3.24384
H	0.60810	-0.36069	2.90585
H	1.51835	-1.83496	2.58984
H	1.22111	-1.30011	4.25908
C	-2.17991	2.94255	1.15139
H	-1.86963	2.20070	1.88964
H	-1.47194	2.91051	0.32627
H	-2.07210	3.92666	1.61449
C	-4.45159	3.00013	2.01823
H	-4.21318	3.99519	2.40423
H	-5.52641	2.95788	1.84080
H	-4.20824	2.26773	2.79136
C	-4.03293	3.78733	-0.30241
H	-5.09968	3.74289	-0.52980
H	-3.82057	4.78836	0.08309
H	-3.49278	3.66942	-1.24237
C	-1.54802	-2.01268	1.57050
H	-1.14484	-2.90247	2.05946
H	-0.78958	-1.69615	0.86101
H	-1.67138	-1.23841	2.33244
C	-2.70707	-3.42238	-0.16058
H	-2.08859	-3.05239	-0.97502
H	-2.22839	-4.30669	0.26901
H	-3.67461	-3.72916	-0.56760
C	-3.73529	-2.96400	2.05709
H	-3.95165	-2.22485	2.83259
H	-4.68185	-3.37936	1.70950
H	-3.17278	-3.78132	2.51530
C	-8.09528	0.81794	-0.65736
H	-8.14123	1.44660	0.23562
H	-7.65596	1.40353	-1.46896
H	-9.12290	0.57736	-0.94023
C	-7.99340	-1.22418	0.74688
H	-7.51366	-2.18558	0.93989
H	-7.95518	-0.63919	1.66909
H	-9.04327	-1.41681	0.50860
C	-7.38813	-1.32911	-1.67759
H	-6.92846	-0.81149	-2.52310
H	-6.87196	-2.28253	-1.54971
H	-8.42986	-1.54219	-1.93329
Pb	1.13139	-0.88917	-1.57129
C	-1.76499	1.55736	-1.91062
H	-2.71105	1.30804	-2.39403
H	-1.78183	2.58960	-1.57571
H	-0.96158	1.43475	-2.64012

R-P=C(Pb (II)Cl)-P(S)ClR

R= H

P	0.74871	0.04774	0.96749
C	0.51241	-0.19479	-0.65348
P	-1.01470	-0.17429	-1.46940
Cl	1.34246	-2.39598	-3.30894
S	-0.77704	0.89634	-3.12061
Pb	1.83625	-0.02934	-2.65369
Cl	-2.61637	0.40895	-0.36347
H	-1.37814	-1.50343	-1.74085
H	2.18077	-0.01977	0.97674

R = Me

P	0.85708	0.09546	0.86656
C	0.40926	-0.14225	-0.70699
P	-1.21792	-0.05270	-1.29730
Cl	1.06477	-2.44622	-3.42202
S	-1.12784	0.85769	-3.06996
Pb	1.48879	-0.02731	-2.85354
Cl	-2.51752	0.91357	-0.04756
C	-1.93483	-1.69563	-1.40454
H	-2.00721	-2.12524	-0.40437
H	-2.92301	-1.62015	-1.85971

H	-1.27399	-2.29825	-2.03309
C	2.68576	-0.10864	0.86956
H	2.95232	-0.81893	1.65732
H	3.08700	-0.46168	-0.08088
H	3.14858	0.84675	1.13478

R = *t*-Bu

P	0.79418	-0.23939	0.99108
C	0.55038	-0.37410	-0.64262
P	-1.04871	-0.28939	-1.33460
Cl	2.12840	-2.46651	-3.29289
S	-0.84605	0.33942	-3.22114
Pb	1.83257	-0.04787	-2.66087
C	2.63854	-0.43504	1.29686
C	2.79055	-0.50598	2.81750
H	2.25692	-1.36385	3.23533
H	3.84744	-0.60844	3.08255
H	2.40674	0.39448	3.30471
C	3.17796	-1.71261	0.66518
H	2.67116	-2.59746	1.05903
H	3.05926	-1.73175	-0.41980
H	4.24527	-1.81458	0.89071
C	3.37613	0.79575	0.77570
H	3.33262	0.87246	-0.31340
H	2.96921	1.72203	1.19073
H	4.43294	0.74052	1.05887
C	-1.99417	-1.88315	-1.21767
C	-2.22666	-2.24282	0.24611
H	-2.74477	-3.20533	0.28362
H	-1.28828	-2.34941	0.79399
H	-2.84537	-1.50507	0.75834
C	-1.15651	-2.96280	-1.90098
H	-0.96189	-2.72922	-2.94831
H	-0.19431	-3.11290	-1.40973
H	-1.71316	-3.90361	-1.85870
C	-3.32365	-1.70931	-1.94821
H	-3.85348	-2.66585	-1.92902
H	-3.95847	-0.96115	-1.47082
H	-3.17399	-1.42780	-2.99212
Cl	-2.25111	1.02764	-0.31575

R = Ph

P	0.78402	0.16093	0.70147
C	0.21854	0.05726	-0.85205
P	-1.45817	0.20272	-1.29328
C	2.58883	-0.01337	0.53080
C	3.24827	-0.88530	1.39721
C	3.34223	0.73113	-0.37871
C	4.62189	-1.05362	1.31591
C	4.71789	0.57616	-0.44687
C	5.35798	-0.32527	0.39260
H	5.12017	-1.75072	1.97987
H	5.29386	1.16578	-1.15122
C	-2.31243	-1.37778	-1.15393
C	-2.84874	-1.98644	-2.28101
C	-2.40940	-1.99284	0.09329
C	-3.49226	-3.20900	-2.15820
C	-3.05205	-3.21162	0.20726
C	-3.59439	-3.81941	-0.91892
H	-3.90642	-3.68682	-3.03804
H	-3.12899	-3.69136	1.17595
Cl	0.61905	-2.41693	-3.47393
S	-1.50499	0.96637	-3.12798
Pb	1.13363	0.00447	-3.07471
Cl	-2.50116	1.38942	0.00996

H	2.85190	1.46457	-1.01051
H	6.43330	-0.44910	0.33643
H	2.67941	-1.44782	2.13107
H	-4.09452	-4.77683	-0.82682
H	-2.75075	-1.50798	-3.24757
H	-1.98519	-1.51969	0.97220

R = Mes

P	0.89425	-0.84034	0.25596
C	0.34399	-0.24001	-1.18742
P	-1.33442	-0.14580	-1.68059
C	2.68921	-0.48949	0.17647
C	3.58419	-1.53631	-0.08576
C	3.17111	0.79516	0.47456
C	4.94824	-1.27173	-0.08052
C	4.54361	1.01247	0.46899
C	5.44854	-0.00403	0.18579
H	5.63894	-2.08356	-0.28977
H	4.91728	2.00450	0.70684
C	-2.46420	-1.43862	-1.07568
C	-2.05574	-2.75577	-1.40517
C	-3.65173	-1.24372	-0.33507
C	-2.78349	-3.82800	-0.90562
C	-4.33432	-2.36495	0.12415
C	-3.91273	-3.66144	-0.12206
H	-2.45325	-4.83088	-1.15718
H	-5.24421	-2.20828	0.69482
Cl	2.21253	-1.68861	-3.91736
S	-1.33557	0.04714	-3.65942
Pb	1.39308	0.57502	-3.16811
C	2.24485	1.92475	0.82280
H	1.63890	2.23731	-0.03183
H	1.54049	1.63870	1.61016
H	2.80767	2.79087	1.17350
C	3.09103	-2.91502	-0.40292
H	2.26114	-3.20765	0.24914
H	2.73082	-2.96343	-1.43529
H	3.88682	-3.65244	-0.28778
C	6.92236	0.26348	0.15515
H	7.49226	-0.61057	0.47593
H	7.25051	0.51132	-0.85928
H	7.18912	1.10296	0.79969
C	-4.29499	0.07703	-0.01792
H	-3.74189	0.63696	0.73733
H	-4.38689	0.71608	-0.89549
H	-5.29749	-0.10282	0.37092
C	-0.92453	-3.09295	-2.33143
H	-1.22134	-2.92523	-3.36971
H	-0.02166	-2.50587	-2.17380
H	-0.65887	-4.14491	-2.22213
C	-4.65132	-4.83650	0.43647
H	-5.71065	-4.61329	0.57406
H	-4.56249	-5.70869	-0.21341
H	-4.24517	-5.11429	1.41421
Cl	-1.85083	1.66595	-0.86403

R = Mes*

P	0.74522	0.93626	1.54586
C	0.16324	0.40292	0.08533
P	-1.47077	0.22155	-0.50518
Cl	1.93334	-2.81819	-0.60362
C	-3.09872	0.08853	0.35677
C	-3.66458	-1.21958	0.42708
C	-3.97027	1.20243	0.47962
C	-5.02286	-1.34279	0.17796

C	-5.32352	0.99238	0.22565
C	-5.86145	-0.25630	-0.03582
H	-5.44687	-2.33919	0.16849
H	-5.97972	1.85129	0.24946
C	2.56555	0.80541	1.25494
C	3.28936	-0.23331	1.88704
C	3.20392	1.59657	0.26805
C	4.49906	-0.62485	1.31965
C	4.39965	1.12537	-0.26667
C	5.03540	-0.01933	0.19418
H	5.02808	-1.45546	1.75784
H	4.86553	1.68936	-1.06487
C	2.83560	-0.90650	3.19659
C	2.75583	3.00822	-0.17337
C	6.29293	-0.53102	-0.50122
C	-3.62566	2.58412	1.07672
C	-2.97194	-2.48717	0.97350
C	-7.31545	-0.47127	-0.43573
C	3.99154	3.92599	-0.20857
H	3.67021	4.95322	-0.39856
H	4.52630	3.90642	0.74384
H	4.69555	3.66044	-0.99770
C	1.78942	3.66725	0.80980
H	0.79827	3.20823	0.80851
H	2.17850	3.64675	1.83078
H	1.64211	4.71310	0.52658
C	2.12869	3.00242	-1.56969
H	2.79525	2.53686	-2.30046
H	1.17546	2.47138	-1.57626
H	1.93705	4.02764	-1.90035
C	7.38432	0.54474	-0.46646
H	8.28601	0.18224	-0.96808
H	7.07012	1.46115	-0.97037
H	7.64760	0.80116	0.56275
C	6.84280	-1.79505	0.15443
H	7.72274	-2.13664	-0.39613
H	7.15089	-1.61759	1.18807
H	6.10977	-2.60514	0.14559
C	5.94780	-0.86112	-1.95875
H	5.17768	-1.63496	-2.00868
H	5.58178	0.01738	-2.49519
H	6.83479	-1.22718	-2.48360
C	3.91881	-1.82599	3.76705
H	4.86458	-1.30194	3.92506
H	3.58215	-2.20053	4.73647
H	4.10056	-2.69386	3.12903
C	2.57443	0.17734	4.25173
H	3.47212	0.77643	4.42453
H	1.76731	0.85278	3.96141
H	2.28827	-0.28880	5.19886
C	1.58318	-1.76656	3.01334
H	0.69710	-1.16393	2.80219
H	1.70362	-2.47566	2.19283
H	1.37910	-2.31872	3.93582
C	-2.17906	2.74145	1.52560
H	-1.86967	1.91576	2.16971
H	-1.48075	2.82129	0.69633
H	-2.08518	3.66037	2.11015
C	-4.46423	2.68351	2.36966
H	-4.23038	3.62320	2.87761
H	-5.53655	2.66513	2.17350
H	-4.23082	1.86144	3.05052
C	-4.01544	3.75226	0.16723
H	-5.07335	3.72503	-0.10071
H	-3.83431	4.69542	0.69010
H	-3.43371	3.75847	-0.75351
C	-1.53021	-2.28470	1.42235
H	-1.21527	-3.15691	2.00054
H	-0.82408	-2.19131	0.59953
H	-1.44165	-1.41238	2.07451
C	-3.04692	-3.67806	0.01601
H	-2.47203	-3.49212	-0.89024
H	-2.63302	-4.56329	0.50617
H	-4.07533	-3.90978	-0.26941
C	-3.75389	-2.84621	2.25611
H	-3.73320	-2.02025	2.97160
H	-4.79719	-3.09244	2.05562
H	-3.28727	-3.71564	2.72680
C	-8.06037	0.84549	-0.63773
H	-8.13106	1.42180	0.28835
H	-7.58084	1.46897	-1.39641
H	-9.07989	0.63975	-0.97237
C	-8.03968	-1.27480	0.65099
H	-7.58602	-2.25638	0.80127
H	-8.02163	-0.74574	1.60701
H	-9.08433	-1.43117	0.36800
C	-7.35129	-1.25349	-1.75426
H	-6.85124	-0.69868	-2.55164
H	-6.85702	-2.22257	-1.66209
H	-8.38661	-1.43106	-2.05818
Pb	1.33865	-0.63325	-1.74063
S	-1.38011	-1.22332	-1.88202
Cl	-1.72023	1.93682	-1.64758

R-P=C(Pb (II)Cl)-P(S)MeR

R= H

P	0.79861	0.14024	0.92090
C	0.57712	-0.11003	-0.70244
P	-0.96794	0.06825	-1.51186
Cl	1.13412	-2.53069	-3.18031
S	-0.54924	1.00005	-3.23155
Pb	1.92382	-0.19977	-2.67023
H	-1.44158	-1.23828	-1.74369
H	2.21951	-0.03976	0.98483
C	-2.29790	0.85387	-0.58169
H	-3.20337	0.86568	-1.19090
H	-2.48028	0.29559	0.33935
H	-2.00818	1.87714	-0.34111

R = Me

P	0.83802	0.16745	0.73298
C	0.46929	-0.09996	-0.85651
P	-1.16420	0.04982	-1.48109
Cl	1.12237	-2.66916	-3.28127
S	-0.94247	0.73910	-3.35907
Pb	1.62251	-0.21327	-2.94781
C	-1.93920	-1.57735	-1.46990
H	-2.06955	-1.91631	-0.43988
H	-2.90574	-1.51949	-1.97386
H	-1.28505	-2.26691	-2.00735
C	2.65883	-0.07042	0.85725
H	2.86192	-0.81153	1.63543
H	3.11050	-0.40168	-0.07859
H	3.12239	0.86653	1.17936
C	-2.25609	1.12380	-0.52210
H	-3.23905	1.15335	-0.99608
H	-2.34945	0.74444	0.49779
H	-1.83552	2.12903	-0.50019

R = *t*-Bu

P	0.77519	-0.32421	0.95824

C	0.54329	-0.35273	-0.68195
P	-1.08895	-0.24904	-1.34722
Cl	2.15488	-2.43787	-3.27905
S	-0.86274	0.31285	-3.27332
Pb	1.81062	-0.00590	-2.69559
C	-2.08973	0.98379	-0.47499
H	-3.08243	1.04922	-0.92204
H	-2.16700	0.73224	0.58378
H	-1.58938	1.94645	-0.58057
C	2.62489	-0.44221	1.27854
C	2.77546	-0.49504	2.79946
H	2.25929	-1.36012	3.22429
H	3.83374	-0.57158	3.06820
H	2.37104	0.40162	3.27727
C	3.19312	-1.71447	0.65926
H	2.70552	-2.60633	1.06205
H	3.07331	-1.74449	-0.42544
H	4.26236	-1.79109	0.88647
C	3.33648	0.79686	0.74476
H	3.28800	0.86228	-0.34459
H	2.91183	1.71819	1.15292
H	4.39539	0.76562	1.02430
C	-2.00616	-1.86205	-1.22920
C	-2.30854	-2.17618	0.23387
H	-2.77500	-3.16415	0.28881
H	-1.40283	-2.20368	0.84392
H	-3.00327	-1.46007	0.67781
C	-1.13636	-2.96006	-1.83402
H	-0.88281	-2.75168	-2.87432
H	-0.20136	-3.09014	-1.28743
H	-1.68899	-3.90371	-1.79853
C	-3.30638	-1.73640	-2.02221
H	-3.82350	-2.70004	-1.99798
H	-3.98328	-0.99088	-1.59825
H	-3.11564	-1.48120	-3.06606

R = Ph

P	0.78904	-0.52126	0.40729
C	0.22750	0.09277	-1.02558
P	-1.49547	0.20393	-1.37929
C	2.59729	-0.36650	0.28535
C	3.37284	-1.38676	0.83691
C	3.23909	0.73299	-0.28851
C	4.75652	-1.33540	0.76973
C	4.62216	0.79226	-0.34056
C	5.38238	-0.24713	0.17966
H	5.34799	-2.14445	1.18287
H	5.11081	1.65397	-0.78138
C	-2.36875	-1.34947	-1.07576
C	-1.97163	-2.47181	-1.80363
C	-3.39932	-1.45644	-0.14622
C	-2.60251	-3.68552	-1.59548
C	-4.02990	-2.67609	0.05413
C	-3.63216	-3.78938	-0.66882
H	-2.28881	-4.55457	-2.16200
H	-4.83368	-2.75319	0.77722
Cl	1.27534	-1.91385	-3.74520
S	-1.61754	0.74869	-3.31148
Pb	1.14305	0.54662	-3.18277
H	2.64653	1.55976	-0.66632
H	6.46452	-0.20177	0.13532
H	2.88603	-2.23512	1.30797
H	-4.12571	-4.74171	-0.51117
H	-1.16664	-2.39445	-2.52874
H	-3.72134	-0.59535	0.42654
C	-2.26935	1.45261	-0.32600
H	-1.73267	2.38781	-0.48614
H	-3.31358	1.58877	-0.61176
H	-2.19222	1.16790	0.72490

R = Mes

P	0.86270	-0.67272	0.30732
C	0.31898	-0.22374	-1.19100
P	-1.38536	-0.10229	-1.65174
C	2.67216	-0.39787	0.20295
C	3.52273	-1.48510	-0.04291
C	3.20975	0.87038	0.46972
C	4.89663	-1.27594	-0.04970
C	4.58967	1.03322	0.45299
C	5.45063	-0.02498	0.18767
H	5.55223	-2.11987	-0.24466
H	5.00431	2.01496	0.66448
C	-2.45603	-1.46090	-1.06922
C	-2.04595	-2.76887	-1.42345
C	-3.63559	-1.29278	-0.31145
C	-2.77285	-3.85524	-0.95373
C	-4.32019	-2.42243	0.12322
C	-3.90259	-3.71155	-0.16566
H	-2.44004	-4.85103	-1.22928
H	-5.22418	-2.28112	0.70742
Cl	2.14944	-1.83145	-3.92189
S	-1.37577	0.07878	-3.66218
Pb	1.35442	0.46426	-3.22989
C	-1.85554	1.51021	-0.96759
H	-1.07166	2.18771	-1.31063
H	-2.80817	1.85711	-1.36125
H	-1.85607	1.49758	0.12195
C	2.33142	2.04745	0.78214
H	1.77190	2.37909	-0.09734
H	1.59081	1.80513	1.55071
H	2.92525	2.88984	1.13976
C	2.97509	-2.85086	-0.32515
H	2.13456	-3.09420	0.33318
H	2.61319	-2.91210	-1.35629
H	3.74193	-3.61535	-0.19105
C	6.93416	0.18056	0.14391
H	7.46912	-0.70534	0.49145
H	7.26813	0.38144	-0.87897
H	7.23797	1.02869	0.76015
C	-4.26380	0.02431	0.04969
H	-3.66005	0.60416	0.74881
H	-4.45245	0.64375	-0.82796
H	-5.22564	-0.15534	0.52962
C	-0.89350	-3.06757	-2.33155
H	-1.12793	-2.78932	-3.36146
H	0.01614	-2.52763	-2.07216
H	-0.66531	-4.13353	-2.31030
C	-4.64122	-4.90283	0.35815
H	-5.69342	-4.67410	0.53561
H	-4.58217	-5.74388	-0.33493
H	-4.21190	-5.23309	1.30933

R = Mes*

P	0.76078	1.19455	1.35349
C	0.16098	0.47638	-0.01505
P	-1.48978	0.29984	-0.61429
Cl	1.83905	-2.89642	-0.26129
C	-3.10703	0.22796	0.29657
C	-3.68662	-1.05472	0.51610
C	-3.97532	1.35187	0.29244
C	-5.04883	-1.19597	0.29114

C	-5.33232	1.12969	0.06871
C	-5.88257	-0.13664	-0.04072
H	-5.47765	-2.18508	0.39487
H	-5.98209	1.99118	-0.00301
C	2.58571	1.00069	1.10483
C	3.28400	0.03690	1.86984
C	3.26479	1.68482	0.06384
C	4.51132	-0.41538	1.39043
C	4.48528	1.17029	-0.36189
C	5.09727	0.07698	0.23621
H	5.01835	-1.19761	1.93159
H	4.99142	1.65283	-1.18714
C	2.79704	-0.47681	3.23976
C	2.81350	3.02902	-0.55410
C	6.38543	-0.49439	-0.34788
C	-3.61065	2.79108	0.72011
C	-2.99680	-2.26058	1.18685
C	-7.34229	-0.38482	-0.39988
C	4.04272	3.88181	-0.90991
H	3.71016	4.88528	-1.18676
H	4.72853	3.97143	-0.06452
H	4.59863	3.48791	-1.76221
C	2.00638	3.86972	0.43729
H	1.03379	3.43338	0.68215
H	2.55279	4.01272	1.37244
H	1.80580	4.85369	0.00428
C	2.00470	2.83895	-1.84014
H	2.56538	2.24921	-2.57052
H	1.05969	2.33562	-1.63933
H	1.78544	3.81073	-2.29336
C	7.47529	0.58352	-0.36236
H	8.39969	0.17891	-0.78424
H	7.18602	1.44765	-0.96408
H	7.68912	0.93592	0.64982
C	6.90221	-1.68907	0.44933
H	7.80569	-2.07980	-0.02531
H	7.16299	-1.41326	1.47436
H	6.16870	-2.49803	0.48489
C	6.11160	-0.96109	-1.78296
H	5.34500	-1.73978	-1.79775
H	5.77088	-0.13880	-2.41617
H	7.02302	-1.37056	-2.22788
C	3.85443	-1.34217	3.93094
H	4.80552	-0.81761	4.05070
H	3.49250	-1.60182	4.92844
H	4.03403	-2.27806	3.39678
C	2.54395	0.72770	4.15774
H	3.45090	1.32691	4.27267
H	1.75377	1.37836	3.77793
H	2.23709	0.38011	5.14841
C	1.53087	-1.33015	3.14356
H	0.65863	-0.74183	2.85308
H	1.64517	-2.13370	2.41435
H	1.30889	-1.76604	4.12253
C	-2.17663	2.95659	1.20884
H	-1.94056	2.23112	1.98951
H	-1.42037	2.86356	0.43356
H	-2.05757	3.95336	1.64132
C	-4.48208	3.09120	1.95911
H	-4.21963	4.07646	2.35439
H	-5.54761	3.09600	1.72925
H	-4.30876	2.35196	2.74435
C	-3.94229	3.84249	-0.34338
H	-4.99615	3.80633	-0.62634
H	-3.74321	4.84183	0.05330
H	-3.35447	3.72159	-1.25300
C	-1.54976	-2.01440	1.59021
H	-1.22090	-2.82479	2.24532
H	-0.86139	-2.00216	0.74767
H	-1.45300	-1.08059	2.14889
C	-3.08834	-3.54594	0.36168
H	-2.52802	-3.45639	-0.56787
H	-2.66675	-4.37516	0.93606
H	-4.12188	-3.80427	0.12007
C	-3.76452	-2.47709	2.50883
H	-3.73245	-1.57988	3.13235
H	-4.81111	-2.73896	2.34793
H	-3.29495	-3.29355	3.06392
C	-8.08222	0.90660	-0.73816
H	-8.13657	1.58314	0.11867
H	-7.60983	1.43811	-1.56814
H	-9.10753	0.67278	-1.03458
C	-8.05957	-1.05781	0.77635
H	-7.60908	-2.01918	1.03042
H	-8.02836	-0.42590	1.66736
H	-9.10801	-1.23824	0.52310
C	-7.39840	-1.30790	-1.62301
H	-6.90583	-0.84789	-2.48305
H	-6.90699	-2.26335	-1.42973
H	-8.43812	-1.51219	-1.89353
Pb	1.31043	-0.85254	-1.66332
S	-1.39969	-1.33777	-1.81281
C	-1.71781	1.63385	-1.83583
H	-1.62606	2.63771	-1.43276
H	-0.94884	1.47723	-2.59245
H	-2.69326	1.49862	-2.30417

PCPX-E(II)-AuCl model systems

E = Si(II)

[R-P=C(Si(II)Cl)-P(O)ClR](AuCl)

Coordination through Si(II) atom

R= H

P	0.80407	-0.10260	1.11290
C	0.50080	-0.30589	-0.50484
P	-1.00073	-0.32247	-1.38538
Cl	1.69669	-2.37389	-2.52939
Si	1.37961	-0.34670	-2.24607
O	-0.37130	-0.21508	-2.78862
H	2.22854	-0.13774	1.02508
H	-1.79496	-1.47465	-1.26978
Cl	-2.31334	1.12054	-1.03045
Au	2.83535	1.22136	-2.88237
Cl	4.31876	2.85901	-3.47553

R = Me

P	0.82454	-0.12224	1.05780
C	0.48027	-0.32854	-0.55090
P	-1.03768	-0.35138	-1.39582
Cl	1.67504	-2.33087	-2.66395
Si	1.31439	-0.31188	-2.29766
O	-0.42140	-0.18977	-2.81182
Cl	-2.29254	1.15329	-1.00619
Au	2.79295	1.25840	-2.89514
Cl	4.31676	2.89056	-3.41386
C	2.65008	-0.15651	1.09402
H	2.98031	-1.20036	1.10023
H	3.08082	0.33854	0.22003

H	3.00592	0.31765	2.00948
C	-2.03198	-1.82570	-1.28546
H	-2.44352	-1.92052	-0.27969
H	-2.84048	-1.77008	-2.01651
H	-1.38154	-2.67362	-1.51048

R = *t*-Bu

P	0.76569	-0.03962	0.84767
C	0.55800	-0.42678	-0.75353
P	-0.98118	-0.57348	-1.57706
Cl	1.99969	-2.56584	-2.65315
Si	1.37098	-0.58957	-2.50912
O	-0.36383	-0.74572	-2.99486
Au	2.59243	1.07478	-3.38614
Cl	3.83575	2.82682	-4.19736
C	2.58651	0.13779	1.17507
C	3.49742	-0.63361	0.23544
C	2.84616	1.64711	1.06486
C	2.79271	-0.31895	2.62169
H	3.26333	-1.69980	0.21596
H	3.44936	-0.24727	-0.78546
H	4.53517	-0.52266	0.56647
H	2.17944	2.23007	1.70761
H	3.87252	1.85321	1.38539
H	2.73715	2.00096	0.03737
H	3.82356	-0.11195	2.92363
H	2.12949	0.20610	3.31464
H	2.62169	-1.39272	2.73532
C	-2.13852	-1.92498	-1.17130
C	-3.23135	-1.96088	-2.24050
C	-1.32749	-3.22338	-1.19621
C	-2.72517	-1.68413	0.21800
H	-3.84354	-1.05801	-2.23067
H	-2.81340	-2.09088	-3.24051
H	-3.88409	-2.81323	-2.03389
H	-0.53183	-3.22440	-0.44947
H	-2.00650	-4.04959	-0.96988
H	-0.87951	-3.40750	-2.17330
H	-3.36195	-2.53461	0.47508
H	-1.94840	-1.60798	0.98312
H	-3.33738	-0.78195	0.25358
Cl	-2.06603	1.11520	-1.53047

R = Ph

P	0.93879	-0.18396	0.70106
C	0.53424	-0.29240	-0.91017
P	-1.09367	-0.42231	-1.52120
Cl	1.67461	-2.40355	-2.95525
Si	1.10730	-0.41007	-2.75416
O	-0.67633	-0.53517	-3.01338
Au	2.27660	1.18478	-3.81122
Cl	3.50922	2.83124	-4.83190
C	-2.12875	-1.78760	-1.03988
C	-1.95913	-2.99210	-1.72317
C	-3.02002	-1.69629	0.02790
C	-2.69475	-4.10017	-1.33729
H	-1.26149	-3.05659	-2.54964
C	-3.74319	-2.81323	0.40800
H	-3.15545	-0.75820	0.55253
C	-3.58199	-4.01163	-0.27443
H	-2.57397	-5.03504	-1.87125
H	-4.43986	-2.74633	1.23498
H	-4.15532	-4.88200	0.02324
C	2.72270	-0.09233	0.81766
C	3.22483	-0.23745	2.11762
C	3.61267	0.13968	-0.23659
C	4.58744	-0.18040	2.35470
H	2.53659	-0.39851	2.94179
C	4.97041	0.21105	0.00656
H	3.24704	0.29440	-1.24624
C	5.45859	0.04392	1.29863
H	4.96919	-0.30242	3.36144
H	5.65172	0.40283	-0.81391
H	6.52586	0.09796	1.48133
Cl	-2.19706	1.22262	-1.22782

R = Mes

P	0.88492	0.52359	0.43753
C	0.61208	-0.55016	-0.80048
P	-0.79120	-0.94799	-1.77807
Cl	1.92308	-3.37468	-1.45422
Si	1.67504	-1.42166	-2.15124
O	0.05429	-1.55069	-2.93643
Au	3.34454	-0.38296	-3.22925
Cl	5.04133	0.78597	-4.25397
C	-2.04278	-2.06314	-1.16229
C	-2.28611	-3.31349	-1.77471
C	-2.77131	-1.66879	-0.01971
C	-3.22732	-4.14659	-1.18259
C	-3.68892	-2.55365	0.52352
C	-3.92562	-3.80236	-0.03410
H	-3.42049	-5.10787	-1.64751
H	-4.24182	-2.24976	1.40662
C	2.67832	0.41568	0.69298
C	3.22976	-0.62090	1.45892
C	3.48476	1.44324	0.18306
C	4.60191	-0.62448	1.66885
C	4.85173	1.39029	0.41274
C	5.43088	0.36009	1.14303
H	5.03675	-1.42207	2.26396
H	5.48176	2.17066	-0.00290
C	2.37727	-1.70342	2.05168
H	1.49064	-1.29546	2.54862
H	2.02793	-2.40073	1.28539
H	2.93813	-2.27431	2.79268
C	2.90238	2.55069	-0.64020
H	2.77278	2.23166	-1.67942
H	1.92115	2.86725	-0.26633
H	3.55573	3.42380	-0.64466
C	6.91407	0.30412	1.33826
H	7.40048	-0.09627	0.44359
H	7.33016	1.29802	1.51475
H	7.18339	-0.33758	2.17852
C	-2.64196	-0.31619	0.61043
H	-1.60639	0.01365	0.72179
H	-3.15825	0.44187	0.01549
H	-3.08788	-0.31886	1.60509
C	-1.62192	-3.81172	-3.02472
H	-1.68705	-3.09595	-3.84402
H	-0.56201	-4.01496	-2.86430
H	-2.10139	-4.73778	-3.34126
C	-4.89526	-4.75398	0.59095
H	-5.69616	-4.22483	1.10965
H	-5.34028	-5.41607	-0.15320
H	-4.38754	-5.38395	1.32809
Cl	-1.75350	0.65949	-2.51627

R = Mes *

P	-0.57086	1.64882	1.52737
C	0.08893	0.57309	0.43669
P	1.68449	-0.13496	0.19918
Cl	-0.97120	0.54341	-2.64108

O	1.26660	-0.83898	-1.13079
C	3.39257	0.45935	0.07751
C	3.97455	0.54943	-1.22286
C	4.24692	0.37500	1.20778
C	5.29586	0.14977	-1.34774
C	5.56988	0.00853	0.99157
C	6.09389	-0.21685	-0.27091
H	5.73003	0.14764	-2.33855
H	6.21285	-0.09863	1.85380
C	-2.33436	1.60591	1.04066
C	-2.82573	2.57239	0.13455
C	-3.17033	0.54577	1.47611
C	-4.02593	2.29449	-0.51602
C	-4.34610	0.33129	0.77085
C	-4.76218	1.14552	-0.27500
H	-4.38999	2.99531	-1.25013
H	-4.96062	-0.51485	1.03980
C	-2.16154	3.94772	-0.08289
C	-2.96273	-0.21797	2.79883
C	-6.00566	0.77434	-1.07526
C	3.90846	0.83193	2.63900
C	3.33022	1.20328	-2.46046
C	7.50388	-0.74286	-0.50364
C	-3.92949	-1.39668	2.94575
H	-3.72673	-1.90072	3.89349
H	-4.97154	-1.07200	2.97609
H	-3.81059	-2.13184	2.14573
C	-3.26524	0.78532	3.92459
H	-2.56717	1.62708	3.90915
H	-4.27681	1.18641	3.82885
H	-3.18236	0.29273	4.89782
C	-1.55959	-0.77598	3.02505
H	-1.23147	-1.41534	2.20407
H	-0.80900	0.01148	3.17495
H	-1.55589	-1.36509	3.94629
C	-7.21455	0.65888	-0.13937
H	-8.10672	0.39381	-0.71331
H	-7.07013	-0.11235	0.61930
H	-7.40755	1.60557	0.37213
C	-6.33215	1.80680	-2.15094
H	-7.21481	1.48340	-2.70775
H	-6.55452	2.78839	-1.72330
H	-5.51408	1.91831	-2.86697
C	-5.75975	-0.57687	-1.75907
H	-4.91496	-0.51668	-2.44930
H	-5.53917	-1.36807	-1.04001
H	-6.64511	-0.87485	-2.32795
C	-3.01865	4.85827	-0.96620
H	-4.02306	4.99786	-0.56096
H	-2.54453	5.84099	-1.02031
H	-3.10430	4.48003	-1.98752
C	-2.02904	4.64693	1.27732
H	-3.00515	4.75523	1.75567
H	-1.37853	4.09951	1.96427
H	-1.59891	5.64388	1.14528
C	-0.78531	3.86814	-0.74886
H	-0.02792	3.42223	-0.09926
H	-0.81893	3.28248	-1.66867
H	-0.43768	4.87731	-0.98872
C	2.48638	1.34405	2.81708
H	2.22591	2.08720	2.05926
H	1.74151	0.54900	2.81463
H	2.39883	1.83797	3.78757
C	4.82692	2.04147	2.91024
H	4.61251	2.44030	3.90526
H	5.88356	1.77488	2.87534
H	4.65420	2.83608	2.18039
C	4.19581	-0.24153	3.69208
H	5.23452	-0.57498	3.66448
H	4.00979	0.16808	4.68832
H	3.55677	-1.11450	3.56127
C	2.16849	2.11504	-2.07327
H	1.78618	2.61523	-2.96593
H	1.31764	1.59714	-1.63930
H	2.50041	2.88514	-1.37176
C	2.92294	0.18408	-3.52880
H	2.13704	-0.48282	-3.18607
H	2.56474	0.71079	-4.41721
H	3.78265	-0.42267	-3.82481
C	4.37450	2.14227	-3.09809
H	4.79901	2.83076	-2.36374
H	5.19231	1.60622	-3.58075
H	3.88053	2.73401	-3.87252
C	8.20649	-1.10962	0.80075
H	8.35962	-0.23840	1.44281
H	7.64849	-1.86145	1.36437
H	9.19132	-1.52635	0.57821
C	8.34028	0.32317	-1.22188
H	7.91712	0.58767	-2.19280
H	8.40997	1.23572	-0.62478
H	9.35370	-0.05017	-1.39135
C	7.42086	-1.99982	-1.37846
H	6.83767	-2.78212	-0.88706
H	6.95566	-1.79213	-2.34401
H	8.42426	-2.39039	-1.56721
Au	-1.84543	-2.37137	-0.56216
Cl	-3.29839	-4.09862	-0.06114
Si	-0.51494	-0.60301	-0.96522
Cl	1.86216	-1.68829	1.49720

Coordination through O atom

R= H

P	0.68754	-0.15581	1.15403
C	0.43909	-0.31608	-0.47915
P	-1.02055	-0.12697	-1.38122
Cl	1.15080	-2.49278	-2.52264
Si	1.50603	-0.43793	-2.15362
O	-0.36606	0.18671	-2.74918
H	2.09225	-0.42595	1.14571
H	-1.80826	-1.28441	-1.45998
Cl	-2.33763	1.25860	-0.83945
Au	-1.44027	0.28583	-4.57949
Cl	-2.59636	0.38737	-6.47005

R = Me

P	0.67263	-0.18041	1.09828
C	0.41603	-0.30856	-0.53571
P	-1.05964	-0.15948	-1.41429
Cl	1.36178	-2.39356	-2.63029
Si	1.46371	-0.30744	-2.21448
O	-0.39866	0.13043	-2.79982
Cl	-2.28473	1.34444	-0.91938
Au	-1.42885	0.21114	-4.65056
Cl	-2.54662	0.28742	-6.56721
C	2.46628	-0.51910	1.25565
H	2.84036	-0.09053	2.18641
H	2.61619	-1.60244	1.29637
H	3.03223	-0.12511	0.40805
C	-2.09106	-1.60886	-1.47780
H	-2.88214	-1.45457	-2.21446
H	-1.45545	-2.44559	-1.77608

H	-2.52244	-1.78900	-0.49179

R = *t*-Bu

P	0.81298	-0.33896	0.88213
C	0.43413	-0.19882	-0.73109
P	-1.19134	-0.29069	-1.35024
Cl	1.77440	-1.56249	-3.20515
Si	1.14071	0.34838	-2.50655
O	-0.82221	0.08063	-2.82118
Au	-2.01945	0.44993	-4.52668
Cl	-3.27989	0.82397	-6.31724
C	2.66798	-0.26596	1.05175
C	3.10524	-1.71694	1.28446
C	3.40412	0.33110	-0.13358
C	2.93005	0.55867	2.31553
H	2.56858	-2.17928	2.11820
H	2.95209	-2.32877	0.39264
H	4.17237	-1.73609	1.52982
H	3.05766	1.34051	-0.36653
H	4.47437	0.39341	0.09114
H	3.30491	-0.29082	-1.02704
H	3.99819	0.53505	2.55161
H	2.63981	1.60410	2.18330
H	2.38782	0.16386	3.17897
C	-2.12379	-1.86110	-1.22351
C	-3.45857	-1.73407	-1.95818
C	-1.26005	-2.94611	-1.87096
C	-2.35208	-2.16486	0.25693
H	-4.10837	-0.98508	-1.50416
H	-3.32871	-1.48670	-3.01441
H	-3.96584	-2.70101	-1.90100
H	-0.29164	-3.05234	-1.37979
H	-1.79391	-3.89597	-1.78313
H	-1.08513	-2.75091	-2.92957
H	-2.86425	-3.12785	0.32916
H	-1.41523	-2.24314	0.81305
H	-2.98087	-1.41351	0.73749
Cl	-2.40998	1.13193	-0.61718

R = Ph

P	0.86686	-0.38279	1.11932
C	0.41255	-0.35394	-0.48444
P	-1.20409	-0.29234	-1.07620
Cl	1.35101	-1.67007	-3.18668
Si	1.12206	0.22610	-2.23484
O	-0.84738	0.35558	-2.45783
Au	-2.21078	0.75282	-4.02709
Cl	-3.67947	1.16569	-5.64350
C	2.66276	-0.42540	1.08917
C	3.29756	-0.00964	2.26580
C	3.44334	-0.83442	0.00220
C	4.67877	0.03554	2.34062
H	2.69790	0.29103	3.11953
C	4.82390	-0.79492	0.08380
H	2.97136	-1.22437	-0.89284
C	5.44126	-0.35388	1.24787
H	5.16209	0.37019	3.25098
H	5.42208	-1.11548	-0.76109
H	6.52335	-0.32530	1.30660
C	-2.06292	-1.84157	-1.24450
C	-3.39881	-1.86953	-1.64796
C	-1.36095	-3.02644	-1.03146
C	-4.02533	-3.08755	-1.83704
H	-3.94243	-0.94834	-1.82220
C	-2.00271	-4.24004	-1.21611
H	-0.31999	-2.99648	-0.73518
C	-3.32941	-4.26960	-1.61824
H	-5.05927	-3.11362	-2.15889
H	-1.46126	-5.16368	-1.05096
H	-3.82671	-5.22136	-1.76591
Cl	-2.48109	0.93364	-0.13140

R = Mes

P	1.19827	-1.31270	1.37492
C	0.27535	-0.73093	0.12291
P	-1.42967	-0.69968	-0.19785
Cl	0.81560	-0.44804	-2.93785
Si	0.61945	0.72509	-1.15946
O	-1.38680	0.61227	-1.04663
Au	-2.67739	2.30984	-1.09913
Cl	-4.00278	4.09258	-1.16585
C	2.86378	-0.71464	0.93351
C	3.36821	0.41582	1.59198
C	3.66466	-1.43479	0.03475
C	4.66621	0.82531	1.31809
C	4.95789	-0.98918	-0.20354
C	5.47519	0.14052	0.42007
H	5.05576	1.70430	1.82323
H	5.58307	-1.54687	-0.89470
C	-2.14641	-2.09699	-1.05313
C	-2.80313	-1.96542	-2.29831
C	-2.03288	-3.36020	-0.43380
C	-3.30212	-3.11838	-2.88934
C	-2.55834	-4.46914	-1.07985
C	-3.18777	-4.37507	-2.31158
H	-3.81194	-3.02146	-3.84231
H	-2.47568	-5.43687	-0.59533
C	2.51696	1.19661	2.54699
H	1.76729	1.78717	2.01087
H	1.97635	0.54168	3.23948
H	3.12014	1.88449	3.14057
C	6.86186	0.61743	0.11587
H	6.85555	1.29705	-0.74205
H	7.29104	1.16038	0.95962
H	7.52423	-0.21394	-0.13189
C	3.15640	-2.66279	-0.66010
H	2.69422	-3.36271	0.04408
H	2.40179	-2.41009	-1.41023
H	3.96911	-3.18751	-1.16365
C	-1.35846	-3.58642	0.88534
H	-0.27520	-3.67209	0.76095
H	-1.53912	-2.79239	1.61077
H	-1.71176	-4.51796	1.32855
C	-3.01688	-0.67596	-3.03117
H	-2.07212	-0.22576	-3.33813
H	-3.61209	-0.85988	-3.92509
H	-3.54715	0.06361	-2.42633
C	-3.71844	-5.58943	-3.00457
H	-2.98567	-5.96734	-3.72437
H	-3.93027	-6.39276	-2.29770
H	-4.63117	-5.36391	-3.55867
Cl	-2.61132	-0.32193	1.39631

R = Mes*

P	2.08237	1.96568	-0.28892
C	1.08578	0.63266	-0.25310
P	-0.64875	0.48119	-0.37291
C	3.73676	1.16585	-0.24052
C	4.45456	1.15274	0.98779
C	4.23307	0.44908	-1.34766
C	5.48555	0.23842	1.12263
C	5.26818	-0.46290	-1.12441

C	5.86974	-0.63263	0.10579
H	6.01383	0.18599	2.06318
H	5.61416	-1.05209	-1.96208
C	3.86194	0.66466	-2.82982
C	4.18665	2.14542	2.13533
C	6.94535	-1.68069	0.36869
C	2.82492	1.92377	2.79835
H	1.99066	2.16576	2.13497
H	2.70100	0.88645	3.11420
H	2.73121	2.57288	3.67399
C	4.27763	3.57879	1.59389
H	5.26083	3.76507	1.15521
H	3.52396	3.78790	0.83158
H	4.12530	4.29410	2.40701
C	5.23427	2.02994	3.24577
H	5.18756	1.06682	3.75934
H	6.24966	2.17826	2.87118
H	5.04066	2.80499	3.99077
C	3.37048	-0.63259	-3.47959
H	2.46415	-1.00204	-2.99496
H	3.14898	-0.45656	-4.53600
H	4.11846	-1.42551	-3.42712
C	2.82423	1.75150	-3.09786
H	1.81391	1.44941	-2.82099
H	3.07276	2.69384	-2.59761
H	2.80349	1.96429	-4.17070
C	5.14568	1.12709	-3.54468
H	5.51321	2.06425	-3.11860
H	5.94712	0.39134	-3.47486
H	4.93589	1.29316	-4.60490
C	7.22288	-2.54550	-0.85782
H	7.60989	-1.95697	-1.69383
H	7.97645	-3.29687	-0.61035
H	6.32668	-3.07395	-1.19213
C	6.47621	-2.59651	1.50610
H	6.30729	-2.03958	2.42987
H	5.54137	-3.09768	1.24488
H	7.23141	-3.36103	1.70875
C	8.25173	-0.99022	0.77733
H	8.12919	-0.39057	1.68154
H	9.02503	-1.73740	0.97625
H	8.61156	-0.33138	-0.01699
C	-2.10731	1.30370	0.29904
C	-2.72087	0.68486	1.42925
C	-2.88873	2.14927	-0.53105
C	-4.10294	0.59474	1.42135
C	-4.26926	2.02133	-0.45504
C	-4.89963	1.16052	0.43274
H	-4.58006	0.06143	2.23346
H	-4.87322	2.62564	-1.11773
C	-2.00174	0.28133	2.72986
C	-2.04753	-1.22211	3.00945
C	-2.73950	1.00298	3.87639
H	-1.42808	-1.78471	2.31257
H	-1.67443	-1.41522	4.01886
H	-3.06640	-1.61004	2.94737
H	-2.79159	2.08014	3.70032
H	-3.75413	0.63271	4.02465
H	-2.19067	0.83593	4.80661
C	-2.35422	3.33693	-1.35312
C	-2.94332	4.59002	-0.67144
C	-2.81959	3.31996	-2.81108
H	-2.63858	4.64607	0.37635
H	-2.57590	5.48438	-1.18187
H	-4.03297	4.60423	-0.70742
H	-2.41434	2.46747	-3.35518
H	-3.90736	3.28505	-2.89145
H	-2.48312	4.23225	-3.31066
C	-6.39869	0.89993	0.41482
C	-7.03004	1.43745	1.70502
C	-6.62948	-0.61397	0.32365
H	-6.87628	2.51541	1.80079
H	-8.10644	1.24657	1.69873
H	-6.61353	0.95657	2.59224
H	-6.17860	-1.03510	-0.57723
H	-6.20203	-1.14437	1.17630
H	-7.70225	-0.82367	0.30267
Cl	1.88027	-1.68525	1.74949
O	-0.66822	-1.02546	0.06680
Au	-2.27872	-2.34899	-0.37539
Cl	-3.97989	-3.71780	-0.82591
C	-0.84076	3.51240	-1.30073
H	-0.46707	3.49781	-0.27384
H	-0.29826	2.77180	-1.88665
H	-0.57893	4.48631	-1.72139
C	-0.56512	0.78582	2.78602
H	0.10449	0.27385	2.09819
H	-0.51638	1.86204	2.59988
H	-0.15858	0.60101	3.78325
C	-7.08510	1.56661	-0.77426
H	-7.01881	2.65712	-0.73034
H	-6.66288	1.23018	-1.72432
H	-8.14563	1.30538	-0.77301
Si	1.30948	-1.33955	-0.27353
Cl	-1.04904	0.35312	-2.36750

Coordination through P (sp^2) atom

R = H

P	0.50562	0.13284	1.11549
C	0.30871	-0.25356	-0.46491
P	-1.09919	-0.29801	-1.46341
Cl	1.29170	-2.62781	-2.18597
Si	1.36906	-0.49748	-2.16982
O	-0.40343	-0.18808	-2.82264
H	1.88508	-0.03306	1.36561
H	-1.81760	-1.49504	-1.32603
Cl	-2.51617	1.07677	-1.21270
Au	-0.90029	0.77800	2.67214
Cl	-2.35271	1.43037	4.26430

R = Me

P	0.54920	0.11872	1.06651
C	0.33194	-0.24855	-0.51360
P	-1.09593	-0.33666	-1.48565
Cl	1.45597	-2.62511	-2.15980
Si	1.37370	-0.48052	-2.21334
O	-0.39480	-0.30957	-2.85565
Cl	-2.38232	1.19163	-1.32325
Au	-0.96062	0.65608	2.57678
Cl	-2.52622	1.17073	4.12013
C	2.29767	-0.03737	1.49236
H	2.44342	-0.97657	2.03315
H	2.91326	-0.03850	0.59222
H	2.58254	0.78172	2.15446
C	-2.07892	-1.80154	-1.24358
H	-2.84969	-1.84470	-2.01510
H	-1.40739	-2.65822	-1.33281
H	-2.53991	-1.77564	-0.25491

R = *t*-Bu

P	0.80873	0.10291	0.86099
C	0.50413	-0.41203	-0.67193
P	-1.02254	-0.60216	-1.48595
Cl	1.93274	-2.65788	-2.41991
Si	1.36609	-0.59616	-2.47454
O	-0.44872	-0.79014	-2.90736
Au	-0.67063	0.79048	2.35410
Cl	-2.20687	1.47791	3.86775
C	2.61823	0.05224	1.24977
C	2.81121	0.61142	2.65685
C	3.08321	-1.40429	1.18748
C	3.36994	0.90828	0.23190
H	2.47490	1.64771	2.73526
H	2.27490	0.02516	3.40651
H	3.87586	0.58256	2.90501
H	2.95043	-1.83435	0.19370
H	4.14782	-1.44300	1.43886
H	2.54944	-2.02942	1.90788
H	4.43942	0.86820	0.46004
H	3.23682	0.55334	-0.79309
H	3.05725	1.95421	0.27311
C	-2.13248	-1.97826	-1.01521
C	-1.29265	-3.25707	-1.00435
C	-2.74058	-1.72514	0.36061
C	-3.21896	-2.06975	-2.08968
H	-0.84181	-3.46130	-1.97569
H	-0.49454	-3.21715	-0.26136
H	-1.95300	-4.09012	-0.74894
H	-3.35015	-0.82117	0.38733
H	-3.38397	-2.57292	0.61088
H	-1.97918	-1.64981	1.13991
H	-3.84361	-2.93863	-1.86595
H	-3.86109	-1.18784	-2.09886
H	-2.79288	-2.20655	-3.08521
Cl	-2.16281	1.05753	-1.43919

R= Ph

P	0.81466	-0.13222	1.00890
C	0.42910	-0.20230	-0.58750
P	-1.14443	-0.25436	-1.31192
Cl	1.64467	-1.88714	-2.96444
Si	1.15416	0.12923	-2.41377
O	-0.69823	0.04669	-2.75464
Au	-0.58989	0.01867	2.70897
Cl	-2.08320	0.11480	4.40598
C	-2.03668	-1.78921	-1.18994
C	-2.77942	-2.09811	-0.05049
C	-1.92638	-2.70004	-2.23871
C	-3.41335	-3.32455	0.03376
H	-2.86750	-1.38761	0.76401
C	-2.57276	-3.92214	-2.14402
H	-1.33928	-2.45157	-3.11402
C	-3.31128	-4.23326	-1.01218
H	-3.99120	-3.56937	0.91678
H	-2.49596	-4.63280	-2.95820
H	-3.81346	-5.19143	-0.94276
C	2.57107	-0.20116	1.26780
C	3.46115	-0.60349	0.26713
C	3.05631	0.17424	2.52414
C	4.82028	-0.60712	0.52216
H	3.09037	-0.94394	-0.69243
C	4.41782	0.16934	2.76844
H	2.36114	0.47396	3.30171
C	5.29848	-0.21761	1.76728
H	5.50966	-0.92299	-0.25173
H	4.79262	0.46557	3.74085
H	6.36507	-0.22484	1.96083
Cl	-2.40972	1.15699	-0.65158

R = Mes

P	0.79940	-0.30541	1.63752
C	0.27375	-0.40067	0.08643
P	-1.24804	-0.32366	-0.75250
Cl	1.44551	-1.66115	-2.54563
Si	1.13880	0.25001	-1.59870
O	-0.69603	0.43729	-1.97561
Au	-0.32417	-0.23514	3.53876
Cl	-1.45433	-0.16398	5.49812
C	-2.06760	-1.85648	-1.17698
C	-2.34889	-2.78531	-0.15535
C	-2.44506	-2.12257	-2.51245
C	-2.96403	-3.98155	-0.49135
C	-3.05807	-3.33984	-2.77938
C	-3.31544	-4.28657	-1.79800
H	-3.18196	-4.69137	0.30029
H	-3.35486	-3.54771	-3.80236
C	2.59472	-0.21280	1.62795
C	3.35474	-1.37097	1.40490
C	3.20911	1.02207	1.88764
C	4.73796	-1.26248	1.44496
C	4.59492	1.07536	1.91056
C	5.37635	-0.05378	1.69573
H	5.33513	-2.15315	1.27522
H	5.07763	2.02947	2.09861
C	-2.01145	-2.55232	1.28433
H	-0.95391	-2.75051	1.47727
H	-2.21922	-1.53256	1.61359
H	-2.59073	-3.22024	1.92194
C	-2.25093	-1.18683	-3.66932
H	-2.62371	-0.18326	-3.46385
H	-1.19485	-1.08766	-3.92490
H	-2.77972	-1.57614	-4.53941
C	-3.94622	-5.59860	-2.14135
H	-4.61261	-5.50806	-3.00060
H	-3.17767	-6.33394	-2.39948
H	-4.51452	-6.00039	-1.30110
C	6.86998	0.02449	1.76118
H	7.21947	-0.14598	2.78434
H	7.33644	-0.73016	1.12594
H	7.23092	1.00683	1.45216
C	2.71749	-2.69925	1.12845
H	1.93150	-2.92864	1.85505
H	2.26315	-2.72105	0.13392
H	3.45760	-3.49830	1.17881
C	2.40240	2.26656	2.09919
H	1.85823	2.54126	1.19021
H	1.66432	2.13993	2.89796
H	3.04403	3.10547	2.36927
Cl	-2.64437	0.83837	0.13426

R = Mes*

P	-1.22485	0.73325	0.01613
C	-0.26043	-0.53321	0.42510
P	1.42508	-0.92817	0.70319
Cl	-1.24208	-3.26331	-0.99783
O	1.21304	-2.45381	0.60882
C	3.05570	-0.52420	0.02176
C	3.60028	-1.48158	-0.88375
C	3.93524	0.35768	0.70237
C	4.94939	-1.77292	-0.75839
C	5.27893	0.00566	0.75870
C	5.79606	-1.11171	0.12285

H	5.36037	-2.54615	-1.39437
H	5.94549	0.64799	1.31682
C	-2.92430	0.09930	0.11216
C	-3.59323	-0.33082	-1.06593
C	-3.47345	-0.17927	1.38784
C	-4.66563	-1.20044	-0.91056
C	-4.53952	-1.07089	1.44513
C	-5.11861	-1.63907	0.32508
H	-5.15786	-1.57018	-1.79433
H	-4.93319	-1.32945	2.41954
C	-3.27500	0.17546	-2.48719
C	-3.10698	0.47461	2.73840
C	-6.22669	-2.67528	0.47873
C	3.59155	1.76879	1.21204
C	2.86963	-2.10252	-2.08805
C	7.24203	-1.56304	0.28208
C	-4.38371	1.14834	3.27603
H	-4.16595	1.62732	4.23427
H	-4.73936	1.91681	2.58515
H	-5.19675	0.44059	3.43717
C	-2.05783	1.58140	2.68470
H	-1.04874	1.20679	2.50649
H	-2.29805	2.34853	1.94331
H	-2.02865	2.08276	3.65617
C	-2.63410	-0.58196	3.74226
H	-3.39312	-1.34582	3.91970
H	-1.73463	-1.09018	3.38854
H	-2.40991	-0.10604	4.70105
C	-7.40584	-2.06290	1.24336
H	-8.20124	-2.80388	1.36137
H	-7.11464	-1.72781	2.24086
H	-7.81758	-1.20431	0.70708
C	-6.73921	-3.18166	-0.86696
H	-7.51289	-3.93482	-0.70009
H	-7.18454	-2.38070	-1.46282
H	-5.94455	-3.64876	-1.45375
C	-5.67432	-3.87135	1.26406
H	-4.82743	-4.32337	0.74268
H	-5.33436	-3.57938	2.25973
H	-6.45057	-4.63218	1.38436
C	-4.15748	-0.49205	-3.54713
H	-5.22015	-0.29884	-3.38627
H	-3.89841	-0.07627	-4.52311
H	-3.99815	-1.57204	-3.59510
C	-3.58286	1.67808	-2.53888
H	-4.63276	1.86482	-2.30041
H	-2.97414	2.25216	-1.83674
H	-3.38424	2.06832	-3.54069
C	-1.83139	-0.08578	-2.92459
H	-1.09802	0.51387	-2.38023
H	-1.56472	-1.13684	-2.80837
H	-1.71852	0.19223	-3.97622
C	2.14346	2.17816	0.98428
H	1.84182	1.98443	-0.04832
H	1.44094	1.69372	1.66039
H	2.03860	3.25341	1.14284
C	4.43122	2.72934	0.34257
H	4.19816	3.76021	0.62044
H	5.50301	2.57710	0.47368
H	4.19643	2.60525	-0.71704
C	3.97227	1.99090	2.67769
H	5.03142	1.79726	2.85796
H	3.77894	3.03236	2.94716
H	3.39360	1.35555	3.34743
C	1.54421	-1.41233	-2.39537
H	1.16324	-1.78004	-3.35118
H	0.76229	-1.62507	-1.67059
H	1.67213	-0.32998	-2.48360
C	2.67189	-3.61277	-1.93435
H	2.01585	-3.84829	-1.09925
H	2.22647	-4.01867	-2.84644
H	3.62842	-4.11754	-1.77684
C	3.75289	-1.85600	-3.32796
H	3.97390	-0.79341	-3.45419
H	4.69773	-2.39887	-3.29313
H	3.21419	-2.19819	-4.21502
C	8.00195	-0.72137	1.30368
H	8.08994	0.32203	0.99043
H	7.52486	-0.74836	2.28652
H	9.01513	-1.11391	1.41633
C	7.96671	-1.45988	-1.06543
H	7.50254	-2.08922	-1.82715
H	7.96690	-0.43092	-1.43312
H	9.00566	-1.78215	-0.95569
C	7.25166	-3.02206	0.75465
H	6.75054	-3.12462	1.72008
H	6.74816	-3.68120	0.04478
H	8.28127	-3.37248	0.86575
Au	-0.71677	2.81365	-0.57849
Cl	-0.21178	4.95191	-1.15149
Si	-0.65744	-2.46756	0.90978
Cl	1.67207	-0.61904	2.70834

Coordination through π(C=P) bond

R = H

P	0.69472	0.17989	1.10190
C	0.33114	0.00754	-0.57998
P	-1.16640	-0.34682	-1.38664
Cl	1.24536	-2.59254	-2.00106
Si	1.19657	-0.48193	-2.35779
O	-0.64833	-0.35774	-2.82862
H	2.09460	-0.08498	1.00090
H	-1.67450	-1.59472	-0.99565
Cl	-2.75954	0.83730	-1.17799
Au	0.71809	2.10029	-0.29106
Cl	1.06215	4.30588	-0.51413

R = Me

P	0.71426	0.20744	0.99808
C	0.32776	0.02436	-0.67444
P	-1.17263	-0.42007	-1.44272
Cl	1.50355	-2.55792	-1.97888
Si	1.19939	-0.47209	-2.43381
O	-0.63116	-0.55768	-2.87746
Cl	-2.68862	0.89829	-1.40704
Au	0.61786	2.13712	-0.39252
Cl	0.88043	4.36420	-0.55661
C	2.50831	-0.16477	1.05017
H	2.96691	0.40145	1.86285
H	2.60661	-1.23074	1.28108
H	3.02399	0.03857	0.11190
C	-1.87753	-1.93861	-0.83012
H	-2.24642	-1.79010	0.18583
H	-2.69723	-2.23736	-1.48564
H	-1.08865	-2.69353	-0.84841

R = *t*-Bu

P	0.64331	0.17443	0.96019
C	0.35142	0.11982	-0.74698
P	-1.15511	-0.34988	-1.52999
Cl	2.02724	-1.93812	-2.56224

Si	1.18140	0.03639	-2.59356
O	-0.60172	-0.44604	-2.96499
Au	0.41969	2.21959	-0.22544
Cl	0.51006	4.46610	-0.08256
C	2.47825	-0.02039	1.24597
C	2.67352	-1.53350	1.40299
C	3.39248	0.52626	0.16312
C	2.75273	0.68162	2.57767
H	2.00073	-1.96003	2.15371
H	2.52162	-2.05597	0.45604
H	3.69797	-1.72759	1.73670
H	3.26258	1.60186	0.02549
H	4.43494	0.35096	0.45008
H	3.23867	0.02922	-0.79644
H	3.78809	0.49638	2.87865
H	2.61420	1.76267	2.49817
H	2.10172	0.31305	3.37529
C	-1.98747	-1.89021	-0.99219
C	-3.03809	-2.23579	-2.04991
C	-0.93347	-2.99626	-0.92193
C	-2.63114	-1.67871	0.37641
H	-3.82274	-1.48020	-2.11093
H	-2.58993	-2.35717	-3.03760
H	-3.50446	-3.18411	-1.76954
H	-0.17210	-2.79155	-0.16756
H	-1.43943	-3.92538	-0.64587
H	-0.43155	-3.14907	-1.87739
H	-3.06409	-2.62858	0.70150
H	-1.90192	-1.37156	1.13020
H	-3.43009	-0.93721	0.34386
Cl	-2.60452	1.05661	-1.49595

R = Ph

P	0.70624	0.17366	1.09576
C	0.22841	0.18574	-0.56754
P	-1.37738	-0.08097	-1.19255
Cl	1.23581	-1.68238	-3.02672
Si	0.78478	0.35331	-2.50956
O	-1.08439	0.23993	-2.67051
Au	0.57242	2.26222	-0.06606
Cl	0.89611	4.48694	0.08177
C	2.48346	-0.14403	1.11432
C	3.13237	0.15556	2.31650
C	3.21352	-0.69432	0.05919
C	4.49310	-0.05956	2.44928
H	2.56634	0.57107	3.14461
C	4.57195	-0.91839	0.20157
H	2.71685	-0.97188	-0.86255
C	5.21324	-0.59556	1.39064
H	4.99203	0.18770	3.37896
H	5.13311	-1.34828	-0.61996
H	6.27817	-0.76967	1.49460
C	-1.99556	-1.73854	-0.97898
C	-2.10217	-2.56964	-2.09000
C	-2.35225	-2.19449	0.28944
C	-2.57366	-3.86309	-1.92589
H	-1.81096	-2.20629	-3.06724
C	-2.81521	-3.48852	0.44180
H	-2.27092	-1.54237	1.15259
C	-2.92699	-4.32040	-0.66590
H	-2.65964	-4.51505	-2.78689
H	-3.09211	-3.84966	1.42502
H	-3.29234	-5.33350	-0.54270
Cl	-2.84047	1.11256	-0.49672

R = Mes

P	0.70795	0.14859	1.51674
C	0.10204	0.17174	-0.11185
P	-1.48872	-0.08167	-0.80565
Cl	1.27374	-0.92560	-3.01509
Si	0.63613	0.82495	-1.95385
O	-1.19236	0.60767	-2.15715
Au	0.28214	2.26425	0.45187
Cl	0.43409	4.49138	0.82501
C	2.50847	-0.07254	1.35050
C	3.37540	0.83875	1.97893
C	3.02402	-1.24410	0.76091
C	4.74560	0.61687	1.90865
C	4.39919	-1.42022	0.72617
C	5.28026	-0.49079	1.26797
H	5.41241	1.33178	2.38148
H	4.79507	-2.32522	0.27485
C	-2.02474	-1.78324	-0.97683
C	-2.35525	-2.31650	-2.24490
C	-2.13051	-2.58018	0.18052
C	-2.70870	-3.65755	-2.30897
C	-2.49304	-3.91272	0.04410
C	-2.76553	-4.47886	-1.19115
H	-2.96503	-4.07083	-3.27918
H	-2.57638	-4.52020	0.93997
C	2.87902	2.04205	2.72126
H	2.67217	2.88113	2.05028
H	1.95090	1.83850	3.26320
H	3.62389	2.37407	3.44605
C	2.14458	-2.34135	0.23760
H	1.36392	-2.60654	0.95720
H	1.65144	-2.06646	-0.69557
H	2.73541	-3.23874	0.05012
C	6.76076	-0.68782	1.16870
H	7.28858	-0.15522	1.96140
H	7.02753	-1.74498	1.22537
H	7.13493	-0.30888	0.21239
C	-1.88590	-2.07729	1.57155
H	-0.84555	-2.22878	1.87162
H	-2.10863	-1.01881	1.70152
H	-2.50850	-2.62950	2.27718
C	-2.38890	-1.54121	-3.52958
H	-2.96195	-0.61810	-3.44238
H	-1.38638	-1.26426	-3.85766
H	-2.84616	-2.15480	-4.30590
C	-3.11568	-5.92731	-1.31973
H	-3.84840	-6.09091	-2.11190
H	-2.22630	-6.51253	-1.57347
H	-3.51717	-6.32626	-0.38719
Cl	-3.04979	0.90887	0.02587

R = Mes*

P	0.68548	0.31836	1.04893
C	0.40885	0.05722	-0.66620
P	-1.11961	-0.16336	-1.59247
O	-0.42902	-0.00202	-2.97679
Cl	2.51777	-0.83105	-3.21163
Au	0.65550	2.23171	-0.51523
Cl	0.73431	4.50259	-0.26653
C	2.51059	0.09287	1.21706
C	3.16106	-1.08485	0.75290
C	3.31518	1.17272	1.66464
C	4.49825	-0.99465	0.39932
C	4.65306	1.19017	1.26659
C	5.24810	0.16509	0.55477
H	4.98601	-1.87733	0.00605
H	5.24989	2.04719	1.54327

C	2.94124	2.25286	2.70395
C	2.60539	-2.52048	0.86097
C	6.67635	0.23418	0.02761
C	3.78157	1.91431	3.95227
H	3.53382	0.92031	4.33425
H	4.85197	1.93633	3.74305
H	3.57448	2.64448	4.73962
C	3.30750	3.66468	2.23580
H	2.75247	3.94869	1.34156
H	3.05674	4.38086	3.02328
H	4.37382	3.77187	2.03078
C	1.48908	2.27402	3.17222
H	1.41212	2.95914	4.02121
H	0.80260	2.63889	2.40827
H	1.15208	1.29365	3.51881
C	2.90172	-3.35583	-0.38806
H	2.50663	-2.88171	-1.28544
H	3.97110	-3.51354	-0.53583
H	2.44418	-4.34390	-0.28574
C	3.34654	-3.15116	2.05669
H	4.42707	-3.14661	1.90621
H	3.13438	-2.60572	2.97979
H	3.02302	-4.18798	2.18905
C	1.11848	-2.64928	1.17336
H	0.82419	-2.05275	2.04300
H	0.48850	-2.37522	0.32962
H	0.90197	-3.69309	1.41870
C	7.32847	1.58683	0.30054
H	6.76135	2.40673	-0.14674
H	7.43130	1.78249	1.37109
H	8.33152	1.60180	-0.13264
C	6.64976	0.00840	-1.48898
H	7.66564	0.04242	-1.89284
H	6.21689	-0.96070	-1.74517
H	6.05678	0.77791	-1.98858
C	7.53064	-0.85422	0.68804
H	7.55989	-0.72472	1.77286
H	7.14824	-1.85528	0.47852
H	8.55636	-0.80602	0.31171
C	-2.36730	-1.49681	-1.68738
C	-2.35683	-2.33340	-2.84649
C	-3.57812	-1.36894	-0.95377
C	-3.58403	-2.65362	-3.40646
C	-1.13646	-3.05294	-3.45154
C	-4.75932	-1.76671	-1.56912
C	-3.71976	-0.98136	0.52843
C	-4.80450	-2.31329	-2.83979
H	-3.58132	-3.22524	-4.32471
C	-0.69866	-2.45906	-4.79395
C	-1.52579	-4.52316	-3.71218
C	0.01382	-3.12991	-2.45598
H	-5.68168	-1.63401	-1.02214
C	-2.41473	-0.59617	1.20092
C	-4.21266	-2.25497	1.24415
C	-4.74205	0.13624	0.75621
C	-6.10534	-2.62459	-3.56746
H	0.10268	-3.06985	-5.21804
H	-1.53176	-2.46342	-5.50182
H	-0.33757	-1.43959	-4.69802
H	-2.21494	-4.64238	-4.54890
H	-0.62000	-5.08000	-3.96458
H	-1.97239	-4.98455	-2.82840
H	0.40819	-2.16366	-2.15391
H	-0.29392	-3.67453	-1.55979
H	0.85036	-3.66832	-2.90585
H	-1.64028	-1.34929	1.04165
H	-2.04370	0.37856	0.88506
H	-2.56695	-0.52009	2.28007
H	-4.30880	-2.05251	2.31419
H	-5.18353	-2.58761	0.87510
H	-3.50263	-3.07555	1.11543
H	-5.73426	-0.12608	0.38579
H	-4.83382	0.32770	1.82841
H	-4.43389	1.06418	0.27431
C	-7.33071	-2.15779	-2.78633
C	-6.22121	-4.13715	-3.79236
C	-6.09366	-1.90777	-4.92314
H	-7.43276	-2.68214	-1.83274
H	-7.30067	-1.08336	-2.58893
H	-8.23226	-2.36235	-3.36820
H	-5.39835	-4.52369	-4.39671
H	-6.22315	-4.67626	-2.84184
H	-7.15391	-4.36605	-4.31475
H	-6.02166	-0.82535	-4.79365
H	-5.25399	-2.22730	-5.54342
H	-7.01545	-2.12542	-5.46908
Cl	-2.35696	1.45806	-1.50700
Si	1.19989	0.61508	-2.42064

[R-P=C(Si(II)Cl)-P(O)MeR](AuCl)
Coordination through Si(II) atom
R= H

P	0.77632	-0.24722	1.12673
C	0.47308	-0.35419	-0.49894
P	-1.03242	-0.30820	-1.39698
Cl	1.73654	-2.36604	-2.54415
Si	1.36436	-0.35031	-2.23004
O	-0.35426	-0.22164	-2.79512
H	2.20179	-0.26832	1.04949
H	-1.79886	-1.48584	-1.29275
Au	2.80107	1.26105	-2.81441
Cl	4.23783	2.96510	-3.35313
C	-2.16382	1.03726	-1.10499
H	-2.95615	1.01586	-1.85628
H	-2.60140	0.93143	-0.10937
H	-1.61807	1.97964	-1.17083

R = Me

P	0.80059	-0.03016	1.03831
C	0.41483	-0.23519	-0.55892
P	-1.13949	-0.29909	-1.36303
Cl	1.67991	-2.23722	-2.63814
Si	1.19721	-0.23265	-2.32588
O	-0.53244	-0.20615	-2.80486
C	-2.25849	1.06238	-1.06428
H	-3.10364	1.00416	-1.75349
H	-2.62247	1.02209	-0.03543
H	-1.72119	1.99790	-1.22400
C	2.62884	0.00658	1.03178
H	3.02048	0.49355	0.13572
H	2.98778	0.51863	1.92564
H	2.99840	-1.02343	1.05977
C	-2.04163	-1.82943	-1.15786
H	-2.41378	-1.90705	-0.13420
H	-2.87982	-1.86084	-1.85727
H	-1.36153	-2.65695	-1.36451
Au	2.56413	1.40921	-3.00621
Cl	3.95121	3.13850	-3.60955

R = *t*-Bu

P	0.76300	-0.03880	0.82836

C	0.55116	-0.43035	-0.76897
P	-1.00035	-0.53460	-1.60277
Cl	2.02813	-2.57538	-2.66434
Si	1.35726	-0.61159	-2.52340
O	-0.35372	-0.76831	-3.01632
C	-1.90681	1.01209	-1.61024
H	-2.76036	0.95704	-2.28739
H	-2.24525	1.25747	-0.60253
H	-1.22150	1.78749	-1.95699
Au	2.53894	1.09120	-3.39371
Cl	3.69751	2.91044	-4.19545
C	2.58597	0.12180	1.17053
C	3.49208	-0.63428	0.21441
C	2.86485	1.62901	1.10090
C	2.78307	-0.37446	2.60513
H	3.25175	-1.69839	0.17287
H	3.44313	-0.22716	-0.79840
H	4.53116	-0.53597	0.54581
H	2.20152	2.20231	1.75579
H	3.89218	1.81678	1.42971
H	2.76342	2.01002	0.08229
H	3.81490	-0.18629	2.91651
H	2.12236	0.13901	3.30921
H	2.60014	-1.44895	2.68992
C	-2.12178	-1.91021	-1.16772
C	-3.20215	-2.00353	-2.24817
C	-1.30781	-3.20327	-1.13074
C	-2.73843	-1.62831	0.20252
H	-3.84847	-1.12356	-2.26743
H	-2.76839	-2.14170	-3.24058
H	-3.83489	-2.86940	-2.03480
H	-0.52868	-3.17110	-0.36750
H	-1.98182	-4.03124	-0.89495
H	-0.83320	-3.41201	-2.09044
H	-3.34972	-2.48615	0.49526
H	-1.97614	-1.48772	0.97361
H	-3.38829	-0.75068	0.19215

R = Ph

P	0.93615	-0.19721	0.69149
C	0.53096	-0.30173	-0.91648
P	-1.11197	-0.39119	-1.53769
Cl	1.72020	-2.40185	-2.96468
Si	1.10279	-0.42302	-2.75750
O	-0.65893	-0.55351	-3.03058
C	-2.06695	1.10831	-1.33495
H	-3.00435	1.03260	-1.88886
H	-2.27286	1.28940	-0.27862
H	-1.47048	1.93444	-1.72560
Au	2.23100	1.21772	-3.79805
Cl	3.38096	2.94068	-4.79828
C	-2.11350	-1.78387	-1.04101
C	-1.91486	-3.00802	-1.67897
C	-3.04191	-1.67597	-0.00611
C	-2.65067	-4.11280	-1.28456
H	-1.19020	-3.08871	-2.48081
C	-3.76868	-2.78793	0.38541
H	-3.20343	-0.73043	0.49851
C	-3.57412	-4.00390	-0.25434
H	-2.50160	-5.06243	-1.78448
H	-4.49087	-2.70382	1.18877
H	-4.14714	-4.87165	0.05151
C	2.72431	-0.10960	0.81488
C	3.22800	-0.26791	2.11157
C	3.61305	0.12977	-0.23769
C	4.59147	-0.21598	2.34766
H	2.54075	-0.43621	2.93521
C	4.97195	0.19513	0.00379
H	3.24542	0.29334	-1.24528
C	5.46224	0.01553	1.29305
H	4.97396	-0.34868	3.35288
H	5.65232	0.39170	-0.81641
H	6.52993	0.06487	1.47468

R = Mes

P	0.88169	0.37527	0.64358
C	0.57907	-0.48163	-0.74454
P	-0.87631	-0.80595	-1.70176
Cl	2.01843	-3.17841	-1.63890
Si	1.58579	-1.21667	-2.21148
O	-0.05187	-1.39207	-2.90285
C	-1.71930	0.65469	-2.31426
H	-2.42267	0.34781	-3.09149
H	-2.25622	1.17219	-1.52115
H	-0.96672	1.31794	-2.74411
Au	3.09181	-0.00599	-3.35752
Cl	4.59086	1.34263	-4.47358
C	-2.08405	-1.97595	-1.05920
C	-2.31686	-3.23045	-1.66684
C	-2.80605	-1.60333	0.09109
C	-3.25376	-4.07220	-1.07979
C	-3.72111	-2.49386	0.63378
C	-3.95713	-3.73761	0.06895
H	-3.43611	-5.03532	-1.54577
H	-4.26574	-2.19991	1.52580
C	2.69195	0.31237	0.80398
C	3.29460	-0.75283	1.48590
C	3.45719	1.38585	0.32728
C	4.67390	-0.73953	1.64574
C	4.83263	1.35160	0.50659
C	5.46113	0.29431	1.15323
H	5.14686	-1.56317	2.17253
H	5.42973	2.17246	0.12118
C	2.48531	-1.89209	2.03000
H	1.60485	-1.53870	2.57784
H	2.12775	-2.54232	1.22668
H	3.07966	-2.49911	2.71413
C	2.82200	2.53409	-0.39539
H	2.59587	2.26106	-1.43138
H	1.88468	2.84637	0.07943
H	3.48763	3.39730	-0.42318
C	6.95146	0.26378	1.29549
H	7.41646	-0.09122	0.37073
H	7.35270	1.25886	1.49772
H	7.26269	-0.40399	2.10036
C	-2.67495	-0.26351	0.75559
H	-1.67841	0.17490	0.67929
H	-3.38953	0.45030	0.33372
H	-2.89820	-0.34712	1.81984
C	-1.63596	-3.73031	-2.90841
H	-1.71733	-3.03104	-3.74039
H	-0.57076	-3.89907	-2.74391
H	-2.08768	-4.67498	-3.21104
C	-4.92192	-4.69935	0.68743
H	-5.43217	-5.29435	-0.07195
H	-4.39625	-5.39599	1.34784
H	-5.67346	-4.18264	1.28603

R = Mes*

P	-0.54787	1.62204	1.53692
C	0.11914	0.55453	0.44723
P	1.71358	-0.21546	0.25686

Cl	-0.93069	0.50772	-2.66550
O	1.28793	-0.87375	-1.11675
C	1.83872	-1.64882	1.34508
H	2.64556	-2.27896	0.96586
H	2.01972	-1.40972	2.38746
H	0.89054	-2.18399	1.25006
C	3.41877	0.41507	0.12335
C	4.01638	0.51114	-1.16675
C	4.26821	0.30766	1.25608
C	5.33702	0.10225	-1.28770
C	5.59078	-0.06931	1.04986
C	6.12512	-0.28233	-0.21074
H	5.77693	0.10499	-2.27617
H	6.22693	-0.18768	1.91607
C	-2.30917	1.59170	1.03372
C	-2.79241	2.53375	0.10004
C	-3.15065	0.54678	1.49515
C	-3.98812	2.23986	-0.55286
C	-4.31879	0.31166	0.78357
C	-4.72489	1.09684	-0.28881
H	-4.34749	2.92302	-1.30586
H	-4.93692	-0.52662	1.06787
C	-2.12906	3.90618	-0.14088
C	-2.94578	-0.18565	2.83952
C	-5.96124	0.70338	-1.08985
C	3.92365	0.76657	2.68654
C	3.38430	1.17352	-2.40552
C	7.53542	-0.81123	-0.43864
C	-4.02943	-1.23957	3.08396
H	-3.86020	-1.69424	4.06277
H	-5.03053	-0.80372	3.10036
H	-3.99992	-2.03835	2.33818
C	-3.06835	0.87606	3.94400
H	-2.28579	1.63583	3.86200
H	-4.03455	1.38342	3.89315
H	-2.97909	0.40582	4.92772
C	-1.60514	-0.90464	3.00934
H	-1.39642	-1.56711	2.16678
H	-0.76672	-0.21191	3.12786
H	-1.63974	-1.50734	3.92102
C	-7.17856	0.61090	-0.16238
H	-8.06547	0.33017	-0.73716
H	-7.04034	-0.14018	0.61742
H	-7.37673	1.57035	0.32272
C	-6.27913	1.70720	-2.19478
H	-7.15627	1.36838	-2.75126
H	-6.50637	2.69916	-1.79451
H	-5.45439	1.80146	-2.90559
C	-5.70857	-0.66473	-1.73639
H	-4.85785	-0.62125	-2.42059
H	-5.49314	-1.43668	-0.99514
H	-6.58871	-0.97855	-2.30499
C	-3.00294	4.80999	-1.01497
H	-4.00242	4.94565	-0.59616
H	-2.53390	5.79460	-1.07952
H	-3.10109	4.42846	-2.03390
C	-1.97080	4.61840	1.20962
H	-2.93683	4.72868	1.70771
H	-1.30404	4.07994	1.88825
H	-1.54601	5.61505	1.05898
C	-0.76531	3.82107	-0.83123
H	-0.00181	3.36216	-0.20015
H	-0.82124	3.24333	-1.75489
H	-0.41614	4.82978	-1.07117
C	2.50625	1.29790	2.85262
H	2.27014	2.05417	2.10076
H	1.73506	0.52865	2.82532
H	2.41139	1.77806	3.82922
C	4.84777	1.96978	2.96678
H	4.63015	2.37086	3.96035
H	5.90245	1.69497	2.93765
H	4.68503	2.76462	2.23525
C	4.20493	-0.30365	3.74546
H	5.24964	-0.61861	3.73789
H	3.99205	0.09935	4.73888
H	3.59357	-1.19715	3.61149
C	2.21351	2.07581	-2.02557
H	1.84199	2.58437	-2.91805
H	1.36210	1.54725	-1.60770
H	2.53207	2.83974	-1.31115
C	2.99298	0.15987	-3.48488
H	2.21084	-0.51552	-3.14967
H	2.63458	0.69002	-4.37122
H	3.85979	-0.43683	-3.78125
C	4.43284	2.12150	-3.02231
H	4.84200	2.80821	-2.27750
H	5.26153	1.59321	-3.49508
H	3.94757	2.71533	-3.80067
C	8.22558	-1.19781	0.86670
H	8.37635	-0.33565	1.52144
H	7.65997	-1.95472	1.41597
H	9.21079	-1.61548	0.64748
C	8.38216	0.26104	-1.13500
H	7.96753	0.54058	-2.10536
H	8.44967	1.16506	-0.52485
H	9.39579	-0.11323	-1.30147
C	7.45739	-2.05597	-1.33094
H	6.87054	-2.84467	-0.85402
H	6.99842	-1.83468	-2.29644
H	8.46138	-2.44560	-1.51878
Au	-1.80941	-2.36449	-0.51742
Cl	-3.25039	-4.07732	0.07719
Si	-0.46820	-0.60837	-0.97211

Coordination through O atom

R = H

P	0.66476	-0.20823	1.17026
C	0.42206	-0.32631	-0.46596
P	-1.04642	-0.14557	-1.38115
Cl	1.23265	-2.50103	-2.44499
Si	1.51237	-0.41935	-2.12395
O	-0.34044	0.11039	-2.75288
H	2.08225	-0.40387	1.16541
H	-1.78134	-1.34331	-1.43844
Au	-1.45558	0.31553	-4.53488
Cl	-2.69771	0.55381	-6.35902
C	-2.21451	1.13390	-0.96080
H	-3.01271	1.15892	-1.70602
H	-2.63661	0.91374	0.02341
H	-1.70167	2.09616	-0.93469

R = Me

P	0.73750	0.04541	1.01710
C	0.42074	-0.22652	-0.58646
P	-1.11673	-0.23587	-1.39784
Cl	1.45825	-2.47678	-2.43161
Si	1.39273	-0.34591	-2.30303
O	-0.49768	-0.14628	-2.84527
C	-2.22760	1.13556	-1.11144
H	-3.07756	1.07155	-1.79463
H	-2.58204	1.10237	-0.07849

H	-1.69094	2.06944	-1.28130
C	2.55995	-0.13128	1.12440
H	3.05770	0.20822	0.21334
H	2.93403	0.42603	1.98455
H	2.79963	-1.18817	1.27616
C	-2.03262	-1.75689	-1.20553
H	-2.40762	-1.82809	-0.18208
H	-2.86718	-1.77423	-1.91029
H	-1.35697	-2.58846	-1.41081
Au	-1.68137	-0.16862	-4.58965
Cl	-2.99755	-0.17541	-6.37895

R = *t*-Bu

P	0.80650	-0.34616	0.84989
C	0.42923	-0.23448	-0.76387
P	-1.20940	-0.30006	-1.38588
Cl	1.81631	-1.66666	-3.17556
Si	1.13785	0.25972	-2.55165
O	-0.78421	-0.03453	-2.88503
C	-2.25468	1.04504	-0.82437
H	-3.18714	1.07169	-1.39037
H	-2.46351	0.93403	0.24107
H	-1.71053	1.97658	-0.98681
Au	-2.03134	0.59854	-4.46316
Cl	-3.37892	1.26771	-6.09895
C	2.66249	-0.26451	1.03258
C	3.11251	-1.71265	1.25473
C	3.39687	0.34694	-0.14672
C	2.91572	0.55287	2.30207
H	2.57799	-2.18570	2.08386
H	2.96431	-2.31816	0.35781
H	4.17963	-1.72603	1.50109
H	3.04011	1.35333	-0.37659
H	4.46586	0.41826	0.08211
H	3.30614	-0.27004	-1.04423
H	3.98315	0.53569	2.54237
H	2.61845	1.59722	2.17586
H	2.37293	0.14869	3.16095
C	-2.10954	-1.88671	-1.21967
C	-3.41392	-1.81702	-2.01723
C	-1.22248	-3.00649	-1.76203
C	-2.40575	-2.11284	0.26415
H	-4.10344	-1.06700	-1.62509
H	-3.24216	-1.60806	-3.07551
H	-3.91095	-2.78850	-1.94559
H	-0.28092	-3.08347	-1.21640
H	-1.75933	-3.95300	-1.65550
H	-0.98852	-2.86354	-2.81756
H	-2.88177	-3.09085	0.37441
H	-1.49770	-2.11544	0.87209
H	-3.09337	-1.36682	0.66854

R = Ph

P	0.86202	-0.43387	1.07052
C	0.41336	-0.37308	-0.53141
P	-1.22288	-0.28649	-1.11301
Cl	1.38649	-1.70789	-3.19999
Si	1.12269	0.20497	-2.27999
O	-0.82224	0.30533	-2.52424
C	-2.33176	0.85155	-0.28983
H	-3.27406	0.92907	-0.83525
H	-2.52427	0.48982	0.72258
H	-1.85529	1.83178	-0.24868
Au	-2.22651	0.88335	-3.98196
Cl	-3.77311	1.49387	-5.45818
C	2.66220	-0.44483	1.06251
C	3.28163	-0.03689	2.24898
C	3.45628	-0.82952	-0.02248
C	4.66186	0.02415	2.33663
H	2.67132	0.24443	3.10184
C	4.83592	-0.77406	0.07131
H	2.99597	-1.21117	-0.92712
C	5.43869	-0.34133	1.24585
H	5.13308	0.35240	3.25575
H	5.44452	-1.07556	-0.77324
H	6.51984	-0.30029	1.31420
C	-2.05077	-1.86255	-1.26844
C	-3.35725	-1.91449	-1.75950
C	-1.38345	-3.03833	-0.93285
C	-3.98728	-3.13749	-1.90678
H	-3.87668	-1.00900	-2.05373
C	-2.02501	-4.25783	-1.07595
H	-0.36359	-2.99568	-0.57125
C	-3.32324	-4.30687	-1.56088
H	-4.99672	-3.17729	-2.29804
H	-1.50522	-5.17205	-0.81547
H	-3.82069	-5.26296	-1.67697

R = Mes

P	1.09250	-1.00675	1.31746
C	0.43175	-0.85324	-0.19863
P	-1.26966	-0.77431	-0.61907
Cl	1.08389	-1.46917	-3.16032
Si	0.96419	0.17787	-1.78574
O	-1.02565	0.29058	-1.77344
C	-2.27387	-0.09074	0.70097
H	-3.30483	0.06314	0.38719
H	-2.25008	-0.80423	1.52971
H	-1.84017	0.85641	1.02406
Au	-1.71464	2.30091	-1.79244
Cl	-2.45435	4.39807	-1.80076
C	2.83593	-0.53048	1.07370
C	3.25389	0.71284	1.57254
C	3.76322	-1.41241	0.50048
C	4.59237	1.06358	1.46577
C	5.09340	-1.02020	0.42087
C	5.52790	0.21372	0.88880
H	4.91255	2.02950	1.84542
H	5.81399	-1.70526	-0.01635
C	-2.13190	-2.21598	-1.27735
C	-3.37265	-2.00034	-1.91449
C	-1.62454	-3.52755	-1.15522
C	-4.06320	-3.09402	-2.42152
C	-2.36881	-4.57783	-1.67304
C	-3.58461	-4.38924	-2.31506
H	-5.01046	-2.91661	-2.92145
H	-1.97251	-5.58364	-1.57736
C	2.27440	1.67517	2.17455
H	1.65664	2.14179	1.40083
H	1.59456	1.17885	2.87588
H	2.78763	2.47185	2.71437
C	6.96175	0.62539	0.75687
H	7.12096	1.17131	-0.17833
H	7.26458	1.28325	1.57352
H	7.62623	-0.24027	0.74721
C	3.35674	-2.75768	-0.02318
H	2.67298	-3.27072	0.65989
H	2.84673	-2.66893	-0.98699
H	4.22921	-3.39667	-0.16572
C	-0.31775	-3.87144	-0.50779
H	0.51425	-3.37657	-1.01009
H	-0.28588	-3.58018	0.54402

H	-0.15492	-4.94810	-0.56011
C	-4.03797	-0.66234	-2.07621
H	-3.35154	0.18171	-2.11419
H	-4.61139	-0.64668	-3.00377
H	-4.74948	-0.48353	-1.26363
C	-4.33393	-5.54420	-2.90014
H	-3.94404	-5.78794	-3.89324
H	-4.23158	-6.43842	-2.28263
H	-5.39535	-5.31779	-3.00970

R = Mes*

P	2.05797	1.94983	-0.47768
C	1.05099	0.63354	-0.34499
P	-0.69688	0.47391	-0.50853
C	3.71769	1.16036	-0.34934
C	4.41265	1.23405	0.89063
C	4.24225	0.37337	-1.39406
C	5.44252	0.33460	1.10939
C	5.27613	-0.51627	-1.08799
C	5.85065	-0.60198	0.16314
H	5.95077	0.34817	2.06221
H	5.64237	-1.15929	-1.87594
C	3.90483	0.47630	-2.89685
C	4.12483	2.30215	1.96426
C	6.92105	-1.62754	0.51962
C	2.76220	2.11011	2.63504
H	1.92940	2.30150	1.95515
H	2.64752	1.09297	3.01419
H	2.66073	2.81033	3.46957
C	4.20379	3.69624	1.32691
H	5.18843	3.86385	0.88373
H	3.45376	3.84454	0.54716
H	4.03732	4.46304	2.08880
C	5.16740	2.27785	3.08568
H	5.12381	1.35643	3.67102
H	6.18403	2.40405	2.70628
H	4.96405	3.10566	3.76883
C	3.44612	-0.87325	-3.45876
H	2.54852	-1.23010	-2.94834
H	3.22576	-0.77591	-4.52573
H	4.21114	-1.64370	-3.35006
C	2.86075	1.52394	-3.27352
H	1.85028	1.22968	-2.99148
H	3.08084	2.50443	-2.83868
H	2.86513	1.65202	-4.35990
C	5.19920	0.90357	-3.61437
H	5.54012	1.87785	-3.25464
H	6.01002	0.19044	-3.46504
H	5.01559	0.98157	-4.68961
C	7.22429	-2.57416	-0.63852
H	7.62945	-2.04402	-1.50451
H	7.97188	-3.30635	-0.32415
H	6.33487	-3.12471	-0.95481
C	6.42852	-2.46398	1.70711
H	6.24000	-1.84488	2.58642
H	5.49923	-2.98304	1.46151
H	7.17945	-3.21155	1.97785
C	8.21853	-0.90867	0.90702
H	8.07656	-0.24833	1.76492
H	8.98785	-1.63894	1.17315
H	8.59465	-0.30529	0.07695
C	-2.13555	1.32394	0.20192
C	-2.73750	0.75489	1.36089
C	-2.93652	2.12579	-0.65260
C	-4.12092	0.65712	1.37389
C	-4.31659	1.99782	-0.55879
C	-4.93374	1.17544	0.37388
H	-4.58452	0.15416	2.21287
H	-4.93061	2.57196	-1.23935
C	-2.00359	0.40492	2.66810
C	-2.03581	-1.08745	3.00375
C	-2.73472	1.16207	3.79588
H	-1.41973	-1.67054	2.32145
H	-1.65036	-1.24073	4.01538
H	-3.05315	-1.48329	2.96823
H	-2.80055	2.23122	3.57983
H	-3.74335	0.78749	3.97254
H	-2.17186	1.03650	4.72425
C	-2.41279	3.27594	-1.53415
C	-3.03547	4.56025	-0.94793
C	-2.85479	3.16468	-2.99596
H	-2.76082	4.68280	0.10223
H	-2.66518	5.42766	-1.50118
H	-4.12361	4.55801	-1.01440
H	-2.44990	2.28166	-3.49075
H	-3.94169	3.11917	-3.08399
H	-2.51688	4.04402	-3.55057
C	-6.43256	0.91024	0.38802
C	-7.04820	1.49994	1.66276
C	-6.66113	-0.60641	0.36398
H	-6.89659	2.58139	1.71050
H	-8.12396	1.30586	1.68056
H	-6.61678	1.05871	2.56334
H	-6.22175	-1.06425	-0.52475
H	-6.22013	-1.09870	1.23248
H	-7.73340	-0.81985	0.36706
Cl	1.84433	-1.60238	1.77940
O	-0.68593	-1.01531	0.05972
Au	-2.26854	-2.36218	-0.35500
Cl	-3.95834	-3.74426	-0.80804
C	-0.90449	3.48864	-1.45070
H	-0.56895	3.57308	-0.41472
H	-0.30993	2.71575	-1.93595
H	-0.64460	4.42347	-1.95311
C	-0.57160	0.92336	2.68802
H	0.08944	0.40173	2.00039
H	-0.53828	1.99442	2.47130
H	-0.14732	0.77018	3.68318
C	-7.13762	1.52473	-0.81819
H	-7.07221	2.61619	-0.82184
H	-6.72905	1.14864	-1.75943
H	-8.19769	1.26300	-0.79050
C	-1.06784	0.22128	-2.26015
H	-2.10765	-0.09096	-2.36957
H	-0.86902	1.09098	-2.87881
H	-0.41524	-0.59731	-2.57585
Si	1.24843	-1.33114	-0.24689

Coordination through P(sp^2) atom

R = H

P	0.52080	0.09607	1.12056
C	0.31683	-0.25053	-0.46524
P	-1.12466	-0.26803	-1.44637
Cl	1.30083	-2.61348	-2.16580
Si	1.34907	-0.47598	-2.18798
O	-0.41220	-0.20463	-2.81415
H	1.90287	-0.03317	1.37824
H	-1.80225	-1.49011	-1.27632
Au	-0.93798	0.68391	2.66256
Cl	-2.49413	1.29589	4.17703
C	-2.35559	1.00400	-1.23253
H	-2.80502	0.90924	-0.24055

H	-1.88523	1.98312	-1.33055
H	-3.12828	0.88794	-1.99586

R = Me

P	0.82918	0.00873	0.94003
C	0.39670	-0.23576	-0.61622
P	-1.18321	-0.27650	-1.35940
Cl	1.34077	-2.46986	-2.53786
Si	1.15781	-0.32684	-2.47059
O	-0.67038	-0.20607	-2.82336
C	-2.28874	1.08064	-0.99246
H	-3.17497	1.00870	-1.62701
H	-2.58710	1.04300	0.05783
H	-1.77527	2.02179	-1.19127
C	2.62266	-0.10691	1.13138
H	3.11056	-0.07939	0.15677
H	2.97364	0.71209	1.76150
H	2.86322	-1.04747	1.63389
C	-2.04758	-1.80621	-1.03330
H	-2.34234	-1.84689	0.01764
H	-2.93444	-1.86574	-1.66798
H	-1.37311	-2.63178	-1.26460
Au	-0.51342	0.39698	2.65179
Cl	-1.96349	0.78708	4.34374

R = *t*-Bu

P	0.81316	0.08370	0.86464
C	0.47748	-0.38505	-0.67373
P	-1.08177	-0.52828	-1.46909
Cl	1.91785	-2.56672	-2.51193
Si	1.29247	-0.51764	-2.49714
O	-0.49917	-0.72602	-2.90273
C	-2.07039	0.96744	-1.40086
H	-2.97115	0.86162	-2.00749
H	-2.34288	1.19548	-0.36870
H	-1.46271	1.78143	-1.79875
Au	-0.66543	0.77376	2.36842
Cl	-2.24204	1.49165	3.83140
C	2.62677	0.01955	1.23731
C	2.83485	0.54899	2.65335
C	3.08453	-1.43716	1.14039
C	3.37323	0.89095	0.22915
H	2.50427	1.58517	2.75553
H	2.30136	-0.04971	3.39533
H	3.90123	0.50992	2.89291
H	2.93676	-1.84522	0.13939
H	4.15205	-1.48666	1.37764
H	2.55586	-2.07426	1.85408
H	4.44566	0.83628	0.44040
H	3.22082	0.56019	-0.80122
H	3.07062	1.93844	0.29855
C	-2.12266	-1.95989	-0.99524
C	-1.24212	-3.20242	-0.87865
C	-2.82349	-1.67829	0.33214
C	-3.14747	-2.15305	-2.11776
H	-0.70691	-3.41371	-1.80494
H	-0.50591	-3.10308	-0.07942
H	-1.88197	-4.05849	-0.64722
H	-3.51603	-0.83673	0.27128
H	-3.40313	-2.56175	0.61327
H	-2.11607	-1.48115	1.14136
H	-3.74757	-3.03749	-1.88709
H	-3.83359	-1.30776	-2.20579
H	-2.66310	-2.31359	-3.08236

R = Ph

P	0.81940	-0.13983	0.99360
C	0.42602	-0.20031	-0.59811
P	-1.17527	-0.21895	-1.30536
Cl	1.64605	-1.88500	-2.97264
Si	1.12525	0.12909	-2.43115
O	-0.70303	0.04074	-2.76307
C	-2.29129	1.06512	-0.75043
H	-3.19149	1.04847	-1.36807
H	-2.56286	0.91150	0.29599
H	-1.78767	2.02738	-0.85260
Au	-0.61829	0.03786	2.67204
Cl	-2.20266	0.19504	4.28472
C	-2.02087	-1.78819	-1.17697
C	-2.72546	-2.12232	-0.01990
C	-1.94813	-2.68440	-2.24067
C	-3.35848	-3.35019	0.06594
H	-2.77646	-1.43864	0.82119
C	-2.58998	-3.90920	-2.14640
H	-1.38841	-2.42046	-3.12953
C	-3.29239	-4.24097	-0.99743
H	-3.90328	-3.61097	0.96542
H	-2.53657	-4.60733	-2.97336
H	-3.79058	-5.20117	-0.92714
C	2.57554	-0.20944	1.26677
C	3.46890	-0.59846	0.26451
C	3.05532	0.14947	2.52919
C	4.82722	-0.60636	0.52534
H	3.10075	-0.92246	-0.70184
C	4.41599	0.13958	2.77974
H	2.35707	0.43999	3.30760
C	5.30083	-0.23478	1.77758
H	5.51952	-0.91149	-0.25034
H	4.78676	0.42226	3.75780
H	6.36657	-0.24574	1.97565

R = Mes

P	0.81724	-0.24990	1.54774
C	0.31489	-0.39943	-0.00462
P	-1.22637	-0.29338	-0.84164
Cl	1.47166	-1.77354	-2.57772
Si	1.19284	0.18326	-1.70544
O	-0.61233	0.39173	-2.09718
C	-2.45031	0.80408	-0.12109
H	-3.24697	0.95922	-0.85221
H	-2.87840	0.37524	0.78571
H	-1.97531	1.75737	0.11442
Au	-0.38219	-0.05373	3.40107
Cl	-1.64133	0.17738	5.27133
C	-2.03891	-1.85276	-1.24310
C	-2.31001	-2.77794	-0.21722
C	-2.44983	-2.12598	-2.56660
C	-2.96037	-3.96338	-0.53060
C	-3.09602	-3.33050	-2.81546
C	-3.35295	-4.26703	-1.82473
H	-3.16794	-4.66705	0.26952
H	-3.41613	-3.53774	-3.83173
C	2.61407	-0.17755	1.59096
C	3.36441	-1.35052	1.41811
C	3.23996	1.05450	1.83617
C	4.74767	-1.26006	1.49554
C	4.62503	1.08973	1.89916
C	5.39616	-0.05498	1.73560
H	5.33646	-2.16265	1.36367
H	5.11579	2.04183	2.07654
C	-1.91235	-2.56700	1.21156

H	-0.85639	-2.80536	1.36264
H	-2.05923	-1.54424	1.56284
H	-2.49396	-3.21495	1.86763
C	-2.24709	-1.20999	-3.73934
H	-2.57863	-0.18973	-3.54459
H	-1.19274	-1.14976	-4.01326
H	-2.80481	-1.59070	-4.59530
C	-4.02004	-5.56698	-2.14702
H	-4.72506	-5.45921	-2.97306
H	-3.27745	-6.31298	-2.44688
H	-4.55529	-5.96592	-1.28397
C	6.88834	0.00509	1.84257
H	7.20492	-0.11542	2.88341
H	7.36143	-0.78843	1.26203
H	7.27380	0.96456	1.49345
C	2.71895	-2.67722	1.15287
H	1.91199	-2.88209	1.86353
H	2.28939	-2.71459	0.14784
H	3.44858	-3.48290	1.23898
C	2.44790	2.31706	1.98798
H	1.94124	2.57471	1.05266
H	1.68056	2.22378	2.76329
H	3.09451	3.15230	2.25780

R = Mes*

P	-1.19686	0.73093	0.04173
C	-0.20913	-0.49676	0.50350
P	1.48836	-0.84662	0.87492
Cl	-1.17471	-3.34440	-0.71975
O	1.26194	-2.39433	0.86411
C	3.10251	-0.52324	0.08483
C	3.64058	-1.55560	-0.73465
C	3.99925	0.40148	0.68158
C	4.98979	-1.84709	-0.59311
C	5.34199	0.04868	0.76432
C	5.84881	-1.12187	0.22212
H	5.38894	-2.67690	-1.16200
H	6.01603	0.73284	1.26106
C	-2.89266	0.07419	0.11071
C	-3.51175	-0.41369	-1.07262
C	-3.49316	-0.15421	1.37319
C	-4.58075	-1.28854	-0.92348
C	-4.55427	-1.05282	1.42547
C	-5.08068	-1.67752	0.31027
H	-5.03236	-1.70231	-1.80935
H	-4.98740	-1.27137	2.39287
C	-3.14658	0.03645	-2.50214
C	-3.19620	0.55626	2.71315
C	-6.18370	-2.71903	0.46493
C	3.66326	1.85223	1.07159
C	2.89925	-2.27613	-1.87397
C	7.29413	-1.56708	0.40640
C	-4.49366	1.26604	3.14423
H	-4.33117	1.77633	4.09745
H	-4.79172	2.01387	2.40519
H	-5.32594	0.57457	3.27511
C	-2.13129	1.64838	2.68131
H	-1.12091	1.25629	2.56190
H	-2.31981	2.39305	1.90342
H	-2.14923	2.18003	3.63683
C	-2.79812	-0.46169	3.78766
H	-3.58950	-1.18845	3.97830
H	-1.90890	-1.02194	3.48933
H	-2.59047	0.05575	4.72862
C	-7.40160	-2.08655	1.14805
H	-8.19363	-2.83109	1.26692
H	-7.15757	-1.70122	2.14012
H	-7.79856	-1.25929	0.55442
C	-6.63131	-3.29415	-0.87629
H	-7.40305	-4.04902	-0.70792
H	-7.05898	-2.52763	-1.52779
H	-5.80630	-3.77709	-1.40554
C	-5.65390	-3.87023	1.32887
H	-4.77945	-4.33434	0.86702
H	-5.36170	-3.52766	2.32360
H	-6.42628	-4.63482	1.45074
C	-3.98508	-0.68281	-3.56421
H	-5.05463	-0.49527	-3.44851
H	-3.69568	-0.30333	-4.54647
H	-3.81366	-1.76205	-3.56338
C	-3.46512	1.53224	-2.62940
H	-4.52471	1.71844	-2.43784
H	-2.88769	2.14188	-1.93083
H	-3.23304	1.88111	-3.63933
C	-1.68612	-0.22881	-2.87698
H	-0.97902	0.41121	-2.34488
H	-1.41123	-1.26757	-2.68997
H	-1.54270	-0.01263	-3.93941
C	2.22884	2.25508	0.75675
H	1.98210	2.02786	-0.28272
H	1.47642	1.79360	1.39484
H	2.11263	3.33356	0.88161
C	4.53591	2.74417	0.16314
H	4.29240	3.79348	0.34788
H	5.60174	2.60719	0.34786
H	4.34324	2.53382	-0.89110
C	4.01068	2.18357	2.52568
H	5.06745	2.00739	2.73598
H	3.80914	3.24045	2.71768
H	3.43245	1.59814	3.24088
C	1.57917	-1.60186	-2.23002
H	1.17443	-2.06160	-3.13480
H	0.81175	-1.72075	-1.47027
H	1.72287	-0.53699	-2.43237
C	2.69113	-3.76486	-1.58455
H	2.04179	-3.91785	-0.72570
H	2.23328	-4.24682	-2.45244
H	3.64565	-4.26158	-1.39182
C	3.77500	-2.14967	-3.13695
H	4.00834	-1.10498	-3.35658
H	4.71336	-2.70019	-3.06304
H	3.22416	-2.55988	-3.98700
C	8.06705	-0.64777	1.34828
H	8.15665	0.36571	0.94905
H	7.59871	-0.59066	2.33416
H	9.07956	-1.03455	1.48467
C	8.00765	-1.57912	-0.95090
H	7.53246	-2.26543	-1.65435
H	8.00973	-0.58371	-1.40159
H	9.04594	-1.89773	-0.82474
C	7.30324	-2.98209	0.99736
H	6.81372	-3.00226	1.97407
H	6.78773	-3.69442	0.35036
H	8.33237	-3.32843	1.12505
Au	-0.73324	2.82226	-0.56201
Cl	-0.26242	4.97400	-1.12399
C	1.71792	-0.48292	2.63245
H	2.66615	-0.91972	2.95034
H	1.68727	0.56963	2.89285
H	0.89973	-1.00344	3.13732
Si	-0.58214	-2.38851	1.10888

Coordination through π(C=P) bond

R = H

P	0.79512	0.12658	1.06714
C	0.34430	-0.00946	-0.59215
P	-1.20474	-0.34908	-1.34696
Cl	1.26030	-2.61556	-1.91988
Si	1.15467	-0.52025	-2.38355
O	-0.68647	-0.46554	-2.79591
H	2.19726	-0.08607	0.89072
H	-1.68258	-1.58526	-0.87188
Au	0.67599	2.09378	-0.26566
Cl	0.83791	4.31669	-0.56531
C	-2.58142	0.77938	-1.20671
H	-3.40643	0.41199	-1.82150
H	-2.90089	0.83430	-0.16337
H	-2.28014	1.76981	-1.55079

R = Me

P	0.73399	0.02117	1.11523
C	0.28023	0.15562	-0.54015
P	-1.25573	-0.17984	-1.33383
Cl	1.53725	-2.14233	-2.19419
Si	1.11113	-0.02851	-2.37439
O	-0.70323	-0.15935	-2.78399
C	-2.59740	0.99304	-1.15811
H	-3.42207	0.69269	-1.80899
H	-2.94083	1.01708	-0.12199
H	-2.25139	1.98605	-1.44741
C	2.54700	-0.24168	1.03764
H	3.00821	0.18011	0.14475
H	3.00959	0.17431	1.93449
H	2.71303	-1.32399	1.03817
C	-1.88404	-1.79593	-0.89291
H	-2.21962	-1.79338	0.14640
H	-2.71787	-2.05371	-1.54931
H	-1.07851	-2.51992	-1.02323
Au	0.46610	2.18956	0.15146
Cl	0.50659	4.43691	0.31490

R = *t*-Bu

P	0.65291	0.12625	0.95636
C	0.34309	0.11622	-0.74378
P	-1.19409	-0.30341	-1.53242
Cl	2.03136	-1.92027	-2.57186
Si	1.15213	0.04541	-2.59630
O	-0.60021	-0.44549	-2.96400
C	-2.44785	0.98463	-1.53822
H	-3.30897	0.66378	-2.12749
H	-2.76437	1.22802	-0.52360
H	-2.01526	1.87383	-1.99931
Au	0.39782	2.20913	-0.16990
Cl	0.38161	4.46506	-0.09682
C	2.49249	-0.05388	1.22998
C	2.71155	-1.56676	1.34959
C	3.39004	0.53179	0.15343
C	2.76795	0.61968	2.57578
H	2.05135	-2.01987	2.09598
H	2.55765	-2.06882	0.39211
H	3.74167	-1.75423	1.66979
H	3.24613	1.60922	0.04553
H	4.43746	0.36180	0.42554
H	3.23128	0.05976	-0.81765
H	3.80796	0.44178	2.86528
H	2.61426	1.70048	2.52365
H	2.12769	0.22350	3.36891
C	-1.98348	-1.86322	-0.97792
C	-2.97847	-2.28084	-2.06490
C	-0.92248	-2.94912	-0.81339
C	-2.69971	-1.61754	0.35049
H	-3.79321	-1.56356	-2.18447
H	-2.48556	-2.41341	-3.02955
H	-3.42466	-3.23688	-1.77747
H	-0.21243	-2.70823	-0.02067
H	-1.42337	-3.88300	-0.54307
H	-0.35873	-3.11392	-1.73206
H	-3.10759	-2.56701	0.70801
H	-2.01948	-1.24195	1.11977
H	-3.53312	-0.91940	0.25006

R = Ph

P	0.70787	0.10921	1.06131
C	0.23806	0.17027	-0.59914
P	-1.39641	-0.05408	-1.22169
Cl	1.28160	-1.69721	-3.02206
Si	0.79280	0.34200	-2.53314
O	-1.05219	0.20509	-2.71308
C	-2.69387	1.05016	-0.66641
H	-3.60874	0.82537	-1.21889
H	-2.87346	0.90855	0.40118
H	-2.39086	2.08224	-0.84835
Au	0.55256	2.23973	-0.02696
Cl	0.76380	4.48273	0.11155
C	2.49179	-0.17711	1.09666
C	3.11695	0.08316	2.32006
C	3.24845	-0.66529	0.03013
C	4.47981	-0.11073	2.46513
H	2.52999	0.45064	3.15635
C	4.60917	-0.86786	0.18379
H	2.77090	-0.91171	-0.91030
C	5.22639	-0.58527	1.39561
H	4.95974	0.10520	3.41253
H	5.19097	-1.24960	-0.64717
H	6.29307	-0.74256	1.50840
C	-1.99050	-1.72608	-0.98206
C	-2.10542	-2.57675	-2.07720
C	-2.34516	-2.16792	0.29218
C	-2.58465	-3.86517	-1.89506
H	-1.80927	-2.23038	-3.05953
C	-2.81875	-3.45669	0.46567
H	-2.23964	-1.51571	1.15344
C	-2.94062	-4.30335	-0.62881
H	-2.67248	-4.52982	-2.74629
H	-3.09053	-3.80297	1.45582
H	-3.31093	-5.31267	-0.49034

R = Mes

P	0.68804	0.13128	1.43370
C	0.05059	0.22346	-0.18946
P	-1.59992	-0.03189	-0.78819
Cl	1.42934	-0.10540	-3.26185
Si	0.36921	1.13173	-1.91808
O	-1.34122	0.63043	-2.18553
C	-2.96584	0.87373	-0.04952
H	-3.87366	0.66980	-0.62174
H	-3.12187	0.60022	0.99196
H	-2.72500	1.93669	-0.11161
Au	0.35107	2.30928	0.35255
Cl	0.71241	4.45003	1.06130
C	2.48223	-0.12355	1.25270
C	3.35046	0.68140	2.01378

C	2.99684	-1.20462	0.50992
C	4.71856	0.44934	1.94146
C	4.37081	-1.39652	0.47937
C	5.25218	-0.56897	1.16542
H	5.38447	1.08256	2.52030
H	4.76500	-2.23413	-0.08888
C	-2.04563	-1.77445	-0.92831
C	-2.15591	-2.42557	-2.17843
C	-2.28649	-2.49279	0.26102
C	-2.46690	-3.78040	-2.18369
C	-2.58206	-3.84674	0.18630
C	-2.66820	-4.51531	-1.02459
H	-2.55361	-4.27908	-3.14378
H	-2.75551	-4.39034	1.10994
C	2.85096	1.78461	2.89615
H	2.59312	2.68139	2.32443
H	1.94781	1.49561	3.44139
H	3.61261	2.06065	3.62687
C	2.12181	-2.19530	-0.20073
H	1.26126	-2.48672	0.40762
H	1.73259	-1.79505	-1.13818
H	2.69045	-3.09742	-0.43160
C	6.73205	-0.77569	1.07114
H	7.24611	-0.38881	1.95266
H	6.98143	-1.83306	0.96235
H	7.13886	-0.25398	0.19899
C	-2.29516	-1.87223	1.62848
H	-2.05968	-2.62438	2.38229
H	-1.57150	-1.06522	1.75558
H	-3.28873	-1.48000	1.86670
C	-1.98279	-1.77018	-3.51887
H	-2.62132	-0.89539	-3.63893
H	-0.95777	-1.43462	-3.67978
H	-2.23259	-2.48574	-4.30239
C	-2.95248	-5.98304	-1.08168
H	-3.51719	-6.24452	-1.97814
H	-2.01805	-6.55229	-1.10720
H	-3.51504	-6.31552	-0.20797

R = Mes*

P	0.69548	0.30887	1.03399
C	0.42064	0.04818	-0.68126
P	-1.14090	-0.11502	-1.60902
O	-0.40206	-0.00921	-2.99495
Cl	2.55122	-0.81734	-3.24373
Au	0.70342	2.22329	-0.54862
Cl	0.73816	4.50505	-0.31847
C	2.52143	0.08518	1.21392
C	3.17739	-1.09024	0.75286
C	3.32209	1.16717	1.66389
C	4.51653	-0.99728	0.40587
C	4.66208	1.18760	1.27298
C	5.26346	0.16340	0.56522
H	5.00741	-1.87859	0.01319
H	5.25543	2.04644	1.55177
C	2.94133	2.25047	2.69828
C	2.62373	-2.52768	0.84781
C	6.69432	0.23534	0.04525
C	3.78369	1.92518	3.94868
H	3.54275	0.93149	4.33581
H	4.85408	1.95343	3.74024
H	3.57111	2.65808	4.73210
C	3.29732	3.66210	2.22171
H	2.73529	3.93918	1.32953
H	3.04774	4.38084	3.00733
H	4.36163	3.77380	2.00877
C	1.48920	2.26585	3.16749
H	1.41014	2.95216	4.01546
H	0.80146	2.62483	2.40209
H	1.15674	1.28422	3.51470
C	2.91386	-3.34622	-0.41372
H	2.51108	-2.86182	-1.30208
H	3.98281	-3.49774	-0.57111
H	2.46021	-4.33712	-0.32118
C	3.37119	-3.17349	2.03138
H	4.45096	-3.16830	1.87568
H	3.16433	-2.63922	2.96219
H	3.04752	-4.21164	2.15282
C	1.13857	-2.66112	1.16739
H	0.85123	-2.07789	2.04792
H	0.50362	-2.37224	0.33228
H	0.92372	-3.70913	1.39624
C	7.34250	1.58915	0.32185
H	6.77623	2.40798	-0.12848
H	7.43883	1.78493	1.39300
H	8.34796	1.60607	-0.10576
C	6.67619	0.01009	-1.47152
H	7.69403	0.04654	-1.87037
H	6.24668	-0.95985	-1.73015
H	6.08375	0.77829	-1.97378
C	7.54753	-0.85146	0.70961
H	7.57130	-0.72186	1.79457
H	7.16752	-1.85309	0.49846
H	8.57500	-0.80174	0.33813
C	-2.36034	-1.48995	-1.68529
C	-2.35803	-2.33509	-2.83554
C	-3.57682	-1.34904	-0.96221
C	-3.58609	-2.65493	-3.39850
C	-1.13976	-3.05978	-3.43831
C	-4.76118	-1.74278	-1.57643
C	-3.71788	-0.96127	0.52138
C	-4.80693	-2.30393	-2.84107
H	-3.58218	-3.23290	-4.31297
C	-0.71171	-2.47968	-4.78982
C	-1.52867	-4.53357	-3.67821
C	0.01813	-3.12121	-2.45107
H	-5.68420	-1.60332	-1.03163
C	-2.41025	-0.58368	1.19428
C	-4.21021	-2.23378	1.23933
C	-4.74576	0.15057	0.75546
C	-6.10766	-2.62137	-3.56747
H	0.09141	-3.09051	-5.21064
H	-1.54758	-2.49695	-5.49450
H	-0.35569	-1.45724	-4.70480
H	-2.22516	-4.66468	-4.50717
H	-0.62417	-5.09225	-3.93122
H	-1.96673	-4.98476	-2.78482
H	0.40721	-2.14939	-2.16249
H	-0.28081	-3.65729	-1.54676
H	0.85263	-3.66251	-2.90134
H	-1.64375	-1.34481	1.03883
H	-2.01386	0.38175	0.87997
H	-2.56270	-0.49834	2.27261
H	-4.30605	-2.03273	2.30979
H	-5.18072	-2.56644	0.86934
H	-3.49962	-3.05331	1.10900
H	-5.74156	-0.12627	0.40608
H	-4.82109	0.35568	1.82629
H	-4.46978	1.08215	0.25889
C	-7.33320	-2.14058	-2.79507
C	-6.22764	-4.13659	-3.77091
C	-6.09339	-1.92508	-4.93357

H	-7.43822	-2.65162	-1.83460
H	-7.30071	-1.06360	-2.61187
H	-8.23457	-2.35038	-3.37541
H	-5.40405	-4.53368	-4.36734
H	-6.23261	-4.66165	-2.81260
H	-7.15970	-4.37124	-4.29201
H	-6.02356	-0.84051	-4.82029
H	-5.25145	-2.25264	-5.54652
H	-7.01322	-2.15136	-5.47936
Si	1.20264	0.57748	-2.42095
C	-2.23250	1.32893	-1.62105
H	-2.69199	1.53764	-0.65973
H	-1.63108	2.18764	-1.92554
H	-3.00931	1.14599	-2.36611

[R-P=C(Si(II)Cl)-P(S)ClR](AuCl)
Coordination through Si(II) atom
R= H

P	0.80758	0.23345	0.90815
C	0.45042	-0.22531	-0.64820
P	-1.12771	-0.40431	-1.37393
Cl	1.62019	-2.36199	-2.58764
Si	1.44177	-0.30935	-2.29543
H	2.22214	0.31452	0.72733
H	-1.62238	-1.71540	-1.25451
Cl	-2.62195	0.62927	-0.53398
Au	3.10492	1.10808	-2.77799
Cl	4.80924	2.57396	-3.19624
S	-0.66480	0.10217	-3.24505

R = Me

P	0.76179	-0.00067	0.91748
C	0.41357	-0.26170	-0.68802
P	-1.17846	-0.31583	-1.40131
Cl	1.61948	-2.32676	-2.70947
Si	1.38539	-0.28213	-2.34017
Cl	-2.51488	0.95595	-0.59090
Au	3.07791	1.13023	-2.76865
Cl	4.81971	2.57944	-3.10920
C	2.58477	-0.03790	0.97993
H	2.90913	-1.07902	1.07861
H	3.03866	0.38429	0.07887
H	2.92410	0.50684	1.86197
C	-1.98001	-1.91411	-1.25799
H	-2.19869	-2.11728	-0.20825
H	-2.90133	-1.91045	-1.84215
H	-1.28757	-2.65984	-1.65389
S	-0.67388	0.15939	-3.28631

R = *t*-Bu

P	0.70253	0.01502	0.69331
C	0.51903	-0.38683	-0.91433
P	-1.08418	-0.50519	-1.63609
Cl	2.08596	-2.51099	-2.69844
Si	1.52121	-0.50793	-2.55007
Au	2.95680	1.10034	-3.19461
Cl	4.41786	2.77569	-3.76863
C	2.51001	0.16017	1.10250
C	3.43605	-0.76292	0.32686
C	2.85896	1.63254	0.84697
C	2.59877	-0.13831	2.60235
H	3.13240	-1.80938	0.39795
H	3.49675	-0.48859	-0.72828
H	4.44928	-0.67825	0.73317
H	2.18364	2.31405	1.37325
H	3.87128	1.82475	1.21706
H	2.83947	1.87465	-0.21782
H	3.61979	0.04948	2.94681
H	1.92629	0.49588	3.18628
H	2.35892	-1.18186	2.82354
C	-2.12741	-1.95376	-1.15703
C	-3.36068	-1.96868	-2.05785
C	-1.28503	-3.20833	-1.38763
C	-2.52237	-1.83850	0.31264
H	-3.99161	-1.09180	-1.90477
H	-3.08959	-2.02804	-3.11419
H	-3.95203	-2.85568	-1.81545
H	-0.39017	-3.22279	-0.76370
H	-1.89522	-4.07752	-1.12785
H	-0.97571	-3.30869	-2.42874
H	-3.08919	-2.73323	0.58430
H	-1.65103	-1.78594	0.96921
H	-3.15258	-0.96916	0.50262
Cl	-2.23617	1.10243	-1.20304
S	-0.53870	-0.50535	-3.57266

R = Ph

P	0.83732	-0.27336	0.43446
C	0.47692	-0.23906	-1.19781
P	-1.19827	-0.36769	-1.70291
Cl	1.98099	-2.21196	-3.10287
Si	1.25955	-0.25932	-2.94443
Au	2.47978	1.41744	-3.81620
Cl	3.75887	3.13439	-4.64503
C	-2.13270	-1.74526	-1.02609
C	-1.80242	-3.01518	-1.50175
C	-3.09101	-1.59450	-0.02783
C	-2.43712	-4.12729	-0.97739
H	-1.05470	-3.12973	-2.27890
C	-3.72021	-2.71568	0.48925
H	-3.34973	-0.61044	0.34088
C	-3.39537	-3.97811	0.01602
H	-2.18467	-5.11300	-1.34946
H	-4.46990	-2.59937	1.26279
H	-3.89352	-4.85133	0.42122
C	2.61112	-0.19034	0.64956
C	3.03610	-0.48469	1.95274
C	3.56172	0.16496	-0.31381
C	4.38117	-0.45507	2.27705
H	2.30084	-0.74023	2.70967
C	4.90165	0.20866	0.01853
H	3.25932	0.43741	-1.31963
C	5.31269	-0.10834	1.30910
H	4.70221	-0.69337	3.28414
H	5.62901	0.49551	-0.73166
H	6.36659	-0.07536	1.56125
Cl	-2.26908	1.27079	-1.20354
S	-0.90406	-0.48310	-3.68576

R = Mes

P	-0.41725	1.47157	1.44297
C	0.14893	0.40405	0.28083
P	1.76859	-0.27762	0.07630
Cl	-1.20971	0.76900	-2.62306
C	3.46693	0.39792	0.16124
C	4.09194	0.72275	-1.08042
C	4.29397	0.11910	1.28266
C	5.41001	0.32717	-1.24719
C	5.61329	-0.24177	1.03208
C	6.16692	-0.25676	-0.23851
H	5.87436	0.51136	-2.20793

H	6.23155	-0.51086	1.87706
C	-2.21104	1.52784	1.09096
C	-2.73555	2.57944	0.30413
C	-3.06373	0.48274	1.53491
C	-3.99723	2.39924	-0.25677
C	-4.30329	0.36775	0.92095
C	-4.76384	1.26656	-0.03268
H	-4.38834	3.16634	-0.90559
H	-4.93404	-0.46666	1.18871
C	-2.04088	3.94656	0.14673
C	-2.79727	-0.35998	2.79849
C	-6.08240	1.00234	-0.75020
C	3.94630	0.38968	2.76032
C	3.53244	1.67149	-2.16027
C	7.56759	-0.77795	-0.52958
C	-3.82441	-1.48231	2.97404
H	-3.57785	-2.04430	3.87780
H	-4.83674	-1.09540	3.10723
H	-3.82126	-2.18196	2.13436
C	-2.94270	0.60195	3.98999
H	-2.19369	1.39820	3.95604
H	-3.92996	1.06946	3.99673
H	-2.81551	0.05570	4.92921
C	-1.41982	-1.01196	2.88132
H	-1.19876	-1.62316	2.00510
H	-0.61241	-0.27983	3.00739
H	-1.38145	-1.65078	3.76796
C	-7.21671	0.86335	0.27187
H	-8.16200	0.67454	-0.24421
H	-7.04601	0.03318	0.95945
H	-7.32797	1.77633	0.86289
C	-6.44858	2.12261	-1.72009
H	-7.38641	1.87448	-2.22266
H	-6.59268	3.07809	-1.20797
H	-5.68661	2.25501	-2.49226
C	-5.94909	-0.30356	-1.54441
H	-5.15600	-0.22547	-2.29194
H	-5.71076	-1.15425	-0.90303
H	-6.88692	-0.52317	-2.06268
C	-2.89390	4.93397	-0.65324
H	-3.87309	5.09599	-0.19795
H	-2.38072	5.89801	-0.68090
H	-3.03902	4.60911	-1.68621
C	-1.84998	4.54861	1.54658
H	-2.80825	4.64670	2.06175
H	-1.19461	3.93656	2.17141
H	-1.39857	5.54199	1.46939
C	-0.68634	3.87222	-0.55726
H	0.06780	3.35602	0.04417
H	-0.75645	3.35629	-1.51536
H	-0.30518	4.88348	-0.72777
C	2.54176	0.92968	2.99001
H	2.32565	1.77690	2.33536
H	1.76577	0.17702	2.86542
H	2.45886	1.29140	4.01776
C	4.90015	1.52659	3.18774
H	4.67966	1.80975	4.22037
H	5.94803	1.22986	3.13619
H	4.76636	2.40872	2.55701
C	4.19524	-0.81136	3.67576
H	5.22186	-1.17488	3.60378
H	4.02540	-0.51566	4.71429
H	3.52641	-1.64035	3.44809
C	2.17291	2.26896	-1.82225
H	1.95521	3.07604	-2.52606
H	1.35287	1.56064	-1.90763

H	2.17080	2.70168	-0.81892
C	3.49722	1.04816	-3.55693
H	2.77620	0.23395	-3.61463
H	3.20790	1.80888	-4.28655
H	4.47443	0.66182	-3.85413
C	4.50986	2.86688	-2.19029
H	4.58759	3.33614	-1.20647
H	5.51267	2.58090	-2.50872
H	4.13497	3.61406	-2.89456
C	8.22288	-1.39357	0.70376
H	8.38471	-0.65536	1.49343
H	7.62689	-2.21312	1.11283
H	9.20036	-1.79841	0.43216
C	8.45420	0.36977	-1.02800
H	8.06860	0.81300	-1.94818
H	8.53080	1.16118	-0.27840
H	9.46164	-0.00068	-1.23525
C	7.47226	-1.85598	-1.61635
H	6.85461	-2.69384	-1.28461
H	7.03776	-1.46431	-2.53818
H	8.46873	-2.23912	-1.85149
Au	-2.17665	-2.25447	-0.73624
Cl	-3.75426	-3.88037	-0.27157
Si	-0.70127	-0.59297	-1.11871
Cl	1.87381	-1.86933	1.35852
S	1.42942	-1.15707	-1.71271

R = Mes*

P	-0.41725	1.47157	1.44297
C	0.14893	0.40405	0.28083
P	1.76859	-0.27762	0.07630
Cl	-1.20971	0.76900	-2.62306
C	3.46693	0.39792	0.16124
C	4.09194	0.72275	-1.08042
C	4.29397	0.11910	1.28266
C	5.41001	0.32717	-1.24719
C	5.61329	-0.24177	1.03208
C	6.16692	-0.25676	-0.23851
H	5.87436	0.51136	-2.20793
H	6.23155	-0.51086	1.87706
C	-2.21104	1.52784	1.09096
C	-2.73555	2.57944	0.30413
C	-3.06373	0.48274	1.53491
C	-3.99723	2.39924	-0.25677
C	-4.30329	0.36775	0.92095
C	-4.76384	1.26656	-0.03268
H	-4.38834	3.16634	-0.90559
H	-4.93404	-0.46666	1.18871
C	-2.04088	3.94656	0.14673
C	-2.79727	-0.35998	2.79849
C	-6.08240	1.00234	-0.75020
C	3.94630	0.38968	2.76032
C	3.53244	1.67149	-2.16027
C	7.56759	-0.77795	-0.52958
C	-3.82441	-1.48231	2.97404
H	-3.57785	-2.04430	3.87780
H	-4.83674	-1.09540	3.10723
H	-3.82126	-2.18196	2.13436
C	-2.94270	0.60195	3.98999
H	-2.19369	1.39820	3.95604
H	-3.92996	1.06946	3.99673
H	-2.81551	0.05570	4.92921
C	-1.41982	-1.01196	2.88132
H	-1.19876	-1.62316	2.00510
H	-0.61241	-0.27983	3.00739
H	-1.38145	-1.65078	3.76796

C	-7.21671	0.86335	0.27187
H	-8.16200	0.67454	-0.24421
H	-7.04601	0.03318	0.95945
H	-7.32797	1.77633	0.86289
C	-6.44858	2.12261	-1.72009
H	-7.38641	1.87448	-2.22266
H	-6.59268	3.07809	-1.20797
H	-5.68661	2.25501	-2.49226
C	-5.94909	-0.30356	-1.54441
H	-5.15600	-0.22547	-2.29194
H	-5.71076	-1.15425	-0.90303
H	-6.88692	-0.52317	-2.06268
C	-2.89390	4.93397	-0.65324
H	-3.87309	5.09599	-0.19795
H	-2.38072	5.89801	-0.68090
H	-3.03902	4.60911	-1.68621
C	-1.84998	4.54861	1.54658
H	-2.80825	4.64670	2.06175
H	-1.19461	3.93656	2.17141
H	-1.39857	5.54199	1.46939
C	-0.68634	3.87222	-0.55726
H	0.06780	3.35602	0.04417
H	-0.75645	3.35629	-1.51536
H	-0.30518	4.88348	-0.72777
C	2.54176	0.92968	2.99001
H	2.32565	1.77690	2.33536
H	1.76577	0.17702	2.86542
H	2.45886	1.29140	4.01776
C	4.90015	1.52659	3.18774
H	4.67966	1.80975	4.22037
H	5.94803	1.22986	3.13619
H	4.76636	2.40872	2.55701
C	4.19524	-0.81136	3.67576
H	5.22186	-1.17488	3.60378
H	4.02540	-0.51566	4.71429
H	3.52641	-1.64035	3.44809
C	2.17291	2.26896	-1.82225
H	1.95521	3.07604	-2.52606
H	1.35287	1.56064	-1.90763
H	2.17080	2.70168	-0.81892
C	3.49722	1.04816	-3.55693
H	2.77620	0.23395	-3.61463
H	3.20790	1.80888	-4.28655
H	4.47443	0.66182	-3.85413
C	4.50986	2.86688	-2.19029
H	4.58759	3.33614	-1.20647
H	5.51267	2.58090	-2.50872
H	4.13497	3.61406	-2.89456
C	8.22288	-1.39357	0.70376
H	8.38471	-0.65536	1.49343
H	7.62689	-2.21312	1.11283
H	9.20036	-1.79841	0.43216
C	8.45420	0.36977	-1.02800
H	8.06860	0.81300	-1.94818
H	8.53080	1.16118	-0.27840
H	9.46164	-0.00068	-1.23525
C	7.47226	-1.85598	-1.61635
H	6.85461	-2.69384	-1.28461
H	7.03776	-1.46431	-2.53818
H	8.46873	-2.23912	-1.85149
Au	-2.17665	-2.25447	-0.73624
Cl	-3.75426	-3.88037	-0.27157
Si	-0.70127	-0.59297	-1.11871
Cl	1.87381	-1.86933	1.35852
S	1.42942	-1.15707	-1.71271

Coordination through S atom

R= H

P	0.67279	-0.20066	1.16497
C	0.44800	-0.32977	-0.47563
P	-0.97740	0.06193	-1.38101
Cl	0.89512	-2.50126	-2.61514
Si	1.67535	-0.63102	-1.99615
H	2.03934	-0.62646	1.17921
H	-1.66032	-1.10827	-1.73748
Cl	-2.42535	1.14322	-0.50417
Au	-1.41067	0.36058	-4.75462
Cl	-2.72939	-0.12431	-6.50973
S	-0.08309	0.90064	-2.99328

R = Me

P	0.67681	-0.17002	1.11235
C	0.43945	-0.22874	-0.53344
P	-1.05513	0.07553	-1.35406
Cl	1.30900	-2.31252	-2.69608
Si	1.66345	-0.31404	-2.07018
Cl	-2.34625	1.33202	-0.42956
Au	-1.49810	0.07268	-4.80994
Cl	-2.72340	-0.67168	-6.54508
C	2.42455	-0.68402	1.29728
H	2.82669	-0.27656	2.22602
H	2.46464	-1.77572	1.36227
H	3.04089	-0.36718	0.45106
C	-2.01837	-1.40834	-1.62766
H	-2.87639	-1.16796	-2.25691
H	-1.37537	-2.13596	-2.12650
H	-2.34656	-1.78662	-0.65728
S	-0.27412	0.86586	-3.06640

R = *t*-Bu

P	0.78161	-0.09987	0.84218
C	0.42111	0.03957	-0.78366
P	-1.25226	0.00881	-1.30077
Cl	1.81579	-1.40873	-3.20475
Si	1.34141	0.52221	-2.45440
Au	-1.98668	-0.36572	-4.83254
Cl	-2.94074	-1.46573	-6.55261
C	2.62686	-0.29410	1.04022
C	2.88745	-1.80353	0.98024
C	3.47713	0.43658	0.01555
C	2.93569	0.22659	2.44673
H	2.27013	-2.35600	1.69505
H	2.70104	-2.20134	-0.01945
H	3.93486	-1.99703	1.23592
H	3.23461	1.49964	-0.04409
H	4.53368	0.35111	0.29259
H	3.38302	0.00184	-0.98342
H	3.98456	0.03002	2.68755
H	2.77053	1.30437	2.52408
H	2.31968	-0.26333	3.20552
C	-2.10952	-1.63902	-1.20615
C	-3.51915	-1.50612	-1.77707
C	-1.29251	-2.65637	-1.99784
C	-2.17553	-2.04922	0.26468
H	-4.13064	-0.80724	-1.20501
H	-3.51291	-1.20018	-2.82572
H	-3.99298	-2.48997	-1.72768
H	-0.27826	-2.76147	-1.61088
H	-1.79433	-3.62403	-1.91335
H	-1.23296	-2.40438	-3.05782
H	-2.66229	-3.02722	0.30903

H	-1.18573	-2.15118	0.71302
H	-2.76226	-1.35336	0.86522
Cl	-2.44016	1.24239	-0.20299
S	-1.00208	0.82655	-3.16346

R = Ph

P	0.85225	-0.27979	1.22111
C	0.36695	-0.21146	-0.37789
P	-1.28921	-0.09879	-0.86570
Cl	1.27007	-1.52022	-3.12529
Si	1.27005	0.31014	-2.04052
Au	-2.36719	0.41508	-4.19131
Cl	-3.72758	-0.20641	-5.87957
C	2.63978	-0.43882	1.13873
C	3.34599	0.03149	2.25277
C	3.35044	-0.98484	0.06341
C	4.72895	-0.00010	2.27166
H	2.80118	0.43623	3.10016
C	4.73443	-1.02235	0.09054
H	2.82161	-1.42996	-0.77249
C	5.42277	-0.52346	1.18811
H	5.26809	0.37814	3.13225
H	5.27716	-1.44914	-0.74470
H	6.50619	-0.55554	1.20533
C	-2.04951	-1.67929	-1.21337
C	-3.37615	-1.72835	-1.64171
C	-1.30395	-2.84597	-1.07485
C	-3.95044	-2.95082	-1.93373
H	-3.95620	-0.81970	-1.75310
C	-1.89329	-4.06656	-1.36521
H	-0.27097	-2.79890	-0.75616
C	-3.20958	-4.11811	-1.79547
H	-4.97578	-2.99113	-2.28019
H	-1.31551	-4.97730	-1.26325
H	-3.66339	-5.07388	-2.03069
Cl	-2.54553	0.78024	0.46627
S	-1.02738	1.11655	-2.49466

R = Mes

P	0.71571	0.24598	1.25883
C	0.38582	-0.18340	-0.30738
P	-1.06333	-0.05692	-1.25537
Au	-1.83996	1.49155	-4.39522
Cl	-3.36242	2.23507	-5.88403
C	2.52759	-0.05626	1.31607
C	3.03894	-1.35898	1.45599
C	3.39855	1.04772	1.28068
C	4.41894	-1.52917	1.50600
C	4.76808	0.82498	1.33404
C	5.29915	-0.45637	1.43717
H	4.81535	-2.53406	1.61621
H	5.43951	1.67810	1.30320
C	-1.94944	-1.63783	-1.33403
C	-2.53423	-2.10152	-0.13079
C	-2.02023	-2.43459	-2.50241
C	-3.16530	-3.33831	-0.12618
C	-2.67817	-3.65542	-2.42220
C	-3.25851	-4.13066	-1.25726
H	-3.60151	-3.68686	0.80463
H	-2.72780	-4.26241	-3.32007
C	2.87060	2.44863	1.17520
H	2.42502	2.64383	0.19459
H	2.09006	2.64112	1.91778
H	3.66712	3.17750	1.32803
C	2.13963	-2.55425	1.57062
H	1.30224	-2.36219	2.24745
H	1.71563	-2.83403	0.60181
H	2.69271	-3.41350	1.95155
C	6.78054	-0.67369	1.46574
H	7.17487	-0.78262	0.45059
H	7.29791	0.16890	1.92768
H	7.03934	-1.58044	2.01466
C	-2.56572	-1.35020	1.16785
H	-1.65734	-0.78716	1.37847
H	-3.39419	-0.63857	1.18515
H	-2.71338	-2.05196	1.98946
C	-1.44592	-2.08892	-3.84203
H	-1.90244	-1.19230	-4.26517
H	-0.36975	-1.92511	-3.79598
H	-1.62721	-2.91180	-4.53291
C	-3.97383	-5.44384	-1.23278
H	-4.07905	-5.82296	-0.21544
H	-4.97839	-5.33997	-1.65423
H	-3.44835	-6.19198	-1.82939
S	-0.25854	0.79151	-2.91487
Si	1.87903	-0.32707	-1.53754
Cl	1.70243	-2.44842	-1.17108
Cl	-2.42924	1.29469	-0.57050

R = Mes*

P	-2.09743	1.77458	0.43722
C	-1.13484	0.42349	0.24066
P	0.61545	0.33811	0.30741
C	-3.78386	1.04855	0.38371
C	-4.57682	1.25388	-0.78090
C	-4.25147	0.18768	1.40014
C	-5.65247	0.40760	-0.98784
C	-5.33655	-0.64216	1.10541
C	-6.01282	-0.59999	-0.09652
H	-6.23672	0.52113	-1.88893
H	-5.66683	-1.33423	1.86715
C	-3.82863	0.19516	2.88495
C	-4.33573	2.40049	-1.78092
C	-7.14468	-1.55828	-0.44888
C	-3.03098	2.22793	-2.56023
H	-2.14967	2.31079	-1.91958
H	-2.98665	1.25422	-3.05104
H	-2.94604	3.01151	-3.31902
C	-4.32432	3.73704	-1.02596
H	-5.26838	3.89026	-0.49763
H	-3.51524	3.80017	-0.29550
H	-4.19091	4.56151	-1.73197
C	-5.45463	2.49709	-2.82161
H	-5.48593	1.62409	-3.47760
H	-6.43672	2.62445	-2.36047
H	-5.27015	3.37004	-3.45185
C	-3.46618	-1.20730	3.38455
H	-2.61841	-1.61781	2.83189
H	-3.19776	-1.16140	4.44356
H	-4.29600	-1.90906	3.28826
C	-2.67599	1.12682	3.24421
H	-1.71224	0.74983	2.90522
H	-2.82441	2.14223	2.85829
H	-2.61967	1.21554	4.33301
C	-5.05067	0.70483	3.67272
H	-5.31986	1.71781	3.36234
H	-5.92467	0.06839	3.53059
H	-4.81790	0.72500	4.74104
C	-7.38524	-2.59639	0.64368
H	-7.70092	-2.13569	1.58347
H	-8.18029	-3.27705	0.33023

H	-6.49238	-3.19659	0.83533
C	-6.78100	-2.29763	-1.74264
H	-6.64287	-1.60734	-2.57716
H	-5.85414	-2.86313	-1.62310
H	-7.57840	-2.99551	-2.01286
C	-8.44277	-0.77052	-0.66075
H	-8.34854	-0.04053	-1.46722
H	-9.25670	-1.45214	-0.92287
H	-8.72798	-0.23354	0.24743
C	2.00259	1.44524	-0.08179
C	2.62959	1.24303	-1.34871
C	2.75778	2.03446	0.96815
C	4.01419	1.22903	-1.37093
C	4.14206	1.98733	0.86046
C	4.79870	1.47885	-0.25063
H	4.50445	1.02028	-2.31338
H	4.72758	2.37374	1.68326
C	1.92891	1.26272	-2.72235
C	2.27230	0.05466	-3.59622
C	2.46671	2.53216	-3.41830
H	1.87928	-0.87016	-3.17582
H	1.83279	0.18949	-4.58800
H	3.34934	-0.06364	-3.72714
H	2.25008	3.42572	-2.82755
H	3.54357	2.48953	-3.58376
H	1.97961	2.64028	-4.39102
C	2.19583	2.90955	2.10583
C	2.74925	4.32323	1.82305
C	2.67208	2.48157	3.49598
H	2.44318	4.67363	0.83442
H	2.35474	5.01894	2.56856
H	3.83789	4.35763	1.87180
H	2.28693	1.50144	3.77409
H	3.76080	2.44613	3.56146
H	2.32275	3.20537	4.23702
C	6.30709	1.28925	-0.31069
C	6.90569	2.26971	-1.32703
C	6.60506	-0.14997	-0.75063
H	6.70670	3.30589	-1.04141
H	7.98910	2.13357	-1.38040
H	6.50191	2.11178	-2.32928
H	6.17589	-0.88020	-0.06090
H	6.20446	-0.36149	-1.74403
H	7.68625	-0.30738	-0.78865
Cl	-2.40777	-1.48135	-1.91246
Au	2.62671	-2.51453	-0.03443
Cl	4.53021	-3.56046	0.59365
C	0.67922	3.05784	2.10241
H	0.30660	3.35242	1.11878
H	0.15582	2.15964	2.42390
H	0.39753	3.84786	2.80305
C	0.41637	1.43981	-2.66377
H	-0.11938	0.55019	-2.33621
H	0.14233	2.27950	-2.01999
H	0.04991	1.66675	-3.66798
C	6.97025	1.52660	1.04344
H	6.86368	2.56158	1.37954
H	6.56228	0.86598	1.81218
H	8.03984	1.32033	0.96365
Si	-1.58132	-1.49992	0.05765
Cl	0.99377	-0.25214	2.22829
S	0.77182	-1.40902	-0.75045

Coordination through P(sp^2) atom

R= H

P	0.47942	0.12544	1.07803
C	0.28697	-0.28316	-0.49835
P	-1.11822	-0.25028	-1.51278
Cl	1.18298	-2.74599	-2.10429
Si	1.59238	-0.65395	-1.95867
H	1.85957	-0.06017	1.31774
H	-1.72137	-1.51833	-1.49207
Cl	-2.65374	0.91380	-0.93690
Au	-0.90522	0.81178	2.63466
Cl	-2.32013	1.50465	4.24345
S	-0.28376	0.20140	-3.24904

R = Me

P	0.52275	0.13529	1.02100
C	0.31462	-0.23121	-0.56519
P	-1.13965	-0.27494	-1.51213
Cl	1.49082	-2.71429	-1.98725
Si	1.59065	-0.57051	-2.03851
Cl	-2.50143	1.15255	-1.06253
Au	-0.97595	0.71761	2.52423
Cl	-2.51275	1.27341	4.08154
C	2.26098	-0.08347	1.46118
H	2.37772	-1.04930	1.96015
H	2.88987	-0.06121	0.56867
H	2.55701	0.70010	2.15982
C	-2.03634	-1.80785	-1.26688
H	-2.86517	-1.84904	-1.97509
H	-1.33727	-2.62577	-1.45352
H	-2.41194	-1.84743	-0.24282
S	-0.35923	-0.07210	-3.33326

R = *t*-Bu

P	0.81540	0.07919	0.74493
C	0.55418	-0.56664	-0.75224
P	-0.99103	-0.65528	-1.56904
Cl	1.97572	-3.06895	-2.13851
Si	1.72297	-0.95036	-2.31544
Au	-0.67962	1.01940	2.07693
Cl	-2.20695	1.97966	3.44388
C	2.60032	0.05108	1.24105
C	2.66850	0.40493	2.72530
C	3.21782	-1.32285	1.00024
C	3.31114	1.12681	0.41258
H	2.22901	1.38251	2.93533
H	2.15845	-0.33536	3.34588
H	3.71850	0.43455	3.02983
H	3.22237	-1.60307	-0.05329
H	4.25651	-1.30398	1.34441
H	2.69552	-2.10666	1.55345
H	4.36051	1.16846	0.72095
H	3.28253	0.90435	-0.65677
H	2.87737	2.11648	0.57578
C	-2.13721	-2.00170	-1.01289
C	-1.38708	-3.32694	-1.12387
C	-2.57433	-1.75729	0.42733
C	-3.33888	-1.99529	-1.95685
H	-1.05793	-3.52786	-2.14383
H	-0.51185	-3.36096	-0.47383
H	-2.06911	-4.12576	-0.81978
H	-3.10589	-0.81310	0.55073
H	-3.25235	-2.56485	0.71743
H	-1.73053	-1.77433	1.11966
H	-3.98454	-2.83592	-1.68917
H	-3.92507	-1.07952	-1.87025
H	-3.03649	-2.12513	-2.99826
Cl	-2.08403	1.04287	-1.34846

S	-0.36656	-0.86186	-3.45645

R = Ph

P	0.74722	-0.08728	0.96545
C	0.37310	-0.13181	-0.63997
P	-1.22778	-0.13956	-1.32609
Cl	1.68976	-1.92976	-2.87170
Si	1.38497	0.12412	-2.31059
Au	-0.63619	0.11516	2.67712
Cl	-2.07220	0.26264	4.41865
C	-2.02679	-1.74481	-1.20950
C	-2.65100	-2.13423	-0.02448
C	-1.95740	-2.61578	-2.29247
C	-3.20732	-3.39758	0.06784
H	-2.71114	-1.45704	0.81996
C	-2.52043	-3.87813	-2.18865
H	-1.46407	-2.30641	-3.20568
C	-3.14292	-4.26789	-1.01286
H	-3.69591	-3.70049	0.98619
H	-2.47006	-4.55720	-3.03139
H	-3.58325	-5.25545	-0.93622
C	2.49807	-0.22357	1.24085
C	3.37166	-0.78643	0.30511
C	2.99718	0.27336	2.44946
C	4.72939	-0.82180	0.57087
H	2.99083	-1.23639	-0.60406
C	4.35591	0.23637	2.70339
H	2.31502	0.69261	3.18186
C	5.22147	-0.30657	1.76257
H	5.40455	-1.26273	-0.15276
H	4.74071	0.62796	3.63742
H	6.28588	-0.33943	1.96513
Cl	-2.50910	1.12169	-0.39320
S	-0.81934	0.44411	-3.18547

R = Mes

P	0.84042	-0.17994	1.38500
C	0.50540	-0.56781	-0.17847
P	-0.99400	-0.45776	-1.07293
Cl	1.66376	-2.58462	-2.22608
Si	1.79284	-0.50250	-1.67813
Au	-0.53706	0.16100	3.07518
Cl	-1.91945	0.46720	4.83948
C	-1.98352	-1.93825	-1.37123
C	-1.83991	-3.11229	-0.60463
C	-2.94317	-1.87701	-2.40551
C	-2.62998	-4.20791	-0.92443
C	-3.70521	-3.00717	-2.66720
C	-3.55846	-4.18676	-1.95417
H	-2.51679	-5.11034	-0.33240
H	-4.44564	-2.95411	-3.45913
C	2.62422	-0.11522	1.61553
C	3.34441	-1.28746	1.88206
C	3.25599	1.13794	1.59745
C	4.70767	-1.18009	2.12106
C	4.62028	1.18962	1.83947
C	5.36410	0.04414	2.09983
H	5.27209	-2.08243	2.33544
H	5.11655	2.15519	1.82666
C	-0.89750	-3.26172	0.54873
H	0.14011	-3.16601	0.22653
H	-1.08083	-2.51690	1.32637
H	-1.02281	-4.24620	0.99946
C	-3.20858	-0.66896	-3.25559
H	-3.19915	0.26471	-2.69256
H	-2.46469	-0.57662	-4.05029
H	-4.18982	-0.76182	-3.72230
C	-4.36749	-5.39952	-2.28869
H	-5.31797	-5.13011	-2.75165
H	-3.82585	-6.03576	-2.99564
H	-4.57175	-5.99956	-1.40038
C	6.84087	0.12757	2.33072
H	7.11334	1.05652	2.83470
H	7.19863	-0.70949	2.93210
H	7.37946	0.10243	1.37827
C	2.68402	-2.63194	1.90013
H	1.73043	-2.61120	2.43717
H	2.48187	-2.98094	0.88278
H	3.32192	-3.37130	2.38536
C	2.49304	2.39144	1.29795
H	2.16547	2.40896	0.25367
H	1.59854	2.48299	1.92290
H	3.10965	3.27403	1.46934
S	-0.17840	0.34647	-2.72063
Cl	-2.29805	0.81756	-0.15408

R = Mes*

P	-1.17730	0.65110	0.10483
C	-0.39219	-0.77719	0.36850
P	1.29803	-1.23122	0.56265
Cl	-1.92970	-2.89930	-1.48406
C	2.91092	-0.51969	0.08869
C	3.46375	-1.03161	-1.12526
C	3.79028	0.03188	1.06123
C	4.81476	-1.33919	-1.12718
C	5.13213	-0.32902	0.97923
C	5.65443	-1.09816	-0.04697
H	5.23335	-1.77329	-2.02629
H	5.79635	0.03249	1.75134
C	-2.95322	0.27810	0.15452
C	-3.71084	0.15027	-1.04169
C	-3.50735	-0.13499	1.39270
C	-4.87803	-0.60063	-0.98078
C	-4.66463	-0.90443	1.35267
C	-5.33626	-1.20525	0.18082
H	-5.44174	-0.74456	-1.88729
H	-5.06079	-1.27656	2.28872
C	-3.37812	0.86406	-2.36570
C	-3.05023	0.28658	2.80659
C	-6.54699	-2.13117	0.20840
C	3.48157	1.15912	2.07058
C	2.77009	-1.08045	-2.50281
C	7.09823	-1.58160	-0.07207
C	-4.25649	0.96222	3.48729
H	-3.96732	1.29572	4.48733
H	-4.58614	1.83593	2.91965
H	-5.10905	0.29236	3.59713
C	-1.93061	1.32295	2.85437
H	-0.95436	0.90980	2.59961
H	-2.13509	2.18600	2.21457
H	-1.84277	1.69934	3.87732
C	-2.62535	-0.92836	3.63573
H	-3.42650	-1.66531	3.71595
H	-1.76207	-1.42932	3.19370
H	-2.35952	-0.61102	4.64805
C	-7.63079	-1.54131	1.11822
H	-8.49923	-2.20519	1.14501
H	-7.27806	-1.41593	2.14390
H	-7.96024	-0.56505	0.75394
C	-7.14773	-2.34279	-1.17873
H	-7.99187	-3.03265	-1.10685
H	-7.52236	-1.41046	-1.60925

H	-6.42318	-2.77753	-1.87149
C	-6.10653	-3.49475	0.75528
H	-5.32923	-3.93468	0.12631
H	-5.70838	-3.41475	1.76883
H	-6.95775	-4.18073	0.78221
C	-4.40873	0.56355	-3.45866
H	-5.41696	0.87496	-3.17693
H	-4.13421	1.12520	-4.35416
H	-4.42833	-0.49525	-3.72780
C	-3.43230	2.37765	-2.11689
H	-4.42453	2.67454	-1.76814
H	-2.70484	2.70466	-1.37075
H	-3.21780	2.91891	-3.04211
C	-2.02211	0.46599	-2.95041
H	-1.17829	0.76710	-2.32691
H	-1.95738	-0.61160	-3.10404
H	-1.88099	0.96965	-3.91087
C	2.05152	1.68124	2.04252
H	1.73699	1.89720	1.01884
H	1.33105	1.01233	2.50465
H	2.00567	2.62640	2.58798
C	4.34919	2.35113	1.60626
H	4.15921	3.20543	2.26091
H	5.41605	2.12933	1.64155
H	4.09352	2.64751	0.58654
C	3.87943	0.80804	3.50664
H	4.93581	0.54499	3.58749
H	3.70938	1.67585	4.14913
H	3.29382	-0.02265	3.89898
C	1.32456	-0.60036	-2.51796
H	1.01602	-0.45774	-3.55653
H	0.62735	-1.31392	-2.08232
H	1.21994	0.36571	-2.01626
C	2.84704	-2.46026	-3.16024
H	2.27967	-3.19968	-2.59528
H	2.42445	-2.40478	-4.16684
H	3.87503	-2.81529	-3.25528
C	3.54974	-0.07380	-3.37634
H	3.51537	0.92913	-2.94417
H	4.59631	-0.35313	-3.50201
H	3.09089	-0.03152	-4.36759
C	7.84653	-1.23572	1.21241
H	7.94051	-0.15601	1.35369
H	7.35638	-1.65830	2.09304
H	8.85751	-1.64698	1.16619
C	7.83744	-0.93876	-1.25187
H	7.38242	-1.20041	-2.20915
H	7.84022	0.15058	-1.16681
H	8.87555	-1.28116	-1.27299
C	7.10266	-3.10618	-0.23860
H	6.59043	-3.59282	0.59477
H	6.60690	-3.41401	-1.16132
H	8.13091	-3.47634	-0.27028
Au	-0.28515	2.63797	-0.35684
Cl	0.64063	4.65413	-0.84272
Si	-1.13959	-2.64134	0.49817
S	1.13900	-3.11493	-0.08331
Cl	1.44277	-1.45281	2.60361

Coordination through π(C=P) bond

R= H

P	0.62478	0.20211	1.07577
C	0.32360	-0.00516	-0.61658
P	-1.18927	-0.30757	-1.43198
Cl	1.20843	-2.67972	-1.89312
Si	1.43760	-0.56971	-2.18437
H	2.02641	-0.07338	1.02576
H	-1.58394	-1.62044	-1.12916
Cl	-2.82442	0.72047	-0.84345
Au	0.72122	2.09097	-0.35678
Cl	1.09077	4.28850	-0.61972
S	-0.64646	-0.07591	-3.32050

R = Me

P	0.65747	0.24799	0.96127
C	0.31385	0.01064	-0.71847
P	-1.22496	-0.37669	-1.45442
Cl	1.55466	-2.61407	-1.88312
Si	1.41916	-0.50519	-2.29587
Cl	-2.76420	0.87892	-1.02943
Au	0.65453	2.12736	-0.50301
Cl	0.98583	4.33901	-0.73611
C	2.43888	-0.16541	1.07937
H	2.88671	0.41705	1.88640
H	2.50694	-1.22556	1.34448
H	2.98875	-0.00227	0.15104
C	-1.82657	-1.96403	-0.87420
H	-2.07703	-1.89081	0.18551
H	-2.71152	-2.23404	-1.45196
H	-1.03288	-2.69644	-1.03500
S	-0.70500	-0.36784	-3.37627

R = *t*-Bu

P	0.59284	0.29477	0.85546
C	0.35940	0.11124	-0.85804
P	-1.20848	-0.30187	-1.56500
Cl	2.17501	-1.94761	-2.55162
Si	1.41043	0.05713	-2.54838
Au	0.55266	2.24596	-0.50567
Cl	0.81228	4.48393	-0.50337
C	2.39210	0.00730	1.26868
C	2.50033	-1.50828	1.47240
C	3.41440	0.48372	0.25084
C	2.60334	0.72860	2.60215
H	1.75055	-1.88463	2.17544
H	2.39858	-2.04605	0.52736
H	3.48463	-1.74226	1.89085
H	3.34410	1.55880	0.07338
H	4.42111	0.27337	0.62842
H	3.32185	-0.03560	-0.70536
H	3.60519	0.50477	2.98032
H	2.52099	1.81254	2.49005
H	1.88083	0.40913	3.35817
C	-1.93919	-1.91181	-1.00020
C	-3.13634	-2.20735	-1.90326
C	-0.87847	-2.99700	-1.16684
C	-2.37886	-1.81375	0.45772
H	-3.92314	-1.45825	-1.80105
H	-2.84441	-2.27131	-2.95318
H	-3.55227	-3.17502	-1.60989
H	-0.01088	-2.82819	-0.52754
H	-1.32647	-3.95366	-0.88397
H	-0.52880	-3.07515	-2.19661
H	-2.75876	-2.79283	0.76289
H	-1.55260	-1.55588	1.12329
H	-3.17593	-1.08350	0.59898
Cl	-2.67266	1.04881	-1.13506
S	-0.75076	-0.27322	-3.50700

R = Ph

P	0.65420	0.20093	1.06346
C	0.20028	0.20575	-0.60991
P	-1.44178	-0.00264	-1.18708
Cl	1.20626	-1.83595	-2.89086
Si	1.03859	0.25744	-2.42400
Au	0.58511	2.28150	-0.11246
Cl	0.94636	4.50201	-0.00417
C	2.42582	-0.14724	1.11642
C	3.09593	0.30919	2.25635
C	3.12831	-0.87148	0.15175
C	4.45207	0.08123	2.40760
H	2.55149	0.85858	3.01823
C	4.48332	-1.10807	0.31544
H	2.61490	-1.27903	-0.71075
C	5.14668	-0.62640	1.43543
H	4.96798	0.45204	3.28549
H	5.02252	-1.67258	-0.43633
H	6.20810	-0.81061	1.55600
C	-1.98912	-1.70607	-1.01936
C	-2.21783	-2.48187	-2.14925
C	-2.16975	-2.24105	0.25580
C	-2.63156	-3.79706	-2.00159
H	-2.06344	-2.05883	-3.13417
C	-2.57784	-3.55530	0.39277
H	-1.99808	-1.63334	1.13773
C	-2.80974	-4.33218	-0.73578
H	-2.80962	-4.40423	-2.88105
H	-2.71821	-3.97460	1.38198
H	-3.13104	-5.36144	-0.62440
Cl	-2.83696	1.04495	-0.14080
S	-1.24551	0.61806	-3.06674

R = Mes

P	0.61996	0.13967	1.60858
C	0.01612	0.27490	-0.01679
P	-1.56650	0.06414	-0.77317
Cl	1.30835	-1.13767	-2.63743
Si	1.02199	0.77305	-1.67954
Au	0.06481	2.30113	0.76730
Cl	-0.04647	4.52170	1.17234
C	2.41433	-0.10067	1.37280
C	3.37417	0.84677	1.76344
C	2.81681	-1.38401	0.95313
C	4.71922	0.52503	1.62579
C	4.17250	-1.65459	0.84008
C	5.14115	-0.70698	1.14663
H	5.46058	1.26374	1.91543
H	4.47956	-2.64438	0.51559
C	-1.99289	-1.67696	-1.03923
C	-2.16649	-2.23788	-2.32711
C	-2.13112	-2.49555	0.10537
C	-2.37517	-3.60826	-2.41983
C	-2.32724	-3.85955	-0.06380
C	-2.43115	-4.44375	-1.31502
H	-2.50299	-4.03557	-3.40905
H	-2.41890	-4.47900	0.82292
C	3.01097	2.18560	2.32795
H	2.73724	2.89822	1.54430
H	2.16344	2.12767	3.01611
H	3.85475	2.60905	2.87468
C	1.82934	-2.48049	0.67961
H	1.09433	-2.57163	1.48667
H	1.28128	-2.31465	-0.25026
H	2.34281	-3.43899	0.59488
C	6.59645	-1.00972	0.96963
H	7.21385	-0.40721	1.63788
H	6.81071	-2.06352	1.15742
H	6.91155	-0.79066	-0.05543
C	-2.17860	-1.99459	1.51820
H	-1.89357	-2.79143	2.20625
H	-1.53520	-1.14126	1.72284
H	-3.19781	-1.68738	1.76884
C	-2.18451	-1.48192	-3.62288
H	-2.87160	-0.63624	-3.59960
H	-1.19868	-1.09407	-3.88017
H	-2.50344	-2.15356	-4.42045
C	-2.60162	-5.92121	-1.47195
H	-1.63127	-6.40137	-1.63258
H	-3.04720	-6.36928	-0.58263
H	-3.22848	-6.16030	-2.33277
S	-1.21906	1.24634	-2.34899
Cl	-3.16512	0.82581	0.25742

R = Mes*

P	0.68880	0.23202	0.99258
C	0.43514	0.13511	-0.74565
P	-1.13396	-0.09323	-1.62948
Cl	2.78080	-0.81485	-3.02666
Au	0.53459	2.28821	-0.33601
Cl	0.42489	4.55150	-0.10084
C	2.52074	0.06628	1.16768
C	3.20164	-1.10285	0.72790
C	3.29828	1.16944	1.60675
C	4.53937	-0.98897	0.38428
C	4.63456	1.21630	1.20655
C	5.25845	0.19268	0.51712
H	5.04964	-1.86787	0.01140
H	5.20917	2.09197	1.47253
C	2.90202	2.23225	2.65494
C	2.66916	-2.54474	0.85189
C	6.68734	0.28736	-0.00389
C	3.78035	1.92566	3.88615
H	3.58496	0.91960	4.26677
H	4.84453	1.99774	3.65847
H	3.55437	2.64177	4.68105
C	3.20302	3.65841	2.18594
H	2.61887	3.92774	1.30606
H	2.94533	4.36286	2.98158
H	4.25982	3.80278	1.95568
C	1.46162	2.18151	3.15611
H	1.37156	2.84789	4.01841
H	0.73858	2.52898	2.41788
H	1.17784	1.18043	3.49161
C	2.95813	-3.38724	-0.39283
H	2.51990	-2.94239	-1.28447
H	4.02804	-3.50439	-0.57116
H	2.54228	-4.39022	-0.26111
C	3.42753	-3.15974	2.04424
H	4.50545	-3.16160	1.87631
H	3.23204	-2.60083	2.96291
H	3.10335	-4.19369	2.19618
C	1.18756	-2.67388	1.18368
H	0.91544	-2.11101	2.08206
H	0.54625	-2.36079	0.36131
H	0.96030	-3.72411	1.38868
C	7.30248	1.66208	0.24334
H	6.71616	2.45713	-0.22366
H	7.39568	1.88293	1.30992
H	8.30667	1.69426	-0.18617
C	6.67565	0.02814	-1.51525
H	7.69300	0.08052	-1.91342
H	6.26966	-0.95702	-1.75303

H	6.06487	0.76955	-2.03520
C	7.56468	-0.76423	0.68529
H	7.58456	-0.61021	1.76715
H	7.20868	-1.77894	0.49595
H	8.59090	-0.69849	0.31315
C	-2.36610	-1.47588	-1.68097
C	-2.35456	-2.31940	-2.83401
C	-3.58471	-1.35273	-0.95794
C	-3.57404	-2.59384	-3.43360
C	-1.16455	-3.15623	-3.34214
C	-4.76352	-1.69305	-1.61323
C	-3.73095	-1.07284	0.54926
C	-4.79576	-2.20608	-2.89943
H	-3.56384	-3.17285	-4.34863
C	-0.80203	-2.89444	-4.80534
C	-1.61395	-4.62955	-3.22516
C	0.06428	-3.04332	-2.45616
H	-5.69366	-1.54787	-1.08227
C	-2.42693	-0.75183	1.25844
C	-4.22785	-2.40526	1.14922
C	-4.75800	0.01503	0.87375
C	-6.08692	-2.44399	-3.67022
H	-0.06723	-3.63426	-5.13349
H	-1.67402	-2.98274	-5.45727
H	-0.37080	-1.90519	-4.94418
H	-2.42087	-4.87958	-3.91463
H	-0.76421	-5.27645	-3.45903
H	-1.94889	-4.86131	-2.21128
H	0.51200	-2.05273	-2.45404
H	-0.17274	-3.33590	-1.43124
H	0.83583	-3.72373	-2.82338
H	-1.65260	-1.48641	1.02705
H	-2.05366	0.24511	1.03296
H	-2.58282	-0.78066	2.33961
H	-4.32586	-2.29736	2.23283
H	-5.19820	-2.70069	0.74841
H	-3.51919	-3.21283	0.94987
H	-5.74762	-0.21541	0.47600
H	-4.85633	0.10369	1.95874
H	-4.45269	0.98581	0.48432
C	-7.31225	-1.93548	-2.91555
C	-6.26633	-3.94453	-3.93071
C	-6.00038	-1.70223	-5.00973
H	-7.46925	-2.47566	-1.97839
H	-7.23574	-0.86874	-2.69112
H	-8.20414	-2.08221	-3.52899
H	-5.44692	-4.35628	-4.52305
H	-6.31794	-4.50249	-2.99256
H	-7.19444	-4.11907	-4.48158
H	-5.88389	-0.62696	-4.85549
H	-5.15574	-2.04753	-5.60920
H	-6.91299	-1.86708	-5.58871
Cl	-2.41976	1.46133	-1.23611
Si	1.53188	0.75819	-2.29431
S	-0.44371	0.29573	-3.47393

[R-P=C(Si(II)Cl)-P(S)MeR](AuCl)

Coordination through Si(II) atom

R= H

P	0.75394	0.26943	0.84892
C	0.42809	-0.15575	-0.72338
P	-1.15679	-0.24132	-1.48835
Cl	1.60496	-2.37095	-2.55308
Si	1.47617	-0.30348	-2.32355
H	2.17646	0.30539	0.72701
H	-1.69274	-1.53930	-1.35932
Au	3.21606	1.04096	-2.75330
Cl	4.98518	2.45186	-3.11433
C	-2.43698	0.83975	-0.84582
H	-3.34328	0.72267	-1.44298
H	-2.64680	0.57218	0.19257
H	-2.08989	1.87190	-0.90190
S	-0.55546	0.15121	-3.36741

R = Me

P	0.71269	0.13905	0.83425
C	0.37516	-0.15845	-0.76542
P	-1.24249	-0.25286	-1.45573
Cl	1.70538	-2.28495	-2.63123
Si	1.35038	-0.23471	-2.40736
C	-2.40415	0.98323	-0.85725
H	-3.34394	0.89898	-1.40652
H	-2.58758	0.82583	0.20808
H	-1.97692	1.97351	-1.01448
C	2.53694	0.16553	0.91129
H	2.98236	0.55899	-0.00637
H	2.85284	0.76295	1.76774
H	2.89350	-0.85827	1.06329
C	-2.00235	-1.86199	-1.18580
H	-2.21660	-1.99284	-0.12262
H	-2.92589	-1.93459	-1.76346
H	-1.30223	-2.62909	-1.51860
Au	2.96870	1.24088	-2.91477
Cl	4.62093	2.77889	-3.33527
S	-0.70877	0.03298	-3.38904

R = *t*-Bu

P	0.71338	0.00488	0.64588
C	0.53946	-0.40414	-0.95873
P	-1.08102	-0.48123	-1.68335
Cl	2.14366	-2.55152	-2.69361
Si	1.54595	-0.55347	-2.58531
C	-2.03553	1.01718	-1.37023
H	-2.98137	0.98737	-1.91271
H	-2.22028	1.12631	-0.30040
H	-1.44439	1.86211	-1.72433
Au	2.96588	1.07578	-3.22282
Cl	4.38710	2.79524	-3.78342
C	2.51703	0.15142	1.08230
C	3.44782	-0.77196	0.31281
C	2.87788	1.62168	0.83692
C	2.58792	-0.15427	2.58127
H	3.14121	-1.81782	0.37906
H	3.51611	-0.49596	-0.74140
H	4.45806	-0.68999	0.72738
H	2.20012	2.30451	1.35850
H	3.88713	1.80824	1.21828
H	2.87113	1.86729	-0.22729
H	3.60564	0.02717	2.93902
H	1.91126	0.48007	3.16038
H	2.34049	-1.19771	2.79458
C	-2.10891	-1.93715	-1.18838
C	-3.30476	-2.01590	-2.13729
C	-1.25712	-3.19715	-1.31584
C	-2.57640	-1.75442	0.25481
H	-3.96336	-1.14873	-2.05253
H	-2.98826	-2.11625	-3.17770
H	-3.89464	-2.90040	-1.88166
H	-0.40071	-3.17944	-0.64012
H	-1.87524	-4.06239	-1.06104

H	-0.88262	-3.33288	-2.33160
H	-3.11417	-2.65543	0.56281
H	-1.73985	-1.61718	0.94435
H	-3.25989	-0.91034	0.36582
S	-0.48940	-0.57730	-3.62445

R = Ph

P	0.86727	-0.35721	0.42567
C	0.49725	-0.27810	-1.19941
P	-1.19745	-0.36990	-1.70006
Cl	2.12119	-2.15790	-3.11757
Si	1.27385	-0.25724	-2.94558
C	-2.10420	1.14313	-1.33987
H	-3.09619	1.10854	-1.79343
H	-2.18405	1.28784	-0.26074
H	-1.53781	1.96837	-1.77301
Au	2.38156	1.52008	-3.77716
Cl	3.50805	3.36695	-4.55804
C	-2.11733	-1.74963	-1.00400
C	-1.65133	-3.03886	-1.26433
C	-3.25000	-1.56395	-0.21599
C	-2.31631	-4.13002	-0.73563
H	-0.77175	-3.18220	-1.88356
C	-3.91249	-2.66341	0.30923
H	-3.62377	-0.56922	-0.00656
C	-3.44659	-3.94283	0.05040
H	-1.95413	-5.13061	-0.93963
H	-4.79484	-2.51738	0.92099
H	-3.96705	-4.80034	0.46108
C	2.64533	-0.25343	0.63419
C	3.09457	-0.61526	1.91071
C	3.57374	0.18070	-0.31725
C	4.44355	-0.57528	2.21932
H	2.37597	-0.93293	2.66016
C	4.91771	0.23367	-0.00044
H	3.25028	0.50458	-1.30121
C	5.35424	-0.15091	1.26272
H	4.78352	-0.86712	3.20602
H	5.62796	0.58014	-0.74189
H	6.41070	-0.11040	1.50279
S	-0.86012	-0.57445	-3.69075

R = Mes

P	0.83983	0.63401	0.28743
C	0.54026	-0.49847	-0.89992
P	-0.96828	-0.71549	-1.82393
Cl	1.58119	-3.40989	-1.52984
Si	1.73209	-1.39065	-2.09476
C	-1.92013	0.79205	-2.11404
H	-2.69043	0.56676	-2.85477
H	-2.38985	1.15983	-1.20417
H	-1.23988	1.54436	-2.51406
Au	3.74558	-0.60729	-2.72558
Cl	5.80204	0.26604	-3.30185
C	-2.10053	-1.97108	-1.14049
C	-2.40870	-3.18915	-1.79149
C	-2.67386	-1.69727	0.12086
C	-3.24216	-4.09123	-1.14142
C	-3.49050	-2.64839	0.71903
C	-3.78251	-3.85810	0.11396
H	-3.47402	-5.02287	-1.64735
H	-3.91305	-2.42366	1.69357
C	2.61580	0.48670	0.61607
C	3.12392	-0.57586	1.37789
C	3.46102	1.51063	0.15987
C	4.48987	-0.61277	1.62569
C	4.81778	1.42443	0.42686
C	5.35489	0.36157	1.14296
H	4.89138	-1.43458	2.21113
H	5.47810	2.19247	0.03698
C	2.23998	-1.65499	1.93120
H	1.33779	-1.24333	2.39457
H	1.91439	-2.34973	1.15240
H	2.77020	-2.23136	2.69042
C	2.93207	2.64323	-0.66592
H	2.79766	2.32935	-1.70609
H	1.96274	3.00052	-0.29925
H	3.62376	3.48628	-0.66417
C	6.83228	0.26210	1.35716
H	7.32709	-0.01580	0.42175
H	7.25385	1.21851	1.67472
H	7.07957	-0.49053	2.10711
C	-2.51339	-0.40892	0.87496
H	-1.56368	0.09839	0.71109
H	-3.31483	0.29021	0.61551
H	-2.58960	-0.59426	1.94711
C	-1.92579	-3.60805	-3.14981
H	-2.14703	-2.86963	-3.92050
H	-0.84843	-3.77566	-3.16352
H	-2.41647	-4.54026	-3.43052
C	-4.63428	-4.88422	0.79132
H	-5.26077	-5.41716	0.07378
H	-4.00855	-5.62909	1.29258
H	-5.27919	-4.43336	1.54685
S	0.00675	-1.14478	-3.56128

R = Mes*

P	-0.39879	1.43168	1.47185
C	0.16549	0.39367	0.28887
P	1.78787	-0.33580	0.10515
Cl	-1.22012	0.84146	-2.61418
C	3.48608	0.35175	0.22170
C	4.13110	0.75887	-0.98218
C	4.30126	-0.02252	1.32383
C	5.45051	0.36963	-1.16320
C	5.62050	-0.38023	1.06456
C	6.19180	-0.29699	-0.19580
H	5.92639	0.62116	-2.10286
H	6.22589	-0.72042	1.89350
C	-2.19596	1.49109	1.12443
C	-2.73436	2.54339	0.35071
C	-3.03418	0.42966	1.55811
C	-3.99215	2.35141	-0.21640
C	-4.26764	0.30023	0.93468
C	-4.73871	1.20249	-0.01141
H	-4.39459	3.12159	-0.85473
H	-4.88721	-0.54668	1.18828
C	-2.06083	3.92402	0.21707
C	-2.75581	-0.42440	2.81586
C	-6.04961	0.92395	-0.73786
C	3.93358	0.13831	2.81331
C	3.58480	1.77531	-2.00444
C	7.59455	-0.80051	-0.50945
C	-3.86438	-1.45188	3.06229
H	-3.63881	-1.99362	3.98379
H	-4.83961	-0.97961	3.19737
H	-3.93219	-2.18519	2.25433
C	-2.74656	0.54510	4.00885
H	-1.93781	1.27709	3.92760
H	-3.68932	1.09312	4.07526
H	-2.60611	-0.00999	4.94104
C	-1.43966	-1.20488	2.82295

H	-1.32122	-1.80015	1.91544
H	-0.56803	-0.55474	2.93346
H	-1.43498	-1.88168	3.68174
C	-7.18656	0.75613	0.27693
H	-8.12640	0.55736	-0.24547
H	-7.00550	-0.07805	0.95706
H	-7.31516	1.66112	0.87665
C	-6.42937	2.04791	-1.69818
H	-7.35991	1.78926	-2.20905
H	-6.59262	2.99525	-1.17682
H	-5.66493	2.20152	-2.46393
C	-5.89196	-0.37130	-1.54480
H	-5.09684	-0.27262	-2.28772
H	-5.64278	-1.22422	-0.91050
H	-6.82382	-0.60103	-2.06956
C	-2.94329	4.91906	-0.54049
H	-3.91923	5.04776	-0.06768
H	-2.44840	5.89307	-0.54546
H	-3.09630	4.62373	-1.58117
C	-1.85335	4.49327	1.62818
H	-2.80304	4.56210	2.16373
H	-1.17604	3.87683	2.22461
H	-1.41972	5.49575	1.56804
C	-0.71810	3.88907	-0.51236
H	0.04947	3.35575	0.05388
H	-0.80197	3.40988	-1.48827
H	-0.35081	4.91024	-0.65182
C	2.55486	0.74073	3.05473
H	2.42215	1.66749	2.49301
H	1.72389	0.08047	2.81553
H	2.44853	0.98572	4.11419
C	4.92603	1.18303	3.36825
H	4.68319	1.39003	4.41394
H	5.95911	0.83776	3.32566
H	4.85849	2.12022	2.81113
C	4.10953	-1.14808	3.62544
H	5.12939	-1.53077	3.55491
H	3.90827	-0.94544	4.68048
H	3.43836	-1.94513	3.30590
C	2.21486	2.33959	-1.65267
H	2.00429	3.19178	-2.30337
H	1.40455	1.63015	-1.80127
H	2.19022	2.70104	-0.62195
C	3.57704	1.24530	-3.43981
H	2.86856	0.42683	-3.55930
H	3.28544	2.04870	-4.12127
H	4.56360	0.89376	-3.74932
C	4.55519	2.97453	-1.93715
H	4.60848	3.38078	-0.92400
H	5.56668	2.71396	-2.25051
H	4.19147	3.76340	-2.60065
C	8.23030	-1.51706	0.67882
H	8.38297	-0.84615	1.52813
H	7.62617	-2.36478	1.01177
H	9.21035	-1.90276	0.38920
C	8.49280	0.37922	-0.90042
H	8.12069	0.89887	-1.78547
H	8.56277	1.10579	-0.08723
H	9.50137	0.02203	-1.12495
C	7.51202	-1.78645	-1.68120
H	6.88828	-2.64676	-1.42703
H	7.09057	-1.32001	-2.57377
H	8.51057	-2.15286	-1.93373
Au	-2.09797	-2.25040	-0.76565
Cl	-3.61603	-3.91790	-0.23800
Si	-0.66055	-0.55172	-1.16083
S	1.45909	-1.06813	-1.78023
C	1.80225	-1.86651	1.08160
H	1.79069	-1.71186	2.15541
H	0.90147	-2.40794	0.78595
H	2.67876	-2.44745	0.79155

Coordination through S atom

R= H

P	0.61172	-0.16158	1.12559
C	0.43974	-0.32679	-0.51791
P	-0.96904	0.08908	-1.46909
Cl	0.90659	-2.58053	-2.51584
Si	1.73549	-0.71057	-1.94948
H	1.97889	-0.58045	1.20694
H	-1.62672	-1.10303	-1.81135
Au	-1.39606	0.33184	-4.79958
Cl	-2.85174	-0.10042	-6.46251
C	-2.23058	1.16533	-0.78383
H	-2.99595	1.33385	-1.54469
H	-2.67897	0.68475	0.08915
H	-1.78106	2.11596	-0.49615
S	0.02507	0.82824	-3.08756

R = Me

P	0.70767	0.09620	0.95232
C	0.45656	-0.15080	-0.67239
P	-1.09522	0.02739	-1.46144
Cl	1.37777	-2.48362	-2.49044
Si	1.66306	-0.39447	-2.19848
C	-2.20282	1.28861	-0.81319
H	-3.08691	1.34802	-1.45168
H	-2.50162	1.02529	0.20420
H	-1.69014	2.25032	-0.80777
C	2.48342	-0.29545	1.18196
H	3.07486	-0.07467	0.28933
H	2.87286	0.26291	2.03474
H	2.58013	-1.36213	1.40594
C	-2.00081	-1.52253	-1.46444
H	-2.28316	-1.76075	-0.43572
H	-2.88890	-1.42631	-2.09171
H	-1.35199	-2.30299	-1.86258
Au	-1.71255	-0.51879	-4.83675
Cl	-3.08971	-1.49770	-6.32882
S	-0.37090	0.52447	-3.31827

R = *t*-Bu

P	0.70659	-0.15837	0.61335
C	0.40685	-0.31008	-1.02068
P	-1.26098	-0.48156	-1.57153
Cl	2.06645	-2.10855	-2.95572
Si	1.35618	-0.09972	-2.72846
C	-2.31670	0.88154	-1.05096
H	-3.30333	0.80367	-1.50819
H	-2.39629	0.86780	0.03830
H	-1.84994	1.81297	-1.37182
Au	-1.73505	1.49479	-4.40411
Cl	-2.57998	3.42437	-5.20925
C	2.55070	-0.14354	0.90511
C	2.96685	-1.61034	1.05576
C	3.36408	0.53578	-0.18395
C	2.74276	0.58720	2.23638
H	2.38004	-2.12549	1.82226
H	2.86107	-2.15310	0.11434
H	4.01752	-1.65677	1.36240

H	3.01710	1.55073	-0.38898
H	4.41209	0.59801	0.12953
H	3.35258	-0.02869	-1.12019
H	3.79498	0.53937	2.53218
H	2.46348	1.64161	2.16306
H	2.14869	0.13670	3.03611
C	-2.08385	-2.07744	-1.11518
C	-3.38014	-2.18847	-1.91847
C	-1.14646	-3.22983	-1.46632
C	-2.38559	-2.07537	0.38238
H	-4.09608	-1.40352	-1.66625
H	-3.19639	-2.16176	-2.99411
H	-3.84937	-3.14751	-1.68264
H	-0.21166	-3.18073	-0.90631
H	-1.64642	-4.17000	-1.21769
H	-0.90178	-3.24823	-2.52959
H	-2.80448	-3.04953	0.64971
H	-1.48715	-1.92410	0.98514
H	-3.12039	-1.31560	0.65587
S	-0.92045	-0.48927	-3.61436

R = Ph

P	0.84288	-0.25088	1.09478
C	0.40494	-0.20947	-0.51674
P	-1.26707	-0.05443	-1.00761
Cl	1.34870	-1.65127	-3.15591
Si	1.36779	0.23525	-2.16243
C	-2.35213	0.85099	0.11014
H	-3.33577	0.95986	-0.35022
H	-2.44851	0.29939	1.04768
H	-1.92609	1.83709	0.29580
Au	-2.34221	0.35630	-4.35090
Cl	-3.81288	-0.25690	-5.95132
C	2.63401	-0.42175	1.08218
C	3.31229	0.07588	2.20049
C	3.36859	-1.00757	0.04562
C	4.69401	0.03038	2.26110
H	2.74795	0.51224	3.01896
C	4.75110	-1.05771	0.11371
H	2.85835	-1.47157	-0.79181
C	5.41338	-0.53284	1.21497
H	5.21210	0.42932	3.12538
H	5.31252	-1.51444	-0.69289
H	6.49545	-0.57507	1.26463
C	-2.03612	-1.65134	-1.29631
C	-3.35430	-1.70863	-1.75056
C	-1.32474	-2.82418	-1.06332
C	-3.95352	-2.93619	-1.96612
H	-3.90952	-0.80243	-1.96514
C	-1.93513	-4.05030	-1.27735
H	-0.29641	-2.77731	-0.72928
C	-3.24457	-4.10633	-1.72794
H	-4.97075	-2.97757	-2.33590
H	-1.37868	-4.96323	-1.10170
H	-3.71523	-5.06660	-1.90448
S	-0.92046	1.05199	-2.71369

R = Mes

P	0.89034	-0.70491	1.04260
C	0.37810	-0.89952	-0.53171
P	-1.32117	-0.87710	-0.99310
Cl	1.30544	-2.21964	-3.20246
Si	1.33153	-0.33670	-2.15055
C	-2.35374	-0.08950	0.25888
H	-3.36786	0.05809	-0.10730
H	-2.37420	-0.75081	1.12941
H	-1.91438	0.87121	0.52877
Au	-0.92406	2.39295	-1.79154
Cl	-0.81279	4.46366	-0.89521
C	2.67391	-0.36803	0.87963
C	3.10767	0.95094	1.09211
C	3.60219	-1.39642	0.66457
C	4.46833	1.22003	1.05465
C	4.95411	-1.07839	0.64196
C	5.40756	0.22192	0.82498
H	4.80304	2.24132	1.21102
H	5.67535	-1.87463	0.48212
C	-2.15115	-2.42066	-1.46565
C	-3.39560	-2.32395	-2.12603
C	-1.61102	-3.69204	-1.17385
C	-4.03288	-3.49169	-2.52712
C	-2.30473	-4.81912	-1.59195
C	-3.50507	-4.74845	-2.28386
H	-4.98199	-3.40541	-3.04712
H	-1.88138	-5.79275	-1.36696
C	2.12890	2.06266	1.31818
H	1.62247	2.34810	0.39072
H	1.34850	1.77885	2.03336
H	2.62588	2.95255	1.70579
C	6.86863	0.54390	0.75942
H	7.14869	0.85575	-0.25166
H	7.12723	1.36146	1.43491
H	7.48043	-0.32243	1.01643
C	3.17487	-2.81919	0.45845
H	2.36505	-3.10491	1.13639
H	2.81553	-2.98116	-0.56228
H	4.00906	-3.50159	0.62723
C	-0.33829	-3.91944	-0.41757
H	0.51652	-3.48449	-0.93495
H	-0.37460	-3.48556	0.58365
H	-0.16202	-4.98998	-0.31121
C	-4.13862	-1.04474	-2.39372
H	-3.50030	-0.17358	-2.52734
H	-4.73644	-1.14769	-3.30005
H	-4.83513	-0.83283	-1.57604
C	-4.19346	-5.98693	-2.76321
H	-3.77322	-6.31022	-3.72059
H	-4.06683	-6.81063	-2.05833
H	-5.26097	-5.81899	-2.91230
S	-1.02581	0.29022	-2.69829

R = Mes*

P	-2.06030	1.75853	0.57315
C	-1.09406	0.42897	0.29009
P	0.67314	0.33056	0.39738
C	-3.75441	1.04291	0.47188
C	-4.54078	1.31130	-0.68466
C	-4.23532	0.13407	1.43888
C	-5.61718	0.48013	-0.94392
C	-5.31976	-0.67724	1.09271
C	-5.98620	-0.57228	-0.11046
H	-6.19501	0.64344	-1.84150
H	-5.65702	-1.40829	1.81391
C	-3.82323	0.04925	2.92525
C	-4.29647	2.51100	-1.62076
C	-7.11653	-1.50891	-0.52140
C	-3.00129	2.37081	-2.42240
H	-2.11400	2.40556	-1.78760
H	-2.97397	1.42675	-2.96948
H	-2.91725	3.19557	-3.13646
C	-4.26580	3.80361	-0.79328
H	-5.20317	3.93514	-0.24729

H	-3.44860	3.82015	-0.06952
H	-4.13283	4.66445	-1.45463
C	-5.42478	2.67747	-2.64265
H	-5.46769	1.84699	-3.35111
H	-6.40184	2.78166	-2.16516
H	-5.24034	3.58628	-3.22008
C	-3.45030	-1.38063	3.33082
H	-2.60472	-1.74923	2.74564
H	-3.17767	-1.40454	4.38971
H	-4.27608	-2.07964	3.19059
C	-2.68410	0.96632	3.35914
H	-1.71605	0.62450	2.99710
H	-2.83672	2.00254	3.03861
H	-2.63781	0.97758	4.45193
C	-5.05360	0.49403	3.73904
H	-5.32843	1.52399	3.49716
H	-5.92225	-0.13668	3.54844
H	-4.82741	0.44235	4.80780
C	-7.36718	-2.60246	0.51336
H	-7.68957	-2.19097	1.47351
H	-8.16067	-3.26441	0.15849
H	-6.47654	-3.21332	0.68020
C	-6.74411	-2.18034	-1.84917
H	-6.59796	-1.44738	-2.64505
H	-5.81913	-2.75318	-1.75216
H	-7.54054	-2.86138	-2.16208
C	-8.41206	-0.70922	-0.70177
H	-8.31027	0.06200	-1.46792
H	-9.22491	-1.37467	-1.00572
H	-8.70359	-0.22002	0.23109
C	2.04820	1.46762	0.00627
C	2.66563	1.34157	-1.27192
C	2.82118	1.98590	1.07994
C	4.05135	1.32421	-1.31067
C	4.20528	1.93903	0.96090
C	4.84931	1.49996	-0.18671
H	4.52972	1.16752	-2.26928
H	4.80007	2.27055	1.80114
C	1.95036	1.43708	-2.63339
C	2.29340	0.28640	-3.58209
C	2.47041	2.74972	-3.25847
H	1.92521	-0.66724	-3.20623
H	1.82919	0.46971	-4.55468
H	3.36903	0.19566	-3.74422
H	2.25028	3.60540	-2.61521
H	3.54649	2.72708	-3.43417
H	1.97437	2.90954	-4.21952
C	2.26936	2.78582	2.27717
C	2.87342	4.19952	2.13632
C	2.70556	2.22748	3.63482
H	2.61368	4.64090	1.17147
H	2.47327	4.84279	2.92477
H	3.95987	4.19561	2.22497
H	2.30598	1.23290	3.83131
H	3.79246	2.16351	3.71094
H	2.35922	2.89049	4.43198
C	6.35540	1.29899	-0.27209
C	6.95454	2.29649	-1.27081
C	6.62933	-0.13273	-0.75116
H	6.77033	3.32748	-0.95789
H	8.03572	2.14987	-1.33939
H	6.53921	2.16820	-2.27242
H	6.20382	-0.87308	-0.06980
H	6.20656	-0.31520	-1.74110
H	7.70754	-0.30300	-0.81253
Cl	-2.37101	-1.37097	-1.96906
Au	2.57597	-2.57140	-0.06295
Cl	4.40491	-3.70749	0.63074
C	0.75939	2.99762	2.24366
H	0.43913	3.42323	1.29058
H	0.16985	2.10048	2.42247
H	0.47937	3.70477	3.02834
C	0.43802	1.59579	-2.54388
H	-0.08298	0.68367	-2.25717
H	0.16638	2.39399	-1.84872
H	0.05381	1.87459	-3.52815
C	7.03961	1.49497	1.07809
H	6.93912	2.51981	1.44585
H	6.64301	0.81253	1.83373
H	8.10754	1.28963	0.97634
Si	-1.51734	-1.47568	-0.01788
S	0.81238	-1.34430	-0.81943
C	1.01524	-0.35953	2.04147
H	2.06891	-0.63305	2.10450
H	0.74473	0.31901	2.84529
H	0.40805	-1.26452	2.11683

Coordination through P(sp^2) atom

R= H

P	0.49306	0.10708	1.03936
C	0.31685	-0.28225	-0.54193
P	-1.11039	-0.20030	-1.55446
Cl	1.20250	-2.76520	-2.05920
Si	1.64254	-0.67043	-1.97413
H	1.86872	-0.06836	1.31235
H	-1.72236	-1.46801	-1.50395
Au	-0.95631	0.77862	2.55398
Cl	-2.49119	1.46545	4.05678
C	-2.40931	0.95117	-1.09730
H	-2.81950	0.68016	-0.12141
H	-1.99998	1.96105	-1.06029
H	-3.19684	0.90394	-1.85282
S	-0.21995	0.14492	-3.30733

R = Me

P	0.79305	0.03053	0.86530
C	0.38921	-0.20940	-0.70450
P	-1.21205	-0.22648	-1.42145
Cl	1.51496	-2.57681	-2.30030
Si	1.46124	-0.42694	-2.34691
C	-2.31682	1.09074	-0.89083
H	-3.23800	1.03288	-1.47506
H	-2.54777	0.98337	0.17169
H	-1.83654	2.05267	-1.06953
C	2.57839	-0.14655	1.08175
H	3.08897	-0.04177	0.12218
H	2.93478	0.60253	1.79003
H	2.78700	-1.13706	1.49432
C	-2.03039	-1.78287	-1.04347
H	-2.25071	-1.83228	0.02550
H	-2.95528	-1.85143	-1.61966
H	-1.35989	-2.59511	-1.32678
Au	-0.56224	0.44059	2.56067
Cl	-2.01295	0.84544	4.24706
S	-0.67817	-0.07784	-3.35289

R = *t*-Bu

P	0.79867	0.08241	0.73968
C	0.52525	-0.48270	-0.78626
P	-1.06151	-0.56082	-1.56353

Cl	2.06547	-2.85740	-2.25613
Si	1.65429	-0.75640	-2.39338
C	-2.02569	0.95350	-1.38102
H	-2.89403	0.90814	-2.04005
H	-2.34526	1.09998	-0.34783
H	-1.39264	1.78700	-1.68623
Au	-0.71871	0.95559	2.10525
Cl	-2.31486	1.87052	3.42995
C	2.58342	0.03355	1.24221
C	2.66191	0.48975	2.69727
C	3.12067	-1.38995	1.10879
C	3.36021	1.00200	0.34777
H	2.27793	1.50358	2.83126
H	2.10616	-0.17309	3.36452
H	3.70971	0.48199	3.01081
H	3.07681	-1.75886	0.08387
H	4.16719	-1.40048	1.42932
H	2.57076	-2.08903	1.74368
H	4.41145	0.99494	0.65255
H	3.31496	0.71858	-0.70692
H	2.99126	2.02583	0.44617
C	-2.11215	-1.98478	-1.00714
C	-1.31340	-3.27559	-1.15583
C	-2.53887	-1.79498	0.44543
C	-3.33765	-2.02103	-1.92229
H	-0.99026	-3.43745	-2.18478
H	-0.42577	-3.28209	-0.52162
H	-1.95171	-4.11263	-0.85920
H	-3.12644	-0.88888	0.60374
H	-3.16463	-2.64477	0.73320
H	-1.68479	-1.77472	1.12470
H	-3.92380	-2.91077	-1.67689
H	-3.98649	-1.15385	-1.78347
H	-3.05555	-2.08854	-2.97543
S	-0.45106	-0.78797	-3.47475

R = Ph

P	0.77923	-0.16379	0.87286
C	0.40685	-0.13949	-0.73132
P	-1.23761	-0.12531	-1.36905
Cl	1.82974	-1.85524	-2.95145
Si	1.38653	0.18155	-2.40226
C	-2.32287	1.09874	-0.61638
H	-3.27619	1.09028	-1.14856
H	-2.48695	0.89067	0.44245
H	-1.85183	2.07603	-0.72465
Au	-0.66435	-0.05733	2.55257
Cl	-2.23475	0.03110	4.18381
C	-2.03241	-1.73310	-1.19466
C	-2.76860	-2.03564	-0.04873
C	-1.86646	-2.69063	-2.19157
C	-3.33912	-3.29010	0.09005
H	-2.89803	-1.30824	0.74438
C	-2.43995	-3.94310	-2.04351
H	-1.28826	-2.45078	-3.07611
C	-3.17589	-4.24228	-0.90633
H	-3.91109	-3.52122	0.98081
H	-2.30985	-4.68700	-2.82058
H	-3.62446	-5.22275	-0.79428
C	2.52812	-0.25754	1.18376
C	3.44327	-0.71311	0.23029
C	2.98015	0.16391	2.43825
C	4.79498	-0.71876	0.52768
H	3.10091	-1.10109	-0.72157
C	4.33319	0.15646	2.72423
H	2.26599	0.50157	3.18235
C	5.24023	-0.28042	1.76758
H	5.50274	-1.07619	-0.21078
H	4.68104	0.48859	3.69513
H	6.30027	-0.29003	1.99422
S	-0.81916	0.33762	-3.28112

R = Mes

P	0.85623	-0.21373	1.36826
C	0.51656	-0.55655	-0.20312
P	-1.01877	-0.42720	-1.07192
Cl	1.70914	-2.53303	-2.22995
Si	1.81014	-0.43965	-1.69088
C	-2.14796	0.71344	-0.24251
H	-3.05460	0.85020	-0.83100
H	-2.41850	0.29389	0.72998
H	-1.64687	1.67142	-0.10179
Au	-0.53979	0.07428	3.06290
Cl	-1.98971	0.35244	4.78044
C	-1.98624	-1.93632	-1.37825
C	-1.85835	-3.10377	-0.59910
C	-2.93941	-1.88955	-2.41808
C	-2.67114	-4.19122	-0.89008
C	-3.72726	-3.00989	-2.65343
C	-3.60724	-4.17401	-1.91280
H	-2.56695	-5.08507	-0.28326
H	-4.46131	-2.96245	-3.45184
C	2.63803	-0.12178	1.61194
C	3.37211	-1.29121	1.85319
C	3.25480	1.13849	1.62851
C	4.73265	-1.17289	2.10258
C	4.61679	1.20163	1.88071
C	5.37362	0.05948	2.11655
H	5.30752	-2.07346	2.29564
H	5.10114	2.17325	1.89404
C	-0.89552	-3.25815	0.53731
H	0.13539	-3.15940	0.19495
H	-1.06115	-2.51809	1.32379
H	-1.01138	-4.24460	0.98653
C	-3.16032	-0.71847	-3.33317
H	-3.10270	0.25224	-2.84156
H	-2.41438	-0.70532	-4.13133
H	-4.14664	-0.79524	-3.79248
C	-4.44370	-5.37735	-2.21450
H	-5.36265	-5.10452	-2.73536
H	-3.89497	-6.07383	-2.85600
H	-4.70936	-5.91570	-1.30286
C	6.84817	0.15517	2.35702
H	7.10807	1.07911	2.87661
H	7.21160	-0.68722	2.94757
H	7.39217	0.14997	1.40732
C	2.72971	-2.64430	1.83097
H	1.78177	-2.65462	2.37826
H	2.52054	-2.95946	0.80402
H	3.38263	-3.39126	2.28351
C	2.48000	2.39002	1.35248
H	2.16067	2.42541	0.30604
H	1.58088	2.45723	1.97387
H	3.08583	3.27520	1.54755
S	-0.17439	0.35263	-2.73999

R = Mes*

P	-1.15529	0.63875	0.08184
C	-0.35542	-0.74806	0.47896
P	1.34718	-1.15834	0.79731
Cl	-1.87650	-3.08404	-1.13439
C	2.95917	-0.52369	0.18642

C	3.50747	-1.15811	-0.96692
C	3.85194	0.10982	1.09480
C	4.85922	-1.46826	-0.94731
C	5.19215	-0.26384	1.04954
C	5.70754	-1.12807	0.09783
H	5.26898	-1.99445	-1.80009
H	5.86048	0.16491	1.78312
C	-2.93376	0.26238	0.13528
C	-3.66359	0.00326	-1.05679
C	-3.51948	-0.01391	1.39650
C	-4.83058	-0.74208	-0.94424
C	-4.67505	-0.78758	1.41179
C	-5.31639	-1.21880	0.26435
H	-5.37095	-0.98643	-1.84345
H	-5.09438	-1.05782	2.37237
C	-3.30362	0.57567	-2.44147
C	-3.10153	0.55250	2.77239
C	-6.52473	-2.14317	0.36346
C	3.53931	1.31931	2.00236
C	2.80334	-1.36099	-2.32318
C	7.15033	-1.61622	0.11016
C	-4.32214	1.30087	3.34256
H	-4.06477	1.72596	4.31633
H	-4.61994	2.11925	2.68249
H	-5.18662	0.65261	3.48331
C	-1.97549	1.58340	2.75563
H	-0.99502	1.14308	2.57491
H	-2.14756	2.37625	2.02285
H	-1.92400	2.06187	3.73760
C	-2.71358	-0.57404	3.73416
H	-3.53326	-1.27794	3.88858
H	-1.86523	-1.14488	3.35109
H	-2.44462	-0.15563	4.70835
C	-7.63247	-1.46479	1.17760
H	-8.49930	-2.12680	1.25556
H	-7.30519	-1.22691	2.19177
H	-7.95588	-0.53556	0.70191
C	-7.09050	-2.50749	-1.00654
H	-7.93428	-3.19008	-0.88065
H	-7.45668	-1.62935	-1.54474
H	-6.34767	-3.01075	-1.63008
C	-6.09371	-3.43699	1.06542
H	-5.29952	-3.93765	0.50696
H	-5.72088	-3.24487	2.07356
H	-6.94308	-4.12111	1.14619
C	-4.30285	0.14762	-3.52088
H	-5.32107	0.47468	-3.29924
H	-4.01088	0.61398	-4.46427
H	-4.30510	-0.93409	-3.67554
C	-3.37762	2.10637	-2.35663
H	-4.38043	2.42805	-2.06469
H	-2.67073	2.51748	-1.63261
H	-3.14513	2.54853	-3.32906
C	-1.92849	0.13703	-2.94639
H	-1.10530	0.53559	-2.35122
H	-1.83818	-0.94961	-2.96012
H	-1.77820	0.51738	-3.96078
C	2.15307	1.91316	1.78268
H	2.04227	2.24281	0.74820
H	1.32116	1.25086	2.00986
H	2.03066	2.79636	2.41395
C	4.50473	2.44290	1.56302
H	4.24796	3.36112	2.09753
H	5.54686	2.21044	1.78289
H	4.41438	2.64090	0.49320
C	3.80499	1.04922	3.48710

H	4.82526	0.69494	3.64858
H	3.68661	1.97753	4.05198
H	3.12954	0.31110	3.91684
C	1.35580	-0.89324	-2.37018
H	1.02742	-0.87674	-3.41224
H	0.67496	-1.55554	-1.83830
H	1.25295	0.12366	-1.98215
C	2.87984	-2.80584	-2.82288
H	2.32604	-3.47865	-2.16847
H	2.44136	-2.86575	-3.82249
H	3.90884	-3.16380	-2.89483
C	3.56805	-0.45568	-3.31226
H	3.53292	0.58930	-2.99479
H	4.61496	-0.74272	-3.41809
H	3.09844	-0.52651	-4.29688
C	7.90975	-1.14533	1.34756
H	8.00438	-0.05694	1.38016
H	7.42766	-1.47832	2.27020
H	8.92043	-1.55975	1.33389
C	7.88236	-1.09697	-1.13319
H	7.41857	-1.45058	-2.05604
H	7.88924	-0.00459	-1.15691
H	8.91922	-1.44391	-1.12844
C	7.15133	-3.14972	0.09685
H	6.64586	-3.54974	0.97910
H	6.64633	-3.54674	-0.78585
H	8.17856	-3.52414	0.09319
Au	-0.29111	2.60613	-0.50676
Cl	0.60840	4.60656	-1.10591
C	1.44143	-1.22833	2.61246
H	2.39468	-1.68190	2.88613
H	1.31107	-0.27586	3.11588
H	0.63516	-1.90455	2.90615
Si	-1.07669	-2.59124	0.79456
S	1.18791	-3.12487	0.32472

Coordination through π(C=P) bond

R= H

P	0.62132	0.19639	1.02280
C	0.33040	-0.02028	-0.66668
P	-1.20925	-0.29468	-1.48439
Cl	1.24811	-2.70890	-1.77816
Si	1.48243	-0.61229	-2.18398
H	2.02842	-0.05778	0.99057
H	-1.61649	-1.59014	-1.11035
Au	0.69914	2.09130	-0.40997
Cl	0.97292	4.29482	-0.76333
C	-2.61807	0.74585	-1.07739
H	-3.47972	0.41266	-1.66022
H	-2.84071	0.65649	-0.01133
H	-2.38962	1.78317	-1.32424
S	-0.59472	-0.22027	-3.38021

R = Me

P	0.62994	0.10033	1.04431
C	0.28386	0.15215	-0.64737
P	-1.28342	-0.13799	-1.41506
Cl	1.65928	-2.21549	-2.08788
Si	1.40378	-0.07866	-2.26966
C	-2.63586	0.96700	-0.97173
H	-3.52531	0.67849	-1.53673
H	-2.84158	0.88994	0.09839
H	-2.36641	1.99327	-1.22132
C	2.42987	-0.24122	1.09923
H	2.96691	0.11377	0.21801
H	2.85219	0.20610	2.00070
H	2.55026	-1.32732	1.16506
C	-1.83431	-1.79931	-0.99536
H	-2.08472	-1.84897	0.06688
H	-2.71022	-2.04787	-1.59763
H	-1.02481	-2.49398	-1.22301
Au	0.52998	2.21723	-0.05647
Cl	0.68582	4.46343	0.02018
S	-0.72796	0.02126	-3.33743

R = *t*-Bu

P	0.60044	0.24471	0.80602
C	0.37444	0.09833	-0.90896
P	-1.22705	-0.27560	-1.61386
Cl	2.24632	-1.92492	-2.56235
Si	1.41939	0.05951	-2.59000
C	-2.48537	0.98957	-1.32603
H	-3.42777	0.67995	-1.78140
H	-2.62627	1.15254	-0.25647
H	-2.15366	1.91653	-1.79398
Au	0.55399	2.23291	-0.50900
Cl	0.72754	4.48292	-0.55325
C	2.39942	-0.02651	1.23548
C	2.53593	-1.54385	1.40242
C	3.42082	0.49574	0.23896
C	2.58518	0.66457	2.58835
H	1.78704	-1.95133	2.08900
H	2.45266	-2.05926	0.44340
H	3.52081	-1.76942	1.82442
H	3.33613	1.57506	0.09559
H	4.42848	0.28805	0.61575
H	3.33975	0.00689	-0.73385
H	3.58827	0.45148	2.96963
H	2.48182	1.74929	2.50354
H	1.86307	0.31137	3.32966
C	-1.93728	-1.88095	-1.00999
C	-3.05870	-2.27815	-1.97135
C	-0.84994	-2.95029	-1.02445
C	-2.49328	-1.70242	0.40216
H	-3.87852	-1.55638	-1.98130
H	-2.68967	-2.40207	-2.99091
H	-3.47121	-3.23581	-1.64182
H	-0.04796	-2.72872	-0.31886
H	-1.29830	-3.90549	-0.73672
H	-0.40466	-3.06502	-2.01360
H	-2.83882	-2.67536	0.76313
H	-1.73766	-1.33946	1.10277
H	-3.34634	-1.02168	0.42813
S	-0.70424	-0.36628	-3.55758

R = Ph

P	0.77923	-0.16379	0.87286
C	0.40685	-0.13949	-0.73132
P	-1.23761	-0.12531	-1.36905
Cl	1.82974	-1.85524	-2.95145
Si	1.38653	0.18155	-2.40226
C	-2.32287	1.09874	-0.61638
H	-3.27619	1.09028	-1.14856
H	-2.48695	0.89067	0.44245
H	-1.85183	2.07603	-0.72465
Au	-0.66435	-0.05733	2.55257
Cl	-2.23475	0.03110	4.18381
C	-2.03241	-1.73310	-1.19466
C	-2.76860	-2.03564	-0.04873
C	-1.86646	-2.69063	-2.19157
C	-3.33912	-3.29010	0.09005
H	-2.89803	-1.30824	0.74438
C	-2.43995	-3.94310	-2.04351
H	-1.28826	-2.45078	-3.07611
C	-3.17589	-4.24228	-0.90633
H	-3.91109	-3.52122	0.98081
H	-2.30985	-4.68700	-2.82058
H	-3.62446	-5.22275	-0.79428
C	2.52812	-0.25754	1.18376
C	3.44327	-0.71311	0.23029
C	2.98015	0.16391	2.43825
C	4.79498	-0.71876	0.52768
H	3.10091	-1.10109	-0.72157
C	4.33319	0.15646	2.72423
H	2.26599	0.50157	3.18235
C	5.24023	-0.28042	1.76758
H	5.50274	-1.07619	-0.21078
H	4.68104	0.48859	3.69513
H	6.30027	-0.29003	1.99422
S	-0.81916	0.33762	-3.28112

R = Mes

P	0.64723	0.02295	1.13559
C	-0.00722	0.37778	-0.45758
P	-1.72726	0.01203	-0.78076
Cl	1.52628	0.76761	-3.45157
Si	0.22700	1.53392	-1.99213
C	-2.90719	0.90017	0.26733
H	-3.92164	0.54468	0.07882
H	-2.66481	0.82152	1.32371
H	-2.83118	1.94283	-0.04332
Au	0.29859	2.35544	0.37255
Cl	0.72891	4.34701	1.40808
C	2.44865	-0.18932	0.97009
C	3.27169	0.47042	1.90339
C	3.01382	-1.10699	0.06451

C	4.64463	0.26442	1.85264
C	4.39086	-1.28095	0.06137
C	5.22822	-0.59065	0.92906
H	5.27444	0.78581	2.56743
H	4.82207	-1.99433	-0.63517
C	-2.05308	-1.78001	-0.76448
C	-1.74633	-2.57764	-1.89105
C	-2.61008	-2.39292	0.38379
C	-2.11668	-3.91760	-1.88063
C	-2.95173	-3.73758	0.32625
C	-2.74848	-4.51473	-0.80324
H	-1.87887	-4.51775	-2.75321
H	-3.37550	-4.19623	1.21409
C	2.71825	1.39170	2.94755
H	2.42699	2.35886	2.52636
H	1.82410	0.97907	3.42395
H	3.46121	1.57331	3.72546
C	2.18741	-1.94236	-0.86699
H	1.28646	-2.32500	-0.38031
H	1.86801	-1.36908	-1.73904
H	2.76829	-2.79495	-1.22238
C	6.71390	-0.76669	0.86884
H	7.17815	-0.57371	1.83757
H	6.98394	-1.77590	0.55181
H	7.15588	-0.06981	0.14956
C	-2.81213	-1.72633	1.71662
H	-2.91771	-2.49317	2.48399
H	-1.96215	-1.10384	2.00165
H	-3.71772	-1.11774	1.74781
C	-0.99917	-2.11722	-3.10850
H	-1.65685	-1.63188	-3.83125
H	-0.20213	-1.41311	-2.87935
H	-0.54789	-2.98347	-3.59455
C	-3.17481	-5.94756	-0.84631
H	-2.55662	-6.52739	-1.53330
H	-3.12336	-6.40922	0.14121
H	-4.21075	-6.02701	-1.19057
S	-1.90078	0.88781	-2.61537

R = Mes*

P	0.70412	0.14367	0.96509
C	0.45160	0.12109	-0.77629
P	-1.15583	-0.05873	-1.65541
Cl	2.86008	-0.72001	-3.01614
Au	0.53569	2.25375	-0.28609
Cl	0.31369	4.51416	-0.03763
C	2.54089	0.01309	1.15384
C	3.24964	-1.13542	0.70769
C	3.29372	1.12701	1.60960
C	4.58691	-0.98999	0.37213
C	4.62985	1.20800	1.21502
C	5.27934	0.20523	0.51804
H	5.11640	-1.85414	-0.00906
H	5.18465	2.09243	1.49425
C	2.87408	2.16323	2.67634
C	2.74353	-2.58885	0.79640
C	6.70799	0.33653	0.00412
C	3.76494	1.86073	3.89966
H	3.59865	0.84327	4.26337
H	4.82596	1.96589	3.67035
H	3.52202	2.55663	4.70749
C	3.13581	3.60420	2.23011
H	2.53748	3.87535	1.36025
H	2.86899	4.28882	3.04001
H	4.18645	3.77767	1.99184
C	1.43800	2.06564	3.18370
H	1.33635	2.71001	4.06140
H	0.70251	2.40989	2.45642
H	1.18145	1.04996	3.49639
C	3.00449	-3.37706	-0.48891
H	2.52511	-2.90890	-1.34646
H	4.06998	-3.45813	-0.70955
H	2.61726	-4.39451	-0.38206
C	3.54523	-3.24014	1.93999
H	4.61725	-3.22893	1.73876
H	3.37576	-2.71665	2.88432
H	3.23295	-4.28142	2.06429
C	1.27557	-2.74694	1.17430
H	1.03514	-2.22907	2.10741
H	0.60104	-2.39866	0.39304
H	1.06193	-3.80805	1.33307
C	7.28925	1.72404	0.26192
H	6.68550	2.50753	-0.20241
H	7.37331	1.94062	1.33014
H	8.29392	1.78288	-0.16371
C	6.71000	0.08593	-1.50868
H	7.72750	0.16675	-1.90181
H	6.33069	-0.90802	-1.75382
H	6.08207	0.81373	-2.02731
C	7.60667	-0.69793	0.69162
H	7.61920	-0.54844	1.77424
H	7.27371	-1.71939	0.49664
H	8.63252	-0.60769	0.32341
C	-2.37551	-1.46087	-1.68143
C	-2.36836	-2.34272	-2.80254
C	-3.60305	-1.29410	-0.98131
C	-3.58754	-2.63394	-3.39944
C	-1.18047	-3.19460	-3.28986
C	-4.78235	-1.64444	-1.63166
C	-3.76045	-0.95140	0.51307
C	-4.81007	-2.21597	-2.89343
H	-3.57487	-3.24551	-4.29302
C	-0.84910	-2.99772	-4.77076
C	-1.62073	-4.66214	-3.09326
C	0.06943	-3.03505	-2.44129
H	-5.71563	-1.46796	-1.11522
C	-2.45264	-0.65714	1.22818
C	-4.30920	-2.23864	1.16403
C	-4.76132	0.17675	0.78138
C	-6.09801	-2.48346	-3.66104
H	-0.09505	-3.72951	-5.07230
H	-1.72571	-3.14793	-5.40489
H	-0.45249	-2.00300	-4.96457
H	-2.45295	-4.94258	-3.73982
H	-0.77882	-5.31816	-3.32991
H	-1.91695	-4.84964	-2.05823
H	0.52570	-2.05159	-2.51952
H	-0.14643	-3.25361	-1.39375
H	0.82174	-3.75233	-2.77690
H	-1.71347	-1.43894	1.04430
H	-2.00901	0.30313	0.97044
H	-2.62432	-0.62297	2.30666
H	-4.41376	-2.08323	2.24129
H	-5.28512	-2.51752	0.76529
H	-3.62669	-3.07680	1.00517
H	-5.75543	-0.05566	0.39613
H	-4.85723	0.32686	1.85970
H	-4.45151	1.12552	0.34193
C	-7.32488	-1.92900	-2.94201
C	-6.28610	-3.99432	-3.84417
C	-5.99951	-1.81375	-5.03696
H	-7.49195	-2.41962	-1.97968

H	-7.24288	-0.85262	-2.77141
H	-8.21381	-2.10077	-3.55326
H	-5.46416	-4.44136	-4.40664
H	-6.34867	-4.50174	-2.87841
H	-7.21079	-4.19226	-4.39298
H	-5.88016	-0.73206	-4.93897
H	-5.15215	-2.19278	-5.61161
H	-6.90861	-2.00504	-5.61333
Si	1.50835	0.77480	-2.31340
S	-0.40203	0.22919	-3.52138
C	-2.26746	1.37022	-1.45765
H	-2.62817	1.51125	-0.44360
H	-1.71751	2.25669	-1.77425
H	-3.10960	1.21400	-2.13423

PCPX-E(II)-AuCl model systems

E = Ge(II)

[R-P=C(Ge(II)Cl)-P(O)ClR](AuCl)

Coordination through Ge(II) atom

R= H

P	0.49593	0.37629	0.47512
C	0.10649	0.28291	-1.13263
P	-1.43497	-0.00849	-1.90287
Ge	0.89471	0.67379	-2.96703
Cl	1.52944	-1.31599	-3.55201
O	-1.08636	0.42819	-3.32311
Au	1.90229	2.65137	-3.65924
Cl	2.88750	4.60344	-4.26329
H	1.87506	0.70748	0.30856
H	-1.88686	-1.33664	-1.84821
Cl	-2.98868	0.97375	-1.14308

R = Me

P	0.54375	0.37472	0.45453
C	0.12537	0.26610	-1.14339
P	-1.42167	-0.06077	-1.88376
Ge	0.86004	0.67209	-2.98343
Cl	1.55327	-1.30268	-3.60086
O	-1.08533	0.38798	-3.31469
C	-1.98499	-1.75093	-1.82496
H	-2.21006	-2.03541	-0.79639
H	-2.87718	-1.85246	-2.44529
H	-1.18134	-2.37374	-2.22406
C	2.32440	0.78963	0.40819
H	2.89420	-0.14519	0.38639
H	2.59436	1.38776	-0.46403
H	2.59427	1.32185	1.32177
Au	1.87845	2.64754	-3.67856
Cl	2.89271	4.59647	-4.25876
Cl	-2.94005	1.01039	-1.13490

R = *t*-Bu

P	0.45371	0.54781	0.09602
C	0.36071	0.16846	-1.51495
P	-1.10133	-0.29805	-2.37643
Ge	1.35454	0.11046	-3.28660
Cl	2.28296	-1.86353	-3.26017
O	-0.48989	-0.43697	-3.78223
Au	2.27711	1.90667	-4.45464
Cl	3.16188	3.70776	-5.52806
C	2.21043	0.99396	0.51922
C	3.21153	0.89626	-0.61568
C	2.13092	2.43163	1.04761
C	2.59496	0.04831	1.66285
H	3.27545	-0.11756	-1.01817
H	2.97715	1.58774	-1.42912
H	4.20801	1.16339	-0.24902
H	1.39861	2.53316	1.85354
H	3.10773	2.72037	1.44801
H	1.87150	3.13883	0.25634
H	3.56925	0.34605	2.06275
H	1.87404	0.08356	2.48451
H	2.67573	-0.98649	1.32101
C	-2.01299	-1.79915	-1.87551
C	-3.07656	-2.07920	-2.93897
C	-0.99165	-2.93955	-1.83684
C	-2.64504	-1.59564	-0.50140
H	-3.83316	-1.29404	-2.97688
H	-2.63490	-2.18868	-3.93107
H	-3.57660	-3.01764	-2.68468
H	-0.21451	-2.76899	-1.09001
H	-1.52032	-3.85909	-1.57219
H	-0.50897	-3.08902	-2.80337
H	-3.13327	-2.52773	-0.20464
H	-1.90050	-1.35351	0.26093
H	-3.39981	-0.80811	-0.51107
Cl	-2.45763	1.19144	-2.36947

R = Ph

P	0.66627	0.40142	0.27965
C	0.22445	0.18329	-1.30685
P	-1.37382	-0.14504	-1.93219
Ge	0.83790	0.55903	-3.19097
Cl	1.58312	-1.38479	-3.86669
O	-1.10693	0.18538	-3.40962
Au	1.72748	2.54565	-4.02355
Cl	2.62382	4.49566	-4.77989
C	2.42372	0.76740	0.25647
C	2.90165	1.47586	1.36452
C	3.31625	0.39694	-0.75455
C	4.23249	1.85089	1.43565
H	2.21869	1.74584	2.16411
C	4.64679	0.76260	-0.67348
H	2.97986	-0.20978	-1.58781
C	5.10286	1.49755	0.41481
H	4.59157	2.41630	2.28740
H	5.33276	0.47491	-1.46127
H	6.14583	1.78755	0.46938
C	-2.02604	-1.79119	-1.73628
C	-1.85321	-2.68943	-2.78759
C	-2.63264	-2.19696	-0.54820
C	-2.29808	-3.99447	-2.64599
H	-1.37674	-2.36547	-3.70458
C	-3.06657	-3.50352	-0.41669
H	-2.77248	-1.49491	0.26579
C	-2.90050	-4.40020	-1.46504
H	-2.17207	-4.69454	-3.46317
H	-3.54088	-3.82306	0.50351
H	-3.24623	-5.42204	-1.35899
Cl	-2.77032	1.07030	-1.15379

R = Mes

P	0.75031	0.19211	0.74825
C	0.19021	0.05019	-0.80791
P	-1.39146	-0.18457	-1.53829
Ge	0.86291	0.77259	-2.55927
Cl	1.54326	-1.02246	-3.61042
O	-1.08770	0.53488	-2.86495

Au	1.82508	2.86284	-2.94561
Cl	2.78704	4.91458	-3.19906
C	2.46602	0.73515	0.48764
C	2.77492	2.08637	0.70574
C	3.46709	-0.18402	0.14265
C	4.08437	2.50700	0.52788
C	4.76506	0.28461	-0.01505
C	5.09177	1.62455	0.15654
H	4.31989	3.55737	0.66908
H	5.54610	-0.42183	-0.27996
C	-2.01075	-1.84871	-1.74611
C	-2.41028	-2.32066	-3.01713
C	-2.09021	-2.68567	-0.61418
C	-2.84508	-3.63635	-3.10964
C	-2.53615	-3.98844	-0.77745
C	-2.90679	-4.48954	-2.01663
H	-3.15825	-4.00293	-4.08190
H	-2.60088	-4.62751	0.09751
C	-2.41548	-1.50631	-4.27750
H	-2.93872	-0.55738	-4.15941
H	-1.40160	-1.26869	-4.60337
H	-2.90587	-2.07177	-5.06982
C	-1.70430	-2.24857	0.76586
H	-0.62110	-2.30378	0.90620
H	-2.01044	-1.22714	0.99670
H	-2.16346	-2.90262	1.50765
C	-3.35087	-5.90905	-2.17434
H	-2.50096	-6.54630	-2.43809
H	-3.77873	-6.29913	-1.24969
H	-4.09134	-6.00887	-2.96960
C	3.17138	-1.64268	-0.04844
H	2.54523	-2.03783	0.75831
H	2.64407	-1.82673	-0.98862
H	4.09447	-2.22329	-0.06537
C	1.71040	3.07683	1.06845
H	1.15808	3.39731	0.17924
H	0.98342	2.65808	1.77396
H	2.14249	3.96882	1.52314
C	6.48747	2.11556	-0.07065
H	6.55137	2.65248	-1.02176
H	6.79689	2.81017	0.71328
H	7.20152	1.29155	-0.10287
Cl	-2.84859	0.84840	-0.59201

R = Mes*

P	-0.44970	1.78621	1.50716
C	0.17918	0.65416	0.46076
P	1.74594	-0.12584	0.20883
Cl	-0.97977	0.54918	-2.72339
O	1.34806	-0.87819	-1.07950
C	3.46082	0.44993	0.08846
C	4.04003	0.48963	-1.21581
C	4.31935	0.38506	1.21690
C	5.35375	0.06443	-1.33497
C	5.63438	-0.01323	1.00618
C	6.14941	-0.28482	-0.25077
H	5.78257	0.02388	-2.32738
H	6.27878	-0.10756	1.86885
C	-2.21860	1.73702	1.03111
C	-2.71155	2.65987	0.08261
C	-3.05722	0.70617	1.53008
C	-3.92232	2.36058	-0.53923
C	-4.24190	0.46561	0.84709
C	-4.66517	1.23221	-0.23234
H	-4.28931	3.02751	-1.30279
H	-4.86256	-0.35903	1.16412
C	-2.03889	4.01711	-0.20955
C	-2.83060	0.00194	2.88538
C	-5.92601	0.83805	-0.99393
C	4.00055	0.89436	2.63521
C	3.40030	1.11282	-2.47159
C	7.54841	-0.84402	-0.47279
C	-3.91395	-1.04133	3.17305
H	-3.72453	-1.47984	4.15531
H	-4.91238	-0.60024	3.20451
H	-3.90802	-1.85403	2.44204
C	-2.93083	1.08841	3.96803
H	-2.14603	1.84231	3.85731
H	-3.89538	1.59940	3.92243
H	-2.82676	0.63916	4.95986
C	-1.48905	-0.71520	3.04656
H	-1.29306	-1.40935	2.22704
H	-0.64291	-0.02593	3.12271
H	-1.50304	-1.28551	3.97922
C	-7.12016	0.78407	-0.03372
H	-8.02522	0.50403	-0.57962
H	-6.97309	0.04811	0.75878
H	-7.29230	1.75675	0.43461
C	-6.25786	1.82160	-2.11289
H	-7.15361	1.48311	-2.63891
H	-6.46129	2.82534	-1.72964
H	-5.45070	1.88792	-2.84666
C	-5.70960	-0.54755	-1.61567
H	-4.87551	-0.53246	-2.32139
H	-5.49020	-1.30740	-0.86311
H	-6.60769	-0.85933	-2.15640
C	-2.90022	4.89022	-1.12593
H	-3.89843	5.05809	-0.71593
H	-2.41922	5.86506	-1.23395
H	-3.00116	4.46307	-2.12635
C	-1.88427	4.78042	1.11330
H	-2.85308	4.91985	1.59848
H	-1.22852	4.26224	1.81788
H	-1.44843	5.76616	0.92718
C	-0.67146	3.89347	-0.88713
H	0.09148	3.47828	-0.22445
H	-0.72017	3.25985	-1.77388
H	-0.32240	4.88658	-1.18474
C	2.59314	1.44810	2.80424
H	2.34800	2.17105	2.02227
H	1.82914	0.67247	2.83005
H	2.52448	1.97637	3.75806
C	4.95004	2.08863	2.86478
H	4.74982	2.52374	3.84752
H	5.99974	1.79547	2.83453
H	4.79357	2.86396	2.11090
C	4.26764	-0.15251	3.71993
H	5.29778	-0.51202	3.69728
H	4.09756	0.29236	4.70389
H	3.60685	-1.01317	3.61951
C	2.23273	2.03078	-2.11955
H	1.87487	2.52548	-3.02534
H	1.36976	1.51480	-1.70789
H	2.54721	2.80555	-1.41520
C	2.99802	0.06569	-3.51418
H	2.21284	-0.59174	-3.15092
H	2.63916	0.56792	-4.41644
H	3.85866	-0.54767	-3.79349
C	4.44920	2.03686	-3.12342
H	4.85895	2.75013	-2.40437
H	5.27757	1.49047	-3.57533
H	3.96463	2.60159	-3.92356

C	8.25126	-1.18099	0.83948
H	8.42255	-0.29236	1.45245
H	7.68311	-1.90438	1.42955
H	9.22760	-1.62186	0.62607
C	8.40063	0.18209	-1.22944
H	7.97731	0.42249	-2.20653
H	8.49001	1.11227	-0.66295
H	9.40618	-0.21524	-1.39129
C	7.43778	-2.12710	-1.30560
H	6.84294	-2.88191	-0.78599
H	6.97089	-1.94222	-2.27496
H	8.43280	-2.54219	-1.48653
Ge	-0.61327	-0.59861	-0.90441
Au	-2.01248	-2.41514	-0.44789
Cl	-3.47523	-4.08183	0.11750
Cl	1.87201	-1.60622	1.60276

Coordination through O atom

R= H

P	0.51603	0.18548	0.69503
C	0.28631	0.28298	-0.94351
P	-1.21534	0.36274	-1.80128
Ge	1.43002	0.67859	-2.64760
Cl	1.31938	-1.39576	-3.41232
O	-0.76372	1.09048	-3.07636
Au	-2.00204	1.37387	-4.76627
Cl	-3.31568	1.66448	-6.53186
H	-1.76368	-0.89376	-2.09474
H	1.94713	0.21213	0.68662
Cl	-2.72826	1.28323	-0.89235

R = Me

P	0.53705	0.13118	0.68073
C	0.28486	0.28691	-0.94799
P	-1.23693	0.31848	-1.77141
Ge	1.35673	0.80889	-2.66094
Cl	1.51287	-1.26447	-3.45942
O	-0.78853	1.00893	-3.08332
C	-1.97736	-1.27147	-2.08044
H	-2.28162	-1.72300	-1.13507
H	-2.84233	-1.14189	-2.73407
H	-1.22293	-1.88896	-2.57334
C	2.36407	0.13447	0.84262
H	2.71575	-0.90016	0.77664
H	2.85867	0.72106	0.06660
H	2.64042	0.51632	1.82695
Au	-2.00841	1.27863	-4.78313
Cl	-3.31114	1.55154	-6.56163
Cl	-2.67272	1.40004	-0.88255

R = *t*-Bu

P	0.69040	-0.35016	0.45049
C	0.24924	0.30937	-1.00637
P	-1.36248	0.19794	-1.66835
Ge	0.92407	1.62430	-2.49424
Cl	1.79223	0.07317	-3.84086
O	-1.17962	1.12463	-2.89530
Au	-2.56330	1.91781	-4.27225
Cl	-4.00761	2.76178	-5.73548
C	-2.02315	-1.45037	-2.11654
C	-3.40413	-1.29648	-2.75488
C	-1.04032	-2.06151	-3.11867
C	-2.11160	-2.30971	-0.85666
H	-4.13472	-0.88249	-2.05886
H	-3.38043	-0.66675	-3.64708
H	-3.74822	-2.29010	-3.05487
H	-0.03785	-2.17049	-2.70183
H	-1.40830	-3.05508	-3.38797
H	-0.96580	-1.46763	-4.03039
H	-2.47356	-3.29975	-1.14639
H	-1.14057	-2.43843	-0.37388
H	-2.81069	-1.89721	-0.12723
C	2.50034	0.00348	0.73477
C	3.19186	-1.36174	0.64564
C	3.13476	0.98495	-0.23234
C	2.58954	0.53089	2.17028
H	2.73430	-2.09607	1.31472
H	3.16545	-1.75958	-0.37150
H	4.24107	-1.25594	0.94029
H	2.63724	1.95854	-0.20841
H	4.18200	1.15294	0.04092
H	3.13375	0.59988	-1.25603
H	3.64075	0.63539	2.45620
H	2.11573	1.51063	2.27164
H	2.11605	-0.14934	2.88334
Cl	-2.73425	1.01677	-0.43752

R = Ph

P	0.51662	-0.26952	0.76271
C	0.22802	0.10184	-0.83089
P	-1.31794	0.13871	-1.60938
Ge	1.18031	1.21276	-2.30276
Cl	1.72860	-0.45334	-3.68439
O	-0.98779	1.10696	-2.76719
Au	-2.27785	1.83990	-4.26547
Cl	-3.62225	2.61638	-5.85618
C	-1.95383	-1.41557	-2.20368
C	-2.63892	-2.27785	-1.34802
C	-1.70090	-1.78060	-3.52452
C	-3.07261	-3.50329	-1.81934
H	-2.84152	-1.98974	-0.32294
C	-2.14687	-3.00841	-3.98683
H	-1.16323	-1.11191	-4.18474
C	-2.82860	-3.86647	-3.13801
H	-3.60972	-4.17397	-1.15943
H	-1.96217	-3.28990	-5.01642
H	-3.17712	-4.82473	-3.50570
C	2.30380	-0.10270	0.94012
C	3.22298	-0.28319	-0.09877
C	2.77411	0.21051	2.21906
C	4.57726	-0.11440	0.13283
H	2.88195	-0.60529	-1.07753
C	4.12887	0.38975	2.44407
H	2.06915	0.32658	3.03655
C	5.02967	0.23094	1.40026
H	5.28380	-0.26213	-0.67565
H	4.48373	0.64795	3.43498
H	6.09096	0.36255	1.57733
Cl	-2.77604	0.90325	-0.45504

R = Mes

P	0.33031	0.20765	0.82469
C	0.17456	0.04963	-0.81730
P	-1.17402	0.04909	-1.91868
Ge	1.60692	0.47503	-2.24285
Cl	2.00495	-1.63150	-2.87631
O	-0.43545	0.59942	-3.16058
Au	-1.06091	1.92730	-4.68474
Cl	-1.67507	3.33328	-6.29431
C	-2.00161	-1.51637	-2.18657

C	-2.67723	-2.10014	-1.09134
C	-1.95987	-2.18138	-3.43468
C	-3.26008	-3.34747	-1.25270
C	-2.57506	-3.42402	-3.52639
C	-3.21664	-4.03204	-2.45808
H	-3.77285	-3.79164	-0.40543
H	-2.54645	-3.93519	-4.48303
C	2.14513	0.31191	1.01613
C	2.73703	1.57213	1.18954
C	2.91858	-0.85697	1.08936
C	4.10788	1.64331	1.39900
C	4.28516	-0.73447	1.30432
C	4.90044	0.50324	1.45256
H	4.56796	2.61844	1.52925
H	4.88646	-1.63676	1.36553
C	1.91939	2.82774	1.12852
H	1.62540	3.06294	0.10068
H	0.99673	2.74059	1.71234
H	2.48001	3.67958	1.51494
C	2.30095	-2.21578	0.94685
H	1.40407	-2.31828	1.56646
H	2.00396	-2.40899	-0.08783
H	3.00390	-2.99399	1.24620
C	6.38150	0.60509	1.64932
H	6.89594	0.64951	0.68428
H	6.64925	1.50580	2.20416
H	6.77259	-0.26031	2.18717
C	-2.85298	-1.43098	0.23843
H	-1.95930	-0.91325	0.59069
H	-3.65642	-0.69125	0.19774
H	-3.11865	-2.16976	0.99494
C	-1.31836	-1.65390	-4.68258
H	-1.74818	-0.69891	-4.99385
H	-0.24643	-1.50164	-4.55698
H	-1.46737	-2.36561	-5.49411
C	-3.82609	-5.39063	-2.59789
H	-4.65658	-5.52924	-1.90401
H	-4.18849	-5.56161	-3.61275
H	-3.08360	-6.16507	-2.38109
Cl	-2.59930	1.39796	-1.43513

R = Mes*

P	1.99096	2.07364	-0.19543
C	1.03069	0.71910	-0.22922
P	-0.69381	0.48678	-0.34685
C	3.65221	1.28112	-0.20149
C	4.39584	1.23219	1.00825
C	4.10752	0.57093	-1.33169
C	5.40512	0.28846	1.10678
C	5.11267	-0.38232	-1.14243
C	5.73609	-0.58481	0.07330
H	5.95260	0.20880	2.03442
H	5.42476	-0.96924	-1.99532
C	3.73419	0.85651	-2.80285
C	4.17032	2.21102	2.17565
C	6.78538	-1.66756	0.29999
C	2.82407	1.99239	2.87126
H	1.97500	2.24696	2.23128
H	2.70057	0.95313	3.17998
H	2.75784	2.63435	3.75460
C	4.25989	3.65065	1.65109
H	5.23161	3.83416	1.18627
H	3.48584	3.87671	0.91460
H	4.13854	4.35582	2.47805
C	5.24755	2.06768	3.25377
H	5.20103	1.09971	3.75806
H	6.25381	2.20508	2.85106
H	5.08749	2.83689	4.01262
C	3.14538	-0.37677	-3.49296
H	2.18156	-0.65471	-3.06115
H	2.98761	-0.16817	-4.55479
H	3.80556	-1.24263	-3.41901
C	2.77839	2.02742	-3.01889
H	1.75138	1.79367	-2.73646
H	3.10354	2.92809	-2.48852
H	2.75873	2.27683	-4.08366
C	5.04088	1.24842	-3.51946
H	5.49060	2.12935	-3.05457
H	5.78044	0.44772	-3.50415
H	4.82797	1.48549	-4.56530
C	6.99753	-2.53591	-0.93711
H	7.38000	-1.95779	-1.78244
H	7.73205	-3.31329	-0.71424
H	6.07470	-3.03288	-1.24665
C	6.32530	-2.57285	1.44942
H	6.20324	-2.01509	2.37991
H	5.36739	-3.04447	1.21893
H	7.06343	-3.36036	1.62486
C	8.12436	-1.01815	0.66882
H	8.04776	-0.41760	1.57744
H	8.88021	-1.78908	0.84239
H	8.47941	-0.36834	-0.13503
C	-2.15503	1.32820	0.30318
C	-2.76476	0.72260	1.44297
C	-2.94446	2.15741	-0.53607
C	-4.14661	0.63054	1.44511
C	-4.32465	2.02254	-0.45406
C	-4.94939	1.17648	0.45061
H	-4.61837	0.10780	2.26722
H	-4.93303	2.61091	-1.12695
C	-2.04194	0.32575	2.74425
C	-2.11836	-1.17234	3.04482
C	-2.75641	1.08288	3.88276
H	-1.52631	-1.75570	2.34083
H	-1.72969	-1.36222	4.04900
H	-3.14659	-1.53798	3.00738
H	-2.77182	2.15926	3.69473
H	-3.78356	0.74961	4.03212
H	-2.21509	0.90777	4.81594
C	-2.42729	3.33757	-1.38081
C	-3.03965	4.59599	-0.73001
C	-2.88612	3.28106	-2.83997
H	-2.74266	4.67870	0.31825
H	-2.68168	5.48457	-1.25707
H	-4.12905	4.59446	-0.77304
H	-2.46395	2.42423	-3.36392
H	-3.97281	3.22460	-2.92311
H	-2.56406	4.18835	-3.35802
C	-6.44803	0.91227	0.44390
C	-7.07592	1.48446	1.72080
C	-6.67800	-0.60361	0.39530
H	-6.92430	2.56505	1.78550
H	-8.15191	1.29084	1.72469
H	-6.65386	1.02984	2.61925
H	-6.22610	-1.05041	-0.49264
H	-6.25117	-1.10893	1.26341
H	-7.75070	-0.81444	0.38004
Cl	2.03397	-1.61644	1.85455
O	-0.74010	-1.00142	0.09712
Cl	-1.04386	0.36400	-2.35715
Au	-2.35214	-2.29693	-0.36537
Cl	-4.04829	-3.66430	-0.84461

C	-0.91791	3.54132	-1.32503
H	-0.55177	3.55696	-0.29565
H	-0.36119	2.79428	-1.88796
H	-0.67085	4.50927	-1.76792
C	-0.59234	0.79298	2.78877
H	0.06601	0.23252	2.12846
H	-0.51143	1.85891	2.55944
H	-0.19789	0.63920	3.79612
C	-7.13878	1.54498	-0.76116
H	-7.07537	2.63645	-0.74678
H	-6.71720	1.18424	-1.70254
H	-8.19865	1.28113	-0.75073
Ge	1.46236	-1.32571	-0.28189

Coordination through P(sp²) atom

R= H

P	0.42616	0.27709	0.53146
C	0.16492	0.17232	-1.07919
P	-1.31066	0.05384	-1.98209
Ge	1.18971	0.46642	-2.92832
Cl	1.34354	-1.71807	-3.35094
O	-0.86635	0.57640	-3.33655
Au	-0.90489	0.26896	2.27645
Cl	-2.27101	0.25672	4.06726
H	1.82650	0.38822	0.67613
H	-1.79337	-1.26257	-2.02964
Cl	-2.88576	1.03861	-1.25472

R = Me

P	0.51440	0.33559	0.49676
C	0.18542	0.22799	-1.09940
P	-1.33172	0.00906	-1.91836
Ge	1.11190	0.45592	-2.99496
Cl	1.48243	-1.74291	-3.25624
O	-0.92001	0.36423	-3.34451
C	-2.01322	-1.63095	-1.77464
H	-2.29777	-1.82712	-0.73984
H	-2.88639	-1.70991	-2.42469
H	-1.23946	-2.33008	-2.09964
C	2.29184	0.51333	0.77078
H	2.65102	-0.37434	1.29817
H	2.82875	0.62514	-0.17042
H	2.46774	1.37633	1.41655
Au	-0.86339	0.22975	2.21257
Cl	-2.27906	0.08358	3.96635
Cl	-2.79375	1.23027	-1.27883

R = *t*-Bu

P	0.73327	-0.34646	0.50134
C	0.17880	0.23589	-0.92681
P	-1.43192	0.14234	-1.60317
Ge	0.67571	1.66382	-2.42813
Cl	1.61721	0.23933	-3.88232
O	-1.28580	1.13328	-2.75887
C	-2.06972	-1.47571	-2.17393
C	-3.37721	-1.20016	-2.92137
C	-1.02890	-2.05565	-3.13432
C	-2.30097	-2.42266	-1.00199
H	-4.15522	-0.82077	-2.25712
H	-3.23357	-0.48986	-3.73737
H	-3.72913	-2.14301	-3.34863
H	-0.07421	-2.24381	-2.64035
H	-1.41042	-3.00913	-3.50955
H	-0.84471	-1.39968	-3.98537
H	-2.68105	-3.36939	-1.39547
H	-1.37786	-2.64053	-0.46075
H	-3.03299	-2.03642	-0.29169
C	2.52777	-0.01611	0.79646
C	3.20964	-1.38825	0.83849
C	3.14371	0.85568	-0.28532
C	2.63515	0.65836	2.16668
H	2.75929	-2.04424	1.58756
H	3.16944	-1.88894	-0.13146
H	4.26185	-1.24932	1.10459
H	2.67894	1.84411	-0.32413
H	4.20437	1.00427	-0.06126
H	3.07718	0.39234	-1.27209
H	3.69283	0.78673	2.41460
H	2.16459	1.64431	2.17182
H	2.17641	0.05562	2.95411
Cl	-2.80785	0.82868	-0.29066
Au	-0.37617	-1.45734	2.06157
Cl	-1.50569	-2.59349	3.66161

R = Ph

P	0.54464	0.49087	0.15773
C	0.40952	-0.02371	-1.39205
P	-1.02931	-0.38713	-2.29854
Ge	1.52631	-0.15031	-3.17791
Cl	2.07823	-2.32241	-2.99370
O	-0.44342	-0.46886	-3.70576
Au	-1.07833	0.98074	1.57498
Cl	-2.77562	1.43100	3.00182
Cl	-2.40255	1.08004	-2.17879
C	2.24951	0.63575	0.65429
C	3.27669	-0.05011	-0.00033
C	2.54385	1.45866	1.74405
C	4.58426	0.11206	0.42018
H	3.04859	-0.73849	-0.80653
C	3.85630	1.62098	2.15247
H	1.74134	1.97227	2.26351
C	4.87492	0.95112	1.48914
H	5.37949	-0.42637	-0.08161
H	4.08391	2.26674	2.99214
H	5.90178	1.07342	1.81422
C	-1.88985	-1.87546	-1.83167
C	-1.59579	-3.04263	-2.53421
C	-2.78381	-1.89519	-0.76146
C	-2.20795	-4.22962	-2.16433
H	-0.89310	-3.01631	-3.35785
C	-3.38321	-3.08780	-0.39825
H	-3.01664	-0.98667	-0.21743
C	-3.09657	-4.25211	-1.09991
H	-1.98675	-5.13902	-2.71021
H	-4.07847	-3.10690	0.43239
H	-3.57177	-5.18342	-0.81353

R = Mes

P	0.45446	0.51661	0.84531
C	0.16398	-0.04834	-0.66332
P	-1.21762	-0.26970	-1.70340
Ge	1.38645	0.18371	-2.36238
Cl	1.76153	-2.00868	-2.71766
O	-0.57370	0.18547	-3.01328
Au	-0.91554	1.04186	2.49759
Cl	-2.29489	1.58523	4.20778
C	2.23182	0.72173	1.04316
C	3.05344	-0.40371	1.21311
C	2.76568	2.02053	1.06330
C	4.41542	-0.19815	1.39003

C	4.13375	2.16786	1.23761
C	4.97473	1.07357	1.40476
H	5.05865	-1.06245	1.52373
H	4.55471	3.16864	1.24186
C	-1.93953	-1.90741	-1.77908
C	-2.34062	-2.54131	-0.58543
C	-2.11930	-2.55103	-3.02452
C	-2.87944	-3.81713	-0.65338
C	-2.66565	-3.82826	-3.02068
C	-3.04042	-4.48539	-1.85794
H	-3.19026	-4.29697	0.26926
H	-2.81019	-4.32371	-3.97538
C	1.89770	3.22794	0.87605
H	1.42914	3.23437	-0.11281
H	1.09217	3.26415	1.61654
H	2.48061	4.14399	0.97306
C	2.50571	-1.79937	1.21508
H	1.63171	-1.88787	1.86784
H	2.19873	-2.11031	0.21256
H	3.25736	-2.50457	1.57070
C	6.44301	1.26351	1.62688
H	6.65506	1.40863	2.69081
H	7.01206	0.39391	1.29472
H	6.81511	2.14245	1.09770
C	-2.20939	-1.91397	0.76768
H	-1.18173	-1.98206	1.13394
H	-2.49318	-0.86044	0.78049
H	-2.84683	-2.42910	1.48638
C	-1.77815	-1.96185	-4.36195
H	-2.20485	-0.96907	-4.50359
H	-0.69861	-1.86403	-4.48595
H	-2.15671	-2.61434	-5.14900
C	-3.59208	-5.87510	-1.90213
H	-4.12345	-6.06375	-2.83629
H	-2.78251	-6.60842	-1.83216
H	-4.27395	-6.06044	-1.07067
Cl	-2.74320	0.99286	-1.27392

R = Mes*

P	-1.15269	0.84528	0.02807
C	-0.25918	-0.47827	0.40047
P	1.39945	-0.99411	0.62444
Cl	-1.42391	-3.08392	-1.40180
O	1.20085	-2.48157	0.32460
C	3.03432	-0.48868	0.02415
C	3.57763	-1.30524	-1.01125
C	3.91685	0.27939	0.82824
C	4.92719	-1.61146	-0.93519
C	5.26067	-0.07881	0.82777
C	5.77682	-1.08739	0.03077
H	5.33527	-2.28450	-1.67804
H	5.92844	0.47093	1.47591
C	-2.88214	0.28852	0.10343
C	-3.58474	-0.04009	-1.08654
C	-3.41913	-0.05901	1.36775
C	-4.67505	-0.89443	-0.96922
C	-4.49829	-0.93646	1.38712
C	-5.11045	-1.41366	0.24099
H	-5.19245	-1.18951	-1.86659
H	-4.88039	-1.25321	2.34910
C	-3.27937	0.55815	-2.47381
C	-3.01735	0.51596	2.74425
C	-6.23831	-2.43491	0.34363
C	3.58493	1.59695	1.55407
C	2.84579	-1.75582	-2.28942
C	7.22407	-1.55481	0.11593
C	-4.28838	1.11023	3.38087
H	-4.03583	1.55294	4.34776
H	-4.71084	1.89457	2.74821
H	-5.06402	0.36457	3.55390
C	-2.01031	1.66295	2.71231
H	-0.99522	1.33697	2.48103
H	-2.30231	2.45296	2.01531
H	-1.96240	2.11642	3.70616
C	-2.46950	-0.58423	3.65791
H	-3.17659	-1.40796	3.77202
H	-1.53675	-0.99612	3.26740
H	-2.26746	-0.17536	4.65194
C	-7.39291	-1.84855	1.16434
H	-8.20349	-2.57796	1.24526
H	-7.08074	-1.58612	2.17722
H	-7.79199	-0.94773	0.69160
C	-6.77977	-2.84111	-1.02445
H	-7.56519	-3.58959	-0.89589
H	-7.21856	-1.99432	-1.55845
H	-6.00246	-3.28177	-1.65335
C	-5.70332	-3.69185	1.04081
H	-4.87803	-4.13061	0.47508
H	-5.34068	-3.47349	2.04742
H	-6.49633	-4.43991	1.12617
C	-4.21654	0.01063	-3.55507
H	-5.26601	0.22955	-3.34647
H	-3.96821	0.49316	-4.50270
H	-4.10057	-1.06707	-3.69332
C	-3.52313	2.07185	-2.40351
H	-4.55790	2.28308	-2.12261
H	-2.87270	2.56536	-1.67800
H	-3.33268	2.52844	-3.37839
C	-1.85982	0.26936	-2.96854
H	-1.08645	0.78026	-2.39057
H	-1.64453	-0.79945	-2.95371
H	-1.75506	0.63641	-3.99334
C	2.14183	2.05437	1.39754
H	1.83918	2.02954	0.34779
H	1.43378	1.47684	1.98835
H	2.05273	3.09285	1.72336
C	4.43300	2.67673	0.84776
H	4.21204	3.65157	1.28960
H	5.50355	2.49520	0.94741
H	4.19330	2.72857	-0.21666
C	3.96752	1.57865	3.03617
H	5.02607	1.35501	3.18185
H	3.77806	2.56374	3.47045
H	3.38709	0.84513	3.59495
C	1.49878	-1.06752	-2.48954
H	1.14424	-1.26840	-3.50336
H	0.72001	-1.43942	-1.82855
H	1.58472	0.01723	-2.38033
C	2.68058	-3.27632	-2.35445
H	2.04259	-3.63937	-1.55096
H	2.22635	-3.55565	-3.30884
H	3.64773	-3.78049	-2.28492
C	3.71300	-1.30713	-3.48328
H	3.89278	-0.22955	-3.45971
H	4.67843	-1.81188	-3.52359
H	3.18341	-1.54230	-4.40979
C	7.98553	-0.87631	1.25144
H	8.07086	0.20272	1.09987
H	7.51087	-1.05291	2.21986
H	8.99969	-1.27932	1.30085
C	7.94400	-1.24582	-1.20232
H	7.47793	-1.75286	-2.04934

H	7.94020	-0.17286	-1.40898
H	8.98418	-1.57807	-1.14672
C	7.23898	-3.06855	0.36126
H	6.74092	-3.31792	1.30120
H	6.73492	-3.61369	-0.43911
H	8.26979	-3.42896	0.41513
Cl	1.60912	-0.95509	2.66557
Au	-0.52857	2.91235	-0.50564
Cl	0.10022	5.03194	-1.02227
Ge	-0.84125	-2.52312	0.69473

Coordination through π(C=P) bond

R= H

P	0.43623	-0.14722	0.66482
C	0.13781	0.54961	-0.88573
P	-1.29828	0.45412	-1.87599
Ge	1.19090	1.06622	-2.69026
Cl	1.38634	-1.07733	-3.31623
O	-0.85617	1.19045	-3.12818
Au	0.17742	2.20616	0.47716
Cl	0.16734	4.24408	1.41643
H	1.86350	-0.11760	0.60806
H	-1.65090	-0.88200	-2.12069
Cl	-3.00420	1.19000	-1.13565

R = Me

P	0.46687	-0.01079	0.65209
C	0.10008	0.70276	-0.87326
P	-1.34777	0.49603	-1.84037
Ge	1.11778	1.20593	-2.69074
Cl	1.61047	-0.94523	-3.17058
O	-0.88589	1.08613	-3.16955
C	-1.87187	-1.20325	-1.98548
H	-2.20966	-1.57569	-1.01747
H	-2.68367	-1.25960	-2.71256
H	-1.01301	-1.77697	-2.34094
C	2.29762	-0.09706	0.66556
H	2.56781	-1.09411	0.30141
H	2.77947	0.64973	0.03558
H	2.65253	-0.00420	1.69368
Au	0.07460	2.33598	0.51831
Cl	0.00271	4.32576	1.56449
Cl	-2.98265	1.46977	-1.18147

R = *t*-Bu

P	0.64836	-0.11054	0.45758
C	0.05982	0.71629	-0.94194
P	-1.45929	0.36517	-1.78265
Ge	0.74818	1.65039	-2.75800
Cl	1.81546	-0.14868	-3.59975
O	-1.14353	0.94535	-3.15968
Au	-0.14250	2.12026	0.68401
Cl	-0.43518	3.88688	2.05244
C	-2.05461	-1.36465	-1.88289
C	-3.20792	-1.38791	-2.88900
C	-0.89710	-2.22173	-2.40030
C	-2.51408	-1.85472	-0.51221
H	-4.06122	-0.80018	-2.54679
H	-2.89590	-1.01618	-3.86631
H	-3.53758	-2.42382	-3.00671
H	-0.06429	-2.25653	-1.69592
H	-1.26572	-3.24249	-2.53367
H	-0.51640	-1.86562	-3.35803
H	-2.81557	-2.90178	-0.60484
H	-1.71500	-1.80480	0.23120
H	-3.36991	-1.28945	-0.14138
C	2.50490	0.07272	0.54866
C	3.03324	-1.26305	0.00923
C	3.11744	1.23344	-0.21495
C	2.82068	0.18953	2.04188
H	2.59821	-2.11793	0.53561
H	2.83631	-1.36983	-1.05981
H	4.11677	-1.30338	0.15970
H	2.70924	2.19516	0.10412
H	4.19712	1.25537	-0.03246
H	2.98408	1.12544	-1.29312
H	3.90374	0.13451	2.18713
H	2.47241	1.13942	2.45467
H	2.36564	-0.61979	2.61984
Cl	-3.01255	1.39628	-0.98824

R = Ph

P	0.33247	0.47169	0.48810
C	0.17461	0.67976	-1.21684
P	-1.27291	0.40031	-2.16674
Ge	1.21828	0.93927	-3.08291
Cl	1.70162	-1.22113	-3.51074
O	-0.78479	0.79151	-3.55858
Au	0.26584	2.68612	-0.40739
Cl	0.38812	4.90381	-0.03783
C	-1.87807	-1.27722	-2.10609
C	-2.44831	-1.78567	-0.93974
C	-1.75619	-2.07324	-3.24092
C	-2.89404	-3.09440	-0.91303
H	-2.54784	-1.16143	-0.05823
C	-2.21243	-3.38215	-3.20535
H	-1.30152	-1.66915	-4.13631
C	-2.77700	-3.89070	-2.04617
H	-3.33679	-3.49480	-0.00881
H	-2.12043	-4.00574	-4.08656
H	-3.12951	-4.91553	-2.02208
C	2.09839	0.26321	0.81834
C	3.01628	-0.26271	-0.09200
C	2.52085	0.59117	2.10989
C	4.33981	-0.42919	0.27933
H	2.69157	-0.57335	-1.07831
C	3.84867	0.43880	2.47002
H	1.80644	0.98064	2.82869
C	4.75870	-0.07054	1.55389
H	5.04708	-0.84374	-0.42967
H	4.17344	0.71227	3.46709
H	5.79720	-0.19801	1.83742
Cl	-2.86033	1.52688	-1.63522

R = Mes

P	0.31520	0.36389	1.04217
C	-0.04966	0.67088	-0.61929
P	-1.44180	0.42953	-1.67975
Ge	1.00455	1.47992	-2.29976
Cl	1.80869	-0.40043	-3.25348
O	-0.95021	1.22660	-2.88731
Au	-0.28236	2.58175	0.39048
Cl	-0.61914	4.74303	0.94995
C	-1.86110	-1.27651	-2.04191
C	-2.24953	-2.12497	-0.98344
C	-1.80316	-1.77196	-3.36556
C	-2.46443	-3.46957	-1.24722
C	-2.04481	-3.12634	-3.56097
C	-2.34727	-3.99718	-2.52452
H	-2.75062	-4.11893	-0.42575
H	-1.99745	-3.51060	-4.57480

C	2.13758	0.28147	1.10012
C	2.72786	-0.88837	0.58433
C	2.91829	1.22073	1.79145
C	4.10315	-1.03980	0.68334
C	4.29284	1.02349	1.85635
C	4.90853	-0.08446	1.29156
H	4.55623	-1.94277	0.28497
H	4.89790	1.75771	2.38000
C	2.33097	2.42062	2.47025
H	2.13826	3.23712	1.76811
H	1.38108	2.19205	2.96155
H	3.01476	2.79949	3.23130
C	1.91128	-1.99518	-0.01528
H	1.04364	-2.24082	0.60690
H	1.54258	-1.73547	-1.00970
H	2.51272	-2.90012	-0.10998
C	6.39535	-0.25202	1.34206
H	6.83120	0.29873	2.17715
H	6.67535	-1.30285	1.43767
H	6.85573	0.12491	0.42337
C	-2.53864	-1.64598	0.40706
H	-2.47106	-2.47346	1.11417
H	-1.86901	-0.86139	0.75890
H	-3.55414	-1.24332	0.46043
C	-1.54014	-0.94639	-4.59080
H	-2.17316	-0.06056	-4.63412
H	-0.50618	-0.60271	-4.62892
H	-1.73269	-1.55147	-5.47705
C	-2.54347	-5.45815	-2.77850
H	-2.95159	-5.63819	-3.77437
H	-1.58701	-5.98682	-2.71721
H	-3.21294	-5.90573	-2.04226
Cl	-3.17207	1.29033	-1.05547

R = Mes*

P	0.68315	0.06050	1.14967
C	0.37669	0.14554	-0.56719
P	-1.08247	-0.14747	-1.60295
O	-0.37151	-0.05615	-2.95396
Cl	2.68454	-1.03794	-3.00785
Au	0.26123	2.20288	0.11787
Cl	-0.03897	4.44752	0.29433
C	2.53088	-0.02589	1.24131
C	3.21687	-1.17659	0.76272
C	3.29869	1.09459	1.64313
C	4.53288	-1.02639	0.35423
C	4.60929	1.18238	1.17035
C	5.22466	0.17543	0.44917
H	5.04678	-1.89058	-0.04597
H	5.17157	2.07501	1.40625
C	2.92565	2.13757	2.71852
C	2.72458	-2.63041	0.91170
C	6.62209	0.30790	-0.14446
C	3.92451	1.90893	3.87268
H	3.84123	0.89297	4.26739
H	4.95764	2.06405	3.56056
H	3.70839	2.60965	4.68374
C	3.08422	3.57555	2.21903
H	2.39829	3.80194	1.40253
H	2.86721	4.27233	3.03330
H	4.10079	3.77837	1.87756
C	1.54389	1.97696	3.34606
H	1.47848	2.62945	4.22088
H	0.72970	2.27441	2.68355
H	1.36670	0.95400	3.68869
C	3.04486	-3.49373	-0.31147
H	2.61617	-3.07370	-1.21988
H	4.11860	-3.60455	-0.46936
H	2.63931	-4.49845	-0.16334
C	3.49478	-3.19586	2.12056
H	4.57299	-3.16251	1.95615
H	3.27524	-2.62653	3.02736
H	3.20601	-4.23747	2.29077
C	1.24423	-2.79702	1.23299
H	0.95231	-2.25003	2.13524
H	0.59870	-2.49933	0.40736
H	1.04145	-3.85336	1.43219
C	7.21447	1.69696	0.07667
H	6.58631	2.47848	-0.35782
H	7.35554	1.91780	1.13801
H	8.19466	1.75533	-0.40258
C	6.53999	0.05199	-1.65408
H	7.53505	0.12461	-2.10222
H	6.13955	-0.93902	-1.87563
H	5.89094	0.78331	-2.14100
C	7.55995	-0.72270	0.49504
H	7.63022	-0.57200	1.57527
H	7.22158	-1.74568	0.31874
H	8.56406	-0.62874	0.07215
C	-2.36613	-1.45447	-1.66726
C	-2.33016	-2.33583	-2.79146
C	-3.60266	-1.27145	-0.99121
C	-3.53992	-2.65097	-3.39108
C	-1.10146	-3.10215	-3.31657
C	-4.76743	-1.66246	-1.64205
C	-3.79808	-0.82725	0.46950
C	-4.77360	-2.25794	-2.89137
H	-3.51290	-3.25734	-4.28648
C	-0.60022	-2.57668	-4.66494
C	-1.51051	-4.57555	-3.52335
C	0.01116	-3.15218	-2.27764
H	-5.70840	-1.48394	-1.14158
C	-2.51062	-0.47359	1.19157
C	-4.37455	-2.05498	1.20332
C	-4.78479	0.33532	0.61329
C	-6.05044	-2.56554	-3.66226
H	0.20428	-3.22051	-5.03022
H	-1.40528	-2.59485	-5.40449
H	-0.22158	-1.56148	-4.59305
H	-2.17744	-4.71924	-4.37410
H	-0.60887	-5.15972	-3.72350
H	-1.99251	-4.98860	-2.63408
H	0.43443	-2.18069	-2.03466
H	-0.34680	-3.62725	-1.36082
H	0.84131	-3.74735	-2.66296
H	-1.76460	-1.26455	1.09045
H	-2.08416	0.47245	0.85818
H	-2.70926	-0.35656	2.25939
H	-4.50737	-1.81321	2.26128
H	-5.34216	-2.35977	0.80325
H	-3.69544	-2.90807	1.13138
H	-5.76495	0.10059	0.19512
H	-4.92670	0.55947	1.67374
H	-4.41464	1.23709	0.12583
C	-7.29422	-2.02976	-2.95837
C	-6.20130	-4.08294	-3.82481
C	-5.95910	-1.91223	-5.04667
H	-7.45382	-2.50808	-1.98862
H	-7.23970	-0.94898	-2.80599
H	-8.17529	-2.23405	-3.57102
H	-5.36335	-4.51895	-4.37209
H	-6.26180	-4.57739	-2.85217

H	-7.11585	-4.31015	-4.37924
H	-5.86048	-0.82744	-4.96216
H	-5.10302	-2.28268	-5.61392
H	-6.86241	-2.12893	-5.62317
Ge	1.43861	0.71240	-2.35503
Cl	-2.29407	1.50514	-1.59320

[R-P=C(Ge(II)Cl)-P(O)MeR](AuCl)
Coordination through Ge(II) atom
R= H

P	0.53608	0.35901	0.47014
C	0.11408	0.28943	-1.12944
P	-1.44949	0.02918	-1.89357
Ge	0.89018	0.66444	-2.97085
Cl	1.53924	-1.33059	-3.53882
O	-1.05723	0.44394	-3.32625
C	-2.83411	0.95281	-1.25070
H	-2.58389	2.01458	-1.24744
H	-3.70794	0.78347	-1.88326
H	-3.05089	0.62081	-0.23252
Au	1.91521	2.63497	-3.66708
Cl	2.89046	4.59657	-4.27329
H	1.91255	0.69659	0.29396
H	-1.84301	-1.32292	-1.83820

R = Me

P	0.54552	0.34860	0.44967
C	0.11370	0.26877	-1.14451
P	-1.45412	-0.02789	-1.88436
Ge	0.84722	0.66221	-2.98658
Cl	1.57508	-1.30944	-3.58705
O	-1.06939	0.39078	-3.32910
C	-2.79883	0.97210	-1.25637
H	-2.51105	2.02256	-1.31019
H	-3.69215	0.80900	-1.86294
H	-3.00828	0.70456	-0.21853
C	-1.96126	-1.74305	-1.82050
H	-2.18254	-2.03242	-0.79125
H	-2.84752	-1.88669	-2.44233
H	-1.14348	-2.35355	-2.20648
C	2.32865	0.76199	0.41098
H	2.89835	-0.17277	0.42206
H	2.60719	1.33619	-0.47422
H	2.58996	1.31930	1.31229
Au	1.85932	2.64410	-3.68158
Cl	2.84054	4.61518	-4.26074

R = *t*-Bu

P	0.45708	0.48299	0.20271
C	0.28404	0.16420	-1.41281
P	-1.23204	-0.24835	-2.23122
Ge	1.17831	0.20543	-3.23621
Cl	2.17840	-1.73052	-3.37595
O	-0.65321	-0.33154	-3.67367
C	-2.43186	1.08283	-2.12275
H	-1.94555	1.99235	-2.47916
H	-3.29723	0.87128	-2.75249
H	-2.74935	1.22835	-1.08929
Au	1.97763	2.11428	-4.32026
Cl	2.70465	4.03728	-5.30559
C	2.22947	0.93261	0.56543
C	3.17697	0.87428	-0.61733
C	2.16937	2.35285	1.13928
C	2.67322	-0.04332	1.66050
H	3.22257	-0.12545	-1.05552
H	2.90272	1.58971	-1.39689
H	4.18907	1.13322	-0.28919
H	1.47276	2.42658	1.97932
H	3.16145	2.63596	1.50490
H	1.87230	3.08144	0.38128
H	3.66407	0.24766	2.02323
H	1.99125	-0.03740	2.51553
H	2.74207	-1.06703	1.28428
C	-2.03088	-1.82461	-1.75616
C	-3.11460	-2.12749	-2.79465
C	-0.97186	-2.92648	-1.77701
C	-2.63618	-1.69264	-0.35951
H	-3.92528	-1.39592	-2.77495
H	-2.70282	-2.16629	-3.80495
H	-3.55037	-3.10491	-2.56988
H	-0.18476	-2.74784	-1.04268
H	-1.45383	-3.87763	-1.53467
H	-0.50367	-3.02092	-2.75772
H	-3.04902	-2.66061	-0.06247
H	-1.88880	-1.40983	0.38650
H	-3.45033	-0.96544	-0.32793

R = Ph

P	0.62761	0.41303	0.27365
C	0.19429	0.19365	-1.31228
P	-1.42039	-0.11123	-1.94425
Ge	0.81788	0.52369	-3.20235
Cl	1.57788	-1.43137	-3.83251
O	-1.10581	0.18352	-3.43540
C	-2.67855	0.99518	-1.31140
H	-2.34176	2.02205	-1.46005
H	-3.61277	0.82803	-1.85055
H	-2.83639	0.81827	-0.24580
Au	1.71134	2.50403	-4.05217
Cl	2.57899	4.46548	-4.82306
C	2.38957	0.77498	0.27422
C	2.86074	1.48800	1.38120
C	3.28976	0.38950	-0.72320
C	4.19434	1.85129	1.46564
H	2.17118	1.77016	2.17095
C	4.62294	0.74429	-0.62972
H	2.95641	-0.21809	-1.55703
C	5.07374	1.48257	0.45811
H	4.54841	2.41975	2.31758
H	5.31475	0.44491	-1.40809
H	6.11867	1.76337	0.52289
C	-2.01174	-1.78864	-1.74483
C	-1.96046	-2.65540	-2.83318
C	-2.49306	-2.23517	-0.51424
C	-2.40085	-3.96228	-2.68945
H	-1.57455	-2.30319	-3.78209
C	-2.92678	-3.54220	-0.37845
H	-2.52136	-1.57175	0.34390
C	-2.88263	-4.40422	-1.46685
H	-2.36293	-4.63703	-3.53640
H	-3.29912	-3.89052	0.57769
H	-3.22366	-5.42734	-1.35790

R = Mes

P	0.75342	0.17090	0.74379
C	0.16766	0.07072	-0.80476
P	-1.43794	-0.14558	-1.52489
Ge	0.82947	0.79053	-2.56032
Cl	1.55273	-0.99910	-3.59963
O	-1.09211	0.55572	-2.86953

C	-2.75611	0.79317	-0.74327
H	-2.40912	1.81598	-0.58960
H	-3.61192	0.79785	-1.42211
H	-3.06000	0.35709	0.20776
Au	1.76111	2.89733	-2.94533
Cl	2.65864	4.98247	-3.19561
C	2.46709	0.72891	0.48513
C	2.77674	2.08087	0.69535
C	3.47020	-0.19190	0.15173
C	4.08788	2.49928	0.52350
C	4.76994	0.27395	-0.00207
C	5.09718	1.61398	0.16422
H	4.32357	3.55022	0.66048
H	5.55112	-0.43486	-0.26040
C	-2.00383	-1.84263	-1.74679
C	-2.39818	-2.32434	-3.01554
C	-2.08288	-2.68215	-0.61964
C	-2.84030	-3.63787	-3.10679
C	-2.53830	-3.98453	-0.77620
C	-2.91262	-4.48787	-2.01162
H	-3.14822	-4.00663	-4.08016
H	-2.60101	-4.62140	0.10088
C	-2.38192	-1.52050	-4.28398
H	-2.89745	-0.56508	-4.18513
H	-1.36191	-1.29267	-4.59700
H	-2.86583	-2.08911	-5.07829
C	-1.66729	-2.26575	0.76016
H	-0.60126	-2.45286	0.91595
H	-1.83280	-1.21081	0.97959
H	-2.21387	-2.84214	1.50783
C	-3.36755	-5.90475	-2.16761
H	-2.54363	-6.53452	-2.51712
H	-3.72016	-6.31810	-1.22168
H	-4.17099	-5.98579	-2.90213
C	3.17283	-1.65024	-0.03818
H	2.54296	-2.04280	0.76709
H	2.64720	-1.83274	-0.97968
H	4.09474	-2.23293	-0.05204
C	1.71341	3.07606	1.04895
H	1.15274	3.37707	0.15798
H	0.99592	2.66932	1.77054
H	2.14842	3.97829	1.48004
C	6.49517	2.10214	-0.05649
H	6.56934	2.62496	-1.01470
H	6.79720	2.80792	0.72034
H	7.20941	1.27766	-0.06963

R = Mes*

P	-0.40276	2.04360	1.20992
C	0.14869	0.66014	0.46998
P	1.73012	-0.15255	0.31009
Cl	-1.06856	0.26967	-2.66951
O	1.32666	-0.96564	-0.96363
C	1.85038	-1.42500	1.58635
H	2.67398	-2.08649	1.31094
H	2.00139	-1.05057	2.59316
H	0.91615	-1.99087	1.54515
C	3.43360	0.47080	0.11550
C	4.02030	0.41602	-1.18248
C	4.29497	0.48638	1.24475
C	5.33845	-0.00903	-1.27006
C	5.61425	0.08233	1.06877
C	6.13615	-0.27250	-0.16466
H	5.76666	-0.12355	-2.25710
H	6.25825	0.05683	1.93678
C	-2.20077	1.95018	0.85582
C	-2.74180	2.71264	-0.20378
C	-3.01448	1.01689	1.54584
C	-3.97201	2.31676	-0.72228
C	-4.22398	0.66857	0.95917
C	-4.69325	1.24771	-0.21271
H	-4.37589	2.85564	-1.56441
H	-4.83257	-0.08481	1.43936
C	-2.09124	4.00919	-0.72687
C	-2.70831	0.46825	2.95820
C	-5.97929	0.73555	-0.85223
C	3.97196	1.09721	2.62350
C	3.37940	0.92711	-2.48678
C	7.54197	-0.83099	-0.34513
C	-4.01922	0.19309	3.71068
H	-3.78703	-0.03239	4.75429
H	-4.68864	1.05618	3.68906
H	-4.55366	-0.67106	3.31353
C	-1.95490	1.50395	3.79583
H	-0.93495	1.69036	3.43659
H	-2.47866	2.46252	3.81824
H	-1.85136	1.14176	4.82218
C	-1.91650	-0.84141	2.94270
H	-2.42947	-1.61098	2.36231
H	-0.92250	-0.70512	2.51800
H	-1.80044	-1.21266	3.96550
C	-7.14070	0.85705	0.14125
H	-8.06318	0.48782	-0.31522
H	-6.96483	0.27406	1.04726
H	-7.29905	1.89810	0.43477
C	-6.34697	1.51144	-2.11422
H	-7.25723	1.08946	-2.54671
H	-6.54145	2.56685	-1.90474
H	-5.56175	1.44758	-2.87154
C	-5.78435	-0.73791	-1.23142
H	-4.96839	-0.85252	-1.94880
H	-5.54860	-1.35746	-0.36416
H	-6.69710	-1.13227	-1.68672
C	-2.99015	4.72832	-1.73576
H	-3.97280	4.96074	-1.31940
H	-2.51778	5.67300	-2.01489
H	-3.12739	4.14862	-2.65150
C	-1.89542	4.96704	0.45722
H	-2.84788	5.17512	0.95047
H	-1.20989	4.56433	1.20651
H	-1.47785	5.91478	0.10545
C	-0.74867	3.78272	-1.42525
H	0.02893	3.43386	-0.74046
H	-0.83418	3.04999	-2.22829
H	-0.39342	4.72740	-1.84764
C	2.56583	1.66854	2.74785
H	2.33897	2.35203	1.92710
H	1.77940	0.91662	2.79810
H	2.49169	2.24431	3.67349
C	4.91641	2.30840	2.77136
H	4.71269	2.81371	3.71933
H	5.96689	2.01691	2.76421
H	4.75906	3.02549	1.96241
C	4.24915	0.13680	3.78419
H	5.28885	-0.19435	3.79802
H	4.05437	0.64307	4.73318
H	3.62300	-0.75561	3.74846
C	2.18072	1.83247	-2.22167
H	1.83593	2.26118	-3.16542
H	1.32536	1.31453	-1.79957
H	2.45641	2.65784	-1.55965
C	3.01737	-0.20872	-3.44798

H	2.25112	-0.85923	-3.03417
H	2.64632	0.21053	-4.38705
H	3.89791	-0.81433	-3.67844
C	4.41279	1.82811	-3.19328
H	4.78476	2.61021	-2.52696
H	5.26786	1.27516	-3.58330
H	3.92801	2.31085	-4.04532
C	8.24751	-1.06370	0.98817
H	8.40718	-0.13103	1.53538
H	7.68770	-1.74945	1.62919
H	9.22944	-1.50699	0.80775
C	8.38362	0.14721	-1.17356
H	7.95779	0.31209	-2.16510
H	8.46166	1.11672	-0.67549
H	9.39387	-0.24896	-1.30687
C	7.44879	-2.17211	-1.08290
H	6.86517	-2.89589	-0.50897
H	6.97813	-2.06418	-2.06199
H	8.44899	-2.58616	-1.23570
Au	-1.86015	-2.64298	-0.25873
Cl	-3.10660	-4.46897	0.33356
Ge	-0.61005	-0.73479	-0.78550

Coordination through O atom

R= H

P	0.53475	0.19662	0.68169
C	0.29462	0.30392	-0.95361
P	-1.21545	0.39364	-1.82151
Ge	1.45099	0.64563	-2.65831
Cl	1.35346	-1.47055	-3.32404
O	-0.68463	1.03087	-3.13510
C	-2.55364	1.31998	-1.08988
H	-2.22277	2.34179	-0.89975
H	-3.40697	1.32970	-1.77122
H	-2.83899	0.84389	-0.14803
Au	-1.97784	1.37990	-4.75638
Cl	-3.39242	1.75637	-6.42717
H	-1.72753	-0.89230	-2.07131
H	1.96549	0.24746	0.68071

R = Me

P	0.52263	0.10077	0.65034
C	0.28136	0.28298	-0.97553
P	-1.25171	0.32322	-1.80710
Ge	1.36892	0.77785	-2.68421
Cl	1.57790	-1.32707	-3.40137
O	-0.73630	0.93358	-3.15329
C	-2.51558	1.36364	-1.08401
H	-2.12167	2.37362	-0.96656
H	-3.39125	1.38767	-1.73631
H	-2.79498	0.96672	-0.10523
C	-1.95091	-1.29972	-2.07454
H	-2.27089	-1.72351	-1.12025
H	-2.80388	-1.22174	-2.75262
H	-1.18136	-1.93159	-2.52065
C	2.34743	0.14200	0.84793
H	2.71772	-0.88781	0.82805
H	2.84643	0.70886	0.06055
H	2.59835	0.56399	1.82295
Au	-2.01891	1.30818	-4.77156
Cl	-3.42593	1.70731	-6.44561

R = *t*-Bu

P	0.62925	0.02623	0.35813
C	0.40201	0.07879	-1.28241
P	-1.17812	0.01789	-2.04950
Ge	1.40237	0.49369	-3.07767
Cl	2.01487	-1.60591	-3.53652
O	-0.69824	0.23594	-3.52688
C	-2.23190	1.38381	-1.55109
H	-1.67867	2.30920	-1.71785
H	-3.14482	1.40409	-2.14813
H	-2.47518	1.29984	-0.49058
Au	-1.86238	0.92942	-5.12991
Cl	-3.10679	1.67894	-6.81390
C	-2.10786	-1.55357	-1.88017
C	-3.34895	-1.50220	-2.77468
C	-1.19951	-2.70212	-2.31704
C	-2.51852	-1.72733	-0.41755
H	-4.05150	-0.72365	-2.47102
H	-3.09487	-1.34585	-3.82502
H	-3.86837	-2.46143	-2.69550
H	-0.30321	-2.77368	-1.69915
H	-1.75673	-3.63826	-2.22299
H	-0.88279	-2.59631	-3.35523
H	-3.01472	-2.69584	-0.31124
H	-1.65961	-1.71953	0.25782
H	-3.22438	-0.96050	-0.09103
C	2.45794	0.15307	0.72048
C	3.32863	0.52401	-0.46508
C	2.58481	1.20234	1.82845
C	2.85649	-1.22168	1.26789
H	3.29818	-0.24413	-1.24281
H	3.04089	1.48680	-0.89609
H	4.37315	0.61185	-0.14698
H	1.94390	0.96963	2.68324
H	3.61879	1.23537	2.18584
H	2.32211	2.20140	1.47095
H	3.88976	-1.18403	1.62850
H	2.22485	-1.52533	2.10763
H	2.79617	-1.99275	0.49630

R = Ph

P	0.48052	0.41728	0.44060
C	0.37166	0.15189	-1.19373
P	-1.10678	0.14905	-2.12225
Ge	1.60501	0.31884	-2.85383
Cl	1.84459	-1.86188	-3.29095
O	-0.47243	0.40111	-3.52678
C	-2.26724	1.44764	-1.70209
H	-1.75653	2.40796	-1.78541
H	-3.11518	1.41767	-2.38887
H	-2.61767	1.31640	-0.67625
Au	-1.54926	0.86142	-5.26875
Cl	-2.70532	1.36062	-7.10148
C	-1.98928	-1.40687	-2.09731
C	-2.79871	-1.73145	-1.00851
C	-1.84578	-2.30153	-3.15424
C	-3.46657	-2.94294	-0.98419
H	-2.90527	-1.04762	-0.17301
C	-2.52275	-3.51078	-3.12401
H	-1.20776	-2.05297	-3.99299
C	-3.33055	-3.83055	-2.04377
H	-4.09569	-3.19502	-0.13875
H	-2.41614	-4.20371	-3.94994
H	-3.85781	-4.77742	-2.02465
C	2.24903	0.35403	0.80372
C	2.69000	1.08842	1.90789
C	3.17444	-0.39308	0.06890
C	4.03419	1.11621	2.24136

H	1.97368	1.65020	2.49966
C	4.51569	-0.37168	0.41174
H	2.83766	-1.02864	-0.74390
C	4.94747	0.38899	1.49103
H	4.36965	1.70069	3.09022
H	5.22603	-0.95795	-0.15962
H	5.99851	0.40321	1.75597

R = Mes

P	0.35943	0.09013	0.80258
C	0.18950	0.05515	-0.84422
P	-1.18726	0.08340	-1.93503
Ge	1.59580	0.53234	-2.27744
Cl	2.07316	-1.57113	-2.88438
O	-0.40062	0.57825	-3.19505
C	-2.41727	1.33356	-1.54111
H	-1.90956	2.29934	-1.51352
H	-3.17648	1.35081	-2.32564
H	-2.88501	1.14137	-0.57715
Au	-1.09234	1.89902	-4.67903
Cl	-1.81710	3.31154	-6.23715
C	-2.00033	-1.50775	-2.19564
C	-2.70631	-2.06663	-1.11007
C	-1.94314	-2.20095	-3.42712
C	-3.31847	-3.30263	-1.26205
C	-2.58642	-3.43007	-3.51515
C	-3.26935	-4.00614	-2.45471
H	-3.85256	-3.72354	-0.41567
H	-2.54383	-3.95861	-4.46202
C	2.16934	0.26153	1.00758
C	2.72662	1.53484	1.19585
C	2.97410	-0.88559	1.07911
C	4.09306	1.64069	1.42034
C	4.33524	-0.72969	1.30735
C	4.91577	0.52217	1.47235
H	4.52562	2.62663	1.56350
H	4.95988	-1.61638	1.36378
C	1.87962	2.77133	1.14036
H	1.58072	3.00260	0.11303
H	0.96015	2.66011	1.72460
H	2.42143	3.63416	1.52940
C	2.39408	-2.25763	0.91043
H	1.49464	-2.39301	1.52027
H	2.11050	-2.43935	-0.13023
H	3.11435	-3.02266	1.20277
C	6.39187	0.66249	1.68369
H	6.91330	0.73701	0.72425
H	6.62941	1.56131	2.25528
H	6.80273	-0.20001	2.21144
C	-2.88114	-1.39081	0.21985
H	-2.02770	-0.78465	0.52616
H	-3.76703	-0.74836	0.21646
H	-3.03218	-2.13765	1.00010
C	-1.25035	-1.71750	-4.66674
H	-1.65024	-0.76316	-5.01710
H	-0.18084	-1.58136	-4.50806
H	-1.38663	-2.44726	-5.46462
C	-3.90872	-5.35196	-2.59015
H	-4.70749	-5.49052	-1.86011
H	-4.32376	-5.49535	-3.58937
H	-3.17124	-6.14408	-2.42756

R = Mes*

P	1.97406	2.05802	-0.37323
C	1.00231	0.71467	-0.31627
P	-0.73661	0.48297	-0.47657
C	3.64317	1.27638	-0.30576
C	4.36043	1.29392	0.92138
C	4.13441	0.51769	-1.38753
C	5.37202	0.36303	1.09183
C	5.14003	-0.41818	-1.12714
C	5.73239	-0.55889	0.11213
H	5.89790	0.33440	2.03484
H	5.47461	-1.04646	-1.94107
C	3.78711	0.71273	-2.87979
C	4.10994	2.33359	2.03045
C	6.77745	-1.62589	0.41902
C	2.75676	2.14280	2.72067
H	1.91462	2.35994	2.05949
H	2.63525	1.11930	3.07909
H	2.67850	2.82671	3.57102
C	4.19611	3.74328	1.42952
H	5.17307	3.90952	0.96903
H	3.43084	3.92221	0.67129
H	4.05761	4.49111	2.21539
C	5.17407	2.25985	3.12871
H	5.12584	1.32290	3.68842
H	6.18489	2.37934	2.73168
H	4.99955	3.07106	3.83913
C	3.18542	-0.55585	-3.48988
H	2.22649	-0.80244	-3.02881
H	3.02043	-0.41426	-4.56182
H	3.84048	-1.41976	-3.36461
C	2.85666	1.88408	-3.18670
H	1.82339	1.69105	-2.89755
H	3.18992	2.81008	-2.70885
H	2.85433	2.06189	-4.26590
C	5.10559	1.03437	-3.60944
H	5.56532	1.93762	-3.20075
H	5.83268	0.22599	-3.53446
H	4.90516	1.20361	-4.67088
C	7.02239	-2.55413	-0.76748
H	7.42566	-2.01770	-1.63048
H	7.75181	-3.31786	-0.48759
H	6.10818	-3.06784	-1.07529
C	6.28895	-2.47415	1.59973
H	6.14231	-1.87082	2.49764
H	5.33741	-2.95815	1.36846
H	7.02283	-3.25057	1.83326
C	8.10579	-0.95592	0.78931
H	8.00491	-0.31100	1.66454
H	8.85798	-1.71529	1.02056
H	8.48058	-0.34594	-0.03652
C	-2.17944	1.34852	0.21681
C	-2.78080	0.78557	1.37945
C	-2.98590	2.14171	-0.64102
C	-4.16388	0.68601	1.39788
C	-4.36583	2.00705	-0.54576
C	-4.97990	1.19205	0.39443
H	-4.62436	0.18773	2.24141
H	-4.98234	2.57082	-1.23266
C	-2.04562	0.43456	2.68601
C	-2.09506	-1.05612	3.02726
C	-2.76349	1.20762	3.81149
H	-1.49504	-1.64737	2.33753
H	-1.69992	-1.21211	4.03477
H	-3.11786	-1.43863	3.00444
H	-2.80730	2.27748	3.59303
H	-3.78014	0.85447	3.98616
H	-2.20542	1.07273	4.74149
C	-2.47599	3.29007	-1.53404
C	-3.12425	4.57263	-0.97203

C	-2.90417	3.15109	-2.99769
H	-2.86376	4.71252	0.07955
H	-2.75958	5.43804	-1.53220
H	-4.21133	4.55515	-1.05102
H	-2.47573	2.27148	-3.47830
H	-3.98915	3.07970	-3.09249
H	-2.58209	4.03106	-3.56087
C	-6.47792	0.92221	0.41224
C	-7.09524	1.52928	1.67794
C	-6.70285	-0.59511	0.41088
H	-6.94745	2.61189	1.70882
H	-8.17032	1.33152	1.69981
H	-6.66104	1.10370	2.58475
H	-6.25949	-1.06592	-0.46908
H	-6.26338	-1.07232	1.28858
H	-7.77466	-0.81119	0.41449
Cl	1.95984	-1.53380	1.92001
O	-0.75640	-0.99162	0.08722
Au	-2.33950	-2.30612	-0.36900
Cl	-4.02233	-3.68260	-0.86980
C	-0.97300	3.53106	-1.44100
H	-0.65004	3.63827	-0.40329
H	-0.36270	2.75792	-1.90446
H	-0.72443	4.46131	-1.95758
C	-0.60577	0.93041	2.70220
H	0.04918	0.38038	2.03115
H	-0.55261	1.99545	2.46111
H	-0.18958	0.79300	3.70308
C	-7.18454	1.51645	-0.80321
H	-7.12266	2.60794	-0.82306
H	-6.77400	1.12775	-1.73845
H	-8.24381	1.25167	-0.77215
Ge	1.38789	-1.33528	-0.22810
C	-1.07431	0.25547	-2.23998
H	-2.11627	-0.04051	-2.37152
H	-0.84964	1.12580	-2.84887
H	-0.43446	-0.57249	-2.55545

Coordination through P(sp^2) atom

R= H

P	0.48645	0.28047	0.52064
C	0.19575	0.19747	-1.08372
P	-1.31608	0.12388	-1.96194
Ge	1.19014	0.43490	-2.95798
Cl	1.28520	-1.77261	-3.30929
O	-0.83114	0.58704	-3.34030
C	-2.68587	1.08244	-1.33588
H	-2.39024	2.12921	-1.25611
H	-3.52907	0.99154	-2.02403
H	-2.97613	0.70636	-0.35150
Au	-0.87177	0.28009	2.25502
Cl	-2.32181	0.29575	3.98409
H	1.88721	0.38321	0.66781
H	-1.77015	-1.20903	-1.97871

R = Me

P	0.53398	0.31206	0.48794
C	0.18343	0.23251	-1.10235
P	-1.36686	0.04177	-1.89920
Ge	1.07383	0.46540	-3.01565
Cl	1.47308	-1.73602	-3.26403
O	-0.93039	0.36822	-3.34117
C	-2.65327	1.15417	-1.33805
H	-2.29524	2.18146	-1.41206
H	-3.53518	1.03269	-1.97095
H	-2.91485	0.93337	-0.30069
C	-1.98484	-1.63003	-1.74602
H	-2.25089	-1.83755	-0.70738
H	-2.86393	-1.74983	-2.38310
H	-1.19928	-2.31227	-2.07430
C	2.31121	0.49624	0.76696
H	2.67511	-0.38824	1.29583
H	2.84379	0.60615	-0.17710
H	2.48628	1.36318	1.40755
Au	-0.87137	0.20406	2.19106
Cl	-2.37217	0.09133	3.88062

R = *t*-Bu

P	0.54604	0.35161	0.41494
C	0.26940	0.11134	-1.17959
P	-1.26130	-0.15364	-2.01453
Ge	1.18620	0.41614	-3.08006
Cl	2.03564	-1.64799	-3.34944
O	-0.75329	-0.01473	-3.46816
C	-2.45638	1.13564	-1.64046
H	-2.00109	2.09423	-1.89328
H	-3.35912	1.00211	-2.23873
H	-2.71060	1.12870	-0.57915
Au	-0.95046	0.41985	2.05409
Cl	-2.51757	0.51887	3.69021
C	2.32394	0.60682	0.85668
C	2.40432	1.90714	1.65973
C	2.71620	-0.57747	1.74643
C	3.21614	0.67277	-0.37196
H	2.14177	2.77589	1.05148
H	1.74648	1.88826	2.53208
H	3.43011	2.04095	2.01560
H	2.68997	-1.52068	1.19622
H	3.73769	-0.42419	2.10761
H	2.06314	-0.66560	2.61832
H	4.25340	0.81018	-0.05142
H	3.17049	-0.24577	-0.96079
H	2.95908	1.51749	-1.01581
C	-2.06012	-1.78584	-1.76035
C	-1.00059	-2.88123	-1.86137
C	-2.75202	-1.84190	-0.40100
C	-3.08237	-1.95125	-2.89002
H	-0.46948	-2.85115	-2.81320
H	-0.26108	-2.80422	-1.06257
H	-1.49597	-3.85225	-1.77281
H	-3.56321	-1.11688	-0.31198
H	-3.18548	-2.83740	-0.27084
H	-2.05645	-1.67968	0.42600
H	-3.53619	-2.94227	-2.80211
H	-3.88910	-1.21682	-2.83230
H	-2.61186	-1.87701	-3.87161

R = Ph

P	0.52279	0.47564	0.28625
C	0.33261	0.03626	-1.27751
P	-1.15562	-0.26496	-2.15774
Ge	1.37948	-0.01814	-3.10958
Cl	1.95197	-2.19501	-3.01999
O	-0.57769	-0.29953	-3.58657
C	-2.38784	1.02430	-1.98332
H	-1.93195	1.97825	-2.25197
H	-3.22207	0.81180	-2.65475
H	-2.75055	1.07780	-0.95491
Au	-1.08396	0.91011	1.75007
Cl	-2.81004	1.32077	3.15942

C	2.24009	0.61272	0.74801
C	3.25120	-0.03385	0.03235
C	2.56059	1.38317	1.86751
C	4.56946	0.11550	0.42422
H	3.00302	-0.68065	-0.80194
C	3.88320	1.53248	2.24800
H	1.77050	1.86639	2.43327
C	4.88623	0.90201	1.52506
H	5.35279	-0.39232	-0.12596
H	4.13097	2.13736	3.11215
H	5.92113	1.01394	1.82769
C	-1.92850	-1.82281	-1.73335
C	-2.72499	-1.94451	-0.59445
C	-1.70460	-2.92549	-2.55397
C	-3.29735	-3.16597	-0.28407
H	-2.89603	-1.09673	0.06086
C	-2.28534	-4.14369	-2.23733
H	-1.07483	-2.82342	-3.42920
C	-3.07902	-4.26364	-1.10645
H	-3.91402	-3.26007	0.60187
H	-2.11249	-5.00217	-2.87557
H	-3.52950	-5.21863	-0.86092

R = Mes

P	0.47052	0.52779	0.81583
C	0.18058	-0.06827	-0.67895
P	-1.23834	-0.25771	-1.70381
Ge	1.38295	0.11908	-2.39667
Cl	1.70810	-2.09291	-2.71613
O	-0.55263	0.16279	-3.02196
C	-2.58726	0.87788	-1.35806
H	-2.19098	1.89314	-1.31101
H	-3.31098	0.80489	-2.17295
H	-3.08467	0.63678	-0.41821
Au	-0.94925	1.11041	2.41513
Cl	-2.42510	1.72455	4.02204
C	2.24456	0.73854	1.04325
C	3.06388	-0.38568	1.23214
C	2.77983	2.03652	1.06536
C	4.42275	-0.17951	1.43212
C	4.14445	2.18514	1.26426
C	4.98218	1.09185	1.45191
H	5.06340	-1.04349	1.57978
H	4.56549	3.18592	1.27051
C	-1.92997	-1.92495	-1.77632
C	-2.28082	-2.59050	-0.58594
C	-2.17196	-2.54230	-3.02370
C	-2.84317	-3.85764	-0.65856
C	-2.73726	-3.81177	-3.02935
C	-3.07165	-4.49327	-1.86863
H	-3.11318	-4.35784	0.26634
H	-2.92690	-4.28196	-3.98918
C	1.91847	3.24423	0.85125
H	1.48043	3.24702	-0.15154
H	1.09142	3.28288	1.56738
H	2.49934	4.16002	0.96265
C	2.51896	-1.78255	1.22860
H	1.63712	-1.87164	1.87058
H	2.22412	-2.09533	0.22301
H	3.26790	-2.48553	1.59433
C	6.44635	1.28255	1.69966
H	6.63956	1.43358	2.76638
H	7.02089	0.41091	1.38255
H	6.82843	2.15830	1.17221
C	-2.05858	-2.01837	0.78114
H	-1.01622	-2.13561	1.08827
H	-2.29395	-0.95585	0.85793
H	-2.67840	-2.53580	1.51374
C	-1.86661	-1.93327	-4.36236
H	-2.26328	-0.92379	-4.47080
H	-0.79003	-1.86539	-4.52534
H	-2.29604	-2.55567	-5.14796
C	-3.64584	-5.87405	-1.92281
H	-4.25880	-6.01598	-2.81459
H	-2.84542	-6.61984	-1.95495
H	-4.25726	-6.08702	-1.04452

R = Mes*

P	-1.13410	0.83689	0.03358
C	-0.20865	-0.44175	0.47114
P	1.46337	-0.91389	0.80891
Cl	-1.31075	-3.20945	-1.16339
O	1.25729	-2.43828	0.60634
C	3.08576	-0.48117	0.08569
C	3.62565	-1.39673	-0.86177
C	3.98386	0.35161	0.80401
C	4.97434	-1.70669	-0.75963
C	5.32582	-0.01348	0.84232
C	5.83263	-1.09989	0.14738
H	5.37288	-2.45481	-1.43280
H	5.99975	0.59393	1.43046
C	-2.85746	0.25013	0.09506
C	-3.51498	-0.16217	-1.09474
C	-3.43583	-0.02642	1.35838
C	-4.59603	-1.02615	-0.96281
C	-4.50299	-0.91841	1.39500
C	-5.06643	-1.47705	0.26129
H	-5.07689	-1.38470	-1.85735
H	-4.91434	-1.18250	2.36078
C	-3.17484	0.35868	-2.50553
C	-3.09304	0.63159	2.71423
C	-6.17869	-2.51256	0.38881
C	3.65587	1.73752	1.39157
C	2.88619	-1.96741	-2.08481
C	7.27685	-1.56886	0.27316
C	-4.39245	1.24604	3.26916
H	-4.18270	1.73965	4.22166
H	-4.79331	1.99358	2.58049
H	-5.16998	0.50380	3.44765
C	-2.09663	1.78709	2.66177
H	-1.07173	1.46261	2.47849
H	-2.36892	2.53673	1.91455
H	-2.09138	2.28997	3.63277
C	-2.57158	-0.41099	3.70728
H	-3.28347	-1.22474	3.85625
H	-1.63645	-0.85324	3.35719
H	-2.38757	0.05701	4.67855
C	-7.36808	-1.90599	1.14208
H	-8.16695	-2.64622	1.23978
H	-7.09168	-1.58224	2.14759
H	-7.76952	-1.04065	0.60890
C	-6.67185	-3.00463	-0.96937
H	-7.44865	-3.75864	-0.82176
H	-7.10756	-2.19692	-1.56315
H	-5.86880	-3.46599	-1.54912
C	-5.64073	-3.71802	1.16973
H	-4.78954	-4.16852	0.65390
H	-5.31327	-3.43657	2.17280
H	-6.42093	-4.47731	1.27304
C	-4.06046	-0.27730	-3.58194
H	-5.12147	-0.07229	-3.42367
H	-3.78957	0.15217	-4.54873

H	-3.91499	-1.35827	-3.64846
C	-3.45570	1.86724	-2.53611
H	-4.50366	2.07002	-2.30165
H	-2.84041	2.41873	-1.82167
H	-3.24331	2.26841	-3.53074
C	-1.73220	0.07916	-2.93435
H	-0.99447	0.66055	-2.37711
H	-1.48429	-0.97738	-2.82819
H	-1.60728	0.36692	-3.98202
C	2.23235	2.20044	1.11198
H	2.01078	2.13549	0.04466
H	1.46059	1.65483	1.65211
H	2.12395	3.24934	1.39565
C	4.55213	2.74141	0.63543
H	4.31087	3.75620	0.96187
H	5.61332	2.57200	0.81937
H	4.37942	2.68418	-0.44136
C	3.98215	1.85324	2.88342
H	5.03225	1.62948	3.08184
H	3.79475	2.87594	3.22068
H	3.38251	1.18318	3.49965
C	1.55212	-1.27763	-2.34684
H	1.16975	-1.59670	-3.31935
H	0.78329	-1.54065	-1.62584
H	1.66649	-0.19050	-2.37480
C	2.69755	-3.48380	-1.99240
H	2.05888	-3.75171	-1.15336
H	2.23402	-3.85287	-2.91122
H	3.65841	-3.99187	-1.87575
C	3.75457	-1.66066	-3.32173
H	3.96007	-0.59076	-3.40712
H	4.70736	-2.19086	-3.31750
H	3.21206	-1.97392	-4.21720
C	8.04936	-0.78622	1.33161
H	8.14101	0.27163	1.07251
H	7.57885	-0.86137	2.31526
H	9.06107	-1.18959	1.41664
C	7.99349	-1.39960	-1.07189
H	7.51882	-1.98456	-1.86195
H	7.99715	-0.35271	-1.38488
H	9.03128	-1.73355	-0.98783
C	7.28224	-3.05045	0.66851
H	6.78943	-3.20050	1.63218
H	6.76721	-3.66805	-0.06980
H	8.31033	-3.41328	0.75139
Au	-0.57822	2.91623	-0.53681
Cl	-0.01672	5.05320	-1.07021
Ge	-0.75737	-2.46944	0.88680
C	1.66408	-0.75022	2.60225
H	2.61618	-1.20583	2.87933
H	1.61017	0.26245	2.98716
H	0.85511	-1.34095	3.03908

Coordination through π(C=P) bond

R= H

P	0.55447	-0.17113	0.65406
C	0.21775	0.55260	-0.87169
P	-1.25944	0.51375	-1.83581
Ge	1.26200	0.99932	-2.69697
Cl	1.41374	-1.18312	-3.21662
O	-0.74729	1.14365	-3.13514
C	-2.72725	1.32371	-1.21513
H	-2.51493	2.37693	-1.02603
H	-3.51919	1.24019	-1.96289
H	-3.04860	0.84206	-0.28880
Au	0.28482	2.19215	0.51814
Cl	0.20773	4.26941	1.37839
H	1.98138	-0.13748	0.57559
H	-1.63316	-0.83295	-2.01257

R = Me

P	0.55636	-0.08732	0.63586
C	0.15769	0.67360	-0.85298
P	-1.32052	0.50588	-1.81146
Ge	1.17101	1.16266	-2.67668
Cl	1.68938	-1.00423	-3.08930
O	-0.79574	1.01778	-3.16637
C	-2.74101	1.46208	-1.28042
H	-2.46916	2.51645	-1.21805
H	-3.54200	1.34076	-2.01360
H	-3.08630	1.11951	-0.30310
C	-1.83597	-1.20698	-1.90612
H	-2.17053	-1.55597	-0.92698
H	-2.64903	-1.30070	-2.62914
H	-0.98203	-1.79819	-2.24065
C	2.38950	-0.12843	0.64199
H	2.68307	-1.10128	0.23351
H	2.84746	0.65615	0.04052
H	2.74802	-0.06879	1.67132
Au	0.10242	2.26098	0.59602
Cl	-0.09510	4.26590	1.60757

R = *t*-Bu

P	0.67708	-0.12608	0.44139
C	0.09892	0.68782	-0.96299
P	-1.44992	0.38463	-1.79752
Ge	0.79806	1.58215	-2.79825
Cl	1.87746	-0.23716	-3.59585
O	-1.06826	0.91094	-3.19850
C	-2.81285	1.36485	-1.14876
H	-2.52233	2.41615	-1.17861
H	-3.69039	1.22793	-1.78363
H	-3.05318	1.09176	-0.12119
Au	-0.09969	2.12230	0.64456
Cl	-0.49272	3.97160	1.87820
C	-2.00642	-1.36350	-1.87968
C	-3.10883	-1.43509	-2.94118
C	-0.83780	-2.25051	-2.30483
C	-2.54035	-1.80500	-0.51734
H	-3.99637	-0.86398	-2.66032
H	-2.75536	-1.07714	-3.90965
H	-3.41461	-2.47893	-3.05484
H	-0.04677	-2.27230	-1.55340
H	-1.20629	-3.27251	-2.43162
H	-0.39746	-1.92336	-3.24733
H	-2.81275	-2.86274	-0.57374
H	-1.79035	-1.70108	0.27102
H	-3.43480	-1.25198	-0.22351
C	2.53556	0.04235	0.54805
C	3.05969	-1.30169	0.02627
C	3.16100	1.19097	-0.22346
C	2.84183	0.17231	2.04177
H	2.61336	-2.14834	0.55668
H	2.87054	-1.41683	-1.04327
H	4.14166	-1.34892	0.18655
H	2.75922	2.15907	0.08469
H	4.24022	1.20587	-0.03685
H	3.02894	1.07543	-1.30074
H	3.92345	0.11300	2.19640
H	2.49626	1.12838	2.44290
H	2.37748	-0.62831	2.62449

R = Ph

P	0.38564	0.35850	0.52151
C	0.20389	0.64879	-1.16499
P	-1.28158	0.42048	-2.10451
Ge	1.22173	0.93348	-3.04070
Cl	1.71680	-1.23327	-3.45505
O	-0.75251	0.77925	-3.50514
C	-2.68315	1.45175	-1.66659
H	-2.39500	2.50198	-1.73058
H	-3.49668	1.24746	-2.36601
H	-3.01604	1.22910	-0.65112
Au	0.25778	2.61948	-0.25751
Cl	0.26224	4.84483	0.11254
C	-1.85805	-1.27681	-2.04039
C	-2.39458	-1.80199	-0.86528
C	-1.77830	-2.06343	-3.18524
C	-2.85252	-3.10790	-0.84037
H	-2.44516	-1.20061	0.03689
C	-2.24283	-3.36970	-3.15376
H	-1.34332	-1.65191	-4.08749
C	-2.77839	-3.89023	-1.98581
H	-3.26559	-3.51762	0.07386
H	-2.17804	-3.98361	-4.04432
H	-3.13666	-4.91311	-1.96384
C	2.15907	0.19153	0.84537
C	3.09265	-0.26331	-0.08656
C	2.57210	0.47623	2.14988
C	4.42143	-0.40236	0.27790
H	2.77691	-0.54101	-1.08547
C	3.90460	0.35183	2.50410
H	1.84566	0.80943	2.88486
C	4.82999	-0.08664	1.56703
H	5.14106	-0.76191	-0.44852
H	4.22108	0.59157	3.51252
H	5.87244	-0.19239	1.84510

R = Mes

P	0.31103	0.20241	1.01811
C	-0.08898	0.70739	-0.58341
P	-1.47895	0.50195	-1.69024
Ge	0.98420	1.61474	-2.19122
Cl	1.95599	-0.21249	-3.10023
O	-0.88461	1.26001	-2.89386
C	-3.02501	1.31989	-1.26318
H	-2.83223	2.38579	-1.13019
H	-3.70896	1.18006	-2.10421
H	-3.47715	0.91538	-0.35981
Au	-0.46559	2.43901	0.66893
Cl	-1.11262	4.49783	1.34958
C	-1.85201	-1.23249	-2.03877
C	-2.31633	-2.04768	-0.98789
C	-1.70569	-1.77564	-3.33584
C	-2.54754	-3.39593	-1.22486
C	-1.96513	-3.12994	-3.51088
C	-2.36254	-3.96350	-2.47546
H	-2.89373	-4.01542	-0.40292
H	-1.85027	-3.54611	-4.50680
C	2.13970	0.20792	1.06852
C	2.74642	-0.95602	0.55940
C	2.91596	1.17813	1.71962
C	4.12593	-1.08144	0.63560
C	4.29504	1.00636	1.76450
C	4.92274	-0.10212	1.21412
H	4.58880	-1.97961	0.23756
H	4.89541	1.76363	2.26006
C	2.32541	2.38731	2.37940
H	2.04948	3.15699	1.65190
H	1.42586	2.14652	2.95236
H	3.04404	2.83563	3.06707
C	1.94026	-2.07407	-0.03290
H	1.09343	-2.34753	0.60681
H	1.54190	-1.80390	-1.01359
H	2.55808	-2.96426	-0.15791
C	6.41339	-0.24030	1.24331
H	6.85130	0.33001	2.06419
H	6.71513	-1.28414	1.34909
H	6.85241	0.13265	0.31265
C	-2.65759	-1.53800	0.38345
H	-2.47903	-2.31137	1.13166
H	-2.08767	-0.66292	0.69239
H	-3.72014	-1.27902	0.43516
C	-1.31669	-0.99731	-4.55947
H	-1.91976	-0.09897	-4.68884
H	-0.27623	-0.67404	-4.51427
H	-1.44318	-1.62727	-5.44051
C	-2.57714	-5.42679	-2.70329
H	-2.97370	-5.61999	-3.70165
H	-1.63074	-5.96995	-2.61857
H	-3.26527	-5.84926	-1.96948

R = Mes*

P	0.70257	-0.03970	1.14881
C	0.38736	0.12853	-0.55649
P	-1.09496	-0.10654	-1.60631
O	-0.32700	-0.08970	-2.95016
Cl	2.71768	-1.05434	-2.97746
Au	0.21694	2.14526	0.23079
Cl	-0.25919	4.36846	0.32805
C	2.55461	-0.08254	1.24626
C	3.26581	-1.21756	0.76907
C	3.29851	1.05626	1.64131
C	4.57770	-1.03849	0.35729
C	4.60403	1.17472	1.16197
C	5.24102	0.17928	0.44319
H	5.10932	-1.89215	-0.04276
H	5.14568	2.08187	1.39145
C	2.90542	2.09320	2.71621
C	2.80376	-2.68224	0.90746
C	6.63239	0.34244	-0.15712
C	-2.13871	1.36963	-1.70222
H	-2.68081	1.60805	-0.79266
H	-1.50078	2.21280	-1.97364
H	-2.84735	1.18565	-2.51318
C	3.93416	1.92004	3.85370
H	3.90367	0.90464	4.25712
H	4.95370	2.11889	3.52290
H	3.70108	2.61692	4.66341
C	2.99380	3.53158	2.20107
H	2.28063	3.72411	1.39861
H	2.76969	4.22883	3.01306
H	3.99249	3.76895	1.83018
C	1.54555	1.87691	3.37468
H	1.47145	2.52640	4.25100
H	0.70350	2.13901	2.73125
H	1.41754	0.84681	3.71741
C	3.11031	-3.52119	-0.33574
H	2.64024	-3.10373	-1.22453
H	4.18118	-3.59592	-0.53017
H	2.74020	-4.53950	-0.18695
C	3.60827	-3.25364	2.09063
H	4.68214	-3.20506	1.90326

H	3.40211	-2.69931	3.00981
H	3.33634	-4.30101	2.25302
C	1.33359	-2.87601	1.25808
H	1.05679	-2.35582	2.17999
H	0.66581	-2.56483	0.45454
H	1.14702	-3.94002	1.43096
C	7.18968	1.74809	0.05017
H	6.53955	2.50935	-0.38802
H	7.32945	1.98137	1.10905
H	8.16602	1.82781	-0.43389
C	6.55166	0.07216	-1.66427
H	7.54270	0.16840	-2.11698
H	6.17763	-0.93114	-1.87623
H	5.88024	0.78070	-2.15441
C	7.59868	-0.65831	0.48732
H	7.66993	-0.49593	1.56583
H	7.28466	-1.69085	0.32211
H	8.59830	-0.54359	0.05884
C	-2.36933	-1.43854	-1.65253
C	-2.33855	-2.33617	-2.76084
C	-3.61625	-1.22405	-1.00406
C	-3.54463	-2.64091	-3.37719
C	-1.11438	-3.12662	-3.25922
C	-4.78033	-1.60173	-1.66630
C	-3.82313	-0.77227	0.45444
C	-4.77916	-2.21943	-2.90527
H	-3.51263	-3.25976	-4.26415
C	-0.60397	-2.63926	-4.61824
C	-1.53832	-4.60072	-3.42644
C	-0.00359	-3.15698	-2.21797
H	-5.72572	-1.40314	-1.18128
C	-2.53973	-0.43046	1.19073
C	-4.41395	-1.99372	1.18755
C	-4.81177	0.39024	0.59115
C	-6.04998	-2.52191	-3.68914
H	0.20150	-3.29395	-4.96166
H	-1.40397	-2.67483	-5.36292
H	-0.22289	-1.62338	-4.56684
H	-2.21247	-4.76008	-4.26875
H	-0.64370	-5.19904	-3.61615
H	-2.01903	-4.98574	-2.52381
H	0.43260	-2.18304	-2.01353
H	-0.37064	-3.59230	-1.28507
H	0.81527	-3.78177	-2.58015
H	-1.80592	-1.23382	1.10601
H	-2.07994	0.50127	0.86015
H	-2.75068	-0.29907	2.25449
H	-4.55682	-1.74990	2.24386
H	-5.37830	-2.29414	0.77633
H	-3.73816	-2.84978	1.12347
H	-5.79402	0.14642	0.18352
H	-4.94727	0.62949	1.64899
H	-4.46318	1.29501	0.09100
C	-7.29576	-1.95846	-3.01078
C	-6.22025	-4.03935	-3.82973
C	-5.93272	-1.89305	-5.08273
H	-7.47430	-2.41879	-2.03563
H	-7.22903	-0.87609	-2.87468
H	-8.17160	-2.16083	-3.63160
H	-5.38096	-4.49532	-4.35832
H	-6.29990	-4.51698	-2.85011
H	-7.13041	-4.26360	-4.39266
H	-5.82540	-0.80774	-5.01500
H	-5.07177	-2.28077	-5.63071
H	-6.82934	-2.11037	-5.66947
Ge	1.44211	0.68675	-2.34723

[R-P=C(Ge(II)Cl)-P(S)ClR](AuCl)

Coordination through Ge(II) atom

R= H

P	0.43110	0.76476	0.31787
C	0.03493	0.37151	-1.24486
P	-1.52503	-0.12198	-1.85741
Ge	0.87718	0.71438	-3.04612
Cl	1.40918	-1.32317	-3.61901
Au	2.16742	2.57971	-3.58636
Cl	3.43869	4.40916	-4.01917
H	1.76648	1.19498	0.04769
H	-1.63708	-1.52299	-1.88365
Cl	-3.10159	0.35164	-0.71444
S	-1.50127	0.70010	-3.66904

R = Me

P	0.45862	0.55260	0.35067
C	0.02421	0.31097	-1.23341
P	-1.57339	-0.07899	-1.82231
Ge	0.84573	0.68474	-3.03100
Cl	1.42279	-1.33724	-3.65733
C	-1.90584	-1.84118	-1.82265
H	-1.92824	-2.20744	-0.79496
H	-2.86278	-2.02683	-2.31238
H	-1.09900	-2.32071	-2.38123
C	2.23822	0.95899	0.26661
H	2.80757	0.02424	0.29584
H	2.50704	1.50623	-0.64005
H	2.51081	1.54446	1.14601
Au	2.19565	2.52622	-3.52896
Cl	3.53740	4.32446	-3.89631
Cl	-3.07467	0.68467	-0.71250
S	-1.48351	0.70872	-3.66510

R = *t*-Bu

P	0.41020	0.51378	0.05131
C	0.27549	0.15728	-1.56836
P	-1.28239	-0.22317	-2.30027
Ge	1.33295	0.34248	-3.27492
Cl	2.22995	-1.63556	-3.54984
Au	2.50732	2.26867	-3.89951
Cl	3.64384	4.17695	-4.40505
C	2.17410	0.95607	0.44914
C	3.23362	0.26692	-0.39606
C	2.25407	2.48043	0.29646
C	2.34679	0.57618	1.92328
H	3.10757	-0.81725	-0.42028
H	3.24660	0.63883	-1.42282
H	4.22225	0.48152	0.02275
H	1.48063	2.99398	0.87548
H	3.22445	2.82384	0.66939
H	2.16839	2.78869	-0.74782
H	3.32446	0.92100	2.27215
H	1.58337	1.03665	2.55614
H	2.30263	-0.50619	2.07119
C	-2.00109	-1.87804	-1.89838
C	-3.23831	-2.08243	-2.77042
C	-0.93680	-2.92305	-2.23318
C	-2.35997	-1.93855	-0.41643
H	-4.02393	-1.35978	-2.54442
H	-2.99797	-2.02094	-3.83393
H	-3.63400	-3.08271	-2.57511
H	-0.03562	-2.80011	-1.63068

H	-1.35522	-3.91095	-2.02240
H	-0.64824	-2.89250	-3.28452
H	-2.73047	-2.94349	-0.19639
H	-1.49359	-1.75759	0.22354
H	-3.14137	-1.22536	-0.15221
Cl	-2.71672	1.08952	-1.72539
S	-0.84072	0.00753	-4.24705

R = Ph

P	0.49886	0.57663	0.21323
C	0.07652	0.26664	-1.36877
P	-1.55384	-0.05971	-1.91043
Ge	0.88631	0.60358	-3.17407
Cl	1.48863	-1.40917	-3.82370
Au	2.20141	2.44505	-3.76313
Cl	3.51852	4.23294	-4.27026
C	2.27217	0.85014	0.19611
C	2.76192	1.70181	1.19257
C	3.16651	0.27048	-0.71009
C	4.10980	2.01152	1.24690
H	2.07652	2.13537	1.91421
C	4.51447	0.57137	-0.64140
H	2.81541	-0.44383	-1.44676
C	4.98400	1.44949	0.32740
H	4.47952	2.69010	2.00641
H	5.20187	0.12400	-1.34927
H	6.03986	1.69056	0.36898
C	-2.05569	-1.77710	-1.74491
C	-2.01496	-2.61118	-2.85736
C	-2.41067	-2.28272	-0.49411
C	-2.33981	-3.95200	-2.71758
H	-1.72656	-2.21330	-3.82255
C	-2.72977	-3.62198	-0.36568
H	-2.44457	-1.63376	0.37343
C	-2.69577	-4.45584	-1.47685
H	-2.31159	-4.60205	-3.58380
H	-3.00940	-4.01679	0.60382
H	-2.94913	-5.50444	-1.37157
Cl	-2.93517	0.95122	-0.83284
S	-1.44857	0.57335	-3.80863

R = Mes

P	0.53029	0.61398	0.49233
C	0.32057	-0.03896	-1.02811
P	-1.23339	-0.32166	-1.80424
Ge	1.43850	0.15304	-2.67678
Cl	1.82412	-1.95094	-3.20072
Au	3.09829	1.75308	-3.08236
Cl	4.74383	3.31512	-3.36455
C	2.28695	1.06200	0.52095
C	2.59098	2.43447	0.52787
C	3.31098	0.10526	0.58246
C	3.92043	2.82407	0.53425
C	4.62725	0.54703	0.59782
C	4.95506	1.89640	0.54766
H	4.15630	3.88286	0.49657
H	5.42380	-0.18968	0.64209
C	-1.94941	-1.97503	-1.90207
C	-3.01212	-2.17576	-2.81102
C	-1.48299	-3.04185	-1.10719
C	-3.56386	-3.44464	-2.91321
C	-2.07532	-4.28760	-1.26624
C	-3.10698	-4.51809	-2.16360
H	-4.38393	-3.59335	-3.60857
H	-1.71383	-5.10770	-0.65441
C	-3.58800	-1.10983	-3.69582
H	-3.70107	-0.14719	-3.19665
H	-2.95748	-0.94976	-4.57403
H	-4.57436	-1.41699	-4.04456
C	-0.38950	-2.92936	-0.08998
H	0.55487	-2.63313	-0.54832
H	-0.62484	-2.19787	0.68560
H	-0.24074	-3.89397	0.39526
C	-3.69613	-5.88272	-2.32946
H	-3.16090	-6.43615	-3.10738
H	-3.62503	-6.46279	-1.40812
H	-4.74441	-5.83113	-2.62783
C	3.02951	-1.36766	0.63583
H	2.16500	-1.59695	1.26497
H	2.82813	-1.77598	-0.35930
H	3.88795	-1.90574	1.04020
C	1.51201	3.47280	0.45049
H	1.15331	3.58715	-0.57725
H	0.64644	3.21598	1.07140
H	1.88260	4.44456	0.77868
C	6.37845	2.34646	0.46786
H	6.61137	2.64004	-0.56058
H	6.55884	3.21561	1.10401
H	7.06843	1.55326	0.75895
Cl	-2.67055	0.76849	-0.86021
S	-0.75151	0.45606	-3.60854

R = Mes*

P	-0.31651	1.58440	1.45279
C	0.23100	0.47217	0.32987
P	1.82931	-0.26199	0.12041
Cl	-1.16943	0.77956	-2.70954
C	3.53611	0.39308	0.17743
C	4.15716	0.66595	-1.07878
C	4.36737	0.14277	1.30252
C	5.46907	0.24878	-1.24025
C	5.67971	-0.24577	1.05558
C	6.22466	-0.31119	-0.21707
H	5.92928	0.39419	-2.20960
H	6.30001	-0.49478	1.90523
C	-2.11156	1.63568	1.09595
C	-2.63239	2.65227	0.26353
C	-2.96706	0.61618	1.59304
C	-3.90093	2.45794	-0.27750
C	-4.21277	0.48409	0.99375
C	-4.67637	1.34574	0.00696
H	-4.29054	3.19729	-0.95843
H	-4.84983	-0.33080	1.30299
C	-1.92956	4.00581	0.03932
C	-2.68605	-0.18325	2.88401
C	-6.01065	1.06997	-0.67700
C	4.03627	0.47188	2.77226
C	3.60104	1.58294	-2.18736
C	7.61560	-0.86251	-0.49931
C	-3.80055	-1.18909	3.18637
H	-3.56425	-1.70005	4.12246
H	-4.76868	-0.70231	3.32131
H	-3.89244	-1.95028	2.40723
C	-2.65686	0.83751	4.03317
H	-1.84110	1.55653	3.91395
H	-3.59331	1.39783	4.08392
H	-2.51307	0.32213	4.98719
C	-1.37580	-0.97026	2.90835
H	-1.27119	-1.62047	2.03770
H	-0.49373	-0.32614	2.96418
H	-1.35462	-1.59541	3.80508
C	-7.12905	1.00033	0.36952

H	-8.08654	0.80518	-0.12106
H	-6.96095	0.20027	1.09260
H	-7.21344	1.94199	0.91846
C	-6.37418	2.14965	-1.69293
H	-7.32398	1.89392	-2.16846
H	-6.49466	3.13048	-1.22454
H	-5.62282	2.23251	-2.48220
C	-5.91543	-0.27356	-1.41099
H	-5.13510	-0.24515	-2.17540
H	-5.68257	-1.09864	-0.73511
H	-6.86621	-0.49879	-1.90252
C	-2.76858	4.95135	-0.82352
H	-3.74957	5.14844	-0.38632
H	-2.24722	5.90801	-0.90345
H	-2.90841	4.56676	-1.83652
C	-1.75250	4.68029	1.40767
H	-2.71624	4.81160	1.90499
H	-1.10943	4.09720	2.07191
H	-1.29312	5.66527	1.28383
C	-0.56652	3.88927	-0.64251
H	0.18352	3.42294	0.00328
H	-0.62265	3.30631	-1.56235
H	-0.18886	4.88847	-0.87899
C	2.64447	1.04808	2.99216
H	2.44011	1.87380	2.30691
H	1.85265	0.30741	2.90160
H	2.57730	1.45018	4.00595
C	5.01499	1.60418	3.15336
H	4.80704	1.92779	4.17668
H	6.05661	1.28563	3.10601
H	4.89395	2.46601	2.49270
C	4.26891	-0.70087	3.72775
H	5.28804	-1.08596	3.66154
H	4.11231	-0.36592	4.75642
H	3.58320	-1.52458	3.53368
C	2.24494	2.19837	-1.86879
H	2.03422	2.98749	-2.59472
H	1.42016	1.49348	-1.93932
H	2.24256	2.65822	-0.87762
C	3.55974	0.91613	-3.56361
H	2.83444	0.10416	-3.59303
H	3.27143	1.65492	-4.31579
H	4.53425	0.51628	-3.85159
C	4.58615	2.77046	-2.25516
H	4.66499	3.27173	-1.28731
H	5.58772	2.46703	-2.56123
H	4.21771	3.49620	-2.98480
C	8.27374	-1.43856	0.75148
H	8.45154	-0.67258	1.51070
H	7.67101	-2.23396	1.19677
H	9.24365	-1.86625	0.48782
C	8.51355	0.25186	-1.05019
H	8.12603	0.66413	-1.98383
H	8.60809	1.07092	-0.33305
H	9.51378	-0.14088	-1.25127
C	7.49508	-1.98078	-1.54208
H	6.86898	-2.79610	-1.17219
H	7.05700	-1.61901	-2.47438
H	8.48385	-2.38698	-1.77109
Ge	-0.73746	-0.60959	-1.06275
Au	-2.32090	-2.28140	-0.62239
Cl	-3.92575	-3.82336	-0.07270
Cl	1.89993	-1.79592	1.47641
S	1.50370	-1.21701	-1.62817

Coordination through S atom

R= H

P	0.55384	0.18513	0.72298
C	0.29881	0.26732	-0.91382
P	-1.20860	0.54045	-1.72220
Ge	1.55521	0.48257	-2.57201
Cl	0.95409	-1.43527	-3.52419
Au	-1.96233	1.46360	-4.92101
Cl	-3.30319	1.12471	-6.69410
H	-1.66359	-0.64888	-2.30594
H	1.97559	0.02390	0.67438
Cl	-2.79050	1.10593	-0.62234
S	-0.61781	1.86614	-3.13616

R = Me

P	0.56302	0.20397	0.72679
C	0.28748	0.34193	-0.90389
P	-1.27143	0.51871	-1.63879
Ge	1.51076	0.74462	-2.54514
Cl	1.35208	-1.25653	-3.51743
C	-1.97008	-1.03813	-2.17861
H	-2.16938	-1.64839	-1.29537
H	-2.89164	-0.84466	-2.72931
H	-1.23669	-1.52523	-2.82452
C	2.37988	-0.00800	0.84942
H	2.60103	-1.08010	0.84094
H	2.92458	0.46624	0.03054
H	2.72496	0.39333	1.80398
Au	-2.02049	1.15991	-4.97878
Cl	-3.27488	0.60655	-6.76415
Cl	-2.70975	1.31161	-0.45528
S	-0.77672	1.76592	-3.17606

R = *t*-Bu

P	0.63953	-0.16224	0.52923
C	0.20369	0.48658	-0.94163
P	-1.46855	0.38520	-1.45538
Ge	1.01109	1.81325	-2.34853
Cl	1.67751	0.33119	-3.87845
Au	-2.54741	1.46314	-4.74453
Cl	-3.55937	1.08192	-6.72222
C	-2.02994	-1.27423	-2.08031
C	-3.49632	-1.19091	-2.49682
C	-1.15133	-1.67234	-3.26277
C	-1.86342	-2.28009	-0.94144
H	-4.15076	-0.95512	-1.65703
H	-3.65893	-0.46002	-3.29230
H	-3.78666	-2.16881	-2.88971
H	-0.09562	-1.72609	-2.99511
H	-1.47254	-2.66272	-3.59671
H	-1.25677	-0.98651	-4.10480
H	-2.19624	-3.25287	-1.31329
H	-0.82296	-2.38334	-0.62935
H	-2.46890	-2.02847	-0.06986
C	2.48764	-0.03817	0.75181
C	2.97405	-1.49171	0.69442
C	3.23660	0.80892	-0.25928
C	2.68974	0.51139	2.16738
H	2.43300	-2.13375	1.39550
H	2.86925	-1.91146	-0.30879
H	4.03424	-1.52680	0.96605
H	2.90856	1.85139	-0.23973
H	4.30649	0.80505	-0.02378
H	3.13386	0.41624	-1.27460

H	3.75265	0.47178	2.42510
H	2.36381	1.55146	2.24757
H	2.14377	-0.07312	2.91279
Cl	-2.76198	0.78799	0.06408
S	-1.52712	1.95215	-2.77159

R = Ph

P	0.44416	-0.04844	0.87282
C	0.16022	0.23173	-0.74429
P	-1.41828	0.30584	-1.46152
Ge	1.34488	1.11951	-2.19816
Cl	1.60858	-0.61910	-3.57802
Au	-2.47052	1.73877	-4.57231
Cl	-3.80830	1.72423	-6.38579
C	-1.93657	-1.25751	-2.17744
C	-2.44427	-2.24046	-1.32615
C	-1.76871	-1.51812	-3.53302
C	-2.79261	-3.47528	-1.84116
H	-2.57675	-2.04115	-0.26935
C	-2.12750	-2.75767	-4.03895
H	-1.36280	-0.76366	-4.19400
C	-2.63805	-3.73256	-3.19762
H	-3.19355	-4.23665	-1.18274
H	-2.00857	-2.95339	-5.09757
H	-2.92057	-4.69902	-3.59888
C	2.24458	-0.06105	0.99578
C	3.09899	-0.49693	-0.02291
C	2.79389	0.37530	2.20525
C	4.47219	-0.45692	0.15487
H	2.68879	-0.91752	-0.93572
C	4.16732	0.42432	2.37425
H	2.13768	0.68997	3.01087
C	5.00625	0.01188	1.34771
H	5.12686	-0.80129	-0.63721
H	4.58516	0.77885	3.30927
H	6.08129	0.04193	1.48326
Cl	-2.88912	0.75511	-0.13761
S	-1.11177	1.84091	-2.75557

R = Mes

P	0.14435	0.66904	0.42163
C	0.07182	0.05350	-1.12015
P	-1.25892	-0.04799	-2.23118
Ge	1.69886	-0.06902	-2.40973
Cl	1.56227	-2.28072	-2.85859
Au	0.12341	2.83342	-3.27353
Cl	0.65112	4.96009	-2.72526
C	-1.97193	-1.70507	-2.37367
C	-2.43503	-2.29423	-1.17410
C	-2.09221	-2.40874	-3.59377
C	-2.96387	-3.57708	-1.21961
C	-2.63108	-3.68784	-3.56285
C	-3.05974	-4.30094	-2.39572
H	-3.32049	-4.01678	-0.29356
H	-2.72265	-4.22307	-4.50229
C	1.93140	0.59782	0.77741
C	2.62998	1.81301	0.86193
C	2.57680	-0.61937	1.05690
C	3.98212	1.78443	1.17805
C	3.92891	-0.59311	1.37186
C	4.65221	0.59343	1.42474
H	4.52451	2.72352	1.23553
H	4.43051	-1.53007	1.59534
C	1.95144	3.12101	0.59054
H	1.83705	3.31066	-0.48118
H	0.94585	3.15586	1.02420
H	2.52166	3.95180	1.00790
C	1.84388	-1.92962	1.06190
H	0.93050	-1.87250	1.66254
H	1.55272	-2.24292	0.05583
H	2.47028	-2.71511	1.48620
C	6.11807	0.58457	1.73040
H	6.70172	0.47043	0.81150
H	6.43231	1.51668	2.20289
H	6.38334	-0.24366	2.38995
C	-2.40051	-1.64050	0.17520
H	-1.42192	-1.76931	0.64428
H	-2.61409	-0.57309	0.15301
H	-3.14117	-2.10787	0.82486
C	-1.68749	-1.89739	-4.94352
H	-2.12425	-0.92559	-5.17451
H	-0.60278	-1.80342	-5.02428
H	-2.01717	-2.60104	-5.70788
C	-3.59925	-5.69552	-2.40952
H	-4.25413	-5.87941	-1.55669
H	-4.15670	-5.89767	-3.32579
H	-2.78062	-6.42020	-2.35967
Cl	-2.84001	1.17225	-1.82180
S	-0.32211	0.67667	-3.91562

R = Mes*

P	-2.04108	1.89965	0.25708
C	-1.10123	0.52480	0.20665
P	0.64165	0.39204	0.27366
C	-3.73050	1.17616	0.29444
C	-4.53426	1.25964	-0.87637
C	-4.17734	0.40844	1.39172
C	-5.59673	0.37953	-0.99423
C	-5.24394	-0.47060	1.18298
C	-5.92904	-0.55112	-0.01291
H	-6.18858	0.39880	-1.89733
H	-5.55527	-1.09632	2.00791
C	-3.74962	0.57751	2.86624
C	-4.31583	2.31293	-1.97865
C	-7.04234	-1.56078	-0.26766
C	-3.01623	2.08637	-2.75386
H	-2.12919	2.24301	-2.13472
H	-2.96140	1.07194	-3.15189
H	-2.95244	2.79745	-3.58286
C	-4.31191	3.71170	-1.34623
H	-5.25129	3.90188	-0.82145
H	-3.49456	3.84776	-0.63511
H	-4.19647	4.47092	-2.12478
C	-5.44677	2.30177	-3.01035
H	-5.47206	1.37341	-3.58559
H	-6.42568	2.45624	-2.55052
H	-5.28178	3.11749	-3.71784
C	-3.28953	-0.74408	3.48833
H	-2.38119	-1.11549	3.00906
H	-3.07347	-0.59645	4.55004
H	-4.04842	-1.52428	3.41164
C	-2.66551	1.61919	3.12394
H	-1.67839	1.28138	2.81163
H	-2.88845	2.57820	2.64378
H	-2.60878	1.81290	4.19893
C	-4.99914	1.07224	3.62053
H	-5.34867	2.02370	3.21176
H	-5.82398	0.36142	3.56482
H	-4.75570	1.22259	4.67588
C	-7.24988	-2.50354	0.91445
H	-7.56757	-1.96906	1.81364
H	-8.03220	-3.22606	0.67052

H	-6.34237	-3.06578	1.14829
C	-6.67438	-2.40295	-1.49565
H	-6.56311	-1.78660	-2.38982
H	-5.73156	-2.93212	-1.34045
H	-7.45690	-3.14118	-1.69196
C	-8.35933	-0.82213	-0.53293
H	-8.28827	-0.16361	-1.40092
H	-9.16036	-1.54107	-0.72588
H	-8.64815	-0.21401	0.32805
C	2.04762	1.44876	-0.18563
C	2.67348	1.14140	-1.42656
C	2.81462	2.10394	0.82244
C	4.06296	1.09130	-1.44272
C	4.19200	2.02123	0.72227
C	4.84469	1.40787	-0.34358
H	4.54675	0.78921	-2.36104
H	4.78793	2.46613	1.50944
C	1.97871	1.07085	-2.80076
C	2.31013	-0.19735	-3.59004
C	2.53882	2.28334	-3.57716
H	1.89577	-1.08552	-3.11482
H	1.88223	-0.11966	-4.59293
H	3.38586	-0.34297	-3.70162
H	2.33094	3.21780	-3.04992
H	3.61605	2.21390	-3.73081
H	2.05919	2.33136	-4.55839
C	2.26572	3.07948	1.88301
C	2.85162	4.45514	1.49950
C	2.72148	2.74728	3.30574
H	2.56219	4.73476	0.48369
H	2.46601	5.21451	2.18527
H	3.94034	4.47082	1.55633
H	2.31363	1.79859	3.65229
H	3.80877	2.69377	3.38291
H	2.38273	3.53146	3.98805
C	6.35097	1.19849	-0.30674
C	7.05802	2.55690	-0.22605
C	6.85800	0.45552	-1.53977
H	6.77335	3.11213	0.67033
H	8.14081	2.40968	-0.19446
H	6.82459	3.17610	-1.09607
H	6.37491	-0.51845	-1.64742
H	6.70439	1.02873	-2.45817
H	7.93197	0.28250	-1.44103
Cl	-2.43315	-1.56869	-1.92584
Cl	0.98834	-0.04854	2.24403
Au	2.74227	-2.39295	0.08661
Cl	4.71939	-3.30536	0.70201
C	0.75294	3.25834	1.85437
H	0.39468	3.48744	0.84828
H	0.21065	2.39507	2.23393
H	0.48141	4.10194	2.49394
C	0.46788	1.26985	-2.76541
H	-0.08123	0.41039	-2.38330
H	0.19921	2.15463	-2.18249
H	0.11152	1.43215	-3.78570
C	6.69637	0.36358	0.93355
H	6.39292	0.86492	1.85527
H	6.20766	-0.61318	0.90203
H	7.77707	0.20465	0.98110
Ge	-1.64876	-1.49925	0.17454
S	0.81337	-1.43050	-0.64329

Coordination through P(sp^2) atom

R= H

P	0.40495	0.26580	0.51080
C	0.14573	0.12163	-1.09943
P	-1.33747	0.05230	-1.99457
Ge	1.39965	0.36795	-2.77792
Cl	1.28785	-1.78964	-3.36996
Au	-0.91505	0.36172	2.26140
Cl	-2.25557	0.46033	4.06902
H	1.80965	0.34850	0.63747
H	-1.64780	-1.29531	-2.23667
Cl	-3.00036	0.66029	-1.04067
S	-0.88658	1.06576	-3.63239

R = Me

P	0.47566	0.36556	0.49074
C	0.13926	0.20867	-1.10405
P	-1.40238	0.02837	-1.88537
Ge	1.30337	0.39334	-2.84065
Cl	1.50442	-1.82535	-3.17314
C	-1.94753	-1.67900	-1.85727
H	-2.14355	-1.98082	-0.82694
H	-2.85447	-1.76859	-2.45702
H	-1.14554	-2.28098	-2.29027
C	2.26229	0.47245	0.74373
H	2.59512	-0.44301	1.24008
H	2.79529	0.59118	-0.19981
H	2.47709	1.31046	1.40964
Au	-0.88211	0.38945	2.22430
Cl	-2.25799	0.37649	4.01486
Cl	-2.91766	1.02427	-0.98552
S	-1.01524	0.72006	-3.71134

R = *t*-Bu

P	0.66299	-0.23996	0.47562
C	0.13912	0.32592	-0.97850
P	-1.50694	0.20511	-1.57105
Ge	0.86609	1.72201	-2.39008
Cl	1.73386	0.26717	-3.87195
C	-2.01622	-1.46684	-2.19053
C	-3.42250	-1.32701	-2.77258
C	-1.03173	-1.87343	-3.28516
C	-2.00014	-2.48635	-1.05742
H	-4.16094	-1.08113	-2.00826
H	-3.46224	-0.57097	-3.55941
H	-3.70192	-2.28654	-3.21581
H	-0.01129	-1.96319	-2.91048
H	-1.34110	-2.84886	-3.67080
H	-1.02358	-1.16432	-4.11343
H	-2.32538	-3.44868	-1.46267
H	-0.99871	-2.62712	-0.64694
H	-2.67579	-2.22107	-0.24337
C	2.47647	-0.04779	0.78641
C	3.02599	-1.47438	0.90611
C	3.20209	0.70955	-0.31287
C	2.61938	0.67514	2.12863
H	2.50240	-2.05146	1.67221
H	2.96063	-2.01341	-0.04171
H	4.08086	-1.41842	1.19152
H	2.85569	1.74278	-0.39296
H	4.26922	0.74481	-0.07266
H	3.10129	0.22316	-1.28550
H	3.67994	0.71723	2.39353
H	2.24214	1.69921	2.08013

H	2.09315	0.15291	2.93113
Cl	-2.86134	0.62065	-0.11175
Au	-0.51553	-1.21128	2.07949
Cl	-1.69333	-2.21127	3.73470
S	-1.55277	1.60530	-2.99109

R = Ph

P	0.47361	0.56891	0.11707
C	0.34035	0.02702	-1.42883
P	-1.12657	-0.28581	-2.31223
Ge	1.69179	-0.11914	-3.01870
Cl	2.06893	-2.34105	-2.87864
Au	-1.12110	1.19068	1.51404
Cl	-2.76256	1.77794	2.95523
Cl	-2.57534	1.07991	-1.92968
C	2.17699	0.62716	0.63787
C	3.15623	-0.20677	0.09002
C	2.51840	1.53943	1.63988
C	4.46606	-0.10124	0.52422
H	2.88933	-0.96574	-0.63733
C	3.83230	1.64265	2.06126
H	1.75171	2.16787	2.08115
C	4.80531	0.82596	1.50102
H	5.22284	-0.75350	0.10483
H	4.09670	2.35704	2.83174
H	5.83293	0.90303	1.83733
C	-1.87515	-1.85414	-1.85431
C	-1.61460	-2.97954	-2.63001
C	-2.65041	-1.95333	-0.69896
C	-2.13871	-4.20536	-2.25016
H	-1.00226	-2.89426	-3.51920
C	-3.16651	-3.18231	-0.32894
H	-2.85865	-1.07801	-0.09431
C	-2.91186	-4.30666	-1.10411
H	-1.93868	-5.08255	-2.85380
H	-3.77266	-3.26042	0.56570
H	-3.32094	-5.26684	-0.81120
S	-0.51141	-0.19511	-4.20201

R = Mes

P	0.51993	0.55454	0.63997
C	0.42469	-0.26154	-0.78234
P	-0.92456	-0.48313	-1.87126
Ge	2.01237	-0.48879	-2.11711
Cl	1.98381	-2.74981	-2.09680
Au	-1.07702	1.25867	1.99161
Cl	-2.68466	1.94991	3.42595
C	2.25371	0.80101	1.06269
C	2.99513	-0.23066	1.65436
C	2.82204	2.06584	0.83664
C	4.31503	0.02585	2.00374
C	4.14420	2.26774	1.20070
C	4.90702	1.26294	1.78683
H	4.89587	-0.76801	2.46313
H	4.59229	3.24041	1.02155
C	-1.81704	-2.05371	-1.88786
C	-1.71822	-2.99198	-0.84048
C	-2.65392	-2.31262	-2.99615
C	-2.42710	-4.18051	-0.95112
C	-3.34051	-3.51814	-3.03873
C	-3.23403	-4.47305	-2.03980
H	-2.34734	-4.90037	-0.14299
H	-3.98668	-3.70989	-3.88956
C	2.03867	3.17211	0.19768
H	1.77826	2.92956	-0.83719
H	1.10200	3.36432	0.73090
H	2.61211	4.09921	0.18814
C	2.40781	-1.58624	1.90383
H	1.39274	-1.52189	2.30733
H	2.35789	-2.17025	0.97956
H	3.01442	-2.14473	2.61740
C	6.32280	1.52249	2.19860
H	6.35437	2.06422	3.14892
H	6.87713	0.59233	2.32955
H	6.84489	2.13365	1.45972
C	-0.90575	-2.79607	0.40130
H	0.15471	-2.69583	0.16726
H	-1.21698	-1.90904	0.95741
H	-1.02768	-3.65728	1.05825
C	-2.86242	-1.37615	-4.15023
H	-2.95636	-0.33297	-3.84802
H	-2.03212	-1.43518	-4.85766
H	-3.77598	-1.64983	-4.67918
C	-3.95598	-5.77944	-2.13704
H	-4.86024	-5.69168	-2.74121
H	-3.31764	-6.53354	-2.60800
H	-4.23259	-6.15606	-1.15088
Cl	-2.38558	0.88783	-1.47080
S	0.03726	-0.05967	-3.57882

R = Mes*

P	-1.10410	0.77572	0.12485
C	-0.37231	-0.68442	0.33634
P	1.29132	-1.22826	0.46696
Cl	-2.04971	-2.69047	-1.78087
C	2.91634	-0.49582	0.06998
C	3.47690	-0.90038	-1.18065
C	3.79053	-0.03560	1.09326
C	4.82740	-1.20982	-1.20069
C	5.13341	-0.38659	0.98647
C	5.66150	-1.06339	-0.09973
H	5.25056	-1.56582	-2.13134
H	5.79353	-0.09465	1.79075
C	-2.89247	0.45982	0.16895
C	-3.65887	0.41563	-1.02636
C	-3.44901	-0.01066	1.38555
C	-4.83849	-0.31902	-1.00086
C	-4.61470	-0.76406	1.30924
C	-5.29649	-0.98738	0.12461
H	-5.40868	-0.40135	-1.91096
H	-5.01190	-1.18419	2.22431
C	-3.31909	1.19830	-2.30864
C	-2.97106	0.33415	2.81312
C	-6.51610	-1.90173	0.10323
C	3.47795	0.99173	2.20370
C	2.79549	-0.82793	-2.56409
C	7.10544	-1.54388	-0.15861
C	-4.17979	0.90566	3.57912
H	-3.86157	1.20751	4.58021
H	-4.58457	1.78455	3.07167
H	-4.98743	0.18372	3.69746
C	-1.90053	1.42023	2.88759
H	-0.91523	1.07581	2.57142
H	-2.17084	2.30666	2.30771
H	-1.78742	1.73625	3.92820
C	-2.46668	-0.91138	3.54608
H	-3.21976	-1.70171	3.56248
H	-1.56888	-1.31465	3.07434
H	-2.22021	-0.66007	4.58165
C	-7.58833	-1.36153	1.05642
H	-8.46238	-2.01848	1.04756
H	-7.22795	-1.30384	2.08550

H	-7.91174	-0.36159	0.75671
C	-7.12755	-2.02058	-1.29022
H	-7.97819	-2.70531	-1.25569
H	-7.49531	-1.05950	-1.65890
H	-6.41148	-2.41811	-2.01341
C	-6.08394	-3.30102	0.55937
H	-5.31600	-3.70672	-0.10341
H	-5.67841	-3.28856	1.57326
H	-6.94090	-3.98022	0.54975
C	-4.37037	0.99379	-3.40386
H	-5.36703	1.31023	-3.08828
H	-4.09312	1.60519	-4.26526
H	-4.41885	-0.04468	-3.74004
C	-3.32436	2.69490	-1.96840
H	-4.30258	3.00016	-1.58847
H	-2.57763	2.95370	-1.21452
H	-3.10462	3.28425	-2.86243
C	-1.98144	0.79592	-2.93184
H	-1.12297	1.04276	-2.30458
H	-1.94791	-0.27343	-3.14199
H	-1.83870	1.34604	-3.86624
C	2.04532	1.50717	2.23390
H	1.71809	1.81022	1.23650
H	1.33428	0.79548	2.64370
H	2.00230	2.40070	2.86074
C	4.33823	2.22648	1.85174
H	4.15294	3.01140	2.58944
H	5.40583	2.00555	1.85475
H	4.07200	2.62135	0.86873
C	3.87985	0.50721	3.59952
H	4.93881	0.24880	3.65658
H	3.70013	1.30653	4.32319
H	3.30250	-0.36397	3.90790
C	1.35274	-0.33932	-2.55337
H	1.05596	-0.10875	-3.57945
H	0.64624	-1.08100	-2.18488
H	1.25045	0.58317	-1.97501
C	2.87287	-2.14739	-3.33553
H	2.29436	-2.92844	-2.84247
H	2.46321	-2.00388	-4.33878
H	3.90003	-2.49979	-3.44800
C	3.58747	0.24554	-3.34198
H	3.55203	1.20847	-2.82689
H	4.63425	-0.02635	-3.48131
H	3.13829	0.37348	-4.33029
C	7.84840	-1.30557	1.15318
H	7.94094	-0.24122	1.38379
H	7.35493	-1.79983	1.99373
H	8.85981	-1.71134	1.07710
C	7.85043	-0.80674	-1.27807
H	7.40061	-0.98927	-2.25593
H	7.85226	0.27201	-1.10428
H	8.88881	-1.14626	-1.32177
C	7.11000	-3.04951	-0.45025
H	6.59479	-3.60287	0.33847
H	6.61689	-3.27955	-1.39672
H	8.13820	-3.41655	-0.50917
Cl	1.43221	-1.65980	2.47905
Au	-0.13343	2.74595	-0.25101
Cl	0.88841	4.73695	-0.63753
Ge	-1.24633	-2.63098	0.32575
S	1.12356	-3.04083	-0.35681

Coordination through π(C=P) bond

R= H

P	0.35501	-0.10812	0.70139
C	0.12830	0.51893	-0.89323
P	-1.31906	0.48494	-1.87039
Ge	1.39656	0.94751	-2.54549
Cl	1.32299	-1.15776	-3.33347
Au	0.19914	2.23972	0.39055
Cl	0.24377	4.31826	1.23622
H	1.78351	-0.14506	0.67229
H	-1.51637	-0.84028	-2.28876
Cl	-3.07584	0.84634	-0.94252
S	-0.88229	1.71475	-3.35561

R = Me

P	0.40701	0.06140	0.70115
C	0.07869	0.67299	-0.87985
P	-1.41701	0.51518	-1.77788
Ge	1.28100	1.14367	-2.56053
Cl	1.60410	-1.00565	-3.18852
C	-1.79956	-1.21367	-2.06624
H	-2.01772	-1.70199	-1.11502
H	-2.66542	-1.27219	-2.72708
H	-0.92855	-1.66723	-2.54433
C	2.23020	-0.12584	0.72153
H	2.44585	-1.16209	0.44041
H	2.75232	0.53995	0.03399
H	2.59321	0.03231	1.73871
Au	0.13505	2.40838	0.39145
Cl	0.16524	4.46779	1.29367
Cl	-3.07759	1.19150	-0.82092
S	-1.02456	1.53165	-3.44177

R = *t*-Bu

P	0.54041	0.09381	0.52283
C	0.04197	0.74293	-1.00389
P	-1.54613	0.41190	-1.71783
Ge	0.90843	1.69259	-2.69872
Cl	1.87311	-0.10878	-3.66427
Au	-0.03902	2.39742	0.40189
Cl	-0.14982	4.36105	1.49858
C	-1.98399	-1.38200	-1.91222
C	-3.26204	-1.44820	-2.74843
C	-0.84130	-2.07141	-2.65362
C	-2.20504	-2.03226	-0.54948
H	-4.10680	-0.97357	-2.24660
H	-3.12862	-0.98556	-3.72786
H	-3.51081	-2.50151	-2.90394
H	0.08886	-2.05983	-2.08401
H	-1.12545	-3.11478	-2.81701
H	-0.64506	-1.61433	-3.62374
H	-2.41956	-3.09233	-0.71187
H	-1.32247	-1.96666	0.08985
H	-3.04970	-1.59505	-0.01667
C	2.40515	0.07132	0.62389
C	2.78098	-1.33807	0.14896
C	3.15897	1.11812	-0.17783
C	2.71716	0.21990	2.11499
H	2.23507	-2.11552	0.69229
H	2.59837	-1.46042	-0.92112
H	3.84802	-1.50184	0.33066
H	2.85843	2.13427	0.08685
H	4.23062	1.02584	0.02949
H	3.03687	0.97487	-1.25344
H	3.78748	0.06251	2.27798

H	2.46408	1.21788	2.48134
H	2.17563	-0.51203	2.72059
Cl	-3.07892	1.13091	-0.57625
S	-1.45263	1.39045	-3.45147

R = Ph

P	0.27494	0.58089	0.51121
C	0.13287	0.69275	-1.20807
P	-1.35332	0.47072	-2.11201
Ge	1.39966	0.84224	-2.91313
Cl	1.62370	-1.35577	-3.39486
Au	0.30239	2.73943	-0.50611
Cl	0.51238	4.96699	-0.25411
C	-1.86086	-1.25367	-2.12669
C	-2.25519	-1.86073	-0.93463
C	-1.84909	-1.97430	-3.31458
C	-2.63569	-3.19018	-0.93858
H	-2.27214	-1.29669	-0.00830
C	-2.23762	-3.30541	-3.30957
H	-1.52794	-1.49626	-4.23158
C	-2.62800	-3.91158	-2.12633
H	-2.94188	-3.66487	-0.01392
H	-2.22795	-3.86922	-4.23467
H	-2.92812	-4.95320	-2.12583
C	2.03058	0.29715	0.84597
C	2.87154	-0.44548	0.01567
C	2.52054	0.79054	2.05859
C	4.19015	-0.66141	0.38260
H	2.49224	-0.88279	-0.90117
C	3.84279	0.58512	2.41153
H	1.86571	1.35111	2.71852
C	4.67858	-0.13995	1.57246
H	4.83735	-1.24159	-0.26487
H	4.22160	0.98686	3.34402
H	5.71242	-0.30703	1.85230
Cl	-2.95695	1.41341	-1.28369
S	-0.88372	1.20321	-3.89858

R = Mes

P	0.24666	0.48768	1.07895
C	-0.09571	0.68983	-0.60987
P	-1.52610	0.50805	-1.62663
Ge	1.24927	1.30286	-2.14127
Cl	1.63717	-0.66675	-3.19212
Au	-0.23823	2.67441	0.27251
Cl	-0.47039	4.87692	0.72310
C	-1.86147	-1.21994	-2.05906
C	-2.14087	-2.11585	-1.00100
C	-1.83047	-1.69853	-3.39097
C	-2.26811	-3.46954	-1.28188
C	-1.98257	-3.06382	-3.59863
C	-2.17139	-3.97183	-2.56867
H	-2.46913	-4.14806	-0.45870
H	-1.95249	-3.42705	-4.62067
C	2.06413	0.31454	1.12372
C	2.58661	-0.91704	0.68171
C	2.90423	1.25841	1.73653
C	3.95476	-1.13042	0.76894
C	4.26769	0.99547	1.79451
C	4.81728	-0.17812	1.29758
H	4.35512	-2.07996	0.42651
H	4.91826	1.73174	2.25703
C	2.39173	2.53090	2.33873
H	2.22147	3.30324	1.58289
H	1.44378	2.38615	2.86384
H	3.11162	2.92828	3.05571
C	1.70695	-2.02285	0.17617
H	0.84378	-2.18290	0.83110
H	1.32825	-1.81936	-0.82786
H	2.26481	-2.95913	0.13476
C	6.29472	-0.41602	1.33676
H	6.76924	0.15030	2.13979
H	6.52501	-1.47379	1.47693
H	6.75858	-0.10359	0.39588
C	-2.41314	-1.71299	0.41777
H	-2.20968	-2.54884	1.08833
H	-1.83530	-0.86176	0.77134
H	-3.46729	-1.44481	0.53110
C	-1.68319	-0.86067	-4.62654
H	-2.39316	-0.03435	-4.65396
H	-0.68247	-0.43626	-4.70959
H	-1.85945	-1.48664	-5.50180
C	-2.27049	-5.43908	-2.84062
H	-2.75185	-5.63440	-3.80043
H	-1.27256	-5.88691	-2.87976
H	-2.83169	-5.95389	-2.05937
Cl	-3.28361	1.16263	-0.79778
S	-1.01861	1.80198	-3.06373

R = Mes*

P	0.67710	0.17138	1.07823
C	0.40520	0.14580	-0.64979
P	-1.12049	-0.08069	-1.59917
Cl	2.82145	-1.08469	-3.04991
Au	0.37785	2.26707	-0.09613
Cl	0.19374	4.52900	-0.03306
C	2.51722	0.04287	1.20675
C	3.19299	-1.12698	0.76007
C	3.29640	1.15863	1.60442
C	4.51795	-1.00611	0.37209
C	4.61876	1.21248	1.16039
C	5.22986	0.18419	0.46612
H	5.02278	-1.88483	-0.00749
H	5.19295	2.09773	1.39442
C	2.91961	2.23319	2.64697
C	2.67371	-2.56842	0.93151
C	6.64004	0.28445	-0.10282
C	3.86710	1.98902	3.84046
H	3.72943	0.98544	4.25137
H	4.91572	2.09657	3.56140
H	3.65125	2.71421	4.62981
C	3.14733	3.65574	2.12932
H	2.50481	3.88753	1.27967
H	2.91775	4.37320	2.92189
H	4.18345	3.82194	1.82991
C	1.50994	2.13948	3.22347
H	1.43733	2.81628	4.07908
H	0.73482	2.45028	2.52137
H	1.27958	1.13372	3.58454
C	2.98382	-3.45863	-0.27433
H	2.56450	-3.04861	-1.19152
H	4.05623	-3.58711	-0.42763
H	2.56324	-4.45448	-0.10895
C	3.42640	-3.12544	2.15530
H	4.50587	-3.11708	1.99645
H	3.21361	-2.53464	3.04994
H	3.11574	-4.15758	2.34352
C	1.18914	-2.69915	1.24766
H	0.90058	-2.11916	2.13067
H	0.55684	-2.41256	0.40841
H	0.96513	-3.74495	1.47696
C	7.25911	1.66058	0.12593

H	6.65774	2.45456	-0.32353
H	7.38420	1.88178	1.18925
H	8.24954	1.69498	-0.33415
C	6.57678	0.02717	-1.61320
H	7.58075	0.07506	-2.04449
H	6.15552	-0.95390	-1.84006
H	5.95381	0.77302	-2.11202
C	7.54425	-0.76500	0.55419
H	7.59861	-0.61473	1.63543
H	7.18851	-1.78104	0.37223
H	8.55734	-0.69208	0.14911
C	-2.35368	-1.46487	-1.67172
C	-2.32877	-2.30514	-2.82637
C	-3.58230	-1.34479	-0.96571
C	-3.53899	-2.57090	-3.44792
C	-1.13193	-3.14531	-3.31012
C	-4.75253	-1.67995	-1.63881
C	-3.74757	-1.07490	0.54149
C	-4.76739	-2.18280	-2.92958
H	-3.51559	-3.14376	-4.36665
C	-0.70394	-2.85502	-4.74997
C	-1.59831	-4.61649	-3.24870
C	0.05393	-3.06351	-2.36449
H	-5.68981	-1.53778	-1.11964
C	-2.45267	-0.75577	1.26864
C	-4.24794	-2.41253	1.12670
C	-4.78110	0.00785	0.86235
C	-6.04762	-2.41052	-3.72143
H	0.01982	-3.60880	-5.07069
H	-1.55418	-2.89752	-5.43483
H	-0.23639	-1.87756	-4.84423
H	-2.36626	-4.84658	-3.98793
H	-0.74105	-5.26466	-3.44917
H	-1.99091	-4.86717	-2.26031
H	0.49887	-2.07281	-2.30802
H	-0.23350	-3.39106	-1.36298
H	0.84616	-3.72626	-2.71867
H	-1.67561	-1.48900	1.04202
H	-2.07899	0.24282	1.05017
H	-2.62210	-0.79021	2.34757
H	-4.35959	-2.31284	2.20986
H	-5.21249	-2.70728	0.71164
H	-3.53505	-3.21700	0.92984
H	-5.76519	-0.22219	0.45099
H	-4.89287	0.08764	1.94681
H	-4.47370	0.98227	0.48387
C	-7.28240	-1.90274	-2.98183
C	-6.22883	-3.90835	-3.99572
C	-5.93789	-1.65942	-5.05400
H	-7.45571	-2.44923	-2.05120
H	-7.20497	-0.83805	-2.74815
H	-8.16544	-2.04131	-3.60985
H	-5.40166	-4.31894	-4.57797
H	-6.29712	-4.47284	-3.06255
H	-7.14888	-4.07537	-4.56230
H	-5.81949	-0.58580	-4.88997
H	-5.08527	-2.00359	-5.64270
H	-6.84210	-1.81645	-5.64823
Ge	1.72078	0.72972	-2.25028
Cl	-2.43847	1.46280	-1.22391
S	-0.41809	0.33249	-3.42710

[R-P=C(Ge(II)Cl)-P(S)MeR](AuCl)

Coordination through Ge(II) atom

R= H

P	0.40301	0.90251	0.24315
C	0.03379	0.50646	-1.32529
P	-1.55421	0.08076	-1.96107
Ge	0.96087	0.74887	-3.09381
Cl	1.43375	-1.33601	-3.55485
C	-2.97853	0.67566	-1.04442
H	-2.93766	1.76399	-0.99189
H	-3.89208	0.36644	-1.55564
H	-2.96277	0.25251	-0.03721
Au	2.38080	2.51766	-3.63886
Cl	3.76394	4.26812	-4.07860
H	1.76108	1.28521	0.02005
H	-1.69073	-1.32220	-1.95541
S	-1.36547	0.81169	-3.82225

R = Me

P	0.43164	0.57740	0.28206
C	0.03861	0.35603	-1.31429
P	-1.57794	0.00019	-1.92069
Ge	0.92512	0.67508	-3.08507
Cl	1.53113	-1.36991	-3.61434
C	-2.91467	0.81354	-1.03229
H	-2.74653	1.89024	-1.04828
H	-3.86608	0.58550	-1.51675
H	-2.93634	0.45929	0.00081
C	-1.91656	-1.76668	-1.87452
H	-1.98075	-2.10244	-0.83709
H	-2.85486	-1.97312	-2.39296
H	-1.09848	-2.28118	-2.38055
C	2.21645	0.97372	0.26801
H	2.77684	0.03989	0.37896
H	2.53375	1.47036	-0.65148
H	2.44858	1.60462	1.12775
Au	2.30656	2.49380	-3.58540
Cl	3.66306	4.28652	-3.95176
S	-1.36285	0.68353	-3.81347

R = *t*-Bu

P	0.42465	0.44757	0.09617
C	0.23946	0.15700	-1.52973
P	-1.36582	-0.18372	-2.21209
Ge	1.23646	0.40300	-3.26111
Cl	2.18235	-1.54261	-3.61484
C	-2.59269	1.02108	-1.66349
H	-2.22634	2.01104	-1.93616
H	-3.54777	0.84320	-2.15933
H	-2.71613	0.96525	-0.58084
Au	2.34671	2.38296	-3.84069
Cl	3.40080	4.35415	-4.29834
C	2.19270	0.90401	0.46978
C	3.23388	0.27254	-0.44071
C	2.25137	2.43400	0.38995
C	2.41583	0.45423	1.91681
H	3.12311	-0.81120	-0.51176
H	3.20522	0.69136	-1.44887
H	4.23273	0.48409	-0.04496
H	1.48987	2.90827	1.01653
H	3.22847	2.77291	0.74935
H	2.12987	2.79045	-0.63531
H	3.39946	0.79449	2.25358
H	1.66636	0.87195	2.59457

H	2.38911	-0.63468	2.01141
C	-2.02141	-1.87485	-1.84584
C	-3.24636	-2.10866	-2.73003
C	-0.93738	-2.89715	-2.17591
C	-2.40063	-1.96028	-0.36838
H	-4.06459	-1.42243	-2.50070
H	-3.00090	-2.02064	-3.79045
H	-3.61271	-3.12405	-2.55548
H	-0.05131	-2.76731	-1.55272
H	-1.33748	-3.89886	-1.99622
H	-0.62568	-2.83618	-3.21964
H	-2.71131	-2.98539	-0.14811
H	-1.55943	-1.72262	0.28725
H	-3.23483	-1.30311	-0.11494
S	-0.94156	0.06228	-4.18150

R = Ph

P	0.47888	0.57550	0.14639
C	0.09452	0.26922	-1.44347
P	-1.55477	-0.04073	-1.98829
Ge	0.93634	0.55176	-3.23993
Cl	1.55427	-1.48375	-3.80180
C	-2.80184	0.92108	-1.11334
H	-2.55151	1.97819	-1.20441
H	-3.77855	0.72908	-1.56060
H	-2.82258	0.63818	-0.05924
Au	2.26679	2.37473	-3.85518
Cl	3.58357	4.16027	-4.38580
C	2.25154	0.86528	0.19107
C	2.70693	1.71527	1.20405
C	3.17446	0.29378	-0.69011
C	4.05145	2.03023	1.30083
H	1.99834	2.14307	1.90656
C	4.51855	0.60157	-0.58082
H	2.84837	-0.41758	-1.44092
C	4.95553	1.47667	0.40548
H	4.39508	2.70676	2.07442
H	5.22819	0.16135	-1.27104
H	6.00858	1.72226	0.47957
C	-2.02620	-1.77215	-1.80560
C	-2.16557	-2.59171	-2.91937
C	-2.22852	-2.28980	-0.52582
C	-2.52151	-3.92208	-2.75402
H	-1.98611	-2.18799	-3.90886
C	-2.58157	-3.61834	-0.36851
H	-2.09663	-1.66562	0.35207
C	-2.73089	-4.43375	-1.48328
H	-2.62852	-4.55999	-3.62328
H	-2.73644	-4.02030	0.62582
H	-3.00561	-5.47465	-1.35714
S	-1.37354	0.52275	-3.92059

R = Mes

P	0.53451	0.56808	0.50897
C	0.31042	-0.02134	-1.03365
P	-1.28154	-0.28507	-1.77927
Ge	1.41450	0.20152	-2.68185
Cl	1.82325	-1.90029	-3.21317
C	-2.53233	0.68780	-0.90893
H	-2.25615	1.74139	-0.95392
H	-3.52188	0.53660	-1.33707
H	-2.54449	0.35811	0.13314
Au	3.06568	1.81379	-3.07717
Cl	4.69505	3.40088	-3.34177
C	2.28511	1.04711	0.54024
C	2.58115	2.42049	0.54295
C	3.31414	0.09696	0.60027
C	3.90821	2.81796	0.54732
C	4.62867	0.54569	0.61168
C	4.94852	1.89636	0.56035
H	4.13801	3.87796	0.50598
H	5.42927	-0.18696	0.65134
C	-1.96166	-1.96517	-1.90067
C	-3.02543	-2.16723	-2.80623
C	-1.50236	-3.03727	-1.10919
C	-3.60217	-3.42821	-2.89049
C	-2.11661	-4.27527	-1.24674
C	-3.16291	-4.49906	-2.12888
H	-4.42288	-3.57291	-3.58624
H	-1.75829	-5.09565	-0.63308
C	-3.56664	-1.12174	-3.73935
H	-3.61875	-0.11986	-3.31385
H	-2.94234	-1.04633	-4.63327
H	-4.57389	-1.39601	-4.05509
C	-0.38175	-2.93795	-0.11978
H	0.54844	-2.63424	-0.60154
H	-0.59111	-2.21646	0.67280
H	-0.21866	-3.90854	0.34893
C	-3.77719	-5.85588	-2.27062
H	-3.21376	-6.45678	-2.99117
H	-3.77276	-6.39622	-1.32241
H	-4.80574	-5.79318	-2.62903
C	3.03895	-1.37729	0.64600
H	2.17882	-1.61370	1.27904
H	2.83112	-1.77778	-0.35100
H	3.90176	-1.91558	1.04079
C	1.49631	3.45265	0.46531
H	1.12183	3.54697	-0.55904
H	0.64355	3.20227	1.10605
H	1.86786	4.43174	0.76970
C	6.36901	2.35578	0.47725
H	6.59251	2.66995	-0.54710
H	6.55003	3.21379	1.12840
H	7.06537	1.56111	0.74855
S	-0.77095	0.49359	-3.59592

R = Mes*

P	-0.22934	1.82487	1.18770
C	0.22279	0.47305	0.31926
P	1.82346	-0.30819	0.17758
Cl	-1.26759	0.57189	-2.69150
C	3.52768	0.37237	0.20894
C	4.17345	0.61620	-1.03835
C	4.34236	0.13377	1.34929
C	5.49041	0.19942	-1.16921
C	5.65926	-0.26137	1.13545
C	6.22961	-0.34159	-0.12533
H	5.96556	0.32386	-2.13428
H	6.26425	-0.49880	1.99957
C	-2.05010	1.83947	0.97126
C	-2.64147	2.72825	0.04448
C	-2.85454	0.88411	1.64518
C	-3.92803	2.43693	-0.40013
C	-4.12338	0.64507	1.13453
C	-4.65641	1.35244	0.06440
H	-4.37340	3.07431	-1.14706
H	-4.72834	-0.12327	1.59477
C	-1.98354	4.05291	-0.38956
C	-2.47353	0.21364	2.98598
C	-6.01313	0.95886	-0.50884
C	3.98274	0.47640	2.81113
C	3.63181	1.49033	-2.18730

C	7.62895	-0.88942	-0.37356
C	-3.73962	-0.09037	3.80120
H	-3.44521	-0.40452	4.80554
H	-4.38394	0.78695	3.89390
H	-4.32278	-0.90721	3.37351
C	-1.64065	1.16697	3.84687
H	-0.64520	1.36596	3.43064
H	-2.14112	2.12897	3.98017
H	-1.47485	0.72322	4.83229
C	-1.72182	-1.10894	2.82356
H	-2.29996	-1.82494	2.23585
H	-0.76059	-0.96754	2.33191
H	-1.53977	-1.54974	3.80861
C	-7.08210	1.02017	0.58855
H	-8.05504	0.73738	0.17742
H	-6.86146	0.33752	1.41122
H	-7.16651	2.02972	0.99950
C	-6.44675	1.87585	-1.64952
H	-7.40806	1.53576	-2.04180
H	-6.57407	2.91042	-1.31895
H	-5.73033	1.86131	-2.47459
C	-5.91853	-0.47341	-1.04939
H	-5.17159	-0.54228	-1.84371
H	-5.63896	-1.19042	-0.27526
H	-6.88349	-0.78298	-1.46067
C	-2.91505	4.89270	-1.26662
H	-3.85927	5.11931	-0.76665
H	-2.42393	5.84277	-1.48970
H	-3.13279	4.40483	-2.21953
C	-1.69098	4.87756	0.87277
H	-2.60588	5.05216	1.44395
H	-0.97330	4.38194	1.53081
H	-1.26965	5.84830	0.59600
C	-0.68989	3.86676	-1.18160
H	0.10483	3.41580	-0.58123
H	-0.84102	3.23477	-2.05677
H	-0.31745	4.84213	-1.50895
C	2.61009	1.11454	2.99144
H	2.48303	1.97334	2.32969
H	1.76994	0.44116	2.83360
H	2.51783	1.47907	4.01744
C	4.98500	1.57380	3.23107
H	4.74980	1.90714	4.24540
H	6.01577	1.21938	3.22518
H	4.91968	2.43729	2.56526
C	4.15713	-0.70398	3.77156
H	5.17431	-1.09876	3.74225
H	3.96390	-0.37410	4.79557
H	3.47986	-1.53001	3.55489
C	2.26173	2.09897	-1.92048
H	2.06073	2.86348	-2.67478
H	1.44904	1.37932	-1.98606
H	2.23197	2.58747	-0.94350
C	3.62693	0.77468	-3.53983
H	2.91983	-0.05363	-3.54979
H	3.33391	1.48018	-4.32166
H	4.61439	0.38750	-3.79997
C	4.60511	2.68582	-2.27524
H	4.65282	3.22438	-1.32546
H	5.61795	2.38365	-2.54368
H	4.24795	3.37915	-3.04110
C	8.26663	-1.44386	0.89746
H	8.42452	-0.66643	1.64945
H	7.66091	-2.23750	1.34211
H	9.24432	-1.86852	0.65876
C	8.53088	0.22275	-0.92224
H	8.15799	0.62036	-1.86808
H	8.60669	1.05184	-0.21447
H	9.53717	-0.16603	-1.09957
C	7.53665	-2.02264	-1.40274
H	6.91075	-2.83838	-1.03311
H	7.11226	-1.67740	-2.34749
H	8.53244	-2.42401	-1.60874
Ge	-0.73984	-0.72115	-0.99629
Au	-2.23623	-2.45952	-0.50204
Cl	-3.73144	-4.09929	0.07373
S	1.49085	-1.28144	-1.59073
C	1.83236	-1.69945	1.34572
H	1.84333	-1.40815	2.39070
H	0.92493	-2.27010	1.13917
H	2.69795	-2.32264	1.11704

Coordination through S atom

R= H

P	0.53048	0.29204	0.66447
C	0.32219	0.31560	-0.98108
P	-1.17988	0.63163	-1.82089
Ge	1.64395	0.35904	-2.58722
Cl	0.94469	-1.58491	-3.42000
C	-2.57101	1.32231	-0.92136
H	-2.28125	2.27535	-0.47830
H	-3.39665	1.47588	-1.61957
H	-2.87642	0.62431	-0.13816
Au	-1.90789	1.42868	-5.01356
Cl	-3.40531	1.09029	-6.66138
H	-1.63290	-0.58277	-2.35987
H	1.94817	0.08967	0.67930
S	-0.44939	1.82150	-3.30783

R = Me

P	0.51695	0.21422	0.63591
C	0.30736	0.35554	-1.00325
P	-1.26055	0.56368	-1.75489
Ge	1.59378	0.66325	-2.60671
Cl	1.41868	-1.39640	-3.45746
C	-2.51007	1.42371	-0.78619
H	-2.12591	2.39798	-0.48476
H	-3.40419	1.55630	-1.39913
H	-2.75665	0.83511	0.10050
C	-1.96328	-1.01686	-2.23499
H	-2.19499	-1.58241	-1.32891
H	-2.86775	-0.84950	-2.82272
H	-1.22827	-1.55691	-2.83258
C	2.32609	0.00073	0.85278
H	2.53901	-1.07053	0.92308
H	2.90886	0.42044	0.03076
H	2.63319	0.45969	1.79458
Au	-1.99513	1.06592	-5.11027
Cl	-3.37293	0.49923	-6.80224
S	-0.66502	1.68633	-3.36847

R = *t*-Bu

P	0.65973	-0.24848	0.47361
C	0.19729	0.47478	-0.95169
P	-1.50630	0.38108	-1.42214
Ge	0.98242	1.84618	-2.32243
Cl	1.66483	0.37100	-3.85817
Au	-2.46963	1.40005	-4.79533
Cl	-3.41336	0.96734	-6.79875
C	-2.05101	-1.27626	-2.06302

C	-3.51029	-1.18634	-2.50845
C	-1.15916	-1.70050	-3.22440
C	-1.92475	-2.28411	-0.91887
H	-4.18660	-0.96962	-1.67909
H	-3.65850	-0.44228	-3.29473
H	-3.79840	-2.15624	-2.92247
H	-0.10973	-1.76309	-2.93409
H	-1.48417	-2.68870	-3.56123
H	-1.23633	-1.01703	-4.07158
H	-2.22388	-3.26269	-1.30428
H	-0.90068	-2.37171	-0.55224
H	-2.57863	-2.05091	-0.07630
C	2.50038	-0.06405	0.72940
C	3.05098	-1.49113	0.62504
C	3.22097	0.85013	-0.24316
C	2.66521	0.44239	2.16519
H	2.53321	-2.18010	1.29849
H	2.96950	-1.87905	-0.39306
H	4.11029	-1.49098	0.90239
H	2.84923	1.87649	-0.18954
H	4.28868	0.88124	0.00065
H	3.14031	0.49122	-1.27284
H	3.72600	0.43704	2.43487
H	2.29620	1.46506	2.27755
H	2.13589	-0.19013	2.88309
S	-1.55091	1.93718	-2.78057
C	-2.61332	0.83130	-0.06645
H	-2.52870	0.10869	0.74659
H	-2.31125	1.81462	0.29419
H	-3.64251	0.88146	-0.42326

R = Ph

P	0.42790	0.78655	0.36118
C	0.40889	0.28157	-1.22508
P	-1.03938	0.34346	-2.20474
Ge	1.91607	0.08074	-2.63930
Cl	1.79049	-2.13455	-2.93489
C	-2.25901	1.58608	-1.73922
H	-1.78083	2.56563	-1.72736
H	-3.06959	1.58608	-2.47016
H	-2.66042	1.35001	-0.75156
Au	-1.30446	-0.09414	-5.70267
Cl	-2.49942	-0.96692	-7.40909
C	-1.89854	-1.23433	-2.22290
C	-1.42728	-2.29717	-1.45847
C	-3.05060	-1.38322	-2.99587
C	-2.11233	-3.50229	-1.46318
H	-0.52249	-2.18648	-0.87533
C	-3.72459	-2.59089	-2.99887
H	-3.41187	-0.57315	-3.61960
C	-3.25654	-3.64927	-2.23125
H	-1.74046	-4.33146	-0.87318
H	-4.60834	-2.70846	-3.61406
H	-3.78376	-4.59628	-2.24082
C	2.13300	0.53259	0.89797
C	2.61493	1.38896	1.89211
C	2.97012	-0.47043	0.39858
C	3.91992	1.27788	2.34200
H	1.96340	2.15342	2.30428
C	4.27142	-0.58513	0.85879
H	2.58997	-1.19296	-0.31697
C	4.74908	0.29307	1.82254
H	4.29002	1.95554	3.10255
H	4.91224	-1.36755	0.46924
H	5.76813	0.20023	2.18025
S	-0.15715	0.87091	-3.99063

R = Mes

P	0.21167	0.53470	0.49646
C	0.11491	0.06811	-1.09549
P	-1.28700	0.02368	-2.15867
Ge	1.72697	0.07540	-2.38366
Cl	1.69030	-2.14007	-2.85079
C	-2.64857	1.14446	-1.76367
H	-2.23510	2.13469	-1.56737
H	-3.30204	1.19315	-2.63726
H	-3.21553	0.79509	-0.90332
Au	-0.00183	2.92807	-3.20475
Cl	0.40097	5.06879	-2.59947
C	-1.99457	-1.64968	-2.32970
C	-2.58113	-2.19764	-1.16754
C	-1.99072	-2.40786	-3.52454
C	-3.11286	-3.48116	-1.21358
C	-2.55092	-3.67828	-3.50297
C	-3.10629	-4.24504	-2.36572
H	-3.54967	-3.88781	-0.30653
H	-2.54286	-4.25154	-4.42435
C	2.01248	0.52242	0.79659
C	2.69172	1.74950	0.86277
C	2.68504	-0.68300	1.05524
C	4.05071	1.74394	1.14644
C	4.04469	-0.63497	1.33655
C	4.74715	0.56356	1.37596
H	4.57881	2.69188	1.19141
H	4.56846	-1.56421	1.54073
C	1.98142	3.04429	0.60978
H	1.78639	3.20382	-0.45529
H	1.01087	3.08003	1.11658
H	2.57151	3.89120	0.96163
C	1.97326	-2.00403	1.05210
H	1.04218	-1.95988	1.62599
H	1.71222	-2.31996	0.03820
H	2.60145	-2.77900	1.49316
C	6.22013	0.58205	1.64602
H	6.78377	0.54085	0.70866
H	6.51862	1.49450	2.16532
H	6.52618	-0.27430	2.24938
C	-2.72909	-1.48975	0.14961
H	-1.98503	-0.71946	0.34013
H	-3.71839	-1.02759	0.22731
H	-2.65245	-2.21061	0.96495
C	-1.42928	-1.96519	-4.84316
H	-1.87608	-1.03621	-5.19821
H	-0.34965	-1.81794	-4.78895
H	-1.62187	-2.73413	-5.59143
C	-3.65681	-5.63573	-2.38689
H	-4.30762	-5.82151	-1.53155
H	-4.22456	-5.82323	-3.30054
H	-2.84553	-6.36920	-2.35357
S	-0.36422	0.75778	-3.86615

R = Mes*

P	-1.99909	1.86324	0.37895
C	-1.05993	0.49325	0.26419
P	0.70144	0.36263	0.36902
C	-3.70665	1.16953	0.36470
C	-4.49038	1.28214	-0.81390
C	-4.19690	0.41759	1.45652
C	-5.56789	0.42182	-0.95781
C	-5.27575	-0.43699	1.22536
C	-5.93341	-0.50654	0.01064
H	-6.14537	0.46122	-1.86964

H	-5.62050	-1.05932	2.03730
C	-3.71745	0.56214	2.91951
C	-4.25660	2.36445	-1.88551
C	-7.05715	-1.49833	-0.26945
C	-2.94996	2.16733	-2.65443
H	-2.07121	2.28949	-2.01794
H	-2.89494	1.17307	-3.09962
H	-2.87419	2.91665	-3.44815
C	-4.25783	3.74222	-1.20779
H	-5.20006	3.91503	-0.68196
H	-3.44428	3.85330	-0.48788
H	-4.13834	4.52650	-1.96058
C	-5.37771	2.38693	-2.92767
H	-5.40177	1.47425	-3.52761
H	-6.36053	2.53432	-2.47384
H	-5.20137	3.22026	-3.61161
C	-2.66342	-0.47945	3.30162
H	-1.73501	-0.32494	2.75335
H	-2.44162	-0.41121	4.37102
H	-3.01495	-1.49220	3.09286
C	-3.17717	1.96460	3.20343
H	-2.24619	2.18667	2.67107
H	-3.90477	2.73426	2.93568
H	-2.95087	2.06213	4.26872
C	-4.90717	0.38022	3.87732
H	-5.75010	1.01632	3.59817
H	-5.25660	-0.65211	3.92440
H	-4.59361	0.65385	4.88769
C	-7.30135	-2.44338	0.90391
H	-7.62881	-1.90833	1.79923
H	-8.08875	-3.15382	0.64120
H	-6.40645	-3.01923	1.15287
C	-6.67934	-2.34050	-1.49433
H	-6.53739	-1.72137	-2.38216
H	-5.74996	-2.88810	-1.32244
H	-7.47060	-3.06351	-1.71154
C	-8.35827	-0.73968	-0.55587
H	-8.26118	-0.07889	-1.41960
H	-9.16640	-1.44574	-0.76647
H	-8.65361	-0.13061	0.30224
C	2.08557	1.48157	-0.05491
C	2.71244	1.30580	-1.32275
C	2.85305	2.04127	1.00227
C	4.09840	1.28881	-1.35282
C	4.23793	1.98998	0.89359
C	4.88965	1.50837	-0.23218
H	4.58255	1.09538	-2.30176
H	4.82758	2.35293	1.72440
C	2.00755	1.34281	-2.69250
C	2.36171	0.15541	-3.59063
C	2.52700	2.63107	-3.36664
H	1.99273	-0.78287	-3.17856
H	1.90429	0.29723	-4.57332
H	3.43877	0.06132	-3.74079
H	2.29803	3.51121	-2.76048
H	3.60458	2.60625	-3.53262
H	2.03808	2.74936	-4.33733
C	2.29851	2.88664	2.16738
C	2.90897	4.29218	1.98025
C	2.72465	2.37500	3.54658
H	2.65786	4.69930	0.99822
H	2.50576	4.96495	2.74209
H	3.99469	4.28786	2.07690
H	2.31410	1.39259	3.77818
H	3.81055	2.30316	3.62913
H	2.38212	3.07056	4.31729
C	6.39683	1.30920	-0.30166
C	6.99871	2.28170	-1.32321
C	6.67974	-0.13379	-0.73954
H	6.80936	3.32011	-1.03912
H	8.08075	2.13689	-1.38190
H	6.58900	2.12513	-2.32316
H	6.25222	-0.85722	-0.04145
H	6.26527	-0.34479	-1.72737
H	7.75911	-0.30082	-0.78879
Cl	-2.34693	-1.45288	-2.06868
Au	2.67634	-2.49329	0.05883
Cl	4.52856	-3.54639	0.82201
C	0.78981	3.10436	2.12002
H	0.47599	3.50023	1.15212
H	0.19774	2.21421	2.32343
H	0.51031	3.83763	2.88062
C	0.49417	1.49728	-2.62180
H	-0.02490	0.59432	-2.30348
H	0.21298	2.32195	-1.96207
H	0.11688	1.73347	-3.61974
C	7.07090	1.54417	1.04742
H	6.96695	2.57889	1.38539
H	6.66913	0.88272	1.81882
H	8.13976	1.33727	0.95934
Ge	-1.56134	-1.53346	0.02806
S	0.87753	-1.36586	-0.76895
C	1.03500	-0.25109	2.04657
H	2.09633	-0.48452	2.13560
H	0.72638	0.44828	2.81824
H	0.46548	-1.17595	2.15381

Coordination through P(sp^2) atom

R= H

P	0.46370	0.28087	0.46656
C	0.20187	0.15525	-1.14340
P	-1.31374	0.20301	-2.02218
Ge	1.48288	0.27385	-2.80796
Cl	1.19473	-1.89368	-3.32034
C	-2.73110	0.94877	-1.21023
H	-2.49982	1.98338	-0.95562
H	-3.57778	0.92385	-1.89979
H	-2.97892	0.39037	-0.30441
Au	-0.90253	0.44803	2.18593
Cl	-2.34596	0.62392	3.91094
H	1.86818	0.28826	0.62150
H	-1.68961	-1.13642	-2.23961
S	-0.73083	1.10583	-3.70506

R = Me

P	0.50492	0.35272	0.43452
C	0.17466	0.23970	-1.16264
P	-1.40000	0.08397	-1.92511
Ge	1.35047	0.38183	-2.88996
Cl	1.57884	-1.85502	-3.09784
C	-2.70015	1.10350	-1.21096
H	-2.38354	2.14634	-1.22217
H	-3.60181	0.99410	-1.81769
H	-2.90716	0.79055	-0.18490
C	-1.95466	-1.62332	-1.81330
H	-2.15825	-1.87734	-0.77051
H	-2.85961	-1.74498	-2.41194
H	-1.16301	-2.26148	-2.20841
C	2.28681	0.46292	0.72161
H	2.61650	-0.45426	1.21622

H	2.82979	0.58857	-0.21548
H	2.49073	1.29903	1.39329
Au	-0.90904	0.33785	2.13290
Cl	-2.40817	0.30852	3.82605
S	-0.95057	0.62597	-3.80732

R = *t*-Bu

P	0.51129	0.42466	0.30127
C	0.29935	0.12630	-1.30088
P	-1.27538	-0.11810	-2.07489
Ge	1.47345	0.39252	-3.03603
Cl	2.23824	-1.72106	-3.22833
C	-2.49893	1.10509	-1.55773
H	-2.05969	2.09302	-1.69768
H	-3.38250	1.01841	-2.19161
H	-2.77396	0.97577	-0.50952
Au	-1.06124	0.65698	1.85474
Cl	-2.69865	0.92254	3.40064
C	2.26472	0.60537	0.86561
C	2.36185	1.95322	1.58484
C	2.49826	-0.53270	1.86566
C	3.26975	0.52724	-0.27122
H	2.20938	2.79002	0.89930
H	1.63441	2.03649	2.39595
H	3.36132	2.04943	2.01918
H	2.45762	-1.51050	1.38047
H	3.49343	-0.41396	2.30486
H	1.76943	-0.51763	2.67984
H	4.27971	0.59999	0.14425
H	3.20489	-0.41720	-0.81552
H	3.15044	1.35184	-0.97800
C	-1.99993	-1.80325	-1.79129
C	-0.98904	-2.85632	-2.23336
C	-2.35001	-1.99803	-0.31934
C	-3.26077	-1.89837	-2.65303
H	-0.71897	-2.74497	-3.28405
H	-0.06956	-2.81303	-1.64801
H	-1.43699	-3.84447	-2.09496
H	-3.07746	-1.27164	0.04763
H	-2.79229	-2.99140	-0.20007
H	-1.46749	-1.95618	0.32128
H	-3.64768	-2.91867	-2.58395
H	-4.04996	-1.22556	-2.31125
H	-3.04981	-1.69286	-3.70482
S	-0.79302	0.15657	-4.01298

R = Ph

P	0.48849	0.48543	0.20189
C	0.31778	0.06438	-1.37497
P	-1.21220	-0.19317	-2.20910
Ge	1.60868	0.02358	-3.01580
Cl	2.06901	-2.19142	-2.94139
C	-2.46222	1.05651	-1.86039
H	-2.04686	2.03193	-2.11474
H	-3.33366	0.85511	-2.48645
H	-2.75288	1.04913	-0.80839
Au	-1.11781	0.94560	1.65849
Cl	-2.82869	1.37886	3.07869
C	2.19899	0.57851	0.69828
C	3.19944	-0.16478	0.06617
C	2.52337	1.42091	1.76441
C	4.51291	-0.03756	0.48387
H	2.94857	-0.87138	-0.71747
C	3.84057	1.54639	2.16969
H	1.74097	1.97828	2.26922
C	4.83458	0.82074	1.52717
H	5.28697	-0.61975	-0.00194
H	4.09135	2.20674	2.99131
H	5.86513	0.91470	1.85004
C	-1.92427	-1.79109	-1.77179
C	-2.70557	-1.91889	-0.62270
C	-1.65607	-2.90519	-2.56105
C	-3.22069	-3.15633	-0.27562
H	-2.91194	-1.06486	0.01276
C	-2.17501	-4.14024	-2.20566
H	-1.03893	-2.80029	-3.44537
C	-2.95680	-4.26587	-1.06728
H	-3.82870	-3.25182	0.61620
H	-1.96422	-5.00670	-2.82124
H	-3.36192	-5.23312	-0.79259
S	-0.61390	-0.10493	-4.12559

R = Mes

P	0.52009	0.53305	0.64793
C	0.40454	-0.24155	-0.79442
P	-0.99509	-0.43515	-1.85670
Ge	1.97635	-0.43169	-2.14756
Cl	1.94746	-2.69934	-2.12797
C	-2.27483	0.78024	-1.46826
H	-1.84290	1.78020	-1.51426
H	-3.10340	0.70100	-2.17116
H	-2.64868	0.58774	-0.45937
Au	-1.07989	1.19059	2.03155
Cl	-2.73150	1.84097	3.43902
C	2.25380	0.80117	1.06139
C	3.01254	-0.23464	1.62323
C	2.80627	2.07693	0.86014
C	4.33238	0.02952	1.96811
C	4.12829	2.28707	1.21957
C	4.90781	1.27816	1.77609
H	4.92657	-0.76820	2.40304
H	4.56349	3.26890	1.05937
C	-1.84865	-2.04215	-1.87802
C	-1.76275	-2.97194	-0.82215
C	-2.67524	-2.32002	-2.98813
C	-2.48747	-4.15291	-0.91245
C	-3.38034	-3.51740	-3.01380
C	-3.29733	-4.45351	-1.99669
H	-2.41517	-4.86158	-0.09356
H	-4.01690	-3.71939	-3.86970
C	2.00731	3.18784	0.24927
H	1.75484	2.96701	-0.79245
H	1.06676	3.35153	0.78515
H	2.56668	4.12347	0.26547
C	2.44474	-1.60348	1.84383
H	1.43808	-1.56183	2.27080
H	2.37970	-2.15923	0.90318
H	3.07264	-2.17654	2.52678
C	6.32444	1.54514	2.18042
H	6.35888	2.09734	3.12454
H	6.88140	0.61762	2.31833
H	6.84239	2.14948	1.43282
C	-0.93417	-2.77663	0.40969
H	0.12288	-2.67986	0.15915
H	-1.23112	-1.88800	0.97154
H	-1.04942	-3.63612	1.07030
C	-2.84046	-1.43003	-4.18750
H	-2.88655	-0.36666	-3.95502
H	-2.00625	-1.55798	-4.88116
H	-3.76062	-1.69188	-4.71142
C	-4.03897	-5.75088	-2.07117
H	-4.90430	-5.67881	-2.73167

H	-3.39016	-6.54074	-2.46218
H	-4.38180	-6.07017	-1.08526
S	-0.01253	-0.01548	-3.57672

R = Mes*

P	-1.08895	0.76522	0.08690
C	-0.32615	-0.64996	0.43387
P	1.35210	-1.14820	0.71706
Cl	-1.90963	-2.91871	-1.50395
C	2.97605	-0.48954	0.16082
C	3.52686	-1.04757	-1.03045
C	3.86928	0.07586	1.11251
C	4.87651	-1.36759	-1.02579
C	5.20758	-0.30224	1.04837
C	5.72212	-1.10423	0.04333
H	5.28698	-1.83773	-1.91047
H	5.87505	0.07245	1.81176
C	-2.87546	0.42237	0.13426
C	-3.61489	0.23249	-1.06373
C	-3.45600	0.08792	1.38376
C	-4.78593	-0.51184	-0.98117
C	-4.61335	-0.68267	1.36860
C	-5.26382	-1.05143	0.20340
H	-5.33281	-0.70712	-1.88835
H	-5.02764	-1.00170	2.31633
C	-3.26050	0.87679	-2.41769
C	-3.01456	0.58652	2.77826
C	-6.47188	-1.97963	0.26142
C	3.56116	1.22507	2.09703
C	2.82955	-1.15241	-2.40170
C	7.16216	-1.60041	0.02778
C	-4.24263	1.23181	3.44925
H	-3.95341	1.63007	4.42521
H	-4.62710	2.05750	2.84583
H	-5.05757	0.52720	3.61220
C	-1.94614	1.67782	2.77426
H	-0.95170	1.30566	2.52704
H	-2.19337	2.49617	2.09333
H	-1.87247	2.10343	3.77878
C	-2.52924	-0.57473	3.64927
H	-3.28778	-1.35389	3.74502
H	-1.63526	-1.03602	3.22546
H	-2.28701	-0.21446	4.65329
C	-7.57232	-1.34863	1.12213
H	-8.43860	-2.01418	1.17001
H	-7.23663	-1.16795	2.14535
H	-7.89959	-0.39424	0.70222
C	-7.04932	-2.26702	-1.12196
H	-7.89239	-2.95550	-1.02723
H	-7.41961	-1.36014	-1.60704
H	-6.31180	-2.73456	-1.77865
C	-6.03441	-3.31079	0.88531
H	-5.24719	-3.77947	0.29020
H	-5.65131	-3.17539	1.89896
H	-6.88311	-3.99854	0.93661
C	-4.27179	0.51482	-3.50981
H	-5.28607	0.83501	-3.26121
H	-3.98519	1.03074	-4.42868
H	-4.28130	-0.55676	-3.72328
C	-3.32191	2.40097	-2.24675
H	-4.31888	2.71374	-1.92625
H	-2.60344	2.76533	-1.50909
H	-3.09630	2.89456	-3.19575
C	-1.89226	0.45773	-2.95740
H	-1.06142	0.82514	-2.35262
H	-1.80721	-0.62706	-3.02512
H	-1.75087	0.88764	-3.95309
C	2.17806	1.83939	1.91730
H	2.06436	2.22770	0.90357
H	1.34368	1.16960	2.11010
H	2.06458	2.68582	2.59868
C	4.53247	2.37018	1.73276
H	4.28303	3.25165	2.32909
H	5.57357	2.11660	1.93319
H	4.44089	2.64078	0.67907
C	3.82287	0.85688	3.56124
H	4.84207	0.49078	3.70150
H	3.70454	1.74576	4.18644
H	3.14499	0.09284	3.93909
C	1.38492	-0.67392	-2.42581
H	1.06327	-0.58589	-3.46638
H	0.69636	-1.36627	-1.94438
H	1.28636	0.31570	-1.97189
C	2.90092	-2.55946	-3.00023
H	2.33872	-3.27233	-2.39760
H	2.46820	-2.54666	-4.00404
H	3.92823	-2.91813	-3.09089
C	3.60556	-0.18539	-3.32123
H	3.57413	0.83539	-2.93258
H	4.65153	-0.47069	-3.44042
H	3.14178	-0.18527	-4.31114
C	7.91743	-1.22409	1.29958
H	8.01858	-0.14146	1.41046
H	7.42782	-1.61891	2.19344
H	8.92566	-1.64301	1.26174
C	7.90297	-0.99661	-1.17140
H	7.44119	-1.27961	-2.11927
H	7.91541	0.09457	-1.11577
H	8.93803	-1.34865	-1.18759
C	7.15580	-3.12887	-0.09610
H	6.64406	-3.58876	0.75274
H	6.65304	-3.45858	-1.00732
H	8.18117	-3.50755	-0.12222
Au	-0.18965	2.74069	-0.41920
Cl	0.75032	4.74833	-0.92422
Ge	-1.17409	-2.59744	0.60361
S	1.20050	-3.07789	0.10774
C	1.44691	-1.34512	2.52415
H	0.64420	-2.03959	2.77874
H	2.39958	-1.81974	2.76162
H	1.32241	-0.42978	3.09361

Coordination through π(C=P) bond

R= H

P	0.37679	-0.07447	0.68499
C	0.21246	0.55587	-0.91295
P	-1.25666	0.62144	-1.89331
Ge	1.54949	0.84041	-2.52697
Cl	1.33378	-1.30380	-3.19227
C	-2.77743	1.19493	-1.12210
H	-2.65762	2.23081	-0.80316
H	-3.58698	1.12949	-1.85231
H	-3.01010	0.56423	-0.26070
Au	0.34147	2.28250	0.37300
Cl	0.41231	4.39704	1.13555
H	1.80271	-0.18061	0.69889
H	-1.53126	-0.71336	-2.24777
S	-0.64309	1.70001	-3.45441

R = Me

P	0.45301	0.02967	0.63598
C	0.16700	0.68727	-0.93119
P	-1.34971	0.56725	-1.84106
Ge	1.42769	1.09208	-2.57482
Cl	1.74727	-1.10482	-3.05059
C	-2.79466	1.34774	-1.09742
H	-2.61037	2.41445	-0.96983
H	-3.64992	1.20637	-1.76227
H	-3.00604	0.89162	-0.12761
C	-1.76896	-1.17300	-2.03310
H	-2.03168	-1.60156	-1.06323
H	-2.61161	-1.26138	-2.72144
H	-0.90106	-1.68713	-2.44877
C	2.27471	-0.15836	0.71443
H	2.50076	-1.18400	0.40453
H	2.81703	0.52954	0.06521
H	2.60441	-0.03274	1.74735
Au	0.19369	2.39223	0.39085
Cl	0.13641	4.46872	1.26329
S	-0.82666	1.41691	-3.58217

R = *t*-Bu

P	0.58789	0.05346	0.41309
C	0.10983	0.74679	-1.09681
P	-1.50456	0.44289	-1.81725
Ge	1.02403	1.63711	-2.79284
Cl	2.02046	-0.22159	-3.62655
C	-2.85577	1.25314	-0.92831
H	-2.68680	2.32979	-0.95048
H	-3.79906	1.03596	-1.43300
H	-2.90151	0.91259	0.10688
Au	0.00613	2.36509	0.35554
Cl	-0.22095	4.34693	1.40888
C	-1.95463	-1.35703	-1.92151
C	-3.13162	-1.48069	-2.89094
C	-0.76712	-2.14592	-2.46312
C	-2.35720	-1.87596	-0.54148
H	-4.02015	-0.94893	-2.54320
H	-2.87366	-1.11558	-3.88628
H	-3.39647	-2.53835	-2.97562
H	0.08314	-2.13024	-1.77974
H	-1.07335	-3.18760	-2.59534
H	-0.42751	-1.76267	-3.42612
H	-2.55165	-2.94953	-0.61979
H	-1.56808	-1.73747	0.20101
H	-3.26987	-1.40457	-0.17193
C	2.44928	0.04719	0.57620
C	2.85935	-1.35331	0.10412
C	3.21587	1.11075	-0.19091
C	2.71000	0.18621	2.07805
H	2.30661	-2.14077	0.62583
H	2.71158	-1.47064	-0.97170
H	3.92226	-1.50415	0.31886
H	2.89641	2.12083	0.07547
H	4.28170	1.02751	0.04837
H	3.12651	0.97932	-1.27083
H	3.77602	0.03803	2.27491
H	2.43511	1.17839	2.44479
H	2.15658	-0.55658	2.65933
S	-1.27396	1.26416	-3.64113

R = Ph

P	0.33359	0.49628	0.48547
C	0.20083	0.66566	-1.22685
P	-1.33025	0.48613	-2.10726
Ge	1.46094	0.84036	-2.92385
Cl	1.69367	-1.37032	-3.37153
C	-2.73813	1.38542	-1.42363
H	-2.52033	2.45376	-1.42699
H	-3.61030	1.18325	-2.04868
H	-2.93906	1.05344	-0.40306
Au	0.33978	2.69552	-0.44911
Cl	0.46323	4.93242	-0.19107
C	-1.83217	-1.24895	-2.10636
C	-2.22949	-1.84512	-0.90946
C	-1.83962	-1.98383	-3.28547
C	-2.63739	-3.16773	-0.89943
H	-2.21103	-1.28630	0.02073
C	-2.25265	-3.30761	-3.26926
H	-1.50987	-1.51904	-4.20676
C	-2.65113	-3.89828	-2.08057
H	-2.94246	-3.63023	0.03193
H	-2.25377	-3.87960	-4.18955
H	-2.96935	-4.93448	-2.07039
C	2.08943	0.24268	0.84776
C	2.96378	-0.44885	0.00836
C	2.54449	0.70591	2.08537
C	4.28066	-0.64416	0.39305
H	2.61205	-0.86258	-0.93018
C	3.86488	0.52132	2.45674
H	1.86325	1.22624	2.75160
C	4.73407	-0.15289	1.60932
H	4.95424	-1.18467	-0.26185
H	4.21616	0.89942	3.40972
H	5.76664	-0.30383	1.90283
S	-0.81289	1.15658	-3.92397

R = Mes

P	0.25447	0.42776	1.04715
C	-0.08205	0.71087	-0.62967
P	-1.53630	0.55171	-1.65841
Ge	1.29215	1.38155	-2.09184
Cl	1.76288	-0.59031	-3.11766
C	-3.11097	1.19443	-1.03245
H	-2.98535	2.25084	-0.79220
H	-3.83556	1.09552	-1.84473
H	-3.47202	0.65326	-0.16074
Au	-0.30713	2.64139	0.36278
Cl	-0.73387	4.81305	0.83765
C	-1.83994	-1.19815	-2.08936
C	-2.17974	-2.07393	-1.03522
C	-1.76135	-1.70575	-3.40858
C	-2.35089	-3.42764	-1.29926
C	-1.95721	-3.06740	-3.60470
C	-2.22857	-3.95288	-2.57304
H	-2.59880	-4.08608	-0.47209
H	-1.89108	-3.44774	-4.61898
C	2.07781	0.29747	1.10106
C	2.60968	-0.93761	0.68090
C	2.91408	1.26055	1.68775
C	3.97916	-1.14148	0.77068
C	4.27916	1.00687	1.75055
C	4.83583	-0.17427	1.28020
H	4.38518	-2.09354	0.44214
H	4.92600	1.75819	2.19381
C	2.39833	2.54732	2.25603
H	2.19795	3.28701	1.47483
H	1.46810	2.40904	2.81340
H	3.13104	2.98389	2.93633

C	1.73688	-2.05190	0.18288
H	0.88119	-2.22129	0.84578
H	1.34818	-1.84656	-0.81726
H	2.30298	-2.98287	0.13354
C	6.31495	-0.40169	1.32301
H	6.78621	0.18328	2.11459
H	6.55221	-1.45500	1.48393
H	6.77635	-0.10485	0.37591
C	-2.45176	-1.65778	0.38297
H	-2.15195	-2.45096	1.06950
H	-1.93812	-0.75425	0.70272
H	-3.52536	-1.49981	0.52865
C	-1.50891	-0.89749	-4.64753
H	-2.19841	-0.05915	-4.74555
H	-0.49840	-0.48933	-4.66177
H	-1.63009	-1.53860	-5.52136
C	-2.37673	-5.41890	-2.83169
H	-2.89253	-5.60556	-3.77557
H	-1.39440	-5.89653	-2.89903
H	-2.93000	-5.91260	-2.03148
S	-0.93745	1.81378	-3.11335

R = Mes*

P	0.70686	-0.02716	1.03587
C	0.42586	0.14718	-0.68284
P	-1.12795	-0.04551	-1.64941
Cl	2.93137	-0.96013	-2.97015
Au	0.27974	2.17230	0.11541
Cl	-0.16518	4.40129	0.23899
C	2.55565	-0.06817	1.18253
C	3.28734	-1.19988	0.73071
C	3.28518	1.07299	1.60328
C	4.60930	-1.01527	0.35465
C	4.60283	1.19659	1.16150
C	5.26440	0.20499	0.45884
H	5.15421	-1.86729	-0.03142
H	5.13577	2.10327	1.41197
C	2.86562	2.09412	2.68386
C	2.82825	-2.66599	0.85503
C	6.67175	0.37555	-0.10045
C	3.85610	1.88555	3.84945
H	3.80138	0.86276	4.23113
H	4.88748	2.07846	3.55356
H	3.60580	2.56904	4.66545
C	2.98647	3.54185	2.20297
H	2.30498	3.75898	1.37987
H	2.73987	4.22335	3.02178
H	4.00017	3.77750	1.87434
C	1.48348	1.88034	3.29493
H	1.38836	2.51505	4.18002
H	0.66487	2.16245	2.63018
H	1.33492	0.84591	3.61563
C	3.09696	-3.47955	-0.41234
H	2.58735	-3.05448	-1.27469
H	4.16045	-3.52783	-0.65086
H	2.75055	-4.50688	-0.26710
C	3.66118	-3.26442	2.00484
H	4.72965	-3.22876	1.78756
H	3.48975	-2.72148	2.93782
H	3.37982	-4.31038	2.15973
C	1.36921	-2.85499	1.25030
H	1.12901	-2.34893	2.18968
H	0.67820	-2.51885	0.47636
H	1.17525	-3.92058	1.40369
C	7.21683	1.78292	0.12701
H	6.57592	2.54271	-0.32696
H	7.32606	2.01341	1.19013
H	8.20594	1.86819	-0.32933
C	6.63571	0.10950	-1.61000
H	7.63925	0.21149	-2.03301
H	6.27190	-0.89446	-1.83587
H	5.97516	0.81596	-2.11739
C	7.62308	-0.62313	0.56898
H	7.66255	-0.46384	1.64959
H	7.31855	-1.65649	0.39154
H	8.63410	-0.50281	0.16982
C	-2.37431	-1.42674	-1.67657
C	-2.35153	-2.33036	-2.77890
C	-3.61757	-1.22831	-1.01371
C	-3.55765	-2.61484	-3.40497
C	-1.16143	-3.20697	-3.21164
C	-4.78423	-1.57421	-1.68869
C	-3.80842	-0.85741	0.47042
C	-4.78663	-2.16898	-2.94032
H	-3.52912	-3.24253	-4.28705
C	-0.77324	-3.03871	-4.68206
C	-1.62990	-4.66464	-3.00809
C	0.05740	-3.04876	-2.31877
H	-5.72843	-1.37494	-1.20109
C	-2.51533	-0.56634	1.21367
C	-4.38684	-2.12785	1.12893
C	-4.80458	0.28402	0.69720
C	-6.05738	-2.43020	-3.73834
H	-0.02119	-3.78728	-4.94491
H	-1.62970	-3.18440	-5.34443
H	-0.35256	-2.05394	-4.87528
H	-2.44220	-4.94367	-3.68016
H	-0.78971	-5.33612	-3.20394
H	-1.96623	-4.83145	-1.98172
H	0.53757	-2.07705	-2.40590
H	-0.20639	-3.23732	-1.27607
H	0.80875	-3.78722	-2.60668
H	-1.78547	-1.36422	1.06588
H	-2.05074	0.38138	0.94366
H	-2.71486	-0.50681	2.28618
H	-4.51580	-1.95433	2.20084
H	-5.35583	-2.40216	0.71055
H	-3.71035	-2.97588	0.99946
H	-5.79025	0.05590	0.28854
H	-4.92779	0.44892	1.77062
H	-4.47523	1.22491	0.25494
C	-7.29503	-1.84767	-3.06120
C	-6.26283	-3.94067	-3.90468
C	-5.91221	-1.78249	-5.12064
H	-7.49456	-2.32069	-2.09621
H	-7.20214	-0.76999	-2.90493
H	-8.16970	-2.01667	-3.69349
H	-5.43261	-4.40776	-4.43790
H	-6.35880	-4.43266	-2.93367
H	-7.17520	-4.13361	-4.47553
H	-5.77997	-0.70126	-5.03484
H	-5.05480	-2.18157	-5.66602
H	-6.80793	-1.96963	-5.71902
Ge	1.69932	0.80814	-2.26908
S	-0.33868	0.21244	-3.49826
C	-2.23786	1.39370	-1.52088
H	-2.68252	1.53441	-0.54097
H	-1.66787	2.28035	-1.79746
H	-3.02141	1.23466	-2.26456

E = Sn(II)

[R-P=C(Sn(II)Cl)-P(O)ClR](AuCl)

Coordination through Sn(II) atom

R= H

P	0.62299	-0.11936	1.17170
C	0.46874	-0.20995	-0.47570
P	-1.00898	-0.20105	-1.41453
Cl	1.76906	-2.39992	-2.79309
Sn	1.66528	-0.10131	-2.35345
O	-0.50492	0.08468	-2.81819
Au	3.24817	1.65597	-3.16505
Cl	4.67816	3.27994	-3.83372
Cl	-2.37544	1.11793	-0.80137
H	2.05016	-0.12229	1.23741
H	-1.73264	-1.40193	-1.34112

R = Me

P	0.65946	-0.14886	1.15963
C	0.48873	-0.22294	-0.48386
P	-1.00156	-0.25811	-1.40336
Cl	1.81396	-2.37672	-2.83387
Sn	1.64410	-0.07304	-2.37048
O	-0.50118	0.05217	-2.81306
C	2.46780	-0.12916	1.43822
H	2.76991	-1.12754	1.77171
H	3.04254	0.14274	0.55247
H	2.69412	0.56299	2.25265
C	-1.89561	-1.80031	-1.33073
H	-2.25224	-1.98145	-0.31621
H	-2.73826	-1.75694	-2.02276
H	-1.20560	-2.59022	-1.63613
Au	3.23474	1.67259	-3.19774
Cl	4.68802	3.28445	-3.85656
Cl	-2.33130	1.12381	-0.80231

R = *t*-Bu

P	0.72093	-0.59641	0.91863
C	0.69392	-0.19926	-0.68803
P	-0.72888	-0.20434	-1.72694
Cl	2.77861	-1.50383	-3.18190
Sn	1.93129	0.54328	-2.37694
O	-0.13138	0.25781	-3.05750
Au	3.03394	2.75363	-2.79506
Cl	4.02733	4.77494	-3.08968
C	2.47070	-0.43984	1.54790
C	2.83160	-1.83722	2.06427
C	3.49848	0.03614	0.54065
C	2.38115	0.53565	2.72692
H	2.09030	-2.21376	2.77466
H	2.92146	-2.55819	1.24806
H	3.79490	-1.79369	2.58217
H	3.27574	1.04177	0.17238
H	4.48450	0.08770	1.01363
H	3.58986	-0.65361	-0.30399
H	3.34740	0.57393	3.23946
H	2.13673	1.54816	2.39669
H	1.62943	0.22401	3.45735
C	-1.67731	-1.75612	-1.91724
C	-2.72788	-1.52955	-3.00610
C	-0.67739	-2.82631	-2.36531
C	-2.33231	-2.15127	-0.59746
H	-3.46805	-0.78453	-2.70986
H	-2.27126	-1.21469	-3.94588
H	-3.25086	-2.47376	-3.18072
H	0.09118	-3.01079	-1.61289
H	-1.22568	-3.75859	-2.52503
H	-0.18216	-2.55770	-3.29928
H	-2.84896	-3.10434	-0.73968
H	-1.59904	-2.28734	0.20097
H	-3.06781	-1.41513	-0.26990
Cl	-2.06127	1.16827	-1.07359

R = Ph

P	0.85065	-0.00992	1.38093
C	0.44323	-0.32759	-0.19516
P	-1.15545	-0.41561	-0.90099
Cl	1.62148	-2.59892	-2.60840
Sn	1.35688	-0.28760	-2.20239
O	-0.83910	-0.29604	-2.38998
Au	2.71232	1.45764	-3.38060
Cl	3.96225	3.05807	-4.39710
C	2.65268	-0.05090	1.40164
C	3.28123	0.76472	2.34662
C	3.43454	-0.84877	0.56039
C	4.66370	0.82717	2.40845
H	2.68112	1.36597	3.02257
C	4.81489	-0.79563	0.63614
H	2.96230	-1.54816	-0.12274
C	5.42960	0.05078	1.55104
H	5.14420	1.47965	3.12793
H	5.41461	-1.41914	-0.01648
H	6.51132	0.09494	1.60233
C	-2.09203	-1.89037	-0.54174
C	-2.10108	-2.90500	-1.49624
C	-2.74615	-2.05910	0.67783
C	-2.77417	-4.08671	-1.22710
H	-1.58575	-2.76600	-2.43856
C	-3.41078	-3.24384	0.93719
H	-2.74344	-1.26623	1.41722
C	-3.42523	-4.25587	-0.01500
H	-2.78742	-4.87630	-1.96884
H	-3.92338	-3.37793	1.88230
H	-3.95009	-5.18158	0.19131
Cl	-2.32001	1.10798	-0.28460

R = Mes

P	0.95963	-0.30883	1.98639
C	0.37042	-0.45452	0.44326
P	-1.21479	-0.40858	-0.31431
Cl	1.55676	-2.09253	-2.43560
Sn	1.35661	0.04571	-1.45181
O	-0.83467	0.16722	-1.67833
Au	2.82493	1.98260	-2.06638
Cl	4.20355	3.74392	-2.48678
C	2.74496	-0.13747	1.65314
C	3.31807	1.14158	1.73884
C	3.53272	-1.25583	1.34196
C	4.66871	1.28724	1.45930
C	4.88320	-1.05871	1.07728
C	5.46640	0.20186	1.11446
H	5.10674	2.27997	1.49720
H	5.49849	-1.92047	0.83651
C	-2.12965	-1.94550	-0.40249
C	-2.60431	-2.43473	-1.64139
C	-2.36873	-2.66591	0.78648
C	-3.27721	-3.64982	-1.64327
C	-3.05014	-3.87174	0.71275
C	-3.50343	-4.38940	-0.49117
H	-3.64770	-4.02612	-2.59138
H	-3.23646	-4.41824	1.63192

Cl	-2.44169	0.92717	0.59388
C	-1.91475	-2.21092	2.14000
H	-0.86311	-2.46207	2.30501
H	-2.02072	-1.13640	2.29300
H	-2.49496	-2.71143	2.91577
C	-4.20562	-5.70882	-0.55024
H	-3.49161	-6.51109	-0.76121
H	-4.69385	-5.94451	0.39651
H	-4.95632	-5.72530	-1.34209
C	-2.44976	-1.73983	-2.96264
H	-2.79439	-0.70648	-2.93357
H	-1.40610	-1.71610	-3.28034
H	-3.02456	-2.27400	-3.71925
C	2.48827	2.34554	2.07119
H	1.93753	2.69909	1.19354
H	1.75241	2.13355	2.85440
H	3.11535	3.16920	2.41375
C	2.95974	-2.64268	1.29893
H	2.30064	-2.83381	2.15164
H	2.37215	-2.81129	0.39199
H	3.75626	-3.38735	1.32061
C	6.91207	0.39850	0.78005
H	7.01455	0.92963	-0.17066
H	7.41613	0.99943	1.54055
H	7.43572	-0.55417	0.69227

R = Mes*

P	-0.18588	2.27013	1.12057
C	0.26410	0.79941	0.48854
P	1.78830	-0.06250	0.26080
Cl	-1.16024	0.33551	-2.78457
O	1.38974	-0.97023	-0.90658
C	3.51729	0.45998	0.06468
C	4.06992	0.33166	-1.24520
C	4.39881	0.50012	1.17691
C	5.37064	-0.13739	-1.34088
C	5.69914	0.04482	0.98692
C	6.18145	-0.38024	-0.23963
H	5.77446	-0.30754	-2.33012
H	6.35882	0.02893	1.84285
C	-1.99897	2.20449	0.83817
C	-2.55833	2.94094	-0.23212
C	-2.80133	1.29115	1.56476
C	-3.80513	2.54388	-0.70703
C	-4.02261	0.92627	1.00912
C	-4.52170	1.48966	-0.15787
H	-4.22538	3.06592	-1.55158
H	-4.61827	0.18061	1.51895
C	-1.89353	4.19648	-0.82879
C	-2.48515	0.77468	2.98517
C	-5.83361	0.98452	-0.74937
C	4.13217	1.16744	2.54082
C	3.41870	0.80808	-2.55855
C	7.56124	-0.99859	-0.42238
C	-3.77244	0.82375	3.82687
H	-3.52941	0.58890	4.86605
H	-4.23112	1.81494	3.79840
H	-4.51424	0.09396	3.50215
C	-1.48053	1.66034	3.72067
H	-0.46353	1.58500	3.31856
H	-1.77836	2.71171	3.70968
H	-1.40737	1.33916	4.76318
C	-1.97732	-0.66854	2.98322
H	-2.67054	-1.33714	2.46827
H	-1.00227	-0.75236	2.50081
H	-1.86966	-1.02692	4.01116
C	-6.96108	1.14659	0.27716
H	-7.90367	0.78663	-0.14392
H	-6.76821	0.57675	1.18809
H	-7.09111	2.19557	0.55575
C	-6.22677	1.74159	-2.01515
H	-7.15589	1.32557	-2.41152
H	-6.39928	2.80372	-1.82124
H	-5.46602	1.65025	-2.79433
C	-5.67730	-0.49915	-1.10447
H	-4.89157	-0.64290	-1.85000
H	-5.42361	-1.10722	-0.23404
H	-6.61227	-0.88610	-1.51873
C	-2.80942	4.90101	-1.83253
H	-3.77000	5.17574	-1.39095
H	-2.32314	5.82127	-2.16404
H	-2.99422	4.29167	-2.72016
C	-1.62068	5.19609	0.30404
H	-2.54613	5.45755	0.82270
H	-0.91915	4.80440	1.04396
H	-1.18747	6.11319	-0.10490
C	-0.58909	3.89011	-1.56893
H	0.20531	3.55099	-0.89779
H	-0.73204	3.12010	-2.32821
H	-0.22128	4.79923	-2.05375
C	2.75131	1.79105	2.68283
H	2.52099	2.44862	1.84158
H	1.95809	1.05319	2.78995
H	2.72695	2.40725	3.58497
C	5.12764	2.34343	2.62575
H	4.96393	2.88667	3.56029
H	6.16595	2.01155	2.60808
H	4.98137	3.04025	1.79690
C	4.39041	0.22934	3.72323
H	5.40604	-0.17003	3.71478
H	4.26281	0.78045	4.65878
H	3.69705	-0.61116	3.72874
C	2.23040	1.73597	-2.32356
H	1.91411	2.16350	-3.27774
H	1.35404	1.23542	-1.92225
H	2.50432	2.56252	-1.66235
C	3.03326	-0.35574	-3.47571
H	2.26832	-0.98428	-3.02605
H	2.65010	0.03437	-4.42237
H	3.90339	-0.97835	-3.69907
C	4.45811	1.67304	-3.30098
H	4.82994	2.48222	-2.66773
H	5.31334	1.10041	-3.66025
H	3.97942	2.11931	-4.17602
C	8.28650	-1.19807	0.90568
H	8.49216	-0.24865	1.40678
H	7.71576	-1.83301	1.58790
H	9.24726	-1.68565	0.72550
C	8.42018	-0.09148	-1.31174
H	7.97977	0.04417	-2.30138
H	8.54511	0.89594	-0.86056
H	9.41181	-0.53169	-1.44652
C	7.40079	-2.36712	-1.09575
H	6.80003	-3.03953	-0.47887
H	6.91594	-2.28493	-2.07043
H	8.38103	-2.82707	-1.24668
Au	-2.20344	-2.73916	-0.17893
Cl	-3.53515	-4.51082	0.35366
Sn	-0.79756	-0.72731	-0.70657
Cl	1.88480	-1.34726	1.85155

Coordination through O atom

R= H

P	0.72527	-0.02047	1.06870
C	0.51102	-0.15236	-0.56938
P	-0.99308	0.00727	-1.40894
Cl	1.18388	-2.45767	-2.93096
Sn	1.76630	-0.18509	-2.49632
O	-0.54690	0.49003	-2.79687
Au	-1.84188	0.77350	-4.44094
Cl	-3.19320	1.07450	-6.17671
Cl	-2.34218	1.23247	-0.59621
H	-1.70584	-1.19668	-1.49971
H	2.14521	-0.20309	1.10154

R = Me

P	0.72897	-0.07462	1.05993
C	0.51549	-0.14741	-0.57906
P	-1.01160	-0.01425	-1.38472
Cl	1.37878	-2.39803	-2.98859
Sn	1.72565	-0.06897	-2.52882
O	-0.57159	0.42656	-2.80087
C	2.53077	-0.31855	1.30282
H	2.69305	-1.35698	1.61002
H	3.12135	-0.11912	0.40796
H	2.87293	0.31720	2.12256
C	-1.96569	-1.51845	-1.43867
H	-2.27800	-1.79064	-0.42961
H	-2.83794	-1.36644	-2.07739
H	-1.32218	-2.29428	-1.86063
Au	-1.86365	0.68521	-4.44506
Cl	-3.22153	0.95680	-6.18261
Cl	-2.25539	1.36207	-0.61157

R = *t*-Bu

P	0.79051	-0.60970	0.94337
C	0.44557	-0.10446	-0.59654
P	-1.17225	-0.08674	-1.24758
Cl	2.00710	-1.10748	-3.50919
Sn	1.39851	0.94306	-2.41355
O	-0.94211	0.61487	-2.60662
Au	-2.27481	1.46369	-3.99676
Cl	-3.64207	2.38151	-5.48972
C	2.63807	-0.49809	1.20389
C	3.09463	-1.94592	1.41582
C	3.42369	0.14251	0.07662
C	2.82048	0.29222	2.50306
H	2.52228	-2.44136	2.20509
H	2.99892	-2.53586	0.50113
H	4.14768	-1.95563	1.71543
H	3.11353	1.17879	-0.09203
H	4.48906	0.16956	0.32902
H	3.33292	-0.42902	-0.85234
H	3.87528	0.27998	2.79518
H	2.51570	1.33565	2.38849
H	2.24263	-0.14000	3.32442
C	-2.04540	-1.68626	-1.44439
C	-3.39951	-1.45197	-2.11505
C	-1.15917	-2.56290	-2.33378
C	-2.23755	-2.33194	-0.07413
H	-4.05989	-0.83545	-1.50371
H	-3.29975	-0.98338	-3.09630
H	-3.87919	-2.42468	-2.25483
H	-0.17792	-2.74308	-1.89198
H	-1.65812	-3.52721	-2.46218
H	-1.00984	-2.12392	-3.32098
H	-2.72895	-3.29772	-0.21929
H	-1.28878	-2.51460	0.43469
H	-2.87045	-1.72793	0.57822
Cl	-2.40128	1.07058	-0.13670

R = Ph

P	0.71433	0.22445	0.86664
C	0.53476	-0.25601	-0.71074
P	-0.91456	-0.20128	-1.65136
Cl	1.82109	-2.82567	-2.70486
Sn	1.96053	-0.43600	-2.47731
O	-0.34752	-0.12352	-3.08367
Au	-1.41959	0.07833	-4.88460
Cl	-2.51560	0.28842	-6.80757
C	2.46307	-0.09898	1.20297
C	3.19380	-1.13067	0.60344
C	3.09453	0.72818	2.13500
C	4.53581	-1.30035	0.90202
H	2.69598	-1.83660	-0.05531
C	4.44043	0.56418	2.42079
H	2.52846	1.51330	2.62682
C	5.16186	-0.44702	1.80187
H	5.09168	-2.10855	0.44077
H	4.92650	1.22216	3.13176
H	6.21226	-0.58168	2.03307
C	-2.01363	-1.59133	-1.45537
C	-2.93121	-1.63600	-0.40643
C	-1.89294	-2.66407	-2.33653
C	-3.73008	-2.75330	-0.24597
H	-3.02908	-0.79821	0.27421
C	-2.70241	-3.77619	-2.16848
H	-1.17401	-2.63098	-3.14545
C	-3.61672	-3.82108	-1.12757
H	-4.44883	-2.78890	0.56398
H	-2.61743	-4.60712	-2.85824
H	-4.24955	-4.69219	-1.00241
Cl	-2.04064	1.42719	-1.27582

R = Mes

P	0.59263	-0.06959	1.29882
C	0.33723	-0.34726	-0.31271
P	-1.04718	-0.25722	-1.35769
Cl	2.00528	-2.52554	-2.42781
Sn	1.93496	-0.17463	-1.90746
O	-0.36121	0.11880	-2.68677
Au	-0.96916	1.37434	-4.26944
Cl	-1.54267	2.70501	-5.95668
C	2.41764	-0.19300	1.39436
C	3.04895	-1.44623	1.46237
C	3.17060	0.98919	1.48077
C	4.43332	-1.48576	1.57531
C	4.55211	0.89730	1.59012
C	5.20332	-0.32972	1.63080
H	4.92379	-2.45298	1.63260
H	5.13482	1.81167	1.65299
C	-2.06259	-1.73280	-1.44813
C	-2.75496	-2.13459	-0.28343
C	-2.14484	-2.51125	-2.62726
C	-3.48799	-3.31130	-0.31203
C	-2.90589	-3.67322	-2.58581
C	-3.58012	-4.09676	-1.45082
H	-4.00724	-3.61821	0.59038
H	-2.96519	-4.27355	-3.48772
Cl	-2.28292	1.28182	-0.89829
C	2.50961	2.33539	1.43833

H	2.16076	2.58188	0.43038
H	1.63500	2.37851	2.09562
H	3.20079	3.11981	1.74846
C	6.69622	-0.40582	1.72056
H	7.13992	-0.46820	0.72199
H	7.11200	0.47714	2.20864
H	7.01733	-1.28957	2.27463
C	2.26611	-2.72512	1.43067
H	1.39802	-2.68231	2.09573
H	1.89333	-2.94018	0.42528
H	2.88767	-3.56467	1.74398
C	-2.78899	-1.34517	0.99061
H	-3.50560	-0.52329	0.91942
H	-3.09878	-1.98630	1.81633
H	-1.82733	-0.90718	1.26162
C	-1.48144	-2.19071	-3.93296
H	-1.80782	-1.22898	-4.33486
H	-0.39588	-2.15528	-3.84019
H	-1.73263	-2.95938	-4.66335
C	-4.40332	-5.34552	-1.46536
H	-4.49237	-5.77666	-0.46712
H	-5.41534	-5.13121	-1.82294
H	-3.97505	-6.09624	-2.13151

R = Mes*

P	1.88951	2.17246	-0.11508
C	0.96328	0.80172	-0.22639
P	-0.75000	0.52089	-0.33576
C	3.56695	1.40509	-0.15668
C	4.31865	1.31745	1.04474
C	4.01811	0.73315	-1.31281
C	5.32798	0.36921	1.10731
C	5.01788	-0.23360	-1.15905
C	5.64946	-0.47437	0.04696
H	5.87967	0.25946	2.02933
H	5.32550	-0.79267	-2.03227
C	3.64804	1.08455	-2.77142
C	4.09953	2.25872	2.24348
C	6.70167	-1.56252	0.23206
C	2.75934	2.01368	2.94222
H	1.90532	2.29153	2.31880
H	2.63918	0.96400	3.21475
H	2.70088	2.62417	3.84812
C	4.17906	3.71371	1.76143
H	5.14596	3.91589	1.29411
H	3.39721	3.95735	1.03905
H	4.06211	4.39285	2.61044
C	5.18662	2.08529	3.30706
H	5.14489	1.10315	3.78358
H	6.18926	2.23556	2.89969
H	5.03265	2.83203	4.08920
C	3.00556	-0.09631	-3.50335
H	2.01462	-0.32207	-3.10249
H	2.88187	0.14452	-4.56289
H	3.61382	-1.00045	-3.43704
C	2.74054	2.30177	-2.93348
H	1.70758	2.10114	-2.64689
H	3.10812	3.16573	-2.37191
H	2.71924	2.59032	-3.98819
C	4.96419	1.45016	-3.48492
H	5.46023	2.28442	-2.98285
H	5.66568	0.61664	-3.52228
H	4.75021	1.74997	-4.51416
C	6.89520	-2.40063	-1.02877
H	7.26734	-1.80315	-1.86519
H	7.63110	-3.18441	-0.83504
H	5.96748	-2.88903	-1.33784
C	6.26305	-2.49566	1.36723
H	6.15115	-1.96057	2.31199
H	5.30425	-2.96800	1.14205
H	7.00794	-3.28270	1.51348
C	8.04542	-0.91890	0.59428
H	7.98191	-0.34050	1.51813
H	8.80540	-1.69201	0.73715
H	8.38608	-0.24916	-0.19936
C	-2.22260	1.35676	0.31007
C	-2.82677	0.75087	1.45228
C	-3.01931	2.18119	-0.52670
C	-4.20843	0.65693	1.46366
C	-4.39931	2.04048	-0.43855
C	-5.01802	1.19737	0.47196
H	-4.67427	0.13472	2.28956
H	-5.01219	2.62292	-1.11244
C	-2.09946	0.34889	2.74996
C	-2.20158	-1.14626	3.05707
C	-2.79271	1.12568	3.88849
H	-1.63779	-1.74294	2.34090
H	-1.79465	-1.34257	4.05269
H	-3.23760	-1.49083	3.04310
H	-2.77830	2.20187	3.69892
H	-3.82901	0.82174	4.03704
H	-2.25671	0.93701	4.82213
C	-2.51744	3.36226	-1.38030
C	-3.14406	4.61898	-0.73988
C	-2.97649	3.28833	-2.83873
H	-2.84862	4.71290	0.30787
H	-2.79517	5.50725	-1.27357
H	-4.23333	4.60618	-0.78337
H	-2.54454	2.43227	-3.35586
H	-4.06250	3.21779	-2.92031
H	-2.66591	4.19522	-3.36459
C	-6.51628	0.93013	0.47430
C	-7.13898	1.51099	1.74977
C	-6.74458	-0.58629	0.43841
H	-6.98942	2.59240	1.80502
H	-8.21452	1.31491	1.76131
H	-6.71062	1.06464	2.64943
H	-6.29451	-1.03963	-0.44718
H	-6.31475	-1.08372	1.30964
H	-7.81704	-0.79882	0.42809
Cl	2.09631	-1.64996	2.04255
O	-0.81877	-0.96411	0.10189
Cl	-1.08189	0.40369	-2.35430
Au	-2.43141	-2.24458	-0.38147
Cl	-4.11346	-3.61938	-0.88931
C	-1.01109	3.58617	-1.32715
H	-0.64581	3.61626	-0.29791
H	-0.44614	2.83950	-1.88168
H	-0.77692	4.55285	-1.77987
C	-0.64048	0.78628	2.78700
H	0.00714	0.19730	2.14097
H	-0.53632	1.84520	2.53573
H	-0.25117	0.64594	3.79822
C	-7.21381	1.55247	-0.73227
H	-7.15214	2.64412	-0.72639
H	-6.79567	1.18544	-1.67275
H	-8.27320	1.28693	-0.71503
Sn	1.56005	-1.41106	-0.29431

Coordination through P(sp^2) atom

R= H

P	0.61606	0.00599	0.92159
C	0.37971	-0.26051	-0.67361
P	-1.10853	-0.26698	-1.56923
Cl	1.25717	-2.76501	-2.78383
Sn	1.55367	-0.38915	-2.69486
O	-0.67389	-0.00820	-2.99563
Au	-0.71856	0.42027	2.61556
Cl	-2.07960	0.83991	4.36269
Cl	-2.48762	1.04382	-0.95144
H	-1.79144	-1.48340	-1.41736
H	2.01221	-0.07699	1.11822

R = Me

P	0.71735	0.05040	0.87419
C	0.41972	-0.18975	-0.71286
P	-1.12671	-0.28768	-1.51000
Cl	1.37422	-2.81369	-2.64303
Sn	1.46961	-0.40826	-2.77777
O	-0.75629	-0.20560	-2.98281
C	2.49248	0.01494	1.21667
H	2.69803	-0.81769	1.89436
H	3.07259	-0.10093	0.30213
H	2.77316	0.93640	1.73189
C	-2.04595	-1.76246	-1.10989
H	-2.31650	-1.76124	-0.05308
H	-2.94509	-1.79736	-1.72738
H	-1.40259	-2.61516	-1.33926
Au	-0.68770	0.33804	2.54877
Cl	-2.12832	0.59681	4.27079
Cl	-2.35947	1.22834	-1.01941

R = *t*-Bu

P	0.74285	0.11358	0.77398
C	0.51468	-0.37019	-0.77289
P	-0.99398	-0.56500	-1.63897
Cl	2.11293	-2.91488	-2.59003
Sn	1.68497	-0.55052	-2.78451
O	-0.53204	-0.70280	-3.08482
Au	-0.74787	0.71450	2.29772
Cl	-2.26087	1.33794	3.86408
C	2.51987	0.14121	1.29128
C	2.80218	1.53687	1.85284
C	2.64807	-0.90220	2.40698
C	3.46851	-0.18278	0.14971
H	2.72602	2.30785	1.08239
H	2.11554	1.79439	2.66272
H	3.81937	1.55878	2.25487
H	2.47508	-1.91465	2.03505
H	3.66248	-0.85948	2.81491
H	1.95017	-0.70922	3.22538
H	4.49589	-0.18053	0.52613
H	3.27655	-1.17129	-0.27479
H	3.41674	0.56881	-0.64313
C	-2.08690	-1.95366	-1.15575
C	-1.24607	-3.23020	-1.23696
C	-2.63822	-1.76783	0.25263
C	-3.22178	-2.00825	-2.18161
H	-0.84101	-3.39270	-2.23610
H	-0.41194	-3.21853	-0.53338
H	-1.89019	-4.07713	-0.98495
H	-3.22971	-0.85672	0.35216
H	-3.28762	-2.61692	0.48312
H	-1.84672	-1.74842	1.00451
H	-3.83455	-2.88759	-1.96531
H	-3.86449	-1.12826	-2.12561
H	-2.84045	-2.10072	-3.19981
Cl	-2.13316	1.10135	-1.45847

R = Ph

P	0.73638	0.19153	0.58064
C	0.55600	-0.47601	-0.90235
P	-0.90957	-0.74675	-1.80121
Cl	1.99249	-3.34448	-2.23877
Sn	1.81379	-0.99534	-2.77088
O	-0.39568	-1.07748	-3.19367
Au	-0.78631	1.09734	1.90145
Cl	-2.38097	1.98721	3.23868
C	2.44210	0.13175	1.10508
C	2.87708	1.06935	2.04361
C	3.33050	-0.83138	0.61850
C	4.19769	1.06947	2.45965
H	2.17844	1.79905	2.43997
C	4.64572	-0.83070	1.04787
H	2.98035	-1.60581	-0.05608
C	5.08122	0.12318	1.95975
H	4.53603	1.80513	3.17958
H	5.33083	-1.58512	0.67939
H	6.11264	0.11875	2.29320
C	-1.95991	-2.03095	-1.14581
C	-2.84440	-1.78977	-0.09529
C	-1.82300	-3.31200	-1.67728
C	-3.59272	-2.83378	0.41863
H	-2.95538	-0.79292	0.31631
C	-2.58126	-4.34885	-1.15696
H	-1.12662	-3.49087	-2.48729
C	-3.46183	-4.11075	-0.11249
H	-4.28196	-2.64858	1.23377
H	-2.48046	-5.34554	-1.56979
H	-4.05241	-4.92495	0.29187
Cl	-2.06494	0.90734	-1.85400

R = Mes

P	0.66149	0.34741	1.16106
C	0.31775	-0.43175	-0.23455
P	-1.06364	-0.60503	-1.28400
Cl	1.72198	-3.08022	-2.00214
Sn	1.75714	-0.66311	-2.01735
O	-0.40207	-0.45719	-2.64717
Au	-0.61538	1.38413	2.63821
Cl	-1.89376	2.45039	4.17309
C	2.44656	0.29454	1.40782
C	3.18496	1.48280	1.27626
C	3.07475	-0.91314	1.75457
C	4.55755	1.43250	1.47126
C	4.45083	-0.90441	1.94521
C	5.20918	0.25141	1.80746
H	5.13467	2.34512	1.35763
H	4.94379	-1.83323	2.21532
C	-2.01293	-2.11601	-1.09680
C	-2.51057	-2.47058	0.17443
C	-2.27142	-2.94062	-2.21591
C	-3.22325	-3.65256	0.31054
C	-2.99210	-4.11029	-2.00898
C	-3.46626	-4.49477	-0.76370
H	-3.60691	-3.91454	1.29155
H	-3.19584	-4.74194	-2.86768
Cl	-2.39378	0.91583	-1.07225
C	2.52773	2.78200	0.91836

H	2.00779	2.72306	-0.04258
H	1.78500	3.07464	1.66727
H	3.26459	3.58233	0.84840
C	2.31192	-2.19191	1.93519
H	1.42115	-2.04482	2.55319
H	1.97918	-2.60368	0.97833
H	2.93551	-2.94093	2.42395
C	6.68668	0.23357	2.04832
H	6.90886	0.48305	3.09059
H	7.11118	-0.75165	1.84955
H	7.20111	0.96430	1.42188
C	-2.30832	-1.64289	1.40589
H	-3.02458	-1.93162	2.17527
H	-1.30725	-1.79194	1.81886
H	-2.43444	-0.57395	1.22954
C	-1.83994	-2.65445	-3.62485
H	-2.11953	-1.65424	-3.95385
H	-0.75662	-2.73344	-3.72832
H	-2.30062	-3.38046	-4.29518
C	-4.20752	-5.78175	-0.58480
H	-4.76060	-6.05190	-1.48582
H	-3.50902	-6.59739	-0.37290
H	-4.90842	-5.72602	0.24962

R = Mes*

P	-1.05258	0.96593	0.06589
C	-0.26220	-0.42349	0.41998
P	1.36296	-1.05293	0.55902
Cl	-1.63010	-2.92839	-1.83415
O	1.16105	-2.47781	0.05881
C	3.00951	-0.48049	0.05086
C	3.55242	-1.14377	-1.08942
C	3.89320	0.16130	0.95820
C	4.90260	-1.45535	-1.06119
C	5.23803	-0.19183	0.90169
C	5.75440	-1.07240	-0.03360
H	5.30899	-2.01849	-1.89117
H	5.90682	0.25648	1.62246
C	-2.82099	0.52957	0.11808
C	-3.54566	0.30016	-1.08116
C	-3.37458	0.15008	1.36638
C	-4.67312	-0.51103	-1.00137
C	-4.48918	-0.68219	1.34900
C	-5.12419	-1.07580	0.18211
H	-5.20657	-0.73463	-1.91005
H	-4.88430	-1.02721	2.29587
C	-3.22239	0.96323	-2.43391
C	-2.94108	0.65320	2.76099
C	-6.29258	-2.05432	0.23840
C	3.57281	1.35199	1.88454
C	2.82231	-1.42524	-2.41740
C	7.20254	-1.54518	-0.01943
C	-4.19329	1.20844	3.46612
H	-3.90740	1.61811	4.43810
H	-4.64817	2.01058	2.88015
H	-4.95409	0.44881	3.64420
C	-1.94156	1.80782	2.74994
H	-0.93065	1.50047	2.47849
H	-2.25576	2.62017	2.08964
H	-1.86789	2.22145	3.75935
C	-2.36469	-0.48872	3.60252
H	-3.06213	-1.32556	3.67959
H	-1.43069	-0.86076	3.17648
H	-2.15159	-0.13593	4.61559
C	-7.41788	-1.46700	1.09819
H	-8.25810	-2.16522	1.14364
H	-7.09109	-1.27453	2.12213
H	-7.78062	-0.52546	0.67863
C	-6.85680	-2.36418	-1.14559
H	-7.67100	-3.08663	-1.05189
H	-7.26392	-1.47284	-1.62970
H	-6.10088	-2.80043	-1.80295
C	-5.80596	-3.36772	0.86313
H	-5.00955	-3.81357	0.26217
H	-5.42167	-3.21768	1.87435
H	-6.62992	-4.08413	0.92179
C	-4.21080	0.54786	-3.52795
H	-5.24122	0.81277	-3.28057
H	-3.95132	1.07947	-4.44584
H	-4.16163	-0.52240	-3.74261
C	-3.36450	2.48188	-2.26298
H	-4.37782	2.74175	-1.94654
H	-2.66923	2.88399	-1.52285
H	-3.16157	2.98648	-3.21120
C	-1.83277	0.61423	-2.97168
H	-1.01990	1.02533	-2.36964
H	-1.69394	-0.46514	-3.03955
H	-1.71415	1.04842	-3.96834
C	2.13080	1.83587	1.83172
H	1.80538	1.97794	0.79845
H	1.43106	1.17739	2.34111
H	2.06175	2.81194	2.31710
C	4.41469	2.52837	1.34471
H	4.21260	3.41915	1.94489
H	5.48559	2.32815	1.38786
H	4.15095	2.75517	0.30928
C	3.97504	1.09676	3.33991
H	5.03616	0.85992	3.43678
H	3.78560	1.99708	3.93034
H	3.40461	0.27807	3.77821
C	1.46069	-0.74541	-2.52874
H	1.12740	-0.78831	-3.56833
H	0.68418	-1.23831	-1.94922
H	1.51656	0.31007	-2.24736
C	2.68275	-2.92583	-2.68654
H	2.05976	-3.40308	-1.93201
H	2.22129	-3.08227	-3.66525
H	3.65808	-3.41871	-2.69483
C	3.67912	-0.80171	-3.53830
H	3.82834	0.26806	-3.37276
H	4.65867	-1.26935	-3.63855
H	3.15823	-0.92780	-4.49064
C	7.96819	-1.02941	1.19607
H	8.04938	0.06052	1.19625
H	7.49930	-1.34117	2.13274
H	8.98377	-1.43183	1.18370
C	7.91797	-1.05644	-1.28462
H	7.44900	-1.44152	-2.19212
H	7.91364	0.03477	-1.34092
H	8.95832	-1.39288	-1.27921
C	7.21965	-3.07829	0.01420
H	6.72502	-3.45568	0.91238
H	6.71286	-3.50801	-0.85206
H	8.25088	-3.44165	0.01405
Sn	-1.06475	-2.62689	0.49718
Cl	1.55488	-1.31339	2.59373
Au	-0.25924	2.98585	-0.43257
Cl	0.56173	5.04523	-0.92293

Coordination through π(C=P) bond

R= H

P	0.69397	-0.02751	1.07102
C	0.47796	0.04697	-0.63785
P	-1.00172	-0.24869	-1.52209
Cl	1.47464	-2.66822	-2.40019
Sn	1.63533	-0.28173	-2.67063
O	-0.61580	-0.07585	-2.97531
Au	0.74848	2.08686	-0.02707
Cl	1.02455	4.31507	0.01631
Cl	-2.58743	0.88055	-1.04029
H	2.11621	-0.16629	1.08513
H	-1.48220	-1.53593	-1.23468

R = Me

P	0.63132	0.01059	1.00086
C	0.43175	0.14156	-0.70334
P	-1.03011	-0.22644	-1.60487
Cl	1.74440	-2.60194	-2.23182
Sn	1.65097	-0.22086	-2.67821
O	-0.56620	-0.21919	-3.05275
C	2.42910	-0.27293	1.22622
H	2.56855	-1.35621	1.30950
H	3.04038	0.10072	0.40534
H	2.75041	0.18070	2.16590
C	-1.75075	-1.78916	-1.12842
H	-2.10733	-1.74287	-0.09872
H	-2.57878	-2.01325	-1.80275
H	-0.97271	-2.54935	-1.23163
Au	0.62328	2.16909	-0.02732
Cl	0.84419	4.39989	0.17251
Cl	-2.52741	1.10562	-1.36145

R = *t*-Bu

P	1.13023	0.07913	1.17828
C	0.66672	0.11867	-0.48372
P	-0.92296	-0.36361	-1.10381
Cl	2.19884	-2.60501	-2.07320
Sn	1.57647	-0.32537	-2.61341
O	-0.61779	-0.77310	-2.53743
Au	0.81340	2.18518	0.10373
Cl	0.93769	4.43173	0.25829
C	2.99177	-0.04797	1.30594
C	3.79307	0.30031	0.06506
C	3.37992	0.86717	2.46954
C	3.22857	-1.51304	1.69642
H	3.62915	-0.42732	-0.73376
H	3.57080	1.30572	-0.30079
H	4.86198	0.26418	0.30055
H	2.78426	0.66273	3.36378
H	4.43103	0.70251	2.72529
H	3.25457	1.92177	2.21277
H	4.28963	-1.65408	1.92575
H	2.66020	-1.79483	2.58758
H	2.96666	-2.19333	0.88240
C	-1.85930	-1.67130	-0.22343
C	-0.92492	-2.87438	-0.06865
C	-2.33568	-1.18132	1.14096
C	-3.04771	-2.04973	-1.11068
H	-0.51756	-3.20709	-1.02426
H	-0.08989	-2.66204	0.60107
H	-1.49971	-3.69734	0.36505
H	-3.03827	-0.35159	1.05383
H	-2.84845	-2.00622	1.64363
H	-1.50585	-0.86941	1.77916
H	-3.58970	-2.86684	-0.62654
H	-3.74105	-1.21710	-1.24111
H	-2.72156	-2.39026	-2.09441
Cl	-2.18213	1.22987	-1.15035

R = Ph

P	0.57416	0.20074	0.95516
C	0.34351	0.18360	-0.74945
P	-1.15730	-0.13157	-1.59978
Cl	1.76069	-2.21800	-2.99602
Sn	1.45054	0.17936	-2.83226
O	-0.78976	0.05514	-3.06256
Au	0.60696	2.27501	-0.24636
Cl	0.89143	4.50956	-0.25203
C	2.34689	-0.08358	1.20202
C	2.92111	0.46171	2.35310
C	3.12229	-0.88193	0.35974
C	4.26167	0.25418	2.62942
H	2.31734	1.06416	3.02475
C	4.45876	-1.10189	0.65110
H	2.67421	-1.36134	-0.50418
C	5.03129	-0.52670	1.77761
H	4.70620	0.69734	3.51289
H	5.05350	-1.72937	-0.00273
H	6.07867	-0.69711	1.99905
C	-1.81481	-1.75939	-1.26937
C	-1.76780	-2.71449	-2.28018
C	-2.34436	-2.07690	-0.01925
C	-2.25716	-3.98906	-2.03672
H	-1.34489	-2.45921	-3.24334
C	-2.82557	-3.35192	0.21510
H	-2.38653	-1.32949	0.76570
C	-2.78244	-4.30662	-0.79422
H	-2.22191	-4.73537	-2.82136
H	-3.23850	-3.60256	1.18502
H	-3.16194	-5.30473	-0.60735
Cl	-2.65942	1.12736	-1.09779

R = Mes

P	0.57337	0.26309	1.36475
C	0.20753	0.16312	-0.31739
P	-1.23925	-0.11515	-1.28119
Cl	1.79414	-1.81933	-2.94948
Sn	1.42561	0.49403	-2.31007
O	-0.79184	0.38995	-2.64571
Au	0.26939	2.29793	0.13672
Cl	0.28085	4.55574	0.07556
C	2.38418	0.03678	1.43857
C	3.21048	1.02540	1.99836
C	2.92120	-1.21997	1.09442
C	4.57549	0.78116	2.09404
C	4.29001	-1.41342	1.21566
C	5.13985	-0.41902	1.68648
H	5.21341	1.55327	2.51403
H	4.70201	-2.38305	0.95219
C	-1.83820	-1.81018	-1.28870
C	-2.01022	-2.50461	-2.51036
C	-2.16074	-2.43979	-0.06918
C	-2.42599	-3.82832	-2.45295
C	-2.57365	-3.76512	-0.08433
C	-2.69274	-4.48652	-1.26086
H	-2.55873	-4.36112	-3.38911
H	-2.82312	-4.23941	0.85993
Cl	-2.85426	1.01088	-0.74438
C	2.67679	2.33263	2.50227

H	2.56988	3.06966	1.70071
H	1.69278	2.22387	2.96733
H	3.35175	2.75569	3.24797
C	2.06189	-2.37789	0.67645
H	1.19879	-2.49590	1.33909
H	1.68335	-2.27346	-0.34198
H	2.63540	-3.30451	0.71947
C	6.61784	-0.64444	1.76276
H	6.85074	-1.68184	2.01046
H	7.09032	-0.42656	0.79976
H	7.08229	0.00156	2.50945
C	-2.10306	-1.76787	1.26983
H	-1.13165	-1.91932	1.74890
H	-2.28258	-0.69476	1.23007
H	-2.85569	-2.20097	1.93058
C	-1.81072	-1.92013	-3.87835
H	-2.33242	-0.97228	-4.00531
H	-0.75506	-1.73576	-4.08085
H	-2.18312	-2.62266	-4.62438
C	-3.10097	-5.92563	-1.25148
H	-3.72388	-6.16822	-2.11435
H	-2.21904	-6.57229	-1.29531
H	-3.65088	-6.17960	-0.34412

R = Mes*

P	0.67193	0.05456	1.16098
C	0.38782	0.09700	-0.55450
P	-1.04930	-0.16312	-1.61903
O	-0.37797	-0.08056	-2.98369
Cl	2.81815	-1.30318	-3.25930
Au	0.34005	2.18661	0.06258
Cl	0.13903	4.44846	0.12150
C	2.52115	-0.03001	1.25402
C	3.20877	-1.16764	0.74675
C	3.28571	1.08827	1.66626
C	4.51569	-1.00057	0.31533
C	4.59406	1.18881	1.18713
C	5.20459	0.20206	0.43410
H	5.02652	-1.85249	-0.11378
H	5.15619	2.07719	1.43832
C	2.91146	2.11602	2.75549
C	2.72999	-2.62457	0.90403
C	6.59366	0.35585	-0.17427
C	3.88645	1.84313	3.91994
H	3.77410	0.82225	4.29418
H	4.92714	1.97953	3.62384
H	3.67504	2.53367	4.74096
C	3.10375	3.56180	2.29043
H	2.43884	3.81596	1.46454
H	2.87940	4.24282	3.11598
H	4.13012	3.75910	1.97661
C	1.51444	1.97496	3.35225
H	1.44248	2.62257	4.23022
H	0.72152	2.29192	2.67247
H	1.30969	0.95365	3.68333
C	3.05906	-3.49498	-0.31116
H	2.63636	-3.08439	-1.22671
H	4.13403	-3.60512	-0.46154
H	2.65533	-4.49950	-0.15769
C	3.50877	-3.17033	2.11687
H	4.58622	-3.12406	1.95001
H	3.28289	-2.59737	3.01987
H	3.23356	-4.21425	2.29482
C	1.25178	-2.80232	1.22936
H	0.95426	-2.24451	2.12381
H	0.60225	-2.52404	0.40073
H	1.06141	-3.85723	1.44696
C	7.20054	1.72734	0.10852
H	6.57553	2.53526	-0.27982
H	7.35458	1.89391	1.17795
H	8.17587	1.80111	-0.37839
C	6.48383	0.17857	-1.69349
H	7.47315	0.25306	-2.15379
H	6.05609	-0.78964	-1.96017
H	5.84690	0.95073	-2.13196
C	7.53551	-0.71202	0.39436
H	7.62053	-0.62193	1.48027
H	7.19261	-1.72280	0.16530
H	8.53419	-0.59693	-0.03584
C	-2.35079	-1.45978	-1.66513
C	-2.32345	-2.35220	-2.78074
C	-3.58777	-1.26171	-0.99346
C	-3.53420	-2.66407	-3.37991
C	-1.10124	-3.13171	-3.30186
C	-4.75498	-1.64704	-1.64453
C	-3.78950	-0.80781	0.46406
C	-4.76520	-2.25457	-2.88750
H	-3.51011	-3.27847	-4.26998
C	-0.61161	-2.62921	-4.66295
C	-1.51731	-4.60705	-3.47981
C	0.02168	-3.17104	-2.27397
H	-5.69519	-1.45402	-1.14810
C	-2.50507	-0.46302	1.19511
C	-4.38319	-2.02591	1.20044
C	-4.76592	0.36497	0.59533
C	-6.04338	-2.55668	-3.65841
H	0.19840	-3.27063	-5.01979
H	-1.42043	-2.66914	-5.39757
H	-0.24395	-1.60874	-4.61131
H	-2.19724	-4.76192	-4.31812
H	-0.62052	-5.19756	-3.68336
H	-1.98807	-5.00392	-2.57721
H	0.45161	-2.19781	-2.05010
H	-0.33048	-3.62827	-1.34593
H	0.84503	-3.77611	-2.65764
H	-1.76653	-1.26188	1.10197
H	-2.06709	0.47597	0.85821
H	-2.71154	-0.33970	2.26078
H	-4.52048	-1.77743	2.25633
H	-5.35106	-2.32366	0.79577
H	-3.71199	-2.88596	1.13722
H	-5.74569	0.13768	0.17220
H	-4.91243	0.59617	1.65373
H	-4.38375	1.26018	0.10512
C	-7.28358	-2.00410	-2.96121
C	-6.20810	-4.07377	-3.80949
C	-5.94370	-1.91514	-5.04771
H	-7.44874	-2.47279	-1.98769
H	-7.21959	-0.92263	-2.81767
H	-8.16552	-2.20570	-3.57358
H	-5.37285	-4.52168	-4.35126
H	-6.27514	-4.55988	-2.83308
H	-7.12346	-4.29711	-4.36425
H	-5.83537	-0.83068	-4.97126
H	-5.08964	-2.29760	-5.61001
H	-6.84772	-2.12818	-5.62456
Sn	1.67250	0.67991	-2.46257
Cl	-2.24781	1.50642	-1.58847

[R-P=C(Sn(II)Cl)-P(O)MeR](AuCl)

Coordination through Sn(II) atom

R= H

P	0.65446	-0.14382	1.17454
C	0.47593	-0.20530	-0.47064
P	-1.01756	-0.16496	-1.40760
Cl	1.76443	-2.41087	-2.78958
Sn	1.65541	-0.10640	-2.35730
O	-0.47610	0.11022	-2.81659
C	-2.23516	1.03212	-0.88074
H	-3.08401	1.01254	-1.56703
H	-2.57463	0.78647	0.12833
H	-1.78500	2.02576	-0.88501
Au	3.25669	1.63528	-3.17177
Cl	4.68257	3.26666	-3.84512
H	2.08186	-0.13059	1.23462
H	-1.67422	-1.41047	-1.34120

R = Me

P	0.65746	-0.15984	1.16105
C	0.47511	-0.21955	-0.48006
P	-1.03181	-0.21589	-1.39842
Cl	1.80175	-2.39494	-2.83167
Sn	1.61996	-0.08715	-2.37299
O	-0.49323	0.07479	-2.81544
C	2.46731	-0.14092	1.44266
H	2.76809	-1.13107	1.79975
H	3.03978	0.10830	0.54873
H	2.69752	0.57137	2.23851
C	-1.86431	-1.80088	-1.33394
H	-2.21805	-2.00261	-0.32105
H	-2.70851	-1.79799	-2.02672
H	-1.15257	-2.57164	-1.63445
C	-2.20348	1.03763	-0.88161
H	-3.06804	1.02806	-1.54848
H	-2.52898	0.84500	0.14277
H	-1.72103	2.01435	-0.93077
Au	3.22109	1.65188	-3.19917
Cl	4.66369	3.27848	-3.85908

R = *t*-Bu

P	0.69434	-0.58428	0.94224
C	0.64333	-0.20450	-0.66631
P	-0.81345	-0.15698	-1.67744
Cl	2.72437	-1.45161	-3.24688
Sn	1.84542	0.55528	-2.37253
O	-0.20350	0.31816	-3.01751
Au	2.90425	2.80365	-2.70643
Cl	3.81778	4.87759	-2.90795
C	2.45643	-0.45577	1.55123
C	2.79761	-1.85446	2.07628
C	3.47711	-0.01271	0.52190
C	2.40828	0.53274	2.72072
H	2.06012	-2.20843	2.80214
H	2.85813	-2.58521	1.26603
H	3.77028	-1.82685	2.57772
H	3.27251	0.99556	0.15055
H	4.47289	0.01636	0.97630
H	3.53335	-0.70796	-0.32099
H	3.38246	0.55497	3.21920
H	2.18207	1.54712	2.38309
H	1.66040	0.24595	3.46529
C	-1.68487	-1.75481	-1.89573
C	-2.72038	-1.56673	-3.00797
C	-0.66012	-2.80717	-2.31861
C	-2.36250	-2.16636	-0.59049
H	-3.50609	-0.86116	-2.72869
H	-2.25575	-1.22289	-3.93378
H	-3.20032	-2.52923	-3.20607
H	0.08881	-2.98007	-1.54405
H	-1.18266	-3.75011	-2.50235
H	-0.13909	-2.52267	-3.23387
H	-2.81874	-3.15097	-0.72567
H	-1.65083	-2.24345	0.23561
H	-3.15627	-1.47466	-0.30024
C	-1.98052	1.06381	-1.06078
H	-2.82229	1.16703	-1.74700
H	-2.33928	0.78619	-0.06881
H	-1.45795	2.01941	-0.99581

R = Ph

P	0.78674	-0.03528	1.22748
C	0.45782	-0.31907	-0.37091
P	-1.11828	-0.37093	-1.15832
Cl	1.75182	-2.58094	-2.74963
Sn	1.45665	-0.27330	-2.33896
O	-0.69927	-0.23298	-2.63590
Au	2.86257	1.49778	-3.41843
Cl	4.12765	3.14607	-4.34320
C	2.58759	-0.06714	1.35154
C	3.16308	0.73522	2.33977
C	3.41423	-0.85316	0.54390
C	4.54013	0.79534	2.47937
H	2.52675	1.32789	2.98977
C	4.78873	-0.80171	0.69578
H	2.97992	-1.53967	-0.17639
C	5.35252	0.03044	1.65505
H	4.98007	1.43746	3.23343
H	5.42316	-1.41569	0.06731
H	6.42978	0.07301	1.76657
C	-1.99334	-1.91204	-0.88818
C	-2.12550	-2.80645	-1.94613
C	-2.52280	-2.22420	0.36346
C	-2.79585	-4.00500	-1.75187
H	-1.69923	-2.56088	-2.91127
C	-3.18912	-3.42251	0.55006
H	-2.40597	-1.54171	1.19932
C	-3.32701	-4.31151	-0.50868
H	-2.89795	-4.70222	-2.57503
H	-3.59899	-3.66653	1.52307
H	-3.84802	-5.25029	-0.35961
C	-2.20820	0.96394	-0.66183
H	-3.12015	0.92489	-1.26022
H	-2.46277	0.87119	0.39565
H	-1.69744	1.91333	-0.82831

R = Mes

P	0.91646	-0.38600	1.79074
C	0.37052	-0.45591	0.22755
P	-1.20876	-0.36953	-0.57327
Cl	1.68233	-1.99301	-2.66779
Sn	1.40202	0.11151	-1.62376
O	-0.74800	0.24731	-1.91270
Au	2.85711	2.09337	-2.12014
Cl	4.18757	3.91710	-2.43365
C	2.71250	-0.17991	1.53551
C	3.27426	1.09837	1.68114
C	3.52140	-1.28428	1.22944
C	4.63573	1.25833	1.46946
C	4.88156	-1.07418	1.03256
C	5.45529	0.18697	1.13268

H	5.06561	2.25166	1.55559
H	5.51170	-1.92597	0.79451
C	-2.04809	-1.95524	-0.77946
C	-2.47837	-2.39489	-2.05230
C	-2.30689	-2.74459	0.35792
C	-3.13573	-3.61584	-2.13666
C	-2.97314	-3.95390	0.20752
C	-3.38819	-4.41545	-1.03088
H	-3.46988	-3.94926	-3.11413
H	-3.17213	-4.55001	1.09295
C	-2.36415	0.78226	0.18689
H	-3.19446	0.92912	-0.50725
H	-2.75395	0.41319	1.13471
H	-1.85238	1.73313	0.34283
C	-1.87453	-2.37985	1.74760
H	-0.86074	-2.73979	1.94391
H	-1.86915	-1.30923	1.94992
H	-2.53488	-2.84606	2.48014
C	-2.28407	-1.64059	-3.33662
H	-2.62856	-0.60852	-3.27666
H	-1.23056	-1.60244	-3.61783
H	-2.83296	-2.14065	-4.13495
C	-4.07355	-5.73746	-1.17812
H	-4.52454	-6.06097	-0.23895
H	-4.85309	-5.69833	-1.94129
H	-3.35833	-6.50723	-1.48397
C	2.95885	-2.67128	1.11511
H	2.26613	-2.89444	1.93280
H	2.40932	-2.80917	0.17953
H	3.75793	-3.41308	1.14328
C	2.42511	2.29024	2.00831
H	1.66182	2.05618	2.75798
H	3.03389	3.10956	2.39191
H	1.90761	2.65953	1.11684
C	6.91401	0.39893	0.87135
H	7.05899	0.93634	-0.07031
H	7.37473	0.99941	1.65913
H	7.44997	-0.54863	0.80371

R = Mes*

P	-0.19960	2.21884	1.16953
C	0.28186	0.76791	0.52156
P	1.81173	-0.13567	0.35568
Cl	-1.10312	0.28622	-2.81771
O	1.40774	-1.01745	-0.85605
C	1.90953	-1.31567	1.72301
H	2.72595	-2.00538	1.50130
H	2.05729	-0.87389	2.70275
H	0.97113	-1.87543	1.71959
C	3.53281	0.43844	0.11986
C	4.09907	0.30286	-1.18111
C	4.41380	0.48814	1.23289
C	5.40413	-0.16060	-1.26819
C	5.71858	0.03692	1.05788
C	6.21172	-0.39257	-0.16306
H	5.81245	-0.33654	-2.25475
H	6.37452	0.03406	1.91725
C	-2.00808	2.15867	0.83741
C	-2.53714	2.89035	-0.25015
C	-2.84010	1.26819	1.56285
C	-3.78001	2.50475	-0.74688
C	-4.06054	0.92439	0.99368
C	-4.52549	1.47453	-0.19413
H	-4.17378	3.01954	-1.60846
H	-4.68411	0.20392	1.50460
C	-1.85204	4.13898	-0.84075
C	-2.54384	0.76187	2.99331
C	-5.83302	0.97911	-0.80317
C	4.13647	1.17501	2.58623
C	3.45132	0.76075	-2.50190
C	7.59815	-1.00010	-0.33555
C	-3.85973	0.59006	3.76863
H	-3.62898	0.39839	4.81923
H	-4.48157	1.48655	3.71414
H	-4.44529	-0.25974	3.41519
C	-1.72383	1.78287	3.78433
H	-0.69933	1.89778	3.40998
H	-2.19346	2.76926	3.77703
H	-1.63018	1.45287	4.82241
C	-1.82584	-0.58997	3.01720
H	-2.38068	-1.34676	2.45783
H	-0.82443	-0.51738	2.59344
H	-1.73106	-0.93978	4.04952
C	-6.97971	1.17974	0.19471
H	-7.91872	0.82515	-0.23892
H	-6.81503	0.62702	1.12162
H	-7.09926	2.23635	0.44807
C	-6.18617	1.71616	-2.09234
H	-7.11285	1.30677	-2.50131
H	-6.34573	2.78454	-1.92335
H	-5.40958	1.59757	-2.85197
C	-5.69298	-0.51387	-1.12506
H	-4.89293	-0.68466	-1.84936
H	-5.46744	-1.10688	-0.23666
H	-6.62461	-0.89488	-1.55234
C	-2.74323	4.84283	-1.86728
H	-3.71188	5.12431	-1.44811
H	-2.24532	5.75911	-2.19258
H	-2.91148	4.23008	-2.75590
C	-1.60052	5.14361	0.29270
H	-2.53623	5.41186	0.78915
H	-0.91768	4.75232	1.05014
H	-1.15412	6.05662	-0.11142
C	-0.53286	3.82681	-1.55153
H	0.24729	3.49214	-0.86304
H	-0.66121	3.05188	-2.30845
H	-0.15657	4.73256	-2.03624
C	2.75725	1.80966	2.70235
H	2.54906	2.46449	1.85389
H	1.94148	1.09423	2.79172
H	2.71990	2.42783	3.60258
C	5.12995	2.35305	2.66694
H	4.95755	2.91021	3.59181
H	6.16820	2.02081	2.66332
H	4.99116	3.03686	1.82638
C	4.39294	0.25848	3.78663
H	5.41830	-0.11494	3.79993
H	4.23534	0.81563	4.71387
H	3.73021	-0.60759	3.80167
C	2.26070	1.68656	-2.27590
H	1.92603	2.08561	-3.23608
H	1.39808	1.19017	-1.84265
H	2.53968	2.53150	-1.64062
C	3.07215	-0.41535	-3.40596
H	2.31195	-1.04291	-2.94732
H	2.68546	-0.03823	-4.35651
H	3.94670	-1.03419	-3.62353
C	4.48801	1.62047	-3.25381
H	4.86059	2.43541	-2.62833
H	5.34301	1.04595	-3.61118
H	4.00648	2.05925	-4.13115
C	8.31769	-1.18741	0.99742

H	8.51221	-0.23395	1.49528
H	7.74850	-1.82395	1.67961
H	9.28368	-1.66761	0.82534
C	8.45440	-0.08913	-1.22345
H	8.01753	0.03930	-2.21560
H	8.56778	0.90101	-0.77524
H	9.45077	-0.52069	-1.35169
C	7.45561	-2.37268	-1.00431
H	6.86100	-3.04998	-0.38656
H	6.97220	-2.29936	-1.98038
H	8.44111	-2.82271	-1.15115
Au	-2.13066	-2.74831	-0.11761
Cl	-3.40341	-4.52994	0.52715
Sn	-0.74391	-0.74245	-0.72355

Coordination through O atom

R= H

P	0.74314	-0.01583	1.04752
C	0.52740	-0.12731	-0.59086
P	-0.98612	0.04417	-1.44087
Cl	1.20472	-2.54805	-2.80579
Sn	1.78757	-0.24802	-2.50362
O	-0.47798	0.41083	-2.86185
C	-2.15471	1.23638	-0.80459
H	-3.04228	1.25813	-1.43993
H	-2.43393	0.94830	0.21232
H	-1.68962	2.22298	-0.79045
Au	-1.82141	0.77923	-4.43424
Cl	-3.26471	1.19392	-6.07190
H	2.16624	-0.17072	1.09164
H	-1.67202	-1.18353	-1.47584

R = Me

P	0.70647	-0.11544	1.03206
C	0.51283	-0.15189	-0.60896
P	-1.02575	-0.01046	-1.42374
Cl	1.44122	-2.47422	-2.90967
Sn	1.73559	-0.11533	-2.54645
O	-0.53314	0.33854	-2.86656
C	2.51186	-0.30622	1.30724
H	2.70098	-1.33330	1.63513
H	3.10591	-0.10390	0.41533
H	2.82575	0.35450	2.11872
C	-1.94088	-1.54720	-1.43385
H	-2.26451	-1.78973	-0.41956
H	-2.80908	-1.45028	-2.08965
H	-1.28186	-2.33223	-1.80887
C	-2.10832	1.27628	-0.80596
H	-3.01234	1.32541	-1.41647
H	-2.37280	1.05803	0.23136
H	-1.58737	2.23317	-0.85187
Au	-1.87188	0.71938	-4.43402
Cl	-3.31556	1.14308	-6.07120

R = *t*-Bu

P	0.79999	-0.59565	0.86954
C	0.45339	-0.11457	-0.67574
P	-1.18444	-0.04990	-1.30768
Cl	2.00813	-1.19962	-3.58395
Sn	1.40640	0.87524	-2.52245
O	-0.91105	0.59155	-2.71054
C	-2.23262	1.04500	-0.34153
H	-3.21653	1.14759	-0.80127
H	-2.32780	0.66336	0.67641
H	-1.75399	2.02493	-0.31037
Au	-2.27433	1.68298	-3.87030
Cl	-3.70819	2.86351	-5.09439
C	2.65017	-0.49965	1.13636
C	3.09248	-1.94491	1.38832
C	3.44154	0.10015	-0.00919
C	2.84258	0.32440	2.41215
H	2.51513	-2.41202	2.19111
H	2.98844	-2.55887	0.49046
H	4.14592	-1.95862	1.68671
H	3.14443	1.13522	-0.20545
H	4.50803	0.11863	0.23973
H	3.33854	-0.49292	-0.92305
H	3.89673	0.30743	2.70680
H	2.55142	1.36802	2.26711
H	2.25807	-0.07664	3.24470
C	-2.02678	-1.66985	-1.49921
C	-3.32658	-1.46776	-2.28174
C	-1.09979	-2.60840	-2.27088
C	-2.32985	-2.24295	-0.11494
H	-4.03435	-0.82026	-1.76015
H	-3.14802	-1.05134	-3.27467
H	-3.80621	-2.44262	-2.40817
H	-0.16686	-2.79342	-1.73661
H	-1.61138	-3.56513	-2.40819
H	-0.84785	-2.21314	-3.25572
H	-2.76526	-3.23817	-0.23996
H	-1.42997	-2.35030	0.49571
H	-3.05234	-1.63608	0.43513

R = Ph

P	0.67819	0.16003	0.90746
C	0.51033	-0.26580	-0.68483
P	-0.96250	-0.18680	-1.61552
Cl	1.76547	-2.85136	-2.65672
Sn	1.90810	-0.45531	-2.47029
O	-0.37141	-0.14143	-3.05752
C	-1.96968	1.26355	-1.30108
H	-2.83718	1.25984	-1.96336
H	-2.29853	1.26960	-0.25989
H	-1.36595	2.15239	-1.48975
Au	-1.47828	0.29567	-4.78308
Cl	-2.64428	0.77618	-6.61533
C	2.43488	-0.12413	1.25086
C	3.18563	-1.13274	0.63892
C	3.04723	0.69844	2.19862
C	4.52922	-1.28404	0.94124
H	2.70346	-1.83381	-0.03642
C	4.39446	0.55306	2.48903
H	2.46539	1.46552	2.70053
C	5.13653	-0.43537	1.85790
H	5.10109	-2.07452	0.46889
H	4.86550	1.20783	3.21309
H	6.18801	-0.55566	2.09220
C	-2.00035	-1.63164	-1.40967
C	-2.79151	-1.76650	-0.26891
C	-1.99424	-2.63129	-2.37817
C	-3.57960	-2.89198	-0.10597
H	-2.79026	-1.00092	0.49993
C	-2.78898	-3.75453	-2.20874
H	-1.36949	-2.53178	-3.25694
C	-3.57989	-3.88435	-1.07784
H	-4.19527	-2.99577	0.77968
H	-2.78713	-4.52967	-2.96561
H	-4.20000	-4.76413	-0.94973

R = Mes

P	0.62038	-0.19467	1.26178
C	0.37012	-0.34263	-0.36662
P	-1.03924	-0.21908	-1.40278
Cl	2.08621	-2.50334	-2.44748
Sn	1.94508	-0.14045	-1.97704
O	-0.31323	0.08192	-2.75242
Au	-0.99348	1.32479	-4.29898
Cl	-1.68201	2.66269	-5.93828
C	2.44955	-0.24704	1.36956
C	3.11444	-1.48148	1.44640
C	3.16966	0.95415	1.46244
C	4.49793	-1.48498	1.57553
C	4.55188	0.89969	1.58977
C	5.23575	-0.30897	1.63994
H	5.01336	-2.43901	1.63628
H	5.10897	1.82949	1.65932
C	-2.05203	-1.71579	-1.47668
C	-2.78082	-2.07599	-0.32375
C	-2.12140	-2.53153	-2.63065
C	-3.54900	-3.23199	-0.33780
C	-2.91643	-3.67078	-2.58065
C	-3.63624	-4.04459	-1.45619
H	-4.09406	-3.50207	0.56149
H	-2.96385	-4.29570	-3.46667
C	2.47618	2.28387	1.41393
H	2.12835	2.51939	0.40301
H	1.59687	2.30605	2.06569
H	3.14661	3.08506	1.72697
C	6.72921	-0.34446	1.74751
H	7.18668	-0.39810	0.75465
H	7.11529	0.55083	2.23767
H	7.06738	-1.21743	2.30863
C	2.36513	-2.77923	1.39504
H	1.48795	-2.76380	2.04973
H	2.00997	-2.99154	0.38254
H	3.00308	-3.60672	1.70778
C	-2.81317	-1.26954	0.94309
H	-3.59560	-0.50591	0.90007
H	-3.04633	-1.91705	1.78908
H	-1.87070	-0.77278	1.17621
C	-1.40573	-2.27510	-3.92406
H	-1.70023	-1.32559	-4.37618
H	-0.32405	-2.25267	-3.79327
H	-1.64484	-3.06882	-4.63167
C	-4.49047	-5.27299	-1.46171
H	-4.70631	-5.61419	-0.44831
H	-5.44667	-5.07344	-1.95514
H	-4.00932	-6.08777	-2.00610
C	-2.08280	1.20136	-1.03683
H	-2.90904	1.23858	-1.74929
H	-2.46882	1.16445	-0.02008
H	-1.46880	2.09564	-1.15856

R = Mes*

P	1.87690	2.16453	-0.28398
C	0.94142	0.79737	-0.31526
P	-0.78709	0.51699	-0.46760
C	3.56251	1.40562	-0.26017
C	4.28687	1.36512	0.96067
C	4.04861	0.69916	-1.38014
C	5.29531	0.42168	1.08299
C	5.04392	-0.26024	-1.16751
C	5.64254	-0.45971	0.06255
H	5.82614	0.34847	2.02081
H	5.37084	-0.85378	-2.00998
C	3.68969	0.98326	-2.85608
C	4.04829	2.35865	2.11328
C	6.68788	-1.54128	0.31374
C	2.69846	2.14717	2.80385
H	1.85409	2.39560	2.15656
H	2.57352	1.11144	3.12355
H	2.62933	2.79869	3.68002
C	4.13524	3.79031	1.56677
H	5.10843	3.97128	1.10366
H	3.36297	3.99979	0.82366
H	4.00717	4.50728	2.38253
C	5.12016	2.23480	3.19911
H	5.07106	1.27578	3.72002
H	6.12869	2.36608	2.79968
H	4.95511	3.01681	3.94368
C	2.92466	-0.18009	-3.49075
H	1.93455	-0.29080	-3.04317
H	2.78787	-0.00291	-4.56146
H	3.45634	-1.12656	-3.37266
C	2.89900	2.27173	-3.07674
H	1.86179	2.19847	-2.74500
H	3.36450	3.12552	-2.57748
H	2.86745	2.49308	-4.14724
C	5.00871	1.17798	-3.62804
H	5.60614	1.97803	-3.18454
H	5.62050	0.27645	-3.65374
H	4.78665	1.45158	-4.66275
C	6.91281	-2.42428	-0.91073
H	7.30726	-1.85716	-1.75801
H	7.64225	-3.20113	-0.67004
H	5.99249	-2.92266	-1.22596
C	6.21852	-2.43295	1.46972
H	6.08075	-1.86384	2.39088
H	5.26599	-2.91368	1.23572
H	6.95868	-3.21391	1.66491
C	8.02315	-0.88675	0.68749
H	7.93667	-0.27543	1.58797
H	8.77784	-1.65492	0.87773
H	8.38540	-0.24617	-0.12057
C	-2.23936	1.37415	0.23292
C	-2.83474	0.80386	1.39481
C	-3.05340	2.17048	-0.61468
C	-4.21756	0.70378	1.42315
C	-4.43306	2.03078	-0.51301
C	-5.04087	1.21216	0.42709
H	-4.67167	0.20045	2.26716
H	-5.05415	2.59424	-1.19598
C	-2.09368	0.43724	2.69397
C	-2.15987	-1.05498	3.02525
C	-2.79385	1.21290	3.82883
H	-1.58359	-1.64914	2.31788
H	-1.74662	-1.22584	4.02298
H	-3.18869	-1.42167	3.01985
H	-2.81787	2.28529	3.61966
H	-3.81683	0.87808	4.00244
H	-2.23634	1.05940	4.75631
C	-2.55811	3.32571	-1.50811
C	-3.22211	4.60154	-0.94912
C	-2.98257	3.17975	-2.97222
H	-2.96745	4.74407	0.10353
H	-2.86372	5.47054	-1.50785
H	-4.30859	4.57404	-1.03261
H	-2.53603	2.30940	-3.45299
H	-4.06592	3.08692	-3.06749
H	-2.67754	4.06646	-3.53449
C	-6.53857	0.94075	0.45344

C	-7.14946	1.55210	1.72020
C	-6.76341	-0.57650	0.45876
H	-7.00301	2.63504	1.74560
H	-8.22413	1.35280	1.74982
H	-6.70847	1.13109	2.62590
H	-6.32177	-1.05099	-0.42013
H	-6.32214	-1.04958	1.33783
H	-7.83519	-0.79286	0.46592
Cl	2.01435	-1.56419	2.13195
O	-0.83108	-0.95939	0.07800
Au	-2.41969	-2.24729	-0.41280
Cl	-4.09432	-3.61911	-0.95430
C	-1.05891	3.58648	-1.41379
H	-0.73965	3.70133	-0.37584
H	-0.44005	2.81707	-1.87125
H	-0.82115	4.51758	-1.93405
C	-0.64734	0.91286	2.70626
H	-0.00075	0.34983	2.03789
H	-0.57938	1.97561	2.45900
H	-0.23172	0.77461	3.70713
C	-7.25177	1.53025	-0.76052
H	-7.19128	2.62173	-0.78409
H	-6.84481	1.13916	-1.69632
H	-8.31061	1.26433	-0.72373
Sn	1.49530	-1.42466	-0.21651
C	-1.11950	0.31801	-2.23760
H	-0.85514	1.18392	-2.83717
H	-0.51253	-0.53194	-2.55851
H	-2.17022	0.06215	-2.38107

Coordination through P(sp^2) atom

R= H

P	0.67524	0.00286	0.91186
C	0.41429	-0.23392	-0.68171
P	-1.09999	-0.18773	-1.56555
Cl	1.17242	-2.80753	-2.72867
Sn	1.54920	-0.43510	-2.71759
O	-0.63016	0.01623	-3.00536
C	-2.29149	1.03890	-1.04491
H	-3.16158	0.99170	-1.70300
H	-2.60016	0.84089	-0.01540
H	-1.84145	2.03054	-1.10705
Au	-0.68395	0.42402	2.59549
Cl	-2.12108	0.87669	4.27753
H	2.07052	-0.08545	1.11248
H	-1.74779	-1.42662	-1.39148

R = Me

P	0.73095	0.02971	0.87470
C	0.41248	-0.18726	-0.70878
P	-1.15986	-0.24548	-1.49477
Cl	1.34885	-2.81856	-2.64833
Sn	1.43007	-0.40774	-2.78954
O	-0.76486	-0.18296	-2.97820
C	2.50742	-0.00335	1.21765
H	2.71831	-0.83961	1.88880
H	3.08156	-0.11391	0.29846
H	2.79013	0.91640	1.73450
C	-2.01311	-1.76896	-1.09770
H	-2.27157	-1.79156	-0.03700
H	-2.92076	-1.84110	-1.70090
H	-1.34949	-2.60147	-1.33745
C	-2.24706	1.11272	-1.06133
H	-3.15790	1.04705	-1.66040
H	-2.50286	1.06939	-0.00032
H	-1.74423	2.05648	-1.27498
Au	-0.69312	0.32792	2.54153
Cl	-2.20158	0.63318	4.20193

R = *t*-Bu

P	0.73088	0.09566	0.82464
C	0.45760	-0.32911	-0.72948
P	-1.08757	-0.46456	-1.57326
Cl	2.05350	-2.79744	-2.69626
Sn	1.56941	-0.43296	-2.77994
O	-0.62337	-0.57266	-3.03803
C	-2.08822	1.01337	-1.34703
H	-2.99723	0.94901	-1.94687
H	-2.34501	1.15250	-0.29551
H	-1.50143	1.86858	-1.68545
Au	-0.74698	0.66137	2.38477
Cl	-2.28602	1.27607	3.93411
C	2.51771	0.11946	1.31177
C	2.80824	1.50212	1.89932
C	2.67349	-0.94855	2.39966
C	3.44094	-0.17540	0.14175
H	2.71698	2.28958	1.14735
H	2.13555	1.74040	2.72681
H	3.83230	1.51816	2.28400
H	2.49501	-1.95249	2.00782
H	3.69605	-0.91269	2.78756
H	1.99206	-0.77736	3.23671
H	4.47640	-0.18385	0.49556
H	3.23707	-1.15116	-0.30566
H	3.37159	0.59549	-0.63071
C	-2.09681	-1.92990	-1.11661
C	-1.21165	-3.17335	-1.17938
C	-2.69135	-1.77525	0.27968
C	-3.21088	-2.03948	-2.16288
H	-0.76215	-3.30699	-2.16380
H	-0.40393	-3.13538	-0.44656
H	-1.82744	-4.05050	-0.96086
H	-3.36546	-0.92022	0.35916
H	-3.27100	-2.67254	0.51444
H	-1.92125	-1.67543	1.04787
H	-3.77973	-2.95316	-1.96902
H	-3.91111	-1.20258	-2.11282
H	-2.80703	-2.09824	-3.17493

R = Ph

P	0.71616	0.21197	0.70588
C	0.49216	-0.39420	-0.79412
P	-1.01014	-0.59388	-1.68329
Cl	1.87064	-3.23439	-2.25948
Sn	1.69621	-0.86514	-2.71083
O	-0.49210	-0.87875	-3.10067
C	-2.05630	0.86331	-1.66063
H	-2.92107	0.68915	-2.30365
H	-2.39271	1.08705	-0.64645
H	-1.48176	1.70957	-2.04007
Au	-0.79368	1.08956	2.07166
Cl	-2.41280	1.96462	3.39440
C	2.43213	0.13470	1.20049
C	2.88862	1.03725	2.16203
C	3.30667	-0.81356	0.66375
C	4.21675	1.01759	2.55362
H	2.20089	1.75558	2.59660
C	4.63012	-0.83240	1.06751
H	2.94031	-1.56056	-0.03284
C	5.08694	0.08645	2.00431

H	4.57156	1.72611	3.29270
H	5.30497	-1.57531	0.65880
H	6.12437	0.06643	2.31804
C	-1.97968	-1.96866	-1.06338
C	-2.76116	-1.84245	0.08510
C	-1.92409	-3.18362	-1.74126
C	-3.48696	-2.92713	0.54634
H	-2.80266	-0.90571	0.63128
C	-2.65547	-4.26459	-1.27365
H	-1.30572	-3.27608	-2.62565
C	-3.43495	-4.13687	-0.13396
H	-4.09286	-2.82726	1.43912
H	-2.61145	-5.21029	-1.80091
H	-4.00427	-4.98466	0.22988

R = Mes

P	0.69432	0.33363	1.11895
C	0.32165	-0.40617	-0.28917
P	-1.08847	-0.53284	-1.34238
Cl	1.80669	-3.04799	-2.01161
Sn	1.75177	-0.62532	-2.07433
O	-0.38279	-0.43424	-2.70396
Au	-0.58015	1.36398	2.61287
Cl	-1.89139	2.44340	4.11554
C	2.47976	0.26706	1.36430
C	3.23359	1.44558	1.23466
C	3.09175	-0.94805	1.71268
C	4.60446	1.37921	1.43527
C	4.46756	-0.95638	1.90757
C	5.24031	0.18981	1.77331
H	5.19322	2.28477	1.32438
H	4.94819	-1.89169	2.17755
C	-2.01412	-2.07715	-1.14647
C	-2.63024	-2.36525	0.08798
C	-2.14410	-2.99051	-2.21946
C	-3.30533	-3.56747	0.25060
C	-2.84537	-4.16979	-1.99645
C	-3.41724	-4.49169	-0.77479
H	-3.76293	-3.77785	1.21251
H	-2.94297	-4.86754	-2.82218
C	2.59410	2.75165	0.86992
H	2.11567	2.70499	-0.11315
H	1.82128	3.03595	1.59101
H	3.33479	3.55104	0.84097
C	2.31297	-2.21800	1.88367
H	1.40630	-2.05977	2.47483
H	2.00264	-2.63113	0.91975
H	2.91746	-2.96934	2.39267
C	6.71687	0.15423	2.01874
H	6.94103	0.42362	3.05563
H	7.12625	-0.84157	1.84253
H	7.24412	0.86268	1.37733
C	-2.65341	-1.41983	1.25114
H	-2.73677	-1.97010	2.18901
H	-1.76667	-0.79282	1.32536
H	-3.52034	-0.75421	1.19525
C	-1.59605	-2.79607	-3.60479
H	-1.85459	-1.82658	-4.02762
H	-0.50756	-2.86743	-3.61105
H	-1.99084	-3.57567	-4.25718
C	-4.11696	-5.79848	-0.57050
H	-4.62013	-6.12718	-1.48161
H	-3.39914	-6.57764	-0.29563
H	-4.85579	-5.73544	0.22993
C	-2.23461	0.84461	-1.19070
H	-2.68980	0.88749	-0.20189
H	-1.68125	1.76710	-1.37340
H	-3.01438	0.73379	-1.94701

R = Mes*

P	-1.04304	0.95903	0.04330
C	-0.19471	-0.36960	0.47801
P	1.44737	-0.95275	0.77332
Cl	-1.45203	-3.13709	-1.58144
O	1.24827	-2.44575	0.43104
C	3.08145	-0.45965	0.10646
C	3.62060	-1.27891	-0.92593
C	3.98275	0.29419	0.90408
C	4.96901	-1.59928	-0.85787
C	5.32438	-0.07662	0.90413
C	5.82976	-1.08731	0.10333
H	5.36492	-2.27870	-1.60175
H	5.99940	0.46577	1.55144
C	-2.79730	0.45608	0.09097
C	-3.47567	0.09961	-1.10445
C	-3.38158	0.16927	1.34988
C	-4.58291	-0.73480	-0.98862
C	-4.47441	-0.69201	1.37117
C	-5.06054	-1.20400	0.22550
H	-5.07813	-1.05448	-1.89003
H	-4.89010	-0.96779	2.33177
C	-3.13055	0.65338	-2.50115
C	-3.00298	0.78756	2.71498
C	-6.20616	-2.20526	0.33185
C	3.66741	1.61334	1.63762
C	2.88166	-1.73506	-2.19720
C	7.27393	-1.56718	0.17905
C	-4.28821	1.35483	3.34744
H	-4.04033	1.84077	4.29447
H	-4.74735	2.10007	2.69377
H	-5.03382	0.58923	3.56006
C	-2.03537	1.96757	2.65286
H	-1.00961	1.67235	2.42880
H	-2.34923	2.72219	1.92766
H	-2.00588	2.45160	3.63285
C	-2.41911	-0.27449	3.65061
H	-3.09630	-1.12181	3.77539
H	-1.47142	-0.65543	3.26376
H	-2.23219	0.15640	4.63842
C	-7.37212	-1.57717	1.10403
H	-8.19591	-2.29155	1.18571
H	-7.08244	-1.28800	2.11634
H	-7.74454	-0.68558	0.59364
C	-6.71897	-2.64722	-1.03619
H	-7.51875	-3.37982	-0.90443
H	-7.13103	-1.81134	-1.60729
H	-5.93277	-3.11844	-1.63088
C	-5.70860	-3.44731	1.08144
H	-4.88185	-3.92060	0.54616
H	-5.36173	-3.20143	2.08745
H	-6.51643	-4.17823	1.17591
C	-4.04866	0.08413	-3.58746
H	-5.10053	0.31904	-3.41000
H	-3.77365	0.53706	-4.54227
H	-3.93944	-0.99807	-3.69214
C	-3.35936	2.17102	-2.47674
H	-4.39675	2.40196	-2.22174
H	-2.71573	2.67583	-1.75285
H	-3.14530	2.59778	-3.46023
C	-1.70293	0.33976	-2.95481
H	-0.94027	0.87087	-2.38177
H	-1.49384	-0.72876	-2.89622

H	-1.57685	0.66618	-3.99094
C	2.25046	2.12293	1.41135
H	2.03190	2.18250	0.34321
H	1.46995	1.52920	1.88272
H	2.15686	3.13463	1.81187
C	4.57429	2.68520	0.99563
H	4.34310	3.66049	1.43157
H	5.63380	2.48706	1.15886
H	4.40087	2.74855	-0.08064
C	3.99344	1.56236	3.13337
H	5.04054	1.30582	3.30624
H	3.81797	2.54461	3.57997
H	3.38513	0.83647	3.67311
C	1.53607	-1.04561	-2.39148
H	1.16738	-1.25942	-3.39743
H	0.76917	-1.40505	-1.71129
H	1.62776	0.03975	-2.29541
C	2.71215	-3.25554	-2.25017
H	2.08859	-3.60847	-1.43111
H	2.23872	-3.54063	-3.19350
H	3.67947	-3.76165	-2.19525
C	3.74113	-1.29671	-3.40034
H	3.92245	-0.21915	-3.38559
H	4.70591	-1.80274	-3.44433
H	3.20468	-1.53737	-4.32158
C	8.04796	-0.89662	1.31082
H	8.13921	0.18217	1.16097
H	7.57857	-1.07185	2.28220
H	9.05983	-1.30643	1.35292
C	7.98885	-1.26141	-1.14256
H	7.51209	-1.76181	-1.98755
H	7.99238	-0.18793	-1.34648
H	9.02664	-1.60271	-1.09527
C	7.28034	-3.08129	0.42124
H	6.78888	-3.32883	1.36532
H	6.76425	-3.62055	-0.37540
H	8.30861	-3.45053	0.46521
Sn	-0.95938	-2.57224	0.71914
Au	-0.38701	3.01376	-0.51611
Cl	0.27845	5.12142	-1.04749
C	1.64038	-0.95342	2.57701
H	2.59153	-1.43378	2.81221
H	1.58567	0.01792	3.05644
H	0.83338	-1.58271	2.96016

Coordination through π(C=P) bond

R= H

P	0.85188	-0.12399	1.06203
C	0.54016	0.06450	-0.61974
P	-1.00470	-0.14556	-1.45334
Cl	1.42426	-2.67790	-2.33647
Sn	1.60765	-0.29712	-2.68808
O	-0.61191	-0.05961	-2.92686
C	-2.33753	0.97664	-1.04299
H	-3.20949	0.72686	-1.65140
H	-2.59015	0.87913	0.01519
H	-2.03157	2.00275	-1.25240
Au	0.85145	2.06506	0.10287
Cl	1.05635	4.30628	0.16054
H	2.27440	-0.25453	1.00043
H	-1.49270	-1.42334	-1.11344

R = Me

P	0.74859	-0.12825	1.04354
C	0.45715	0.14730	-0.62601
P	-1.05794	-0.16708	-1.49439
Cl	1.76719	-2.55848	-2.16259
Sn	1.60738	-0.18049	-2.64341
O	-0.58680	-0.21716	-2.95472
C	2.56478	-0.35760	1.16645
H	2.74798	-1.43675	1.12619
H	3.12194	0.12506	0.36383
H	2.91282	0.00941	2.13397
C	-1.75027	-1.73884	-0.97952
H	-2.06838	-1.69264	0.06396
H	-2.60302	-1.98354	-1.61616
H	-0.97917	-2.50223	-1.09879
C	-2.34290	1.06951	-1.29189
H	-3.19104	0.80288	-1.92686
H	-2.66673	1.11781	-0.25063
H	-1.96260	2.04546	-1.59611
Au	0.61400	2.11862	0.21229
Cl	0.69219	4.35690	0.48190

R = *t*-Bu

P	1.06166	-0.06261	1.10549
C	0.59437	0.12266	-0.53928
P	-1.00593	-0.28670	-1.22347
Cl	2.22238	-2.37499	-2.41518
Sn	1.50396	-0.07304	-2.70761
O	-0.65235	-0.55010	-2.69796
C	-1.83237	-1.75838	-0.49366
Au	0.68965	2.13780	0.23311
Cl	0.65833	4.38493	0.46010
C	2.92704	-0.15099	1.23183
C	3.71915	0.33931	0.03374
C	3.28930	0.65465	2.48098
C	3.20424	-1.64014	1.47579
H	3.56978	-0.30593	-0.83525
H	3.47302	1.37091	-0.22991
H	4.78894	0.30445	0.26599
H	2.69985	0.34561	3.34896
H	4.34504	0.49760	2.72240
H	3.13304	1.72565	2.32970
H	4.26806	-1.77503	1.69631
H	2.64055	-2.02541	2.33058
H	2.96350	-2.24201	0.59609
C	-2.16355	1.09073	-1.13271
H	-2.39565	1.35854	-0.10197
H	-1.71349	1.95173	-1.62950
H	-3.08021	0.82492	-1.66268
C	-2.37152	-1.43227	0.89821
H	-2.80877	-2.33873	1.32673
H	-1.58346	-1.09600	1.57666
H	-3.15557	-0.67274	0.87038
C	-0.83429	-2.91302	-0.42059
H	-0.03157	-2.71495	0.29162
H	-1.36306	-3.80989	-0.08516
H	-0.37969	-3.12468	-1.38935
C	-2.97846	-2.14149	-1.43527
H	-3.45218	-3.05110	-1.05518
H	-3.74964	-1.36977	-1.48819
H	-2.61608	-2.34125	-2.44480

R = Ph

P	0.61932	0.11764	0.98191
C	0.38094	0.17599	-0.71762
P	-1.15098	-0.08446	-1.57198
Cl	1.77909	-2.23886	-2.93819
Sn	1.47604	0.16645	-2.80389

O	-0.73409	0.06027	-3.04070
C	-2.47604	1.06163	-1.17468
H	-3.34644	0.81327	-1.78547
H	-2.74186	0.98857	-0.11863
H	-2.15107	2.07965	-1.39481
Au	0.61533	2.25076	-0.12017
Cl	0.79385	4.50050	-0.12707
C	2.39925	-0.13401	1.22660
C	2.96408	0.39641	2.38883
C	3.18877	-0.89792	0.36637
C	4.30891	0.20923	2.65965
H	2.34929	0.97128	3.07462
C	4.52991	-1.09799	0.65173
H	2.74892	-1.36642	-0.50775
C	5.09279	-0.53716	1.79012
H	4.74571	0.64128	3.55253
H	5.13581	-1.69880	-0.01679
H	6.14366	-0.69177	2.00677
C	-1.78479	-1.73117	-1.23680
C	-1.79273	-2.67283	-2.26082
C	-2.27395	-2.06721	0.02508
C	-2.29581	-3.94327	-2.02296
H	-1.39558	-2.40820	-3.23284
C	-2.77290	-3.33735	0.25632
H	-2.25681	-1.34480	0.83499
C	-2.78513	-4.27451	-0.76908
H	-2.29761	-4.67737	-2.82003
H	-3.15038	-3.59858	1.23800
H	-3.17457	-5.26941	-0.58549

R = Mes

P	0.56280	0.08271	1.35659
C	0.15285	0.26884	-0.30660
P	-1.30610	-0.00426	-1.30398
Cl	2.12016	-1.37421	-2.92884
Sn	1.35962	0.82985	-2.23878
O	-0.78303	0.45170	-2.67383
Au	0.03934	2.27368	0.52660
Cl	-0.29775	4.50367	0.71534
C	2.38587	-0.06019	1.36795
C	3.23130	0.91860	1.91249
C	2.91103	-1.30510	0.97129
C	4.59914	0.67287	1.94952
C	4.28320	-1.50225	1.03487
C	5.14867	-0.51857	1.49687
H	5.25303	1.43655	2.36048
H	4.68458	-2.46280	0.72569
C	-1.82827	-1.73906	-1.27232
C	-1.78339	-2.54599	-2.43282
C	-2.30271	-2.28701	-0.06312
C	-2.14822	-3.88295	-2.31796
C	-2.64185	-3.63230	-0.01119
C	-2.55647	-4.45532	-1.12278
H	-2.10979	-4.49953	-3.21048
H	-2.99456	-4.04215	0.93055
C	2.71916	2.21363	2.46770
H	2.52578	2.95049	1.68201
H	1.78557	2.08259	3.02193
H	3.45005	2.65140	3.14942
C	2.02927	-2.43636	0.53037
H	1.16417	-2.55849	1.19114
H	1.65222	-2.28496	-0.48339
H	2.58541	-3.37464	0.53936
C	6.62911	-0.74225	1.51225
H	6.87252	-1.78488	1.72558
H	7.06515	-0.49967	0.53807
H	7.12022	-0.11404	2.25722
C	-2.54643	-1.48968	1.18667
H	-2.42012	-2.11948	2.06823
H	-1.88312	-0.63523	1.31058
H	-3.57601	-1.11773	1.20141
C	-1.39681	-2.07144	-3.80401
H	-1.93258	-1.16945	-4.09689
H	-0.33237	-1.84101	-3.86248
H	-1.61575	-2.85604	-4.52907
C	-2.88667	-5.91237	-1.03744
H	-3.32346	-6.27660	-1.96898
H	-1.98209	-6.49955	-0.85064
H	-3.58469	-6.11737	-0.22415
C	-2.75107	1.01148	-0.94052
H	-2.47352	2.05974	-1.06506
H	-3.52209	0.75746	-1.67205
H	-3.13909	0.85545	0.06364

R = Mes*

P	0.68890	-0.04664	1.16220
C	0.39749	0.08040	-0.54479
P	-1.06162	-0.12174	-1.62624
O	-0.33331	-0.11653	-2.98440
Cl	2.84466	-1.33981	-3.23497
Au	0.29580	2.13274	0.17552
Cl	-0.07742	4.37762	0.15454
C	2.54294	-0.08611	1.25979
C	3.25599	-1.20772	0.75393
C	3.28302	1.05106	1.66445
C	4.55742	-1.01069	0.31720
C	4.58529	1.18368	1.17721
C	5.21677	0.20881	0.42564
H	5.08608	-1.85187	-0.11186
H	5.12656	2.08695	1.42194
C	2.88996	2.07025	2.75588
C	2.80950	-2.67557	0.90451
C	6.59794	0.39440	-0.19189
C	-2.09684	1.36394	-1.70076
H	-2.61957	1.60732	-0.78132
H	-1.46110	2.20433	-1.98524
H	-2.82193	1.18525	-2.49803
C	3.89725	1.84768	3.90347
H	3.83718	0.82470	4.28373
H	4.92542	2.02864	3.58871
H	3.67019	2.53220	4.72540
C	3.01308	3.51958	2.27905
H	2.31671	3.74486	1.47027
H	2.78679	4.19804	3.10623
H	4.02156	3.75200	1.93277
C	1.51324	1.87150	3.38312
H	1.43319	2.51292	4.26490
H	0.69167	2.15688	2.72267
H	1.35692	0.84093	3.71160
C	3.12459	-3.52522	-0.32853
H	2.65716	-3.12190	-1.22519
H	4.19656	-3.59726	-0.51825
H	2.75964	-4.54373	-0.16944
C	3.62418	-3.22275	2.09257
H	4.69677	-3.16167	1.90100
H	3.41285	-2.66146	3.00638
H	3.36645	-4.27179	2.26666
C	1.34221	-2.88076	1.26096
H	1.06102	-2.35006	2.17603
H	0.66943	-2.58908	0.45486
H	1.16870	-3.94382	1.45167
C	7.17123	1.78253	0.07971

H	6.52331	2.57183	-0.30945
H	7.32722	1.95913	1.14729
H	8.14145	1.87849	-0.41355
C	6.48393	0.20597	-1.70944
H	7.46830	0.30357	-2.17610
H	6.07962	-0.77432	-1.96827
H	5.82399	0.95832	-2.14821
C	7.57014	-0.64573	0.37693
H	7.65965	-0.54703	1.46175
H	7.25114	-1.66611	0.15612
H	8.56290	-0.50833	-0.06045
C	-2.35464	-1.44373	-1.65252
C	-2.33248	-2.35422	-2.75038
C	-3.60193	-1.21356	-1.00947
C	-3.53908	-2.65589	-3.36694
C	-1.11488	-3.15970	-3.24045
C	-4.76808	-1.58515	-1.67264
C	-3.81522	-0.75060	0.44530
C	-4.77062	-2.21601	-2.90452
H	-3.50987	-3.28448	-4.24722
C	-0.61212	-2.69905	-4.61136
C	-1.54699	-4.63467	-3.37696
C	0.00310	-3.17845	-2.20698
H	-5.71273	-1.37084	-1.19291
C	-2.53452	-0.42350	1.19255
C	-4.42909	-1.95957	1.18057
C	-4.78930	0.42579	0.56779
C	-6.04220	-2.51220	-3.68961
H	0.19877	-3.35288	-4.94301
H	-1.41434	-2.75699	-5.35227
H	-0.24061	-1.67880	-4.58179
H	-2.23234	-4.80535	-4.20786
H	-0.65751	-5.24072	-3.56661
H	-2.01889	-5.00109	-2.46197
H	0.44419	-2.20289	-2.02094
H	-0.36079	-3.59495	-1.26433
H	0.81691	-3.81229	-2.56389
H	-1.81170	-1.23744	1.11551
H	-2.05830	0.49910	0.86118
H	-2.75383	-0.28621	2.25392
H	-4.57669	-1.70791	2.23445
H	-5.39425	-2.24931	0.76378
H	-3.76472	-2.82522	1.12638
H	-5.77150	0.19245	0.15391
H	-4.92994	0.67298	1.62323
H	-4.42509	1.32265	0.06464
C	-7.28415	-1.93029	-3.01985
C	-6.22804	-4.02882	-3.81818
C	-5.91451	-1.89633	-5.08804
H	-7.46969	-2.37994	-2.04102
H	-7.20677	-0.84747	-2.89321
H	-8.16039	-2.12942	-3.64121
H	-5.39174	-4.49785	-4.34001
H	-6.31567	-4.49728	-2.83480
H	-7.13865	-4.24850	-4.38227
H	-5.79612	-0.81166	-5.02883
H	-5.05564	-2.29731	-5.62972
H	-6.81144	-2.10933	-5.67599
Sn	1.68048	0.64198	-2.45515

[R-P=C(Sn(II)Cl)-P(S)ClR](AuCl)

Coordination through Sn(II) atom

R= H

P	0.68673	0.29534	0.99904
C	0.43878	-0.11822	-0.58909
P	-1.11866	-0.29764	-1.36007
Cl	1.62196	-2.37551	-2.90618
Sn	1.63754	-0.06445	-2.45401
Au	3.47472	1.53095	-3.05247
Cl	5.13893	2.99612	-3.51955
Cl	-2.66537	0.51450	-0.36865
H	2.11229	0.38082	0.94678
H	-1.49870	-1.65074	-1.36160
S	-0.84999	0.42691	-3.19329

R = Me

P	0.66074	0.10158	1.05053
C	0.39260	-0.18564	-0.56077
P	-1.18176	-0.26045	-1.31527
Cl	1.58887	-2.38499	-2.97388
Sn	1.57508	-0.08183	-2.42554
C	2.48167	0.10759	1.22498
H	2.78593	-0.86745	1.61993
H	3.01607	0.29893	0.29323
H	2.76160	0.85846	1.96714
C	-1.83622	-1.93050	-1.33599
H	-2.01141	-2.26511	-0.31231
H	-2.76677	-1.94314	-1.90496
H	-1.09146	-2.56480	-1.82230
Au	3.49915	1.44983	-2.92009
Cl	5.25858	2.84589	-3.24640
Cl	-2.60512	0.78840	-0.33606
S	-0.85655	0.46460	-3.15769

R = *t*-Bu

P	0.65693	-0.39556	0.82998
C	0.65203	-0.13420	-0.81147
P	-0.86477	-0.14934	-1.71395
Cl	2.76904	-1.40805	-3.33174
Sn	1.97127	0.62456	-2.42116
Au	3.34749	2.72612	-2.45361
Cl	4.58260	4.63345	-2.37692
C	2.40477	-0.36366	1.47642
C	2.55698	-1.70491	2.20499
C	3.50744	-0.19321	0.45015
C	2.43418	0.77908	2.49788
H	1.75893	-1.86819	2.93464
H	2.56143	-2.54609	1.50723
H	3.50855	-1.71269	2.74560
H	3.48242	0.79611	-0.01526
H	4.48246	-0.27720	0.94119
H	3.47135	-0.96219	-0.32655
H	3.38966	0.76032	3.03142
H	2.33947	1.75470	2.01559
H	1.63730	0.68432	3.24080
C	-1.65410	-1.81124	-1.92184
C	-2.85620	-1.65237	-2.85103
C	-0.60964	-2.72808	-2.55916
C	-2.08810	-2.36037	-0.56646
H	-3.62667	-1.01177	-2.41895
H	-2.56587	-1.24771	-3.82245
H	-3.29342	-2.64068	-3.01668
H	0.26697	-2.85918	-1.92302
H	-1.06835	-3.70881	-2.71208
H	-0.27267	-2.35589	-3.52738

H	-2.51197	-3.35614	-0.72296
H	-1.24856	-2.46313	0.12417
H	-2.85058	-1.73938	-0.09510
Cl	-2.28529	0.95169	-0.76563
S	-0.39549	0.70012	-3.47181

R = Ph

P	0.72934	0.24295	1.28727
C	0.30736	-0.22872	-0.25120
P	-1.32288	-0.30300	-0.87416
Cl	1.47250	-2.63777	-2.54110
Sn	1.38603	-0.29935	-2.16813
Au	3.18581	1.23346	-3.01349
Cl	4.84247	2.62772	-3.70620
C	2.52471	0.09298	1.31682
C	3.21595	1.01038	2.11300
C	3.24236	-0.88296	0.61726
C	4.59993	0.99043	2.16091
H	2.66518	1.75463	2.67969
C	4.62442	-0.90918	0.68177
H	2.71741	-1.64888	0.05424
C	5.30331	0.03420	1.44258
H	5.13091	1.72116	2.75942
H	5.17443	-1.66721	0.13681
H	6.38622	0.01742	1.48135
C	-2.11467	-1.88461	-0.55233
C	-2.27707	-2.79998	-1.58613
C	-2.50525	-2.20859	0.74714
C	-2.84097	-4.03871	-1.31926
H	-1.95903	-2.54503	-2.58952
C	-3.06488	-3.44657	1.00278
H	-2.37874	-1.49566	1.55418
C	-3.23368	-4.36095	-0.03037
H	-2.96893	-4.75252	-2.12413
H	-3.37237	-3.69971	2.01046
H	-3.67341	-5.33024	0.17452
Cl	-2.54051	1.03603	0.03886
S	-1.11376	0.10043	-2.82384

R = Mes

P	0.80724	0.03700	1.90088
C	0.25688	-0.32355	0.37246
P	-1.37427	-0.25379	-0.27931
Cl	1.17200	-2.24983	-2.40734
Sn	1.38471	-0.06964	-1.49363
Au	3.39709	1.34374	-2.01781
Cl	5.27745	2.58849	-2.36925
C	2.60907	0.04525	1.64748
C	3.26814	1.28517	1.69312
C	3.33074	-1.13888	1.42661
C	4.63274	1.32629	1.45264
C	4.69860	-1.04473	1.19844
C	5.36427	0.17504	1.18354
H	5.13546	2.28828	1.44581
H	5.26162	-1.95647	1.02292
C	-2.17666	-1.86914	-0.42698
C	-2.77264	-2.32060	-1.62783
C	-2.17576	-2.70622	0.71277
C	-3.27318	-3.61565	-1.66414
C	-2.69696	-3.98779	0.60424
C	-3.22901	-4.47552	-0.57780
H	-3.72930	-3.95806	-2.58729
H	-2.69340	-4.61953	1.48697
Cl	-2.64332	0.85032	0.87660
C	-1.65877	-2.31307	2.06439
H	-0.58428	-2.49630	2.14187
H	-1.83369	-1.26709	2.31171
H	-2.14952	-2.91550	2.82967
C	-3.74207	-5.87632	-0.67946
H	-2.95558	-6.54249	-1.04764
H	-4.06538	-6.25528	0.29118
H	-4.57912	-5.94491	-1.37622
C	-2.94383	-1.51532	-2.88194
H	-3.36592	-0.52819	-2.69444
H	-1.99462	-1.36957	-3.39985
H	-3.61761	-2.04547	-3.55549
C	2.51383	2.56068	1.92488
H	2.00693	2.89003	1.01218
H	1.74844	2.45099	2.70038
H	3.18857	3.36132	2.22946
C	2.67799	-2.49171	1.44947
H	2.01393	-2.60180	2.31227
H	2.07844	-2.67888	0.55414
H	3.43277	-3.27659	1.50932
C	6.82084	0.26440	0.85716
H	6.95266	0.73804	-0.12061
H	7.35448	0.87625	1.58829
H	7.28667	-0.72128	0.82579
S	-1.01853	0.79255	-1.96350

R = Mes*

P	-0.04048	2.03697	1.10579
C	0.32779	0.58165	0.37476
P	1.88394	-0.23138	0.17667
Cl	-1.28669	0.55868	-2.81735
C	3.61181	0.37694	0.15466
C	4.22666	0.49837	-1.12857
C	4.44869	0.22310	1.29323
C	5.52764	0.03785	-1.25527
C	5.74929	-0.22134	1.07664
C	6.28069	-0.43025	-0.18556
H	5.98074	0.06884	-2.23819
H	6.37172	-0.39847	1.94250
C	-1.86583	2.08226	0.92827
C	-2.44842	2.93658	-0.03846
C	-2.68239	1.17541	1.65144
C	-3.74919	2.65792	-0.44697
C	-3.96423	0.94051	1.16837
C	-4.50050	1.61798	0.08117
H	-4.18956	3.26912	-1.21809
H	-4.57827	0.20653	1.67222
C	-1.75610	4.20623	-0.56968
C	-2.30564	0.54808	3.01238
C	-5.88569	1.25143	-0.44050
C	4.14882	0.70152	2.72977
C	3.68057	1.29575	-2.33159
C	7.65623	-1.03983	-0.41956
C	-3.54908	0.51490	3.91728
H	-3.24650	0.22293	4.92586
H	-4.03207	1.49312	3.97487
H	-4.28779	-0.21559	3.58615
C	-1.27607	1.39482	3.75970
H	-0.28522	1.38647	3.28857
H	-1.59699	2.43502	3.85377
H	-1.12657	0.98875	4.76370
C	-1.78891	-0.88622	2.88670
H	-2.50747	-1.52399	2.36714
H	-0.84190	-0.93101	2.34867
H	-1.62569	-1.30916	3.88247
C	-6.92138	1.42096	0.67754
H	-7.91607	1.15942	0.30667
H	-6.70812	0.77568	1.53178

H	-6.95126	2.45440	1.03273
C	-6.30819	2.12319	-1.62021
H	-7.29125	1.80245	-1.97254
H	-6.38675	3.17835	-1.34435
H	-5.61257	2.03442	-2.45835
C	-5.86961	-0.21050	-0.90260
H	-5.14759	-0.35788	-1.70929
H	-5.60754	-0.89798	-0.09627
H	-6.85720	-0.49646	-1.27479
C	-2.67756	5.02123	-1.48006
H	-3.60165	5.31381	-0.97664
H	-2.15850	5.93661	-1.77336
H	-2.93299	4.48221	-2.39524
C	-1.40087	5.10123	0.62664
H	-2.29427	5.35149	1.20372
H	-0.68662	4.62437	1.30168
H	-0.94958	6.03302	0.27418
C	-0.49321	3.91166	-1.37882
H	0.30364	3.48414	-0.76346
H	-0.69134	3.21566	-2.19453
H	-0.09747	4.84303	-1.79474
C	2.77925	1.33992	2.91855
H	2.59409	2.11504	2.17193
H	1.96266	0.62145	2.89614
H	2.74443	1.82318	3.89814
C	5.16530	1.83495	2.99030
H	4.98076	2.26087	3.98012
H	6.19673	1.48296	2.96279
H	5.05995	2.63295	2.25145
C	4.36340	-0.38294	3.78871
H	5.36998	-0.80261	3.74635
H	4.23230	0.05170	4.78316
H	3.65199	-1.20072	3.68204
C	2.33203	1.96090	-2.09000
H	2.14195	2.67702	-2.89300
H	1.49559	1.26549	-2.09626
H	2.33205	2.51802	-1.14994
C	3.63006	0.48158	-3.62583
H	2.90107	-0.32512	-3.56114
H	3.34034	1.13442	-4.45324
H	4.60088	0.04766	-3.87375
C	4.68120	2.45591	-2.52740
H	4.76032	3.06330	-1.62233
H	5.68074	2.10710	-2.78830
H	4.32716	3.09811	-3.33801
C	8.31140	-1.50351	0.87857
H	8.51304	-0.67001	1.55636
H	7.69412	-2.23585	1.40474
H	9.26901	-1.97830	0.65322
C	8.57446	-0.00688	-1.08404
H	8.18820	0.31946	-2.05161
H	8.69311	0.87699	-0.45254
H	9.56400	-0.44052	-1.25127
C	7.50254	-2.25335	-1.34473
H	6.86096	-3.01286	-0.89186
H	7.06537	-1.97646	-2.30606
H	8.48005	-2.70327	-1.53758
Au	-2.58543	-2.54766	-0.35761
Cl	-4.16914	-4.09620	0.19600
Sn	-0.89205	-0.76372	-0.88550
Cl	1.91463	-1.60689	1.70298
S	1.55765	-1.38154	-1.44588

Coordination through S atom

R= H

P	0.77103	0.00051	1.10259
C	0.52617	-0.17476	-0.52843
P	-0.94664	0.18602	-1.36251
Cl	0.76408	-2.44009	-3.03691
Sn	1.88217	-0.42855	-2.37252
Au	-1.80117	0.85004	-4.60613
Cl	-3.28554	0.50567	-6.26074
Cl	-2.42106	1.07749	-0.32654
H	-1.55759	-1.00342	-1.78103
H	2.15078	-0.38235	1.13239
S	-0.29310	1.25894	-2.95635

R = Me

P	0.78405	0.03734	1.10351
C	0.52562	-0.11062	-0.52773
P	-1.00583	0.18969	-1.27971
Cl	1.19346	-2.41276	-3.04228
Sn	1.88094	-0.20597	-2.38377
C	2.53956	-0.43862	1.33965
H	2.56197	-1.46419	1.72254
H	3.13693	-0.39084	0.42794
H	2.98343	0.20123	2.10571
C	-1.92637	-1.31476	-1.58617
H	-2.17379	-1.76600	-0.62322
H	-2.83262	-1.07200	-2.14263
H	-1.28964	-1.98350	-2.16982
Au	-1.92011	0.57303	-4.62329
Cl	-3.38823	0.05042	-6.24858
Cl	-2.29119	1.32849	-0.20401
S	-0.44559	1.15247	-2.99224

R = *t*-Bu

P	0.97982	-0.06260	0.74165
C	0.69529	-0.10154	-0.89682
P	-0.92642	0.14620	-1.49274
Cl	1.93319	-2.06831	-3.60620
Au	-1.89430	0.29087	-4.99227
Cl	-3.16984	-0.37348	-6.72787
S	-0.57135	1.04559	-3.30326
C	-2.00509	-1.36918	-1.49916
C	-3.40449	-1.00769	-1.98982
C	-1.36784	-2.41706	-2.40679
C	-2.06957	-1.88747	-0.06217
H	-3.90604	-0.30377	-1.32529
H	-3.39469	-0.59667	-3.00202
H	-3.99425	-1.92766	-2.01879
H	-0.36062	-2.68710	-2.08837
H	-1.99160	-3.31436	-2.36840
H	-1.32159	-2.08881	-3.44633
H	-2.71550	-2.76953	-0.06056
H	-1.09039	-2.19208	0.30996
H	-2.49775	-1.15744	0.62630
C	2.80513	-0.38575	1.01378
C	3.31962	-1.58457	0.22858
C	3.57555	0.88623	0.65853
C	2.94022	-0.65693	2.51426
H	2.76334	-2.49420	0.46710
H	3.25825	-1.44580	-0.85264
H	4.37149	-1.76139	0.47689
H	3.19602	1.75788	1.19892
H	4.62912	0.76230	0.93059
H	3.53413	1.11021	-0.41037
H	3.99315	-0.81018	2.76884

H	2.56788	0.17927	3.11189
H	2.39064	-1.55328	2.81376
Sn	1.98156	0.18785	-2.78995
Cl	-1.97610	1.47040	-0.35360

R = Ph

P	0.74541	0.49833	0.81017
C	0.53679	-0.09477	-0.72924
P	-0.93123	0.02774	-1.64155
Cl	1.67467	-2.93408	-2.48523
Sn	2.11127	-0.57285	-2.30682
Au	-1.73649	-0.07919	-5.10257
Cl	-3.19730	-0.56461	-6.74908
C	2.43731	-0.00676	1.21327
C	3.01355	-1.20374	0.77247
C	3.18284	0.84427	2.03275
C	4.32014	-1.51737	1.11313
H	2.41962	-1.91824	0.20941
C	4.49276	0.53347	2.35980
H	2.73573	1.76141	2.40362
C	5.06275	-0.64490	1.89729
H	4.75451	-2.45081	0.77427
H	5.06919	1.20851	2.98169
H	6.08440	-0.89194	2.16200
C	-1.93026	-1.46025	-1.52381
C	-2.74208	-1.63998	-0.40311
C	-1.83878	-2.45263	-2.49408
C	-3.46807	-2.80883	-0.26723
H	-2.81652	-0.86817	0.35366
C	-2.57416	-3.61841	-2.34891
H	-1.19964	-2.32278	-3.35722
C	-3.38691	-3.79604	-1.24129
H	-4.10568	-2.94617	0.59797
H	-2.51038	-4.38502	-3.11135
H	-3.96405	-4.70735	-1.13494
Cl	-2.16290	1.50656	-0.99352
S	-0.22307	0.46183	-3.49431

R = Mes

P	0.59025	0.20907	1.20622
C	0.34157	-0.19060	-0.38560
P	-1.08163	-0.04948	-1.38081
Cl	1.82373	-2.69691	-2.28028
Sn	2.12255	-0.35434	-1.78291
Au	-1.65047	1.50611	-4.58092
Cl	-3.06347	2.28514	-6.15548
C	2.38856	-0.10692	1.37191
C	2.87412	-1.41377	1.55398
C	3.26851	0.98910	1.40267
C	4.24306	-1.59601	1.71531
C	4.62651	0.75467	1.56682
C	5.13534	-0.53089	1.71504
H	4.62074	-2.60397	1.85854
H	5.30630	1.60145	1.58510
C	-1.99323	-1.61301	-1.49492
C	-2.62131	-2.07250	-0.31196
C	-2.04458	-2.40182	-2.66992
C	-3.26742	-3.30125	-0.33066
C	-2.71921	-3.61488	-2.61339
C	-3.33646	-4.08868	-1.46696
H	-3.73624	-3.64697	0.58520
H	-2.75312	-4.21630	-3.51572
Cl	-2.44108	1.32849	-0.74421
C	2.76288	2.39162	1.23910
H	2.44189	2.58625	0.21064
H	1.89900	2.58776	1.88231
H	3.53791	3.11771	1.48645
C	6.60771	-0.76128	1.86049
H	7.08005	-0.86627	0.87878
H	7.09349	0.07388	2.36808
H	6.81485	-1.67387	2.42156
C	1.95657	-2.59994	1.59734
H	1.08370	-2.40922	2.22878
H	1.58476	-2.85887	0.60187
H	2.47642	-3.47176	1.99605
C	-2.68769	-1.32157	0.98522
H	-3.50464	-0.59651	0.97238
H	-2.87665	-2.02049	1.80068
H	-1.77904	-0.77286	1.23042
C	-1.43851	-2.05308	-3.99450
H	-1.88518	-1.15413	-4.42409
H	-0.36405	-1.88844	-3.92523
H	-1.60650	-2.87238	-4.69294
C	-4.06708	-5.39356	-1.46817
H	-4.20001	-5.77836	-0.45624
H	-5.06049	-5.27500	-1.91161
H	-3.53682	-6.14364	-2.05803
S	-0.18288	0.77478	-3.00244

R = Mes*

P	1.94434	1.98299	0.11734
C	1.05093	0.60619	-0.16265
P	-0.68441	0.43096	-0.23469
C	3.66125	1.33166	-0.04149
C	4.44645	1.15658	1.12855
C	4.14398	0.85469	-1.28103
C	5.52129	0.28345	1.05970
C	5.21237	-0.04476	-1.26195
C	5.87708	-0.39642	-0.10072
H	6.10048	0.10354	1.95327
H	5.54696	-0.46227	-2.20046
C	3.68602	1.36176	-2.66710
C	4.20704	1.94434	2.42979
C	7.00099	-1.42619	-0.06685
C	2.89237	1.56740	3.11387
H	2.01968	1.86349	2.52631
H	2.82449	0.49227	3.28498
H	2.81521	2.08690	4.07366
C	4.21418	3.44577	2.10922
H	5.16123	3.74084	1.65075
H	3.40731	3.73000	1.43043
H	4.08655	4.02252	3.02950
C	5.31881	1.70432	3.45392
H	5.33494	0.67157	3.80974
H	6.30581	1.95518	3.05819
H	5.13883	2.34404	4.32081
C	2.75861	0.37279	-3.37662
H	1.79799	0.28435	-2.86760
H	2.56131	0.71165	-4.39797
H	3.21099	-0.62009	-3.43640
C	3.01115	2.73027	-2.59357
H	2.03648	2.70571	-2.09660
H	3.63902	3.46047	-2.07749
H	2.82168	3.09910	-3.60526
C	4.92536	1.56576	-3.55728
H	5.67345	2.18942	-3.06262
H	5.39967	0.62662	-3.84379
H	4.62247	2.06436	-4.48133
C	7.22362	-2.08413	-1.42593
H	7.54258	-1.36306	-2.18314
H	8.01004	-2.83766	-1.34090
H	6.32211	-2.58712	-1.78508

C	6.64337	-2.52142	0.94530
H	6.51789	-2.11792	1.95168
H	5.71027	-3.02006	0.67376
H	7.43768	-3.27216	0.98081
C	8.30764	-0.74705	0.36026
H	8.22477	-0.29558	1.35091
H	9.11788	-1.48056	0.39551
H	8.59009	0.03851	-0.34518
C	-2.10758	1.43852	0.29756
C	-2.75530	1.02765	1.49650
C	-2.85713	2.18338	-0.66075
C	-4.14521	0.98768	1.48917
C	-4.23667	2.10511	-0.58525
C	-4.90963	1.40738	0.41321
H	-4.64386	0.60704	2.36944
H	-4.81827	2.62280	-1.33775
C	-2.08637	0.82286	2.87033
C	-2.43706	-0.51324	3.52871
C	-2.65341	1.95888	3.75011
H	-2.01588	-1.35421	2.97938
H	-2.02870	-0.53322	4.54256
H	-3.51513	-0.66413	3.60485
H	-2.43278	2.93806	3.31779
H	-3.73327	1.88190	3.87959
H	-2.18976	1.91018	4.73899
C	-2.29521	3.24099	-1.63351
C	-2.86718	4.58494	-1.13420
C	-2.75240	3.03507	-3.07978
H	-2.57159	4.77561	-0.09974
H	-2.47737	5.39605	-1.75539
H	-3.95590	4.61446	-1.18458
H	-2.35865	2.11146	-3.50241
H	-3.83995	3.00614	-3.16432
H	-2.39837	3.86721	-3.69423
C	-6.41778	1.22087	0.34131
C	-7.10672	2.58957	0.40284
C	-6.94353	0.35482	1.48293
H	-6.80503	3.23457	-0.42537
H	-8.19102	2.46201	0.34618
H	-6.87386	3.10741	1.33692
H	-6.46938	-0.62947	1.48888
H	-6.79466	0.82474	2.45893
H	-8.01797	0.20379	1.35648
Cl	2.49112	-1.92982	1.83995
Cl	-1.02926	0.15617	-2.23898
Au	-2.85606	-2.30467	-0.26097
Cl	-4.86191	-3.12452	-0.91635
C	-0.78083	3.40394	-1.59567
H	-0.41678	3.53900	-0.57462
H	-0.25047	2.57206	-2.05448
H	-0.50663	4.30023	-2.15787
C	-0.57487	1.01368	2.88054
H	-0.02595	0.19139	2.42351
H	-0.29176	1.94992	2.39225
H	-0.23504	1.07140	3.91751
C	-6.76408	0.52905	-0.98365
H	-6.44759	1.12175	-1.84480
H	-6.28847	-0.45218	-1.05227
H	-7.84640	0.39122	-1.05461
Sn	1.73187	-1.58176	-0.42918
S	-0.87936	-1.47260	0.50658

Coordination through P(sp^2) atom

R= H

P	0.59381	-0.01685	0.91664
C	0.34979	-0.34024	-0.66914
P	-1.12799	-0.28357	-1.57513
Cl	1.14536	-2.79782	-2.86026
Sn	1.75828	-0.49683	-2.50527
Au	-0.70667	0.54308	2.59522
Cl	-2.02028	1.11462	4.33555
Cl	-2.68630	0.63378	-0.68835
H	-1.61963	-1.59470	-1.67692
H	1.98811	-0.14867	1.10242
S	-0.62930	0.49814	-3.32481

R = Me

P	0.67670	0.08945	0.89451
C	0.35579	-0.23323	-0.67708
P	-1.19756	-0.26362	-1.45867
Cl	1.34677	-2.86718	-2.62477
Sn	1.65376	-0.46628	-2.57272
C	2.45039	-0.04392	1.22374
H	2.60646	-0.85503	1.93967
H	3.01992	-0.24110	0.31589
H	2.79386	0.88096	1.69234
C	-1.97259	-1.86773	-1.25355
H	-2.18910	-2.03394	-0.19694
H	-2.89372	-1.88992	-1.83756
H	-1.27046	-2.61748	-1.62582
Au	-0.66883	0.57942	2.56957
Cl	-2.02643	1.04571	4.31453
Cl	-2.55204	1.01624	-0.66124
S	-0.81329	0.18852	-3.36127

R = *t*-Bu

P	0.71399	0.19567	0.67452
C	0.50456	-0.33114	-0.86806
P	-1.05030	-0.48030	-1.66250
Cl	2.22251	-2.90264	-2.58075
Sn	1.87187	-0.51340	-2.73860
Au	-0.78868	0.93964	2.12312
Cl	-2.29829	1.69665	3.63271
C	2.45932	0.12592	1.29031
C	2.80978	1.52574	1.80119
C	2.43707	-0.86181	2.46336
C	3.45717	-0.32904	0.23952
H	2.83981	2.25800	0.99092
H	2.09632	1.87440	2.55152
H	3.79926	1.49484	2.26684
H	2.21467	-1.87861	2.13160
H	3.42475	-0.86757	2.93422
H	1.70569	-0.57616	3.22323
H	4.44736	-0.40763	0.69877
H	3.20854	-1.31096	-0.17055
H	3.54334	0.39415	-0.57606
C	-2.02929	-1.97418	-1.15992
C	-1.17486	-3.20522	-1.45695
C	-2.37358	-1.91394	0.32383
C	-3.30107	-1.99959	-2.00679
H	-0.91783	-3.28076	-2.51395
H	-0.24595	-3.21249	-0.88523
H	-1.75257	-4.09174	-1.18061
H	-2.96541	-1.03494	0.58242
H	-2.96468	-2.80028	0.57102
H	-1.48186	-1.93097	0.95279
H	-3.84479	-2.91888	-1.77306

H	-3.95669	-1.15551	-1.78848
H	-3.07630	-2.00538	-3.07506
Cl	-2.27590	1.08169	-1.20736
S	-0.64613	-0.46486	-3.61848

R = Ph

P	0.67022	0.31288	0.52966
C	0.47944	-0.41105	-0.93036
P	-1.00228	-0.63546	-1.81322
Cl	1.97098	-3.34386	-2.10085
Sn	1.97771	-0.96599	-2.58022
Au	-0.78713	1.38888	1.79421
Cl	-2.28773	2.45621	3.10871
C	2.35926	0.15286	1.08805
C	2.87478	1.14603	1.92347
C	3.15537	-0.93908	0.72951
C	4.18607	1.06911	2.36036
H	2.24637	1.97804	2.22397
C	4.46248	-1.01232	1.17965
H	2.74031	-1.75256	0.14286
C	4.97978	-0.00647	1.98568
H	4.58739	1.84645	2.99965
H	5.07464	-1.86470	0.90959
H	6.00362	-0.06921	2.33614
C	-1.93767	-2.02894	-1.16620
C	-2.71892	-1.88198	-0.02015
C	-1.81316	-3.27076	-1.78136
C	-3.37907	-2.98069	0.50019
H	-2.82060	-0.91561	0.46028
C	-2.47947	-4.36473	-1.25114
H	-1.19474	-3.37877	-2.66382
C	-3.26019	-4.22061	-0.11499
H	-3.99100	-2.86666	1.38706
H	-2.38395	-5.33203	-1.72976
H	-3.78106	-5.07807	0.29557
Cl	-2.26792	0.93951	-1.61063
S	-0.45100	-0.85587	-3.71333

R = Mes

P	0.62123	0.44562	1.09944
C	0.26730	-0.37507	-0.27488
P	-1.15431	-0.46735	-1.28030
Cl	1.46354	-3.17165	-1.96139
Sn	1.89036	-0.78715	-1.84984
Au	-0.64524	1.60402	2.49358
Cl	-1.90782	2.78476	3.95578
C	2.39633	0.31282	1.39392
C	3.20199	1.44609	1.19156
C	2.94930	-0.89457	1.85122
C	4.56412	1.33976	1.43154
C	4.31869	-0.94427	2.07954
C	5.14195	0.15614	1.87630
H	5.19280	2.20919	1.26519
H	4.75365	-1.87418	2.43264
C	-2.01382	-2.05639	-1.13772
C	-2.35397	-2.50571	0.15879
C	-2.35132	-2.84411	-2.26261
C	-2.96489	-3.74351	0.30244
C	-2.96148	-4.07284	-2.04334
C	-3.26206	-4.55493	-0.77962
H	-3.22484	-4.07377	1.30323
H	-3.21932	-4.67297	-2.90989
Cl	-2.58282	0.92027	-0.81554
C	2.62490	2.74374	0.71196
H	2.13552	2.63440	-0.26069
H	1.87267	3.12886	1.40762
H	3.40342	3.49985	0.60928
C	2.11194	-2.11254	2.10667
H	1.24128	-1.87502	2.72560
H	1.74103	-2.55493	1.17767
H	2.69320	-2.87237	2.62957
C	6.60987	0.08019	2.16023
H	6.82197	0.41923	3.17903
H	6.98107	-0.94176	2.07047
H	7.18006	0.71583	1.48053
C	-2.10899	-1.73681	1.42184
H	-2.77587	-2.09660	2.20572
H	-1.08433	-1.87729	1.77347
H	-2.27939	-0.66577	1.31968
C	-2.12654	-2.47313	-3.69861
H	-2.50687	-1.48154	-3.94251
H	-1.06552	-2.48541	-3.95229
H	-2.63590	-3.19603	-4.33644
C	-3.88281	-5.90269	-0.59293
H	-4.52829	-6.16425	-1.43307
H	-3.10742	-6.67216	-0.52460
H	-4.47141	-5.94774	0.32469
S	-0.36018	0.04660	-3.04315

R = Mes*

P	-1.00028	0.88860	0.13338
C	-0.34166	-0.60568	0.32149
P	1.29256	-1.23212	0.37080
Cl	-2.25143	-2.57919	-2.08331
C	2.93506	-0.48508	0.06989
C	3.52591	-0.77735	-1.19781
C	3.78481	-0.11468	1.14865
C	4.87799	-1.08058	-1.21400
C	5.13225	-0.44826	1.04109
C	5.68771	-1.02646	-0.08720
H	5.32295	-1.35457	-2.16201
H	5.77326	-0.22453	1.88177
C	-2.80503	0.65709	0.18757
C	-3.57688	0.66087	-1.00401
C	-3.38011	0.20413	1.40295
C	-4.79103	-0.01758	-0.98070
C	-4.58129	-0.49136	1.32512
C	-5.27830	-0.67051	0.14055
H	-5.36567	-0.06617	-1.89032
H	-4.99746	-0.89748	2.23773
C	-3.20765	1.44217	-2.27915
C	-2.86591	0.50706	2.82758
C	-6.54646	-1.51669	0.11806
C	3.44540	0.80539	2.34285
C	2.87956	-0.58808	-2.58753
C	7.13563	-1.49358	-0.15627
C	-4.05274	1.01580	3.66764
H	-3.68933	1.31975	4.65236
H	-4.52544	1.88142	3.19785
H	-4.81879	0.25738	3.82806
C	-1.82387	1.62165	2.89080
H	-0.84471	1.32020	2.51558
H	-2.14667	2.51593	2.35215
H	-1.66559	1.90461	3.93492
C	-2.30122	-0.74960	3.49451
H	-3.03046	-1.56293	3.49428
H	-1.40079	-1.09931	2.98643
H	-2.03708	-0.53531	4.53410
C	-7.58525	-0.91908	1.07416
H	-8.49572	-1.52448	1.06391
H	-7.22124	-0.88518	2.10307
H	-7.85061	0.09874	0.77781

C	-7.16457	-1.59743	-1.27517
H	-8.05045	-2.23603	-1.24237
H	-7.48127	-0.61668	-1.63920
H	-6.47102	-2.02910	-2.00071
C	-6.19529	-2.93966	0.57005
H	-5.46001	-3.39064	-0.10074
H	-5.78286	-2.95330	1.58128
H	-7.09115	-3.56662	0.56631
C	-4.26566	1.28635	-3.37572
H	-5.24971	1.63868	-3.05842
H	-3.96487	1.89322	-4.23235
H	-4.35269	0.25276	-3.71906
C	-3.15654	2.93405	-1.92175
H	-4.12129	3.27221	-1.53520
H	-2.39766	3.15481	-1.16777
H	-2.91740	3.52439	-2.81015
C	-1.88464	0.99964	-2.90612
H	-1.01748	1.23323	-2.28620
H	-1.87981	-0.07175	-3.10882
H	-1.73380	1.53864	-3.84571
C	2.00647	1.29977	2.40734
H	1.68444	1.69479	1.44081
H	1.30067	0.54366	2.74001
H	1.94957	2.12757	3.11806
C	4.29401	2.07656	2.11422
H	4.09566	2.78773	2.92032
H	5.36382	1.86682	2.10478
H	4.03052	2.55841	1.16993
C	3.83598	0.19981	3.69410
H	4.89803	-0.04636	3.74484
H	3.63206	0.92540	4.48591
H	3.26867	-0.70517	3.91062
C	1.43700	-0.09960	-2.57385
H	1.16542	0.21288	-3.58506
H	0.72173	-0.86584	-2.28082
H	1.32291	0.77493	-1.92725
C	2.97928	-1.83823	-3.46483
H	2.39534	-2.66015	-3.05107
H	2.58973	-1.61254	-4.46097
H	4.01040	-2.17628	-3.58494
C	3.68832	0.54696	-3.25292
H	3.63855	1.46323	-2.65981
H	4.73860	0.28764	-3.38973
H	3.26282	0.75774	-4.23753
C	7.84716	-1.37308	1.18866
H	7.92893	-0.33378	1.51690
H	7.33694	-1.94298	1.96920
H	8.86217	-1.76717	1.09969
C	7.90082	-0.65354	-1.18600
H	7.47325	-0.74674	-2.18617
H	7.89215	0.40457	-0.91358
H	8.94199	-0.98338	-1.23770
C	7.15695	-2.96606	-0.58422
H	6.62755	-3.59159	0.13841
H	6.68692	-3.11077	-1.55893
H	8.18861	-3.32158	-0.65270
Sn	-1.41172	-2.68182	0.19175
Cl	1.42440	-1.88252	2.33001
Au	0.05845	2.82386	-0.19869
Cl	1.18131	4.77063	-0.52557
S	1.12760	-2.95358	-0.63341

Coordination through π(C=P) bond

R= H

P	0.62401	0.05020	1.09973
C	0.46791	0.01424	-0.61979
P	-1.01128	-0.22261	-1.51751
Cl	1.35983	-2.73231	-2.41030
Sn	1.84102	-0.36422	-2.47846
Au	0.77309	2.08405	-0.12569
Cl	1.10571	4.30391	-0.20219
Cl	-2.68857	0.59476	-0.73606
H	2.03874	-0.14338	1.16367
H	-1.32860	-1.58731	-1.42915
S	-0.62152	0.36211	-3.36729

R = Me

P	0.58559	0.12859	1.04171
C	0.40415	0.09801	-0.67203
P	-1.09927	-0.17934	-1.52653
Cl	1.68782	-2.66959	-2.30531
Sn	1.79377	-0.24993	-2.51662
C	2.35887	-0.25382	1.31113
H	2.42366	-1.32609	1.52438
H	2.99702	-0.02396	0.45767
H	2.71196	0.28540	2.19235
C	-1.67319	-1.85170	-1.21899
H	-1.92733	-1.96132	-0.16344
H	-2.55101	-2.03639	-1.83940
H	-0.86634	-2.53446	-1.49587
Au	0.71098	2.17393	-0.18644
Cl	1.06242	4.39592	-0.19583
Cl	-2.66339	0.95439	-0.88367
S	-0.68542	0.16775	-3.44253

R = *t*-Bu

P	1.05103	0.38920	1.16301
C	0.64255	0.10069	-0.49036
P	-1.01626	-0.21940	-1.00987
Cl	2.16078	-2.58166	-2.36507
Sn	1.69214	-0.20687	-2.56920
Au	1.01795	2.23934	-0.34656
Cl	1.42175	4.43340	-0.65650
C	2.86962	0.04345	1.42755
C	3.75812	0.04043	0.19755
C	3.34240	1.09661	2.43138
C	2.87676	-1.34223	2.08611
H	3.49853	-0.77317	-0.48493
H	3.72354	0.99445	-0.33491
H	4.79766	-0.12565	0.49984
H	2.69690	1.13713	3.31327
H	4.35343	0.84954	2.76915
H	3.36935	2.09374	1.98541
H	3.89507	-1.57851	2.41074
H	2.23312	-1.38112	2.96979
H	2.55961	-2.12096	1.38794
C	-1.76619	-1.75162	-0.27334
C	-0.83282	-2.92368	-0.56634
C	-1.95820	-1.58862	1.23139
C	-3.11440	-1.97716	-0.95770
H	-0.66615	-3.06031	-1.63502
H	0.14153	-2.80835	-0.09002
H	-1.29700	-3.83294	-0.17385
H	-2.64627	-0.77866	1.47468
H	-2.37982	-2.51957	1.62091
H	-1.01662	-1.40886	1.75381
H	-3.54467	-2.90112	-0.56175

H	-3.81688	-1.16618	-0.75914
H	-3.00497	-2.09148	-2.03734
Cl	-2.31071	1.24706	-0.40842
S	-0.95013	-0.27852	-3.00273

R = Ph

P	0.52468	0.33572	0.96693
C	0.30236	0.20980	-0.73874
P	-1.22865	-0.04922	-1.54817
Cl	1.67097	-2.35885	-2.82472
Sn	1.64637	0.06559	-2.64839
Au	0.63862	2.32150	-0.36122
Cl	1.00198	4.54059	-0.49289
C	2.28079	-0.03244	1.22766
C	2.92988	0.62900	2.27305
C	2.96727	-1.00492	0.49824
C	4.25859	0.35785	2.55054
H	2.39595	1.37087	2.85861
C	4.29299	-1.28415	0.79213
H	2.46011	-1.57025	-0.27699
C	4.94124	-0.59774	1.80914
H	4.76247	0.88956	3.34925
H	4.81804	-2.04405	0.22493
H	5.97942	-0.81507	2.03262
C	-1.79232	-1.73847	-1.29329
C	-1.85377	-2.62229	-2.36355
C	-2.14926	-2.15585	-0.01117
C	-2.27785	-3.92543	-2.15060
H	-1.56207	-2.29192	-3.35253
C	-2.56707	-3.45810	0.19240
H	-2.10947	-1.46482	0.82391
C	-2.63212	-4.34238	-0.87788
H	-2.32389	-4.61585	-2.98423
H	-2.84554	-3.78448	1.18743
H	-2.96055	-5.36248	-0.71473
Cl	-2.74673	1.06289	-0.76109
S	-0.90479	0.41835	-3.45300

R = Mes

P	0.50200	0.36222	1.39401
C	0.15317	0.19637	-0.29419
P	-1.30029	-0.01765	-1.25737
Cl	1.65696	-2.02000	-2.76453
Sn	1.69079	0.31241	-2.06105
Au	0.28913	2.34014	0.07289
Cl	0.38778	4.59783	-0.02264
C	2.30462	0.05665	1.46548
C	3.18259	1.03894	1.95435
C	2.78742	-1.23628	1.17218
C	4.54053	0.74865	2.02255
C	4.15264	-1.47159	1.25763
C	5.05097	-0.48712	1.65321
H	5.21730	1.51588	2.38693
H	4.52229	-2.46640	1.02801
C	-1.83113	-1.75185	-1.32546
C	-1.99713	-2.45560	-2.54358
C	-2.08963	-2.40862	-0.10134
C	-2.33425	-3.80100	-2.48207
C	-2.42538	-3.75675	-0.11655
C	-2.53101	-4.48146	-1.28996
H	-2.45777	-4.33550	-3.41832
H	-2.62376	-4.24623	0.83212
Cl	-2.96873	0.97253	-0.56580
C	2.71494	2.38724	2.41333
H	2.61651	3.09337	1.58344
H	1.74021	2.34030	2.90626
H	3.42705	2.81218	3.12251
C	1.87584	-2.39047	0.86881
H	1.14486	-2.52759	1.67236
H	1.32600	-2.27185	-0.06575
H	2.45128	-3.31306	0.79036
C	6.52226	-0.76007	1.69157
H	6.72902	-1.79299	1.97800
H	6.96825	-0.60107	0.70474
H	7.03295	-0.09817	2.39272
C	-2.05037	-1.76737	1.25458
H	-1.08308	-1.92979	1.73758
H	-2.23960	-0.69736	1.24986
H	-2.80621	-2.22784	1.89305
C	-1.86909	-1.87630	-3.92120
H	-2.47226	-0.97852	-4.05388
H	-0.83575	-1.61465	-4.15084
H	-2.20045	-2.61712	-4.64944
C	-2.85322	-5.94191	-1.27975
H	-3.52327	-6.20686	-2.09990
H	-1.94108	-6.53412	-1.40205
H	-3.31936	-6.24240	-0.34041
S	-0.76761	0.91291	-2.94687

R = Mes*

P	0.66143	0.18855	1.11861
C	0.40591	0.08804	-0.60119
P	-1.09520	-0.09580	-1.57733
Cl	2.90877	-1.40482	-3.30886
Au	0.45175	2.24297	-0.14653
Cl	0.37464	4.50963	-0.23726
C	2.50311	0.05104	1.23777
C	3.16932	-1.11145	0.75615
C	3.28844	1.16191	1.63273
C	4.48249	-0.98209	0.32989
C	4.60564	1.21839	1.17021
C	5.19986	0.20517	0.44004
H	4.97581	-1.85236	-0.08238
H	5.18588	2.09822	1.40902
C	2.92116	2.23722	2.67736
C	2.65929	-2.55299	0.94983
C	6.59726	0.31708	-0.15800
C	3.85706	1.97231	3.87486
H	3.69752	0.97103	4.28341
H	4.90888	2.05916	3.59986
H	3.65246	2.70040	4.66452
C	3.17257	3.65894	2.16709
H	2.54052	3.90150	1.31222
H	2.94427	4.37497	2.96130
H	4.21308	3.81609	1.87861
C	1.50449	2.16969	3.23944
H	1.43413	2.85517	4.08831
H	0.74372	2.48637	2.52386
H	1.25215	1.17153	3.60592
C	2.98426	-3.46471	-0.23570
H	2.58796	-3.06908	-1.16968
H	4.05809	-3.60749	-0.36496
H	2.55138	-4.45358	-0.06184
C	3.41496	-3.07571	2.18741
H	4.49483	-3.05263	2.03179
H	3.18870	-2.47301	3.07077
H	3.11960	-4.10913	2.39203
C	1.17481	-2.69195	1.26290
H	0.87294	-2.08687	2.12541
H	0.54354	-2.44036	0.41263
H	0.96463	-3.73185	1.52854
C	7.23976	1.67271	0.12256

H	6.64253	2.49539	-0.27822
H	7.38717	1.84207	1.19254
H	8.22182	1.71518	-0.35435
C	6.49220	0.13524	-1.67715
H	7.48630	0.17989	-2.13091
H	6.03916	-0.82170	-1.94296
H	5.88051	0.92344	-2.12315
C	7.50515	-0.77345	0.42266
H	7.58409	-0.68127	1.50889
H	7.13798	-1.77578	0.19423
H	8.50982	-0.68644	0.00007
C	-2.33647	-1.47602	-1.65964
C	-2.31372	-2.31380	-2.81639
C	-3.56765	-1.35349	-0.95825
C	-3.52220	-2.56862	-3.44521
C	-1.12055	-3.16179	-3.29610
C	-4.73736	-1.67671	-1.63892
C	-3.74118	-1.09764	0.55092
C	-4.75022	-2.17242	-2.93214
H	-3.49792	-3.13784	-4.36620
C	-0.67046	-2.86163	-4.72705
C	-1.60091	-4.62921	-3.25629
C	0.05219	-3.10220	-2.33280
H	-5.67591	-1.53089	-1.12307
C	-2.45091	-0.79007	1.29138
C	-4.24998	-2.43947	1.11925
C	-4.77289	-0.01459	0.87670
C	-6.02800	-2.38509	-3.73205
H	0.04533	-3.62278	-5.04799
H	-1.51405	-2.88210	-5.42123
H	-0.18539	-1.89149	-4.80399
H	-2.35754	-4.84577	-4.01112
H	-0.74587	-5.28281	-3.44849
H	-2.01292	-4.88584	-2.27735
H	0.49809	-2.11347	-2.25427
H	-0.25307	-3.44098	-1.34022
H	0.84750	-3.76116	-2.68564
H	-1.67298	-1.52063	1.05884
H	-2.07600	0.21076	1.08738
H	-2.62873	-0.83950	2.36842
H	-4.36949	-2.35032	2.20255
H	-5.21210	-2.72743	0.69395
H	-3.53790	-3.24401	0.91954
H	-5.75612	-0.23825	0.45986
H	-4.88868	0.05671	1.96140
H	-4.46091	0.96169	0.50685
C	-7.26224	-1.86958	-2.99691
C	-6.22130	-3.87987	-4.01451
C	-5.90410	-1.62885	-5.06045
H	-7.44564	-2.41895	-2.06991
H	-7.17608	-0.80685	-2.75747
H	-8.14306	-1.99691	-3.63048
H	-5.39417	-4.29521	-4.59342
H	-6.30038	-4.44798	-3.08437
H	-7.13932	-4.03598	-4.58753
H	-5.77674	-0.55718	-4.89057
H	-5.05112	-1.97792	-5.64573
H	-6.80628	-1.77489	-5.66061
Sn	1.97249	0.62369	-2.30798
Cl	-2.41017	1.45207	-1.17589
S	-0.43424	0.33185	-3.41829

[R-P=C(Sn(II)Cl)-P(S)MeR](AuCl)

Coordination through Sn(II) atom

R= H

P	0.70147	0.42588	0.92033
C	0.47567	0.01864	-0.67262
P	-1.09851	-0.09488	-1.46136
Cl	1.62089	-2.37247	-2.85110
Sn	1.72065	-0.04128	-2.49715
C	-2.45270	0.78026	-0.66791
H	-3.36789	0.62501	-1.24189
H	-2.58722	0.39806	0.34667
H	-2.21706	1.84443	-0.63563
Au	3.66871	1.41962	-3.09088
Cl	5.42494	2.78467	-3.54438
H	2.12918	0.47845	0.91646
H	-1.49529	-1.44709	-1.44295
S	-0.69990	0.52727	-3.32842

R = Me

P	0.45410	0.60323	0.29595
C	0.14044	0.33319	-1.30886
P	-1.47626	0.02823	-1.94769
Cl	1.57006	-1.66999	-3.75170
Sn	1.18277	0.61112	-3.23028
S	-1.32650	0.73461	-3.83830
C	2.25316	0.92572	0.38765
H	2.74099	0.02598	0.77610
H	2.69943	1.19090	-0.57190
H	2.43316	1.72732	1.10763
C	-1.83022	-1.73673	-1.92943
H	-1.89535	-2.08862	-0.89752
H	-2.77107	-1.92452	-2.45011
H	-1.01859	-2.25187	-2.44575
C	-2.80392	0.83420	-1.03612
H	-3.75875	0.61860	-1.51920
H	-2.82118	0.46448	-0.00849
H	-2.63184	1.91040	-1.03590
Au	2.79738	2.42712	-3.85504
Cl	4.26766	4.10210	-4.30482

R = *t*-Bu

P	0.65225	-0.41375	0.81051
C	0.64018	-0.12469	-0.82441
P	-0.90739	-0.10636	-1.70453
Cl	2.78357	-1.39772	-3.33912
Sn	1.95142	0.62927	-2.43878
Au	3.31105	2.74343	-2.43981
Cl	4.50820	4.67723	-2.32583
C	2.39699	-0.37400	1.47065
C	2.56383	-1.72019	2.18537
C	3.49755	-0.18175	0.44583
C	2.41527	0.75842	2.50287
H	1.76759	-1.89871	2.91346
H	2.57505	-2.55383	1.47872
H	3.51595	-1.72529	2.72515
H	3.45912	0.80969	-0.01378
H	4.47352	-0.25745	0.93652
H	3.47028	-0.94504	-0.33670
H	3.36923	0.74308	3.03932
H	2.31406	1.73768	2.02925
H	1.61711	0.64992	3.24272
C	-1.66430	-1.78413	-1.92609
C	-2.82614	-1.65323	-2.91126
C	-0.60390	-2.72305	-2.49507

C	-2.16620	-2.30353	-0.58042
H	-3.62612	-1.01382	-2.53123
H	-2.49510	-1.26441	-3.87601
H	-3.25282	-2.64617	-3.07809
H	0.23438	-2.85582	-1.80957
H	-1.06127	-3.70131	-2.66751
H	-0.20693	-2.36106	-3.44467
H	-2.54400	-3.32023	-0.72038
H	-1.37181	-2.34718	0.16818
H	-2.98524	-1.70144	-0.18195
C	-2.12068	0.94010	-0.87005
H	-3.04342	0.98611	-1.44958
H	-2.32376	0.55629	0.13086
H	-1.69688	1.94172	-0.79472
S	-0.39620	0.70662	-3.49115

R = Ph

P	0.69305	0.16473	1.08386
C	0.37887	-0.23995	-0.49701
P	-1.24158	-0.28530	-1.19168
Cl	1.69136	-2.62623	-2.71661
Sn	1.55204	-0.28668	-2.35412
Au	3.38058	1.26559	-3.09714
Cl	5.04647	2.69681	-3.69687
C	2.48658	0.05763	1.24014
C	3.10069	0.95581	2.11642
C	3.27113	-0.87599	0.55637
C	4.47811	0.95930	2.26165
H	2.49622	1.66706	2.67069
C	4.64598	-0.87843	0.71614
H	2.80305	-1.62731	-0.07249
C	5.25029	0.04567	1.55864
H	4.95033	1.67533	2.92393
H	5.24834	-1.60318	0.18131
H	6.32801	0.04740	1.67292
C	-2.00513	-1.90242	-0.94347
C	-2.28554	-2.73222	-2.02211
C	-2.30417	-2.31692	0.35446
C	-2.87740	-3.96769	-1.80391
H	-2.03063	-2.41253	-3.02563
C	-2.89444	-3.55042	0.56495
H	-2.06310	-1.68786	1.20553
C	-3.18318	-4.37502	-0.51521
H	-3.09238	-4.61487	-2.64590
H	-3.12425	-3.87264	1.57375
H	-3.64194	-5.34266	-0.34758
C	-2.36913	0.91946	-0.46421
H	-3.33100	0.86068	-0.97595
H	-2.50826	0.70778	0.59766
H	-1.94348	1.91552	-0.58765
S	-0.89584	0.11262	-3.14086

R = Mes

P	0.77635	0.04693	1.52346
C	0.50252	-0.57054	0.00099
P	-1.07366	-0.55135	-0.81922
Cl	1.81368	-2.90783	-2.21147
Sn	1.85890	-0.58303	-1.71322
Au	3.93220	0.76847	-2.15450
Cl	5.83941	2.00320	-2.40703
C	2.59097	0.18370	1.57382
C	3.14697	1.47152	1.48317
C	3.41983	-0.93959	1.70241
C	4.52595	1.60465	1.46123
C	4.79755	-0.75347	1.68380
C	5.37109	0.50293	1.54041
H	4.95545	2.59529	1.34969
H	5.44237	-1.62229	1.77732
C	-2.02646	-2.09598	-0.94333
C	-3.10822	-2.11946	-1.85079
C	-1.75652	-3.22982	-0.14995
C	-3.89059	-3.26490	-1.93084
C	-2.57151	-4.34645	-0.28455
C	-3.64086	-4.39229	-1.16571
H	-4.72282	-3.27069	-2.62795
H	-2.35623	-5.21434	0.33058
C	-2.16679	0.62558	0.01439
H	-3.15672	0.63989	-0.43806
H	-2.25977	0.31394	1.05784
H	-1.71744	1.61779	-0.02989
C	-0.63552	-3.32422	0.83948
H	0.33148	-3.16481	0.36114
H	-0.72964	-2.59151	1.64338
H	-0.62820	-4.31620	1.29152
C	-3.46684	-1.00678	-2.79472
H	-3.34082	-0.00553	-2.38511
H	-2.84444	-1.05241	-3.69190
H	-4.50839	-1.10808	-3.10222
C	-4.47664	-5.62551	-1.30391
H	-4.56688	-6.15411	-0.35335
H	-5.47856	-5.39040	-1.66617
H	-4.02172	-6.31708	-2.01979
C	2.86852	-2.32743	1.85456
H	1.95686	-2.34210	2.45800
H	2.62390	-2.77385	0.88567
H	3.59985	-2.97821	2.33609
C	2.28189	2.68907	1.34207
H	1.41648	2.65834	2.01232
H	2.84754	3.59422	1.56559
H	1.90110	2.78544	0.32013
C	6.85249	0.67726	1.43620
H	7.11979	0.94880	0.41009
H	7.20525	1.48021	2.08766
H	7.38400	-0.23875	1.69724
S	-0.44548	0.12023	-2.63956

R = Mes*

P	-0.04215	1.99054	1.13930
C	0.34632	0.55679	0.38481
P	1.90813	-0.29273	0.23956
Cl	-1.26433	0.55788	-2.85119
C	3.63342	0.34685	0.21195
C	4.26994	0.50118	-1.05422
C	4.45813	0.15984	1.35472
C	5.57625	0.04967	-1.17491
C	5.76302	-0.27899	1.14979
C	6.31614	-0.44662	-0.10967
H	6.04169	0.10664	-2.15097
H	6.37279	-0.47946	2.01992
C	-1.86885	2.03723	0.94097
C	-2.44320	2.89005	-0.03055
C	-2.69474	1.13844	1.66597
C	-3.74236	2.61383	-0.44835
C	-3.97663	0.91204	1.17993
C	-4.50165	1.58239	0.08240
H	-4.17401	3.22134	-1.22730
H	-4.60029	0.18824	1.68548
C	-1.74942	4.16322	-0.55369
C	-2.32177	0.51465	3.03145
C	-5.88347	1.21466	-0.44729
C	4.13052	0.59882	2.79869
C	3.73362	1.31524	-2.24904

C	7.69940	-1.04086	-0.34066
C	-3.58636	0.32131	3.88292
H	-3.28958	0.04467	4.89749
H	-4.18128	1.23575	3.93932
H	-4.22104	-0.48383	3.51005
C	-1.41972	1.45532	3.83350
H	-0.42466	1.58358	3.39029
H	-1.86591	2.44717	3.93735
H	-1.25585	1.04380	4.83312
C	-1.65020	-0.85553	2.91354
H	-2.27280	-1.55600	2.35211
H	-0.68312	-0.78642	2.41740
H	-1.49069	-1.27443	3.91176
C	-6.92866	1.39566	0.65994
H	-7.92066	1.13211	0.28315
H	-6.72347	0.75813	1.52199
H	-6.95995	2.43242	1.00525
C	-6.29390	2.07677	-1.63826
H	-7.27459	1.75520	-1.99659
H	-6.37224	3.13445	-1.37220
H	-5.59087	1.97937	-2.46921
C	-5.86609	-0.25138	-0.89661
H	-5.13692	-0.40661	-1.69536
H	-5.61135	-0.93134	-0.08156
H	-6.85088	-0.53922	-1.27492
C	-2.66635	4.97810	-1.46895
H	-3.59606	5.26534	-0.97280
H	-2.14812	5.89647	-1.75437
H	-2.91149	4.44148	-2.38839
C	-1.40915	5.05542	0.64917
H	-2.30877	5.29986	1.21913
H	-0.69926	4.57822	1.32860
H	-0.95839	5.99035	0.30414
C	-0.47718	3.88023	-1.35234
H	0.31727	3.45748	-0.73206
H	-0.66301	3.18549	-2.17198
H	-0.08690	4.81632	-1.76294
C	2.78003	1.28711	2.95968
H	2.66898	2.10937	2.25030
H	1.91892	0.63118	2.85072
H	2.71386	1.71304	3.96385
C	5.16932	1.68943	3.14035
H	4.95665	2.09045	4.13499
H	6.18940	1.30543	3.14500
H	5.12040	2.51244	2.42382
C	4.28548	-0.52725	3.82545
H	5.28947	-0.95498	3.80372
H	4.12045	-0.13116	4.83082
H	3.57955	-1.34280	3.66926
C	2.37399	1.95776	-2.01215
H	2.18024	2.68165	-2.80722
H	1.55105	1.24684	-2.03463
H	2.35625	2.50002	-1.06383
C	3.71010	0.52382	-3.55840
H	2.99510	-0.29653	-3.51408
H	3.41642	1.18658	-4.37655
H	4.69129	0.11266	-3.80494
C	4.72333	2.48954	-2.40979
H	4.78134	3.08266	-1.49363
H	5.73104	2.15700	-2.66111
H	4.37425	3.14133	-3.21491
C	8.33939	-1.53591	0.95350
H	8.52398	-0.72032	1.65748
H	7.72077	-2.28789	1.44979
H	9.30415	-1.99669	0.72952
C	8.62010	0.01607	-0.96249
H	8.24468	0.36739	-1.92551
H	8.72366	0.88246	-0.30467
H	9.61494	-0.40599	-1.12822
C	7.56902	-2.22910	-1.30133
H	6.92845	-3.00681	-0.87866
H	7.14162	-1.92891	-2.26000
H	8.55272	-2.66549	-1.49398
Au	-2.50876	-2.55562	-0.33077
Cl	-4.04006	-4.12382	0.31892
Sn	-0.84199	-0.76653	-0.92935
S	1.58945	-1.38827	-1.45899
C	1.90298	-1.60064	1.50240
H	1.91695	-1.23809	2.52485
H	0.99252	-2.17906	1.33818
H	2.76210	-2.24646	1.31691

Coordination through S atom

R= H

P	0.77450	0.12330	1.02189
C	0.56886	-0.13178	-0.60453
P	-0.89834	0.26313	-1.47287
Cl	0.71544	-2.60267	-2.87551
Sn	1.94787	-0.60742	-2.36739
C	-2.16664	1.24107	-0.65944
H	-2.99204	1.39412	-1.35769
H	-2.52426	0.70521	0.22293
H	-1.75006	2.20465	-0.36508
Au	-1.76181	0.81817	-4.68579
Cl	-3.42797	0.55280	-6.17844
H	2.14177	-0.28979	1.13009
H	-1.51513	-0.94637	-1.83133
S	-0.12067	1.15198	-3.13956

R = Me

P	0.40962	0.40797	0.47164
C	0.28981	0.34377	-1.18022
P	-1.26506	0.43831	-1.98335
Cl	1.47670	-1.77838	-3.62132
Sn	1.74622	0.51567	-2.93829
S	-0.74068	1.48309	-3.67436
C	2.20318	0.26240	0.83185
H	2.39498	-0.74754	1.20763
H	2.83793	0.44272	-0.03700
H	2.46679	0.95925	1.63101
C	-1.88384	-1.20358	-2.36498
H	-2.10556	-1.71658	-1.42584
H	-2.78429	-1.11914	-2.97615
H	-1.11389	-1.74934	-2.91211
C	-2.57685	1.29832	-1.09849
H	-3.46200	1.34366	-1.73640
H	-2.81370	0.76014	-0.17796
H	-2.24825	2.30913	-0.85776
Au	-2.17409	0.80922	-5.31216
Cl	-3.64838	0.19166	-6.90246

R = *t*-Bu

P	0.96941	-0.12090	0.71306
C	0.72903	-0.13629	-0.93177
P	-0.91088	0.13626	-1.52676
Cl	2.01880	-2.22253	-3.48604
C	-1.80142	1.40117	-0.59112
H	-2.72087	1.66579	-1.11413
H	-2.02993	1.04600	0.41500
H	-1.15939	2.27944	-0.52541
Au	-1.98723	0.37473	-4.99439

Cl	-3.47093	-0.11120	-6.62346
S	-0.48743	0.94333	-3.37964
C	-1.97850	-1.38703	-1.53772
C	-3.40005	-0.99858	-1.94452
C	-1.41016	-2.41963	-2.50349
C	-1.98754	-1.95755	-0.11812
H	-3.88410	-0.35802	-1.20490
H	-3.43422	-0.50745	-2.92054
H	-3.99131	-1.91452	-2.02508
H	-0.38651	-2.70069	-2.25393
H	-2.03460	-3.31593	-2.45193
H	-1.42301	-2.06347	-3.53491
H	-2.66192	-2.81831	-0.10973
H	-1.00062	-2.30330	0.19065
H	-2.35604	-1.24739	0.62551
C	2.79798	-0.37570	1.04297
C	3.34198	-1.60363	0.32408
C	3.55908	0.88627	0.64056
C	2.90945	-0.57405	2.55622
H	2.80098	-2.51020	0.60620
H	3.28357	-1.52183	-0.76310
H	4.39507	-1.74738	0.58854
H	3.15814	1.77774	1.13081
H	4.60934	0.79063	0.93631
H	3.53451	1.05784	-0.43844
H	3.95924	-0.70138	2.83706
H	2.51613	0.28461	3.10695
H	2.36477	-1.46197	2.88792
Sn	2.05027	0.08391	-2.79976

R = Ph

P	0.71796	0.44160	0.81322
C	0.54994	-0.14135	-0.73370
P	-0.93882	0.00746	-1.64603
Cl	1.62181	-3.03602	-2.36635
Sn	2.13275	-0.68339	-2.26928
C	-2.03777	1.34219	-1.13535
H	-2.89299	1.36511	-1.81335
H	-2.38423	1.18394	-0.11281
H	-1.49268	2.28438	-1.19560
Au	-1.85326	0.15483	-5.03594
Cl	-3.50873	-0.00445	-6.56112
C	2.41502	-0.02361	1.25217
C	3.00886	-1.22005	0.83598
C	3.14270	0.84883	2.06378
C	4.31673	-1.51239	1.19187
H	2.42767	-1.95026	0.27932
C	4.45336	0.55876	2.40746
H	2.68216	1.76686	2.41558
C	5.04214	-0.61937	1.96850
H	4.76511	-2.44552	0.87074
H	5.01616	1.25068	3.02341
H	6.06450	-0.84945	2.24543
C	-1.88814	-1.51999	-1.52467
C	-2.54737	-1.78323	-0.32295
C	-1.94757	-2.43735	-2.56638
C	-3.27623	-2.95002	-0.17761
H	-2.48621	-1.08819	0.50782
C	-2.68162	-3.60358	-2.41397
H	-1.42261	-2.24499	-3.49323
C	-3.34663	-3.85859	-1.22570
H	-3.78976	-3.15091	0.75523
H	-2.73057	-4.31257	-3.23146
H	-3.92047	-4.77100	-1.11137
S	-0.18263	0.38942	-3.50244

R = Mes

P	0.60530	0.12280	1.19018
C	0.38145	-0.23221	-0.41678
P	-1.08393	-0.06680	-1.38489
Cl	1.93294	-2.70243	-2.27697
Sn	2.17180	-0.34723	-1.78704
Au	-1.72619	1.51715	-4.49645
Cl	-3.27661	2.37342	-5.89562
C	2.41336	-0.13241	1.36963
C	2.93383	-1.42534	1.55443
C	3.26371	0.98641	1.40843
C	4.30602	-1.57103	1.72559
C	4.62621	0.78901	1.58372
C	5.16894	-0.48234	1.73395
H	4.70981	-2.56888	1.86863
H	5.28264	1.65393	1.60806
C	-1.99241	-1.64698	-1.50076
C	-2.65466	-2.08546	-0.33126
C	-2.04604	-2.44886	-2.66690
C	-3.35412	-3.28585	-0.35398
C	-2.77274	-3.63240	-2.62039
C	-3.43758	-4.07380	-1.48758
H	-3.84721	-3.61009	0.55733
H	-2.80819	-4.23882	-3.51951
C	2.72337	2.37570	1.24306
H	2.40120	2.56090	0.21318
H	1.85317	2.54952	1.88397
H	3.47945	3.12100	1.49218
C	6.64607	-0.67275	1.88939
H	7.12823	-0.76113	0.91081
H	7.10511	0.17351	2.40354
H	6.87413	-1.58140	2.44881
C	2.04894	-2.63621	1.58621
H	1.16788	-2.47240	2.21395
H	1.68968	-2.89800	0.58687
H	2.59012	-3.49615	1.98247
C	-2.69127	-1.34815	0.97802
H	-3.54875	-0.67026	1.02231
H	-2.81056	-2.06223	1.79381
H	-1.79303	-0.77092	1.19340
C	-1.38357	-2.14523	-3.97690
H	-1.78349	-1.24108	-4.43914
H	-0.30751	-2.01476	-3.87105
H	-1.55453	-2.97329	-4.66462
C	-4.21973	-5.34885	-1.49964
H	-4.45391	-5.68396	-0.48843
H	-5.16495	-5.21477	-2.03417
H	-3.67109	-6.14396	-2.00881
S	-0.16834	0.68722	-3.05030
C	-2.23934	1.22071	-0.85458
H	-1.69710	2.16633	-0.87842
H	-3.05707	1.26129	-1.57713
H	-2.63288	1.04427	0.14298

R = Mes*

P	-1.91086	1.98071	0.32669
C	-1.00269	0.58948	0.27742
P	0.74944	0.40607	0.36837
C	-3.62798	1.30050	0.34908
C	-4.41567	1.37061	-0.83025
C	-4.11438	0.58458	1.46700
C	-5.48970	0.50057	-0.94297
C	-5.19098	-0.28271	1.26783
C	-5.85163	-0.39505	0.05744
H	-6.06692	0.50543	-1.85573
H	-5.53303	-0.87672	2.10214

C	-3.63748	0.78430	2.92454
C	-4.19050	2.42083	-1.93498
C	-6.97761	-1.39447	-0.18482
C	-2.88045	2.21547	-2.69639
H	-2.00427	2.38835	-2.06791
H	-2.80450	1.20327	-3.09557
H	-2.82177	2.92953	-3.52332
C	-4.20583	3.81751	-1.29722
H	-5.15036	3.99762	-0.77783
H	-3.39397	3.95513	-0.57999
H	-4.09197	4.58052	-2.07236
C	-5.31073	2.39887	-2.97786
H	-5.32391	1.46748	-3.54872
H	-6.29566	2.55046	-2.52986
H	-5.14235	3.21218	-3.68732
C	-2.61543	-0.26867	3.35900
H	-1.68395	-0.16919	2.80199
H	-2.38740	-0.15345	4.42294
H	-3.00085	-1.27964	3.20633
C	-3.05791	2.18103	3.15191
H	-2.11237	2.34870	2.62683
H	-3.75727	2.95932	2.83801
H	-2.84599	2.32050	4.21548
C	-4.83974	0.67989	3.87877
H	-5.65880	1.33079	3.56491
H	-5.22416	-0.33721	3.96524
H	-4.52610	0.98554	4.87988
C	-7.21682	-2.30049	1.01998
H	-7.53997	-1.73649	1.89895
H	-8.00572	-3.01860	0.78427
H	-6.32128	-2.86894	1.28371
C	-6.61053	-2.27628	-1.38460
H	-6.46529	-1.68667	-2.29159
H	-5.68562	-2.82741	-1.20004
H	-7.40852	-2.99886	-1.57698
C	-8.27885	-0.64196	-0.48719
H	-8.18602	-0.01098	-1.37333
H	-9.09023	-1.35241	-0.66824
H	-8.56639	-0.00353	0.35200
C	2.15151	1.50093	-0.07980
C	2.77181	1.30109	-1.34724
C	2.92960	2.07219	0.96351
C	4.15743	1.27489	-1.38491
C	4.31372	2.00651	0.84946
C	4.95650	1.50424	-0.27199
H	4.63495	1.06424	-2.33355
H	4.91021	2.37650	1.67225
C	2.06187	1.31872	-2.71507
C	2.40920	0.11676	-3.59643
C	2.58152	2.59524	-3.41088
H	2.03891	-0.81367	-3.16814
H	1.94779	0.24483	-4.57915
H	3.48529	0.01707	-3.74977
H	2.35597	3.48508	-2.81768
H	3.65862	2.56582	-3.57927
H	2.09010	2.69943	-4.38197
C	2.39216	2.94542	2.11684
C	3.02336	4.33843	1.90559
C	2.81448	2.45026	3.50322
H	2.77729	4.73240	0.91695
H	2.63092	5.02998	2.65623
H	4.10894	4.32006	2.00142
H	2.38617	1.48028	3.75405
H	3.89917	2.35884	3.58224
H	2.48892	3.16589	4.26293
C	6.46243	1.29724	-0.34742
C	7.06312	2.26566	-1.37362
C	6.73908	-0.14736	-0.78343
H	6.87950	3.30532	-1.09026
H	8.14427	2.11627	-1.43809
H	6.64676	2.10905	-2.37087
H	6.31196	-0.86821	-0.08238
H	6.32079	-0.35882	-1.76958
H	7.81784	-0.31750	-0.83651
Cl	-2.36015	-1.45985	-2.28385
Au	2.78132	-2.41283	0.12108
Cl	4.64540	-3.42208	0.91687
C	0.88718	3.18729	2.07048
H	0.57745	3.57222	1.09682
H	0.28180	2.31011	2.28919
H	0.62240	3.93759	2.81971
C	0.54929	1.47674	-2.64209
H	0.02998	0.57919	-2.30897
H	0.27183	2.31191	-1.99429
H	0.16890	1.69761	-3.64234
C	7.14300	1.53221	0.99845
H	7.04713	2.56862	1.33363
H	6.73949	0.87560	1.77305
H	8.21022	1.31820	0.90713
Sn	-1.68112	-1.59762	0.03152
S	0.95369	-1.34835	-0.73174
C	1.07955	-0.16941	2.06140
H	2.14105	-0.39903	2.15766
H	0.76879	0.54673	2.81670
H	0.51487	-1.09380	2.19120

Coordination through P(sp²) atom

R= H

P	0.66385	0.00714	0.86972
C	0.41970	-0.30207	-0.71760
P	-1.07088	-0.12119	-1.62534
Cl	1.00017	-2.87711	-2.79038
Sn	1.82604	-0.62515	-2.52304
C	-2.37513	0.87993	-0.89998
H	-3.22093	0.89751	-1.59053
H	-2.68922	0.45276	0.05510
H	-2.01012	1.89597	-0.74740
Au	-0.66610	0.64530	2.50730
Cl	-2.06347	1.30233	4.15420
H	2.04348	-0.19790	1.09508
H	-1.62210	-1.41387	-1.71370
S	-0.44377	0.53983	-3.40494

R = Me

P	0.42905	0.51839	0.37757
C	0.16658	0.28785	-1.21833
P	-1.39866	0.08188	-1.99503
Cl	1.57291	-2.14879	-3.10767
Sn	1.49442	0.27637	-3.09933
S	-1.02196	0.51826	-3.92313
C	2.19543	0.65698	0.74455
H	2.48315	-0.17881	1.38717
H	2.79420	0.64298	-0.16607
H	2.37387	1.58064	1.29920
C	-1.93434	-1.62288	-1.78306
H	-2.13472	-1.81722	-0.72697
H	-2.83766	-1.78911	-2.37318
H	-1.13612	-2.27556	-2.14029
C	-2.71330	1.12467	-1.34020
H	-3.61168	0.96946	-1.94145
H	-2.91891	0.87006	-0.29794

H	-2.41012	2.16920	-1.41120
Au	-1.02705	0.63371	2.03829
Cl	-2.55742	0.73179	3.70220

R = *t*-Bu

P	0.71391	0.16228	0.70744
C	0.48545	-0.31807	-0.84500
P	-1.10258	-0.42462	-1.62312
Cl	2.22040	-2.86669	-2.56591
Sn	1.83378	-0.47781	-2.72778
C	-2.14917	1.00314	-1.26100
H	-3.03804	0.96408	-1.89182
H	-2.43537	1.03325	-0.20828
H	-1.58228	1.90149	-1.50638
Au	-0.80008	0.85759	2.18068
Cl	-2.37010	1.58982	3.64484
C	2.46604	0.12379	1.30931
C	2.79171	1.52428	1.83356
C	2.48071	-0.87862	2.46919
C	3.45933	-0.29572	0.23964
H	2.79935	2.26582	1.03123
H	2.07782	1.84964	2.59413
H	3.78557	1.50965	2.29072
H	2.27647	-1.89551	2.12632
H	3.47355	-0.86910	2.92904
H	1.75192	-0.61964	3.24116
H	4.45703	-0.35907	0.68499
H	3.22524	-1.27685	-0.18060
H	3.51792	0.43839	-0.56834
C	-2.04245	-1.95214	-1.13843
C	-1.19435	-3.17672	-1.46691
C	-2.37392	-1.92968	0.35042
C	-3.32969	-1.97346	-1.96495
H	-0.94586	-3.22683	-2.52761
H	-0.25858	-3.19120	-0.90640
H	-1.76408	-4.07275	-1.20446
H	-2.98332	-1.07167	0.64052
H	-2.94586	-2.83125	0.58849
H	-1.47630	-1.94091	0.97080
H	-3.84754	-2.91652	-1.76981
H	-4.01083	-1.16413	-1.69409
H	-3.12463	-1.92092	-3.03628
S	-0.67128	-0.44625	-3.59520

R = Ph

P	0.68136	0.24878	0.63034
C	0.47082	-0.35971	-0.87615
P	-1.06502	-0.51262	-1.72364
Cl	1.96475	-3.22637	-2.15984
Sn	1.91882	-0.83437	-2.58945
C	-2.14968	0.91526	-1.53576
H	-3.04508	0.75005	-2.13774
H	-2.42831	1.07083	-0.49201
H	-1.62030	1.79476	-1.90316
Au	-0.80929	1.17740	1.98303
Cl	-2.39751	2.09800	3.31083
C	2.38466	0.11458	1.15505
C	2.88137	1.06660	2.04711
C	3.20912	-0.92290	0.71139
C	4.20186	1.00527	2.45835
H	2.23155	1.85515	2.41250
C	4.52589	-0.98114	1.13547
H	2.81081	-1.70759	0.07595
C	5.02403	-0.01503	1.99981
H	4.58841	1.75122	3.14265
H	5.16102	-1.79182	0.79830
H	6.05539	-0.06621	2.32956
C	-1.96825	-1.94478	-1.09839
C	-2.72894	-1.83569	0.06623
C	-1.86827	-3.16774	-1.75368
C	-3.39322	-2.94508	0.56093
H	-2.80399	-0.89447	0.59962
C	-2.53457	-4.27425	-1.25041
H	-1.26547	-3.24875	-2.65025
C	-3.29708	-4.16359	-0.09776
H	-3.98571	-2.85597	1.46382
H	-2.45321	-5.22612	-1.76173
H	-3.81784	-5.03050	0.29264
S	-0.52603	-0.73078	-3.64869

R = Mes

P	0.66980	0.45275	1.00433
C	0.30384	-0.34776	-0.37903
P	-1.16474	-0.40753	-1.35746
Cl	1.59752	-3.11710	-2.03531
Sn	1.92584	-0.70923	-1.95544
Au	-0.61052	1.62899	2.38241
Cl	-1.93365	2.83651	3.77217
C	2.43845	0.29611	1.31995
C	3.26569	1.42140	1.16189
C	2.96391	-0.93209	1.75380
C	4.62077	1.28766	1.42282
C	4.32877	-1.00884	2.00486
C	5.17199	0.08278	1.84575
H	5.26608	2.15081	1.29051
H	4.74308	-1.95467	2.34001
C	-1.99391	-2.02997	-1.20076
C	-2.48714	-2.38509	0.07413
C	-2.16411	-2.93149	-2.27960
C	-3.08111	-3.62826	0.25672
C	-2.77997	-4.15169	-2.02988
C	-3.23143	-4.53451	-0.77647
H	-3.44223	-3.88381	1.24832
H	-2.90471	-4.83562	-2.86321
C	2.71796	2.73920	0.70360
H	2.25672	2.66108	-0.28570
H	1.95044	3.11458	1.38770
H	3.50813	3.48786	0.64391
C	2.10703	-2.14651	1.95552
H	1.19217	-1.90798	2.50553
H	1.80349	-2.59025	1.00286
H	2.64992	-2.90488	2.52041
C	6.63408	-0.02281	2.14997
H	6.84800	0.36491	3.15081
H	6.97443	-1.05862	2.11761
H	7.22964	0.55817	1.44313
C	-2.47556	-1.49604	1.28319
H	-2.37997	-2.09713	2.18846
H	-1.67073	-0.76653	1.29896
H	-3.41802	-0.94590	1.36694
C	-1.74737	-2.69840	-3.70274
H	-2.14494	-1.77012	-4.11136
H	-0.66214	-2.65784	-3.79876
H	-2.11071	-3.52218	-4.31797
C	-3.84208	-5.88181	-0.55314
H	-4.47407	-6.17555	-1.39356
H	-3.06239	-6.64301	-0.45194
H	-4.44431	-5.90217	0.35623
C	-2.40136	0.86072	-0.99592
H	-2.85824	0.73280	-0.01650
H	-1.91490	1.83500	-1.05207
H	-3.17074	0.79749	-1.76859

S	-0.34654	0.06771	-3.14111

R = Mes*

P	-0.99035	0.88573	0.09493
C	-0.29310	-0.56216	0.42994
P	1.35813	-1.15223	0.66227
Cl	-2.02718	-2.82644	-1.86806
C	2.99760	-0.47362	0.16553
C	3.56141	-0.95411	-1.05310
C	3.88224	0.02856	1.15982
C	4.91068	-1.27609	-1.05531
C	5.22149	-0.34512	1.08443
C	5.74608	-1.08208	0.03614
H	5.32918	-1.69006	-1.96397
H	5.88157	-0.02005	1.87640
C	-2.79232	0.61131	0.14447
C	-3.53920	0.46243	-1.05393
C	-3.38370	0.28314	1.39142
C	-4.73471	-0.24499	-0.97862
C	-4.56758	-0.44604	1.36964
C	-5.23201	-0.77848	0.19998
H	-5.28649	-0.41297	-1.88815
H	-4.99417	-0.75969	2.31334
C	-3.17152	1.11992	-2.39828
C	-2.90590	0.74357	2.78724
C	-6.47872	-1.65514	0.24903
C	3.56915	1.11168	2.21614
C	2.88083	-0.97046	-2.43696
C	7.18606	-1.57782	0.00361
C	-4.11864	1.31529	3.54597
H	-3.78392	1.71801	4.50513
H	-4.58732	2.12534	2.98251
H	-4.88152	0.56717	3.75980
C	-1.88070	1.87600	2.76531
H	-0.88628	1.55322	2.45480
H	-2.19406	2.70164	2.12198
H	-1.76648	2.27268	3.77770
C	-2.33839	-0.42935	3.59045
H	-3.05756	-1.24772	3.66707
H	-1.43205	-0.81998	3.12420
H	-2.08654	-0.10459	4.60427
C	-7.55412	-0.97812	1.10665
H	-8.45004	-1.60368	1.14731
H	-7.21583	-0.81607	2.13218
H	-7.83524	-0.00844	0.68846
C	-7.06100	-1.91271	-1.13824
H	-7.93100	-2.56781	-1.05049
H	-7.39350	-0.98955	-1.61983
H	-6.33951	-2.40521	-1.79451
C	-6.10645	-3.00679	0.87075
H	-5.34662	-3.51482	0.27202
H	-5.71588	-2.89276	1.88429
H	-6.98797	-3.65176	0.92273
C	-4.19223	0.79748	-3.49399
H	-5.19865	1.13813	-3.24040
H	-3.89420	1.31962	-4.40563
H	-4.22583	-0.27058	-3.72207
C	-3.19961	2.64182	-2.19814
H	-4.18745	2.97059	-1.86541
H	-2.46797	2.97570	-1.45886
H	-2.96924	3.14755	-3.13956
C	-1.81305	0.68460	-2.94932
H	-0.97393	1.03235	-2.34479
H	-1.74708	-0.40029	-3.03412
H	-1.66986	1.12602	-3.93969
C	2.18443	1.73395	2.08142
H	2.06365	2.18178	1.09313
H	1.35219	1.05276	2.23968
H	2.07668	2.53813	2.81321
C	4.53660	2.28097	1.92569
H	4.29039	3.11911	2.58286
H	5.57923	2.01564	2.10107
H	4.43762	2.62311	0.89368
C	3.83213	0.64975	3.65345
H	4.85457	0.28540	3.77243
H	3.70322	1.49361	4.33635
H	3.16142	-0.14508	3.97730
C	1.43819	-0.48627	-2.44915
H	1.12850	-0.33536	-3.48605
H	0.74222	-1.20307	-2.01669
H	1.33930	0.47524	-1.93848
C	2.95675	-2.33696	-3.12266
H	2.38612	-3.08528	-2.57334
H	2.53641	-2.25923	-4.12879
H	3.98450	-2.69171	-3.22338
C	3.66954	0.05070	-3.28446
H	3.63483	1.04514	-2.83291
H	4.71643	-0.22894	-3.40869
H	3.21796	0.11413	-4.27799
C	7.92959	-1.28065	1.30303
H	8.02917	-0.20691	1.48128
H	7.43183	-1.72963	2.16634
H	8.93829	-1.69667	1.24878
C	7.93863	-0.90297	-1.14945
H	7.48603	-1.12779	-2.11712
H	7.95040	0.18284	-1.02785
H	8.97384	-1.25396	-1.17700
C	7.18033	-3.09586	-0.21320
H	6.66073	-3.60599	0.60149
H	6.68543	-3.36885	-1.14719
H	8.20574	-3.47336	-0.25325
Sn	-1.34689	-2.65030	0.45557
Au	-0.02540	2.83790	-0.38721
Cl	0.98764	4.81737	-0.86105
S	1.21884	-3.03760	-0.08420
C	1.46062	-1.48367	2.45047
H	0.65329	-2.18529	2.66642
H	2.40904	-1.98636	2.64263
H	1.35504	-0.61109	3.08662

Coordination through π(C=P) bond

R= H

P	0.68588	0.03286	1.07565
C	0.54022	0.05431	-0.64286
P	-0.98495	-0.05900	-1.53265
Cl	1.26184	-2.76197	-2.30204
Sn	1.90102	-0.42931	-2.46601
C	-2.39630	0.86457	-0.90205
H	-3.27111	0.63202	-1.51267
H	-2.58652	0.57809	0.13506
H	-2.18630	1.93318	-0.95956
Au	0.92260	2.10222	-0.08201
Cl	1.28854	4.32101	-0.16498
H	2.09270	-0.20866	1.15804
H	-1.37370	-1.40709	-1.41178
S	-0.49241	0.40299	-3.41113

R = Me

P	0.65646	0.03055	1.02380
C	0.46822	0.12009	-0.68445
P	-1.07992	-0.11080	-1.52111

Cl	1.75346	-2.66386	-2.15708
Sn	1.85038	-0.25254	-2.51241
C	2.43786	-0.32763	1.27316
H	2.52893	-1.41210	1.39518
H	3.07023	-0.01239	0.44281
H	2.77650	0.14632	2.19651
C	-1.67235	-1.76892	-1.14197
H	-1.93549	-1.83656	-0.08392
H	-2.54647	-1.98275	-1.75965
H	-0.87641	-2.47783	-1.37697
C	-2.40935	1.01022	-1.03972
H	-3.30850	0.75241	-1.60369
H	-2.60731	0.91302	0.03015
H	-2.12335	2.03688	-1.26864
Au	0.74444	2.16600	-0.05766
Cl	1.00001	4.40468	-0.00129
S	-0.61699	0.08606	-3.46598

R = *t*-Bu

P	0.99470	0.14204	1.01469
C	0.61181	0.10737	-0.66658
P	-1.05585	-0.21541	-1.24216
Cl	2.31444	-2.39985	-2.51025
Sn	1.71861	-0.04457	-2.71814
C	-1.76787	-1.79127	-0.55537
Au	0.85816	2.21351	-0.17643
Cl	1.04559	4.46137	-0.23300
C	2.83087	-0.10518	1.27272
C	3.74479	0.21046	0.10274
C	3.18667	0.76425	2.48021
C	2.94664	-1.58869	1.64572
H	3.59161	-0.48569	-0.72542
H	3.62283	1.23688	-0.25196
H	4.78788	0.09379	0.41620
H	2.52254	0.57485	3.32822
H	4.20889	0.54123	2.80060
H	3.13357	1.82891	2.23932
H	3.97269	-1.79816	1.96480
H	2.28473	-1.85695	2.47462
H	2.72209	-2.23377	0.79268
C	-2.22574	1.09576	-0.80622
H	-2.25005	1.25116	0.27316
H	-1.91220	2.01595	-1.29942
H	-3.22063	0.82703	-1.16582
C	-2.17655	-1.59090	0.90351
H	-2.52746	-2.54903	1.29771
H	-1.34337	-1.26293	1.52904
H	-2.99347	-0.87458	1.00995
C	-0.73488	-2.90738	-0.67044
H	0.13971	-2.72525	-0.04441
H	-1.19565	-3.84293	-0.34036
H	-0.38722	-3.04029	-1.69567
C	-2.99694	-2.14927	-1.39280
H	-3.42091	-3.07733	-0.99897
H	-3.77714	-1.38636	-1.34141
H	-2.73752	-2.31115	-2.43978
S	-0.86846	-0.31621	-3.24660

R = Ph

P	0.57579	0.26509	0.93364
C	0.37614	0.19330	-0.77567
P	-1.19556	-0.01612	-1.57213
Cl	1.73769	-2.38245	-2.79953
Sn	1.71719	0.05115	-2.67501
C	-2.52526	1.03161	-0.94147
H	-3.44203	0.79097	-1.48302
H	-2.67506	0.85224	0.12501
H	-2.26481	2.07784	-1.10479
Au	0.68576	2.30160	-0.32021
Cl	0.97128	4.53599	-0.44245
C	2.33411	-0.07252	1.22524
C	2.95126	0.57853	2.29585
C	3.05153	-1.01589	0.48816
C	4.27968	0.32638	2.59243
H	2.39257	1.29744	2.88703
C	4.37697	-1.27623	0.80063
H	2.56908	-1.57266	-0.30882
C	4.99370	-0.60018	1.84376
H	4.75884	0.85015	3.41140
H	4.92667	-2.01340	0.22679
H	6.03171	-0.80275	2.08161
C	-1.75886	-1.71222	-1.30107
C	-1.84695	-2.60659	-2.36052
C	-2.11620	-2.11753	-0.01494
C	-2.29986	-3.89821	-2.13646
H	-1.54913	-2.29033	-3.35280
C	-2.56587	-3.40824	0.20265
H	-2.03492	-1.43388	0.82404
C	-2.65955	-4.29838	-0.85951
H	-2.36241	-4.59464	-2.96418
H	-2.84075	-3.72143	1.20306
H	-3.00947	-5.30973	-0.68680
S	-0.81646	0.37826	-3.50163

R = Mes

P	0.51924	0.34083	1.35444
C	0.15410	0.26475	-0.33557
P	-1.35775	0.05896	-1.26228
Cl	1.82119	-1.82057	-2.78882
Sn	1.66923	0.51311	-2.08213
Au	0.20234	2.39189	0.17476
Cl	0.09796	4.65355	0.13284
C	2.32376	0.04960	1.40397
C	3.23620	1.02039	1.84897
C	2.76222	-1.26359	1.14060
C	4.58395	0.68812	1.91529
C	4.11924	-1.54338	1.22665
C	5.05046	-0.57738	1.58666
H	5.28941	1.44403	2.24761
H	4.45448	-2.55561	1.02118
C	-1.81603	-1.71123	-1.33218
C	-1.84856	-2.46637	-2.52964
C	-2.15258	-2.34435	-0.11455
C	-2.13717	-3.82365	-2.44826
C	-2.41968	-3.70810	-0.10289
C	-2.39983	-4.47521	-1.25383
H	-2.15397	-4.39363	-3.37158
H	-2.66243	-4.17674	0.84611
C	2.81383	2.39739	2.26370
H	2.67742	3.06119	1.40451
H	1.86881	2.38977	2.81338
H	3.57015	2.84904	2.90749
C	1.80811	-2.37998	0.82921
H	0.94018	-2.36698	1.49649
H	1.43661	-2.33126	-0.19698
H	2.30452	-3.34325	0.95318
C	6.51275	-0.89588	1.62710
H	6.68698	-1.93446	1.91438
H	6.96488	-0.75081	0.64090
H	7.04297	-0.24973	2.32868
C	-2.32969	-1.64862	1.20572
H	-2.07783	-2.32709	2.02214

H	-1.72435	-0.75528	1.33918
H	-3.37787	-1.36296	1.34158
C	-1.62885	-1.93281	-3.91513
H	-2.26159	-1.07373	-4.13574
H	-0.59472	-1.62449	-4.06773
H	-1.85804	-2.71713	-4.63741
C	-2.64784	-5.94999	-1.21502
H	-3.21473	-6.28068	-2.08738
H	-1.70002	-6.49708	-1.21764
H	-3.19433	-6.24056	-0.31655
C	-2.83399	0.94145	-0.68525
H	-2.61549	2.01007	-0.68058
H	-3.62958	0.74267	-1.40728
H	-3.15628	0.62310	0.30362
S	-0.81117	0.97284	-2.97681

R = Mes*

P	0.68894	0.00840	1.07969
C	0.43223	0.09078	-0.64221
P	-1.10127	-0.05758	-1.63281
Cl	3.01878	-1.30238	-3.25498
Au	0.38417	2.17451	0.03600
Cl	0.08130	4.42929	-0.00271
C	2.53884	-0.04968	1.21392
C	3.25575	-1.17719	0.72719
C	3.27933	1.08349	1.63372
C	4.56655	-0.99012	0.31380
C	4.59326	1.20318	1.17632
C	5.23287	0.22399	0.43672
H	5.09612	-1.83592	-0.10527
H	5.13738	2.10088	1.43416
C	2.87293	2.10642	2.71696
C	2.79832	-2.64102	0.87909
C	6.62687	0.40107	-0.15357
C	3.83897	1.85463	3.89367
H	3.74151	0.83242	4.26832
H	4.88026	2.01010	3.60970
H	3.60478	2.54238	4.71090
C	3.04355	3.55506	2.25241
H	2.38393	3.79739	1.41833
H	2.79851	4.23343	3.07428
H	4.06900	3.77012	1.94762
C	1.47265	1.93899	3.29952
H	1.38027	2.58179	4.17911
H	0.67998	2.24400	2.61316
H	1.28176	0.91257	3.62253
C	3.08018	-3.48635	-0.36391
H	2.59246	-3.07904	-1.24736
H	4.14689	-3.55484	-0.58204
H	2.71951	-4.50570	-0.19970
C	3.62907	-3.20338	2.04903
H	4.69829	-3.16138	1.83569
H	3.44661	-2.64031	2.96796
H	3.35679	-4.24808	2.22678
C	1.33720	-2.82689	1.26921
H	1.08578	-2.29291	2.19085
H	0.65046	-2.52174	0.47986
H	1.15125	-3.88783	1.45940
C	7.21136	1.77860	0.14672
H	6.58136	2.58054	-0.24589
H	7.34810	1.94031	1.21936
H	8.19227	1.86820	-0.32610
C	6.53556	0.23424	-1.67523
H	7.52901	0.32460	-2.12382
H	6.12137	-0.73626	-1.95441
H	5.89329	1.00155	-2.11434
C	7.57723	-0.65780	0.41743
H	7.64842	-0.57607	1.50506
H	7.25245	-1.67146	0.17531
H	8.57897	-0.52431	-0.00013
C	-2.35366	-1.43971	-1.67148
C	-2.33468	-2.33760	-2.77864
C	-3.59751	-1.24557	-1.00823
C	-3.54007	-2.61291	-3.40956
C	-1.14755	-3.21734	-3.21230
C	-4.76476	-1.58081	-1.68840
C	-3.79267	-0.89299	0.48014
C	-4.76804	-2.16421	-2.94508
H	-3.51141	-3.23438	-4.29607
C	-0.74261	-3.03584	-4.67653
C	-1.62487	-4.67482	-3.02998
C	0.06054	-3.07441	-2.30351
H	-5.70881	-1.38247	-1.20010
C	-2.50175	-0.61783	1.23321
C	-4.37981	-2.16880	1.12038
C	-4.78373	0.25061	0.71807
C	-6.03812	-2.41140	-3.74851
H	0.00162	-3.79129	-4.94161
H	-1.59559	-3.16081	-5.34776
H	-0.30595	-2.05514	-4.85290
H	-2.42664	-4.94440	-3.71841
H	-0.78388	-5.34739	-3.21874
H	-1.97888	-4.85037	-2.01102
H	0.53259	-2.09689	-2.36406
H	-0.21396	-3.28733	-1.26825
H	0.82234	-3.79741	-2.60164
H	-1.77277	-1.41429	1.07247
H	-2.03492	0.33280	0.98007
H	-2.70525	-0.57688	2.30587
H	-4.51269	-2.00822	2.19389
H	-5.34808	-2.43380	0.69453
H	-3.70652	-3.01815	0.98302
H	-5.76943	0.03197	0.30433
H	-4.90904	0.40379	1.79307
H	-4.44832	1.19437	0.28664
C	-7.27435	-1.82863	-3.06898
C	-6.25136	-3.91907	-3.92970
C	-5.88600	-1.75155	-5.12428
H	-7.47864	-2.30954	-2.10889
H	-7.17590	-0.75300	-2.90235
H	-8.14844	-1.98697	-3.70488
H	-5.42207	-4.38551	-4.46489
H	-6.35259	-4.41962	-2.96360
H	-7.16315	-4.10182	-4.50488
H	-5.74830	-0.67191	-5.02786
H	-5.02892	-2.14981	-5.67076
H	-6.78098	-1.92848	-5.72695
Sn	1.95611	0.70522	-2.34199
S	-0.36306	0.21921	-3.50186
C	-2.21365	1.37887	-1.47033
H	-2.63111	1.51366	-0.47759
H	-1.65384	2.26836	-1.75803
H	-3.01723	1.22274	-2.19246

[R-P=C(Pb(II)Cl)-P(O)ClR](AuCl)
Coordination through Pb(II) atom

R= H

P	0.82625	0.08960	0.83597
C	0.43990	-0.10123	-0.76223
P	-1.16184	-0.22556	-1.46941
O	-0.95473	0.03880	-2.93819
Pb	1.44180	-0.06541	-2.84078
Cl	1.50836	-2.49219	-3.08066
Au	2.75524	1.61751	-4.31006
Cl	3.91132	3.11928	-5.52527
Cl	-2.48653	1.01556	-0.62389
H	2.24762	0.15470	0.69822
H	-1.75848	-1.47008	-1.21022

R = Me

P	0.84400	0.02204	0.83673
C	0.46128	-0.10713	-0.76451
P	-1.13906	-0.27436	-1.47205
O	-0.91374	0.00518	-2.94371
Pb	1.44169	-0.01375	-2.84028
Cl	1.60115	-2.45129	-3.09808
C	-1.88510	-1.86885	-1.17571
H	-2.05252	-2.01780	-0.10861
H	-2.83154	-1.92299	-1.71622
H	-1.19551	-2.62412	-1.56024
C	2.67101	0.14324	0.88877
H	3.13274	0.27682	-0.08960
H	2.95051	0.97191	1.54459
H	3.05852	-0.77162	1.34815
Au	2.72699	1.63737	-4.36937
Cl	3.86819	3.11996	-5.62653
Cl	-2.44286	1.04065	-0.67473

R = *t*-Bu

P	0.82212	-0.24810	0.72314
C	0.53661	-0.16788	-0.90234
P	-1.00883	-0.44383	-1.71200
O	-0.68185	-0.23893	-3.17908
Pb	1.59593	0.28947	-2.89329
Cl	2.46217	-1.97849	-3.28547
Au	2.49053	2.31568	-4.24284
Cl	3.27945	4.12839	-5.33147
C	2.62801	0.11121	1.05047
C	3.17182	-1.16160	1.70846
C	3.46602	0.47936	-0.15763
C	2.62455	1.25577	2.06918
H	2.56996	-1.46264	2.57039
H	3.20445	-1.99656	1.00447
H	4.19112	-0.97952	2.06365
H	3.10147	1.38960	-0.64422
H	4.49551	0.68481	0.15315
H	3.51644	-0.34121	-0.88026
H	3.64571	1.43346	2.42067
H	2.25313	2.18604	1.63219
H	2.00855	1.02075	2.94139
C	-1.85092	-2.04196	-1.41587
C	-3.07380	-2.09258	-2.33400
C	-0.85383	-3.13618	-1.80924
C	-2.26060	-2.18827	0.04547
H	-3.81332	-1.33496	-2.06930
H	-2.79530	-1.95835	-3.38042
H	-3.54356	-3.07433	-2.22817
H	0.03707	-3.12664	-1.17908
H	-1.34484	-4.10549	-1.68710
H	-0.53492	-3.04383	-2.84825
H	-2.72276	-3.17032	0.17883
H	-1.40384	-2.13098	0.72079
H	-2.98701	-1.43090	0.34376
Cl	-2.31985	0.97532	-1.09350

R = Ph

P	0.91582	0.11942	0.80099
C	0.47807	-0.20490	-0.76216
P	-1.12129	-0.37905	-1.46430
O	-0.88138	-0.19743	-2.94673
Pb	1.49701	-0.09849	-2.80078
Cl	1.80334	-2.51563	-3.16241
Au	2.70888	1.60997	-4.32884
Cl	3.78527	3.12890	-5.60381
C	2.72328	0.16468	0.74668
C	3.36125	1.06982	1.59760
C	3.49671	-0.66614	-0.07082
C	4.74200	1.18719	1.58582
H	2.77073	1.69709	2.25811
C	4.87728	-0.55671	-0.06696
H	3.02198	-1.43502	-0.67365
C	5.49931	0.37800	0.75116
H	5.22827	1.90889	2.23168
H	5.47012	-1.20890	-0.69772
H	6.57959	0.46522	0.74725
C	-1.92836	-1.92661	-1.08886
C	-1.90979	-2.91792	-2.06716
C	-2.50948	-2.17049	0.15449
C	-2.48143	-4.15203	-1.79788
H	-1.45016	-2.71983	-3.02752
C	-3.07410	-3.40608	0.41378
H	-2.52867	-1.39592	0.91283
C	-3.06041	-4.39536	-0.56219
H	-2.47135	-4.92423	-2.55779
H	-3.53033	-3.59836	1.37762
H	-3.50620	-5.36162	-0.35555
Cl	-2.35581	1.04233	-0.73137

R = Mes

P	1.03272	-0.17782	1.32694
C	0.45747	-0.35234	-0.21494
P	-1.09670	-0.39443	-1.05173
O	-0.71804	0.10809	-2.42694
Pb	1.66105	0.08948	-2.08274
Cl	1.95330	-2.22874	-2.87377
Au	3.10342	1.98894	-3.10187
Cl	4.41427	3.66972	-3.85500
C	2.81298	0.06606	0.97679
C	3.32090	1.37633	0.94656
C	3.65523	-1.03387	0.75465
C	4.66046	1.56394	0.63812
C	4.99152	-0.79246	0.45157
C	5.50989	0.49383	0.37450
H	5.05047	2.57631	0.59344
H	5.64658	-1.63996	0.27398
C	-1.95461	-1.96969	-1.03585
C	-2.22006	-2.64981	-2.24739
C	-2.33819	-2.54137	0.19727
C	-2.83239	-3.89570	-2.17242
C	-2.93664	-3.79200	0.19891
C	-3.18689	-4.49206	-0.97232
H	-3.03787	-4.41859	-3.10085
H	-3.22312	-4.22727	1.15115
Cl	-2.36113	0.95815	-0.21372

C	2.43853	2.56179	1.20586
H	1.76797	2.39318	2.05464
H	3.03439	3.44965	1.41896
H	1.80848	2.79212	0.34072
C	3.14887	-2.44362	0.83637
H	2.47622	-2.58207	1.68838
H	2.59471	-2.72331	-0.06456
H	3.97763	-3.14398	0.94630
C	6.94152	0.73398	0.00931
H	7.01190	1.20237	-0.97661
H	7.42307	1.40845	0.72124
H	7.50845	-0.19714	-0.01872
C	-2.18099	-1.85610	1.52065
H	-1.21014	-1.37482	1.64658
H	-2.94018	-1.08141	1.65231
H	-2.29470	-2.57702	2.33055
C	-1.90057	-2.13728	-3.62152
H	-2.30530	-1.14163	-3.79784
H	-0.82318	-2.07558	-3.78342
H	-2.31663	-2.81899	-4.36352
C	-3.80352	-5.85458	-0.94018
H	-3.02790	-6.62207	-0.85395
H	-4.47328	-5.96965	-0.08633
H	-4.36548	-6.05932	-1.85267

R = Mes*

P	0.01610	2.43878	1.03925
C	0.41548	0.91920	0.50664
P	1.88761	-0.03836	0.29832
Cl	-1.27139	0.34757	-2.71795
O	1.46525	-1.02443	-0.77082
C	3.62361	0.44237	0.04506
C	4.13921	0.20461	-1.26465
C	4.53529	0.54255	1.12853
C	5.42723	-0.29869	-1.35892
C	5.81969	0.04192	0.94047
C	6.26101	-0.48074	-0.26331
H	5.80007	-0.55147	-2.34275
H	6.50039	0.07021	1.77948
C	-1.80421	2.39584	0.78169
C	-2.36301	3.09700	-0.31136
C	-2.61044	1.51243	1.54164
C	-3.61631	2.69470	-0.76507
C	-3.83305	1.12819	0.99736
C	-4.33552	1.66031	-0.18355
H	-4.03449	3.18930	-1.62678
H	-4.43148	0.40361	1.53495
C	-1.67948	4.30781	-0.97410
C	-2.31642	1.06465	2.99128
C	-5.65195	1.14505	-0.75600
C	4.32416	1.31491	2.44633
C	3.46211	0.59049	-2.59506
C	7.62169	-1.14356	-0.43438
C	-3.59325	1.28169	3.82411
H	-3.37630	1.07542	4.87510
H	-3.94744	2.31211	3.74409
H	-4.40664	0.61917	3.52847
C	-1.23546	1.90054	3.67494
H	-0.23046	1.69984	3.29241
H	-1.43503	2.97193	3.59141
H	-1.20500	1.64897	4.73849
C	-1.93352	-0.41415	3.07910
H	-2.67500	-1.05407	2.59505
H	-0.95989	-0.60464	2.62234
H	-1.86531	-0.72165	4.12645
C	-6.77637	1.34924	0.26625
H	-7.72214	0.98192	-0.14096
H	-6.58610	0.80906	1.19576
H	-6.89878	2.40781	0.50952
C	-6.04068	1.86212	-2.04620
H	-6.97310	1.43970	-2.42768
H	-6.20588	2.93118	-1.88755
H	-5.28081	1.73917	-2.82177
C	-5.50638	-0.35032	-1.06261
H	-4.71955	-0.52296	-1.80103
H	-5.26307	-0.93144	-0.17065
H	-6.44268	-0.74388	-1.46724
C	-2.59916	4.99255	-1.98809
H	-3.54550	5.30519	-1.54057
H	-2.09962	5.88834	-2.36375
H	-2.81249	4.35375	-2.84810
C	-1.35262	5.34940	0.10499
H	-2.25839	5.66334	0.62947
H	-0.64625	4.97264	0.84787
H	-0.90196	6.23269	-0.35593
C	-0.40335	3.92494	-1.72903
H	0.39883	3.60135	-1.05969
H	-0.58783	3.11671	-2.43825
H	-0.02542	4.79508	-2.27375
C	2.96811	1.99357	2.57219
H	2.74197	2.59739	1.69064
H	2.15456	1.29150	2.74400
H	2.98365	2.67128	3.42940
C	5.35771	2.46053	2.42540
H	5.23094	3.07594	3.32017
H	6.38465	2.09491	2.41279
H	5.21570	3.09910	1.55019
C	4.58135	0.45560	3.68736
H	5.58335	0.02296	3.68436
H	4.49467	1.07581	4.58361
H	3.86219	-0.35890	3.76831
C	2.27520	1.53223	-2.41236
H	1.96855	1.91531	-3.38838
H	1.39460	1.05053	-1.99730
H	2.54575	2.38940	-1.78981
C	3.05732	-0.63561	-3.41770
H	2.30551	-1.22829	-2.90116
H	2.64770	-0.31382	-4.37893
H	3.92192	-1.27304	-3.61975
C	4.49054	1.39919	-3.41256
H	4.85931	2.25975	-2.84885
H	5.34905	0.80629	-3.72832
H	4.00367	1.76991	-4.31785
C	8.37636	-1.26553	0.88669
H	8.61574	-0.28806	1.31336
H	7.80911	-1.83625	1.62616
H	9.32108	-1.78774	0.71888
C	8.47752	-0.32296	-1.40679
H	8.01461	-0.24758	-2.39258
H	8.63548	0.69084	-1.03073
H	9.45553	-0.79502	-1.53367
C	7.41406	-2.55228	-1.00388
H	6.81534	-3.16474	-0.32562
H	6.90498	-2.52791	-1.96931
H	8.37970	-3.04484	-1.14618
Au	-2.32491	-2.77951	-0.02340
Cl	-3.60760	-4.57959	0.46413
Pb	-0.89832	-0.66033	-0.49528
Cl	1.96322	-1.16355	2.01623

Coordination through O atom

R= H

P	0.84877	0.08847	1.00871
C	0.57903	-0.13388	-0.61043
P	-0.96185	-0.01132	-1.38567
O	-0.62575	0.43645	-2.81132
Pb	1.81260	-0.29252	-2.66548
Cl	1.17284	-2.66380	-2.94281
Au	-2.00955	0.70978	-4.37607
Cl	-3.44059	1.00763	-6.04838
Cl	-2.28292	1.21647	-0.52203
H	2.26833	-0.10522	1.01343
H	-1.65778	-1.22878	-1.38987

R = Me

P	0.84716	0.02016	1.00575
C	0.58394	-0.13384	-0.61882
P	-0.97649	-0.02727	-1.36347
O	-0.64266	0.39300	-2.80946
Pb	1.77533	-0.17715	-2.70163
Cl	1.35674	-2.60755	-3.02204
C	-1.90450	-1.54937	-1.32721
H	-2.15733	-1.80215	-0.29686
H	-2.81207	-1.42902	-1.92186
H	-1.27040	-2.32525	-1.76397
C	2.65425	-0.21871	1.22192
H	3.20386	-0.29483	0.28362
H	3.05227	0.60510	1.82079
H	2.80889	-1.13441	1.80138
Au	-2.02981	0.63261	-4.37012
Cl	-3.47511	0.89014	-6.03896
Cl	-2.20008	1.34283	-0.53878

R = *t*-Bu

P	0.91268	-0.55250	0.93666
C	0.48729	-0.09767	-0.59635
P	-1.15988	-0.10107	-1.16404
O	-1.05556	0.61215	-2.52671
Pb	1.42041	0.94688	-2.54784
Cl	2.05271	-1.20915	-3.62991
Au	-2.48630	1.50736	-3.77270
Cl	-3.95035	2.48857	-5.12824
C	2.77285	-0.41805	1.10038
C	3.25659	-1.85566	1.31933
C	3.49496	0.20394	-0.07876
C	3.01314	0.40312	2.37018
H	2.72922	-2.33879	2.14659
H	3.12068	-2.46640	0.42358
H	4.32332	-1.84955	1.56620
H	3.16255	1.23358	-0.25200
H	4.57078	0.25217	0.12033
H	3.36807	-0.39384	-0.98701
H	4.08120	0.40820	2.60996
H	2.69066	1.44061	2.24893
H	2.48128	-0.01614	3.22842
C	-1.99430	-1.72658	-1.31818
C	-3.39039	-1.52820	-1.90962
C	-1.13490	-2.56975	-2.26489
C	-2.08903	-2.38941	0.05342
H	-4.03252	-0.93519	-1.25683
H	-3.35900	-1.04957	-2.89036
H	-3.84976	-2.51314	-2.03121
H	-0.12344	-2.72065	-1.88438
H	-1.60889	-3.54928	-2.37151
H	-1.05906	-2.12119	-3.25627
H	-2.55973	-3.36826	-0.07194
H	-1.10735	-2.54784	0.50462
H	-2.69985	-1.80930	0.74722
Cl	-2.35032	1.00683	0.04170

R = Ph

P	0.81997	0.26792	0.92298
C	0.54382	-0.22435	-0.63333
P	-0.94397	-0.18155	-1.50909
O	-0.48059	-0.04563	-2.96840
Pb	1.99889	-0.39168	-2.50378
Cl	1.87995	-2.87108	-2.74566
Au	-1.67552	0.18293	-4.67973
Cl	-2.89248	0.42846	-6.52532
C	2.59667	-0.04494	1.13219
C	3.27280	-1.10288	0.51381
C	3.30638	0.81317	1.97419
C	4.63609	-1.26723	0.70275
H	2.71920	-1.83240	-0.07127
C	4.67232	0.65407	2.15009
H	2.78583	1.61872	2.48301
C	5.33824	-0.38256	1.51168
H	5.14817	-2.09743	0.22970
H	5.21765	1.33686	2.79139
H	6.40429	-0.51284	1.65809
C	-1.98225	-1.61394	-1.27846
C	-2.86396	-1.70227	-0.20221
C	-1.84333	-2.67829	-2.16744
C	-3.60958	-2.85321	-0.02285
H	-2.97632	-0.87165	0.48476
C	-2.59784	-3.82526	-1.97949
H	-1.15138	-2.61285	-2.99778
C	-3.47754	-3.91262	-0.91176
H	-4.30147	-2.92185	0.80814
H	-2.49715	-4.65005	-2.67450
H	-4.06826	-4.81056	-0.77115
Cl	-2.09597	1.40062	-1.01427

R = Mes

P	0.71383	-0.01967	1.36234
C	0.37362	-0.34172	-0.22212
P	-1.03561	-0.25823	-1.22640
O	-0.43168	0.14269	-2.57936
Pb	2.05115	-0.19630	-1.87612
Cl	2.11440	-2.63997	-2.39790
Au	-1.08856	1.41592	-4.11456
Cl	-1.69973	2.77214	-5.76897
C	2.54781	-0.13683	1.33487
C	3.19179	-1.38608	1.37003
C	3.30029	1.05017	1.33478
C	4.58163	-1.41752	1.35828
C	4.68736	0.96574	1.32094
C	5.34715	-0.25762	1.32422
H	5.08049	-2.38164	1.38881
H	5.26813	1.88348	1.31848
C	-2.01966	-1.75827	-1.27481
C	-2.66777	-2.17360	-0.08961
C	-2.11645	-2.54334	-2.44829
C	-3.38012	-3.36359	-0.09686
C	-2.85499	-3.71879	-2.38525
C	-3.49178	-4.15126	-1.23222
H	-3.86614	-3.68001	0.82070
H	-2.92553	-4.32321	-3.28365
Cl	-2.28609	1.25013	-0.69296
C	2.63240	2.39458	1.34267
H	2.14789	2.61658	0.38653

H	1.85367	2.45312	2.10954
H	3.35551	3.18720	1.53748
C	2.41817	-2.66920	1.43347
H	1.59244	-2.60575	2.14831
H	1.98724	-2.92307	0.46072
H	3.06506	-3.49309	1.73691
C	6.84263	-0.32707	1.28448
H	7.19627	-0.46858	0.25847
H	7.29483	0.59038	1.66409
H	7.21727	-1.16561	1.87433
C	-2.67225	-1.38989	1.18872
H	-1.70762	-0.94109	1.42911
H	-3.40086	-0.57690	1.14512
H	-2.94728	-2.03938	2.02024
C	-1.48330	-2.21987	-3.76873
H	-1.82768	-1.26445	-4.16906
H	-0.39620	-2.17337	-3.69557
H	-1.73768	-2.99637	-4.48978
C	-4.29400	-5.41378	-1.22300
H	-4.35475	-5.84097	-0.22093
H	-5.31677	-5.21874	-1.56046
H	-3.86701	-6.16091	-1.89409

R = Mes*

P	1.72102	2.31195	-0.18726
C	0.82149	0.92373	-0.24172
P	-0.87610	0.57350	-0.33864
C	3.41059	1.55931	-0.20361
C	4.17995	1.54676	0.98832
C	3.84945	0.81862	-1.32388
C	5.19810	0.61023	1.09270
C	4.85333	-0.13785	-1.12464
C	5.50835	-0.29708	0.08387
H	5.76268	0.55983	2.01193
H	5.15430	-0.74594	-1.96722
C	3.47079	1.09129	-2.79847
C	3.96288	2.54743	2.13796
C	6.57867	-1.35840	0.31605
C	2.63539	2.31601	2.86619
H	1.76971	2.55456	2.24289
H	2.53417	1.27784	3.18677
H	2.58172	2.96588	3.74452
C	4.01290	3.97629	1.58007
H	4.97089	4.16946	1.09087
H	3.21842	4.16997	0.85652
H	3.89524	4.69625	2.39460
C	5.06637	2.44579	3.19387
H	5.04383	1.49062	3.72351
H	6.06155	2.58685	2.76513
H	4.91256	3.23189	3.93639
C	2.81528	-0.12290	-3.46154
H	1.81582	-0.30457	-3.05796
H	2.69970	0.05393	-4.53440
H	3.41157	-1.02987	-3.33996
C	2.57096	2.30457	-3.02254
H	1.53881	2.12898	-2.71831
H	2.94870	3.19449	-2.51102
H	2.54634	2.53384	-4.09156
C	4.78517	1.40683	-3.53905
H	5.29278	2.26019	-3.08295
H	5.47874	0.56585	-3.54029
H	4.56600	1.65733	-4.58020
C	6.74110	-2.28798	-0.88365
H	7.08136	-1.75375	-1.77464
H	7.49015	-3.04941	-0.65478
H	5.80919	-2.80596	-1.12532
C	6.19468	-2.20720	1.53389
H	6.11262	-1.60436	2.43995
H	5.23320	-2.70341	1.38502
H	6.95475	-2.97350	1.70893
C	7.92441	-0.67216	0.58026
H	7.88217	-0.02452	1.45826
H	8.70015	-1.42241	0.75635
H	8.22843	-0.06183	-0.27390
C	-2.36201	1.41229	0.28123
C	-2.94943	0.83663	1.44726
C	-3.17730	2.19635	-0.57565
C	-4.32963	0.72592	1.47543
C	-4.55491	2.03838	-0.47174
C	-5.15481	1.22010	0.47256
H	-4.78128	0.22587	2.32267
H	-5.18096	2.58676	-1.16185
C	-2.20747	0.48091	2.75078
C	-2.31088	-1.00220	3.11131
C	-2.88792	1.30119	3.86639
H	-1.76642	-1.62502	2.40229
H	-1.88269	-1.16741	4.10366
H	-3.34835	-1.34200	3.13188
H	-2.86331	2.37032	3.64099
H	-3.92693	1.01355	4.02790
H	-2.35020	1.13824	4.80392
C	-2.70366	3.35447	-1.47609
C	-3.35375	4.62256	-0.88333
C	-3.16609	3.21392	-2.92862
H	-3.05781	4.76290	0.15907
H	-3.02362	5.49610	-1.45219
H	-4.44265	4.58728	-0.92288
H	-2.71846	2.34704	-3.41334
H	-4.25073	3.11764	-3.00226
H	-2.87641	4.10561	-3.49126
C	-6.64886	0.93042	0.49538
C	-7.27133	1.53918	1.75777
C	-6.85351	-0.58979	0.50633
H	-7.13830	2.62393	1.77965
H	-8.34357	1.32687	1.78386
H	-6.82920	1.12669	2.66688
H	-6.40256	-1.06190	-0.36899
H	-6.40925	-1.05384	1.38871
H	-7.92248	-0.81971	0.51059
Cl	2.18325	-1.44677	2.21666
O	-0.93774	-0.89041	0.14358
Cl	-1.19537	0.39488	-2.35896
Au	-2.51832	-2.20725	-0.31500
Cl	-4.16174	-3.63697	-0.79988
C	-1.20239	3.61218	-1.43773
H	-0.83562	3.68963	-0.41156
H	-0.62382	2.85565	-1.96369
H	-0.99022	4.56523	-1.92873
C	-0.74576	0.91166	2.76257
H	-0.10288	0.28840	2.14447
H	-0.63572	1.95670	2.46115
H	-0.35468	0.81788	3.77844
C	-7.36548	1.50540	-0.72347
H	-7.31878	2.59743	-0.75178
H	-6.95033	1.11528	-1.65597
H	-8.42091	1.22599	-0.68854
Pb	1.60702	-1.33172	-0.21863

Coordination through P(sp^2) atom

R= H

P	0.70535	0.09431	0.86507
C	0.43930	-0.22943	-0.71387
P	-1.07866	-0.27753	-1.56076
O	-0.74098	-0.09578	-3.01909
Pb	1.62465	-0.48751	-2.83366
Cl	1.34742	-2.95095	-2.76806
Au	-0.58472	0.57679	2.57750
Cl	-1.89374	1.06734	4.34777
Cl	-2.42307	1.06999	-0.92886
H	2.10427	0.01539	1.04435
H	-1.75391	-1.48003	-1.29849

R = Me

P	0.80736	0.13245	0.80916
C	0.48623	-0.15291	-0.76470
P	-1.08496	-0.29468	-1.51087
O	-0.81073	-0.28137	-3.00084
Pb	1.54324	-0.51275	-2.91973
Cl	1.45050	-2.98832	-2.60824
C	-1.97331	-1.75075	-0.98544
H	-2.19009	-1.69666	0.08223
H	-2.90182	-1.82255	-1.55424
H	-1.33709	-2.61236	-1.20209
C	2.58771	0.11689	1.13268
H	3.15702	-0.07248	0.22371
H	2.87783	1.07487	1.57064
H	2.79907	-0.66036	1.87130
Au	-0.55994	0.47158	2.50762
Cl	-1.95522	0.78663	4.25927
Cl	-2.29996	1.24142	-1.01853

R = *t*-Bu

P	0.76640	0.08766	0.91443
C	0.47641	-0.16748	-0.67354
P	-1.06272	-0.35743	-1.48556
O	-0.74057	-0.17920	-2.95809
Pb	1.60959	0.09829	-2.80776
Cl	2.29386	-2.29705	-3.01871
Au	-0.64819	0.29918	2.60713
Cl	-2.07849	0.52479	4.35052
C	2.56849	0.19814	1.33309
C	2.86482	-1.00563	2.23464
C	3.46183	0.16966	0.10460
C	2.77024	1.49546	2.11915
H	2.20979	-1.02690	3.10901
H	2.75499	-1.94986	1.69644
H	3.89694	-0.93585	2.59131
H	3.29171	1.04247	-0.53351
H	4.50890	0.20684	0.42000
H	3.32777	-0.74548	-0.47787
H	3.80728	1.54641	2.46388
H	2.57340	2.37772	1.50515
H	2.12175	1.54080	2.99722
C	-1.96153	-1.92636	-1.18527
C	-3.18591	-1.92679	-2.10358
C	-1.01185	-3.06060	-1.57998
C	-2.37952	-2.06607	0.27311
H	-3.90336	-1.15301	-1.82567
H	-2.90481	-1.78545	-3.14841
H	-3.68273	-2.89651	-2.01126
H	-0.11219	-3.07844	-0.96246
H	-1.53738	-4.00937	-1.44017
H	-0.70161	-2.99245	-2.62317
H	-2.91861	-3.01045	0.38949
H	-1.51965	-2.09572	0.94531
H	-3.04095	-1.26067	0.59559
Cl	-2.34090	1.10896	-0.90081

R = Ph

P	0.80165	0.28984	0.48061
C	0.64000	-0.49824	-0.94161
P	-0.80753	-0.84933	-1.84301
O	-0.31472	-1.29134	-3.20514
Pb	2.02437	-1.20616	-2.78365
Cl	2.21394	-3.57493	-1.99678
Au	-0.71276	1.32594	1.71358
Cl	-2.28806	2.35791	2.97077
C	2.50379	0.25942	1.03087
C	2.95179	1.28780	1.86099
C	3.37307	-0.77460	0.67228
C	4.26811	1.30566	2.29163
H	2.26691	2.07357	2.16301
C	4.68352	-0.75552	1.11688
H	3.01005	-1.61496	0.08882
C	5.13353	0.28761	1.91726
H	4.61647	2.11174	2.92644
H	5.35217	-1.56576	0.85046
H	6.16057	0.29730	2.26389
C	-1.85083	-2.07466	-1.06906
C	-2.75171	-1.74558	-0.05739
C	-1.68615	-3.40068	-1.46540
C	-3.48921	-2.74551	0.55156
H	-2.88392	-0.71441	0.24988
C	-2.43212	-4.39363	-0.85025
H	-0.97700	-3.64875	-2.24571
C	-3.33007	-4.06723	0.15482
H	-4.19185	-2.49112	1.33610
H	-2.30807	-5.42510	-1.15803
H	-3.91159	-4.84689	0.63358
Cl	-1.96802	0.79818	-2.01895

R = Mes

P	0.71475	0.51227	1.03246
C	0.41194	-0.41769	-0.27401
P	-0.90420	-0.69213	-1.38184
O	-0.20422	-0.69308	-2.72587
Pb	2.05594	-0.88255	-1.95456
Cl	1.99400	-3.36822	-1.65113
Au	-0.57789	1.74075	2.34036
Cl	-1.86898	3.00515	3.70605
C	2.49268	0.45430	1.34735
C	3.26072	1.60893	1.11831
C	3.08507	-0.72010	1.84243
C	4.62666	1.55351	1.35781
C	4.45574	-0.71723	2.07168
C	5.24421	0.40074	1.82943
H	5.22453	2.44183	1.17781
H	4.91848	-1.61803	2.46301
C	-1.87405	-2.16770	-1.05323
C	-2.45239	-2.35306	0.22028
C	-2.06553	-3.13609	-2.06574
C	-3.18468	-3.50561	0.46451
C	-2.80985	-4.26715	-1.75356
C	-3.36866	-4.48155	-0.50272
H	-3.63118	-3.63578	1.44532
H	-2.96179	-5.00816	-2.53184
Cl	-2.23078	0.85310	-1.38284
C	2.64122	2.88465	0.63014
H	2.08349	2.74114	-0.29999

H	1.93643	3.29040	1.36266
H	3.40529	3.64046	0.44710
C	2.28897	-1.95450	2.14649
H	1.37750	-1.71817	2.70311
H	1.98623	-2.47679	1.23453
H	2.87768	-2.64712	2.74860
C	6.72336	0.36138	2.05753
H	7.24562	0.05106	1.14703
H	7.10961	1.34301	2.33692
H	6.98625	-0.34939	2.84250
C	-2.31516	-1.37725	1.34834
H	-3.08404	-1.56038	2.09916
H	-1.34542	-1.48387	1.84167
H	-2.40905	-0.33813	1.03216
C	-1.53348	-3.04660	-3.46670
H	-1.78430	-2.10387	-3.95102
H	-0.44570	-3.13366	-3.47931
H	-1.94657	-3.86310	-4.05956
C	-4.13419	-5.73176	-0.20418
H	-4.63464	-6.11503	-1.09496
H	-3.45959	-6.51552	0.15459
H	-4.88399	-5.56515	0.57088

R = Mes*

P	-0.90808	1.18045	0.07241
C	-0.17986	-0.24764	0.38936
P	1.39869	-0.98582	0.49086
Cl	-1.83137	-2.66424	-2.03183
O	1.16121	-2.36998	-0.07923
C	3.06830	-0.43992	0.02203
C	3.59307	-1.06324	-1.14897
C	3.97011	0.12932	0.95893
C	4.93407	-1.41342	-1.13829
C	5.30467	-0.25959	0.88401
C	5.79652	-1.10598	-0.09448
H	5.32421	-1.94759	-1.99486
H	5.98502	0.13141	1.62698
C	-2.69441	0.80389	0.10930
C	-3.42797	0.64720	-1.09502
C	-3.25405	0.37832	1.34065
C	-4.57450	-0.14091	-1.04897
C	-4.38253	-0.43482	1.28847
C	-5.03108	-0.75669	0.10581
H	-5.11402	-0.31029	-1.96558
H	-4.78329	-0.81466	2.21983
C	-3.08477	1.35252	-2.42070
C	-2.81494	0.81969	2.75461
C	-6.21572	-1.71720	0.11739
C	3.68656	1.27792	1.94877
C	2.85690	-1.26622	-2.48887
C	7.23074	-1.61989	-0.10628
C	-4.05969	1.37302	3.47450
H	-3.77132	1.74005	4.46263
H	-4.49459	2.20577	2.91688
H	-4.83699	0.62291	3.61927
C	-1.79195	1.95315	2.78985
H	-0.78668	1.63563	2.51054
H	-2.08669	2.79430	2.15736
H	-1.71594	2.32873	3.81382
C	-2.26543	-0.36353	3.55638
H	-2.97948	-1.18932	3.59897
H	-1.33200	-0.73195	3.12499
H	-2.05387	-0.05432	4.58381
C	-7.32324	-1.16424	1.02170
H	-8.17516	-1.84951	1.03259
H	-6.98549	-1.03943	2.05260
H	-7.67291	-0.19320	0.66280
C	-6.79727	-1.93693	-1.27664
H	-7.62338	-2.64941	-1.21690
H	-7.19257	-1.01202	-1.70424
H	-6.05458	-2.34721	-1.96499
C	-5.74456	-3.07285	0.65829
H	-4.96707	-3.49736	0.01781
H	-5.34412	-2.98760	1.67115
H	-6.58009	-3.77747	0.68884
C	-4.09319	1.02096	-3.52502
H	-5.11252	1.30990	-3.25869
H	-3.82021	1.58422	-4.41980
H	-4.08164	-0.03954	-3.78784
C	-3.16431	2.86757	-2.18958
H	-4.16610	3.15625	-1.86135
H	-2.45268	3.21197	-1.43606
H	-2.94155	3.40011	-3.11782
C	-1.71202	0.96572	-2.97671
H	-0.88167	1.32742	-2.36715
H	-1.61731	-0.11622	-3.07675
H	-1.58164	1.42361	-3.96122
C	2.26249	1.81413	1.91877
H	1.94944	2.03013	0.89467
H	1.53744	1.14942	2.38238
H	2.22421	2.76000	2.46389
C	4.57081	2.45364	1.48035
H	4.39416	3.31584	2.12846
H	5.63431	2.21630	1.51775
H	4.32206	2.74658	0.45789
C	4.07152	0.92676	3.38885
H	5.12285	0.64606	3.47525
H	3.91182	1.79858	4.02892
H	3.46956	0.10578	3.77792
C	1.50689	-0.55882	-2.57295
H	1.18410	-0.53627	-3.61645
H	0.71808	-1.07156	-2.02814
H	1.57668	0.47803	-2.23174
C	2.68860	-2.74977	-2.82707
H	2.05956	-3.24780	-2.09099
H	2.22002	-2.85220	-3.80961
H	3.65396	-3.26069	-2.86292
C	3.72661	-0.60643	-3.57880
H	3.88922	0.45300	-3.36587
H	4.70057	-1.08177	-3.69557
H	3.20829	-0.68358	-4.53778
C	8.01083	-1.18811	1.13249
H	8.12236	-0.10227	1.18774
H	7.53335	-1.53356	2.05286
H	9.01479	-1.61763	1.09828
C	7.95967	-1.08817	-1.34614
H	7.47939	-1.41302	-2.27113
H	7.98611	0.00418	-1.34686
H	8.99010	-1.45378	-1.35984
C	7.20479	-3.15255	-0.15024
H	6.70012	-3.56071	0.72871
H	6.68528	-3.52321	-1.03604
H	8.22529	-3.54464	-0.17097
Au	-0.04250	3.18851	-0.35134
Cl	0.85582	5.23378	-0.75970
Pb	-1.20566	-2.45986	0.39070
Cl	1.56182	-1.35197	2.51918

Coordination through π(C=P) bond

R= H

P	0.74223	-0.08108	0.95638
C	0.38133	0.10628	-0.71677
P	-1.15523	-0.23261	-1.48243
O	-0.99795	0.12839	-2.93782
Pb	1.41125	0.07018	-2.95643
Cl	1.47272	-2.41983	-2.92198
Au	0.57405	2.11127	0.01859
Cl	0.71885	4.35007	0.15667
Cl	-2.75907	0.66556	-0.66780
H	-1.47275	-1.58588	-1.28238
H	2.16828	-0.10220	0.86011

R = Me

P	0.76688	0.06467	0.86292
C	0.32686	0.23411	-0.78865
P	-1.22301	-0.21131	-1.48731
O	-1.06379	-0.01753	-2.98080
Pb	1.30744	0.11867	-3.03223
Cl	1.66132	-2.35609	-2.80575
C	-1.66673	-1.88812	-1.05758
H	-1.82796	-1.97531	0.01746
H	-2.57635	-2.15704	-1.59678
H	-0.84427	-2.53399	-1.37560
C	2.59741	-0.06510	0.84656
H	3.06103	0.36366	-0.04128
H	3.00155	0.40958	1.74303
H	2.83792	-1.13263	0.89439
Au	0.46773	2.25179	-0.07581
Cl	0.56243	4.49334	0.12336
Cl	-2.77786	0.89573	-0.81465

R = *t*-Bu

P	0.71903	-0.01283	0.70697
C	0.34737	0.28087	-0.94766
P	-1.10267	-0.28886	-1.79393
O	-0.72379	-0.27683	-3.26173
Pb	1.50581	0.49880	-3.10050
Cl	2.51948	-1.79933	-3.01646
Au	0.14814	2.20227	0.00019
Cl	-0.07023	4.38759	0.51903
C	2.57039	0.10846	0.96315
C	3.00762	-1.35503	1.11037
C	3.37650	0.78360	-0.13116
C	2.75098	0.84089	2.29404
H	2.44085	-1.87384	1.88888
H	2.89787	-1.90319	0.17116
H	4.06377	-1.38642	1.39673
H	3.01642	1.79378	-0.34381
H	4.42162	0.86818	0.18483
H	3.38021	0.18454	-1.04576
H	3.79837	0.77933	2.60504
H	2.48265	1.89699	2.21283
H	2.14218	0.39708	3.08693
C	-1.79147	-1.91680	-1.30015
C	-2.86753	-2.27744	-2.32711
C	-0.64906	-2.93405	-1.36955
C	-2.38257	-1.86951	0.10532
H	-3.70170	-1.57384	-2.30701
H	-2.46135	-2.30781	-3.33895
H	-3.25723	-3.26947	-2.08211
H	0.11767	-2.74268	-0.61696
H	-1.06479	-3.92715	-1.17740
H	-0.16611	-2.94916	-2.34758
H	-2.73956	-2.87058	0.36366
H	-1.64269	-1.57865	0.85438
H	-3.22827	-1.18361	0.16884
Cl	-2.63770	1.02066	-1.51926

R = Ph

P	0.73086	0.29284	0.83340
C	0.20032	0.29381	-0.79925
P	-1.39627	-0.12593	-1.39179
O	-1.35884	0.12832	-2.88308
Pb	0.99600	0.42763	-3.12616
Cl	1.49082	-2.01610	-3.43218
Au	0.40854	2.38842	-0.29930
Cl	0.52931	4.63806	-0.37258
C	-1.82419	-1.81759	-1.00008
C	-1.84406	-2.75127	-2.03163
C	-2.10295	-2.20611	0.30958
C	-2.14814	-4.07467	-1.74914
H	-1.61630	-2.44110	-3.04342
C	-2.40210	-3.52842	0.58216
H	-2.09395	-1.47650	1.11205
C	-2.42510	-4.46171	-0.44769
H	-2.16300	-4.80374	-2.55049
H	-2.62136	-3.83319	1.59864
H	-2.66087	-5.49732	-0.23050
C	2.53744	0.14277	0.74019
C	3.18694	-0.61450	-0.23601
C	3.28559	0.73859	1.75799
C	4.56656	-0.74279	-0.20963
H	2.61327	-1.13406	-0.99691
C	4.66543	0.62334	1.76868
H	2.78459	1.30798	2.53477
C	5.30666	-0.11630	0.78407
H	5.06336	-1.34054	-0.96529
H	5.24177	1.10630	2.54920
H	6.38607	-0.21510	0.79877
Cl	-2.87124	0.97364	-0.53923

R = Mes

P	0.80797	0.34298	1.21908
C	0.15880	0.24399	-0.37120
P	-1.40336	-0.12757	-1.08958
O	-1.27926	0.42861	-2.49377
Pb	1.07969	0.69376	-2.61364
Cl	1.52006	-1.65926	-3.39769
Au	0.16439	2.37056	0.10949
Cl	0.02146	4.62394	0.01550
C	-1.84658	-1.87106	-1.02194
C	-2.14902	-2.57851	-2.21084
C	-1.91193	-2.53038	0.22301
C	-2.43562	-3.93354	-2.10845
C	-2.20756	-3.88673	0.25327
C	-2.45253	-4.61409	-0.89955
H	-2.66784	-4.47484	-3.02003
H	-2.25968	-4.38251	1.21785
C	2.61622	0.25522	0.96217
C	3.17281	-0.94518	0.47692
C	3.45565	1.29241	1.40172
C	4.55161	-1.03056	0.33966
C	4.82948	1.15667	1.23914
C	5.39714	0.01634	0.68856
H	4.97910	-1.95715	-0.03193
H	5.47465	1.96596	1.56845
Cl	-2.97584	0.85079	-0.22040
C	2.92696	2.53892	2.04633
H	2.62552	3.28808	1.30796

H	2.05237	2.33911	2.67198
H	3.69168	2.99238	2.67898
C	2.34097	-2.15824	0.17483
H	1.59505	-2.33926	0.95453
H	1.80957	-2.07778	-0.77560
H	2.97707	-3.04242	0.11857
C	6.87702	-0.09241	0.48934
H	7.22729	-1.11324	0.65358
H	7.14954	0.18330	-0.53429
H	7.42042	0.57160	1.16343
C	-1.69270	-1.86437	1.54838
H	-0.64874	-1.94527	1.86504
H	-1.95481	-0.80819	1.55806
H	-2.29582	-2.35903	2.31150
C	-2.73053	-6.08314	-0.84742
H	-3.46876	-6.37513	-1.59661
H	-1.81794	-6.65165	-1.05198
H	-3.09479	-6.38675	0.13511
C	-2.21447	-1.98164	-3.58666
H	-2.82776	-1.08242	-3.62123
H	-1.22146	-1.70977	-3.94718
H	-2.63463	-2.71625	-4.27423

R = Mes*

P	0.65775	0.07201	1.16926
C	0.38139	0.08183	-0.54173
P	-1.03841	-0.15397	-1.62623
O	-0.39666	-0.06326	-2.99702
Pb	1.82453	0.62517	-2.49737
Cl	2.94796	-1.45571	-3.38137
Au	0.40554	2.18946	0.01289
Cl	0.32671	4.45742	-0.09189
C	2.50843	-0.02254	1.26483
C	3.18948	-1.15843	0.74510
C	3.27766	1.09134	1.67877
C	4.49266	-0.99151	0.30095
C	4.58662	1.18823	1.19870
C	5.18891	0.20641	0.43222
H	4.99524	-1.84040	-0.14334
H	5.15449	2.07093	1.45655
C	2.90620	2.12121	2.76709
C	2.70869	-2.61385	0.90749
C	6.57664	0.36010	-0.18003
C	3.86137	1.82682	3.94224
H	3.72452	0.80759	4.31271
H	4.90784	1.94289	3.65752
H	3.65466	2.51968	4.76252
C	3.12773	3.56588	2.31072
H	2.47954	3.83261	1.47527
H	2.90044	4.24615	3.13609
H	4.16141	3.75124	2.01434
C	1.49821	2.00674	3.34292
H	1.42452	2.65991	4.21663
H	0.72234	2.33406	2.64793
H	1.26870	0.99128	3.67491
C	3.04708	-3.49291	-0.29881
H	2.64359	-3.08470	-1.22423
H	4.12307	-3.61509	-0.43209
H	2.63148	-4.49260	-0.14609
C	3.48118	-3.14929	2.12927
H	4.55957	-3.09967	1.96905
H	3.24673	-2.57242	3.02761
H	3.20874	-4.19327	2.31082
C	1.22856	-2.79031	1.22489
H	0.92295	-2.22097	2.10985
H	0.58431	-2.52410	0.38865
H	1.03989	-3.84238	1.45688
C	7.20550	1.71277	0.14272
H	6.59315	2.54243	-0.21919
H	7.36394	1.84407	1.21639
H	8.18079	1.78604	-0.34429
C	6.45511	0.23291	-1.70347
H	7.44364	0.29466	-2.16728
H	5.99795	-0.71319	-1.99936
H	5.84102	1.03903	-2.11368
C	7.50752	-0.73830	0.34715
H	7.59631	-0.68806	1.43530
H	7.15362	-1.73641	0.08266
H	8.50624	-0.61863	-0.08152
C	-2.34088	-1.45472	-1.66349
C	-2.31458	-2.34407	-2.78198
C	-3.57781	-1.26465	-0.98908
C	-3.52533	-2.66038	-3.37887
C	-1.09127	-3.11513	-3.31442
C	-4.74535	-1.65180	-1.63937
C	-3.78337	-0.81812	0.47065
C	-4.75674	-2.25673	-2.88321
H	-3.50065	-3.27162	-4.27110
C	-0.61683	-2.60595	-4.67839
C	-1.49911	-4.59293	-3.49232
C	0.04236	-3.15217	-2.29846
H	-5.68514	-1.46239	-1.14080
C	-2.50189	-0.47352	1.20657
C	-4.37546	-2.04068	1.20099
C	-4.76285	0.35176	0.60565
C	-6.03560	-2.56168	-3.65203
H	0.20096	-3.23456	-5.03998
H	-1.42950	-2.65690	-5.40813
H	-0.26524	-1.57975	-4.62701
H	-2.18632	-4.75045	-4.32415
H	-0.60042	-5.17693	-3.70608
H	-1.95843	-4.99502	-2.58611
H	0.46583	-2.17691	-2.07134
H	-0.29731	-3.61693	-1.36950
H	0.86674	-3.74755	-2.69420
H	-1.76207	-1.27081	1.11087
H	-2.06500	0.46648	0.87232
H	-2.71177	-0.35502	2.27221
H	-4.51451	-1.79729	2.25790
H	-5.34214	-2.33912	0.79404
H	-3.70219	-2.89888	1.13463
H	-5.74178	0.12376	0.18094
H	-4.91077	0.57872	1.66485
H	-4.38228	1.24931	0.11850
C	-7.27623	-2.01499	-2.95095
C	-6.19548	-4.07896	-3.80602
C	-5.94159	-1.91700	-5.04023
H	-7.43724	-2.48612	-1.97791
H	-7.21564	-0.93361	-2.80533
H	-8.15895	-2.21857	-3.56161
H	-5.35949	-4.52274	-4.35003
H	-6.25890	-4.56722	-2.83042
H	-7.11106	-4.30458	-4.35960
H	-5.83681	-0.83233	-4.96164
H	-5.08729	-2.29517	-5.60506
H	-6.84612	-2.13205	-5.61563
Cl	-2.24009	1.51804	-1.55655

[R-P=C(Pb(II)Cl)-P(O)MeR](AuCl)

Coordination through Pb(II) atom

R= H

P	0.85769	0.09359	0.83241
C	0.44611	-0.08511	-0.75990
P	-1.17105	-0.18391	-1.46739
O	-0.92222	0.08841	-2.94477
Pb	1.42477	-0.06259	-2.84848
Cl	1.47545	-2.49625	-3.08378
C	-2.37059	0.92030	-0.72982
H	-3.32836	0.80688	-1.24121
H	-2.49021	0.67826	0.32887
H	-2.02240	1.94876	-0.83415
Au	2.77940	1.58768	-4.31779
Cl	3.95172	3.08138	-5.53379
H	2.27751	0.17652	0.68858
H	-1.68596	-1.47717	-1.24145

R = Me

P	0.84761	0.02686	0.84262
C	0.44498	-0.10867	-0.75169
P	-1.17602	-0.23325	-1.45286
O	-0.91479	0.04780	-2.93504
Pb	1.40234	-0.03306	-2.83897
Cl	1.55310	-2.47484	-3.11098
C	-2.33396	0.94289	-0.74881
H	-3.29901	0.84780	-1.25070
H	-2.45724	0.75686	0.32005
H	-1.94929	1.95210	-0.89910
C	-1.85159	-1.87381	-1.19469
H	-2.01560	-2.05801	-0.13134
H	-2.79542	-1.96539	-1.73653
H	-1.13769	-2.59988	-1.58798
C	2.67699	0.14378	0.88712
H	3.13062	0.24121	-0.09944
H	2.96327	0.99560	1.50945
H	3.06605	-0.75483	1.37546
Au	2.72067	1.60801	-4.35130
Cl	3.87372	3.09986	-5.59276

R = *t*-Bu

P	0.82827	-0.23272	0.76305
C	0.50188	-0.16410	-0.85383
P	-1.09120	-0.35202	-1.62202
O	-0.77236	-0.10574	-3.10226
Pb	1.49883	0.30607	-2.87544
Cl	2.33772	-1.94962	-3.39389
C	-2.21840	0.90539	-0.99703
H	-3.17452	0.84913	-1.51939
H	-2.37210	0.79040	0.07662
H	-1.76910	1.88067	-1.18991
Au	2.38498	2.39208	-4.13846
Cl	3.13874	4.27030	-5.14437
C	2.65524	0.05975	1.05205
C	3.16486	-1.22836	1.70688
C	3.47610	0.38347	-0.18036
C	2.72503	1.21044	2.06017
H	2.57258	-1.49942	2.58528
H	3.14644	-2.06888	1.00901
H	4.19926	-1.08526	2.03558
H	3.14175	1.30821	-0.66089
H	4.52268	0.53959	0.10104
H	3.46451	-0.43857	-0.90260
H	3.76067	1.34930	2.38610
H	2.38186	2.15189	1.62382
H	2.12084	1.00788	2.94877
C	-1.86428	-1.99951	-1.38114
C	-3.08667	-2.06587	-2.30120
C	-0.85357	-3.06900	-1.79422
C	-2.27732	-2.19307	0.07559
H	-3.86348	-1.35454	-2.01132
H	-2.81410	-1.87921	-3.34133
H	-3.52021	-3.06794	-2.23713
H	0.03040	-3.06385	-1.15450
H	-1.32800	-4.05091	-1.71012
H	-0.52184	-2.93853	-2.82521
H	-2.67900	-3.20304	0.19732
H	-1.43142	-2.09062	0.76014
H	-3.05671	-1.49175	0.38156

R = Ph

P	0.88868	0.16471	0.80502
C	0.45203	-0.18061	-0.75211
P	-1.16127	-0.33110	-1.45832
O	-0.88314	-0.17885	-2.95337
Pb	1.45867	-0.15247	-2.80339
Cl	1.76047	-2.58134	-3.09124
C	-2.28570	0.93145	-0.85307
H	-3.24377	0.83143	-1.36616
H	-2.43643	0.82649	0.22288
H	-1.85707	1.91215	-1.06374
Au	2.69528	1.52027	-4.35170
Cl	3.77110	3.02849	-5.64489
C	2.69889	0.19951	0.77362
C	3.33719	1.11394	1.61357
C	3.47171	-0.65473	-0.01864
C	4.71934	1.21508	1.61690
H	2.74625	1.76073	2.25469
C	4.85347	-0.56059	-0.00099
H	2.99441	-1.42617	-0.61601
C	5.47714	0.38151	0.80703
H	5.20630	1.94346	2.25482
H	5.44564	-1.23029	-0.61383
H	6.55836	0.45631	0.81428
C	-1.90006	-1.92481	-1.08970
C	-2.05704	-2.84168	-2.12456
C	-2.30181	-2.25485	0.20390
C	-2.62446	-4.08044	-1.86519
H	-1.72866	-2.58045	-3.12328
C	-2.86677	-3.49266	0.45597
H	-2.16383	-1.55409	1.02124
C	-3.02953	-4.40441	-0.57974
H	-2.74437	-4.79518	-2.67077
H	-3.17761	-3.74990	1.46177
H	-3.47026	-5.37435	-0.37958

R = Mes

P	1.03958	-0.19707	1.30240
C	0.43492	-0.31605	-0.23267
P	-1.15241	-0.33163	-1.03856
O	-0.75640	0.14855	-2.43555
Pb	1.57926	0.13591	-2.13740
Cl	1.94637	-2.17977	-2.91675
Au	2.94056	2.08047	-3.18201
Cl	4.14236	3.82808	-3.97112
C	2.82088	0.04863	0.95857
C	3.34295	1.35276	0.93850
C	3.65639	-1.05824	0.74642
C	4.69022	1.52785	0.65592
C	5.00032	-0.83062	0.46958
C	5.53373	0.45016	0.40703

H	5.09145	2.53635	0.62305
H	5.64929	-1.68461	0.30047
C	-1.95090	-1.95549	-1.02678
C	-2.19949	-2.65583	-2.22994
C	-2.33140	-2.52830	0.20386
C	-2.79945	-3.90765	-2.14903
C	-2.91629	-3.78696	0.21761
C	-3.15532	-4.49945	-0.94674
H	-2.99059	-4.44146	-3.07458
H	-3.19558	-4.21888	1.17393
C	2.47151	2.54875	1.18649
H	1.78109	2.38428	2.01983
H	3.07490	3.42732	1.41667
H	1.86480	2.79186	0.30818
C	3.13279	-2.46256	0.80512
H	2.44759	-2.60378	1.64679
H	2.58571	-2.72183	-0.10622
H	3.95152	-3.17465	0.91551
C	6.97477	0.67437	0.06865
H	7.07305	1.06950	-0.94673
H	7.43190	1.40183	0.74309
H	7.54677	-0.25263	0.12361
C	-2.18421	-1.84302	1.53205
H	-1.30666	-1.20079	1.60343
H	-3.06460	-1.23172	1.75363
H	-2.10242	-2.58372	2.32847
C	-1.86924	-2.15857	-3.60819
H	-2.26101	-1.16057	-3.79840
H	-0.79029	-2.10886	-3.76361
H	-2.28656	-2.84413	-4.34630
C	-3.75544	-5.86975	-0.90885
H	-2.96976	-6.63168	-0.90112
H	-4.36306	-6.01565	-0.01435
H	-4.37896	-6.05516	-1.78521
C	-2.27880	0.87997	-0.32630
H	-3.19540	0.88677	-0.91965
H	-2.51801	0.66952	0.71403
H	-1.79861	1.85710	-0.40014

R = Mes*

P	-0.00912	2.38376	1.11188
C	0.43179	0.88380	0.56155
P	1.91384	-0.10845	0.42728
Cl	-1.17175	0.26838	-2.76657
O	1.48045	-1.08783	-0.67288
C	2.01653	-1.12690	1.92109
H	2.83921	-1.83047	1.78188
H	2.15182	-0.58074	2.84883
H	1.08432	-1.69385	1.97194
C	3.63999	0.42184	0.10830
C	4.16508	0.15850	-1.19053
C	4.55650	0.55297	1.18523
C	5.45904	-0.33574	-1.27427
C	5.84668	0.05866	1.01458
C	6.29544	-0.48636	-0.17669
H	5.83307	-0.60776	-2.25264
H	6.52744	0.11618	1.85252
C	-1.82159	2.34728	0.78136
C	-2.33193	3.03875	-0.34038
C	-2.67399	1.50344	1.53998
C	-3.58132	2.65715	-0.82424
C	-3.89746	1.15443	0.97733
C	-4.34842	1.66508	-0.23387
H	-3.95945	3.13964	-1.71093
H	-4.53834	0.47022	1.51631
C	-1.61172	4.23259	-0.99770
C	-2.40181	1.06242	2.99767
C	-5.66252	1.17029	-0.82924
C	4.33728	1.36376	2.47964
C	3.48411	0.50294	-2.52912
C	7.66481	-1.13551	-0.33415
C	-3.72843	0.97985	3.77017
H	-3.51209	0.83497	4.83126
H	-4.31506	1.89508	3.66373
H	-4.34462	0.13606	3.45671
C	-1.54901	2.09076	3.74314
H	-0.51844	2.14670	3.37441
H	-1.98129	3.09205	3.68055
H	-1.47876	1.81185	4.79787
C	-1.73464	-0.31216	3.09627
H	-2.31467	-1.07627	2.57219
H	-0.72569	-0.29587	2.68343
H	-1.66148	-0.61490	4.14492
C	-6.80945	1.43036	0.15445
H	-7.75293	1.07669	-0.26999
H	-6.65964	0.91108	1.10307
H	-6.91055	2.49797	0.36603
C	-5.99585	1.86238	-2.14824
H	-6.92869	1.45508	-2.54503
H	-6.13549	2.93940	-2.02208
H	-5.21845	1.69866	-2.89847
C	-5.54801	-0.33620	-1.09271
H	-4.74686	-0.54809	-1.80505
H	-5.34131	-0.89801	-0.17946
H	-6.48341	-0.71701	-1.51164
C	-2.48671	4.90914	-2.05617
H	-3.44532	5.23959	-1.64950
H	-1.96365	5.79301	-2.42812
H	-2.67511	4.25768	-2.91256
C	-1.31967	5.28861	0.07758
H	-2.24290	5.61686	0.56141
H	-0.64616	4.91761	0.85322
H	-0.84458	6.16157	-0.37869
C	-0.31017	3.83379	-1.69857
H	0.46700	3.52238	-0.99616
H	-0.47095	3.01307	-2.39920
H	0.08694	4.69322	-2.24673
C	2.98316	2.05385	2.56851
H	2.77842	2.63941	1.67013
H	2.14721	1.37658	2.73224
H	2.98581	2.74716	3.41316
C	5.36946	2.51005	2.43786
H	5.23411	3.15185	3.31277
H	6.39654	2.14499	2.44525
H	5.23493	3.12157	1.54271
C	4.59281	0.54502	3.74885
H	5.60471	0.13672	3.76979
H	4.47920	1.18468	4.62818
H	3.90093	-0.29151	3.85285
C	2.30631	1.45626	-2.35868
H	1.95887	1.78079	-3.34209
H	1.44704	1.00192	-1.87541
H	2.60293	2.34630	-1.79729
C	3.06838	-0.74646	-3.31041
H	2.31949	-1.32025	-2.76961
H	2.65255	-0.45306	-4.27809
H	3.93090	-1.39125	-3.49901
C	4.50700	1.28010	-3.38284
H	4.89747	2.14826	-2.84600
H	5.35142	0.66906	-3.70292
H	4.00726	1.63698	-4.28675
C	8.41971	-1.22238	0.98960

H	8.64618	-0.23382	1.39735
H	7.85882	-1.78548	1.73987
H	9.37122	-1.73584	0.83324
C	8.51130	-0.32241	-1.32088
H	8.04741	-0.27059	-2.30769
H	8.65570	0.70006	-0.96330
H	9.49549	-0.78388	-1.43918
C	7.47791	-2.55722	-0.87743
H	6.89006	-3.16658	-0.18673
H	6.96619	-2.55781	-1.84173
H	8.45028	-3.03837	-1.01320
Au	-2.27276	-2.77386	0.05353
Cl	-3.53420	-4.55227	0.67126
Pb	-0.83261	-0.68773	-0.51729

Coordination through O atom

R= H

P	0.85986	0.08493	0.98463
C	0.59543	-0.10644	-0.63856
P	-0.95630	0.02527	-1.42480
O	-0.55893	0.35200	-2.88578
Pb	1.83898	-0.35663	-2.66779
Cl	1.21591	-2.75110	-2.80215
C	-2.10282	1.21877	-0.74733
H	-3.03192	1.20537	-1.32023
H	-2.30605	0.96183	0.29555
H	-1.65697	2.21308	-0.79786
Au	-1.99295	0.72093	-4.36866
Cl	-3.51824	1.14257	-5.92985
H	2.28374	-0.07360	1.00445
H	-1.62301	-1.21290	-1.37057

R = Me

P	0.82152	-0.01704	0.97640
C	0.58239	-0.13242	-0.65372
P	-0.99077	-0.02081	-1.40749
O	-0.60494	0.29779	-2.88443
Pb	1.79005	-0.23646	-2.71627
Cl	1.42762	-2.69442	-2.92014
C	-2.05581	1.26316	-0.75037
H	-2.99817	1.28212	-1.30156
H	-2.24787	1.06821	0.30724
H	-1.55712	2.22738	-0.85419
C	-1.87864	-1.57239	-1.31909
H	-2.14260	-1.78941	-0.28215
H	-2.78226	-1.51036	-1.92967
H	-1.22715	-2.35876	-1.70545
C	2.63251	-0.20315	1.22503
H	3.19907	-0.19442	0.29346
H	2.98879	0.59431	1.88243
H	2.81281	-1.14968	1.74386
Au	-2.03975	0.66021	-4.36165
Cl	-3.57209	1.07126	-5.92104

R = *t*-Bu

P	0.89950	-0.50119	0.86098
C	0.49915	-0.10952	-0.69428
P	-1.15859	-0.06787	-1.27044
O	-0.98418	0.53563	-2.69969
Pb	1.47550	0.79210	-2.69236
Cl	2.09225	-1.43093	-3.65112
C	-2.17780	1.02673	-0.27113
H	-3.18823	1.09653	-0.67607
H	-2.20584	0.66770	0.75906
H	-1.72285	2.01817	-0.28936
Au	-2.38235	1.65966	-3.77094
Cl	-3.84124	2.88800	-4.91745
C	2.76104	-0.39342	1.05627
C	3.21714	-1.82499	1.35563
C	3.51024	0.15373	-0.14289
C	2.99861	0.48786	2.28480
H	2.66947	-2.25591	2.19840
H	3.08159	-2.47836	0.49042
H	4.28056	-1.82537	1.61684
H	3.19772	1.17756	-0.37585
H	4.58413	0.19321	0.06876
H	3.38360	-0.48795	-1.02059
H	4.06252	0.48544	2.54289
H	2.69905	1.52362	2.10386
H	2.44451	0.12455	3.15455
C	-1.97808	-1.70842	-1.37911
C	-3.30639	-1.54918	-2.12262
C	-1.06235	-2.65356	-2.15661
C	-2.22398	-2.24832	0.02891
H	-4.00560	-0.89529	-1.59720
H	-3.16588	-1.16028	-3.13246
H	-3.77634	-2.53358	-2.20402
H	-0.10820	-2.80869	-1.65104
H	-1.56134	-3.62223	-2.25047
H	-0.85118	-2.28326	-3.16072
H	-2.64408	-3.25464	-0.05276
H	-1.30199	-2.32145	0.61099
H	-2.93871	-1.63907	0.58652

R = Ph

P	0.78595	0.22950	0.92748
C	0.53483	-0.23609	-0.63963
P	-0.96754	-0.17812	-1.52032
O	-0.46664	-0.10762	-2.98932
Pb	1.97399	-0.48629	-2.51000
Cl	1.81416	-2.97418	-2.63851
C	-1.99637	1.24151	-1.13522
H	-2.89535	1.22251	-1.75370
H	-2.27300	1.22612	-0.07910
H	-1.42799	2.14934	-1.34243
Au	-1.66563	0.36093	-4.63665
Cl	-2.91849	0.87902	-6.40135
C	2.56580	-0.05181	1.16858
C	3.25955	-1.10760	0.56826
C	3.25529	0.81838	2.01406
C	4.62153	-1.25844	0.77787
H	2.72080	-1.84410	-0.02203
C	4.61994	0.67288	2.21121
H	2.72044	1.62292	2.50961
C	5.30444	-0.36228	1.59031
H	5.14768	-2.08708	0.31759
H	5.14980	1.36532	2.85527
H	6.36941	-0.48194	1.75294
C	-1.95230	-1.65348	-1.26197
C	-2.65030	-1.83055	-0.06762
C	-1.99695	-2.63423	-2.24824
C	-3.39764	-2.97783	0.13100
H	-2.60647	-1.08042	0.71523
C	-2.74885	-3.78085	-2.04246
H	-1.44429	-2.50184	-3.16995
C	-3.44834	-3.95177	-0.85822
H	-3.94112	-3.11398	1.05857
H	-2.78482	-4.54201	-2.81259
H	-4.03525	-4.84952	-0.70141

R = Mes

P	0.73022	-0.16074	1.35667
C	0.38840	-0.34502	-0.24874
P	-1.05194	-0.22488	-1.23499
O	-0.41584	0.11953	-2.61103
Pb	2.03072	-0.15755	-1.92783
Cl	2.16365	-2.61016	-2.41760
Au	-1.17489	1.38707	-4.08756
Cl	-1.93250	2.75800	-5.66954
C	2.56828	-0.20114	1.33176
C	3.25024	-1.42949	1.35996
C	3.28470	1.00691	1.34681
C	4.64022	-1.42017	1.35787
C	4.67408	0.96474	1.34328
C	5.37071	-0.23777	1.34047
H	5.16731	-2.36948	1.38020
H	5.22697	1.89957	1.35402
C	-2.02221	-1.75238	-1.27841
C	-2.69911	-2.14450	-0.10419
C	-2.10520	-2.56273	-2.43501
C	-3.43567	-3.32103	-0.10340
C	-2.86717	-3.72386	-2.36974
C	-3.53999	-4.12556	-1.22628
H	-3.94094	-3.61477	0.81162
H	-2.92509	-4.34374	-3.25869
C	2.57956	2.33221	1.36483
H	2.10069	2.55278	0.40554
H	1.79060	2.35831	2.12283
H	3.27843	3.14163	1.57864
C	2.51290	-2.73446	1.39496
H	1.68084	-2.70723	2.10514
H	2.09541	-2.97892	0.41380
H	3.17979	-3.54668	1.68668
C	6.86804	-0.26191	1.31068
H	7.23293	-0.39845	0.28794
H	7.28976	0.67080	1.68835
H	7.26409	-1.08541	1.90789
C	-2.70417	-1.35481	1.17375
H	-1.76144	-0.84756	1.38154
H	-3.49791	-0.60196	1.16619
H	-2.90149	-2.01713	2.01741
C	-1.43114	-2.28230	-3.74613
H	-1.75220	-1.33430	-4.18159
H	-0.34631	-2.24585	-3.64391
H	-1.67549	-3.07522	-4.45298
C	-4.36280	-5.37529	-1.21470
H	-4.51101	-5.74661	-0.19962
H	-5.35106	-5.18836	-1.64617
H	-3.89468	-6.16386	-1.80681
C	-2.11589	1.15713	-0.78244
H	-2.98843	1.17555	-1.43802
H	-2.43121	1.10155	0.25739
H	-1.53963	2.07127	-0.93702

R = Mes*

P	1.71145	2.29999	-0.39784
C	0.79787	0.92201	-0.35122
P	-0.91606	0.56987	-0.48255
C	3.40643	1.55273	-0.33699
C	4.14538	1.60142	0.87372
C	3.88086	0.76410	-1.40794
C	5.16053	0.67292	1.05191
C	4.87850	-0.17979	-1.13576
C	5.49760	-0.28213	0.09733
H	5.70169	0.67002	1.98653
H	5.19920	-0.83195	-1.93647
C	3.51492	0.94860	-2.89916
C	3.90689	2.66530	1.96150
C	6.55868	-1.33209	0.41058
C	2.56771	2.47657	2.68010
H	1.71282	2.67775	2.03046
H	2.46116	1.45861	3.05859
H	2.50162	3.17549	3.51914
C	3.96732	4.06024	1.32429
H	4.93375	4.22591	0.84149
H	3.18532	4.21157	0.57741
H	3.83602	4.82511	2.09470
C	4.99242	2.62541	3.04012
H	4.95912	1.70314	3.62469
H	5.99505	2.74014	2.62094
H	4.82691	3.45388	3.73240
C	2.73148	-0.24575	-3.44850
H	1.73767	-0.30282	-2.99805
H	2.59621	-0.14587	-4.52922
H	3.25033	-1.18953	-3.26473
C	2.73875	2.22883	-3.20461
H	1.70340	2.19320	-2.86224
H	3.21848	3.10809	-2.76727
H	2.70469	2.37569	-4.28771
C	4.83080	1.07177	-3.69127
H	5.44372	1.88919	-3.30446
H	5.42893	0.16110	-3.66438
H	4.60438	1.28236	-4.73960
C	6.75547	-2.31658	-0.73908
H	7.12312	-1.82419	-1.64323
H	7.49596	-3.06697	-0.45273
H	5.83020	-2.84466	-0.98434
C	6.13663	-2.12307	1.65438
H	6.02382	-1.47782	2.52727
H	5.18112	-2.62856	1.49826
H	6.89127	-2.87837	1.89059
C	7.89757	-0.63581	0.68315
H	7.83061	0.05160	1.52876
H	8.66621	-1.37774	0.91664
H	8.22807	-0.06561	-0.18874
C	-2.37677	1.43020	0.20634
C	-2.95029	0.88412	1.39032
C	-3.21320	2.19173	-0.65087
C	-4.33108	0.76683	1.44091
C	-4.58984	2.03440	-0.52845
C	-5.17425	1.23461	0.44157
H	-4.76695	0.28061	2.30439
H	-5.22714	2.56816	-1.22027
C	-2.18835	0.55736	2.68841
C	-2.24444	-0.92551	3.06086
C	-2.87576	1.36213	3.81077
H	-1.68514	-1.53643	2.35409
H	-1.80663	-1.07007	4.05222
H	-3.27233	-1.29373	3.08975
H	-2.90052	2.42887	3.57440
H	-3.89759	1.03378	4.00264
H	-2.30915	1.23160	4.73628
C	-2.74926	3.32936	-1.58321
C	-3.43292	4.60892	-1.05754
C	-3.18309	3.13067	-3.03837
H	-3.17308	4.78750	-0.01167
H	-3.09492	5.46726	-1.64481
H	-4.51932	4.55945	-1.13126
H	-2.72083	2.25663	-3.49707
H	-4.26488	3.01068	-3.12049
H	-2.90313	4.00688	-3.62953
C	-6.66698	0.93973	0.49275

C	-7.27346	1.56800	1.75320
C	-6.86600	-0.58063	0.53340
H	-7.14510	2.65353	1.75337
H	-8.34420	1.35142	1.80034
H	-6.81471	1.17445	2.66242
H	-6.42710	-1.06604	-0.34094
H	-6.40543	-1.02672	1.41669
H	-7.93370	-0.81526	0.55877
Cl	2.10615	-1.32336	2.31658
O	-0.94864	-0.88372	0.10137
Au	-2.49941	-2.21219	-0.36766
Cl	-4.12927	-3.64637	-0.88708
C	-1.25494	3.62207	-1.51089
H	-0.92946	3.77314	-0.47951
H	-0.62573	2.85072	-1.95005
H	-1.04005	4.54190	-2.06041
C	-0.74303	1.03635	2.67059
H	-0.10122	0.45201	2.01602
H	-0.67934	2.08995	2.38635
H	-0.31704	0.93268	3.67117
C	-7.40473	1.49059	-0.72471
H	-7.36076	2.58202	-0.77365
H	-7.00391	1.08447	-1.65677
H	-8.45890	1.20983	-0.66801
Pb	1.54587	-1.33492	-0.12610
C	-1.25513	0.33087	-2.24759
H	-2.30621	0.07068	-2.37970
H	-0.99511	1.18265	-2.86914
H	-0.65167	-0.52688	-2.55225

Coordination through P(sp^2) atom

R= H

P	0.76399	0.09517	0.85531
C	0.47319	-0.19967	-0.72261
P	-1.06986	-0.19935	-1.56142
O	-0.69607	-0.06128	-3.03118
Pb	1.61655	-0.53787	-2.85142
Cl	1.24869	-2.99296	-2.70670
C	-2.24150	1.04339	-1.02687
H	-3.14550	0.96394	-1.63396
H	-2.49238	0.89062	0.02571
H	-1.80313	2.03374	-1.15697
Au	-0.55091	0.57656	2.56026
Cl	-1.93823	1.08835	4.26914
H	2.16257	0.01745	1.03793
H	-1.69829	-1.43073	-1.28545

R = Me

P	0.81955	0.11719	0.81681
C	0.47446	-0.15413	-0.75173
P	-1.12108	-0.25044	-1.49222
O	-0.81850	-0.23797	-2.99267
Pb	1.49943	-0.51829	-2.92136
Cl	1.40107	-3.00131	-2.62107
C	-2.20126	1.11105	-1.04429
H	-3.13985	1.01751	-1.59473
H	-2.40333	1.10074	0.02902
H	-1.72121	2.05260	-1.31321
C	-1.93413	-1.76738	-0.99133
H	-2.14219	-1.75191	0.08039
H	-2.86750	-1.87541	-1.54813
H	-1.27134	-2.60154	-1.22918
C	2.60176	0.09902	1.13825
H	3.16354	-0.08661	0.22357
H	2.89730	1.05422	1.57815
H	2.81713	-0.68358	1.86975
Au	-0.55985	0.48205	2.51030
Cl	-2.01493	0.85764	4.20550

R = *t*-Bu

P	0.76293	0.08924	0.91570
C	0.45849	-0.15373	-0.66906
P	-1.10288	-0.29345	-1.48277
O	-0.74198	-0.12700	-2.96429
Pb	1.58299	0.10481	-2.81009
Cl	2.29063	-2.28738	-3.04513
C	-2.22056	1.02046	-0.96604
H	-3.14729	0.97010	-1.53940
H	-2.43838	0.95481	0.10128
H	-1.73217	1.97457	-1.16937
Au	-0.67199	0.32837	2.59753
Cl	-2.16827	0.61143	4.28053
C	2.56444	0.18956	1.34490
C	2.84992	-1.00926	2.25569
C	3.45828	0.14434	0.11727
C	2.77526	1.49048	2.12159
H	2.19198	-1.01960	3.12818
H	2.73361	-1.95629	1.72383
H	3.88156	-0.94579	2.61510
H	3.29873	1.01543	-0.52550
H	4.50565	0.16913	0.43323
H	3.31140	-0.77009	-0.46293
H	3.81066	1.53483	2.47235
H	2.59063	2.36937	1.49896
H	2.12219	1.54971	2.99563
C	-1.93814	-1.90771	-1.20648
C	-3.13687	-1.95450	-2.15926
C	-0.95923	-3.02564	-1.56220
C	-2.40180	-2.05470	0.23887
H	-3.90348	-1.22189	-1.89685
H	-2.83307	-1.78845	-3.19429
H	-3.59559	-2.94518	-2.09366
H	-0.08312	-3.02426	-0.91157
H	-1.46814	-3.98672	-1.44420
H	-0.60812	-2.95015	-2.59191
H	-2.88543	-3.02894	0.35517
H	-1.56855	-2.01871	0.94388
H	-3.12788	-1.29282	0.52944

R = Ph

P	0.77892	0.33462	0.64238
C	0.55538	-0.38892	-0.80227
P	-0.93457	-0.65562	-1.69505
O	-0.44758	-1.01925	-3.09842
Pb	1.87258	-1.04007	-2.71429
Cl	2.04406	-3.44837	-2.04332
C	-1.99973	0.78948	-1.73608
H	-2.86831	0.57077	-2.35997
H	-2.32923	1.06295	-0.73191
H	-1.44146	1.62224	-2.16635
Au	-0.70214	1.34600	1.94745
Cl	-2.27881	2.36093	3.22185
C	2.49746	0.27502	1.14377
C	2.98164	1.26956	1.99368
C	3.34389	-0.75381	0.72296
C	4.31059	1.25959	2.38448
H	2.31502	2.05139	2.34329
C	4.66793	-0.76248	1.12661
H	2.95396	-1.56802	0.11998
C	5.15349	0.24711	1.94862

H	4.68674	2.03970	3.03578
H	5.31954	-1.56881	0.81058
H	6.19071	0.23487	2.26343
C	-1.87855	-1.99912	-0.96893
C	-2.61756	-1.81836	0.19990
C	-1.84541	-3.24577	-1.58744
C	-3.32464	-2.87896	0.73942
H	-2.63950	-0.85600	0.70109
C	-2.55615	-4.30368	-1.04119
H	-1.25997	-3.38042	-2.48873
C	-3.29457	-4.12086	0.11798
H	-3.89823	-2.73586	1.64761
H	-2.52779	-5.27438	-1.52213
H	-3.84813	-4.95017	0.54354

R = Mes

P	0.73454	0.52191	1.00353
C	0.42080	-0.43399	-0.28025
P	-0.93507	-0.66269	-1.38092
O	-0.20244	-0.69256	-2.72487
Pb	2.01994	-0.97667	-1.97715
Cl	1.87074	-3.45923	-1.63770
Au	-0.59326	1.79992	2.23854
Cl	-1.96481	3.11891	3.47332
C	2.50772	0.47140	1.34738
C	3.28106	1.62247	1.12014
C	3.09226	-0.69805	1.86312
C	4.64234	1.57055	1.38546
C	4.45879	-0.69298	2.11640
C	5.25157	0.42269	1.87877
H	5.24330	2.45718	1.20715
H	4.91459	-1.59047	2.52328
C	-1.86716	-2.17932	-1.04366
C	-2.39200	-2.41148	0.24355
C	-2.11652	-3.11195	-2.07594
C	-3.13581	-3.56047	0.47837
C	-2.86728	-4.24291	-1.77693
C	-3.37954	-4.49602	-0.51375
H	-3.53829	-3.72177	1.47376
H	-3.06012	-4.95318	-2.57490
C	2.67340	2.89173	0.60139
H	2.15934	2.73755	-0.35194
H	1.93357	3.29526	1.29969
H	3.43906	3.65256	0.44791
C	2.29348	-1.93155	2.16482
H	1.37976	-1.69246	2.71657
H	1.99288	-2.45553	1.25318
H	2.87869	-2.62342	2.77126
C	6.72652	0.38555	2.13352
H	7.26512	0.06794	1.23513
H	7.10767	1.36953	2.41184
H	6.97546	-0.31867	2.92893
C	-2.17454	-1.50046	1.41456
H	-2.93636	-1.67558	2.17479
H	-1.20091	-1.68675	1.87535
H	-2.20713	-0.44008	1.16562
C	-1.63046	-2.98850	-3.49233
H	-1.85321	-2.01899	-3.93572
H	-0.54826	-3.11862	-3.54472
H	-2.09680	-3.76400	-4.10101
C	-4.15368	-5.74501	-0.23011
H	-4.72857	-6.06386	-1.10143
H	-3.47637	-6.56410	0.03117
H	-4.84058	-5.60776	0.60657
C	-2.11043	0.70168	-1.40755
H	-2.67046	0.78860	-0.47691
H	-1.56362	1.62827	-1.58907
H	-2.80797	0.52754	-2.22966

R = Mes*

P	0.91516	1.17362	-0.03155
C	0.10410	-0.18526	-0.42968
P	-1.49825	-0.86533	-0.72290
Cl	1.56825	-2.93693	1.81614
O	-1.27334	-2.34166	-0.36130
C	-3.14919	-0.40253	-0.06571
C	-3.66485	-1.22137	0.97897
C	-4.07300	0.31560	-0.86991
C	-5.00466	-1.57771	0.92048
C	-5.40450	-0.09168	-0.86227
C	-5.88112	-1.10328	-0.04580
H	-5.38048	-2.25723	1.67468
H	-6.09481	0.42198	-1.51686
C	2.68240	0.69969	-0.05903
C	3.36484	0.39527	1.14778
C	3.27153	0.37212	-1.30646
C	4.48283	-0.42984	1.06342
C	4.37213	-0.48087	-1.29598
C	4.96521	-0.94116	-0.13113
H	4.97916	-0.71152	1.97670
H	4.79265	-0.78639	-2.24561
C	3.00546	0.98643	2.52522
C	2.89070	0.93994	-2.69324
C	6.12150	-1.93378	-0.19862
C	-3.79690	1.63267	-1.62319
C	-2.91086	-1.64438	2.25349
C	-7.31189	-1.62352	-0.11116
C	4.17338	1.49209	-3.34402
H	3.92364	1.94488	-4.30681
H	4.62768	2.26136	-2.71542
H	4.92367	0.72412	-3.53043
C	1.91485	2.11473	-2.67380
H	0.89101	1.82070	-2.44056
H	2.22214	2.89506	-1.97364
H	1.88489	2.56480	-3.66979
C	2.31456	-0.15763	-3.59230
H	2.99510	-1.00710	-3.68311
H	1.36225	-0.52036	-3.19839
H	2.13228	0.23453	-4.59686
C	7.27964	-1.32558	-0.99795
H	8.11164	-2.03312	-1.05025
H	6.98646	-1.08237	-2.02136
H	7.64194	-0.40919	-0.52576
C	6.63983	-2.31435	1.18567
H	7.44731	-3.04316	1.08303
H	7.04336	-1.45139	1.72137
H	5.85878	-2.76881	1.79983
C	5.63660	-3.21100	-0.89557
H	4.82149	-3.67470	-0.33409
H	5.28102	-3.00951	-1.90865
H	6.45320	-3.93466	-0.96695
C	3.93297	0.47124	3.63029
H	4.98059	0.71957	3.44544
H	3.64862	0.95137	4.56889
H	3.84265	-0.60854	3.77149
C	3.20110	2.50704	2.45365
H	4.23444	2.75289	2.19582
H	2.54975	2.97480	1.71203
H	2.97334	2.95927	3.42252
C	1.58366	0.65339	2.98466
H	0.81099	1.15411	2.39814
H	1.39924	-0.42106	2.95238

H	1.44976	1.00151	4.01269
C	-2.39777	2.18995	-1.39895
H	-2.18616	2.27176	-0.33088
H	-1.59761	1.61218	-1.85646
H	-2.33361	3.19769	-1.81506
C	-4.73969	2.68650	-1.00363
H	-4.53441	3.66168	-1.45263
H	-5.79183	2.45512	-1.17065
H	-4.57588	2.77070	0.07273
C	-4.11364	1.54735	-3.11937
H	-5.15048	1.25186	-3.29223
H	-3.97000	2.52793	-3.58094
H	-3.47785	0.83449	-3.64456
C	-1.57857	-0.92620	2.43582
H	-1.20708	-1.11373	3.44591
H	-0.80489	-1.28524	1.76307
H	-1.68976	0.15510	2.31821
C	-2.70838	-3.16006	2.32475
H	-2.08550	-3.50792	1.50288
H	-2.21926	-3.42286	3.26657
H	-3.66485	-3.68787	2.28563
C	-3.77495	-1.20848	3.45424
H	-3.97507	-0.13441	3.42819
H	-4.73092	-1.73028	3.50568
H	-3.23254	-1.42982	4.37686
C	-8.10657	-0.99019	-1.25007
H	-8.22609	0.08781	-1.11523
H	-7.63494	-1.16649	-2.22019
H	-9.10724	-1.42750	-1.28351
C	-8.03239	-1.31929	1.20773
H	-7.53990	-1.79423	2.05837
H	-8.06511	-0.24354	1.39658
H	-9.06048	-1.68983	1.16807
C	-7.27761	-3.14044	-0.33247
H	-6.78158	-3.38727	-1.27436
H	-6.74478	-3.65410	0.47006
H	-8.29544	-3.53852	-0.36856
Au	0.23461	3.24123	0.44907
Cl	-0.45446	5.36232	0.89156
C	-1.68494	-0.88189	-2.52868
H	-2.62909	-1.37614	-2.76303
H	-1.64074	0.08557	-3.01746
H	-0.87067	-1.50513	-2.90503
Pb	1.07975	-2.41537	-0.59441

Coordination through π(C=P) bond

R= H

P	0.86528	-0.13003	0.91378
C	0.44558	0.14599	-0.73017
P	-1.14434	-0.11045	-1.46380
O	-0.93478	0.14511	-2.94915
Pb	1.43415	0.02133	-2.97404
Cl	1.44704	-2.47139	-2.80976
C	-2.50964	0.84047	-0.79800
H	-3.42273	0.57129	-1.33316
H	-2.63078	0.62160	0.26524
H	-2.31365	1.90555	-0.93125
Au	0.69731	2.11447	0.09103
Cl	0.79977	4.36066	0.23917
H	-1.48547	-1.45315	-1.19966
H	2.28896	-0.15552	0.77977

R = Me

P	0.87335	-0.03786	0.84513
C	0.37261	0.25739	-0.76780
P	-1.21595	-0.14519	-1.45380
O	-0.99870	-0.05747	-2.96493
Pb	1.33815	0.09725	-3.00537
Cl	1.74178	-2.36228	-2.64492
C	-2.56079	0.93198	-0.94473
H	-3.47610	0.61605	-1.45052
H	-2.70431	0.88135	0.13616
H	-2.33353	1.96004	-1.22967
C	-1.66064	-1.81053	-0.95383
H	-1.81842	-1.85930	0.12541
H	-2.57156	-2.11082	-1.47573
H	-0.84520	-2.47838	-1.23925
C	2.70537	-0.13021	0.76508
H	3.13336	0.41042	-0.07884
H	3.13102	0.24273	1.69880
H	2.96208	-1.19116	0.67443
Au	0.50631	2.21825	0.09289
Cl	0.48473	4.45794	0.37666

R = *t*-Bu

P	0.75871	-0.03767	0.78657
C	0.32754	0.29319	-0.84059
P	-1.18865	-0.19432	-1.65349
O	-0.83400	-0.12269	-3.14274
Pb	1.38794	0.58732	-3.03699
Cl	2.43305	-1.70427	-3.09916
C	-2.54391	0.94428	-1.30812
H	-3.42666	0.63504	-1.87112
H	-2.77842	0.98342	-0.24436
H	-2.25132	1.94051	-1.64350
Au	0.18196	2.20742	0.14709
Cl	-0.09141	4.40936	0.57766
C	2.61998	0.06400	0.98595
C	3.05115	-1.40491	1.08565
C	3.39172	0.75605	-0.12234
C	2.85341	0.76559	2.32463
H	2.50455	-1.93838	1.86878
H	2.90747	-1.92976	0.13773
H	4.11555	-1.45112	1.33770
H	3.03842	1.77628	-0.29488
H	4.44999	0.81773	0.15301
H	3.34734	0.18306	-1.05214
H	3.91100	0.69354	2.59676
H	2.58814	1.82470	2.27637
H	2.27088	0.30755	3.12915
C	-1.76864	-1.88603	-1.21527
C	-2.83193	-2.27306	-2.24787
C	-0.59813	-2.86348	-1.31039
C	-2.36301	-1.90600	0.19165
H	-3.71396	-1.63043	-2.19813
H	-2.43347	-2.23650	-3.26267
H	-3.16082	-3.29614	-2.04379
H	0.15714	-2.67277	-0.54619
H	-0.97703	-3.87830	-1.15760
H	-0.10654	-2.82532	-2.28356
H	-2.64478	-2.93337	0.44031
H	-1.64724	-1.57063	0.94648
H	-3.26285	-1.29216	0.26852

R = Ph

P	0.77688	0.24336	0.81323
C	0.25704	0.28535	-0.81959
P	-1.36723	-0.08598	-1.42769
O	-1.25424	0.10747	-2.93840
Pb	1.07828	0.35924	-3.13600

Cl	1.55422	-2.10243	-3.36020
C	-2.68666	0.93313	-0.75395
H	-3.63221	0.62115	-1.20195
H	-2.74454	0.82197	0.33022
H	-2.49367	1.97885	-0.99862
Au	0.45748	2.37172	-0.26391
Cl	0.48846	4.62802	-0.35661
C	-1.77990	-1.78765	-1.01825
C	-1.86855	-2.72230	-2.04477
C	-2.01542	-2.17608	0.30008
C	-2.19847	-4.03754	-1.75259
H	-1.66902	-2.41573	-3.06402
C	-2.34400	-3.48993	0.58563
H	-1.93255	-1.45872	1.11039
C	-2.43681	-4.42028	-0.44197
H	-2.26166	-4.76529	-2.55300
H	-2.52516	-3.79063	1.61097
H	-2.69163	-5.44952	-0.21618
C	2.58819	0.11480	0.74407
C	3.25345	-0.63775	-0.22457
C	3.32123	0.71862	1.76769
C	4.63421	-0.75306	-0.18530
H	2.69166	-1.16354	-0.99020
C	4.70205	0.61624	1.79157
H	2.80782	1.28418	2.53924
C	5.35947	-0.11863	0.81404
H	5.14320	-1.34706	-0.93583
H	5.26659	1.10572	2.57675
H	6.43962	-0.20719	0.83878

R = Mes

P	0.81420	0.15097	1.21337
C	0.09886	0.31466	-0.34194
P	-1.49650	-0.04587	-1.06220
O	-1.32091	0.46802	-2.49165
Pb	0.96230	1.01237	-2.53175
Cl	1.77457	-1.18187	-3.48098
Au	0.03298	2.31345	0.50698
Cl	-0.36783	4.53353	0.70157
C	-1.85726	-1.82246	-0.97096
C	-1.94037	-2.61705	-2.13797
C	-2.06802	-2.41971	0.28942
C	-2.16778	-3.98166	-1.99168
C	-2.27989	-3.78948	0.36906
C	-2.31868	-4.59499	-0.75800
H	-2.22838	-4.58659	-2.89092
H	-2.42958	-4.23471	1.34817
C	2.61601	0.15166	0.89650
C	3.15956	-1.01610	0.32716
C	3.46483	1.17061	1.35644
C	4.53245	-1.08788	0.13478
C	4.83227	1.05150	1.13617
C	5.38492	-0.05605	0.50771
H	4.94800	-1.98882	-0.30679
H	5.48610	1.84657	1.48287
C	2.95666	2.37678	2.08859
H	2.58717	3.14841	1.40610
H	2.13540	2.12902	2.76695
H	3.75421	2.82492	2.68344
C	2.31077	-2.20210	-0.02675
H	1.56499	-2.41480	0.74616
H	1.77768	-2.05520	-0.96867
H	2.93385	-3.09052	-0.13786
C	6.85709	-0.14350	0.24831
H	7.21926	-1.16841	0.34952
H	7.08920	0.18331	-0.77019
H	7.42172	0.49099	0.93345
C	-2.15359	-1.65928	1.58249
H	-1.85852	-2.29850	2.41557
H	-1.52182	-0.77344	1.62309
H	-3.18499	-1.34330	1.76853
C	-2.50771	-6.07546	-0.64814
H	-3.04449	-6.47322	-1.51114
H	-1.53904	-6.58318	-0.60450
H	-3.05846	-6.34231	0.25540
C	-1.82495	-2.10736	-3.54585
H	-2.46841	-1.24865	-3.73024
H	-0.80464	-1.79652	-3.77589
H	-2.09860	-2.90455	-4.23799
C	-2.90625	0.84357	-0.36844
H	-3.04270	0.67278	0.69679
H	-2.75098	1.90898	-0.54846
H	-3.79961	0.51980	-0.90778

R = Mes*

P	0.67231	-0.01869	1.17244
C	0.38926	0.06609	-0.53312
P	-1.05176	-0.11111	-1.63549
O	-0.35319	-0.09476	-3.00104
Pb	1.83023	0.58859	-2.48907
Cl	2.96456	-1.49313	-3.36593
Au	0.36961	2.14547	0.11359
Cl	0.12809	4.40173	-0.06756
C	2.52807	-0.07193	1.27023
C	3.23180	-1.19339	0.75121
C	3.27549	1.05933	1.67646
C	4.52962	-0.99936	0.30125
C	4.57867	1.18557	1.18785
C	5.19931	0.21416	0.42247
H	5.04787	-1.83872	-0.14342
H	5.12802	2.08196	1.43946
C	2.88822	2.08102	2.76775
C	2.78043	-2.65888	0.90777
C	6.57927	0.39660	-0.19944
C	-2.09594	1.37164	-1.67128
H	-2.59266	1.60811	-0.73566
H	-1.47206	2.21607	-1.96899
H	-2.84317	1.19607	-2.44831
C	3.87465	1.82975	3.92706
H	3.78436	0.80732	4.30273
H	4.91073	1.98566	3.62451
H	3.65504	2.51699	4.74877
C	3.04794	3.53050	2.30137
H	2.36939	3.77335	1.48236
H	2.82084	4.20755	3.12949
H	4.06602	3.74764	1.97408
C	1.49739	1.91435	3.37238
H	1.41770	2.56041	4.25085
H	0.69456	2.21580	2.69580
H	1.31094	0.88910	3.70104
C	3.10692	-3.51916	-0.31484
H	2.66425	-3.11614	-1.22433
H	4.18128	-3.60854	-0.48276
H	2.72472	-4.53142	-0.15709
C	3.58529	-3.19557	2.10761
H	4.65944	-3.13241	1.92515
H	3.36361	-2.62924	3.01589
H	3.32887	-4.24421	2.28570
C	1.30985	-2.86024	1.25313
H	1.01884	-2.31641	2.15790
H	0.64460	-2.58089	0.43705
H	1.13677	-3.92034	1.45965

C	7.17933	1.76453	0.11377
H	6.54599	2.57844	-0.24785
H	7.34128	1.90405	1.18593
H	8.14968	1.85786	-0.37969
C	6.45090	0.26039	-1.72153
H	7.43475	0.34281	-2.19215
H	6.01320	-0.69692	-2.01059
H	5.81532	1.05007	-2.13090
C	7.53838	-0.67798	0.32605
H	7.63349	-0.62079	1.41335
H	7.20498	-1.68492	0.06859
H	8.53120	-0.53806	-0.11024
C	-2.34321	-1.44109	-1.65342
C	-2.32234	-2.34702	-2.75554
C	-3.58990	-1.22324	-1.00455
C	-3.52965	-2.65501	-3.36758
C	-1.10255	-3.14033	-3.26095
C	-4.75705	-1.59800	-1.66507
C	-3.80535	-0.76867	0.45299
C	-4.76183	-2.22432	-2.89885
H	-3.50005	-3.27932	-4.25089
C	-0.61703	-2.66749	-4.63395
C	-1.52291	-4.61838	-3.40211
C	0.02615	-3.15779	-2.23943
H	-5.70084	-1.38983	-1.18096
C	-2.52622	-0.44557	1.20436
C	-4.41956	-1.98112	1.18230
C	-4.77999	0.40685	0.58035
C	-6.03496	-2.52488	-3.67986
H	0.20287	-3.30579	-4.97330
H	-1.42381	-2.73601	-5.36907
H	-0.26377	-1.64086	-4.60210
H	-2.21421	-4.79062	-4.22775
H	-0.62981	-5.21543	-3.60314
H	-1.98338	-4.99400	-2.48500
H	0.45465	-2.17856	-2.04347
H	-0.32327	-3.59026	-1.29849
H	0.84516	-3.77486	-2.61270
H	-1.80396	-1.25947	1.12256
H	-2.04865	0.47720	0.87695
H	-2.74750	-0.31410	2.26612
H	-4.56724	-1.73474	2.23747
H	-5.38466	-2.26926	0.76437
H	-3.75508	-2.84641	1.12378
H	-5.76189	0.17459	0.16509
H	-4.92125	0.64978	1.63679
H	-4.41591	1.30534	0.08011
C	-7.27739	-1.95191	-3.00331
C	-6.21322	-4.04202	-3.81260
C	-5.91656	-1.90404	-5.07689
H	-7.45622	-2.40553	-2.02506
H	-7.20525	-0.86910	-2.87352
H	-8.15518	-2.15393	-3.62163
H	-5.37601	-4.50474	-4.33859
H	-6.29472	-4.51390	-2.83033
H	-7.12458	-4.26513	-4.37423
H	-5.80368	-0.81894	-5.01463
H	-5.05758	-2.29854	-5.62310
H	-6.81468	-2.12008	-5.66200

[R-P=C(Pb(II)Cl)-P(S)ClR](AuCl)

Coordination through Pb(II) atom

R= H

P	0.85812	0.50629	0.64378
C	0.40446	-0.01987	-0.86145
P	-1.23757	-0.32377	-1.38076
Pb	1.36022	-0.05595	-2.95982
Cl	1.26032	-2.49436	-3.19750
Au	2.99073	1.51417	-4.23192
Cl	4.42334	2.90982	-5.26705
Cl	-2.64504	0.40798	-0.14131
H	2.25804	0.64239	0.38661
H	-1.49865	-1.70043	-1.27550
S	-1.36300	0.34842	-3.24003

R = Me

P	0.85491	0.24846	0.73057
C	0.36468	-0.11953	-0.80731
P	-1.30102	-0.28861	-1.31766
Pb	1.30844	-0.05470	-2.90540
Cl	1.24558	-2.48774	-3.31164
C	-1.83956	-1.99551	-1.19862
H	-1.82217	-2.31259	-0.15486
H	-2.84810	-2.07825	-1.60585
H	-1.14541	-2.59601	-1.79184
C	2.68192	0.34242	0.64230
H	3.10036	0.09940	-0.33440
H	2.99112	1.35122	0.93157
H	3.09252	-0.34102	1.39135
Au	3.03475	1.48858	-4.08163
Cl	4.57344	2.85670	-5.00134
Cl	-2.59720	0.69327	-0.10996
S	-1.35417	0.41416	-3.18617

R = *t*-Bu

P	0.72458	-0.02975	0.61390
C	0.47614	-0.13425	-1.02295
P	-1.14086	-0.39920	-1.68462
Pb	1.60422	0.30153	-2.98327
Cl	2.37482	-1.99285	-3.46219
Au	2.88512	2.34060	-3.96348
Cl	4.00735	4.15141	-4.71323
C	2.54338	0.19272	0.97536
C	2.91404	-1.04305	1.80481
C	3.46682	0.31884	-0.21996
C	2.62838	1.44376	1.85564
H	2.24702	-1.17617	2.66116
H	2.88814	-1.95522	1.20378
H	3.93078	-0.92366	2.19235
H	3.26262	1.22439	-0.79874
H	4.50371	0.40006	0.12235
H	3.41748	-0.55910	-0.87091
H	3.64986	1.55294	2.23315
H	2.37904	2.34929	1.29725
H	1.95954	1.38044	2.71828
C	-1.82514	-2.09898	-1.40797
C	-3.16432	-2.19341	-2.13715
C	-0.82557	-3.08785	-2.00807
C	-2.00538	-2.36410	0.08301
H	-3.90586	-1.50859	-1.72254
H	-3.05787	-1.99225	-3.20465
H	-3.54457	-3.21216	-2.02297
H	0.14757	-3.04176	-1.51706
H	-1.22595	-4.09686	-1.87598
H	-0.67234	-2.91967	-3.07467

H	-2.38050	-3.38413	0.20407
H	-1.06372	-2.29130	0.63071
H	-2.72532	-1.68357	0.53898
Cl	-2.48283	0.81869	-0.75396
S	-1.02122	0.06946	-3.62405

R = Ph

P	0.78939	0.38577	0.70801
C	0.34226	-0.11496	-0.81010
P	-1.28980	-0.28215	-1.41691
Pb	1.48037	-0.14154	-2.79155
Cl	1.59531	-2.58655	-3.11295
Au	3.18676	1.41451	-3.98331
Cl	4.70005	2.78322	-4.95012
C	2.59582	0.31260	0.66255
C	3.29098	1.29620	1.36953
C	3.31359	-0.68031	-0.01307
C	4.67620	1.32274	1.35157
H	2.74262	2.05433	1.91990
C	4.69837	-0.65847	-0.01444
H	2.78934	-1.49522	-0.50396
C	5.37925	0.34908	0.65677
H	5.20838	2.10376	1.88178
H	5.24771	-1.43235	-0.53770
H	6.46276	0.36834	0.64594
C	-1.94700	-1.92505	-1.09532
C	-2.10301	-2.82258	-2.14525
C	-2.24313	-2.30874	0.21267
C	-2.56609	-4.10395	-1.88575
H	-1.85678	-2.51979	-3.15545
C	-2.70320	-3.58840	0.46117
H	-2.12096	-1.60953	1.03235
C	-2.86550	-4.48554	-0.58814
H	-2.68748	-4.80441	-2.70324
H	-2.93728	-3.88821	1.47579
H	-3.22604	-5.48816	-0.38885
Cl	-2.56136	0.94363	-0.41326
S	-1.19376	0.17858	-3.35128

R = Mes

P	0.85888	0.20176	1.23168
C	0.32162	-0.20772	-0.28574
P	-1.28470	-0.22698	-1.00709
Pb	1.61916	0.01627	-2.14056
Cl	1.51561	-2.32766	-2.93346
Au	3.61557	1.50313	-2.89810
Cl	5.42403	2.77270	-3.39924
C	2.66187	0.24999	0.94768
C	3.28700	1.50778	0.89232
C	3.40827	-0.92725	0.77887
C	4.64170	1.56752	0.60264
C	4.76610	-0.81297	0.49638
C	5.39704	0.41940	0.38374
H	5.11922	2.53929	0.52426
H	5.34753	-1.71978	0.35980
C	-1.99639	-1.89325	-1.05608
C	-2.30933	-2.55793	-2.26644
C	-2.18335	-2.56280	0.17823
C	-2.73676	-3.87863	-2.19657
C	-2.60264	-3.88557	0.16823
C	-2.87197	-4.57117	-1.00467
H	-2.97229	-4.38422	-3.12721
H	-2.73287	-4.38955	1.12066
Cl	-2.61939	0.88794	0.06427
C	2.50743	2.77539	1.08449
H	1.81833	2.70654	1.93218
H	3.17614	3.61811	1.26192
H	1.90752	3.01488	0.20063
C	2.78977	-2.29004	0.90394
H	2.08806	-2.33912	1.74137
H	2.23671	-2.57435	0.00380
H	3.56130	-3.04350	1.06705
C	6.84351	0.52572	0.01798
H	6.94891	0.99533	-0.96474
H	7.38710	1.14758	0.73351
H	7.32070	-0.45419	-0.01800
C	-2.02802	-1.93342	1.52993
H	-1.15860	-1.28394	1.62077
H	-2.90345	-1.32722	1.77473
H	-1.93737	-2.71015	2.28961
C	-2.24079	-1.97029	-3.64532
H	-2.79429	-1.03585	-3.72934
H	-1.21187	-1.77122	-3.94741
H	-2.66684	-2.68081	-4.35396
C	-3.28458	-6.00840	-0.98752
H	-2.40824	-6.65641	-1.08787
H	-3.78157	-6.27076	-0.05232
H	-3.95677	-6.23971	-1.81544
S	-0.95733	0.75304	-2.71993

R = Mes*

P	0.13246	2.21629	1.01999
C	0.46114	0.70095	0.41208
P	1.97783	-0.19212	0.24493
Cl	-1.28020	0.47491	-2.81078
C	3.71792	0.37948	0.14180
C	4.30788	0.36927	-1.15879
C	4.57667	0.31459	1.27246
C	5.59772	-0.12553	-1.26824
C	5.86311	-0.17737	1.07227
C	6.36394	-0.51085	-0.17522
H	6.02987	-0.19384	-2.25870
H	6.49977	-0.28880	1.93876
C	-1.69630	2.28192	0.85341
C	-2.26825	3.07956	-0.16637
C	-2.52590	1.43807	1.63643
C	-3.57689	2.80037	-0.54935
C	-3.81502	1.19537	1.17379
C	-4.34581	1.81328	0.04889
H	-4.00782	3.36587	-1.35945
H	-4.44160	0.51006	1.72856
C	-1.55093	4.28805	-0.79751
C	-2.15990	0.88925	3.03438
C	-5.74434	1.44769	-0.43726
C	4.32253	0.93259	2.66417
C	3.74881	1.05828	-2.42153
C	7.72205	-1.16862	-0.37888
C	-3.39613	0.96894	3.94699
H	-3.09774	0.73186	4.97104
H	-3.83538	1.96913	3.94197
H	-4.16974	0.25219	3.67007
C	-1.08861	1.73555	3.72114
H	-0.10279	1.65122	3.24998
H	-1.36399	2.79257	3.74798
H	-0.95484	1.39102	4.75001
C	-1.70706	-0.57189	2.99602
H	-2.45211	-1.20673	2.50999
H	-0.75729	-0.68790	2.47265
H	-1.56766	-0.94556	4.01464
C	-6.76567	1.73075	0.67106
H	-7.77058	1.47121	0.32735
H	-6.56340	1.14608	1.57057

H	-6.76285	2.78836	0.94733
C	-6.15049	2.24010	-1.67714
H	-7.14366	1.92040	-2.00089
H	-6.20014	3.31439	-1.48000
H	-5.46182	2.07115	-2.50860
C	-5.77516	-0.04425	-0.78879
H	-5.06652	-0.27256	-1.58855
H	-5.52711	-0.67589	0.06639
H	-6.77395	-0.32868	-1.13070
C	-2.46440	5.05846	-1.75386
H	-3.37522	5.40892	-1.26312
H	-1.92719	5.93763	-2.11672
H	-2.74281	4.46238	-2.62590
C	-1.15389	5.26014	0.32297
H	-2.03187	5.58138	0.88863
H	-0.44481	4.81577	1.02493
H	-0.68022	6.14793	-0.10533
C	-0.31046	3.89532	-1.60064
H	0.49092	3.51050	-0.96371
H	-0.53903	3.13081	-2.34421
H	0.09297	4.77671	-2.10796
C	2.97546	1.62513	2.81949
H	2.79711	2.33434	2.00860
H	2.13984	0.93124	2.87501
H	2.97384	2.19538	3.75186
C	5.37383	2.05628	2.79833
H	5.22126	2.57825	3.74681
H	6.39494	1.67471	2.78525
H	5.27407	2.78409	1.98953
C	4.53254	-0.05481	3.81497
H	5.52573	-0.50670	3.78957
H	4.43816	0.47382	4.76724
H	3.79637	-0.85733	3.80021
C	2.41569	1.76666	-2.22009
H	2.22099	2.40606	-3.08443
H	1.56789	1.08900	-2.14492
H	2.44379	2.41102	-1.33807
C	3.65861	0.12542	-3.63081
H	2.91921	-0.65866	-3.47355
H	3.36060	0.70130	-4.51078
H	4.61734	-0.34654	-3.85548
C	4.76292	2.17690	-2.74680
H	4.86959	2.86685	-1.90595
H	5.75162	1.78754	-2.99136
H	4.40260	2.74439	-3.60878
C	8.39428	-1.52793	0.94342
H	8.62606	-0.64136	1.53926
H	7.77327	-2.19637	1.54491
H	9.33751	-2.04165	0.74418
C	8.64704	-0.21973	-1.15055
H	8.24698	0.02755	-2.13577
H	8.79659	0.71415	-0.60314
H	9.62398	-0.68812	-1.29721
C	7.52567	-2.45679	-1.18772
H	6.87810	-3.15817	-0.65641
H	7.07458	-2.25788	-2.16181
H	8.48990	-2.94347	-1.35706
Au	-2.70598	-2.58387	-0.15540
Cl	-4.27195	-4.12669	0.39302
Pb	-0.93951	-0.74667	-0.68171
Cl	1.99537	-1.39739	1.91620
S	1.61714	-1.48550	-1.24142

Coordination through S atom

R= H

P	0.89866	0.11767	1.05349
C	0.60297	-0.15506	-0.55454
P	-0.89646	0.16422	-1.35234
Pb	1.92303	-0.53152	-2.53831
Cl	0.69129	-2.62896	-3.03933
Au	-1.96850	0.77745	-4.53975
Cl	-3.56313	0.41534	-6.08544
Cl	-2.35203	1.06322	-0.29241
H	2.27610	-0.27562	1.07470
H	-1.49938	-1.04877	-1.71195
S	-0.34773	1.20752	-3.00574

R = Me

P	0.90809	0.13432	1.05823
C	0.59150	-0.11794	-0.54762
P	-0.96160	0.15765	-1.25899
Pb	1.92088	-0.32733	-2.55009
Cl	1.14746	-2.63851	-3.08058
C	-1.86839	-1.36655	-1.50334
H	-2.07648	-1.80108	-0.52364
H	-2.79620	-1.14862	-2.03392
H	-1.24152	-2.03980	-2.09346
C	2.67234	-0.32531	1.27370
H	3.15297	-0.69006	0.36569
H	3.21702	0.54132	1.65999
H	2.72920	-1.10091	2.04339
Au	-2.09761	0.54633	-4.53164
Cl	-3.68092	0.04988	-6.05478
Cl	-2.23015	1.29897	-0.16304
S	-0.50068	1.10249	-3.01108

R = *t*-Bu

P	0.98540	0.11770	0.74989
C	0.75370	-0.10741	-0.87928
P	-0.84438	0.14707	-1.53834
Pb	2.15016	-0.21936	-2.83763
Cl	2.11455	-2.70815	-3.09639
C	-1.97298	-1.32898	-1.41637
C	2.74123	-0.34494	1.20201
Au	-2.05810	0.32090	-4.95008
Cl	-3.59924	-0.12081	-6.53605
Cl	-1.86056	1.60640	-0.54140
S	-0.44927	0.82333	-3.42536
C	3.69125	-0.61249	0.05146
H	3.35191	-1.44688	-0.56989
H	3.83642	0.28222	-0.56355
H	4.67902	-0.88708	0.43691
C	3.25926	0.80610	2.06849
H	2.57981	1.02589	2.89626
H	4.23046	0.53765	2.49614
H	3.38956	1.72322	1.48805
C	2.58903	-1.60462	2.06334
H	3.55708	-1.86155	2.50588
H	1.88134	-1.45419	2.88354
H	2.25117	-2.45826	1.47098
C	-1.98607	-1.78092	0.04378
H	-2.69722	-2.60713	0.12751
H	-1.01083	-2.14479	0.36857
H	-2.31254	-0.99023	0.72209
C	-3.38459	-0.94931	-1.85597
H	-3.84503	-0.22474	-1.18438
H	-3.41127	-0.55488	-2.87465
H	-3.99107	-1.85882	-1.84437

C	-1.41359	-2.43390	-2.30834
H	-1.43631	-2.15529	-3.36320
H	-0.39159	-2.71086	-2.04617
H	-2.04651	-3.31698	-2.18413

R = Ph

P	0.85993	0.54383	0.85395
C	0.55410	-0.07172	-0.65641
P	-0.95175	0.03249	-1.49942
Pb	2.13505	-0.56272	-2.37340
Cl	1.73159	-3.02537	-2.50231
Au	-2.03098	0.02904	-4.86595
Cl	-3.63556	-0.40207	-6.39010
C	2.58411	0.05021	1.14301
C	3.12291	-1.16230	0.69691
C	3.39211	0.92225	1.87522
C	4.45216	-1.47020	0.94404
H	2.48646	-1.89023	0.20104
C	4.72386	0.61684	2.10895
H	2.97646	1.85154	2.25255
C	5.25541	-0.57666	1.64045
H	4.85638	-2.41658	0.60333
H	5.34738	1.30880	2.66326
H	6.29416	-0.81923	1.83277
C	-1.90033	-1.48613	-1.35685
C	-2.66849	-1.70248	-0.21239
C	-1.80666	-2.46724	-2.33854
C	-3.34954	-2.89665	-0.06380
H	-2.74458	-0.93929	0.55278
C	-2.49548	-3.65925	-2.17967
H	-1.20145	-2.30835	-3.22106
C	-3.26565	-3.87318	-1.04827
H	-3.95426	-3.06245	0.81986
H	-2.42888	-4.41801	-2.94964
H	-3.80718	-4.80482	-0.93132
Cl	-2.19204	1.46701	-0.76897
S	-0.37344	0.51287	-3.38546

R = Mes

P	0.71571	0.24598	1.25883
C	0.38582	-0.18340	-0.30738
P	-1.06333	-0.05692	-1.25537
Pb	2.21152	-0.35906	-1.81145
Cl	1.93055	-2.80362	-2.28194
Au	-1.83996	1.49155	-4.39522
Cl	-3.36242	2.23507	-5.88403
C	2.52759	-0.05626	1.31607
C	3.03894	-1.35898	1.45599
C	3.39855	1.04772	1.28068
C	4.41894	-1.52917	1.50600
C	4.76808	0.82498	1.33404
C	5.29915	-0.45637	1.43717
H	4.81535	-2.53406	1.61621
H	5.43951	1.67810	1.30320
C	-1.94944	-1.63783	-1.33403
C	-2.53423	-2.10152	-0.13079
C	-2.02023	-2.43459	-2.50241
C	-3.16530	-3.33831	-0.12618
C	-2.67817	-3.65542	-2.42220
C	-3.25851	-4.13066	-1.25726
H	-3.60151	-3.68686	0.80463
H	-2.72780	-4.26241	-3.32007
Cl	-2.42953	1.29497	-0.57035
C	2.87060	2.44863	1.17520
H	2.42502	2.64383	0.19459
H	2.09006	2.64112	1.91778
H	3.66712	3.17750	1.32803
C	2.13963	-2.55425	1.57062
H	1.30224	-2.36219	2.24745
H	1.71563	-2.83403	0.60181
H	2.69271	-3.41350	1.95155
C	6.78054	-0.67369	1.46574
H	7.17487	-0.78262	0.45059
H	7.29791	0.16890	1.92768
H	7.03934	-1.58044	2.01466
C	-2.56572	-1.35020	1.16785
H	-1.65734	-0.78716	1.37847
H	-3.39419	-0.63857	1.18515
H	-2.71338	-2.05196	1.98946
C	-1.44592	-2.08892	-3.84203
H	-1.90244	-1.19230	-4.26517
H	-0.36975	-1.92511	-3.79598
H	-1.62721	-2.91180	-4.53291
C	-3.97383	-5.44384	-1.23278
H	-4.07905	-5.82296	-0.21544
H	-4.97839	-5.33997	-1.65423
H	-3.44835	-6.19198	-1.82939
S	-0.25854	0.79151	-2.91487

R = Mes*

P	1.78778	2.14533	0.10305
C	0.92176	0.75060	-0.15515
P	-0.80082	0.50801	-0.22643
C	3.51607	1.50978	-0.03967
C	4.30222	1.37185	1.13423
C	4.00421	1.00331	-1.26595
C	5.38866	0.51083	1.08781
C	5.08222	0.11482	-1.22315
C	5.75440	-0.19451	-0.05355
H	5.96799	0.35984	1.98647
H	5.42329	-0.32241	-2.15052
C	3.54355	1.46569	-2.66705
C	4.04721	2.18006	2.41977
C	6.89890	-1.20044	0.00575
C	2.73559	1.79306	3.10513
H	1.86062	2.08190	2.51756
H	2.67730	0.71738	3.27776
H	2.65489	2.31366	4.06402
C	4.03293	3.67549	2.07308
H	4.97815	3.97783	1.61539
H	3.22579	3.93516	1.38490
H	3.89060	4.26565	2.98262
C	5.15860	1.97434	3.45184
H	5.18655	0.94890	3.82745
H	6.14377	2.23109	3.05508
H	4.96678	2.62815	4.30553
C	2.64039	0.43752	-3.35224
H	1.68069	0.34255	-2.84192
H	2.43540	0.74376	-4.38224
H	3.11889	-0.54488	-3.38972
C	2.83953	2.82115	-2.63672
H	1.86076	2.78925	-2.15003
H	3.44734	3.57705	-2.13387
H	2.65583	3.15947	-3.66008
C	4.78358	1.67072	-3.55624
H	5.51382	2.32560	-3.07557
H	5.28167	0.73509	-3.81223
H	4.47575	2.13536	-4.49619
C	7.12327	-1.90101	-1.33164
H	7.42182	-1.20128	-2.11666
H	7.92501	-2.63571	-1.22752
H	6.22924	-2.43426	-1.66538

C	6.57730	-2.26642	1.06003
H	6.45233	-1.83095	2.05290
H	5.65308	-2.79612	0.81881
H	7.38952	-2.99664	1.11308
C	8.19426	-0.47748	0.39428
H	8.10981	0.00767	1.36871
H	9.02110	-1.19111	0.44786
H	8.45225	0.28813	-0.34179
C	-2.25398	1.49572	0.27097
C	-2.89724	1.10686	1.47939
C	-3.01560	2.19963	-0.70841
C	-4.28621	1.04102	1.47131
C	-4.39383	2.09673	-0.63374
C	-5.05615	1.41570	0.38284
H	-4.77943	0.67556	2.36103
H	-4.98327	2.58175	-1.40178
C	-2.22844	0.95178	2.86015
C	-2.55536	-0.37277	3.55314
C	-2.81920	2.09983	3.70810
H	-2.11507	-1.21929	3.02792
H	-2.15103	-0.35737	4.56870
H	-3.63057	-0.54300	3.62906
H	-2.61623	3.07111	3.25001
H	-3.89773	2.00584	3.83749
H	-2.35721	2.08657	4.69889
C	-2.47420	3.24269	-1.70864
C	-3.07949	4.58680	-1.25072
C	-2.91988	2.98491	-3.15022
H	-2.79394	4.81337	-0.22068
H	-2.70493	5.38875	-1.89287
H	-4.16828	4.59026	-1.30692
H	-2.50332	2.05868	-3.54427
H	-4.00612	2.92841	-3.23768
H	-2.58239	3.80698	-3.78721
C	-6.56098	1.20183	0.31498
C	-7.27262	2.55989	0.34693
C	-7.07203	0.35213	1.47546
H	-6.98079	3.19167	-0.49492
H	-8.35470	2.41335	0.29275
H	-7.04878	3.10156	1.26963
H	-6.58017	-0.62304	1.50331
H	-6.93220	0.84620	2.44081
H	-8.14356	0.17922	1.35216
Cl	2.50134	-1.80468	1.99850
Cl	-1.12595	0.17506	-2.22866
Au	-2.94552	-2.24617	-0.18194
Cl	-4.93650	-3.11681	-0.82060
C	-0.96434	3.44321	-1.66946
H	-0.60867	3.61562	-0.65108
H	-0.41218	2.61149	-2.10169
H	-0.70934	4.32972	-2.25577
C	-0.72086	1.17155	2.86952
H	-0.15504	0.34807	2.43577
H	-0.45398	2.09994	2.35776
H	-0.38543	1.26249	3.90554
C	-6.89630	0.47563	-0.99419
H	-6.59051	1.05466	-1.86842
H	-6.40402	-0.49859	-1.04156
H	-7.97621	0.31800	-1.06117
Pb	1.73725	-1.50115	-0.37462
S	-0.97291	-1.37952	0.55991

Coordination through P(sp^2) atom

R= H

P	0.68557	0.07500	0.87784
C	0.41465	-0.33302	-0.68271
P	-1.08390	-0.32739	-1.55340
Pb	1.82468	-0.56723	-2.64739
Cl	1.17171	-2.96149	-2.89655
Au	-0.57097	0.73981	2.55412
Cl	-1.82910	1.42606	4.29609
Cl	-2.64384	0.56170	-0.63561
H	2.08176	-0.04902	1.05383
H	-1.53746	-1.65514	-1.60741
S	-0.69892	0.43004	-3.34178

R = Me

P	0.76845	0.17666	0.86028
C	0.40447	-0.22646	-0.68127
P	-1.16812	-0.28630	-1.41817
Pb	1.69182	-0.53123	-2.70752
Cl	1.35004	-3.01781	-2.68955
C	-1.88950	-1.91115	-1.17902
H	-2.05991	-2.08022	-0.11458
H	-2.83133	-1.96015	-1.72718
H	-1.18228	-2.64348	-1.57666
C	2.55081	0.05124	1.15378
H	3.08453	-0.31454	0.27730
H	2.93205	1.03237	1.44691
H	2.71324	-0.62867	1.99362
Au	-0.50574	0.79197	2.55196
Cl	-1.77784	1.40012	4.31926
Cl	-2.53036	0.95662	-0.57114
S	-0.90059	0.17229	-3.33890

R = *t*-Bu

P	0.72545	0.19242	0.83753
C	0.45432	-0.11506	-0.75085
P	-1.12620	-0.28354	-1.48170
Pb	1.77076	0.12960	-2.77886
Cl	2.37858	-2.29605	-3.01118
Au	-0.71581	0.55653	2.48229
Cl	-2.15772	0.91892	4.19256
C	2.51248	0.18494	1.33111
C	2.66473	-1.00492	2.28677
C	3.46696	0.03945	0.15877
C	2.77806	1.48960	2.08543
H	1.96623	-0.94485	3.12490
H	2.51112	-1.95708	1.77384
H	3.67960	-0.99953	2.69585
H	3.42347	0.90895	-0.50383
H	4.49229	-0.01629	0.53695
H	3.28199	-0.87242	-0.41469
H	3.79850	1.47096	2.47944
H	2.68214	2.36218	1.43474
H	2.09467	1.61632	2.92829
C	-1.90348	-1.94372	-1.19348
C	-3.23579	-1.97199	-1.94086
C	-0.95722	-2.99809	-1.76584
C	-2.11983	-2.17906	0.29709
H	-3.95197	-1.25919	-1.52974
H	-3.10663	-1.76834	-3.00547
H	-3.66024	-2.97445	-1.83899
H	0.01629	-2.99422	-1.27376
H	-1.41269	-3.98052	-1.61256
H	-0.79163	-2.86259	-2.83515
H	-2.59353	-3.15694	0.42143

H	-1.17894	-2.19943	0.84960
H	-2.77302	-1.43146	0.74919
Cl	-2.46568	1.02597	-0.67480
S	-0.91145	0.11813	-3.42592

R = Ph

P	0.74203	0.43860	0.43091
C	0.55167	-0.43352	-0.94204
P	-0.90744	-0.73189	-1.83395
Pb	2.15713	-1.17237	-2.57073
Cl	2.13127	-3.58195	-1.85200
Au	-0.68162	1.69207	1.56491
Cl	-2.14122	2.95341	2.74854
C	2.42545	0.29301	1.02165
C	2.97155	1.35735	1.74059
C	3.18298	-0.86170	0.80363
C	4.27674	1.28541	2.19817
H	2.37188	2.24040	1.93595
C	4.48354	-0.92889	1.27388
H	2.74194	-1.72249	0.31007
C	5.03287	0.14578	1.96179
H	4.70182	2.11776	2.74645
H	5.06424	-1.82996	1.11467
H	6.05097	0.08764	2.32935
C	-1.82840	-2.08449	-1.08483
C	-2.60987	-1.86113	0.04860
C	-1.69077	-3.36746	-1.60494
C	-3.25856	-2.92447	0.65025
H	-2.72036	-0.86260	0.45573
C	-2.34461	-4.42602	-0.99325
H	-1.07141	-3.53503	-2.47737
C	-3.12673	-4.20575	0.12949
H	-3.87134	-2.75110	1.52692
H	-2.23755	-5.42547	-1.39758
H	-3.63796	-5.03564	0.60389
Cl	-2.19632	0.83853	-1.75203
S	-0.36852	-1.08458	-3.71658

R = Mes

P	0.67340	0.61820	0.95879
C	0.35685	-0.35241	-0.31999
P	-0.99954	-0.54774	-1.39060
Pb	2.14868	-0.97271	-1.79051
Cl	1.70473	-3.44325	-1.63972
Au	-0.60153	1.97095	2.15792
Cl	-1.87262	3.35691	3.42008
C	2.43634	0.47729	1.33155
C	3.27630	1.56895	1.05268
C	2.94652	-0.69346	1.91852
C	4.62943	1.45349	1.33973
C	4.30808	-0.75339	2.18911
C	5.16614	0.30130	1.90231
H	5.28244	2.29328	1.12244
H	4.70735	-1.65198	2.64931
C	-1.86837	-2.11232	-1.09789
C	-2.29754	-2.38617	0.22149
C	-2.12469	-3.04971	-2.12549
C	-2.92282	-3.59647	0.48762
C	-2.75400	-4.24072	-1.78518
C	-3.14768	-4.54781	-0.49285
H	-3.25207	-3.79066	1.50358
H	-2.94902	-4.95572	-2.57780
Cl	-2.44873	0.88047	-1.15752
C	2.74512	2.84023	0.46062
H	2.20691	2.66302	-0.47532
H	2.04366	3.33316	1.14096
H	3.55589	3.53864	0.25225
C	2.07080	-1.85824	2.27449
H	1.18542	-1.53536	2.83024
H	1.72083	-2.39317	1.38693
H	2.61544	-2.56748	2.89816
C	6.63360	0.19381	2.17871
H	7.15996	-0.21658	1.31121
H	7.07088	1.17000	2.39443
H	6.83211	-0.46781	3.02334
C	-2.13108	-1.45965	1.38851
H	-2.85821	-1.70982	2.16145
H	-1.13674	-1.56230	1.82942
H	-2.27374	-0.40969	1.13796
C	-1.78949	-2.88133	-3.57801
H	-2.16124	-1.94391	-3.99037
H	-0.71134	-2.90490	-3.74300
H	-2.23589	-3.70026	-4.14275
C	-3.78685	-5.86092	-0.17022
H	-4.38399	-6.22958	-1.00602
H	-3.02179	-6.61438	0.04185
H	-4.42835	-5.78765	0.70932
S	-0.14613	-0.22407	-3.16834

R = Mes*

P	-0.85435	1.09617	0.13034
C	-0.25346	-0.42114	0.29683
P	1.34047	-1.13304	0.30009
Cl	-2.44120	-2.37799	-2.20365
C	3.01605	-0.43359	0.05643
C	3.61940	-0.68525	-1.21380
C	3.85915	-0.13760	1.16267
C	4.96192	-1.02913	-1.22342
C	5.19793	-0.50640	1.06098
C	5.75450	-1.05042	-0.08352
H	5.41394	-1.27377	-2.17615
H	5.83122	-0.33978	1.92060
C	-2.66874	0.92863	0.19602
C	-3.44349	0.96562	-0.99213
C	-3.25622	0.49123	1.41177
C	-4.68589	0.33897	-0.96654
C	-4.48559	-0.15381	1.33578
C	-5.19598	-0.29654	0.15342
H	-5.26313	0.31437	-1.87527
H	-4.91577	-0.54593	2.24788
C	-3.04195	1.72231	-2.27237
C	-2.71502	0.75298	2.83469
C	-6.49766	-1.09042	0.13203
C	3.52711	0.73745	2.39232
C	3.00313	-0.41254	-2.60351
C	7.18891	-1.55776	-0.15191
C	-3.87645	1.24593	3.71785
H	-3.48350	1.53258	4.69626
H	-4.36348	2.11934	3.27831
H	-4.63688	0.48424	3.89007
C	-1.66287	1.85821	2.90142
H	-0.69522	1.56007	2.49506
H	-1.99141	2.76642	2.39067
H	-1.47729	2.11436	3.94790
C	-2.14596	-0.52592	3.45500
H	-2.88637	-1.32989	3.45337
H	-1.26066	-0.86883	2.91650
H	-1.85433	-0.34308	4.49325
C	-7.50363	-0.46555	1.10544
H	-8.43783	-1.03362	1.09462
H	-7.13072	-0.46046	2.13175
H	-7.73015	0.56609	0.82524

C	-7.13025	-1.12815	-1.25652
H	-8.04237	-1.72872	-1.22336
H	-7.40761	-0.13025	-1.60570
H	-6.46166	-1.57998	-1.99306
C	-6.19918	-2.53243	0.56118
H	-5.49096	-3.00235	-0.12590
H	-5.77770	-2.57561	1.56812
H	-7.11901	-3.12367	0.55933
C	-4.10308	1.59878	-3.37004
H	-5.07326	1.99256	-3.05857
H	-3.77676	2.18624	-4.23082
H	-4.22892	0.56620	-3.70426
C	-2.93037	3.21378	-1.92790
H	-3.88175	3.59563	-1.54890
H	-2.16591	3.40958	-1.17265
H	-2.66332	3.78535	-2.82064
C	-1.73654	1.21940	-2.89157
H	-0.85955	1.44627	-2.28317
H	-1.76768	0.14261	-3.06343
H	-1.57435	1.72491	-3.84775
C	2.10373	1.27544	2.45276
H	1.81902	1.73739	1.50435
H	1.36549	0.52506	2.72279
H	2.05630	2.06039	3.21137
C	4.42026	1.98917	2.24035
H	4.22560	2.66992	3.07323
H	5.48290	1.74551	2.24531
H	4.19463	2.52059	1.31301
C	3.87109	0.05783	3.72095
H	4.92204	-0.23131	3.77635
H	3.68159	0.75493	4.54148
H	3.26646	-0.83328	3.88791
C	1.57785	0.12427	-2.58942
H	1.33207	0.48799	-3.59005
H	0.83402	-0.62923	-2.33793
H	1.48313	0.97432	-1.90789
C	3.07538	-1.62572	-3.53382
H	2.45878	-2.44496	-3.16451
H	2.70904	-1.34396	-4.52450
H	4.09676	-1.99228	-3.65393
C	3.85978	0.72236	-3.20612
H	3.83130	1.61292	-2.57377
H	4.90280	0.43365	-3.33946
H	3.45723	0.99067	-4.18639
C	7.88222	-1.51710	1.20722
H	7.98862	-0.49597	1.58212
H	7.34335	-2.10513	1.95436
H	8.88676	-1.93670	1.11591
C	7.99511	-0.69774	-1.13265
H	7.58035	-0.73398	-2.14186
H	8.01406	0.34728	-0.81410
H	9.02657	-1.05692	-1.18415
C	7.17375	-3.01040	-0.64322
H	6.61404	-3.65006	0.04332
H	6.71589	-3.09876	-1.63039
H	8.19545	-3.39381	-0.71127
Au	0.26999	3.00142	-0.16467
Cl	1.46591	4.91220	-0.44327
Pb	-1.47817	-2.52928	0.12185
Cl	1.43782	-1.89617	2.22690
S	1.13025	-2.79236	-0.79429

Coordination through π(C=P) bond

R= H

P	0.68291	-0.00181	1.00197
C	0.38199	0.08014	-0.69485
P	-1.15377	-0.21235	-1.47027
Pb	1.62312	-0.01380	-2.81056
Cl	1.31045	-2.49667	-2.91934
Au	0.58793	2.12408	-0.07253
Cl	0.77210	4.36437	-0.04834
Cl	-2.80326	0.32238	-0.42245
H	-1.31787	-1.60651	-1.50512
H	2.10928	-0.07681	0.94500
S	-1.07734	0.60017	-3.27187

R = Me

P	0.72839	0.17031	0.91650
C	0.31221	0.19277	-0.75316
P	-1.27341	-0.17989	-1.39377
Pb	1.46080	0.07964	-2.91359
Cl	1.58162	-2.43801	-2.86772
C	-1.60582	-1.92838	-1.16042
H	-1.65916	-2.15044	-0.09340
H	-2.55388	-2.16745	-1.64361
H	-0.79273	-2.48708	-1.63126
C	2.55034	-0.04895	0.93321
H	3.04123	0.24725	0.00642
H	2.97204	0.51136	1.77019
H	2.74033	-1.11248	1.11273
Au	0.52099	2.26660	-0.22162
Cl	0.69933	4.51084	-0.22673
Cl	-2.81241	0.70280	-0.38923
S	-1.26790	0.36371	-3.30755

R = *t*-Bu

P	0.59335	0.26567	0.76539
C	0.32975	0.25049	-0.93696
P	-1.21763	-0.18855	-1.65747
Pb	1.58159	0.59207	-3.03604
Cl	2.48283	-1.74360	-3.33601
Au	0.34963	2.35272	-0.38402
Cl	0.41590	4.60479	-0.33370
C	2.43077	0.15202	1.10638
C	2.61619	-1.30253	1.55793
C	3.36456	0.45915	-0.04914
C	2.69407	1.09519	2.28105
H	1.94804	-1.56403	2.38352
H	2.44678	-2.00432	0.73728
H	3.64360	-1.44097	1.90917
H	3.21958	1.47501	-0.42808
H	4.40315	0.38737	0.29079
H	3.24993	-0.26285	-0.86225
H	3.71241	0.94262	2.65152
H	2.59485	2.14257	1.98596
H	2.00654	0.91127	3.11148
C	-1.68245	-1.96525	-1.37382
C	-2.96503	-2.23883	-2.15916
C	-0.55102	-2.84081	-1.90715
C	-1.90539	-2.23360	0.11121
H	-3.80586	-1.64884	-1.79123
H	-2.83558	-2.04008	-3.22420
H	-3.21608	-3.29644	-2.04034
H	0.38747	-2.68022	-1.37530
H	-0.84056	-3.88745	-1.77622
H	-0.36384	-2.67094	-2.96771
H	-2.17108	-3.28804	0.22854

H	-1.00739	-2.05292	0.70511
H	-2.71699	-1.63312	0.52305
Cl	-2.77910	0.82415	-0.79802
S	-1.11855	0.27661	-3.59372

R = Ph

P	0.67974	0.42885	0.85725
C	0.16174	0.32061	-0.78075
P	-1.45829	-0.05346	-1.32302
Pb	1.20718	0.31255	-2.99948
Cl	1.42857	-2.18769	-3.24568
Au	0.41411	2.44123	-0.41553
Cl	0.59061	4.67882	-0.61870
C	-1.80584	-1.79452	-1.02598
C	-1.94148	-2.66983	-2.09634
C	-1.91138	-2.26077	0.28444
C	-2.18823	-4.01316	-1.85474
H	-1.84530	-2.30091	-3.10965
C	-2.15492	-3.60191	0.51597
H	-1.81328	-1.57756	1.12103
C	-2.29405	-4.47792	-0.55421
H	-2.29068	-4.69635	-2.68928
H	-2.23938	-3.96563	1.53323
H	-2.48481	-5.52884	-0.36902
C	2.47812	0.19085	0.77641
C	3.07506	-0.72906	-0.08774
C	3.26990	0.88851	1.69107
C	4.44813	-0.91907	-0.05708
H	2.46740	-1.32321	-0.76331
C	4.64251	0.70722	1.70529
H	2.81012	1.58817	2.38204
C	5.23252	-0.19532	0.83020
H	4.90315	-1.64019	-0.72640
H	5.25358	1.26762	2.40327
H	6.30625	-0.34302	0.84845
Cl	-2.89889	0.89313	-0.22723
S	-1.56959	0.47596	-3.23452

R = Mes

P	0.73478	0.44223	1.27528
C	0.10278	0.27250	-0.32355
P	-1.46203	-0.03796	-1.04649
Pb	1.38638	0.52021	-2.40369
Cl	1.41419	-1.88380	-3.18538
Au	0.16511	2.41453	0.05321
Cl	0.11038	4.67403	-0.06334
C	-1.84608	-1.81272	-1.06012
C	-2.13623	-2.52645	-2.24906
C	-1.85078	-2.49359	0.17833
C	-2.34017	-3.89674	-2.15782
C	-2.06551	-3.86638	0.19335
C	-2.28911	-4.59480	-0.96088
H	-2.55901	-4.43803	-3.07247
H	-2.06934	-4.37392	1.15325
C	2.53974	0.27092	1.00953
C	3.05043	-0.97361	0.58365
C	3.42110	1.30666	1.36286
C	4.42106	-1.10402	0.40411
C	4.78529	1.12152	1.16611
C	5.30476	-0.06217	0.66255
H	4.81137	-2.06215	0.07435
H	5.46181	1.92959	1.42854
Cl	-3.07093	0.79632	-0.06032
C	2.95177	2.60256	1.95356
H	2.65160	3.32149	1.18531
H	2.09061	2.46674	2.61308
H	3.75066	3.06172	2.53803
C	2.18273	-2.18702	0.40876
H	1.57453	-2.36281	1.30153
H	1.50650	-2.11463	-0.44456
H	2.80189	-3.07105	0.25493
C	6.77335	-0.22252	0.42005
H	7.10487	-1.23916	0.63984
H	7.01496	-0.02228	-0.62855
H	7.35635	0.47002	1.02910
C	-1.65544	-1.85777	1.52364
H	-0.61807	-1.95363	1.85601
H	-1.91712	-0.80427	1.56479
H	-2.27367	-2.37515	2.25937
C	-2.47710	-6.07817	-0.92489
H	-3.25123	-6.39762	-1.62534
H	-1.55134	-6.58678	-1.21112
H	-2.74958	-6.42305	0.07350
C	-2.27362	-1.93611	-3.62108
H	-2.96875	-1.09755	-3.64649
H	-1.31485	-1.58209	-4.00113
H	-2.64291	-2.70394	-4.30157
S	-1.33097	0.95629	-2.77610

R = Mes*

P	0.64543	0.20898	1.14854
C	0.38950	0.06861	-0.56152
P	-1.08713	-0.09066	-1.56075
Pb	2.10879	0.52683	-2.32226
Cl	3.01678	-1.58429	-3.42441
Au	0.49275	2.23234	-0.17299
Cl	0.52481	4.48832	-0.42055
C	2.49029	0.06022	1.26052
C	3.14547	-1.10295	0.76509
C	3.28369	1.16775	1.64493
C	4.45146	-0.97577	0.31411
C	4.59748	1.21888	1.16932
C	5.17774	0.20713	0.42579
H	4.93337	-1.84525	-0.11282
H	5.18473	2.09481	1.40512
C	2.92770	2.25125	2.68506
C	2.63549	-2.54246	0.97173
C	6.57074	0.31390	-0.18435
C	3.86145	1.98218	3.88291
H	3.69064	0.98515	4.29718
H	4.91396	2.05511	3.60606
H	3.66654	2.71696	4.66880
C	3.19178	3.66850	2.16809
H	2.56153	3.91233	1.31194
H	2.96976	4.38952	2.95954
H	4.23339	3.81694	1.87897
C	1.50971	2.20383	3.24561
H	1.44574	2.89867	4.08735
H	0.75427	2.52064	2.52424
H	1.24491	1.21244	3.62101
C	2.96769	-3.46760	-0.20154
H	2.59026	-3.07927	-1.14668
H	4.04159	-3.62415	-0.31351
H	2.52192	-4.45005	-0.02471
C	3.38954	-3.04797	2.21778
H	4.46989	-3.01910	2.06580
H	3.15544	-2.43881	3.09466
H	3.10020	-4.08137	2.43050
C	1.15029	-2.68162	1.28118
H	0.84128	-2.06198	2.13122
H	0.52189	-2.44707	0.42427
H	0.94442	-3.71719	1.56611

C	7.23356	1.65515	0.11742
H	6.64555	2.49405	-0.26302
H	7.39046	1.80169	1.18937
H	8.21270	1.69348	-0.36572
C	6.45053	0.16336	-1.70581
H	7.44185	0.19170	-2.16673
H	5.97081	-0.77572	-1.98855
H	5.85990	0.97880	-2.13203
C	7.47059	-0.79923	0.36544
H	7.55654	-0.73305	1.45298
H	7.09270	-1.79228	0.11556
H	8.47362	-0.71210	-0.06084
C	-2.32677	-1.47324	-1.65299
C	-2.30182	-2.30477	-2.81448
C	-3.55965	-1.35816	-0.95273
C	-3.50896	-2.55935	-3.44595
C	-1.10676	-3.14636	-3.30165
C	-4.72790	-1.68009	-1.63691
C	-3.73969	-1.11224	0.55771
C	-4.73843	-2.16866	-2.93264
H	-3.48197	-3.12249	-4.37056
C	-0.65923	-2.83335	-4.73059
C	-1.58266	-4.61554	-3.27294
C	0.06770	-3.09300	-2.34017
H	-5.66737	-1.53893	-1.12152
C	-2.45442	-0.80483	1.30693
C	-4.24676	-2.45892	1.11605
C	-4.77584	-0.03405	0.88586
C	-6.01471	-2.37910	-3.73557
H	0.06418	-3.58518	-5.05605
H	-1.50288	-2.85684	-5.42466
H	-0.18328	-1.85822	-4.80076
H	-2.34004	-4.82837	-4.02806
H	-0.72594	-5.26483	-3.47209
H	-1.99214	-4.88135	-2.29534
H	0.50875	-2.10311	-2.25316
H	-0.23528	-3.43970	-1.34955
H	0.86481	-3.74512	-2.70052
H	-1.67266	-1.53065	1.07281
H	-2.08299	0.19856	1.11024
H	-2.63843	-0.86247	2.38257
H	-4.37167	-2.37652	2.19932
H	-5.20582	-2.74787	0.68455
H	-3.53100	-3.25984	0.91493
H	-5.75746	-0.25891	0.46593
H	-4.89431	0.03219	1.97065
H	-4.46582	0.94455	0.52049
C	-7.25102	-1.87062	-2.99900
C	-6.20471	-3.87245	-4.02752
C	-5.89069	-1.61450	-5.05915
H	-7.43430	-2.42585	-2.07546
H	-7.16730	-0.80917	-2.75309
H	-8.13082	-1.99603	-3.63442
H	-5.37574	-4.28255	-4.60752
H	-6.28401	-4.44635	-3.10095
H	-7.12152	-4.02700	-4.60296
H	-5.76560	-0.54367	-4.88241
H	-5.03607	-1.95817	-5.64521
H	-6.79174	-1.75866	-5.66153
Cl	-2.40696	1.45609	-1.14480
S	-0.43732	0.35482	-3.39961

[R-P=C(Pb(II)Cl)-P(S)MeR](AuCl)

Coordination through Pb(II) atom

R= H

P	0.85630	0.64014	0.55373
C	0.41450	0.12878	-0.96024
P	-1.25216	-0.11359	-1.49080
Pb	1.41507	-0.00147	-3.02438
Cl	1.22481	-2.44876	-3.17166
C	-2.51333	0.65878	-0.46736
H	-3.49858	0.41644	-0.86924
H	-2.43284	0.28585	0.55623
H	-2.36974	1.73957	-0.48133
Au	3.18987	1.39802	-4.29998
Cl	4.74142	2.66690	-5.33465
H	2.26486	0.75223	0.33830
H	-1.51415	-1.49433	-1.38813
S	-1.25679	0.50006	-3.39456

R = Me

P	0.82398	0.29423	0.65593
C	0.37355	-0.05441	-0.89769
P	-1.30842	-0.20109	-1.42025
Pb	1.37009	-0.06773	-2.96373
Cl	1.32766	-2.52303	-3.25852
C	-2.46132	0.78291	-0.44498
H	-3.47117	0.64698	-0.83637
H	-2.42692	0.46592	0.59960
H	-2.18284	1.83394	-0.51915
C	-1.83390	-1.91595	-1.26354
H	-1.85136	-2.20288	-0.20993
H	-2.82744	-2.02962	-1.70120
H	-1.12203	-2.54049	-1.80643
C	2.65332	0.39187	0.63281
H	3.09165	0.25387	-0.35590
H	2.95857	1.36189	1.03469
H	3.04470	-0.37228	1.31084
Au	3.15936	1.39051	-4.15192
Cl	4.74431	2.70589	-5.07883
S	-1.26494	0.39221	-3.34572

R = *t*-Bu

P	0.75755	-0.07018	0.61755
C	0.47016	-0.12045	-1.01401
P	-1.18969	-0.32041	-1.63050
Pb	1.55925	0.32601	-2.99226
Cl	2.32487	-1.96300	-3.52234
C	-2.31647	0.81973	-0.79378
H	-3.32312	0.72068	-1.20142
H	-2.32406	0.62592	0.27981
H	-1.95766	1.83273	-0.97664
Au	2.85480	2.38157	-3.91731
Cl	3.97177	4.22151	-4.61149
C	2.58356	0.13647	0.96060
C	2.96474	-1.11185	1.76495
C	3.48401	0.27499	-0.25089
C	2.69163	1.37429	1.85603
H	2.31407	-1.25325	2.63247
H	2.92036	-2.01528	1.15193
H	3.99009	-1.00462	2.13300
H	3.27446	1.19008	-0.81210
H	4.52810	0.34274	0.07212
H	3.41350	-0.59118	-0.91527
H	3.71973	1.47261	2.21842

H	2.43922	2.28875	1.31372
H	2.03671	1.30324	2.72886
C	-1.85373	-2.03683	-1.39139
C	-3.17194	-2.14387	-2.15866
C	-0.84280	-3.03034	-1.95705
C	-2.08482	-2.30304	0.09421
H	-3.93932	-1.47265	-1.76629
H	-3.03679	-1.93752	-3.22182
H	-3.54892	-3.16558	-2.05882
H	0.10842	-2.99574	-1.42398
H	-1.25273	-4.03946	-1.85805
H	-0.63991	-2.84765	-3.01327
H	-2.41197	-3.33978	0.21365
H	-1.17428	-2.17516	0.68382
H	-2.86312	-1.66331	0.51518
S	-1.05935	0.14664	-3.59158

R = Ph

P	0.78295	0.37194	0.66198
C	0.36338	-0.10789	-0.86942
P	-1.29088	-0.25723	-1.46729
Pb	1.50582	-0.18791	-2.84371
Cl	1.63196	-2.64640	-3.07044
C	-2.43283	0.87465	-0.64746
H	-3.42673	0.74789	-1.07930
H	-2.47080	0.66203	0.42256
H	-2.08808	1.89637	-0.80785
Au	3.24565	1.31116	-4.05768
Cl	4.77889	2.64940	-5.04265
C	2.59166	0.32850	0.66344
C	3.25891	1.31300	1.39479
C	3.33511	-0.65001	-0.00383
C	4.64382	1.35417	1.41048
H	2.68960	2.06038	1.93862
C	4.71936	-0.61286	0.02674
H	2.83122	-1.46460	-0.51609
C	5.37363	0.39481	0.72334
H	5.15491	2.13565	1.96054
H	5.28889	-1.37502	-0.49211
H	6.45685	0.42574	0.73796
C	-1.91443	-1.92026	-1.13730
C	-2.22989	-2.77976	-2.18220
C	-2.07166	-2.33840	0.18393
C	-2.71579	-4.04933	-1.90603
H	-2.08366	-2.45482	-3.20552
C	-2.55736	-3.60573	0.45270
H	-1.80044	-1.68452	1.00673
C	-2.88157	-4.46069	-0.59328
H	-2.95703	-4.71984	-2.72227
H	-2.67699	-3.93061	1.47961
H	-3.25730	-5.45494	-0.38057
S	-1.14224	0.14004	-3.43133

R = Mes

P	0.89492	0.16027	1.20728
C	0.35937	-0.22148	-0.31837
P	-1.28093	-0.22234	-1.00744
Pb	1.64663	0.00471	-2.17315
Cl	1.57089	-2.35466	-2.94182
Au	3.66022	1.46351	-2.93399
Cl	5.48054	2.72452	-3.42904
C	2.69819	0.24810	0.93277
C	3.30356	1.51503	0.87467
C	3.46432	-0.91682	0.77021
C	4.65825	1.59596	0.59092
C	4.82130	-0.78196	0.49227
C	5.43295	0.45963	0.37829
H	5.12024	2.57502	0.51003
H	5.41694	-1.67990	0.35816
C	-1.97714	-1.91097	-1.07005
C	-2.28620	-2.58776	-2.27474
C	-2.19499	-2.56765	0.16271
C	-2.76770	-3.88932	-2.19678
C	-2.66687	-3.87438	0.16643
C	-2.95356	-4.56146	-0.99946
H	-3.00096	-4.39913	-3.12590
H	-2.81670	-4.36406	1.12385
C	2.50386	2.77140	1.05798
H	1.81698	2.69744	1.90698
H	3.15917	3.62613	1.22807
H	1.90028	2.99346	0.17197
C	2.86614	-2.28891	0.89072
H	2.15904	-2.34895	1.72301
H	2.32375	-2.57995	-0.01383
H	3.64785	-3.03081	1.05868
C	6.87799	0.58818	0.01395
H	6.97563	1.04326	-0.97649
H	7.40806	1.23222	0.71992
H	7.37404	-0.38287	-0.00525
C	-2.00228	-1.95185	1.51953
H	-1.20611	-1.21208	1.57090
H	-2.92632	-1.47487	1.86102
H	-1.76088	-2.72904	2.24575
C	-2.15105	-2.03015	-3.66172
H	-2.68604	-1.08992	-3.79041
H	-1.10751	-1.84927	-3.92088
H	-2.55469	-2.74981	-4.37445
C	-3.42694	-5.98026	-0.97448
H	-2.59281	-6.66456	-1.15760
H	-3.86158	-6.24026	-0.00824
H	-4.17255	-6.16381	-1.75043
C	-2.45438	0.82093	-0.10600
H	-3.38942	0.83936	-0.66911
H	-2.64359	0.45981	0.90225
H	-2.03732	1.82763	-0.06941
S	-0.91536	0.73058	-2.75153

R = Mes*

P	0.12632	2.18114	1.03975
C	0.48116	0.67365	0.43478
P	2.00861	-0.24587	0.34537
Cl	-1.21391	0.41206	-2.85851
C	3.74489	0.35811	0.20227
C	4.34999	0.34275	-1.08844
C	4.59838	0.29962	1.33757
C	5.64597	-0.14284	-1.18518
C	5.88964	-0.18818	1.15625
C	6.40612	-0.51960	-0.08592
H	6.08504	-0.21507	-2.17240
H	6.51890	-0.29258	2.02939
C	-1.70272	2.24537	0.84193
C	-2.25917	3.02678	-0.19793
C	-2.54865	1.42772	1.63727
C	-3.56682	2.75068	-0.58879
C	-3.83830	1.19437	1.17272
C	-4.35187	1.78873	0.02706
H	-3.98385	3.30080	-1.41656
H	-4.47958	0.53273	1.73813
C	-1.53250	4.22538	-0.83918
C	-2.19067	0.89903	3.04643
C	-5.74963	1.42468	-0.46253
C	4.32164	0.92233	2.72379

C	3.79270	1.00913	-2.36239
C	7.77276	-1.16463	-0.27594
C	-3.45481	0.83613	3.91885
H	-3.16176	0.62815	4.95069
H	-4.00379	1.78033	3.90432
H	-4.13281	0.03653	3.61728
C	-1.23531	1.84996	3.77033
H	-0.23774	1.88960	3.31819
H	-1.62889	2.86871	3.79730
H	-1.08947	1.51012	4.79922
C	-1.58965	-0.50845	3.02530
H	-2.25212	-1.21455	2.51810
H	-0.62233	-0.52056	2.52415
H	-1.44411	-0.86581	4.04911
C	-6.77835	1.74218	0.62941
H	-7.78254	1.48336	0.28287
H	-6.58916	1.17708	1.54417
H	-6.76854	2.80595	0.88085
C	-6.13782	2.19166	-1.72395
H	-7.13134	1.87401	-2.04886
H	-6.17839	3.27067	-1.55216
H	-5.44378	1.99705	-2.54525
C	-5.79073	-0.07476	-0.77982
H	-5.07648	-0.32752	-1.56714
H	-5.55524	-0.68813	0.09201
H	-6.78888	-0.35837	-1.12454
C	-2.43249	4.97980	-1.82093
H	-3.35042	5.33826	-1.34957
H	-1.88994	5.85295	-2.19039
H	-2.69803	4.36925	-2.68702
C	-1.15507	5.21524	0.27267
H	-2.04238	5.54317	0.81973
H	-0.45651	4.78190	0.99191
H	-0.67620	6.09747	-0.16156
C	-0.27825	3.82635	-1.61729
H	0.51624	3.45853	-0.96318
H	-0.49140	3.04775	-2.35075
H	0.12526	4.70168	-2.13504
C	2.99441	1.66409	2.82852
H	2.88712	2.39490	2.02481
H	2.11484	1.02446	2.81846
H	2.96421	2.21222	3.77347
C	5.39609	2.01700	2.90480
H	5.21787	2.54455	3.84576
H	6.40605	1.60841	2.93625
H	5.35079	2.74546	2.09210
C	4.47457	-0.07267	3.87818
H	5.46451	-0.53256	3.88252
H	4.35196	0.44946	4.83077
H	3.74041	-0.87735	3.84279
C	2.44831	1.69768	-2.17444
H	2.24091	2.31727	-3.05009
H	1.61603	1.00290	-2.08581
H	2.46422	2.35632	-1.30303
C	3.72361	0.06025	-3.56089
H	2.99931	-0.73576	-3.39454
H	3.41632	0.62005	-4.44807
H	4.69238	-0.39470	-3.77835
C	4.79290	2.13764	-2.69452
H	4.88392	2.83963	-1.86172
H	5.78891	1.75963	-2.92792
H	4.43039	2.68926	-3.56587
C	8.43810	-1.51139	1.05323
H	8.65442	-0.61999	1.64773
H	7.82012	-2.18504	1.65222
H	9.38892	-2.01485	0.86410
C	8.69447	-0.20933	-1.04347
H	8.29947	0.03036	-2.03254
H	8.82958	0.72808	-0.49836
H	9.67750	-0.66782	-1.18078
C	7.59795	-2.45799	-1.08120
H	6.95549	-3.16564	-0.55178
H	7.15069	-2.26807	-2.05879
H	8.56898	-2.93389	-1.24246
Au	-2.65514	-2.57591	-0.08806
Cl	-4.20452	-4.09478	0.57387
Pb	-0.88005	-0.77692	-0.71190
S	1.65340	-1.54425	-1.18190
C	2.01090	-1.36410	1.78058
H	2.02722	-0.86378	2.74331
H	1.10282	-1.96255	1.70004
H	2.87008	-2.02869	1.68299

Coordination through S atom

R= H

P	0.91170	0.24836	0.96279
C	0.65282	-0.10507	-0.63681
P	-0.85325	0.23811	-1.45726
Pb	1.98097	-0.71173	-2.53358
Cl	0.64164	-2.78671	-2.84352
C	-2.10843	1.21380	-0.61881
H	-2.96774	1.32480	-1.28305
H	-2.41236	0.70041	0.29633
H	-1.70252	2.19601	-0.37545
Au	-1.94121	0.75312	-4.61362
Cl	-3.70216	0.48076	-5.99315
H	2.28146	-0.15982	1.06164
H	-1.45647	-0.99607	-1.75074
S	-0.19418	1.09684	-3.19094

R = Me

P	0.86716	0.15174	0.96534
C	0.62387	-0.11567	-0.65054
P	-0.94154	0.17343	-1.38162
Pb	1.99349	-0.48318	-2.58417
Cl	1.20891	-2.83302	-2.91958
C	-2.03369	1.32392	-0.52738
H	-2.94151	1.45393	-1.12007
H	-2.28989	0.92633	0.45710
H	-1.53101	2.28430	-0.41482
C	-1.84323	-1.37072	-1.54927
H	-2.08328	-1.74165	-0.54979
H	-2.75617	-1.19688	-2.12182
H	-1.21070	-2.09319	-2.06815
C	2.62534	-0.27763	1.27765
H	3.17295	-0.55983	0.37778
H	3.11944	0.57054	1.75964
H	2.65518	-1.10911	1.98815
Au	-2.12659	0.49286	-4.61927
Cl	-3.87534	0.10516	-5.99009
S	-0.39783	0.94817	-3.20640

R = *t*-Bu

P	0.86972	-0.04154	0.81879
C	0.65893	-0.14041	-0.82515
P	-0.97013	0.10983	-1.45422
Pb	2.07449	-0.05617	-2.76175
Cl	2.14454	-2.52169	-3.20152
C	-1.91796	1.33441	-0.52176
H	-2.82109	1.59405	-1.07493
H	-2.18099	0.94806	0.46448

H	-1.29588	2.22191	-0.40724
Au	-2.29520	0.72144	-4.74108
Cl	-4.02518	0.59389	-6.18587
S	-0.54683	0.91833	-3.29868
C	-1.99106	-1.44771	-1.50063
C	-3.44778	-1.08387	-1.78889
C	-1.45989	-2.38923	-2.57499
C	-1.89036	-2.11403	-0.12739
H	-3.90536	-0.52090	-0.97327
H	-3.56123	-0.51998	-2.71875
H	-4.01158	-2.01320	-1.90571
H	-0.40961	-2.64422	-2.42582
H	-2.04142	-3.31476	-2.53710
H	-1.57309	-1.96535	-3.57417
H	-2.57038	-2.97039	-0.11889
H	-0.88355	-2.48044	0.07306
H	-2.18761	-1.45269	0.69049
C	2.68581	-0.33573	1.19623
C	3.11860	-1.70937	0.69424
C	3.54811	0.77046	0.59740
C	2.79330	-0.28572	2.72176
H	2.52169	-2.50641	1.14539
H	3.02635	-1.81295	-0.38890
H	4.16509	-1.88768	0.96447
H	3.21960	1.76366	0.91466
H	4.58734	0.64414	0.91924
H	3.54709	0.75494	-0.49579
H	3.82889	-0.46176	3.02805
H	2.48602	0.68597	3.11779
H	2.17060	-1.05014	3.19372

R = Ph

P	0.83316	0.50304	0.82893
C	0.57693	-0.11329	-0.68961
P	-0.94574	0.01085	-1.54160
Pb	2.17143	-0.71269	-2.34372
Cl	1.67507	-3.16357	-2.34260
C	-2.05531	1.31661	-0.97891
H	-2.94032	1.32048	-1.61773
H	-2.35075	1.14664	0.05769
H	-1.53753	2.27259	-1.05976
Au	-2.09011	0.22911	-4.83966
Cl	-3.85671	0.11325	-6.24000
C	2.56049	0.04566	1.16810
C	3.11445	-1.17388	0.76337
C	3.35133	0.94378	1.88603
C	4.44333	-1.46377	1.03532
H	2.48955	-1.92051	0.28024
C	4.68232	0.65577	2.14579
H	2.92404	1.88020	2.23136
C	5.23032	-0.54527	1.71711
H	4.85937	-2.41576	0.72572
H	5.29290	1.36818	2.68865
H	6.26853	-0.77336	1.92912
C	-1.84825	-1.54154	-1.38244
C	-2.44203	-1.82974	-0.15277
C	-1.93108	-2.45463	-2.42620
C	-3.13027	-3.01735	0.01895
H	-2.36067	-1.13741	0.67851
C	-2.62325	-3.64245	-2.24697
H	-1.45688	-2.24132	-3.37558
C	-3.22401	-3.92232	-1.03051
H	-3.59339	-3.23780	0.97353
H	-2.68968	-4.34899	-3.06532
H	-3.76528	-4.85160	-0.89504
S	-0.30221	0.41206	-3.43624

R = Mes

P	0.73072	0.16466	1.24070
C	0.42645	-0.22597	-0.34213
P	-1.06808	-0.07328	-1.25701
Pb	2.25585	-0.36755	-1.82292
Cl	2.02248	-2.82504	-2.27702
Au	-1.90540	1.49639	-4.31531
Cl	-3.54567	2.31207	-5.63607
C	2.55200	-0.07853	1.31280
C	3.09711	-1.36678	1.45760
C	3.39480	1.04713	1.28214
C	4.48081	-1.50122	1.51734
C	4.76923	0.86071	1.34654
C	5.33310	-0.40614	1.45447
H	4.90240	-2.49562	1.62951
H	5.41837	1.73111	1.31944
C	-1.94960	-1.67122	-1.33959
C	-2.56844	-2.11693	-0.14902
C	-2.02198	-2.47964	-2.49979
C	-3.25170	-3.32678	-0.14838
C	-2.73110	-3.67277	-2.42953
C	-3.35879	-4.11840	-1.27754
H	-3.71159	-3.65625	0.77831
H	-2.78160	-4.28374	-3.32481
C	2.83283	2.43432	1.17121
H	2.38777	2.61521	0.18763
H	2.04423	2.60829	1.90974
H	3.61046	3.18298	1.32606
C	2.22891	-2.58547	1.56343
H	1.38102	-2.41607	2.23331
H	1.82069	-2.87370	0.59027
H	2.80118	-3.43064	1.94765
C	6.81955	-0.58428	1.49308
H	7.22480	-0.67241	0.48020
H	7.31063	0.26717	1.96751
H	7.09811	-1.48918	2.03535
C	-2.56900	-1.38055	1.16152
H	-1.66565	-0.80122	1.34835
H	-3.42668	-0.70553	1.23326
H	-2.66093	-2.09635	1.97934
C	-1.39172	-2.17619	-3.82561
H	-1.80540	-1.27563	-4.28184
H	-0.31366	-2.04304	-3.74287
H	-1.57279	-3.00802	-4.50618
C	-4.12606	-5.40247	-1.26381
H	-4.30901	-5.74808	-0.24552
H	-5.09701	-5.27493	-1.75194
H	-3.59322	-6.18717	-1.80476
S	-0.24741	0.70956	-2.95963
C	-2.22797	1.18979	-0.67489
H	-1.71538	2.14931	-0.74723
H	-3.09186	1.19745	-1.34239
H	-2.54959	1.01928	0.34913

R = Mes*

P	-1.76371	2.14815	0.27786
C	-0.88200	0.74254	0.27600
P	0.85874	0.49828	0.35793
C	-3.49077	1.48472	0.32616
C	-4.28181	1.53134	-0.85183
C	-3.97680	0.79387	1.46044
C	-5.36199	0.66545	-0.94148
C	-5.05828	-0.07380	1.28362
C	-5.72689	-0.20563	0.07899
H	-5.94027	0.65210	-1.85337

H	-5.40167	-0.64696	2.13216
C	-3.49597	1.01959	2.91317
C	-4.04860	2.54741	-1.98630
C	-6.86545	-1.19726	-0.13568
C	-2.74335	2.30237	-2.74547
H	-1.86295	2.49885	-2.13001
H	-2.67524	1.27315	-3.10076
H	-2.68668	2.97975	-3.60280
C	-4.04561	3.96207	-1.38963
H	-4.98722	4.16968	-0.87508
H	-3.23096	4.11040	-0.67780
H	-3.92316	4.70017	-2.18722
C	-5.17265	2.50761	-3.02461
H	-5.19555	1.56156	-3.57047
H	-6.15482	2.68035	-2.57798
H	-4.99914	3.30017	-3.75592
C	-2.50199	-0.05001	3.37252
H	-1.57110	0.00915	2.80804
H	-2.26309	0.08675	4.43140
H	-2.92034	-1.05325	3.25442
C	-2.87956	2.40488	3.11106
H	-1.92552	2.53274	2.59177
H	-3.55481	3.19366	2.77170
H	-2.67517	2.56491	4.17319
C	-4.70282	0.96929	3.86649
H	-5.50134	1.63727	3.53621
H	-5.11805	-0.03362	3.97307
H	-4.38318	1.28686	4.86197
C	-7.10220	-2.08067	1.08631
H	-7.41118	-1.49933	1.95904
H	-7.90041	-2.79455	0.87013
H	-6.21017	-2.65436	1.35125
C	-6.52317	-2.10224	-1.32528
H	-6.37950	-1.52955	-2.24310
H	-5.60338	-2.66292	-1.14387
H	-7.33268	-2.81703	-1.49739
C	-8.16108	-0.43391	-0.43556
H	-8.06997	0.18181	-1.33249
H	-8.98283	-1.13719	-0.59658
H	-8.43159	0.22122	0.39640
C	2.28536	1.54062	-0.14347
C	2.89369	1.26956	-1.39770
C	3.08425	2.14468	0.87188
C	4.28375	1.20229	-1.44056
C	4.46000	2.04202	0.75361
C	5.08882	1.46453	-0.34557
H	4.74620	0.92342	-2.37708
H	5.07404	2.44562	1.54946
C	2.18007	1.23891	-2.76263
C	2.49749	-0.00713	-3.59267
C	2.72812	2.47188	-3.51403
H	2.10491	-0.90907	-3.12537
H	2.03740	0.09106	-4.57944
H	3.57050	-0.13951	-3.74241
H	2.52401	3.39189	-2.96035
H	3.80396	2.40954	-3.68151
H	2.23730	2.54422	-4.48830
C	2.57080	3.08863	1.98056
C	3.22938	4.45564	1.69803
C	2.98764	2.65374	3.38850
H	2.98615	4.80483	0.69202
H	2.85639	5.19239	2.41495
H	4.31498	4.41901	1.78878
H	2.54004	1.70616	3.68720
H	4.07049	2.54319	3.46895
H	2.68004	3.41225	4.11339
C	6.59339	1.23578	-0.34170
C	7.31971	2.58073	-0.21954
C	7.07084	0.53782	-1.61224
H	7.05587	3.10356	0.70242
H	8.40118	2.42031	-0.21149
H	7.07953	3.23650	-1.06041
H	6.57337	-0.42475	-1.75187
H	6.90765	1.14948	-2.50367
H	8.14422	0.34835	-1.53981
Cl	-2.36638	-1.41904	-2.34519
Au	2.89939	-2.31662	0.16995
Cl	4.80257	-3.28346	0.92914
C	1.07098	3.35829	1.92814
H	0.76334	3.69676	0.93675
H	0.45056	2.50549	2.19555
H	0.82501	4.15267	2.63733
C	0.67148	1.43517	-2.69504
H	0.13209	0.56454	-2.32436
H	0.41482	2.30309	-2.08280
H	0.29437	1.62211	-3.70342
C	6.95052	0.34851	0.85794
H	6.67343	0.81906	1.80402
H	6.44528	-0.61873	0.80183
H	8.02928	0.17109	0.87862
Pb	-1.71288	-1.50255	0.07826
S	1.03134	-1.30644	-0.66203
C	1.19474	-0.00298	2.07420
H	2.25426	-0.24155	2.17097
H	0.90162	0.75180	2.79843
H	0.62159	-0.91335	2.25175

Coordination through P(sp^2) atom

R= H

P	0.75718	0.09420	0.83287
C	0.48732	-0.28988	-0.73305
P	-1.02087	-0.13508	-1.61567
Pb	1.89471	-0.71218	-2.65428
Cl	0.97899	-3.03265	-2.83156
C	-2.32983	0.84599	-0.86911
H	-3.19280	0.83465	-1.53802
H	-2.60971	0.42598	0.09953
H	-1.98548	1.87239	-0.73948
Au	-0.52453	0.84010	2.46611
Cl	-1.86223	1.60882	4.11644
H	2.13550	-0.11927	1.05756
H	-1.54616	-1.44046	-1.67387
S	-0.48176	0.52103	-3.42556

R = Me

P	0.80617	0.17056	0.78812
C	0.46042	-0.18271	-0.76762
P	-1.14381	-0.21203	-1.48928
Pb	1.74416	-0.62114	-2.76015
Cl	1.39102	-3.10101	-2.52252
C	-2.28180	1.03953	-0.86777
H	-3.21644	0.96636	-1.42776
H	-2.47639	0.88650	0.19614
H	-1.84754	2.02714	-1.02303
C	-1.89032	-1.81097	-1.13366
H	-2.06898	-1.90354	-0.06008
H	-2.83168	-1.89384	-1.68018
H	-1.20145	-2.58918	-1.46764
C	2.58305	0.05881	1.11958
H	3.13771	-0.23851	0.23002

H	2.94143	1.02497	1.48205
H	2.74262	-0.67380	1.91432
Au	-0.53742	0.71651	2.46035
Cl	-1.94807	1.26301	4.14493
S	-0.82784	0.03906	-3.46053

R = *t*-Bu

P	0.74025	0.17411	0.81752
C	0.47854	-0.11919	-0.77291
P	-1.12410	-0.24991	-1.51087
Pb	1.82239	0.07084	-2.78429
Cl	2.43806	-2.36620	-2.92968
C	-2.27890	0.99234	-0.88541
H	-3.19265	0.96195	-1.47996
H	-2.50963	0.83099	0.16898
H	-1.81298	1.97061	-1.00588
Au	-0.73899	0.53616	2.43992
Cl	-2.27507	0.92651	4.06386
C	2.51954	0.19166	1.34134
C	2.67483	-0.98619	2.31040
C	3.48858	0.04562	0.18115
C	2.76013	1.50677	2.08511
H	1.96532	-0.92640	3.13935
H	2.53776	-1.94479	1.80489
H	3.68444	-0.96586	2.73188
H	3.44093	0.90637	-0.49215
H	4.51032	0.00585	0.57116
H	3.31968	-0.87406	-0.38470
H	3.77410	1.50349	2.49607
H	2.66611	2.37079	1.42279
H	2.06214	1.63623	2.91571
C	-1.88765	-1.92006	-1.22553
C	-3.22383	-1.94514	-1.96979
C	-0.95779	-2.98814	-1.79289
C	-2.11154	-2.15968	0.26434
H	-3.95519	-1.26174	-1.53326
H	-3.10223	-1.70536	-3.02818
H	-3.63714	-2.95517	-1.90088
H	0.01632	-2.99139	-1.30174
H	-1.42047	-3.96745	-1.64005
H	-0.78889	-2.85313	-2.86190
H	-2.58451	-3.13829	0.38795
H	-1.17373	-2.17856	0.82176
H	-2.77061	-1.41969	0.72258
S	-0.83148	0.07077	-3.48191

R = Ph

P	0.74735	0.39098	0.56981
C	0.53343	-0.35643	-0.86909
P	-0.98526	-0.57674	-1.72618
Pb	2.07793	-1.01663	-2.58150
Cl	2.09301	-3.44872	-1.92452
C	-2.10176	0.83577	-1.61552
H	-2.99064	0.61980	-2.21089
H	-2.38863	1.03732	-0.58194
H	-1.59179	1.70800	-2.02533
Au	-0.70776	1.47999	1.84043
Cl	-2.24930	2.56564	3.09771
C	2.45103	0.26770	1.10859
C	2.98340	1.29734	1.88526
C	3.23686	-0.84344	0.79102
C	4.30242	1.23716	2.30334
H	2.36271	2.14499	2.15709
C	4.55183	-0.89968	1.22175
H	2.80901	-1.68148	0.24893
C	5.08679	0.14205	1.96870
H	4.71663	2.04334	2.89726
H	5.15553	-1.76791	0.98458
H	6.11609	0.09225	2.30502
C	-1.85913	-1.98629	-1.01025
C	-2.56190	-1.83167	0.18515
C	-1.79714	-3.23159	-1.62559
C	-3.20890	-2.91757	0.74917
H	-2.60331	-0.87167	0.68849
C	-2.44508	-4.31538	-1.05278
H	-1.23731	-3.34779	-2.54581
C	-3.15151	-4.15914	0.12956
H	-3.75685	-2.79317	1.67576
H	-2.39201	-5.28536	-1.53284
H	-3.65763	-5.00819	0.57468
S	-0.46829	-0.89558	-3.64185

R = Mes

P	0.72674	0.58618	0.93302
C	0.42157	-0.42753	-0.31504
P	-0.97773	-0.57477	-1.37535
Pb	2.19508	-1.11751	-1.76093
Cl	1.64731	-3.56963	-1.57751
Au	-0.60250	1.96865	2.04899
Cl	-1.98004	3.37636	3.17328
C	2.48657	0.48978	1.33493
C	3.31640	1.57866	1.01860
C	3.00531	-0.64671	1.97800
C	4.66660	1.49605	1.32953
C	4.36373	-0.67610	2.26907
C	5.21117	0.37736	1.94913
H	5.31117	2.33460	1.08387
H	4.76896	-1.54893	2.77178
C	-1.85127	-2.15592	-1.10560
C	-2.25118	-2.46613	0.21285
C	-2.18306	-3.04371	-2.15402
C	-2.93523	-3.65089	0.45541
C	-2.86686	-4.21267	-1.84192
C	-3.24269	-4.54873	-0.55153
H	-3.23841	-3.86961	1.47466
H	-3.11777	-4.88680	-2.65466
C	2.77800	2.81239	0.35763
H	2.27895	2.58209	-0.58847
H	2.04131	3.31571	0.99145
H	3.57981	3.52090	0.14852
C	2.14155	-1.80896	2.36971
H	1.24909	-1.47680	2.90846
H	1.80247	-2.37795	1.49924
H	2.69052	-2.48973	3.02078
C	6.67614	0.30314	2.24916
H	7.22239	-0.11964	1.40011
H	7.09307	1.29232	2.44558
H	6.87386	-0.33267	3.11357
C	-1.97671	-1.61403	1.41725
H	-2.68576	-1.85617	2.20949
H	-0.97243	-1.80247	1.80355
H	-2.05058	-0.54243	1.23824
C	-1.86598	-2.84324	-3.60741
H	-2.18724	-1.87229	-3.98358
H	-0.79377	-2.91903	-3.79282
H	-2.36791	-3.61485	-4.19220
C	-3.94332	-5.83802	-0.25964
H	-4.60716	-6.12469	-1.07731
H	-3.21687	-6.64641	-0.13089
H	-4.53093	-5.77475	0.65756
C	-2.23475	0.71789	-1.22002
H	-2.80333	0.63517	-0.29439

H	-1.74845	1.69247	-1.27244
H	-2.91526	0.60925	-2.06742
S	-0.05475	-0.29251	-3.14914

R = Mes*

P	-0.84475	1.09382	0.09400
C	-0.20042	-0.37871	0.41048
P	1.41168	-1.06356	0.61741
Cl	-2.12530	-2.61148	-2.08067
C	3.07844	-0.42702	0.14677
C	3.63505	-0.89056	-1.08142
C	3.97259	0.02424	1.15654
C	4.97439	-1.25196	-1.08634
C	5.30113	-0.38589	1.07771
C	5.80950	-1.11096	0.01337
H	5.38512	-1.65452	-2.00366
H	5.96639	-0.10018	1.88051
C	-2.65740	0.87193	0.14150
C	-3.40742	0.76616	-1.05888
C	-3.25932	0.53754	1.38240
C	-4.62553	0.09548	-0.99674
C	-4.46638	-0.15280	1.34737
C	-5.14214	-0.44065	0.17150
H	-5.17874	-0.04161	-1.91036
H	-4.90514	-0.46799	2.28488
C	-3.01530	1.42663	-2.39480
C	-2.75975	0.94691	2.78682
C	-6.41964	-1.27300	0.20431
C	3.68668	1.09094	2.23742
C	2.96231	-0.84861	-2.46881
C	7.23449	-1.64814	-0.02532
C	-3.95394	1.49688	3.58943
H	-3.59564	1.87386	4.55041
H	-4.43666	2.32148	3.06014
H	-4.71112	0.74296	3.80389
C	-1.72821	2.07434	2.78426
H	-0.74073	1.75758	2.44693
H	-2.04770	2.92011	2.17105
H	-1.59509	2.43870	3.80640
C	-2.18559	-0.25727	3.53815
H	-2.91179	-1.07143	3.59973
H	-1.28885	-0.63329	3.04204
H	-1.91386	0.02822	4.55860
C	-7.46935	-0.57554	1.07721
H	-8.38758	-1.16857	1.10560
H	-7.12531	-0.44702	2.10561
H	-7.71487	0.41216	0.67944
C	-7.01146	-1.48109	-1.18722
H	-7.90489	-2.10548	-1.11174
H	-7.31008	-0.53657	-1.64902
H	-6.30876	-1.98579	-1.85446
C	-6.09724	-2.65013	0.79743
H	-5.36121	-3.17503	0.18324
H	-5.70006	-2.57139	1.81198
H	-7.00215	-3.26247	0.84037
C	-4.03962	1.14367	-3.49810
H	-5.03728	1.51080	-3.24656
H	-3.72216	1.66588	-4.40308
H	-4.10124	0.07902	-3.73605
C	-2.99961	2.94654	-2.17948
H	-3.97934	3.30169	-1.84967
H	-2.26281	3.25117	-1.43271
H	-2.74846	3.45398	-3.11462
C	-1.66755	0.95541	-2.94328
H	-0.82052	1.28853	-2.34173
H	-1.62913	-0.13154	-3.02237
H	-1.51474	1.38686	-3.93661
C	2.31908	1.75306	2.11853
H	2.20329	2.21344	1.13533
H	1.46927	1.09407	2.27701
H	2.24018	2.55312	2.85849
C	4.68506	2.24030	1.97319
H	4.46497	3.06786	2.65269
H	5.72055	1.94150	2.13647
H	4.59167	2.61141	0.95069
C	3.93564	0.58956	3.66391
H	4.94903	0.19892	3.77658
H	3.82432	1.41966	4.36657
H	3.24587	-0.19727	3.96700
C	1.53588	-0.31820	-2.47507
H	1.23606	-0.13179	-3.50905
H	0.81564	-1.02308	-2.06328
H	1.46535	0.63269	-1.94048
C	2.99939	-2.19790	-3.19066
H	2.40185	-2.94184	-2.66458
H	2.58864	-2.08029	-4.19691
H	4.01624	-2.58204	-3.29448
C	3.78728	0.16948	-3.28494
H	3.78220	1.15183	-2.80642
H	4.82533	-0.13996	-3.41262
H	3.34284	0.27441	-4.27819
C	7.97949	-1.40650	1.28476
H	8.10986	-0.34106	1.49085
H	7.46375	-1.86211	2.13392
H	8.97570	-1.85095	1.22467
C	8.01318	-0.96681	-1.15699
H	7.55903	-1.15272	-2.13215
H	8.05683	0.11465	-1.00713
H	9.03758	-1.34796	-1.18910
C	7.18517	-3.15927	-0.28114
H	6.64648	-3.67436	0.51789
H	6.68691	-3.39325	-1.22386
H	8.19914	-3.56599	-0.32658
Au	0.17041	3.03199	-0.34479
Cl	1.23666	4.99539	-0.76720
Pb	-1.43076	-2.49094	0.34192
S	1.22460	-2.92095	-0.18593
C	1.50976	-1.45199	2.39539
H	2.44404	-1.98767	2.56632
H	1.43451	-0.59662	3.05865
H	0.68488	-2.13634	2.59638

Coordination through π(C=P) bond

R= H

P	0.71190	0.02870	0.95480
C	0.45534	0.14994	-0.74532
P	-1.12538	-0.01599	-1.52261
Pb	1.72936	-0.11019	-2.80305
Cl	1.22184	-2.57029	-2.76297
C	-2.54588	0.69910	-0.67541
H	-3.45295	0.43910	-1.22450
H	-2.60502	0.30165	0.34060
H	-2.43912	1.78403	-0.64370
Au	0.75511	2.17804	-0.08335
Cl	1.00332	4.41659	-0.07816
H	-1.37607	-1.40197	-1.50561
H	2.13478	-0.11128	0.94769
S	-0.89044	0.66737	-3.38259

R = Me

P	0.77593	0.11547	0.85041
C	0.38322	0.23386	-0.81850
P	-1.23940	-0.09104	-1.45939
Pb	1.57510	0.02904	-2.92687
Cl	1.69411	-2.48431	-2.66900
C	-2.57875	0.84934	-0.69683
H	-3.52692	0.53548	-1.13857
H	-2.59607	0.66389	0.37956
H	-2.42993	1.91294	-0.88387
C	-1.60580	-1.82404	-1.13002
H	-1.70095	-1.98595	-0.05393
H	-2.53703	-2.09032	-1.63308
H	-0.78910	-2.42911	-1.52918
C	2.59783	-0.10104	0.89264
H	3.10398	0.26685	0.00013
H	3.00229	0.39441	1.77756
H	2.78690	-1.17525	0.99054
Au	0.59596	2.27871	-0.17483
Cl	0.71870	4.53031	-0.12203
S	-1.12621	0.27723	-3.42988

R = *t*-Bu

P	0.66781	0.16455	0.74397
C	0.35330	0.28551	-0.94322
P	-1.24373	-0.12621	-1.63615
Pb	1.57779	0.63070	-3.03908
Cl	2.50096	-1.71395	-3.26036
C	-2.59386	0.86952	-0.95208
H	-3.54417	0.53134	-1.36860
H	-2.61651	0.79322	0.13594
H	-2.43515	1.90874	-1.24063
Au	0.34824	2.33850	-0.22240
Cl	0.29712	4.59095	-0.08066
C	2.51430	0.09591	1.05225
C	2.77073	-1.38455	1.36130
C	3.41676	0.55117	-0.07938
C	2.75442	0.93607	2.30720
H	2.12380	-1.75461	2.16200
H	2.62545	-2.01009	0.47694
H	3.80706	-1.50806	1.69159
H	3.20930	1.58261	-0.37776
H	4.46096	0.51258	0.24897
H	3.33727	-0.11155	-0.94508
H	3.78345	0.79377	2.65141
H	2.60782	2.00138	2.11260
H	2.08705	0.64623	3.12382
C	-1.69852	-1.90087	-1.30691
C	-2.87561	-2.25911	-2.21542
C	-0.51088	-2.79768	-1.64013
C	-2.10368	-2.07634	0.15583
H	-3.76241	-1.65453	-2.01174
H	-2.61694	-2.15196	-3.26949
H	-3.14326	-3.30393	-2.03373
H	0.34285	-2.61218	-0.98705
H	-0.81449	-3.84059	-1.51042
H	-0.17294	-2.66708	-2.66880
H	-2.29767	-3.13782	0.33520
H	-1.31847	-1.76420	0.84815
H	-3.01680	-1.53052	0.40117
S	-1.08330	0.22792	-3.61244

R = Ph

P	0.72375	0.37861	0.77424
C	0.24532	0.30548	-0.87592
P	-1.41206	-0.02443	-1.41272
Pb	1.31836	0.25181	-3.06619
Cl	1.53097	-2.26320	-3.21166
C	-2.69582	0.89906	-0.53737
H	-3.66994	0.58656	-0.91791
H	-2.64233	0.70123	0.53493
H	-2.55454	1.96471	-0.72092
Au	0.48533	2.42471	-0.45387
Cl	0.59687	4.67019	-0.65443
C	-1.76789	-1.76632	-1.08052
C	-1.92736	-2.66716	-2.12593
C	-1.88431	-2.20334	0.23925
C	-2.21191	-3.99706	-1.85334
H	-1.81703	-2.32461	-3.14755
C	-2.16826	-3.53145	0.50518
H	-1.74338	-1.51408	1.06554
C	-2.33393	-4.42853	-0.54241
H	-2.32926	-4.69770	-2.67149
H	-2.25713	-3.86834	1.53140
H	-2.55306	-5.46918	-0.33226
C	2.52810	0.16789	0.74943
C	3.15953	-0.74038	-0.10179
C	3.28532	0.87120	1.68834
C	4.53374	-0.91324	-0.03386
H	2.57788	-1.33785	-0.79721
C	4.65922	0.70690	1.74000
H	2.79789	1.56197	2.36929
C	5.28444	-0.18428	0.87777
H	5.01595	-1.62523	-0.69388
H	5.24379	1.27177	2.45691
H	6.35908	-0.31866	0.92492
S	-1.43805	0.40966	-3.36943

R = Mes

P	0.75184	0.40808	1.24836
C	0.09345	0.31669	-0.34595
P	-1.53544	0.01358	-1.00002
Pb	1.32059	0.68983	-2.41581
Cl	1.52042	-1.70126	-3.23796
Au	0.10219	2.43967	0.17351
Cl	-0.12498	4.69364	0.13785
C	-1.85174	-1.78978	-1.03537
C	-2.02567	-2.53272	-2.22838
C	-1.91791	-2.46403	0.20524
C	-2.18180	-3.91092	-2.13369
C	-2.06462	-3.84578	0.22715
C	-2.18021	-4.59576	-0.92947
H	-2.30811	-4.47002	-3.05515
H	-2.09934	-4.34424	1.19127
C	2.55333	0.25880	0.96092
C	3.03854	-0.99883	0.54930
C	3.45265	1.28771	1.28651
C	4.40601	-1.15847	0.36701
C	4.81142	1.07576	1.08448
C	5.30779	-0.12821	0.60316
H	4.77701	-2.12801	0.04829
H	5.50396	1.87670	1.32638
C	3.00584	2.60110	1.85540
H	2.67005	3.29453	1.07835
H	2.17491	2.48346	2.55600
H	3.82691	3.08217	2.38925
C	2.13858	-2.18615	0.36251
H	1.38933	-2.25456	1.15730
H	1.60592	-2.15789	-0.59110
H	2.72496	-3.10574	0.38110

C	6.77264	-0.31698	0.35744
H	7.08231	-1.34331	0.56323
H	7.01793	-0.10733	-0.68848
H	7.37090	0.35469	0.97529
C	-1.91818	-1.80324	1.55525
H	-1.48239	-2.47309	2.29802
H	-1.36525	-0.86856	1.60868
H	-2.94563	-1.60029	1.87425
C	-2.29389	-6.08686	-0.88655
H	-2.97071	-6.45618	-1.65937
H	-1.31784	-6.54975	-1.06193
H	-2.65411	-6.43316	0.08338
C	-2.08987	-1.96934	-3.61794
H	-2.81748	-1.16373	-3.70781
H	-1.12401	-1.57708	-3.93612
H	-2.37402	-2.76363	-4.30915
C	-2.94542	0.75980	-0.13358
H	-3.04889	0.40729	0.89004
H	-2.81522	1.84275	-0.14350
H	-3.84477	0.50430	-0.69870
S	-1.41425	0.99806	-2.75621

R = Mes*

P	0.67171	0.04588	1.11078
C	0.41989	0.07081	-0.60738
P	-1.09329	-0.05353	-1.61641
Pb	2.09438	0.60238	-2.37114
Cl	3.10065	-1.50302	-3.39810
Au	0.44919	2.18071	-0.01555
Cl	0.27929	4.44037	-0.21780
C	2.52413	-0.02892	1.23754
C	3.22640	-1.15954	0.73659
C	3.27627	1.09920	1.64729
C	4.53111	-0.97928	0.29938
C	4.58794	1.20860	1.17814
C	5.21037	0.22842	0.42492
H	5.04664	-1.82611	-0.13443
H	5.14252	2.10048	1.43356
C	2.88326	2.13261	2.72549
C	2.76337	-2.61971	0.90343
C	6.59996	0.39663	-0.17915
C	3.83707	1.86373	3.90788
H	3.71444	0.84555	4.28602
H	4.88295	1.99392	3.62747
H	3.61526	2.55993	4.72140
C	3.08413	3.57699	2.25877
H	2.43449	3.82869	1.41938
H	2.84528	4.26060	3.07811
H	4.11516	3.77512	1.96165
C	1.47479	1.99999	3.29679
H	1.38867	2.65423	4.16859
H	0.69628	2.31380	2.59806
H	1.25786	0.98184	3.62866
C	3.05603	-3.48275	-0.32499
H	2.59919	-3.07644	-1.22562
H	4.12579	-3.57772	-0.51683
H	2.67030	-4.49248	-0.15921
C	3.58556	-3.16455	2.08795
H	4.65647	-3.11931	1.88333
H	3.39164	-2.59244	2.99898
H	3.31663	-4.20853	2.27443
C	1.29878	-2.79977	1.28359
H	1.03635	-2.24503	2.19039
H	0.61951	-2.51480	0.48112
H	1.11517	-3.85615	1.49929
C	7.21267	1.75563	0.14815
H	6.59517	2.57894	-0.21945
H	7.36138	1.88899	1.22306
H	8.19087	1.83829	-0.33144
C	6.48752	0.26917	-1.70332
H	7.47804	0.34031	-2.16159
H	6.03985	-0.68029	-2.00306
H	5.86826	1.06998	-2.11614
C	7.53954	-0.69210	0.35242
H	7.62124	-0.64282	1.44123
H	7.19898	-1.69367	0.08367
H	8.53936	-0.56040	-0.07007
C	-2.34181	-1.44259	-1.66568
C	-2.32116	-2.33186	-2.78002
C	-3.58667	-1.26056	-1.00058
C	-3.52544	-2.60525	-3.41372
C	-1.13202	-3.20492	-3.22215
C	-4.75310	-1.59272	-1.68420
C	-3.78600	-0.92527	0.49153
C	-4.75481	-2.16379	-2.94629
H	-3.49421	-3.21834	-4.30595
C	-0.72275	-3.00461	-4.68272
C	-1.60646	-4.66569	-3.05993
C	0.07241	-3.07197	-2.30744
H	-5.69773	-1.40197	-1.19399
C	-2.49829	-0.65451	1.25173
C	-4.37131	-2.20936	1.11665
C	-4.78013	0.21352	0.73977
C	-6.02377	-2.40579	-3.75307
H	0.02340	-3.75553	-4.95488
H	-1.57424	-3.12171	-5.35738
H	-0.28648	-2.02148	-4.84501
H	-2.40271	-4.92961	-3.75694
H	-0.76215	-5.33336	-3.25126
H	-1.96669	-4.85439	-2.04548
H	0.53327	-2.08821	-2.34524
H	-0.20336	-3.30829	-1.27758
H	0.84319	-3.77880	-2.61976
H	-1.76468	-1.44466	1.08076
H	-2.03607	0.30136	1.01182
H	-2.70518	-0.62958	2.32427
H	-4.50751	-2.06075	2.19151
H	-5.33759	-2.47286	0.68545
H	-3.69513	-3.05521	0.97189
H	-5.76475	-0.00314	0.32244
H	-4.90735	0.35574	1.81612
H	-4.44583	1.16188	0.31766
C	-7.26169	-1.83218	-3.06884
C	-6.23399	-3.91195	-3.94938
C	-5.87197	-1.73215	-5.12217
H	-7.46561	-2.32286	-2.11361
H	-7.16533	-0.75806	-2.89151
H	-8.13508	-1.98597	-3.70688
H	-5.40313	-4.37140	-4.48818
H	-6.33510	-4.42218	-2.98834
H	-7.14484	-4.09093	-4.52731
H	-5.73649	-0.65325	-5.01491
H	-5.01340	-2.12313	-5.67155
H	-6.76602	-1.90502	-5.72747
S	-0.37611	0.24405	-3.48899
C	-2.21269	1.37576	-1.42878
H	-2.61537	1.49980	-0.42841
H	-1.66084	2.26999	-1.71735
H	-3.02664	1.22274	-2.13958
